# Controllable Enzymatic Synthesis of Natural Asymmetric Human Milk Oligosaccharides

**DOI:** 10.1021/jacsau.4c00830

**Published:** 2024-11-02

**Authors:** Hsien-Wei Tseng, Hsin-Kai Tseng, Kai-Eng Ooi, Cheng-En You, Hung-Kai Wang, Wen-Hua Kuo, Chi-Kung Ni, Yoshiyuki Manabe, Chun-Cheng Lin

**Affiliations:** † Department of Chemistry, National Tsing Hua University, Hsinchu 30013, Taiwan; ‡ Institute of Atomic and Molecular Sciences, Academia Sinica, Taipei 10617, Taiwan; § Department of Chemistry, Graduate School of Science, Osaka University, 1-1 Machikaneyama, Toyonaka, Osaka 560-0043, Japan; ∥ Department of Medicinal and Applied Chemistry, Kaohsiung Medical University, Kaohsiung 80708, Taiwan

**Keywords:** enzymatic synthesis, human milk oligosaccharides, fucosylation, branched oligosaccharides, asymmetric glycans

## Abstract

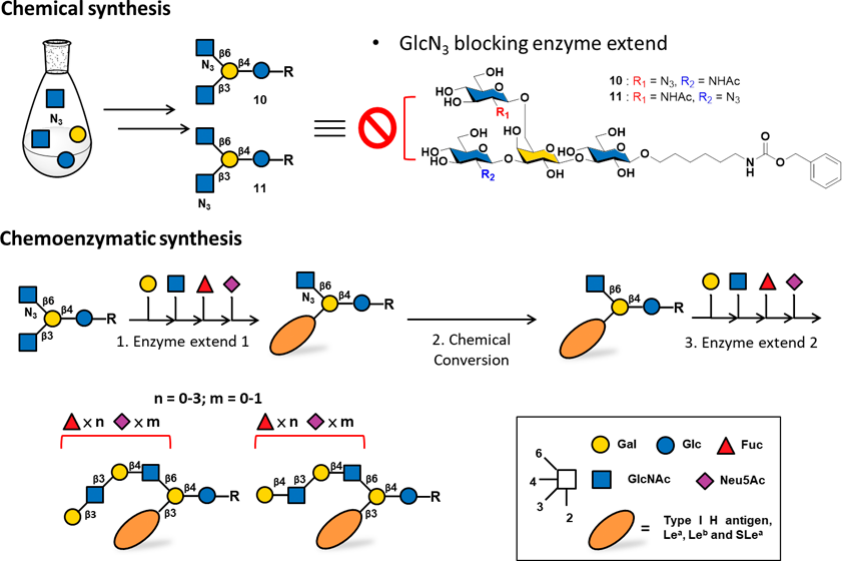

Among human milk oligosaccharides (HMOs), linear HMOs are synthesized through mature but varied routes. Although branched HMOs can be synthesized by chemical, enzymatic, or chemoenzymatic methods, these methods cannot be easily applied to the synthesis of asymmetric multiantennary oligosaccharides. Herein, we developed a controllable method to synthesize asymmetric biantennary HMOs. In our synthetic route, GlcNAcβ1,3(GlcN3β1,6)Glaβ1,4Glc was first chemically synthesized as the core tetrasaccharide, which contains β1,6GlcN3 as the “stop” sugar in transferase-catalyzed glycosylation. The desired sugars at the GlcNAcβ1–3Gal arm can be assembled using galactosyltransferase, *N*-acetylglucosaminyltransferase, and fucosyltransferase. Then, the Staudinger reduction and acetylation were used to transform GlcN3 to GlcNAc and assemble sugars by initiating the “go” process. By manipulating transferase-catalyzed glycosylations, 22 natural asymmetric biantennary oligosaccharides were synthesized.

## Introduction

Breast milk is the primary nutrient source for infants in their early stages and plays multiple roles in physiological development.^1,2^ After lactose and lipids, human milk oligosaccharides (HMOs) are the most abundant component in breast milk.^3,4^ Despite being an essential nutrient, HMOs cannot be directly digested in the infant’s gastrointestinal tract. Instead, they serve as metabolic substrates for prebiotics in the gut, promoting the proliferation of gut microbiota and contributing to the maturation of an infant’s gastrointestinal tract.^3,5^ HMOs also support the intelligence and cognitive development of infants and are involved in certain immune mechanisms.^2,6^ However, the functional roles of many HMOs remain unknown because pure and sufficient quantities of these complex materials for detailed studies are limited.

HMOs contain more than 200 oligosaccharides,^7^ and approximately 150 have well-defined structures. All HMOs begin with unconjugated lactose and extend to more intricate glycans. Lactose is elongated with *N*-acetyllactosamine (LacNAc) or lacto-*N*-biose (LNB) by a β1,3-linkage from the nonreducing end Gal of lactose. Branched HMOs are generated by incorporating LacNAc with a β1,6-linkage to the Gal of the core lactose. LacNAc also serves as a minimal unit for extending branched HMOs and is often decorated with α1,3- or α1,4- with or without α1,2-fucose or α2,3- or α2,6-sialic acid to form Lewis or blood type motifs. Due to the complexity and diversity of these structures, the direct isolation of HMOs from breast milk is inefficient, prompting the need for efficient synthetic strategies.

**Figure 1:**
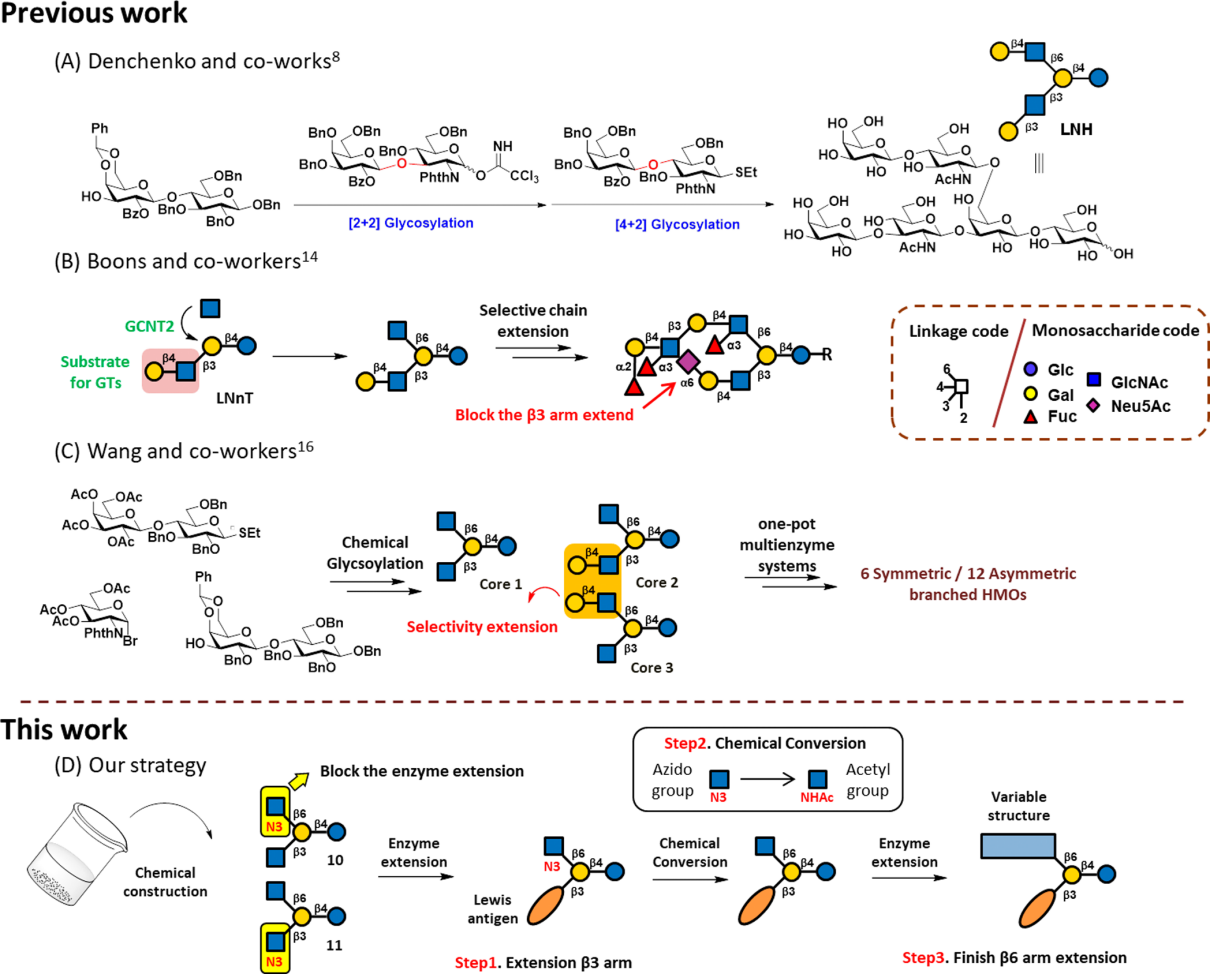
Strategies for synthesizing branched HMOs in the literature and the use of acceptor-mediated glycosylation in this work.

Methods for synthesizing linear HMOs, including chemical, enzymatic, and chemoenzymatic synthesis, have matured, leading to diverse glycan structures.^1^ In contrast, the synthesis of branched HMOs has been rare. Only a few branched HMOs, including lacto-*N*-hexaose (LNH)^8^ (Figure 1A), lacto-*N*-neohexaose (LNnH),^9^ difucosyllacto-*N*-hexaose (DF-LNH),^10^ difucosyllacto-*N*-neohexaose (DF-LNnH),^10,11^ and *iso*-lacto-*N*-octaose (*i*LNO), have been successfully generated through chemical synthesis.^12^ However, the overall yields are low due to several factors, including the manipulative steps needed for hydroxy group protection and deprotection, glycosylation, and side reactions; thus, the chemical synthesis of branched HMOs is challenging.^13^

In 2017, Boons et al. successfully synthesized asymmetric multiarmed HMOs using an enzymatic synthesis strategy.^14^ By using mammalian glycosyltransferases and leveraging GCNT2 (glucosaminyl-*N*-acetyl transferase 2, a β1,6-*N*-acetylglucosaminyltransferase) to regio-specifically form β6 branched arms, the researchers constructed asymmetric branch glycans (Figure 1B). However, this enzymatic synthesis focused on the LNnH core skeleton (β3 arm with LacNAc) and α2,6-sialic acid to terminate the sugar chain extension of the β3 arm, reducing synthetic flexibility. High manufacturing costs and low protein production yields of mammalian cell expression also limit its widespread application. In 2024, Fang et al. successfully utilized *Pichia pastoris* as host to express the recombinant GCNT2 and combining bacterial enzymes to complete the synthesis of both bi- and triantennary HMOs.^15^ In contrast, Wang et al. developed a core synthesis/enzymatic extension (CSSE) strategy that involved using chemical synthesis to prebuild asymmetric branch glycan cores (Figure 1C).^16^ After the sugar chain was extended from the unprotected arm, the other arm was deprotected and extended further. However, different core structures must be prepared in advance, reducing synthetic flexibility. In 2024, Cao et al. also synthesized a β6-branched arm tetrasaccharide through chemical method and successfully achieved selective enzymatic glycosylation to form LNB at the β3 arm using β1,3-glycosyltransferase Cvβ3GalT.^17^ A more elegant strategy for synthesizing asymmetric multiantennary *N*-glycans was reported.^18^ In this method, a “stop and go” strategy was employed by using glucosamine (GlcN) or 2-azido-2-deoxy-d-glucose (GlcAz) as capped terminal sugars, which cannot serve as acceptors for mammalian β1,4-galactosyltransferase. The amine/azide group was transformed to NHAc, transforming the resulting terminal GlcNAc into an acceptor for enzymatic galactosylation. Through this strategy, the extension of the sugar arm was easily controlled, increasing the feasibility of synthesizing asymmetric branched glycans.

We previously developed an acceptor-mediated glycosylation strategy^19^ to specifically assemble fucose at the desired GlcNAc in a poly-*N*-acetyllactosamine chain.^20^ In this study, we found that GlcAz is not recognized by bacterial β1,4- and β1,3-galactosyltransferases (GTs, Hp0826, NmLgtB, EcWbgO, and Cvβ3GalT). By coupling with the “stop and go” strategy, bacterial enzymes were applied to the synthesis of asymmetric HMOs. Although Yu et al. used chemical synthesis to construct tetrasaccharides with GlcNAc at the β6 arm and GlcN at the β3 arm, the weak activity of HP0826 for GlcN acceptors resulted in mixed extended sugar chains.^21^

Enzymatic glycan assembly can be categorized into three types based on the sugar nucleotide donor supply. The Directly Using Sugar Donor system (DUSD, Figure S1(A)) presynthesizes and purifies sugar nucleotide donors (SNDs),^22^ which allows for precise control donor amount but requires tedious SND purification steps and high costs. The One-Pot MultiEnzyme (OPME, Figure S1(B)) system^23^ or the Sequential One-Pot Enzymatic (SOPE) system^24^ generates SNDs in situ, which increased the reaction efficiency but still generates carbon waste. The Sugar Nucleotide Regeneration System (SNRS, Figure S1(C))^25−27^ uses pyruvate kinase (PK) and phosphoenolpyruvate (PEP) to recycle nucleoside diphosphate (NDP), reducing carbon waste. However, this method is not suitable for the enzymatic reactions which require specific control of fucose or sialic acid assembly. Therefore, the appropriate system should be selected based on the synthetic target. The enzymatic reaction systems (abbreviations) used in this report are outlined in Table S1.

In this study, we developed a chemoenzymatic strategy to access asymmetric HMOs (Figure 1D). Using chemical synthesis to construct intermediate compounds **10** and **11**, we focused on synthesizing branched HMOs containing LNB at the β3 arm. With GlcAz as the stop terminus, bacterial glycosyltransferases (*b*GTs) selectively extended the sugar chain, and the desired glycan sequence was constructed. The azide was then chemically converted to an *N*-acetyl group, allowing further extension by *b*GTs. This strategy enables the controlled extension of sugar chains at specific arms, making the synthesis of asymmetrically branched HMOs more flexible and efficient.

## Results and Discussion

**Scheme 1:**
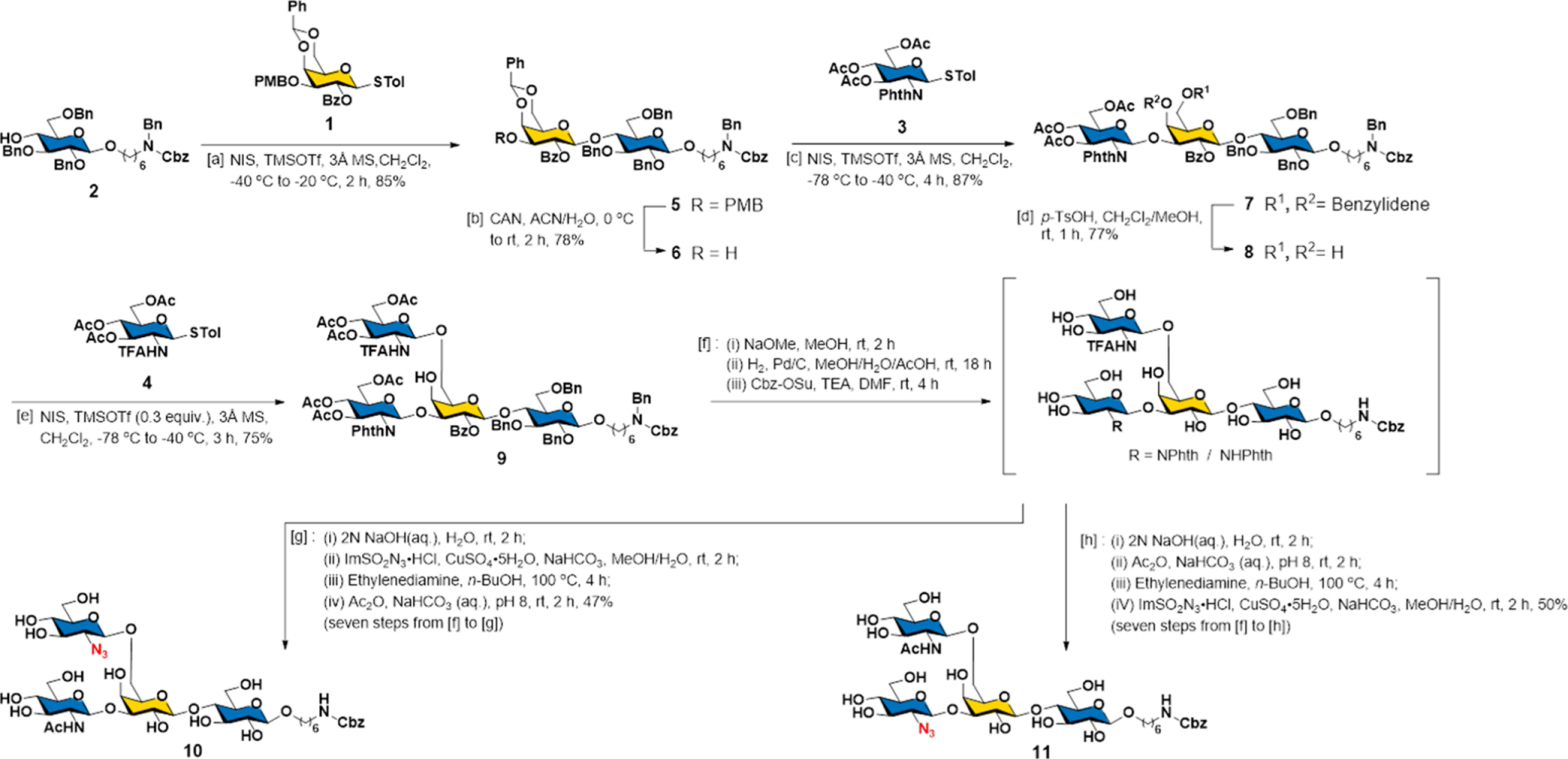
Chemical Synthesis of Core Tetrasaccharides **10** and **11**

When lactose was used to directly construct core tetrasaccharides, the results were unsuccessful (Scheme S1). Thus, to synthesize core tetrasaccharides **10** and **11** (as shown in Scheme 1), building blocks **1**,^28^**2**^29^ (Scheme S2), **3**(30) and **4**(31) were first prepared by following reported procedures. Glycosylation of acceptor **2** with donor **1** yielded disaccharide **5** in 85% yield. The removal of PMB from **5** with ceric ammonium nitrate (CAN) generated **6**, which was glycosylated with donor **3** to afford trisaccharide **7** (87%). Then, the benzylidene acetal of **7** was deprotected under acidic conditions to provide diol **8**, which was glycosylated with thiol donor **4** to generate **9** in a 75% yield. Attempts at [3 + 1] glycosylation using *N*-Boc thiol or an immediate donor (see Scheme S3) with **8** failed, likely due to Boc group instability during TMSOTf-promoted glycosylation.^32^

To install the azido group at the β3 or β6 arms, a seven-step deprotection process was designed. Initially, deacetylation of compound **9** was performed under Zemplén conditions, followed by deprotection of the benzyl ether and benzyl carbamate groups via catalytic hydrogenolysis with 10% Pd/C. The resulting amine on the linker was protected with a benzyloxycarbonyl (Cbz) group. Unexpectedly, two spots were observed via thin layer chromatography (TLC) analysis. In addition to the desired product, another product was generated, likely due to the ring-opening of the Phth group under basic conditions. Due to the difficulty of separating these intermediates, the mixture was used in subsequent reactions. Compound **10** was obtained through the following sequential deprotection steps: (i) removal of the TFA group, (ii) azidolation, (iii) removal of the Phth group, and (iv) *N*-acylation, with a total yield of 47%. Alternatively, compound **11** was synthesized by altering the deprotection sequence through the following steps: (i) removal of the TFA group, (ii) *N*-acylation, (iii) removal of the Phth group, and (iv) azidolation, with a total yield of 50%.

In this study, we focused on enzymatic synthesis starting from the β3 arm with LNB. Before extending the sugar chain from tetrasaccharide **10**, we investigated how glycosyltransferase activity is affected by the acceptor structure. We investigated four common Lewis and two sialyl-LacNAc motifs found in HMOs (Figure S2). Using either DUSD or SOPE to supply sugar donors, we examined the catalytic activities of various *b*GTs. Initially, Lewis X (Le^x^, **S8**) and Lewis A (Le^a^, **S9**) were tested with three β1,3-*N*-acetylglucosaminyltransferases (β1,3GnTs), HP1105, NmLgtA,^20^ and HpLgtA^33^ (Figure S2(A)). None of the tested β1,3GnTs could transfer Gal to Le^x^ or Le^a^ acceptors, indicating that nonreducing Fuc blocked the tested β1,3GnTs. Next, we tested α1,2-fucosyltransferase (FutC) with 6’SLN and 3′SLN (Figure S2(B)) and found that neither functioned as substrates. Finally, type 1 and type 2 H motifs (Figure S2(C,D)) were tested with three α2,3-sialyltransferases (Cst-I, PmST3, and PmST1(M144D)) and two α2,6-sialyltransferases (Psp2,6ST and Pd2,6ST). Among the α2,3-sialyltransferases, only PmST1 (M144D) accepted the tested acceptors, while both α2,6-sialyltransferases accepted the tested substrates; therefore, PmST1 (M144D), Psp2,6ST, and Pd2,6ST are insensitive to α1,2-fucose on the nonreducing end of Gal. In addition, Psp2,6ST and Pd2,6ST exhibited a preference for the type 2 H motif as an acceptor over the type 1 H motif (Figure S3).

**Scheme 2:**
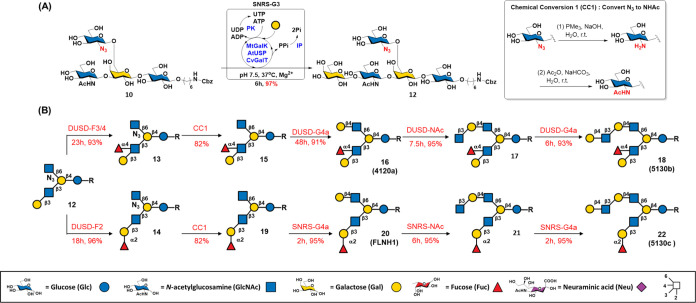
(A) Enzymatic Synthesis Initiated from the β1,3-GlcNAc Arm (β3 Arm). (B) Chemoenzymatic Synthesis of the Named HOMs **16** (4120a), **18** (5130b), **20** (FLNH1), and **22** (5130c)^,^ Please refer to Table S1 for summary of reaction systems and abbreviations. Condition: SNRS-G3 (CvGaIT, β1,3-galactosylation under sugar nucleotide regeneration system); DUSD-F3/4 (FucTa, α1,3/4-fucosylation with directly using sugar donor, GDP-Fuc as the donor); DUSD-F2 (FutC, α1,2-fucosylation with directly using sugar donor, GDP-Fuc as the donor); CC1: (i) PMe_3_, NaOH, H_2_O; (ii) Ac_2_O, NaHCO_3_, H_2_O; DUSD-G4a (NmLgtB, β1,4-galactosylation with directly using sugar donor, UDP-Gal as the donor); SNRS-G4a (NmLgtB, β1,4-galactosylation under sugar nucleotide regenereation system); DUSD-NAc (HP1105, β1,3-*N*-acetylglucosaminylation with directly using sugar donor, UDP-GlcNAc as the donor); SNRS-NAc. (HP1105, β1,3-*N*-acetylglucosaminylation under sugar nucleotide regeneration system).

Then, we extended Gal from the β3 arm of **10** (Scheme 2) using two β1,3-galactosyltransferases, EcWbgO and CvGalT, under DUSD conditions. TLC analysis (Figure S4) showed that CvGalT exhibited better efficiency than EcWbgO, completing the transformation within 5 h. The product structure (compound **12)** was confirmed by NMR spectrum analysis. The reaction was further simplified using SNRS coupled with CvGalT (denoted as SNRS-G3), yielding **12** in 97% yield (Scheme 2A). As shown in Scheme 2B, subsequent modification of **12** with Fuc using α1,3/4-fucosyltransferase (FucTa) or FutC and GDP-Fuc (denoted as DUSD-F3/4 and DUSD-F2, respectively) produced compounds **13** (93%) and **14** (96%), respectively. Previous studies have shown that the presence of Fuc inhibited the assembly of GlcNAc on the corresponding Gal acceptors. Thus, selective LacNAc elongation on the β6 arms of **13** and **14** could be achieved after N_3_ was converted to NHAc. Reduction of azide and *N*-acetylation afforded GlcNAc in 82% yield in two steps. NmLgtB and UDP-Gal (denoted as DUSD-G4a) were used to transform **15** to **16** (4120a) in 91% yield. Subsequently, β1,3-*N*-acetylglucosaminyltransferase (HP1105) and UDP-GlcNAc (denoted as DUSD-NAc) were applied to **16** (4120a) to produce **17**, followed by DUSD-G4a again to yield **18** (5130b) in 88% yield in two steps. Moreover, we investigated the *b*GT catalytic efficiency in the SNRS. Compound **19** was transformed to **20** (FLNH1) by NmLgtB (denoted as SNRS-G4a) in 95% yield and then glycosylated by HP1105 in SNRS (denoted as SNRS-NAc) to yield **21** and then SNRS-G4a to afford **22** (5130c) in 90% yield in two steps.

The reaction times between DUSD-G4a (**15** to **16**) and SNRS-G4a (**19** to **20**) ranged from 48 to 2 h. In contrast, the subsequent glycan extensions **17** to **18** and **21** to **22** did not significantly affect the reaction time. This result suggested that the structure of Le^a^ at the β3 arm generated steric hindrance in NmLgtB, potentially affecting the initial Gal modification but not subsequent glycan elongation. Overall, the SNRS showed better catalytic efficiency for enzymatic assembly of sugar, except with FucTs. As the use of SNRS conserved material and reduced the time needed to purify sugar donors, SNRS was chosen for most glycosyltransferase-catalyzed glycosylations in this study.

**Scheme 3:**
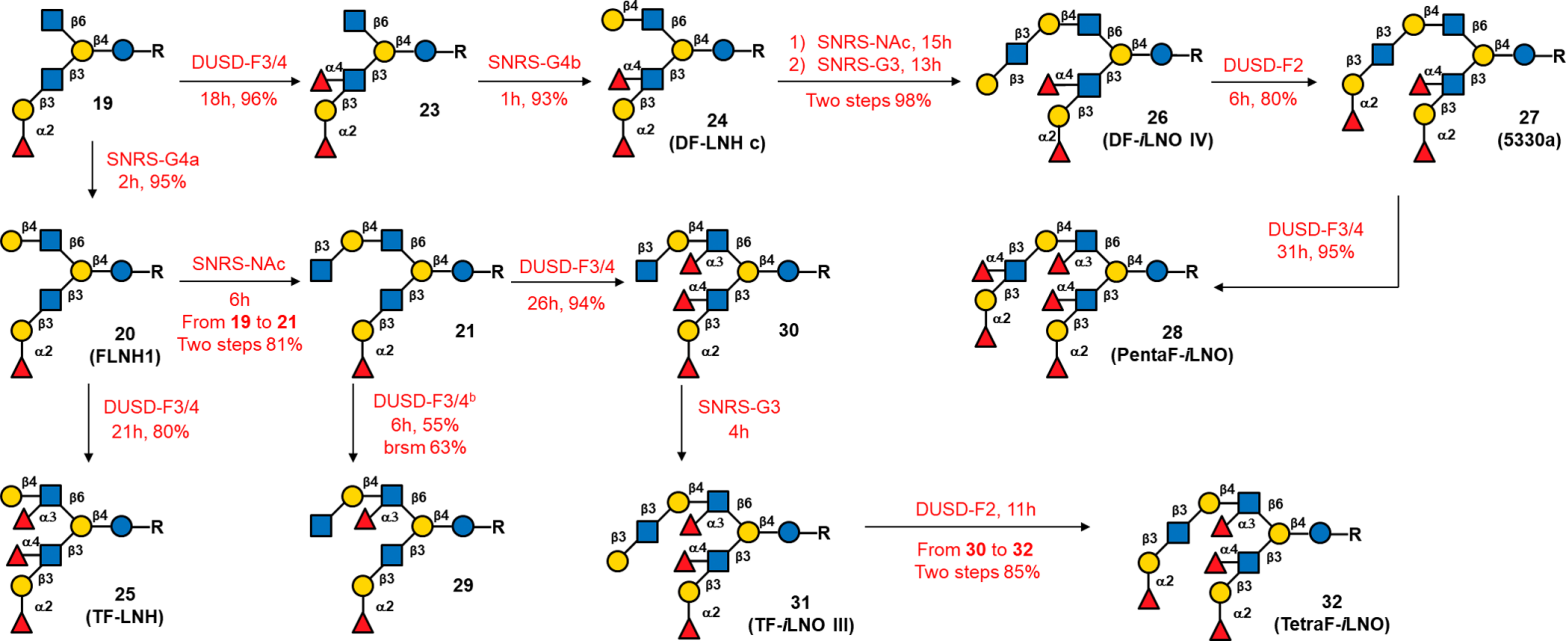
Enzymatic Synthesis of Multifucosylated LNH and *i*LNO Glycans from Compound **19**, Please refer to Table S1 for a summary of reaction systems and abbreviations. Condition: SNRS-G3 (CvGaIT, β1,3-galactosylation under sugar nucleotide regenereation system); SNRS-G4a (NmLgtB, β1,4-galactosylation under sugar nucleotide regeneration system); SNRS-G4b (HP0826, β1,4-galactosylation under sugar nucleotide regeneration system); SNRS-NAc (HP1105, β1,3-*N*-acetylglucosaminylation under sugar nucleotide regeneration system); DUSD-F2 (FutC, α1,2-fucosylation with directly using sugar donor, GDP-Fuc as the donor); DUSD-F3/4 (FucTa, α1,3/4-fucosylation with directly using sugar donor, GDP-Fuc as the donor); ^b^controling GDP-Fuc at 1.05 equiv achieve selectivity fucosylation.

In Scheme 2B, we demonstrated the feasibility of our strategy for asymmetric glycan synthesis by using GlcN3 to block *b*GT activity. Following this approach, we synthesized multifucosylated branched HMOs. In Scheme 3, the fucosylation of **19** using DUSD-F3/4 yielded the Lewis B (Le^b^) motif in compound **23** at 96%. Compound **23** was galactosylated by SNRS-G4a, resulting in **24** (DF-LNH c) in 47% yield (76% based on the recovered starting material (brsm)) in 48 h. On the other hand, fucosylation of **20** (FLNH1) by DUSD-F3/4 with 2.2 equiv of GDP-Fuc produced **25** (TF-LNH) in 80% yield. The results indicated that the enzymatic synthetic route should be carefully selected based on the acceptor structure to ensure good yield. TLC observation also revealed that FucTa preferred to transfer fucose to LacNAc over the type 1 H motif, consistent with previous reports.^34^

Previously, we found that the presence of the Le^a^ motif at the β3 arm significantly decreased the reaction rate of NmLgtB during galactosylation of the β6 arm (**15** to **16** (4120a)). A similar effect was also observed for the galactosylation of **23**, which contains a Le^b^ motif. To further increase the rate of enzymatic galactosylation, we investigated GalT HP0826 from *Helicobacter pylori*(35) and found that the activity of this enzyme was not affected by the presence of the Le^a^ or Le^b^ motif at the β3 arm (Figure S5). By using SNRS-G4b (GalT HP0826 with SNRS), **23** was transformed to **24** (DF-LNH c) in 93% yield within 1 h. Thus, for subsequent β1,4-galactosylation, GalT HP0826 was used to replace NmLgtB.

We synthesized the *i*LNO (*iso*-lacto-*N*-octaose) series of HMOs (Scheme 3) from **24** (DF-LNH c) and used SNRS-NAc and SNRS-G3 to obtain **26** (DF-*i*LNO IV) in 98% yield in two steps. Subsequently, the application of DUSD-F2 to **26** generated compound **27** (5330a) in a 80% yield. Then, DUSD-F3/4 (with 2.2 equiv of GDP-Fuc) with **27** yielded 95% of **28** (PentaF-*i*LNO). Moreover, compound **21** was further fucosylated by DUSD-F3/4 using different GDP-Fuc equivalents, producing compound **29** in 55% yield with 1.05 equiv of GDP-Fuc for 6 h and in 94% yield of compound **30** with 2.2 equiv of GDP-Fuc for 26 h. Moreover, we observed that β1,3-GlcNAc accelerated FucTa catalytic activity on compound **21**, yielding a DF product (**29**) in a shorter time (Figures S6 vs S7(A)); in contrast, at least 4 h was needed when compound **20** was applied. The ratio of DF products (**29** and **S21)** was determined by ^1^H NMR to be 10:1 (Figure S7(B)). The application of SNRS-G3 to compound **30** afforded **31** (TF-*i*LNO III) with 90%, followed by α1,2-fucosylation by DUSD-F2 to afford **32** (TetraF-*i*LNO) with 85% yield from **30** in two steps. Determining the second Fuc linkage on compound **29** (obtained from **21**) was challenging, but comparative ^1^H NMR analysis with compounds **16** (4120a), **20** (FLNH1), **24** (DF-LNH c), and **25** (TF-LNH) (Figure S8) helped identify this linkage. The chemical shifts of the anomeric protons indicated that the second Fuc was located on the GlcNAc of the β6 arm (see Figure S8 for a detailed discussion).

**Scheme 4:**
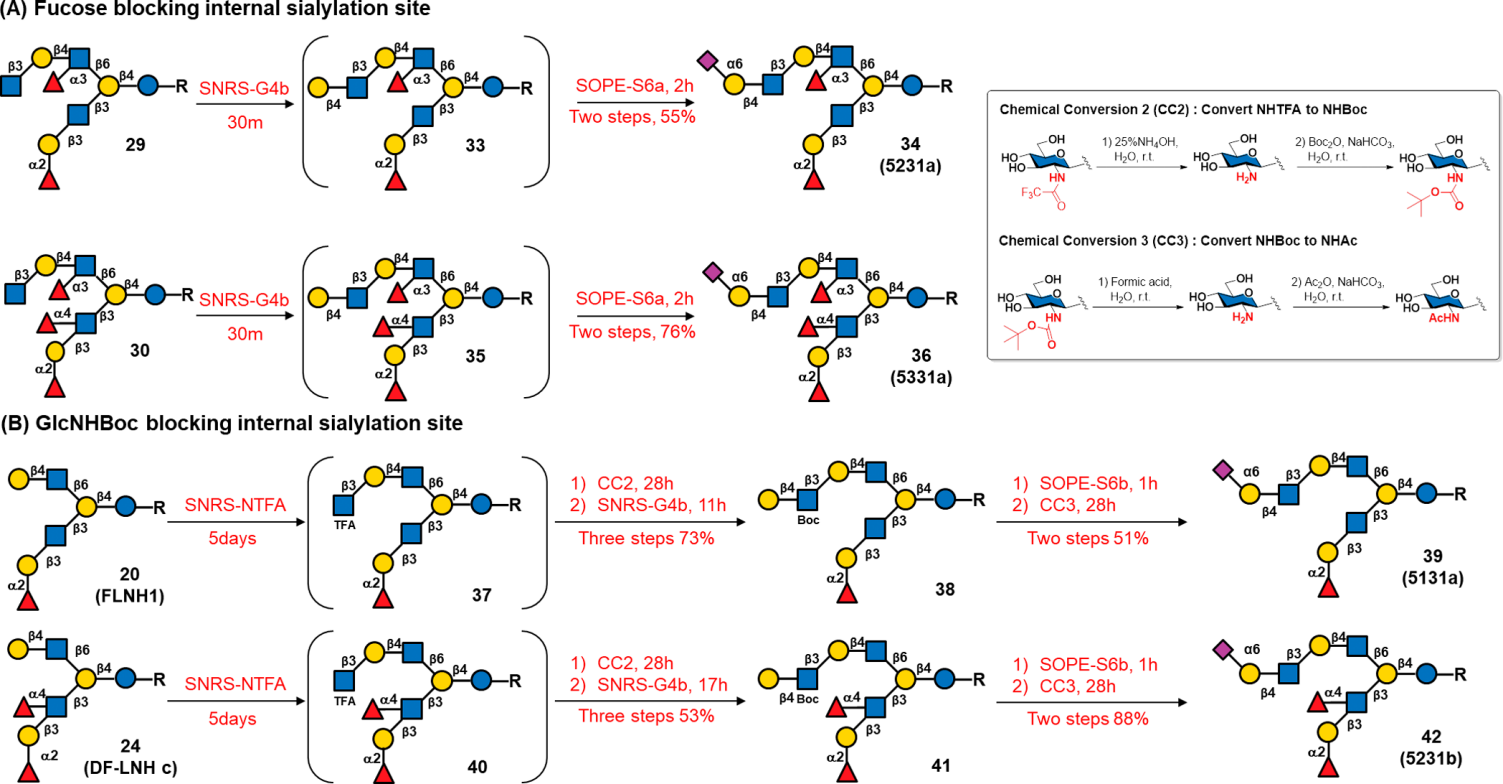
Synthesis of Sialylated HMOs^,^ Two strategies were applied to produce terminal 6′SLNs: (A) installation of Fuc on the internal GlcNAc of LNnT to guide Neu5Ac assembly on terminal Gal; and (B) use of GlcNBoc in LNnT to block the sialylation of the reducing end Gal. Please refer to Table S1 for a summary of reaction systems and abbreviations. Condition: SNRS-G4b (SNRS with HP0826); SOPE-S6a (SOPE with Psp2,6ST); SNRS-NTFA (SNRS with HP1105 using GIcNTFA as the donor precursor); chemical conversion of GIcNHTFA to GIcNHBoc (CC2): (i) 25% NH_4_OH, H_2_O; (ii) Boc_2_O, NaHCO_3_, H_2_O; chemical conversion of GIcNHBoc to GIcNAc (CC3): (i) formic acid; (ii)Ac_2_O, NaHCO_3_, H_2_O.

After successfully constructing complex multifucosyl HMOs, we attempted to synthesize asymmetric glycans featuring a Lewis-type motif at the β3 arm and a 6’SLN at the β6 arm. Four HMOs, **34** (5231a), **36** (5331a), **39** (5131a), and **42** (5231b), were chosen as synthetic targets (Scheme 4). Selective α2,6-sialylation was first needed to achieve this goal. Previous studies (Figures S2 and S3) indicated that α2,6-sialyltransferase (STs) prefer the type 2 H motif as an acceptor. Additionally, Pd2,6ST-catalyzed sialylations showed that Fuc on the GlcNAc of oligo-LacNAcs inhibited the sialylation of the subsequent nonreducing end of the Gal acceptor.^33^ As shown in Scheme 4A, compound **29** was synthesized using SNRS-G4b, yielding intermediate **33** in 86% yield. To prevent isomers from forming during SOPE sialylation, 1.05 equiv of Nue5Ac were used with Pd2,6ST or Psp2,6ST. The results (Figures S9 and S10) demonstrated that both STs could catalyze the sialylation of compound **33**, with Pd2,6ST showing slightly greater activity than that of Psp2,6ST. Given the acceptor scope (Figures S2 and S3), both enzymes tolerated α1,2-Fuc on the galactose acceptor but favored the type 2 H motif. To minimize isomer production, we used less active Psp2,6ST and 1.5 equiv of Neu5Ac to ensure that the starting material was completely consumed. After SOPE-S6a (SOPE with Psp2,6ST) was performed for 2 h, the major product was isolated in 64% yield. NMR spectroscopy confirmed that a single Neu5Ac signal was present, and 2D NMR spectroscopy confirmed that Neu5Ac was linked to the C6 position of the terminal Gal at the β6 arm, verifying the structure of **34** (5231a). Using a similar strategy as that applied for compound **30**, we obtained **36** (5331a) in 76% yield over two steps without other sialylated isomers.

Previous studies have shown that GlcNHBoc in oligo-LacNAcs blocks the glycosylation activity of FucTa on GlcNHBoc.^20^ Further exploration revealed that GlcNHBoc also inhibits α2,6-sialyltransferase activity (Pd2,6ST and Psp2,6ST) on the reducing end Gal of GlcNBoc (unpublished results). This reactivity characteristic was utilized in the synthesis of **39** (5131a) and **42** (5231b) (Scheme 4B). Compound **20** (FLNH1) was glycosylated using SNRS with HP1105 and GlcNHTFA as donor precursors (denoted as SNRS-NTFA) to yield compound **37** (Scheme 4B). Then, TFA was removed under weakly basic conditions, and the resulting amine was transformed to NHBoc with Boc_2_O under alkaline conditions (this process is denoted CC2). The Boc-protected saccharide was glycosylated under SNRS-G4b to generate compound **38** in 73% yield over three steps. Sialylation of **38** under SOPE-S6b (SOPE with Pd2,6ST) followed by Boc deprotection under acidic conditions and subsequent acetylation (this process was denoted as CC3) afforded **39** (5131a) in 51% yield over two steps. Through a similar process, **42** (5231b) was obtained with a higher overall yield (47% over five steps from **24**). Although TLC analysis revealed that each step involved a quantitative transformation, some products were lost during purification. The previous strategy of using sugar as a blocking element^33^ was not feasible for the synthesis of these compounds.

When **20** and **24** were modified with GlcNAc (yielding **21** and **S21**, respectively, Figure S11) or GlcNHTFA (yielding **37** and **40**, respectively, Scheme 4B) using SNRS, longer reaction times were needed for GlcNHTFA as the donor precursor (5 days vs 6–15 h, Figure S11). These results suggested that UDP-GlcNHTFA was not the preferred donor for HP1105. In contrast, the reaction rates of α2,6-sialylations on GlcNHBoc- and GlcNHAc-modified glycans were similar (2 h vs 1 h, Scheme 4B), indicating that GlcNHBoc does not hinder α2,6-sialyltransferase activity on terminal Gal. However, the type 1 H motif (of compound **38**) was a better acceptor for Psp2,6ST (more spots were observed via TLC, Figure S10), while Pd2,6ST provided better selectivity for the α2,6-sialylation of **38**. Thus, Psp2,6ST was used for synthesizing compounds **34** and **36**, while Pd2,6ST was used for synthesizing compounds **39** and **42**.

**Scheme 5:**
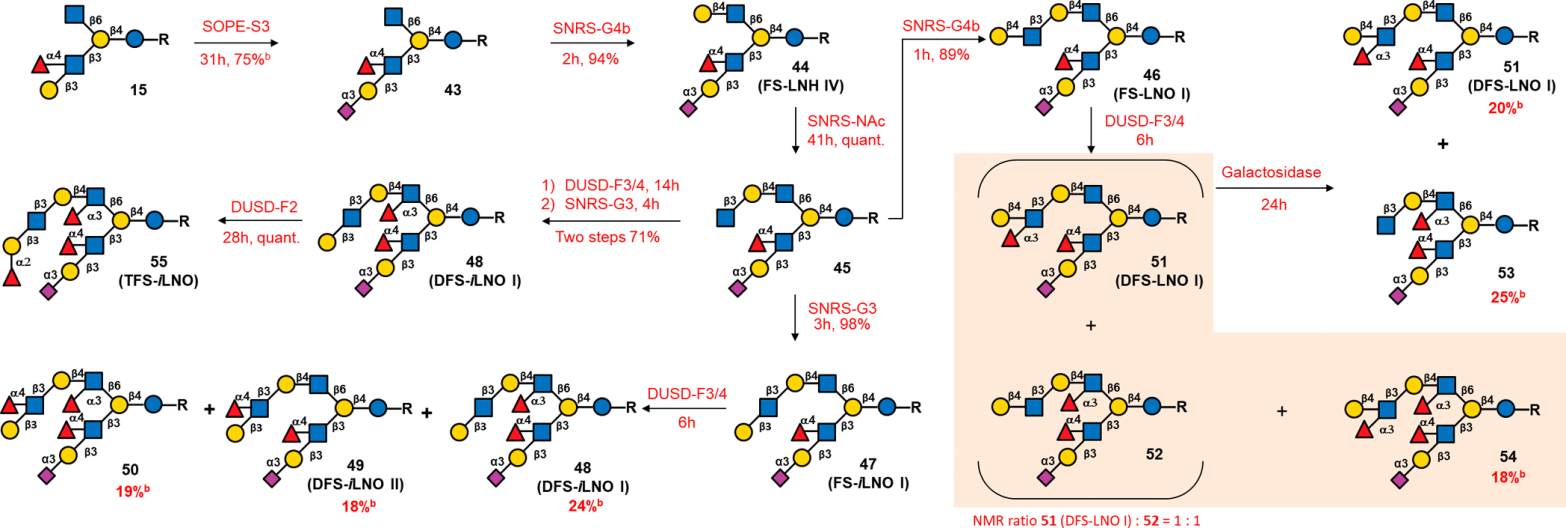
Synthesis of SLe^a^-Based HMOs Condition: SOPE-S3 (PmST1(M144D), Sequential one-pot enzymatic α2,3-sialylation); SNRS-G4b (HP0826, β1,4-galactosylation under sugar nucleotide regeneration system); SNRS-NAc (HP1105, β1,3-*N*-acetylglucosaminylation under sugar nucleotide regeneration system); SNRS-G3 (CvGaIT, β1,3-galactosylation under sugar nucleotide regeneration system); DUSD-F3/4 (FucTa, α1,3/4-fucosylation with directly using sugar donor, GDP-Fuc as the donor); DUSD-F2 (FutC, α1,2-fucosylation with directly using sugar donor, GDP-Fuc as the donor); ^b^purification by HPLC-HILIC to get separation yield.

Next, we focused on constructing HMOs with an SLe^a^ motif at the β3 arm (compound **43**, Scheme 5). It was previously reported that the α2,3-sialyltransferase Cst-I could recognize the Le^a^ motif in linear oligo-LacNAcs to form a Neu5Acα2,3Gal glycosidic bond;^36,37^ however, the use of Cst-I in SOPE on branched compound **13** (Figure S12) was unsuccessful, indicating that Cst-I could not tolerate an acceptor with internally branched sugars. Thus, we tested another α2,3ST, PmST1 (M144D), which has major α2,3ST activity and minor α2,6ST activity.^19^ Using PmST1 (M144D), compound **13** was sialylated, resulting in two regioisomers (**S25**(α2,3)/**S26**(α2,6) = 3:1, determined by ^1^H NMR analysis) with a yield of 73% (brsm 98%, Figure S12(A)). Attempts to separate these isomers using HPLC-HILIC were unsuccessful. Therefore, compound **15** (the azide of **13** was transformed to NHAc) was sialylated by PmST1(M144D), forming a mixture of the desired α2,3 sialylated compound **43** and the α2,6 isomer **S27** (ratio 7.2 (74.4%): 1 (7.5%), Figure S12(B)), which could be separated by HPLC-HILIC. We also tested PmST3, another α2,3ST from *Pasteurella multocida*,^38^ but it was ineffective, and the presence of an azido group reduced the reaction rate (70 vs 31 h, Figure S12).

After observing the remote azide effect, which slows the catalytic efficiency of enzymes, we decided to investigate the impact of the azide on fucosyltransferases. Thus, compounds **12** and **S28** (Figure S13) were used as acceptors for FucTa- and FutC-catalyzed reactions in the DUSD.

The results showed that the presence of an azide at the β6 arm reduced the reactivity of both FucTa and FutC. In the FucTa-catalyzed reaction, compound **S28** with GlcNAc at the β6 arm was fully consumed within 2 h, while compound **12** with GlcN3 at the β6 arm remained partially unreacted. Similar trends were observed with FutC, which convert compound **S28** in 18 h, while compound **12** remained half unreacted (Figure S13).

As shown in Scheme 5, compound **43** was subjected to SNRS-G4b, yielding **44** (FS-LNH IV) in 94% yield, followed by SNRS-NAc to afford compound **45** in quantitative yield. Further application of SNRS-G4b and SNRS-G3 to **45** yielded **46** (FS-LNO I) in 89% yield and **47** (FS-*i*LNO I) in 98% yield. Fucosylation of compound **45** by DUSD-F3/4 followed by SNRS-G3 afforded **48** (DFS-*i*LNO I) in 71% yield over two steps. Surprisingly, the SLe^a^ moiety at the β3 arm did not hinder FucTa in the fucosylation of the internal GlcNAc at the β6 arm. Using **46** and **47** as acceptors with 1.5 equiv of GDP-Fuc for fucosylation resulted in the production of difucosylated saccharides (DFS isomers) and a trifucosylated saccharide (TFS), indicating that FucTa did not exhibit regioselectivity for the LNT and LNnT sequences in the β6 arm glycan. The DFS and TFS products from **47** (FS-*i*LNO I) were separated by HPLC-HILIC to afford 24% of **48** (DFS-*i*LNO I**)**, 18% of **49** (DFS-*i*LNO II), and 19% of **50**. Conversely, the DFS products **51** (DFS-LNO I) and **52** from **46** (FS-LNO I) could not be separated by HILIC-HPLC. The observed phenomenon suggested that **48** and **49** exhibited different structures at the β6 arm, resulting in distinct interactions within the HILIC column and leading to different retention times. However, the similar structures of **51** and **52** at the β6 arm provided similar interactions in the HILIC column. To differentiate the structures and test the resistance of the Le^x^ structure to galactosidase hydrolysis, the DFS products (**51** and **52**) were incubated with β1,4-galactosidase (from *Aspergillaceae oryzae*) for 24 h. As expected, only compound **52** was hydrolyzed to compound **53**. The resulting mixture was separated to yield 25% **53**, 20% **51**, and 18% TFS **54**. Finally, **48** was fucosylated by DUSD-F2 to yield **55** (TFS-*i*LNO) in quantitative yield.

The glycan microarray was prepared using our previous Cu_2_O@Ag slides,^39^ which provide a metal-enhanced fluorescence effect to increase the detection sensitivity; in addition, the slides enable surface Cu_2_O nanoparticles (NPs) to catalyze alkyne–azide cycloaddition. To facilitate the fabrication of the glycan microarray, the benzyloxycarbonyl (Cbz) group in the synthesized HMOs was deprotected under hydrogenation conditions and subsequently transformed into an azide (Scheme S4). These azido glycans, along with some simple glycans (Figure S14), were printed on Cu_2_O@Ag slides to construct the glycan microarray. Although HMOs are crucial for the development of the infant immune system, nervous system, and gut microbiome, there are currently few studies on the interactions between related GBPs and branched HMOs.^14,40−42^ Although galectins are known as HMO receptors, the binding specificities of galectins for branched HMOs remain unclear.^40^ Herein, human galectins, dendritic cell-specific intercellular adhesion molecule-3-grabbing nonintegrin (DC-SIGN), and virus hemagglutinin were used to evaluated the their binding profiles with synthetic HMOs.

**Figure 2:**
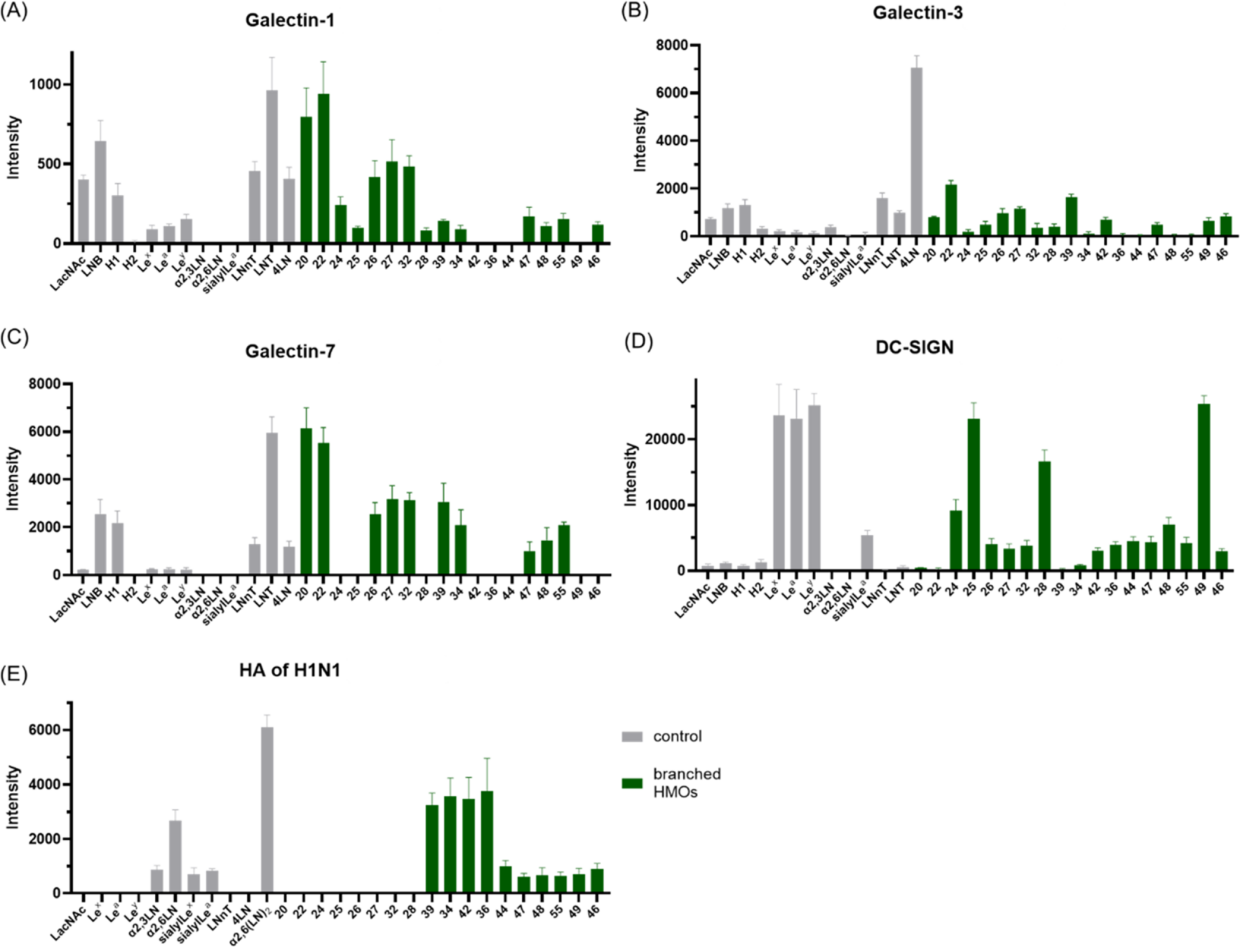
Binding results of HMOs on the microarray with lectins. (A) hGal-1 (25 μg/mL), (B) hGal-3 (25 μg/mL), (C) hGal-7 (25 μg/mL), (D) DC-SIGN (20 μg/mL), and (E) HA from H1N1 A/California/04/2009 (25 μg/mL) (also see Figure S15).

Human galectin-1 (hGal-1) plays a critical role in tumor progression and participates in processes such as invasion, angiogenesis, and metastasis.^43^ hGal-1 is a dimeric carbohydrate binding protein (exotype lectin) that predominantly engages with the terminal LacNAc unit within a poly-LacNAc chain.^44^ As shown in Figure 2A, the results demonstrated that LNB exhibited a greater binding affinity for disaccharide than did LacNAc, and the addition of lactose at the reduced end (resulting in LNT and LNnT, respectively) further increased the binding affinity. The extension of LacNAc did not increase the affinity, suggesting that the terminal LNB residue was crucial for the hGal-1 interaction. The addition of fucose and/or NeuNAc (type 1 H (H1), type 2 H (H2), Le^x^, Le^a^, Le^y^, α2,3-LN, α2,6-LN, and SLe^a^) dramatically decreased the binding affinity.

Among the evaluated branched HMOs, compound **22** (5130c), which contains diLacNAc and a type 1 H motif, exhibited the strongest binding affinity (comparable to that of LNT). Compound **20** (FLNH1), which contains LacNAc and a type 1 H motif, showed slightly lower affinity than **22** (5130c) but higher affinity than most linear glycans, indicating that glycans at the β3 arm are crucial for the binding of hGal-1. The results also revealed that terminal Neu5Ac significantly diminished binding affinity, and the internal/terminal Fuc exhibited a similar but less effective impact (see the Supporting Information for a detailed discussion).

Human galectin-3 (hGal-3) participates in many biological functions, such as immune response regulation,^45^ T-cell apoptosis induction,^46^ and bone development.^47^ In contrast to hGal-1, hGal-3, a chimera-type galectin, binds tightly to glycans with 3–4 repeating LacNAc units. The binding strength is further intensified when the terminal β-Gal residue is decorated with α1,3-Gal/GalNAc or α1,2-Fuc residues.^48^ The glycan microarray binding profile of hGal-3, which is shown in Figure 2B, revealed that 4 LN exhibited the strongest binding signal. The stronger affinity of LNnT compared to LNT suggested that LacNAc is crucial for this interaction, which is consistent with previous studies.^48^ Among the synthetic branched HMOs investigated, only **22** (5130c), which features diLacNAc and a type 1 H motif, displayed a notable binding affinity; however, this affinity was only slightly greater than that of the linear tetrasaccharide LNnT, indicating the importance of oligo-LacNAc for binding.

Human galectin-7 (hGal-7) is crucial for epidermal homeostasis, skin repair, cancer progression, and metastasis.^49^ Recent studies have revealed that hGal-7 plays a role in the innate immune system through specifically binding to the blood group B motif present in various species.^50^ Previous studies have shown that hGal-7 preferentially binds to LNB, shorter poly-LacNAc, and blood group H motifs.^40,51,52^ The glycan microarray binding results of linear HMOs were consistent with the literature, with LNT showing the strongest binding affinity. The binding tendency of branched HMOs was similar to that of hGal-1 (Figure 2A,C). Branched HMOs **20** (FLNH1) and its β6 arm extended derivative **22** (5130c) exhibited the strongest binding with hGal-7. Intriguingly, further extension of LNB (with Fuc) at the β6 arm (compounds **26** (DF-*i*LNO IV), **27** (5330a), and **32** (TetraF-*i*LNO) vs **24** (DF-LNH c)) restored hGal-7 binding.

DC-SIGN, which was identified through its affinity for gp120 on the HIV envelope and function as a Ca^2+^-dependent myeloid C-type lectin, plays a crucial role in innate immune interactions with viral glycoproteins. Although the binding specificity of DC-SIGN has been extensively examined for various glycoconjugates,^53^ its preference for binding to structurally well-defined branched HMOs remains uncertain. As depicted in Figure 2D, DC-SIGN shows good affinity for linear HMOs containing the α1,3/4-Fuc motif (Le^x^, Le^a^, and Le^y^), consistent with previous reports.^41^ However, in branched HMOs, only glycans with a β6 arm containing the terminal Le^x^, Le^a^, or Le^y^ motif provided good binding affinity (compounds **25** (TF-LNH), **28** (PentaF-*i*LNO), and **49** (DFS-*i*LNO II)), indicating that the longer glycan present in the β6 arm decreased the binding affinity.

The initial step of virus infection by epidemic influenza virus involves the attachment of hemagglutinin (HA) to sialic acid on the host cell. Sialylated branched HMOs were also found to be HA receptors.^42^ Moreover, exoenzymatic fucosylation on live cells increases susceptibility to influenza A (IVA) infection.^54,55^ Herein, the interaction of HA from H1N1 A/California/04/2009, which exhibits a preference for binding poly-LacNAc terminated with α2,6-sialic acid,^56^ with synthetic branched HMOs containing multiple spatial fucoses was investigated (Figure 2E). As expected, only the glycans with α2,6-NeuNAc showed good binding affinities for HA. Linear α2,6-sialyl diLacNAc provided the strongest binding signal, while α2,6-sialyl-LacNAc demonstrated medium affinity. Notably, branched HMOs **34** (5231a), **36** (5331a), **39** (5131a), and **42** (5231b), featuring the α2,6-sialyl diLacNAc moiety, displayed good binding affinity with the HA of H1N1; however, the binding affinity was weaker than that of their linear counterparts. As expected, FS-series glycans, which feature the sLe^a^ motif at their β3 arm, exhibited weak binding with this HA.

## Conclusions

In summary, we utilized chemical synthesis to construct a conversion intermediate and employed a strategy that combines *N* masked glucosamine to hinder transferase catalytic activity. We successfully developed a controllable chemoenzymatic synthetic strategy for the production of asymmetric biantennary HMOs. In our approach, GlcN3 on the β6 arm of the core tetrasaccharide was used to block β1,4-galactosyltransferase activity. Thus, the enzymatic extension of the glycan epitope could be controlled at the β3 arm. After the β3 arm was constructed, GlcN3 was converted to GlcNAc, which could be recognized by galactosyltransferase for further extension. Thus, different glycan epitopes could be assembled. Our results demonstrated that this strategy is very flexible and can be used to synthesize asymmetric branched HMOs. Using this strategy, we synthesized a series of β3 arm asymmetric biantennary HMOs with an LNB core motif (22 total HMOs) to demonstrate the feasibility of the developed strategy for accessing most asymmetric biantennary HMOs. The glycan microarray binding results indicated that variations in epitopes at both arms of branched HMOs spatially influence binding affinities. Our results showed that hGal-1 and hGal-7 were better receptors for these fucosylated branched HMOs than hGal-3, which exhibits a strong preference for poly-LacNAc. We observed that these synthetic HMOs did not exhibit multivalent interactions with DC-SIGN or HA. However, these natural HMOs can be further modified for biomedical applications in the future by combining various glycosidases or GTs for further derivatization as specific ligands.

## Supporting Information Available

Detailed experimental procedures; purification of enzyme; enzymatic reaction conditions; analysis of 1D and 2D NMR spectra; characterization of new compounds; and ^1^H NMR and ^13^C NMR spectra (PDF)

## References

[ref1] Xu L. L.; Townsend S. D. Synthesis as an Expanding Resource in Human Milk Science. J. Am. Chem. Soc. 2021, 143, 11277–11290. 10.1021/jacs.1c05599. 34296874 PMC12010734

[ref2] Zhu L.; Li H.; Luo T.; Deng Z.; Li J.; Zheng L.; Zhang B. Human Milk Oligosaccharides: A Critical Review on Structure, Preparation, Their Potential as a Food Bioactive Component, and Future Perspectives. J. Agric. Food. Chem. 2023, 71, 15908–15925. 10.1021/acs.jafc.3c04412. 37851533

[ref3] Kassai S.; de Vos P. Gastrointestinal barrier function, immunity, and neurocognition: The role of human milk oligosaccharide (hMO) supplementation in infant formula. Compr. Rev. Food. Sci. Food Saf. 2024, 23, e13271 10.1111/1541-4337.13271. 38284595

[ref4] Thurl S.; Munzert M.; Boehm G.; Matthews C.; Stahl B. Systematic review of the concentrations of oligosaccharides in human milk. Nutr. Rev. 2017, 75, 920–933. 10.1093/nutrit/nux044. 29053807 PMC5914348

[ref5] Shao Y.; Garcia-Maurino C.; Clare S.; Dawson N. J. R.; Mu A.; Adoum A.; Harcourt K.; Liu J.; Browne H. P.; Stares M. D.; Rodger A.; Brocklehurst P.; Field N.; Lawley T. D. Primary succession of Bifidobacteria drives pathogen resistance in neonatal microbiota assembly. Nat. Microbiol. 2024, 9, 2570–2582. 10.1038/s41564-024-01804-9. 39242817 PMC11445081

[ref6] Henrick B. M.; Rodriguez L.; Lakshmikanth T.; Pou C.; Henckel E.; Arzoomand A.; Olin A.; Wang J.; Mikes J.; Tan Z.; Chen Y.; Ehrlich A. M.; Bernhardsson A. K.; Mugabo C. H.; Ambrosiani Y.; Gustafsson A.; Chew S.; Brown H. K.; Prambs J.; Bohlin K.; Mitchell R. D.; Underwood M. A.; Smilowitz J. T.; German J. B.; Frese S. A.; Brodin P. Bifidobacteria-mediated immune system imprinting early in life. Cell 2021, 184, 3884–3898. 10.1016/j.cell.2021.05.030. 34143954

[ref7] Chen X. Human Milk Oligosaccharides (HMOS): Structure, Function, and Enzyme-Catalyzed Synthesis. In Advances in Carbohydrate Chemistry and Biochemistry; Elsevier, 2015; Vol. 72, pp 113–190. 26613816 10.1016/bs.accb.2015.08.002 PMC9235823

[ref8] Bandara M. D.; Stine K. J.; Demchenko A. V. Chemical Synthesis of Human Milk Oligosaccharides: Lacto-N-hexaose Galβ1–3GlcNAcβ1–3 [Galβ1–4GlcNAcβ1–6] Galβ1–4Glc. J. Org. Chem. 2019, 84, 16192–16198. 10.1021/acs.joc.9b02701. 31749363 PMC8114087

[ref9] Bandara M. D.; Stine K. J.; Demchenko A. V. Chemical synthesis of human milk oligosaccharides: lacto-N-neohexaose (Galβ1–4GlcNAcβ1-)23,6Galβ1–4Glc. Org. Biomol. Chem. 2020, 18, 1747–1753. 10.1039/D0OB00172D. 32048706 PMC7658587

[ref10] Kim H. M.; Kim I. J.; Danishefsky S. J. Total syntheses of tumor-related antigens N3: probing the feasibility limits of the glycal assembly method. J. Am. Chem. Soc. 2001, 123, 35–48. 10.1021/ja0022730. 11273599

[ref11] Lee J. C.; Wu C. Y.; Apon J. V.; Siuzdak G.; Wong C. H. Reactivity-based one-pot synthesis of the tumor-associated antigen N3 minor octasaccharide for the development of a photocleavable DIOS-MS sugar array. Angew. Chem., Int. Ed. 2006, 45, 2753–2757. 10.1002/anie.200504067. 16548041

[ref12] Knuhr P.; Castro-Palomino J.; Grathwohl M.; Schmidt R. R. Complex structures of antennary human milk oligosaccharides - Synthesis of a branched octasaccharide. Eur. J. Org. Chem. 2001, 2001, 4239–4246. 10.1002/1099-0690(200111)2001:22<4239::AID-EJOC4239>3.0.CO;2-M.

[ref13] Krasnova L.; Wong C. H. Oligosaccharide Synthesis and Translational Innovation. J. Am. Chem. Soc. 2019, 141, 3735–3754. 10.1021/jacs.8b11005. 30716271 PMC6538563

[ref14] Prudden A. R.; Liu L.; Capicciotti C. J.; Wolfert M. A.; Wang S.; Gao Z.; Meng L.; Moremen K. W.; Boons G. J. Synthesis of asymmetrical multiantennary human milk oligosaccharides. Proc. Natl. Acad. Sci. U.S.A. 2017, 114, 6954–6959. 10.1073/pnas.1701785114. 28630345 PMC5502611

[ref15] Li Y.; Li Y.; Guo Y.; Chen C.; Yang L.; Jiang Q.; Ling P.; Wang S.; Li L.; Fang J. Enzymatic modular synthesis of asymmetrically branched human milk oligosaccharides. Carbohydr. Polym. 2024, 333, 121908 10.1016/j.carbpol.2024.121908. 38494200

[ref16] Xiao Z.; Guo Y.; Liu Y.; Li L.; Zhang Q.; Wen L.; Wang X.; Kondengaden S. M.; Wu Z.; Zhou J.; Cao X.; Li X.; Ma C.; Wang P. G. Chemoenzymatic Synthesis of a Library of Human Milk Oligosaccharides. J. Org. Chem. 2016, 81, 5851–5865. 10.1021/acs.joc.6b00478. 27305319 PMC5953189

[ref17] Xia H.; Zhong K.; Li Y.; Ye J.; Wang D.; Cai C.; Mu W.; Liu C.-C.; Cao H. Regioselective Enzymatic Galactosylation Enabled Divergent Synthesis of Asymmetrical Biantennary Human Milk Oligosaccharides. ACS Catal. 2024, 14, 13390–13399. 10.1021/acscatal.4c03373.

[ref18] Liu L.; Prudden A. R.; Capicciotti C. J.; Bosman G. P.; Yang J. Y.; Chapla D. G.; Moremen K. W.; Boons G. J. Streamlining the chemoenzymatic synthesis of complex N-glycans by a stop and go strategy. Nat. Chem. 2019, 11, 161–169. 10.1038/s41557-018-0188-3. 30532014 PMC6347513

[ref19] Tseng H. K.; Su Y. Y.; Chang T. W.; Liu H. C.; Li P. J.; Chiang P. Y.; Lin C. C. Acceptor-mediated regioselective enzyme catalyzed sialylation: chemoenzymatic synthesis of GAA-7 ganglioside glycan. Chem. Commun. 2021, 57, 3468–3471. 10.1039/D1CC00653C. 33688902

[ref20] Tseng H.-K.; Wang H.-K.; Wu C.-Y.; Ni C.-K.; Lin C.-C. Exploring Regioselective Fucosylation Catalyzed by Bacterial Glycosyltransferases through Substrate Promiscuity and Acceptor-Mediated Glycosylation. ACS Catal. 2023, 13, 10661–10671. 10.1021/acscatal.3c01563.

[ref21] Ooi K. E.; Zhang X. W.; Kuo C. Y.; Liu Y. J.; Yu C. C. Chemoenzymatic Synthesis of Asymmetrically Branched Human Milk Oligosaccharide Lacto-N-Hexaose. Front. Chem. 2022, 10, 905105 10.3389/fchem.2022.905105. 35711960 PMC9194828

[ref22] Li S.; Wang S. S.; Wang Y. Q.; Qu J. Y.; Liu X. W.; Wang P. G.; Fang J. Q. Gram-scale production of sugar nucleotides and their derivatives. Green Chem. 2021, 23, 2628–2633. 10.1039/D1GC00711D.

[ref23] Yu H.; Chen X. One-pot multienzyme (OPME) systems for chemoenzymatic synthesis of carbohydrates. Org. Biomol. Chem. 2016, 14, 2809–2818. 10.1039/C6OB00058D. 26881499 PMC4795158

[ref24] Chien W. T.; Liang C. F.; Yu C. C.; Lin C. H.; Li S. P.; Primadona I.; Chen Y. J.; Mong K. K.; Lin C. C. Sequential one-pot enzymatic synthesis of oligo-*N*-acetyllactosamine and its multi-sialylated extensions. Chem. Commun. 2014, 50, 5786–5789. 10.1039/C4CC01227E. 24756160

[ref25] Tsai T. I.; Lee H. Y.; Chang S. H.; Wang C. H.; Tu Y. C.; Lin Y. C.; Hwang D. R.; Wu C. Y.; Wong C. H. Effective sugar nucleotide regeneration for the large-scale enzymatic synthesis of Globo H and SSEA4. J. Am. Chem. Soc. 2013, 135, 14831–14839. 10.1021/ja4075584. 24044869

[ref26] Wu H. R.; Anwar M. T.; Fan C. Y.; Low P. Y.; Angata T.; Lin C. C. Expedient assembly of Oligo-LacNAcs by a sugar nucleotide regeneration system: Finding the role of tandem LacNAc and sialic acid position towards siglec binding. Eur. J. Med. Chem. 2019, 180, 627–636. 10.1016/j.ejmech.2019.07.046. 31351394

[ref27] Anwar M. T.; Kawade S. K.; Huo Y. R.; Adak A. K.; Sridharan D.; Kuo Y. T.; Fan C. Y.; Wu H. R.; Lee Y. S.; Angata T.; Lin C. C. Sugar nucleotide regeneration system for the synthesis of Bi- and triantennary N-glycans and exploring their activities against siglecs. Eur. J. Med. Chem. 2022, 232, 114146 10.1016/j.ejmech.2022.114146. 35149460

[ref28] Zhang Z. Y.; Ollmann I. R.; Ye X. S.; Wischnat R.; Baasov T.; Wong C. H. Programmable one-pot oligosaccharide synthesis. J. Am. Chem. Soc. 1999, 121, 734–753. 10.1021/ja982232s.

[ref29] Hsu C. H.; Chu K. C.; Lin Y. S.; Han J. L.; Peng Y. S.; Ren C. T.; Wu C. Y.; Wong C. H. Highly alpha-selective sialyl phosphate donors for efficient preparation of natural sialosides. Chem. - Eur. J. 2010, 16, 1754–1760. 10.1002/chem.200903035. 20066711

[ref30] Grann Hansen S.; Skrydstrup T. Studies directed to the synthesis of oligochitosans–preparation of building blocks and their evaluation in glycosylation studies. Eur. J. Org. Chem. 2007, 2007, 3392–3401. 10.1002/ejoc.200700048.

[ref31] Forman A.; Pfoh R.; Eddenden A.; Howell P. L.; Nitz M. Synthesis of defined mono-de-N-acetylated β(1–6)-N-acetyl-d-glucosamine oligosaccharides to characterize PgaB hydrolase activity. Org. Biomol. Chem. 2019, 17, 9456–9466. 10.1039/C9OB02079A. 31642455

[ref32] Zhang A. J.; Russell D. H.; Zhu J. P.; Burgess K. A method for removal of BOC protecting groups from substrates on TFA-sensitive resins. Tetrahedron Lett. 1998, 39, 7439–7442. 10.1016/S0040-4039(98)01631-1.

[ref33] Ye J. F.; Xia H.; Sun N.; Liu C. C.; Sheng A. R.; Chi L. L.; Liu X. W.; Gu G. F.; Wang S. Q.; Zhao J.; Wang P.; Xiao M.; Wang F. S.; Cao H. Z. Reprogramming the enzymatic assembly line for site-specific fucosylation. Nat. Catal. 2019, 2, 514–522. 10.1038/s41929-019-0281-z.

[ref34] Rasko D. A.; Wang G.; Palcic M. M.; Taylor D. E. Cloning and characterization of the α(1,3/4) fucosyltransferase of Helicobacter pylori. J. Biol. Chem. 2000, 275, 4988–4994. 10.1074/jbc.275.7.4988. 10671538

[ref35] Namdjou D. J.; Chen H. M.; Vinogradov E.; Brochu D.; Withers S. G.; Wakarchuk W. W. A. β-1,4-galactosyltransferase from Helicobacter pylori is an efficient and versatile biocatalyst displaying a novel activity for thioglycoside synthesis. ChemBioChem 2008, 9, 1632–1640. 10.1002/cbic.200700775. 18491328

[ref36] Tsai T.-W.; Fang J.-L.; Liang C.-Y.; Wang C.-J.; Huang Y.-T.; Wang Y.-J.; Li J.-Y.; Yu C.-C. Exploring the Synthetic Application of Helicobacter pylori α1,3/4-Fucosyltransferase FucTIII toward the Syntheses of Fucosylated Human Milk Glycans and Lewis Antigens. ACS Catal. 2019, 9, 10712–10720. 10.1021/acscatal.9b03752.

[ref37] Hou K. L.; Chiang P. Y.; Lin C. H.; Li B. Y.; Chien W. T.; Huang Y. T.; Yu C. C.; Lin C. C. Water-Soluble Sulfo-Fluorous Affinity (SOFA) Tag-Assisted Enzymatic Synthesis of Oligosaccharides. Adv. Synth. Catal. 2018, 360, 2313–2323. 10.1002/adsc.201800085.

[ref38] Thon V.; Li Y.; Yu H.; Lau K.; Chen X. PmST3 from *Pasteurella multocida* encoded by Pm1174 gene is a monofunctional α2–3-sialyltransferase. Appl. Microbiol. Biotechnol. 2012, 94, 977–985. 10.1007/s00253-011-3676-6. 22075637

[ref39] Fan C.-Y.; Kawade S. K.; Adak A. K.; Cho C.; Tan K.-T.; Lin C.-C. Silver-Coated Cu2O Nanoparticle Substrates for Surface Azide–Alkyne Cycloaddition. ACS Appl. Nano Mater. 2021, 4, 1558–1566. 10.1021/acsanm.0c03046.

[ref40] Noll A. J.; Gourdine J. P.; Yu Y.; Lasanajak Y.; Smith D. F.; Cummings R. D. Galectins are human milk glycan receptors. Glycobiology 2016, 26, 655–669. 10.1093/glycob/cww002. 26747425 PMC4847615

[ref41] Noll A. J.; Yu Y.; Lasanajak Y.; Duska-McEwen G.; Buck R. H.; Smith D. F.; Cummings R. D. Human DC-SIGN binds specific human milk glycans. Biochem. J. 2016, 473, 1343–1353. 10.1042/BCJ20160046. 26976925 PMC4875834

[ref42] Yu Y.; Mishra S.; Song X.; Lasanajak Y.; Bradley K. C.; Tappert M. M.; Air G. M.; Steinhauer D. A.; Halder S.; Cotmore S.; Tattersall P.; Agbandje-McKenna M.; Cummings R. D.; Smith D. F. Functional glycomic analysis of human milk glycans reveals the presence of virus receptors and embryonic stem cell biomarkers. J. Biol. Chem. 2012, 287, 44784–44799. 10.1074/jbc.M112.425819. 23115247 PMC3531791

[ref43] Astorgues-Xerri L.; Riveiro M. E.; Tijeras-Raballand A.; Serova M.; Neuzillet C.; Albert S.; Raymond E.; Faivre S. Unraveling galectin-1 as a novel therapeutic target for cancer. Cancer Treat. Rev. 2014, 40, 307–319. 10.1016/j.ctrv.2013.07.007. 23953240

[ref44] Nagae M.; Yamaguchi Y. Three-dimensional structural aspects of protein-polysaccharide interactions. Int. J. Mol. Sci. 2014, 15, 3768–3783. 10.3390/ijms15033768. 24595239 PMC3975366

[ref45] Díaz-Alvarez L.; Ortega E. The Many Roles of Galectin-3, a Multifaceted Molecule, in Innate Immune Responses against Pathogens. Mediators Inflammation 2017, 2017, 9247574 10.1155/2017/9247574. PMC5457773 28607536

[ref46] Yang R. Y.; Hsu D. K.; Liu F. T. Expression of galectin-3 modulates T-cell growth and apoptosis. Proc. Natl. Acad. Sci. U.S.A. 1996, 93, 6737–6742. 10.1073/pnas.93.13.6737. 8692888 PMC39096

[ref47] Iacobini C.; Fantauzzi C. B.; Pugliese G.; Menini S. Role of Galectin-3 in Bone Cell Differentiation, Bone Pathophysiology and Vascular Osteogenesis. Int. J. Mol. Sci. 2017, 18, 2481 10.3390/ijms18112481. 29160796 PMC5713447

[ref48] Stowell S. R.; Arthur C. M.; Mehta P.; Slanina K. A.; Blixt O.; Leffler H.; Smith D. F.; Cummings R. D. Galectin-1, −2, and −3 exhibit differential recognition of sialylated glycans and blood group antigens. J. Biol. Chem. 2008, 283, 10109–10123. 10.1074/jbc.M709545200. 18216021 PMC2442294

[ref49] Advedissian T.; Deshayes F.; Viguier M. Galectin-7 in Epithelial Homeostasis and Carcinomas. Int. J. Mol. Sci. 2017, 18, 2760 10.3390/ijms18122760. 29257082 PMC5751359

[ref50] Wu S. C.; Kamili N. A.; Dias-Baruffi M.; Josephson C. D.; Rathgeber M. F.; Yeung M. Y.; Lane W. J.; Wang J.; Jan H. M.; Rakoff-Nahoum S.; Cummings R. D.; Stowell S. R.; Arthur C. M. Innate immune Galectin-7 specifically targets microbes that decorate themselves in blood group-like antigens. iScience 2022, 25, 104482 10.1016/j.isci.2022.104482. 35754739 PMC9218387

[ref51] Brewer C. F. Thermodynamic binding studies of galectin-1, −3 and −7. Glycoconjugate J. 2002, 19, 459–465. 10.1023/B:GLYC.0000014075.62724.d0. 14758069

[ref52] Ho A. D.; Wu S. C.; Kamili N. A.; Blenda A. V.; Cummings R. D.; Stowell S. R.; Arthur C. M. An Automated Approach to Assess Relative Galectin-Glycan Affinity Following Glycan Microarray Analysis. Front. Mol. Biosci. 2022, 9, 893185 10.3389/fmolb.2022.893185. 36032675 PMC9403319

[ref53] Guo Y.; Feinberg H.; Conroy E.; Mitchell D. A.; Alvarez R.; Blixt O.; Taylor M. E.; Weis W. I.; Drickamer K. Structural basis for distinct ligand-binding and targeting properties of the receptors DC-SIGN and DC-SIGNR. Nat. Struct. Mol. Biol. 2004, 11, 591–598. 10.1038/nsmb784. 15195147

[ref54] Hong S.; Grande G.; Yu C.; Chapla D. G.; Reigh N.; Yang J. Y.; Yang Y.; Izumori K.; Moremen K. W.; Xie J.; Wu P. hFUT1-Based Live-Cell Assay To Profile α1–2-Fucoside-Enhanced Influenza Virus A Infection. ACS Chem. Biol. 2020, 15, 819–823. 10.1021/acschembio.9b00869. 32271008 PMC7521629

[ref55] Hong S.; Shi Y.; Wu N. C.; Grande G.; Douthit L.; Wang H.; Zhou W.; Sharpless K. B.; Wilson I. A.; Xie J.; Wu P. Bacterial glycosyltransferase-mediated cell-surface chemoenzymatic glycan modification. Nat. Commun. 2019, 10, 1799 10.1038/s41467-019-09608-w. 30996301 PMC6470217

[ref56] Bradley K. C.; Jones C. A.; Tompkins S. M.; Tripp R. A.; Russell R. J.; Gramer M. R.; Heimburg-Molinaro J.; Smith D. F.; Cummings R. D.; Steinhauer D. A. Comparison of the receptor binding properties of contemporary swine isolates and early human pandemic H1N1 isolates (Novel 2009 H1N1). Virology 2011, 413, 169–182. 10.1016/j.virol.2011.01.027. 21353280

