# Supplementary material for: Controllable Enzymatic Synthesis of Natural Asymmetric Human Milk Oligosaccharides
Source: JACS Au. 2024 Nov 2;4(11):4496–506. doi: 10.1021/jacsau.4c00830 (PMC11600167; doi:10.1021/jacsau.4c00830)
Supplement: Supplementary file 1 — au4c00830_si_001.pdf [file au4c00830_si_001.pdf]

Supporting information

Controllable Enzymatic Synthesis of Natural Asymmetric Human Milk Oligosaccharides

Hsien-Wei Tseng,[a] Hsin-Kai Tseng,[a] Kai-Eng Ooi,[a] Cheng-En You,[a] Hung-Kai Wang,[a] Wen-Hua Kuo,[a] Chi-Kung Ni,[b] Yoshiyuki Manabe,[c] Chun-Cheng Lin*,[a,d]

[a]Department of Chemistry, National Tsing Hua University, Hsinchu 30013, Taiwan

[b]Institute of Atomic and molecular Sciences, Academia Sinica, Taipei 10617, Taiwan

[c]Department of Chemistry, Graduate School of Science, Osaka University, 1-1 Machikaneyama, Toyonaka, Osaka 560-0043, Japan

[d]Department of Medicinal and Applied Chemistry, Kaohsiung Medical University, Kaohsiung 80708, Taiwan

*E-Mail: cclin66@mx.nthu.edu.tw

Table of Contents

Materials and method......4

Cloning and Overexpression of Enzyme......5

Scheme S1. An inefficient synthetic route the preparation of compound **S3** as a precursor for the synthesis of compounds **10** and **11**......5

Secheme S2. Synthesis of building block **2.**......6

Scheme S3. Synthesis of glycosyl donors **S6** and **S7.**......6

Figure S1. Enzymatic reaction systems. 7

Figure S2. Investigating the acceptor tolerance of the glycosyltransferase using various terminal epitopes......7

Figure S3. The acceptor preference of α2,6-sialyltransferase (Psp26ST and Pd26ST)......8

Figure S4. β1,3-Galactosyltransferase (CvGalT and WbgO) catalytic activity on compound **10**......8

Figure S5. NmLgtB and HP0826 were respectively used under SNRS to catalyze β1,4-galactosylation of **23**.. 9

Figure S6. Transformation of **20** (FLNH1) to **25** (TF-LNH) by using 2.2 equivalent of GDP-Fuc and FucTa. 9

Figure S7. Compound **21** as the acceptor for FucTa catalyzed fucosylation......10

Figure S8. Structure determination of compound **29** by ^{1}H NMR spectra......10

Figure S9. α2,6-Sialylation of compound **33** by Pd2,6ST and Psp2,6ST, respectively.. 11

Figure S10. α2,6-Sialylation of compound **38** by Pd2,6ST and Psp2,6ST, respectively.. 12

Figure S11. Effect of GlcNAc and GlcNHTFA as the donor in SNRS with to HP1105......12

Figure S12. Effect of azide at β6 arm on construction of SLe^{a} motif at β3 arm by α2,3sialyltransferases.. 13

Figure S13. Effect of azide at β6 arm on fucosylation at β3 arm by fucosyltransferases...... 13

Table S1. Enzymatic reaction systems and abbreviations for assembling sugars and chemical

conversion of N-modified glucosamine......14

General Procedures for enzymatic reactions......15

General Procedures for Chemical Conversion...... 19

General Purification Procedure......21

Synthetic procedures and characterization of new compounds 22

Scheme S4. General Procedure for conversion NHCbz to Azide 63

Table S2. The synthetic yields and m/z ESI-MS data of azido functionalized branched HMOs......64

Fabrication of Glycan microarray......65

Figure S14. Additional glycan structures on glycan microarray......65

Glycan microarry binding assay with GBPs......65

Table S3. Microarray Data......67

Figure S15. The binding profiles of different concentrations of GBPs with branched HMO microarray......68

Detailed discussion of glycan microarray binding results with Galectins and DC-SIGN......69

Reference 70

NMR Spectra......73

Materials and method

All solvents were dried and distilled by standard techniques. *N*,*N*-Dimethylformamide (DMF) was vacuum-distilled over sodium hydride. Pyridine was distilled over sodium hydride. Tetrahydrofuran (THF) was distilled from sodium under nitrogen (N_2). Dichloromethane (DCM), toluene, and acetonitrile (ACN) were distilled from calcium hydride under N_2. The chemicals for the synthesis were all obtained from Acros, Merck, Fluka, or Sigma-Aldrich and used without further purification unless otherwise noted.

All reactions were carried out in oven-dried glassware (104 °C) and performed under anhydrous conditions with N_2 unless indicated otherwise. The reactions were monitored by analytical thin-layer chromatography (TLC) on Merck silica gel 60 F_{254} plates (0.25 mm). Detection was accomplished by examination under UV light (254 nm) and by staining with *p*-anisaldehyde, ninhydrin, cerium molybdate, or potassium permanganate staining solution. Silica gel column chromatography was performed using a forced flow of the indicated solvent on silica gel 60 (Merck). Size exclusion column chromatography was performed by gravity on polymethacrylic polymer beads (Toyopearl HW-40S) with deionized H_2O and polyacrylamide gel (Bio-Gel P-2) with deionized H_2O. C18 reverse-phase silica column (Sep-Pak Vac C18 cartridge 20 cc/5 g 55-105 µm, Waters) was used to perform reverse-phase column chromatography with methanol, acetonitrile, and deionized H_2O. Capto DEAE Sepharose (Cytiva) was used for anion exchange chromatography. ^{1}H and ^{13}C NMR spectra were recorded by Bruker AV-400, AV-600, AV-850, Varian-Unity INOVA-500, or VNMRS-700. Chemical shifts are expressed in ppm using residual $CDCl_3$ (7.24 ppm), CD_3OD (3.31 or 4.87 ppm), or D_2O (4.79 ppm at 298 K) as internal standard in ^{1}H-NMR spectra. ^{13}C-NMR spectra were recorded in either $CDCl_3$, CD_3OD, $(CD_3)_2SO$ or D_2O at 100, 125, 150, 176, or 214 MHz, using the central resonances of $CDCl_3$ (77.0 ppm), CD_3OD (49.0 ppm), and $(CD_3)_2SO$ (39.5 ppm) as the internal references. 2D NMR (COSY, HSQC, HMBC, or HSQC-TOCSY) experiments were used to assist assignment of the products. Multiplicities are reported by using the following abbreviations: s = singlet, d = doublet, t = triplet, q = quartet, m = multiplet, br = broad; *J* = coupling constant values are expressed in Hertz. Mass spectra were obtained in ESI mode.

Pyruvate kinase (PK) from rabbit muscle was purchased from Calzyme. Alkaline phosphatase from calf intestinal was purchased from Sigma-Aldrich. Chemical competent *E. coli* BL21(DE3) were purchased from Yeastern Biotechnology (Taipei, Taiwan). Matrixes of bacterial cultural media were purchased from BD Bioscience (San Jose, CA, USA). IMPACT™ system (<u>I</u>ntein <u>M</u>ediated <u>P</u>urification with <u>A</u>ffinity <u>C</u>hitin binding <u>T</u>ag) was purchased from New England Biolabs. His-tag purification resin was purchased from Roche (Basel, Switzerland). Amylose resin was purchased from Cyrusbioscience (New Taipei City, Taiwan). Protein molecular weight standards and the *Pfu* DNA polymeras were purchased from Thermo Fisher Scientific (Waltham, MA, USA). The protein concentrations were

determined with the Bradford Protein Assay (Bio-Rad) and Pierce™ BCA protein assay kit (Thermo Fisher Scientific) using bovine serum albumin as the standard. Protein purification devices were using centrifugal filter devices (Vivaspin® Turbo 15 10 kDa MWCO, Satorius).

Cloning and Overexpression of Enzyme

Enzyme Resources. BlNahK (*N*-acetylhexosamine 1-kinase from *Bifidobacterium longum*),[1] AGX1 (recombinant human UDP-GalNAc pyrophosphorylase),[2] NmLgtA (*N*-acetylglucosaminyltransferase from *Neisseria meningitidis*),[3] HP1105 (β1,3-*N*-acetyl-glucosaminyltransferase from *H. pylori* ATCC 26695),[4] MtGalK (galactokinase from *Meiothermus taiwanensis* sp. nov. WR-220),[5] AtUSP (uridine diphosphate-sugar pyrophosphorylase from *Arabidopsis thaliana*),[6] NmGalT (β1,4-galactosyltransferase from *Neisseria meningitidis*),[1] HpGalT (β1,4-galactosyltransferase encoded from *hp0826* gene from *H. pylori* NCTC 11637),[4] EcWbgO (β1,3-galactosyltransferase from *E. coli* O55:H7),[7] Cvβ3GalT (β1,3-galactosyltransferase from *Chromobacterium violaceum*),[8] IP (inorganic pyrophosphatase from *E. coli* MG1655 ATCC 700926),[9] BfFKP (fucokinase/L-fucose-1-P-guanylyltransferase from *B. fragilis* NCTC 9343),[10] FucTa (α1,3/1,4- fucosyltransferase from *H. pylori* UA948),[11] FutC (α1,2-fucosyltransferase from *H. pylori* ATCC 26695),[12] NmCSS (CMP-sialic acid synthetase from *Neisseria meningitidis*),[13] PmST1 (N-terminal amino acid 2-25 truncated α(2,3)-sialyltransferase 1 from *Pasteurella multocida*),[14] PmST3 (α2,3-sialyltransferase 3 from *Pasteurella multocida* Pm70)[15], PmST1(M144D) (M144D mutant α2,3-sialyltransferase 1 from *Pasteurella multocida*)[16], and CjCst-I (α(2,3)- sialyltransferase from *Campylobacter jejuni*),[17] were cloned, overexpressed, and purified as previously reported procedures.

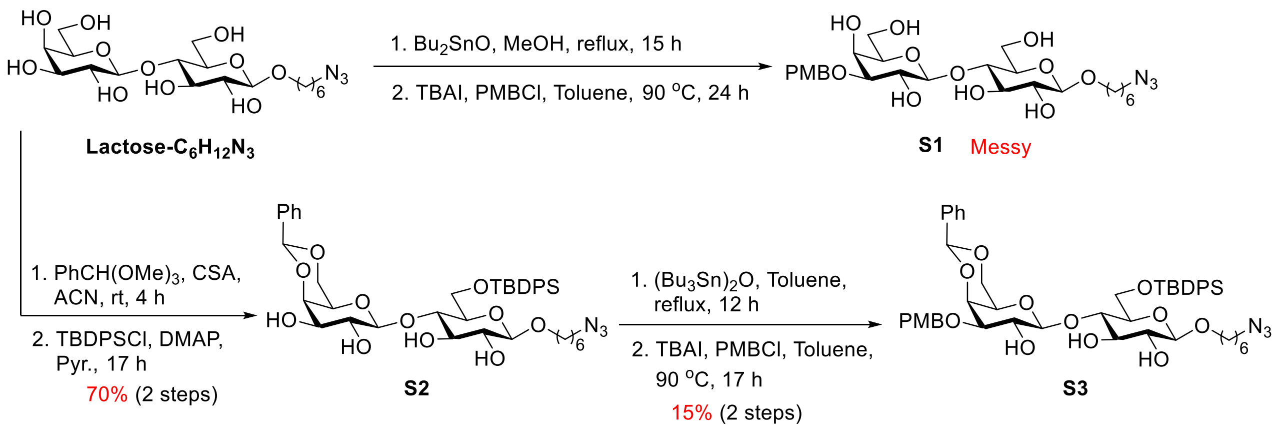

Scheme S1. An inefficient synthetic route the preparation of compound **S3** as a precursor for the synthesis of compounds **10** and **11**.

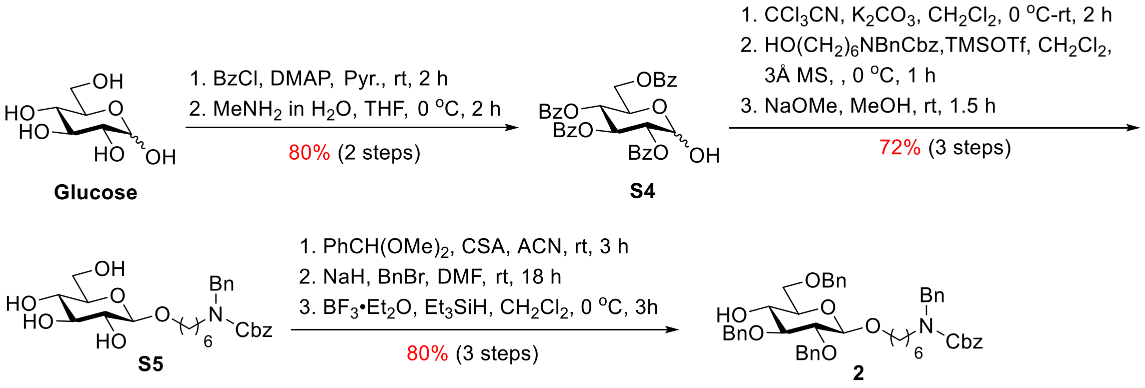

Scheme S2. Synthesis of building block **2**.

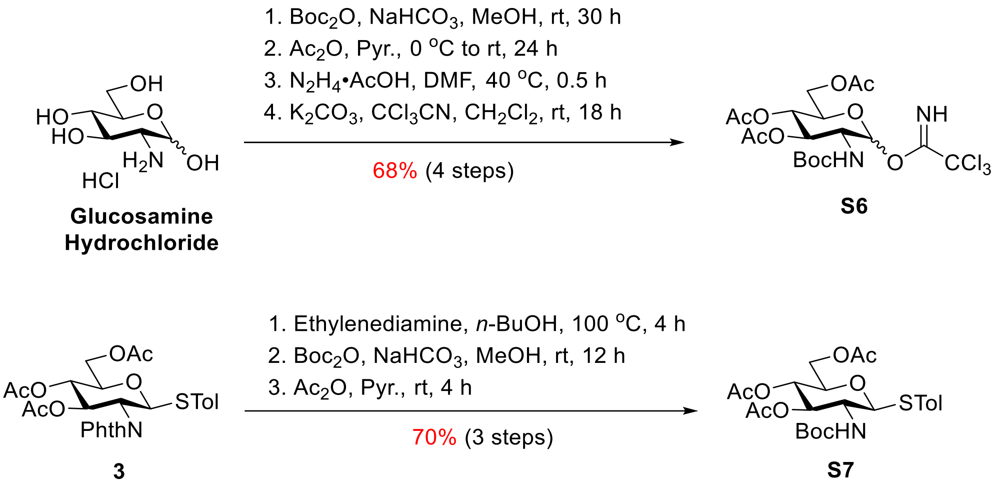

Scheme S3. Synthesis of glycosyl donors **S6** and **S7**.

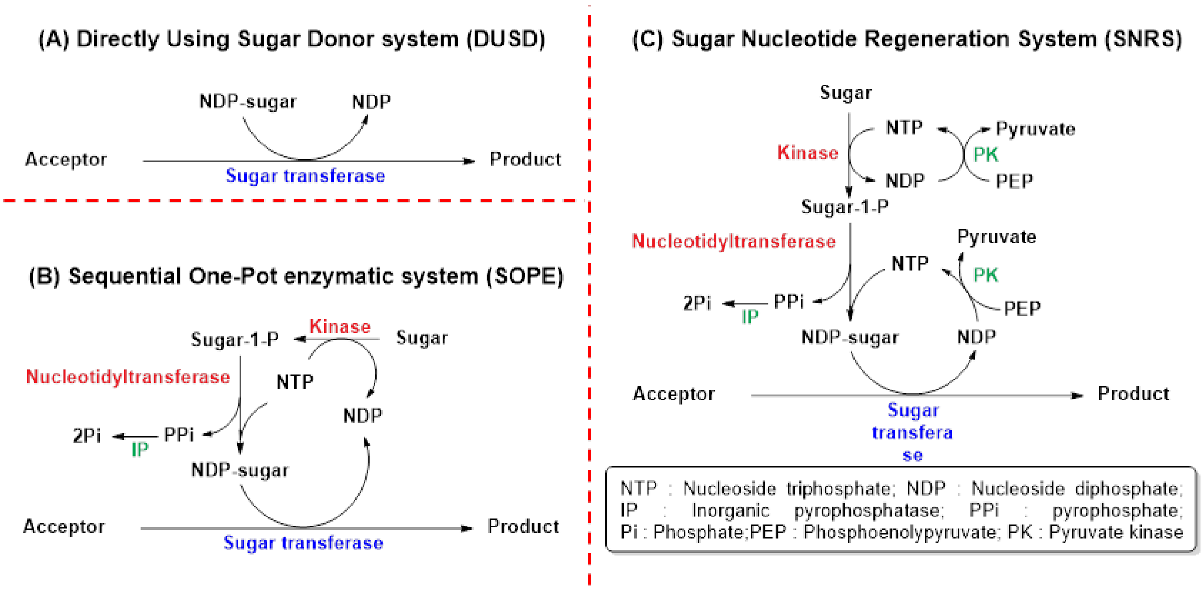

Figure S1. Enzymatic reaction systems. The glycans were synthesized by an appropriate system. (A) Directly Using Sugar Donor system (DUSD): In this reaction system, pure NDP-sugar donor was used for transferase catalyzed glycosylation. (B) Sequential One-Pot Enzymatic system (SOPE): In this reaction system, kinase and nucleotidyltransferase were first used to generate NDP-sugar donor. Then, glycosyl transferase and acceptor were added into the same reaction flask. (C) Sugar Nucleotide Regeneration System (SNRS): In this reaction system, all the enzymes and materials were incubated in the same reaction flask at the same time by using catalytic amount of ATP and UTP. While the production of the product, the released NDPs were transformed to NTPs which were used to product sugar-1-phosphate and UDP-sugar donor.

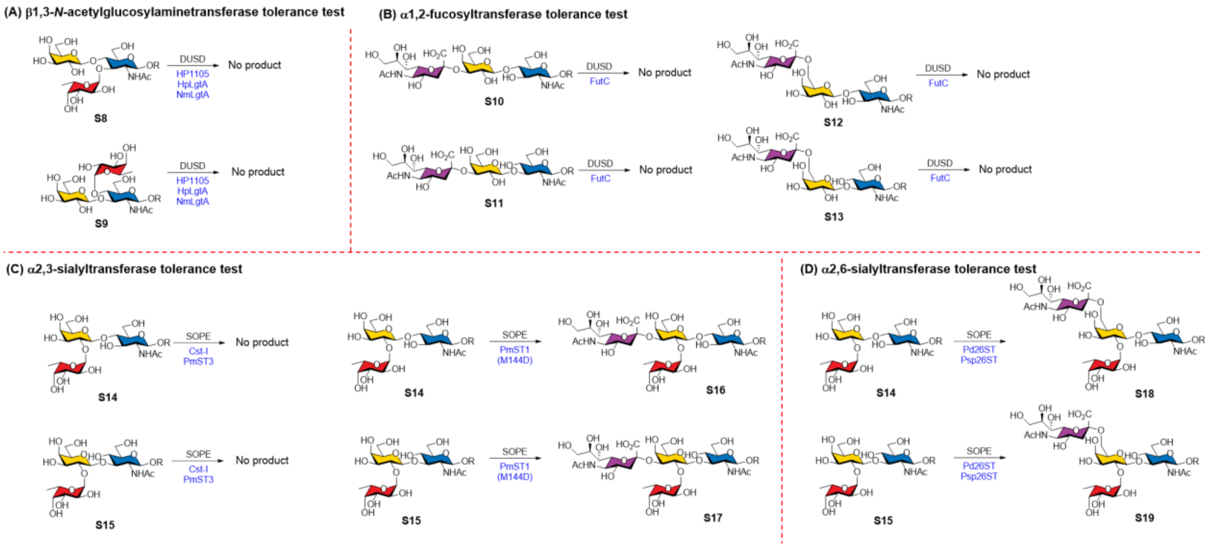

Figure S2. Investigating the acceptor tolerance of the glycosyltransferase using various terminal epitopes. (A) Lewis X (Le^{x}, **S8**) and Lewis A (Le^{a}, **S9**) were not acceptors for β1,3-*N*-acetylglucosaminyltransferases (HP1105 and NmLgtA); (B) Neither 3'SLNs (Type II **S10** and Type I **S11**) nor 6'SLNs (Type II **S12** and Type I **S13**) could be accepted by α1,2-fucosyltransferase (FutC); (C) H antigen (Type II **S14** and Type I **S15**) couldn't be the acceptor for α2,3-sialyltransferases Cst-I and PmST3 but could be recognized by PmST1(M144D) to produce compounds **S16** and **S17**, respectively. (D) H antigens (**S14** and **S15**) were the acceptors of α2,6-sialyltransferases Pd26ST and Psp26ST to yield compound **S18** or **S19**.

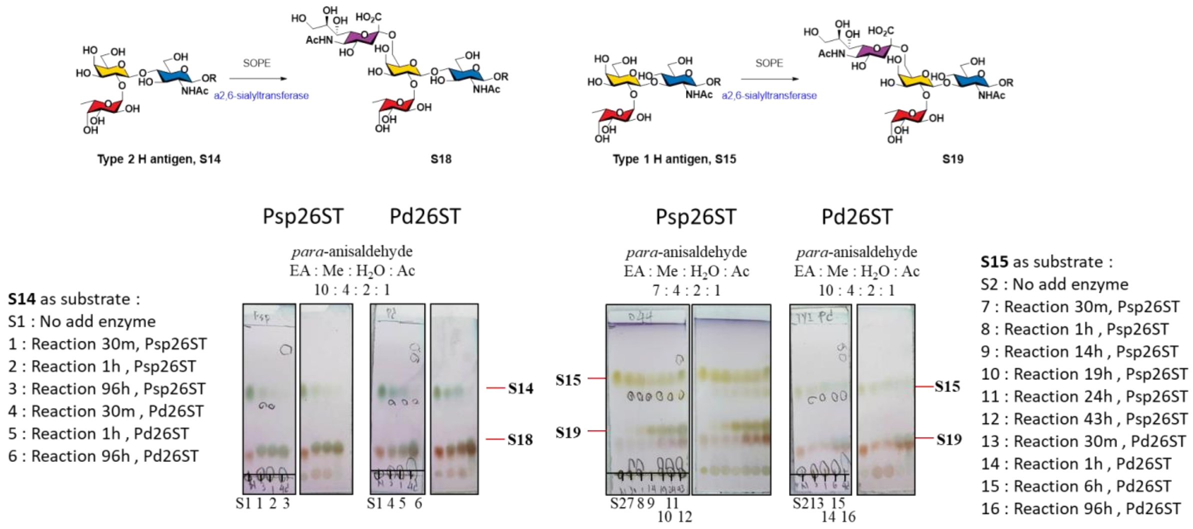

Figure S3. The acceptor preference of α2,6-sialyltransferases (Psp2,6ST and Pd2,6ST). Type 2 H antigen (**S14**) and type 1 H antigen (**S15**) were respectively used for α2,6-sialyltransferase catalyzed sialylation and the reaction progresses were monitored by TLC. The TLC results showed that Psp2,6ST and Pd2,6ST preferred type 2 H antigen as the acceptor. However, when the reaction was extended to 96 h, the type 1 H antigen could be also fully converted to product.

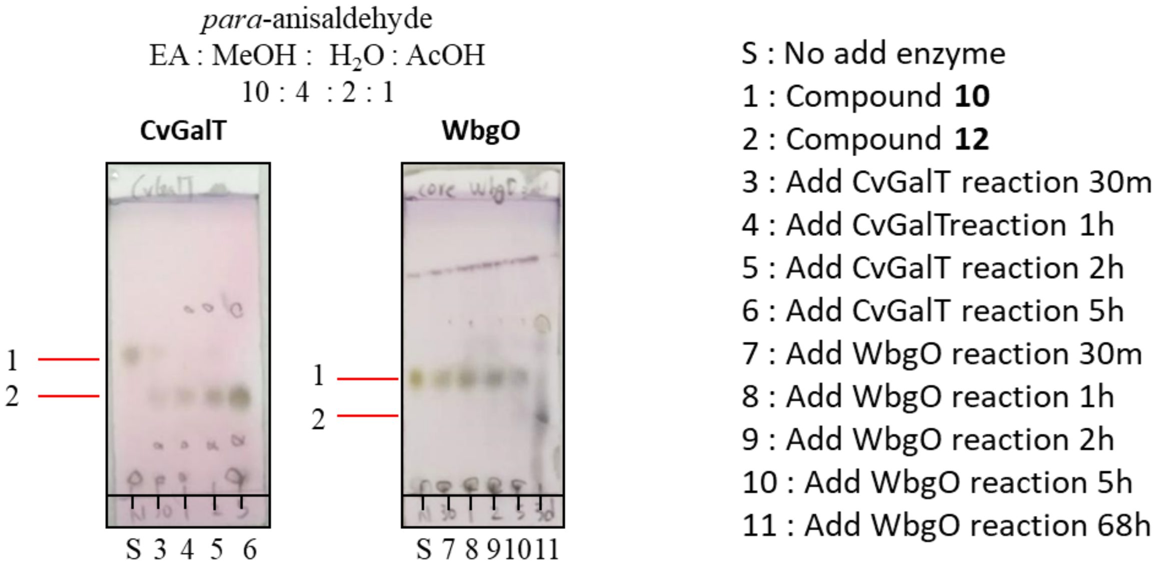

Figure S4. The catalytic activity of β1,3-Galactosyltransferase (CvGalT and WbgO) on compound **10**. Compound **10** was used as the acceptor for β1,3-galactosyltransferase (CvGalT and WbgO) catalyzed galactosylation in DUSD. The TLC results showed that WbgO could not recognize **10** as the acceptor to produce product **12**.

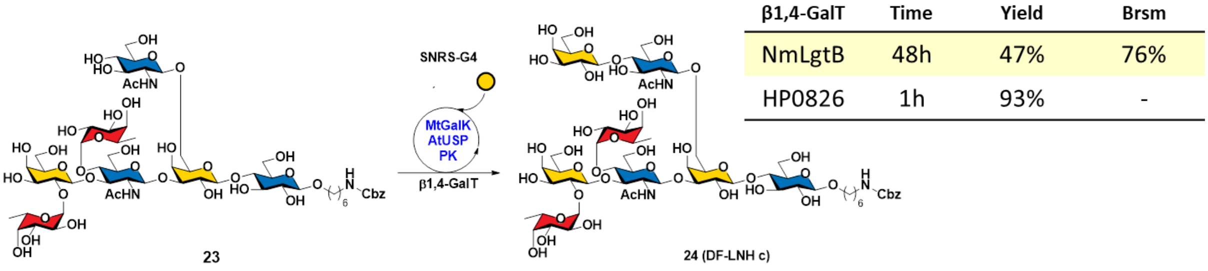

β1,4-GalT	Time	Yield	Brsm
NmLgtB	48h	47%	76%
HP0826	1h	93%	-

Figure S5. NmLgtB and HP0826 were respectively used under SNRS to catalyze β1,4-galactosylation of **23**. The catalytic activity of HP0826 wasn't affected by Le^b epitope. By contrast the catalytic activity of NmLgtB was hindered by Le^b epitope.

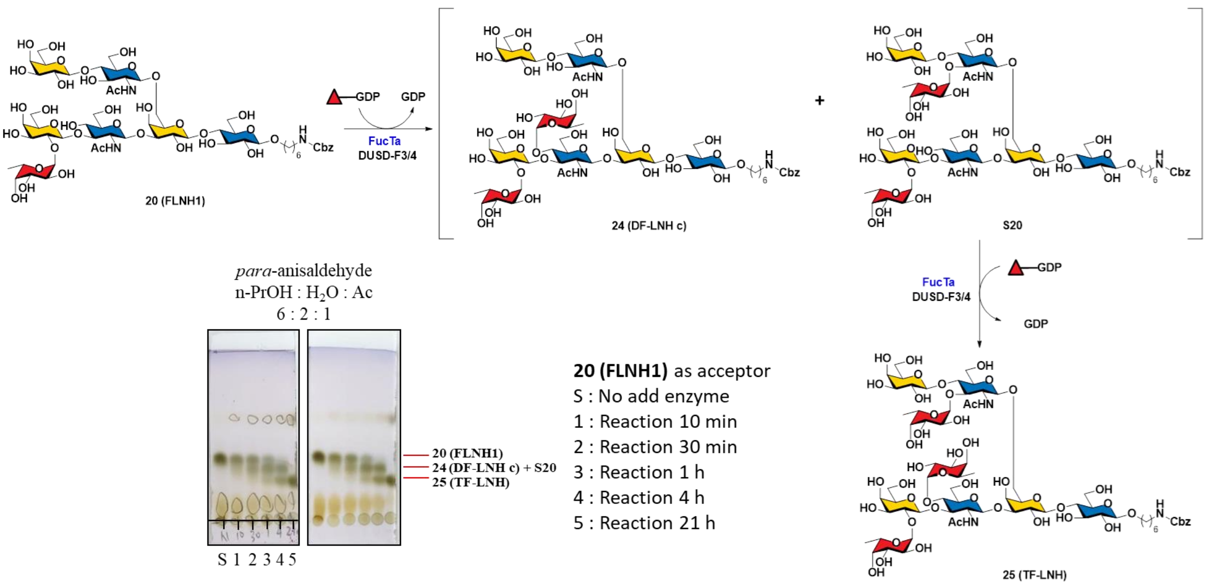

Figure S6. Transformation of **20** (FLNH1) to **25** (TF-LNH) by using 2.2 equivalent of GDP-Fuc and FucTa. The TLC analysis showed that **20** (FLNH1) was gradually fucosylated by FucTa and was transformed to **25** (TF-LNH) after 21 h incubation. It should be noted that the intermediate ratio of **24** (DF-LNH c) to **S20** was not analyzed. Moreover, TLC analysis showed that during the fucosylation of **20** (FLNH1) with DUSD-F3/4 and 2.2 equivalents of GDP-Fuc, di-fucosylated isomers (isomer **S20** as the major product and a minor **24** (DF-LNH c) and desired tri-fucosylated product **25** (TF-LNH) formed in a short reaction time (4 h). Although **25** (TF-LNH) was produced within 30 min, 21 h was needed for **25** to become the major product. To simplify the enzymatic fucosylation process, we used FucTa with SNRS (SNRS-F3/4); however, the efficiency was lower due to differences in the optimized enzyme reaction temperatures (37 °C for FKP and 30 °C for FucTa). Consequently, all subsequent fucosylations were carried out using DUSD-F3/4.

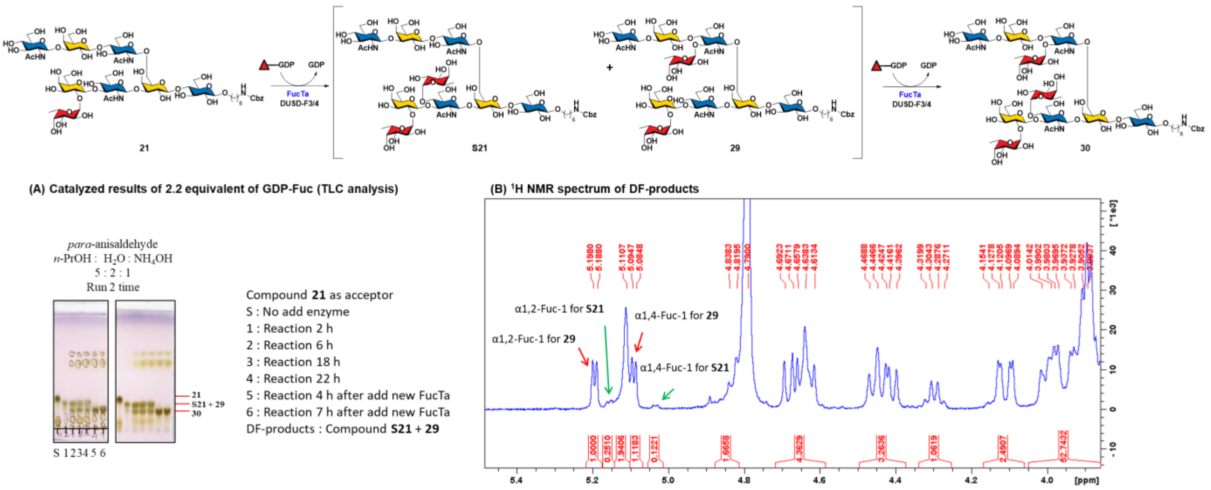

Figure S7. Compound **21** as the acceptor for FucTa catalyzed fucosylation. (A) TLC results indicated that formation rate of intermediates was different. According to these results and results of Figure S6, the controlled equivalent of GDP-Fuc was used to investigate the selectivity of FucTa. (B) The ^{1}H NMR (500 MHz, D$_2$O) spectrum of separated DF-products formed by FucTa catalyzed fucosylation with 1.05 eq. of GDP-Fuc for 6 h. The structures of **29** and **S21** were determined based on the ^{1}H NMR analyses shown in Figure S8.

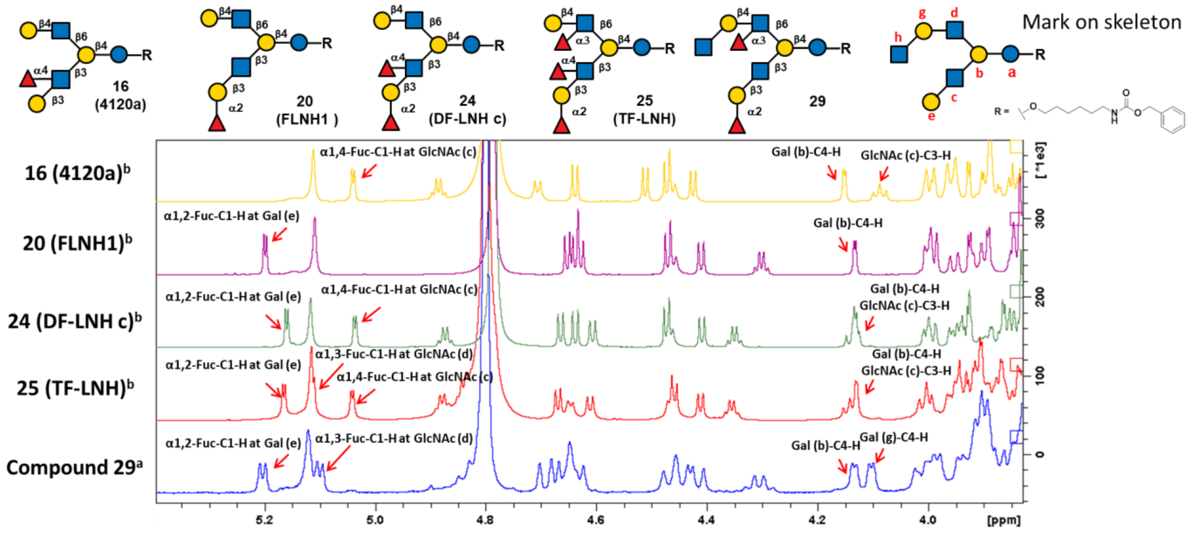

Figure S8. Structure determination of compound **29** by ^{1}H NMR spectra. The structure of compound **29** was determined by comparing ^{1}H NMR (^{a}500 MHz (D$_2$O) and ^{b}850 MHz (D$_2$O)) spectra of **16** (4120a), **20** (FLNH1), **24** (DF-LNH c), **25** (TF-LNH), and compound **29**. Alphabet represents the sugar on the glycan. The Arabic number indicates the position of the saccharide proton.

The chemical shifts of anomeric protons of mono-fucosylated **16** (4120a) and **20** (FLNH1) showed that C1-H of α1,4-Fuc appears at 5.04 ppm while that of α1,2-Fuc appears at 5.20 ppm. However, in the di-fucosylated compound with Le^{b} structure such as **24**

(DF-LNH c), the chemical shift of C1-H of α1,2-Fuc appears up-field shift to 5.16 ppm (The presence of α1,4-Fuc induces the up-filed shift of C1-H of α1,2-Fuc) in comparison with that found in mono-fucosylated **20** (FLNH1) while the chemical shift of C1-H of α1,4-Fuc remains at 5.04 ppm. However, in the ^{1}H NMR spectrum of tri-fucosylated **25** (TF-LNH), the peaks of C1-H of α1,3-Fuc appeared at 5.11 ppm without interfering with the C1-H protons of Fuc on Le^{b} motif. In summary of these available information, the ^{1}H NMR spectrum of compound **29** showed C1-H of second fucose appeared closely to 5.10 ppm while that of the first Fuc remains at 5.20 ppm, determining that the Fuc located at the GlcNAc of β6 arm.

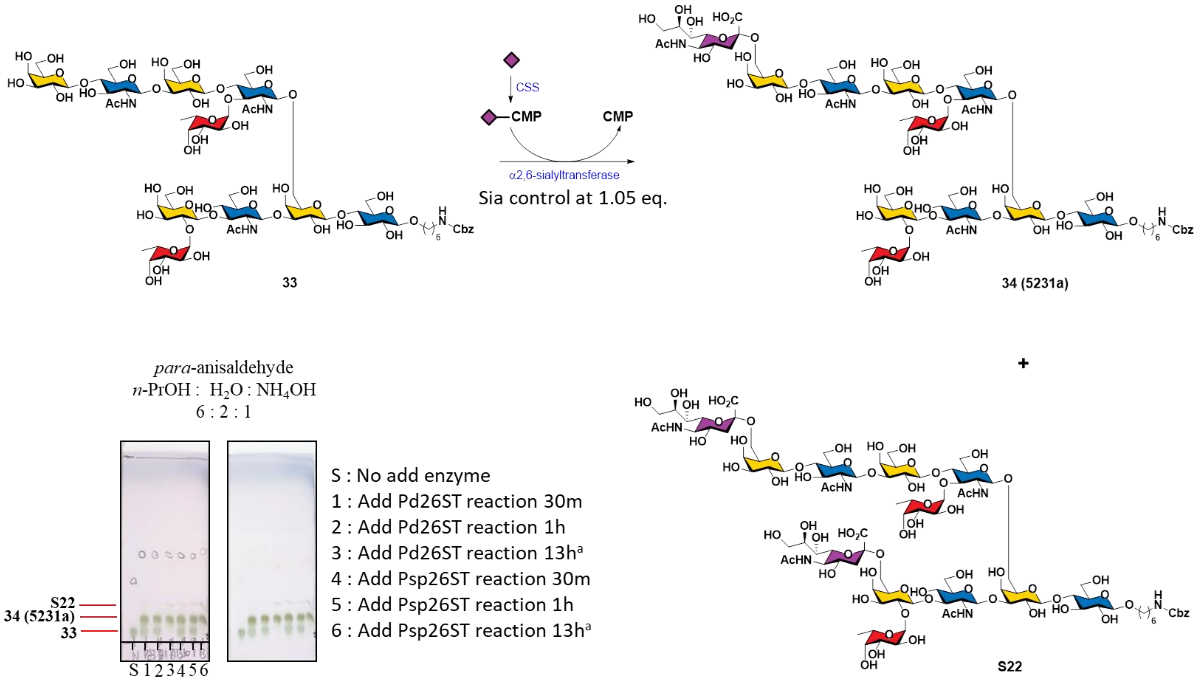

Figure S9. α2,6-Sialylation of compound **33** by Pd2,6ST and Psp2,6ST, respectively. The results showed that the catalytic activity of Pd2,6ST was higher than that of Psp2,6ST for acceptor **33**. After reacting for 1 h, Pd2,6ST catalyzed reaction provided more darker spot of **34** (5231a) on TLC. We also noticed that a small amount of compound **S22** would be produced after reaction for 13 h. [a]After reacting for 1 h, another 1 eq. of CMP-Sia was added into reaction mixture.

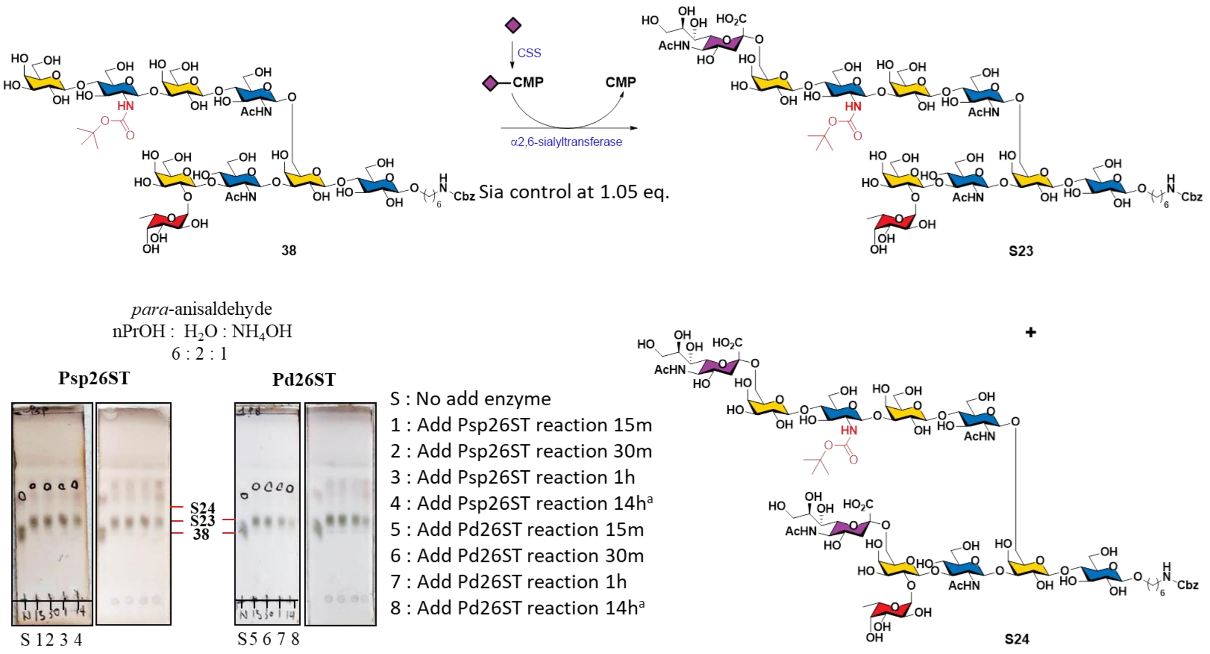

Figure S10. α2,6-Sialylation of compound **38** by Pd2,6ST and Psp2,6ST, respectively. The TLC analysis of reaction progress revealed that catalytic activity of Psp2,6ST might be better than that of Pd2,6ST. After reacting for 14 h, the use of Psp2,6ST as the catalyst produced a small amount of compound **S24** more than that of Pd2,6ST. [a]After reacting for 1h, another 1 eq. of CMP-Sia was added into reaction mixture.

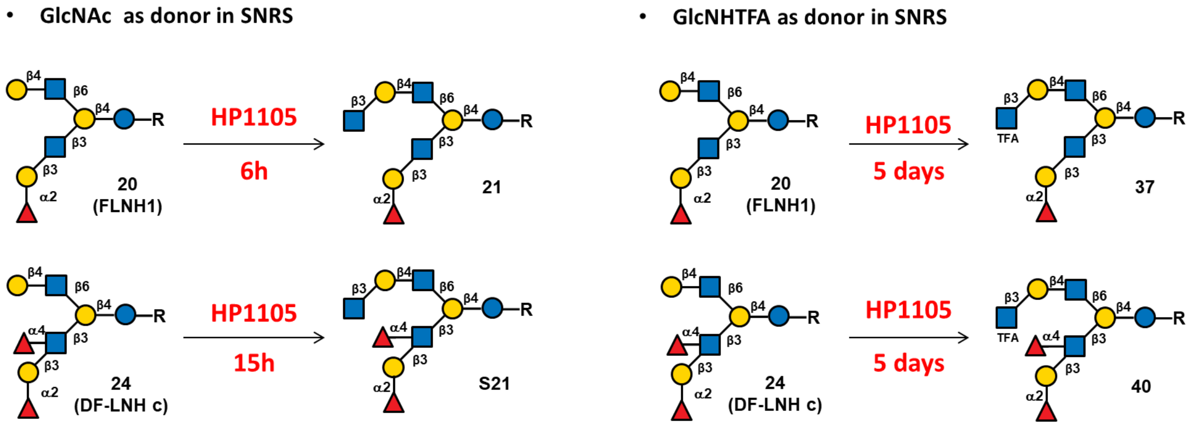

Figure S11. Effect of GlcNAc and GlcNHTFA as the donor in SNRS with HP1105. The results indicated that GlcNHTFA was a weaker donor for HP1105.

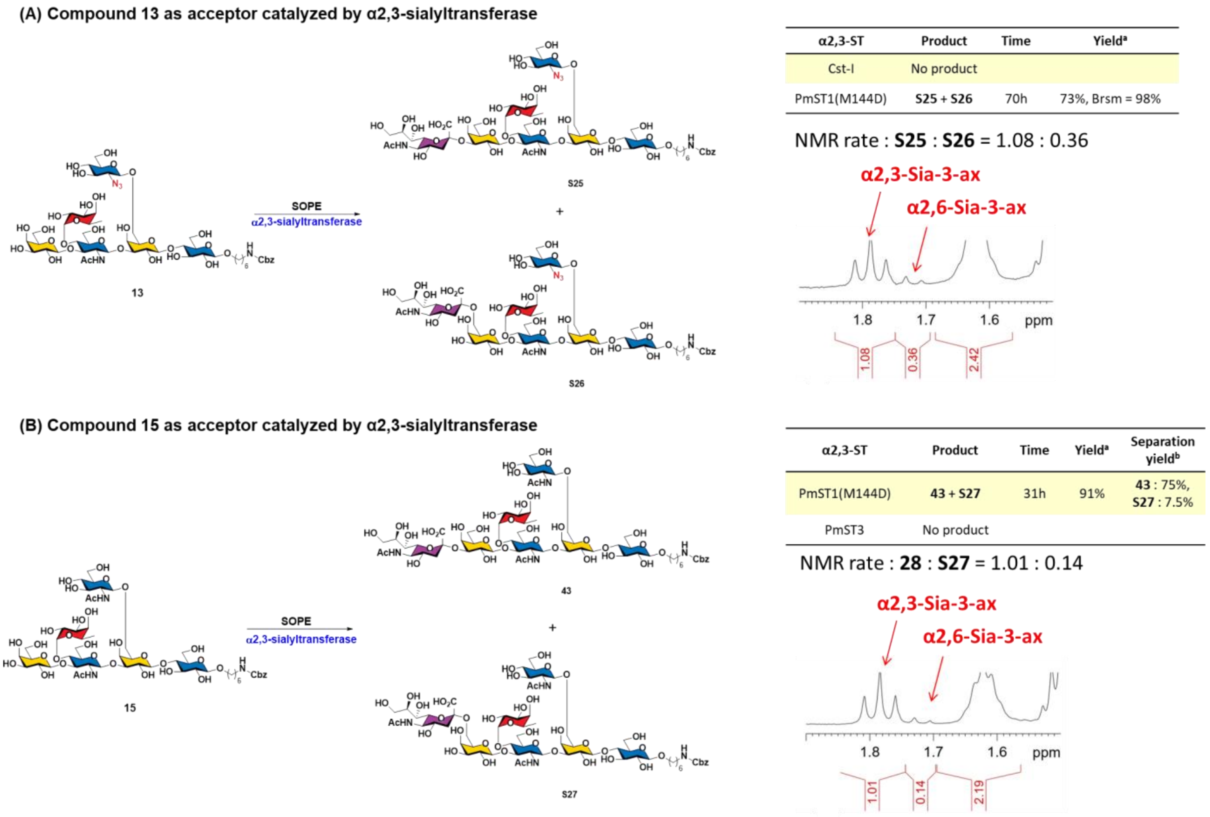

Figure S12. Effect of azide at β6 arm on construction of SLe[a] motif at β3 arm by α2,3sialyltransferases. Only PmST1(M144D) could produce desired product and the presence of remote azide led the decrease of ST catalytic activity. [a]Partially purified by C18, the mixture contains isomers (**S25** + **S26** and **43** + **S27**, respectively). [b]After purification by C18, the mixture was further separated by HPLC-HILIC to obtain yield.

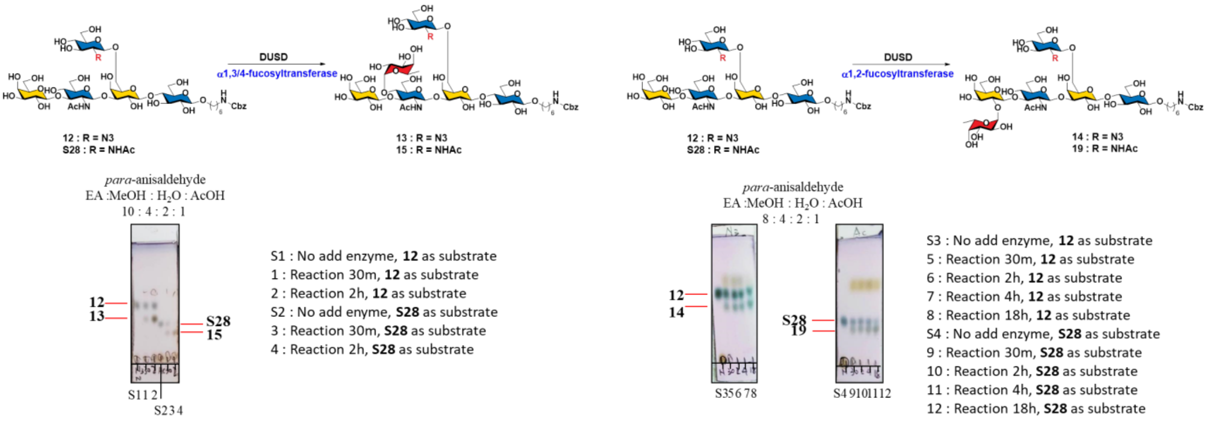

Figure S13. Effect of azide at β6 arm on fucosylation at β3 arm by fucosyltransferases. Compounds **12** (containing $GlcN_3$ group) and **S28** (containing GlcNAc) were used as acceptors for fucosylation by fucosyltransferases (FucTa and FutC). Based on the TLC analysis, the presence of $GlcN_3$ at β6 arm decreased the catalytic activity of the fucosylatransferases. In addition, the decrease in FucTa activity was more significant.

Tables S1. Enzymatic reaction systems and abbreviations for assembling sugars and chemical conversion of *N*-modified glucosamine.

Catalytic function(s)	GT	Method	Abbreviation
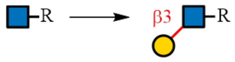	CvGalT	SNRS	SNRS-G3
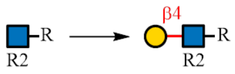 R2 = NHAc or NHTFA	NmLgtB	DUSD	DUSD-G4a
		SNRS	SNRS-G4a
	HP0826	SNRS	SNRS-G4b
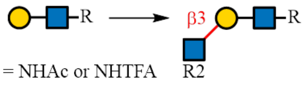 R2 = NHAc or NHTFA	HP1105	DUSD	DUSD-NAc
		SNRS	SNRS-NAc / SNRS-NTFA
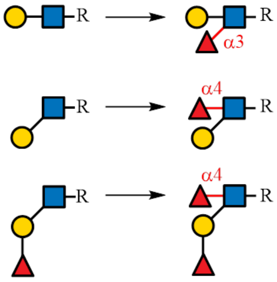	FucTa	DUSD	DUSD-F3/4
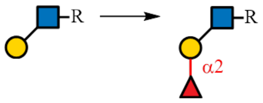	FutC	DUSD	DUSD-F2
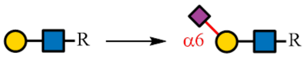	Psp26ST	SOPE	SOPE-S6a
	Pd26ST		SOPE-S6b
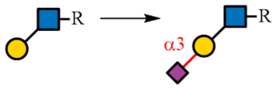	PmST1(M144D)	SOPE	SOPE-S3

Chemical conversion	Reagents	Abbreviation
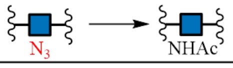	(i) PMe_3, NaOH, H_2O; (ii) Ac_2O, $NaHCO_3$, H_2O	CC1
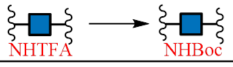	(i) 25% NH_4OH, H_2O; (ii) Boc_2O, $NaHCO_3$, H_2O	CC2
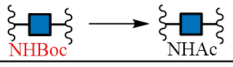	(i) Formic acid; (ii) Ac_2O, $NaHCO_3$, H_2O	CC3

SNRS : sugar nucleotide regeneration system; DUSD : directly using sugar donor; SOPE : sequential one-pot enzymatic glycosylation.

General Procedures for Enzymatic Reactions

General Method for Preparation of GDP-Fuc

To a buffered (200 mM HEPES, pH 7.5) solution containing 40 mM $MgCl_2$, 60 mM Fuc, 60 mM ATP, 60 mM GTP was added FKP (1 mg/mL) and IP (0.2 mg/mL). The reaction solution was incubated at 30 ^oC with agitation at 500 rpm for 80 h. The formation of GDP-Fuc was monitored by TLC (*n*-PrOH/H_2O/25% $NH_4OH_{(aq)}$ = 6/2/1 (v/v/v), R_f = 0.28). The reaction solution was quenched by addition of the same reaction volume amount of EtOH. The mixture was centrifuged at 4 ^oC, 9000 rpm for 10 min to remove insoluble precipitates. The supernatant was collected, concentrated and purified by size-exclusion chromatography (P-2 Bio Gel packed in 2.5 cm × 100 cm column, Biored) using H_2O as eluent. The fractions containing GDP-Fuc were pooled and concentrated. The crude mixture containing GDP-Fuc was resuspended in 6 mL ddH_2O (one batch for less than 300 mg crude mixture), filtered (0.45 μm PVDF filter, Millipore), purified by a 120 mL Capto™ DEAE anion exchange column (Cytiva) with gradient of 0 mM-200 mM NaCl in ddH_2O, flow rate = 10 mL/min. The fractions containing GDP-Fuc were pooled, concentrated, and purified by size-exclusion chromatography (P-2 Bio Gel packed in 2.5 cm × 100 cm column, Biored) using H_2O as eluent to give pure GDP-Fuc.

General Procedure for Enzymatic β1,4-Galactosylation by Sugar Nucleotide Regeneration System (SNRS-G4a: SNRS with NmLgtB)

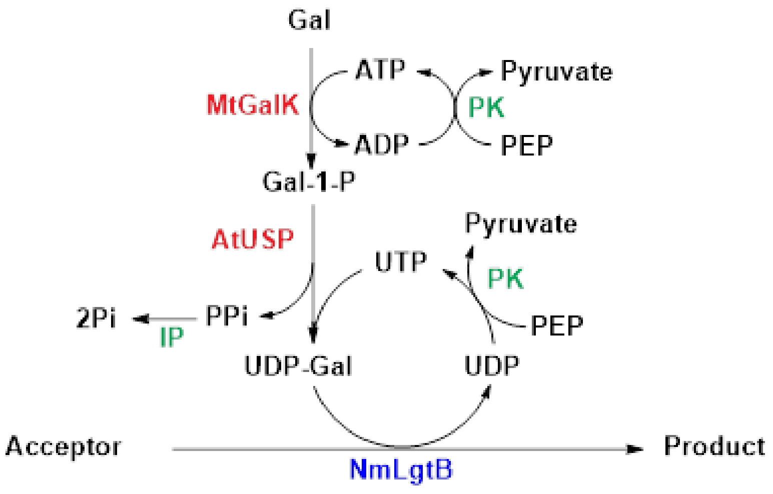

The pH of buffer solution (100 mM HEPES or 100 mM Tris-HCl) containing 12-20 mM Gal, 2 mM of ATP, 5 mM of UTP, 36-60 mM of phosphoenolpyruvate (PEP), and 20 mM $MgCl_2$ was adjusted to 7-7.5 by adding 2N $NaOH_{(aq)}$. To the above solution were added 10 mM acceptor, 0.08 mg/mL of MtGalK, 0.025 mg/mL of AtUSP, 0.012 mg/mL of inorganic (IP), 10 U/mL of pyruvate kinase (PK), and 0.15-0.3 mg/mL of NmLgtB. The solution was incubated at 37 ^oC. More enzymes were added if necessary. The reaction progression was monitored by TLC analysis and stained with *para*-anisaldehyde stain.

General Procedure for Enzymatic β1,4-Galactosylation by Sugar Nucleotide Regeneration System (SNRS-G4b: SNRS with HP0826)

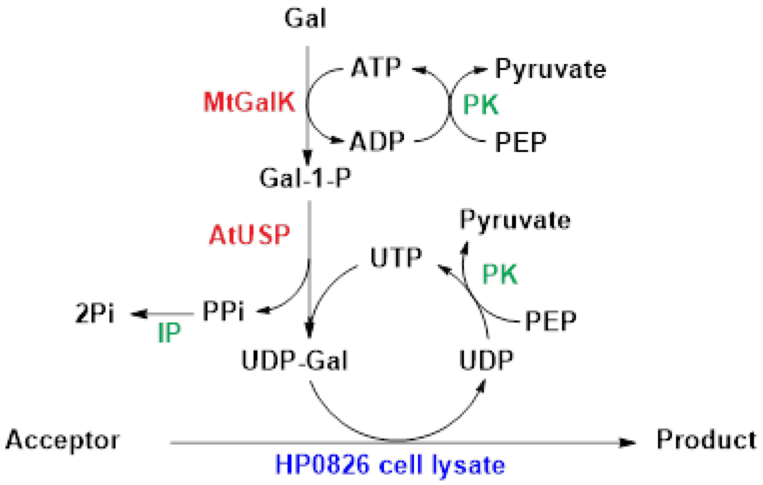

The pH of buffer solution (50 mM HEPES) containing 12 mM Gal, 2 mM of ATP, 5 mM of UTP, 36 mM of phosphoenolpyruvate (PEP), and 20 mM $MgCl_2$ was adjusted to 7-7.5 by adding 2N $NaOH_{(aq)}$. To the above solution were added 10 mM acceptor, 0.08 mg/mL of MtGalK, 0.025 mg/mL of AtUSP, 0.012 mg/mL of IP, 10 U/mL of PK, and HP0826 cell lysate (20% (v/v)). The solution was incubated at 37 ^{o}C. More enzymes were added if necessary. The reaction progression was monitored by TLC analysis and stained with *para*-anisaldehyde stain.

General Procedure for Enzymatic β1,3-Galactosylation by Sugar Nucleotide Regeneration System (SNRS-G3: SNRS with CvβGalT)

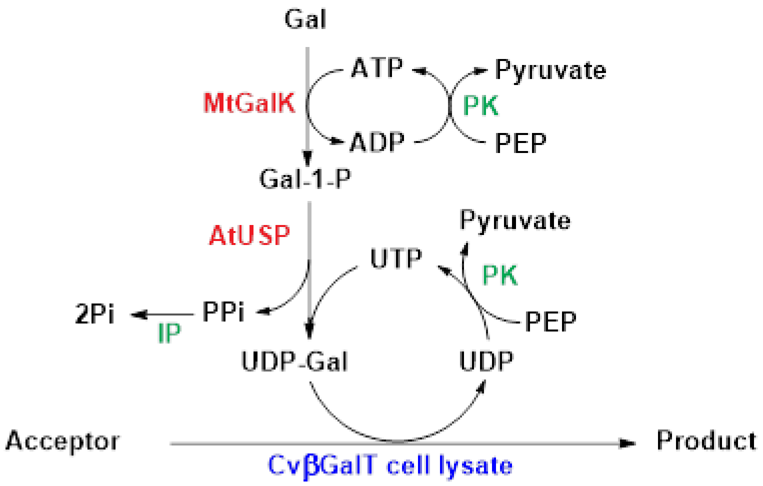

The pH of buffer solution (100 mM Tris-HCl) containing 12 mM Gal, 2 mM of ATP, 5 mM of UTP, 36 mM of phosphoenolpyruvate (PEP), and 20 mM $MgCl_2$ was adjusted to 7-7.5 by adding 2N $NaOH_{(aq)}$. To the above solution were added 10 mM acceptor, 0.08 mg/mL of MtGalK, 0.025 mg/mL of AtUSP, 0.012 mg/mL of IP, 10 U/mL of PK, and CvβGalT cell

lysate (20% (v/v)). The solution was incubated at 37 °C. More enzymes were added if necessary. The reaction progression was monitored by TLC analysis and stained with *para*-anisaldehyde stain.

General Procedure for Enzymatic β1,3-*N*-Acetylglucosaminylation by Sugar Nucleotide Regeneration System (SNRS-NAc: SNRS with HP1105)

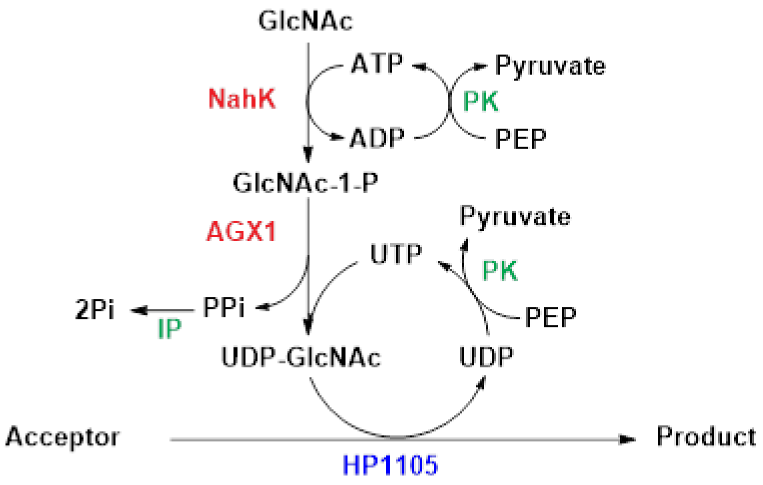

The pH of buffer solution (100 mM Tris-HCl) containing 12 mM GlcNAc, 2 mM of ATP, 5 mM of UTP, 36 mM of phosphoenolpyruvate (PEP), and 20 mM $MgCl_2$ was adjusted to 7-7.5 by adding 2N $NaOH_{(aq)}$. To the above solution were added 10 mM acceptor, 0.07 mg/mL of NahK, 0.01 mg/mL of AGX1, 0.012 mg/mL of IP, 10 U/mL of PK, and 0.5 mg/mL of HP1105. The solution was incubated at 25 °C. More enzymes were added if necessary. The reaction progression was monitored by TLC analysis and stained with *para*-anisaldehyde stain.

General Procedure for Enzymatic β1,3-*N*-Trifluoroacetylglucosaminylation by Sugar Nucleotide Regeneration System (SNRS-NTFA: SNRS with HP1105 and GlcNTFA as donor precursor)

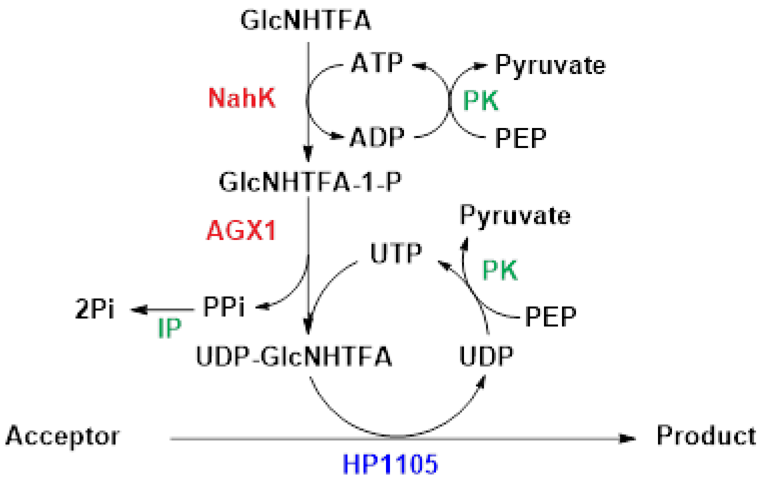

The pH of buffer solution (100 mM Tris-HCl) containing 12 mM GlcNTFA, 2 mM of ATP, 5 mM of UTP, 36 mM of phosphoenolpyruvate (PEP), and 20 mM $MgCl_2$ was adjusted to 7-7.5 by adding 2N $NaOH_{(aq)}$. To the above solution were added 10 mM acceptor, 0.07 mg/mL of NahK, 0.01 mg/mL of AGX1, 0.012 mg/mL of IP, 10 U/mL of PK, and 0.5 mg/mL of HP1105. The solution was incubated at 25 ^{o}C. More enzymes were added if necessary. The reaction progression was monitored by TLC analysis and stained with *para*-anisaldehyde stain.

General Procedure for Enzymatic β1,3-galactosylation by Directly Using Sugar Donor (DUSD-G4a: DUSD with NmLgtB)

The pH buffer solution (100 mM HEPES) containing 20 mM $MgCl_2$ was adjust to 7-7.5 by adding 4N $HCl_{(aq)}$. To the above solution were added 10 mM of acceptor, 15 mM of UDP-Gal, and 0.3 mg/mL of NmLgtB. The solution was incubated at 37 ^{o}C. More enzymes were added if necessary. The reaction progression was monitored by TLC analysis and stained with *para*-anisaldehyde stain.

General Procedure for Enzymatic α1,3/1,4-Fucosylation by Directly Using Sugar Donor (DUSD-F3/4: DUSD with FucTa)

The pH buffer solution (100 mM Tris-HCl) containing 20 mM $MgCl_2$ was adjust to 7-7.5 by adding 4N $HCl_{(aq)}$. To the above solution were added 10 mM of acceptor, GDP-Fuc (11-15 mM for installation of one Fuc, 15-42 mM for installation of two Fuc.), and 0.084 mg/mL of FucTa. The solution was incubated at 30 ^{o}C. More enzymes were added if necessary. The reaction progression was monitored by TLC analysis and stained with *para*-anisaldehyde stain.

General Procedure for Enzymatic α1,2-Fucosylation by Directly Using Sugar Donor (DUSD-F2: DUSD with FutC)

The pH buffer solution (50 mM HEPES) containing 20 mM $MgCl_2$ was adjust to 7-7.5 by adding 4N $HCl_{(aq)}$. To the above solution were added 10 mM of acceptor, 15 mM of GDP-Fuc, and 0.5 mg/mL of FutC. The solution was incubated at 30 ^{o}C. More enzymes were added if necessary. The reaction progression was monitored by TLC analysis and stained with *para*-anisaldehyde stain.

General Procedure for Sequential One Pot Enzymatic α2,3-sialylation (SOPE-S3: SOPE with PmST1)

The pH of a buffer solution (100 mM Tris-HCl) containing 20 mM of sialic acid, 24 mM of CTP, and 20 mM of $MgCl_2$, was adjusted to 8.5 by adding 2N $NaOH_{(aq)}$. Then, 0.083 mg/mL of CSS and 0.012 mg/mL IP were added to the above solution. The resulting mixture was

incubated at 37 ^{o}C with agitation at 600 rpm for 1 h and the formation of CMP-sialic acid was monitored by TLC analysis (*n*-PrOH/H_2O/AcOH = 6/2/1 (v/v/v), R_f = 0.06). After the completion of the reaction as indicated by the disappearance of all of sialic acid on TLC, 10 mM of acceptor was added and the pH of the reaction solution was adjusted to pH 8.5. Finally, 0.1 mg/mL PmST1(M144D) was added and the solution was incubated at 37 ^{o}C. Furthermore, 1.9U/mL of alkaline phosphatase was added to decompose CMP. More enzymes were added if necessary by TLC analysis and stained with *para*-anisaldehyde stain.

General Procedure for Sequential One Pot Enzymatic α2,6-sialylation (SOPE-S6a: SOPE with Psp2,6ST)

The pH of a buffer solution (100 mM Tris-HCl) containing 15 mM of sialic acid, 18 mM of CTP, and 20 mM of $MgCl_2$ was adjusted to 8.5 by adding 2N $NaOH_{(aq)}$. Then, 0.083 mg/mL of CSS and 0.012 mg/mL IP were added to the above solution. The resulting mixture was incubated at 37 ^{o}C with agitation at 600 rpm for 1 h and the formation of CMP-sialic acid was monitored by TLC analysis (*n*-PrOH/H_2O/AcOH = 6/2/1 (v/v/v), R_f = 0.06). After the completion of the reaction as indicated by the disappearance of all of sialic acid on TLC, 10 mM of acceptor was added and the pH of the reaction solution was adjusted to pH 8.5. Finally, 0.1 mg/mL Psp26ST was added and the solution was incubated at 37 ^{o}C. Furthermore, 1.9 U/mL of alkaline phosphatase was added to decompose CMP. More enzymes were added if necessary by TLC analysis and stained with *para*-anisaldehyde stain.

General Procedure for Sequential One Pot Enzymatic α2,6-sialylation (SOPE-S6b: SOPE with Pd2,6ST)

The pH of a buffer solution (100 mM Tris-HCl) containing 15 mM of sialic acid, 18 mM of CTP, and 20 mM of $MgCl_2$ was adjusted to 8.5 by adding 2N $NaOH_{(aq)}$. Then, 0.083 mg/mL of CSS and 0.012 mg/mL IP were added to the above solution. The resulting mixture was incubated at 37 ^{o}C with agitation at 600 rpm for 1 h and the formation of CMP-sialic acid was monitored by TLC analysis (*n*-PrOH/H_2O/AcOH = 6/2/1 (v/v/v), R_f = 0.06). After the completion of the reaction as indicated by the disappearance of all of sialic acid on TLC, 10 mM of acceptor was added, and the pH of the reaction solution was adjusted to pH 8.5. Finally, 0.1 mg/mL Pd26ST was added and the solution was incubated at 37 ^{o}C. Furthermore, 1.9U/mL of alkaline phosphatase was added to decompose CMP. More enzymes were added if necessary by TLC analysis and stained with *para*-anisaldehyde stain.

General Procedure for Chemical Conversion

General Procedure for Chemical Conversion 1 (CC1, from GlcN3 to GlcNAc)

To a water solution containing 10 mM of GlcN3, 80 mM of trimethylphosphine (PMe_3) and 100 mM of sodium bicarbonate ($NaHCO_3$) were added. The resulting mixture was stirred at rt

for 30 min, and reaction was monitored by TLC analysis. After the completion of the reaction as indicated by the disappearance of starting material on TLC. The reaction was concentrated to give amine containing compound. Ten mM of above amine (1.0 equiv.) was dissolved in H_2O followed by adding solid $NaHCO_3$ (10.0 equiv.) and acetic anhydride (Ac_2O, 10.0 equiv.) After being sonicated for 1 min, the reaction mixture was incubated at rt for 30 min. After the completion of the reaction as indicated by the disappearance of starting material on TLC, the resulting solution was concentrated and resulting residue was purified by a C18 reverse-phase silica column (Sep-Pak Vac C18 cartridge 5 g, 55-105 μm, Waters) using a solution followed by stepwise elution of 0-100% MeOH in ddH_2O as eluent (increase 10% MeOH per step, 30 mL eluent for each step). The fractions containing products were further purified by size-exclusion column using H_2O as eluent (Toyopearl HW-40S packed in a column 2.5 cm × 50 cm for hexa- saccharides).

General Procedure for Chemical Conversion 2 (CC2, from GlcNTFA to GlcNBoc)

To a water solution containing 10 mM of GlcNTFA was added the same volume of ammonium solution. The resulting mixture was incubated at rt with agitation at 600 rpm for 30 min, and the reaction was monitored by TLC analysis. After the completion of the reaction as indicated by the disappearance of starting material on TLC. The reaction was concentrated then lyophilizes to give amine containing compound. Ten mM of above amine (1.0 equiv.) was dissolved in MeOH followed by adding di-*tert*-butyl dicarbonate (Boc_2O, 3.0 equiv.) and solid $NaHCO_3$ (6.0 equiv.). If the amine containing compound could not be dissolved in the solution, an appropriate amount of ddH_2O was added till the amine compound dissolved. The reaction mixture was stirred at rt for 27 h. After the completion of the reaction as indicated by the disappearance of starting material on TLC, the reaction was neutralized by addition of acetic acid then concentrated. The resulting residue was purified by a C18 reverse-phase silica column (Sep-Pak Vac C18 cartridge 5 g, 55-105 μm, Waters) using a solution followed by stepwise elution of 0-100% MeOH in ddH_2O containing 0.1% acetic acid as eluent (increase 10% MeOH per step, 30 mL eluent for each step).

General Procedure for Chemical Conversion 3 (CC3, from GlcNBoc to GlcNAc)

To a water solution containing 5 mM of GlcNBoc was added the same volume of formic acid at 0 ^{o}C. The resulting mixture was stirred at 0 ^{o}C to rt for 23 h and the reaction was monitored by TLC analysis. After the completion of the reaction as indicated by the disappearance of starting material on TLC. The reaction was concentrated and then lyophilized to give amine containing compound. Five mM of above amine (1.0 equiv.) was dissolved in H_2O followed by adding solid $NaHCO_3$ (10.0 equiv.) and acetic anhydride (Ac_2O, 10.0 equiv.) After being sonicated for 1 min, the reaction mixture was incubated at rt for 30 min. After the completion of the reaction as indicated by the disappearance of starting material on TLC, the resulting

solution was concentrated, and resulting residue was purified by a C18 reverse-phase silica column (Sep-Pak Vac C18 cartridge 5 g, 55-105 μm, Waters) using a solution followed by stepwise elution of 0-100% MeOH in ddH_2O as eluent (increase 10% MeOH per step, 30 mL eluent for each step). The fractions containing products were further purified by size-exclusion column using H_2O as eluent (Toyopearl HW-40S packed in a column 2.5 cm × 50 cm for deca- to undeca- saccharides).

General Purification Procedure

General Purification Procedure 1

If there are regioisomer formed in enzymatic glycosylation, the reaction mixture is suitable for this purification procedure. The enzymatic reaction was quenched by addition of the same reaction volume of EtOH. The reaction solution was centrifuged (10,000 × g, 10 min) to remove enzymes and insoluble precipitates. The supernatant was collected, filtered (0.45 μm, PVDF filter; Millipore), and then concentrated. The resulting residue was purified by a C18 reverse-phase silica column (Sep-Pak Vac C18 cartridge 5g, 55-105μm**,** Waters) using a solution followed by stepwise elution of 0-100% MeOH in H_2O as eluent (increase 10% MeOH per step, 30 mL eluent for each step). The fractions containing products were polled, concentrated, and then purified by size-exclusion column using H_2O as eluent (Toyopearl HW-40S packed in a column 2.5 cm × 50 cm).

General Purification Procedure 2

If there are regioisomers formed in enzymatic glycosylation (sialylation or fucosylation), the reaction mixture is suitable for this purification procedure. Follow the same procedure as described in **general purification procedure 1**, but the residues after C18 reverse-phase silica column were pooled and purified by Xbridge® BEH Amide OBD™ Prep Column (10 × 250 mm, 130Å, 5 μm) with a flow rate of 3 mL/min. HPLC purification was monitored by ELSD (evaporative light scattering detector), and fractions were analyzed by TLC. After pooled and concentrated the solution containing product, further desalting procedure was applied on the product by a C18 reverse-phase silica column (Sep-Pak Vac C18 cartridge 5g, 55-105μm**,** Waters) using a solution followed by stepwise elution of 0-100% MeOH in H_2O as eluent (increase 10% MeOH per step, 30 mL eluent for each step) or size-elusion column using H_2O (or 10 mM of ammonium bicarbonatedas in H_2O) as eluent (Toyopearl HW-40S packed in a column 2.5 cm × 50 cm).

HPLC mobile phase condition for sialylated compounds **34** (5231a) and **42** (5231b):

Using an elution of 62% acetonitrile (CAN) in water containing 100 mM ammonium formate (pH = 3.45) for 50 mins.

HPLC mobile phase condition for sialylated compounds **43** and **S27**:

Using an elution of 69% acetonitrile (CAN) in water containing 100 mM ammonium formate

(pH = 3.45) for 50 mins.

HPLC mobile phase condition for fucosylated compounds **48** (DFS-*i*LNO I) and **49** (DFS-*i*LNO II):

Using an elution of 64% acetonitrile (CAN) in water containing 100 mM ammonium formate (pH = 3.45) for 50 mins.

HPLC mobile phase condition for hydrolysed compounds **51** (DFS-LNO I) and **53**:

Using an elution of 64% acetonitrile (CAN) in water containing 100 mM ammonium formate (pH = 3.45) for 50 mins.

General Purification Procedure 3

The reaction mixture containing sialylated glycans is suitable for this purification procedure. By following the same procedure as described in **general purification procedure 1**, the residue after C-18 reverse-phase silica column was purified by anion exchange chromatography (DEAE Sepharose Fast Flow resin, GE Healthcare). The column was washed with water (10 mL) followed by the elution with a gradient of aqueous sodium chloride (NaCl, 10-100 mM, increase 10 mM for each gradient and 10 mL for each gradient). The fractions containing product were pooled, concentrated, and then purified by size-exclusion column using H_2O as eluent (BioGel P2 gel packed in a column 2.5 cm x 100 cm, Biorad).

Synthetic procedures and characterization of new compounds

Compound **1**,[18] **3**,[19] and **4**[20] were reported previously.

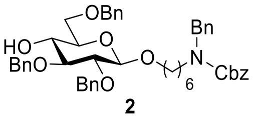

2

Compound 2. To a solution of compound **S5**[21] (1.16 g, 2.303 mmol) in anhydrous ACN (12 mL) was added benzaldehyde dimethyl acetal (0.4 mL, 2.764 mmol, 1.2 equiv.) and CSA (107 mg, 0.46 mmol, 0.2 equiv.) under nitrogen atmosphere. The reaction mixture was stirred at rt for 1 h and then quenched with Et_3N. The mixture was concentrated under reduced pressure. The residue was extracted with EtOAc and the resulting organic solution was washed with H_2O and brine. The organic layer was dried over $MgSO_4$, filtered, and concentrated. The residue was purified by silica gel chromatography (EtOAc/Hexane = 3/2, R_f = 0.38) to afford 4,6-*O*-benzylidene compound.

To a solution of 4,6-*O*-benzylidene compound in anhydrous DMF (23 mL) was added NaH (221 mg, 9.21 mmol, 4 equiv.) under a nitrogen atmosphere at 0 °C. The reaction mixture was stirred at 0 °C for 30 min and then benzyl bromide (1.1 mL, 9.21 mmol, 4 equiv.) was added to the solution. The reaction mixture was gradually warmed to rt and stirred for 1 h. The reaction was quenched by MeOH and then concentrated in vacuo. The residue was

extracted with EtOAc and the resulting organic solution was washed with H_2O and brine. The organic layer was dried over $MgSO_4$, filtered, and concentrated.

To a solution of above residue in DCM (12 mL) was added Et_3SiH (3.5 mL, 22.11 mmole, 12.0 equiv.). The reaction mixture was stirred at 0 °C for 15 min and then BF_3•OEt_2 (1 mL, 7.37 mmole, 4.0 equiv.) was slowly added to the solution. The reaction mixture was stirred at rt for 1 h. The reaction mixture was quenched with Et_3N and then concentrated under reduced pressure. The residue was extracted with EtOAc and the resulting organic solution was washed with H_2O and brine. The organic layer was dried over $MgSO_4$, filtered, and concentrated. The residue was purified by silica gel chromatography (EtOAc/Hexane = 1/2, R_f = 0.38) to afford compound **2** (1.42 g, 80% over there steps). $[\alpha]_D^{23}$ = 8.13 (c = 2.0, $CHCl_3$); ^{1}H NMR (400 MHz, $CDCl_3$) δ 7.37 – 7.21 (m, 24H, Ph), 7.18-7.12 (m, 1H, Ph), 5.16 (br, 2H, $PhCH_2$), 4.91 (d, *J* = 11.4 Hz, 2H, 2×PhCH), 4.71 (d, *J* = 11.2 Hz, 1H), 4.68 (d, *J* = 9.9 Hz, 1H), 4.59 (d, *J* = 12.1 Hz, 1H, PhCH), 4.55 (d, *J* = 12.1 Hz, 1H, PhCH), 4.43 (d, *J* = 6.1 Hz, 2H, $PhCH_2$), 4.37 (d, *J* = 7.2 Hz, 1H, H-1), 3.88 (br, 1H, CH_{linker}), 3.75 (dd, *J* = 10.4, 3.9 Hz, 1H, H-6a), 3.68 (dd, *J* = 10.4, 5.3 Hz, 1H, H-6b), 3.62-3.52 (m, 1H, H-4), 3.50-3.34 (m, 4H, H-2, H-3, H-5, CH_{linker}), 3.25-3.14 (m, 2H, $CH_{2\,linker}$), 2.56 (br, 1H, OH), 1.59 -1.41 (m, 4H, 2×$CH_{2\,linker}$), 1.39-1.24 (m, 4H, 2×$CH_{2\,linker}$); ^{13}C NMR (101 MHz, $CDCl_3$) δ 156.74 (CO_{Cbz}), 156.16 (CO_{Cbz}), 138.62, 138.44, 137.92, 136.83, 128.50, 128.39, 128.34, 128.03, 127.94, 127.79, 127.67, 127.24, 103.65, 84.03, 81.69, 77.32, 77.20, 77.00, 76.69, 75.23, 74.65, 73.99, 73.64, 71.63, 70.35, 69.98, 67.12, 50.42, 50.13, 47.12, 46.16, 29.65, 28.04, 27.65, 26.58, 25.88. HRMS (ESI) m/z of $C_{48}H_{55}NO_8$ $[M+H]^+$: calcd 773.3928, found 773.3923.

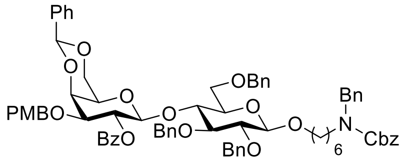

5

Compound 5. A mixture of the acceptor **2** (2.88 g, 3.72 mmol, 1 equiv.), donor **1** (2.12 g, 4.46 mmol, 1.2 equiv.), and 3Å flame-dried molecular sieves was stirred in CH_2Cl_2 (50 mL) under a nitrogen atmosphere at rt for 30 min. The reaction mixture was then cooled to -40 ^{o}C, and then NIS (1 g, 4.46 mmol, 1.2 equiv.) was added to the solution followed by slow addition of TMSOTf (100 μL, 0.56 mmol, 0.15 equiv.). The reaction mixture was stirred for 1 h, allowing the temperature to rise from -40 °C to -20 °C, and then quenched with Et_3N. The reaction mixture was filtered through a pad of Celite® and the resulting solution was concentrated under reduced pressure. The residue was dissolved in EtOAc and the resulting solution was washed with saturated $Na_2S_2O_{3(aq)}$, $NaHCO_3$, and then brine. The organic layer was dried over $MgSO_4$, filtered, and concentrated. The residue was purified by silica gel chromatography. (EtOAc/Hexane = 2/3, R_f = 0.3) to give compound **5** (4.97 g, 85%). $[\alpha]_D^{24}$ =

19.71 (c = 2.0, $CHCl_3$); ^{1}H NMR (850 MHz, $CDCl_3$) δ 7.98 (d, *J* = 8.0 Hz, 2H, Ph), 7.59 (t, *J* = 7.4 Hz, 1H, Ph), 7.56-7.53 (m, 2H, Ph), 7.45 (t, *J* = 7.7 Hz, 2H, Ph), 7.39 (d, *J* = 6.5 Hz, 2H, Ph), 7.34 – 7.22 (m, 22H, Ph), 7.20-7.16 (m, 4H, Ph), 7.15-7.13 (m, 1H, Ph), 7.11 (d, *J* = 8.5 Hz, 2H, Ph), 6.71 (d, *J* = 8.6 Hz, , 2H, Ph), 5.56 (dd, *J* = 9.8, 8.3 Hz, 1H, H-2'), 5.48 (s, 1H, $CH_{Benzylidene}$), 5.18-5.14 (m, 2H, $PhCH_2$), 5.12 (d, *J* = 10.9 Hz, 1H, PhCH), 4.88-4.85 (m, 1H, PhCH), 4.84 (d, *J* = 10.9 Hz, 1H, PhCH), 4.72 (d, *J* = 8.3 Hz, 1H, H-1'), 4.68 (d, *J* = 11.1 Hz, 1H, PhCH), 4.58 (d, *J* = 12.6 Hz, 1H, PhCH), 4.52 (d, *J* = 12.6 Hz, 1H, PhCH), 4.47-4.44 (m, 3H, 3*PhCH), 4.28-4.24 (m, 1H, H-1), 4.22 (d, *J* = 12.2 Hz, 1H, PhCH), 4.17 (d, *J* = 11.9 Hz, 1H, H-6a'), 4.10 (d, *J* = 3.1 Hz, 1H, H-4'), 3.88 (t, *J* = 9.3 Hz, 1H, H-4), 3.85-3.79 (m, 2H, H-6b', CH_{linker}), 3.75 (s, 3H, $CH_{3\ PMB}$), 3.62 (t, *J* = 9.0 Hz, 1H, H-3), 3.59 (dd, *J* = 10.9, 4.3 Hz, 1H, H-6a), 3.55 (d, *J* = 9.4 Hz, 1H, H-6b), 3.49 (dd, *J* = 9.8, 3.1 Hz, 1H, H-3'), 3.43-3.35 (m, 1H, CH_{linker}), 3.37 (t, *J* = 8.5 Hz, 1H, H-2), 3.24-3.23 (m, 2H, H-5, CH_{linker}), 3.17-3.12 (m, 1H, CH_{linker}), 2.99 (s, 1H, H-5'), 1.58-1.42 (m, 4H, 2×$CH_{2\ linker}$), 1.35-1.20 (m, 4H, 2×$CH_{2\ linker}$); ^{13}C NMR (214 MHz, $CDCl_3$) δ 164.84 (*C*O $_{Bz}$), 159.17 (*C*O$CH_{3\ PMB}$), 156.68 (CO $_{Cbz}$), 156.10 (CO $_{Cbz}$), 139.08, 138.56, 138.41, 137.89, 137.74, 136.81, 136.73, 133.01, 129.97, 129.81, 129.77, 129.13, 128.79, 128.44, 128.35, 128.21, 128.17, 128.03, 128.00, 127.85, 127.73, 127.63, 127.49, 127.41, 127.24, 127.14, 127.10, 127.04, 126.45, 113.63, 103.38, 101.12, 101.06, 83.03, 81.83, 77.54, 76.68, 75.47, 74.67, 74.30, 73.12, 72.78, 71.41, 70.23, 69.81, 68.78, 68.29, 67.08, 66.58, 55.16, 50.37, 50.05, 47.07, 46.10, 29.52, 27.95, 27.57, 26.50, 25.83, 25.76. HRMS (ESI) m/z of $C_{76}H_{81}NO_{15}$ $[M+H]^+$: calcd 1247.5606, found 1247.5599.

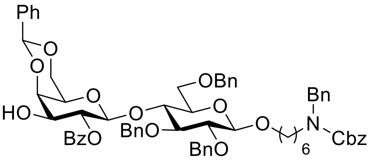

6

Compound 6. To a solution of compound **5** (3.82 g, 3.06 mmol) in MeCN (97 mL) and H_2O (4.9 mL) was added CAN (6.71 g, 12.23 mmol, 4.0 equiv.) at 0 ºC. The reaction mixture was gradually warmed to rt and then stirred for 1 h. The mixture was dissolved in EtOAc and the resulting organic solvent was washed by H_2O, $NaHCO_{3(aq)}$, and brine. The organic layer was dried over $MgSO_4$, filtered, and concentrated. The residue was purified by silica gel chromatography (EtOAc/Hexane = 1/1, R_f = 0.38) to afford compound **6** (2.68 g, 78%). $[\alpha]_D^{24}$ = -5.85 (c = 2.0, $CHCl_3$); ^{1}H NMR (850 MHz, $CDCl_3$) δ 7.99 (d, *J* = 7.6 Hz, 2H, Ph), 7.55 (t, *J* = 7.4 Hz, 1H, Ph), 7.55-7.45 (m, 2H, Ph), 7.44-7.39 (m, 4H, Ph), 7.36-7.24 (m, 17H, Ph), 7.24-7.11 (m, 9H, Ph), 5.50 (s, 1H, $CH_{Benzylidene}$), 5.30 (dd, *J* = 9.6, 8.4 Hz, 1H, H-2'), 5.14-5.12 (m, 2H, $PhCH_2$), 5.08 (d, *J* = 10.7 Hz, 1H, PhCH), 4.87-4.81 (m, 1H, PhCH), 4.83 (d, *J* = 10.8 Hz, 1H, PhCH), 4.74 (d, *J* = 8.4 Hz, 1H, H-1'), 4.67 (d, *J* = 12.1 Hz, 1H, PhCH), 4.55 (d, *J* = 12.1 Hz, 1H, PhCH), 4.46-4.41 (m, 2H, $PhCH_2$), 4.27-4.24 (m, 2H, PhCH, H-1),

4.19 (d, J = 12.1 Hz, 1H, H-6a’), 4.10 (d, J = 3.4 Hz, 1H, H-4’), 3.90 (t, J = 9.3 Hz, 1H, H-4), 3.83-3.84 (m, 1H, H-6b’), 3.84-3.78 (m, 1H, CH_{linker}), 3.65 (dd, J = 10.9, 4.3 Hz, 1H, H-6a), 3.64-3.61 (m, 1H, H-3’), 3.61-3.57 (m, 2H, H-3, H-6b), 3.42-3.35 (m, 1H, CH_{linker}), 3.37 (t, J = 8.5 Hz, 1H, H-2), 3.26-3.23 (m, 1H, H-5), 3.22-3.18 (m, 1H, CH_{linker}), 3.15-3.10 (m, 1H, CH_{linker}), 3.09 (s, 1H, H-5’), 2.49 (d, J = 10.3 Hz, 1H, OH), 1.54-1.38 (m, 4H, $2\times CH_{2linker}$), 1.35-1.19 (m, 4H, $2\times CH_{2linker}$); ^{13}C NMR (214 MHz, $CDCl_3$) δ 165.84 (CO_{Bz}), 156.67 (CO_{Cbz}), 156.09 (CO_{Cbz}), 139.00, 138.52, 138.30, 137.89, 137.84, 137.40, 136.81, 136.72, 133.18, 129.74, 129.68,129.17, 128.44, 128.39, 128.30, 128.20, 128.16, 128.12, 127.99, 127.88, 127.73, 127.69, 127.64, 127.45, 127.19, 127.15, 127.09, 126.41, 103.41, 101.42, 100.61, 83.01, 81.81, 77.55, 77.20, 75.54, 75.49, 74.70, 74.34, 73.42, 73.22, 71.84, 69.80, 68.64, 68.31, 67.07, 66.50, 50.37, 50.05, 47.06, 46.09, 29.53, 27.96, 27.57, 26.50, 25.82, 25.76. HRMS (ESI) m/z of $C_{68}H_{73}NO_{14}$ $[M+H]^+$: calcd 1127.5031, found 1127.5025.

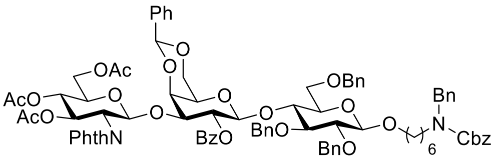

7

Compound 7. A mixture of the acceptor **6** (2.68 g, 2.38 mmol, 1 equiv.), donor **3** (1.54 g, 2.85 mmol, 1.2 equiv.), and 3Å flame-dried molecular sieves was stirred in CH_2Cl_2 (105 mL) under a nitrogen atmosphere at rt for 30 min. The reaction mixture was then cooled to -78 ^oC, and then NIS (641 mg, 2.85 mmol, 1.2 equiv.) was added to the solution followed by slow addition of TMSOTf (65 μL, 0.36 mmol, 0.15 equiv.). The reaction mixture was stirred for 1 h, allowing the temperature to rise from -78 °C to -60 °C, and then was quenched with Et_3N. The reaction mixture was filtered through a pad of Celite® and the resulting solution was concentrated under reduced pressure. The residue was dissolved in EtOAc and the resulting solution was washed with saturated $Na_2S_2O_{3(aq)}$, $NaHCO_3$, and then brine. The organic layer was dried over $MgSO_4$, filtered, and concentrated. The residue was purified by silica gel chromatography. (EtOAc/Hexane = 5/4, R_f = 0.38) to give compound **7** (3.34 g, 91%). $[\alpha]_D^{25}$ = 0.99 (c = 1.8, $CHCl_3$); ^1H NMR (400 MHz, $CDCl_3$) δ 7.54-7.48 (m, 4H), 7.48-7.39 (m, 2H), 7.38-7.24 (m, 18H), 7.23-7.17 (m, 10H), 7.16-7.09 (m, 4H), 5.64 (dd, J = 10.7, 9.0 Hz, 1H), 5.50 (d, J = 8.6 Hz, 1H), 5.49 (br, 1H), 5.32 (dd, J = 10.1, 8.0 Hz, 1H), 5.16-5.09 (m, 3H), 5.00 (d, J = 11.0 Hz, 1H), 4.78 (d, J = 11.0 Hz, 1H), 4.69 (d, J = 11.0 Hz, 1H), 4.61 (d, J = 11.0 Hz, 1H), 4.60 (d, J = 8.0 Hz, 1H), 4.49 (d, J = 12.2 Hz, 1H), 4.44-4.39 (m, 2H), 4.37 (dd, J = 12.3, 2.6 Hz, 1H), 4.33 (dd, J = 10.7, 8.4 Hz, 1H), 4.28 (d, J = 3.6 Hz, 1H), 4.22-4.10 (m, 4H), 3.91-3.82 (m, 2H), 3.77 (t, J = 9.3 Hz, 1H), 3.71-3.68 (m, 1H), 3.65 (dd, J = 10.1, 3.6 Hz, 1H), 3.47 (t, J = 9.0 Hz, 1H), 3.38-3.23 (m, 4H), 3.21-3.13 (m, 1H), 3.08 (br, 2H), 2.99-2.92 (m, 1H), 2.03 (s, 3H), 2.00 (s, 3H), 1.73 (s, 3H), 1.52-1.35 (m, 4H), 1.23-1.08 (m, 4H); ^{13}C NMR (101 MHz, $CDCl_3$) δ 170.36 (CO_{Ac}), 169.93 (CO_{Ac}), 169.25 (CO_{Ac}), 167.62

(CO $_{Phth}$), 166.83 (CO $_{Phth}$), 163.99 (CO $_{Bz}$), 156.58 (CO $_{Cbz}$), 156.01 (CO $_{Cbz}$), 138.91, 138.48, 138.34, 137.76, 136.68, 133.65, 132.65, 130.60, 129.33, 129.06, 128.52, 128.36, 128.29, 128.25, 128.06, 127.93, 127.87, 127.76, 127.65, 127.30, 127.10, 126.96, 126.25, 122.97, 103.22, 100.68, 100.48, 99.13, 82.83, 81.59,79.08, 76.84, 75.55, 75.37, 74.55, 74.05, 73.11, 71.74, 70.82, 70.32, 69.64, 68.70, 68.52, 67.94, 66.97, 66.52, 61.74, 54.23, 50.27, 49.97, 46.97, 46.00, 29.40, 27.85, 27.47, 26.39, 25.66, 20.72, 20.50, 20.17. HRMS (ESI) m/z of $C_{88}H_{92}N_2O_{23}$ $[M+H]^+$: calcd 1544.6091, found 1544.6109.

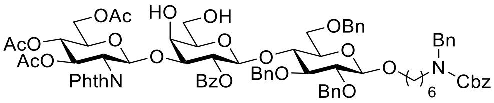

8

Compound 8. To a solution of compound **7** (457 mg, 0.30 mmol) in MeOH (5 mL) and CH_2Cl_2 (5 mL) was added *p*-TsOH (765 mg, 4.44 mmol, 15.0 equiv.) at 0 ºC. The reaction mixture was gradually warmed to rt and stirred for 1 h. The reaction was quenched with Et_3N and the resulting solution was concentrated under reduced pressure. The residue was extracted with EtOAc and the resulting organic solution was washed with H_2O and brine. The organic layer was dried over $MgSO_4$, filtered, and concentrated. The residue was purified by silica gel chromatography (EtOAc/Hexane = 3/2, R_f = 0.2) to afford compound **8** (333 mg, 77%). $[\alpha]_D^{25}$ = 21.01 (c = 0.3, $CHCl_3$); ^1H NMR (850 MHz, $CDCl_3$) δ 7.45 (d, *J* = 7.6 Hz, 2H), 7.44-7.40 (br, 2H), 7.38-7.32 (m, 7H), 7.31-7.24 (m, 12H), 7.24-7.17 (m, 8H), 7.14-7.09 (m, 3H), 5.65 (dd, *J* = 10.4, 9.4 Hz, 1H), 5.49 (d, *J* = 8.4 Hz, 1H), 5.27 (t, *J* = 8.9 Hz, 1H), 5.12 (d, *J* = 9.9 Hz, 2H), 5.07 (t, *J* = 9.6 Hz, 1H), 4.87 (d, *J* = 10.3 Hz, 1H), 4.82-4.76 (m, 1H), 4.68 (d, *J* = 10.3 Hz, 1H), 4.64 (d, *J* = 11.2 Hz, 1H), 4.56 (d, *J* = 12.0 Hz, 1H), 4.49 (d, *J* = 8.1 Hz, 1H), 4.44-4.40 (m, 2H), 4.30 (dd, *J* = 10.6, 8.5 Hz, 1H), 4.26 (dd, *J* = 11.9, 2.0 Hz, 1H), 4.22-4.16 (m, 2H), 4.14-4.10 (m, 1H), 4.03 (br, 1H), 3.90 (ddd, *J* = 10.0, 5.7, 2.1 Hz, 1H), 3.78 (t, *J* = 9.4 Hz, 1H), 3.74-3.65 (m, 1H), 3.68 (dd, *J* = 11.8, 7.4 Hz, 1H), 3.58-3.55 (m, 2H), 3.39 (t, *J* = 9.1 Hz, 1H), 3.37-3.34 (m, 2H), 3.33-3.27 (m, 1H), 3.28 (d, *J* = 10.1 Hz, 1H), 3.25 (t, *J* = 8.5 Hz, 1H), 3.18 (br, 1H), 3.11 (br, 1H), 2.95 (dd, *J* = 9.7, 1.5 Hz, 1H), 2.73-2.71 (m, 1H), 2.10 (s, 3H), 2.00 (s, 3H), 1.74 (s, 3H), 1.51-1.38 (m, 4H), 1.28-1.19 (m, 4H); ^{13}C NMR (214 MHz, $CDCl_3$) δ 170.45 (CO $_{Ac}$), 169.71 (CO $_{Ac}$), 169.14 (CO $_{Ac}$), 167.50 (CO_{Phth}), 166.34 (CO_{Phth}), 163.97 (CO_{Bz}), 156.44 (CO_{Cbz}), 155.87 (CO_{Cbz}), 138.46, 138.34, 137.99, 137.64, 136.54,133.72, 132.52, 130.32, 129.03, 128.70, 128.23,128.16, 127.95, 127.91, 127.62, 127.51, 127.21, 126.98,122.84, 103.13, 99.80, 98.35, 82.31, 81.67, 81.18, 75.86 75.41, 74.39,74.17, 73.92, 73.11, 71.79, 70.78, 70.01, 69.42, 68.61, 67.81, 67.36, 66.82, 61.81, 61.29, 54.01, 50.14, 49.83, 46.84, 45.88, 29.25, 27.71, 27.34, 26.23, 25.52, 20.48, 20.32, 19.99. HRMS (ESI) m/z of $C_{81}H_{88}NO_{23}$ $[M+H]^+$: calcd 1456.5778, found 1456.5748.

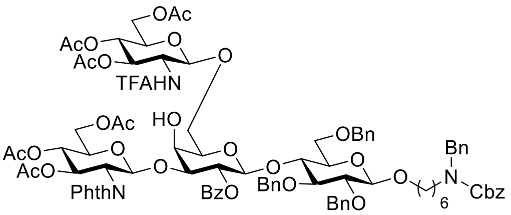

9

Compound 9. A mixture of the acceptor **8** (2 g, 1.37 mmol, 1 equiv.), donor **4** (836 mg, 0.96 mmol, 1.2 equiv.) and 3Å flame-dried molecular sieves was stirred in CH_2Cl_2 (50 mL) under nitrogen atmosphere at rt for 30 min. The reaction mixture was then cooled to -78 ^{o}C, and then NIS (216 mg, 0.96 mmol, 1.2 equiv.) was added to the solution followed by slow addition of TMSOTf (90 µL, 0.49 mmole, 0.3 equiv.). The reaction mixture was stirred for 1 h, allowing the temperature to rise from -78 °C to -60 °C, and then was quenched with Et_3N. The reaction mixture was filtered through a pad of Celite® and the resulting solution was concentrated under reduced pressure. The residue was dissolved in EtOAc and the resulting solution was washed with saturated $Na_2S_2O_{3(aq)}$, $NaHCO_3$, and then brine. The organic layer was dried over $MgSO_4$, filtered, and concentrated. The residue was purified by silica gel chromatography (EtOAc/Hexane = 5/4, R_f = 0.25) to give compound **9** (1.89 g, 75%). $[\alpha]_D^{25}$ = -7.13 (c = 1.0, $CHCl_3$); ^{1}H NMR (400 MHz, $CDCl_3$) δ 7.47-7.44 (m, 2H), 7.44-7.41 (dd, *J* = 5.4, 3.1 Hz, 2H), 7.41-7.36 (m, 6H), 7.35-7.24 (m, 17H, Ph), 7.24-7.17 (m, 4H, Ph), 7.13-7.09 (m, 3H), 5.67 (dd, *J* = 10.7, 9.1 Hz, 1H), 5.47 (d, *J* = 8.5 Hz, 1H), 5.26 (dd, *J* = 9.8, 8.1 Hz, 1H), 5.12 (br, 1H), 5.08 (dd, *J* = 10.1, 9.4 Hz, 1H), 5.04-4.97 (m, 2H), 4.94 (d, *J* = 10.9 Hz, 1H), 4.85 (d, *J* = 11.0 Hz, 1H), 4.68 (d, *J* = 11.0 Hz, 2H), 4.64 (d, *J* = 12.4 Hz, 1H), 4.48-4.40 (m, 4H), 4.31-4.19 (m, 4H), 4.14 (br, 1H), 4.07-3.88 (m, 6H), 3.80 (t, *J* = 9.1 Hz, 1H), 3.72 (br, 1H), 3.54-3.46 (m, 3H), 3.39-3.29 (m, 4H), 3.23-3.10 (m, 4H), 2.90-2.87 (m, 1H), 2.74 (br, 1H), 2.13 (s, 3H), 2.02 (s, 3H), 2.01 (s, 3H), 1.96 (s, 3H), 1.81 (s, 3H), 1.74 (s, 3H), 1.55-1.38 (m, 4H), 1.29-1.15 (m, 4H); ^{13}C NMR (101 MHz, $CDCl_3$) δ 170.44 (x 2, CO_{Ac}), 170.29 (CO_{Ac}), 169.61 (CO_{Ac}), 169.10 (CO_{Ac}), 168.89 (CO_{Ac}), 167.46 (CO_{Phth}), 166.20 (CO_{Phth}), 163.83 (CO_{Bz}), 157.30 (d, *J* = 37.3 Hz, *C*OCF_3) , 156.40 (CO_{Cbz}), 155.81 (CO_{Cbz}), 138.97, 138.43, 137.69, 137.57, 136.48, 133.70, 132.50, 130.25, 128.96, 128.50, 128.19, 128.10, 127.95, 127.88, 127.58, 127.43, 127.25, 126.93, 126.84, 122.86, 115.58 (d, *J* = 289.13, CF_3), 103.08, 100.04, 99.67, 98.22, 82.96, 81.62, 81.18, 75.69, 75.48, 74.59, 73.68, 73.11, 71.79, 71.66, 70.84, 70.30, 69.95, 69.38, 68.55, 67.73, 66.78 61.79, 61.11, 53.94, 50.08, 49.80, 46.80, 45.86, 29.17, 27.64, 27.28, 26.16, 25.39, 20.51, 20.39, 20.24, 20.14, 20.10, 19.90. HRMS (ESI) m/z of $C_{95}H_{104}F_3N_3O_{31}$ $[M+H]^+$: calcd 1839.6606, found1839.6506.

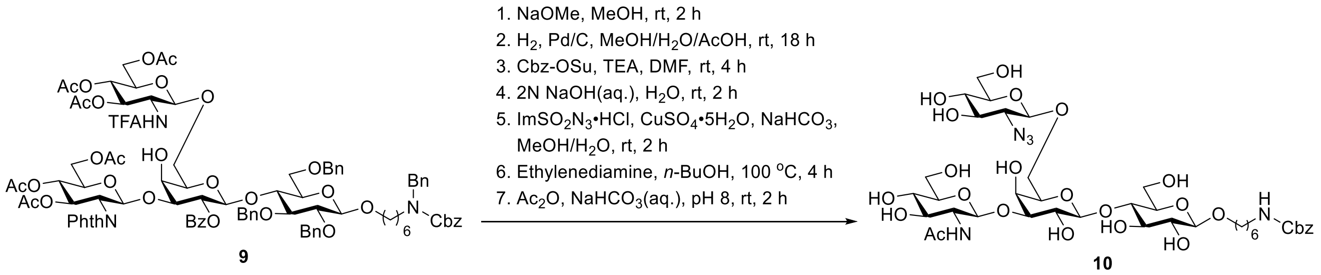

Compound 10. Compound **10** was synthesized from compound **9** by following 7-steps global deprotection and *N*-modification. **Step 1 (Removing ester type protecting group)**: To a solution of compound **9** (1.03 g, 0.56 mmol) in anhydrous MeOH (20 mM) was added NaOMe (4.0 equiv.) under nitrogen atmosphere. The reaction mixture was stirred at rt for 2 h and then quenched with Amberlite® IR-120 (H^+) resin. The suspension was then filtered and the solution was concentrated under reduced pressure. **Step 2 (Removing ether type protecting group)**: Without further purification, the above residue was dissolved in solution of MeOH/H_2O/AcOH (10/2/1, 20 mM). The solution was cooled to 0 °C, and then palladium (10 wt.% on carbon, 0.5 eq.) was added dropwise. The reaction was gradually warmed to rt and then stirred under H_2 atmosphere. The resulting solution was filtered through Celite and concentrated. **Step 3 (Cbz protection of the linker amine)**: Without further purification, the above deprotected intermediate was dissolved in DMF (20 mM). TEA (6.0 equiv.) and Cbz-OSu (4.0 equiv.) were added dropwise at rt. After completion of the starting material as indicated by the TLC analysis, the reaction was concentrated under reduced pressure. The residue was purified by RP C-18 gel chromatography (MeOH/H_2O = 1/2 to 4/5, every elution contains 5 mM ammonium bicarbonate) and the fractions with product were concentrated under reduced pressure to afford *N*-carboxybenzyl intermediate. **Step 4 (Removing *N*-TFA protecting group)**: A solution of above residue in H_2O/2N NaOH (20/1, 20 mM) was stirred at rt. After completion of the starting material as indicated by the TLC analysis, the mixture was purified by RP C-18 gel chromatography (MeOH/H_2O = 1/5 to 4/5, every elution contains 1% AcOH). The eluents containing product were concentrated under reduced pressure to afford amine intermediate. **Step 5 (Transformation of amine to azide)**: To a solution of above amine intermediate in H_2O/MeOH (1/1, 20 mM) was added $NaHCO_3$ (8.0 equiv.), $CuSO_4 \cdot 5H_2O$ (0.1 equiv.) and imidazole-1-sulfonyl azide hydrochloride (4.0 equiv.). The solution was stirred at rt. After disappearance of starting material as indicated by TLC analysis, the solvent was removed under reduced pressure. The residue was purified by RP C-18 gel chromatography (MeOH/H_2O = 1/5 to 7/10, every elution contains 5 mM ammonium bicarbonate). The eluents containing product were concentrated under reduced pressure to afford azide intermediate. **Step 6 (Removal of *N*-Phth and *N*HPhth protecting groups)**: A solution of above azide intermediate in ethylenediamine/*n*-BuOH (1/4) was stirred at 100 ºC. After completion of starting material as indicated by the TLC analysis, the solvent was removed under reduced pressure. The residue was purified by RP C-18 gel

chromatography ($MeOH/H_2O$ = 1/5 to 4/5, every elution contains 1% AcOH). The eluents containing product were concentrated under reduced pressure to afford amine intermediate. **Step 7 (Acetylation of $GlcNH_2$)**: A solution of above intermediate in $NaHCO_{3(aq)}$ (10 mM, pH 8.2) was added Ac_2O (20.0 equiv.) dropwise at rt. After completion of amine intermediate as indicated by the TLC analysis, the solvent was removed under reduced pressure. The resulting crude was purified by RP C-18 gel chromatography ($MeOH/H_2O$ = 1/5 to 4/5) and further purified by Toyopearl HW-40F column. The eluents containing product were concentrated under reduced pressure to afford compound **10** (254 mg, 47%).^{1}H NMR (400 MHz, D_2O) δ 7.45-7.38 (m, 5H), 5.09 (s, 2H), 4.84 (d, *J* = 8.1 Hz, 1H), 4.66 (d, *J* = 8.4 Hz, 1H), 4.47 (d, *J* = 7.9 Hz, 1H), 4.45 (d, *J* = 8.4 Hz, 1H), 4.15 (d, *J* = 3.2 Hz, 1H), 4.00-3.94 (m, 2H), 3.94-3.84 (m, 6H), 3.80-3.68 (m, 5H), 3.66-3.60 (m, 2H), 3.59-3.51 (m, 3H), 3.50-3.29 (m, 5H), 3.37-3.28 (m, 2H), 3.10 (t, *J* = 6.5 Hz, 2H), 2.02 (s, 3H), 1.64-1.55 (m, 2H), 1.51-1.42 (m, 2H), 1.37-1.27 (m, 4H); ^{13}C NMR (101 MHz, $CDCl_3$) δ 175.88 (CO_{Ac}), 159.41 (CO_{Cbz}), 137.63, 129.73, 129.27, 128.55, 103.84, 103.45, 103.05, 102.74, 82.83, 77.89, 76.83, 76.64, 75.82, 75.25, 75.18, 75.03, 74.54, 74.02, 71.44, 70.97, 70.66, 70.57, 69.84, 69.39, 67.64, 66.62, 61.57, 61.47, 60.78, 56.64, 41.28, 29.58, 26.47, 25.60, 23.13. HRMS (ESI) m/z of $C_{40}H_{63}N_5O_{22}$ $[M+H]^+$: calcd 965.3965, found 965.4002.

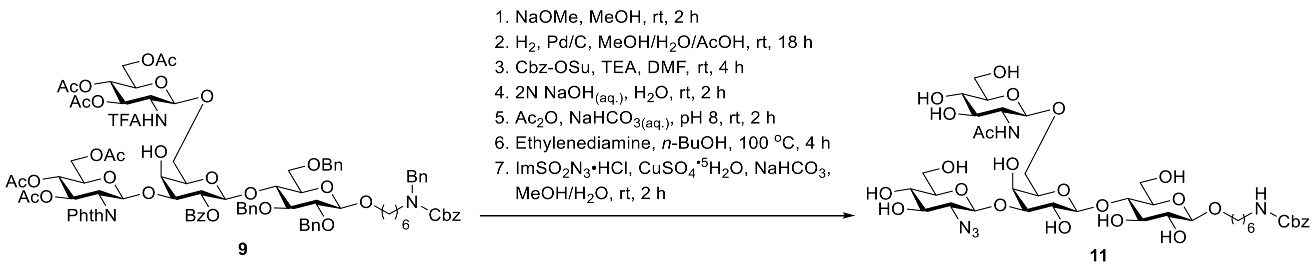

Compound 11. Compound **11** was synthesized from compound **9** by following 7-steps global deprotection and *N*-modification. The first four steps performed on compound **11** was applied to compound **9** (55 mg, 0.56 mmol). **Step 5 (Acetylation of $GlcNH_2$)**: A solution of above intermediate in $NaHCO_{3(aq)}$ (10 mM, pH 8.2) was added Ac_2O (20.0 equiv.) dropwise at rt. After completion of amine intermediate as indicated by the TLC analysis, the solvent was removed under reduced pressure. The resulting crude was purified by RP C-18 gel chromatography ($MeOH/H_2O$ = 1/5 to 4/5). **Step 6 (Removal of *N*-Phth and *N*HPhth protecting groups)**: A solution of above azide intermediate in ethylenediamine/*n*-BuOH (1/4) was stirred at 100 °C. After completion of starting material as indicated by the TLC analysis, the solvent was removed under reduced pressure. The residue was purified by RP C-18 gel chromatography ($MeOH/H_2O$ = 1/5 to 4/5, every elution contains 1% AcOH). The eluents containing product were concentrated under reduced pressure to afford amine intermediate. **Step 7 (Transformation of amine to azide)**: To a solution of above amine intermediate in H_2O/MeOH (1/1, 20 mM) was added $NaHCO_3$ (8.0 equiv.), $CuSO_4{\cdot}5H_2O$ (0.1 equiv.), and

imidazole-1-sulfonyl azide hydrochloride (4.0 equiv.). The solution was stirred at rt. After disappearance of starting material as indicated by TLC analysis, the solvent was removed by under reduced pressure. The residue was purified by RP C-18 gel chromatography ($MeOH/H_2O$ = 1/5 to 7/10) and further purified by Toyopearl HW-40F column. The eluents containing product were concentrated under reduced pressure to afford compound **11** (15 mg, 50%).^{1}H NMR (500 MHz, D_2O) δ 7.56-7.30 (m, 5H), 5.12 (s, 2H), 4.77 (1H, H-1 $GlcN_3$ merged in solvent residual peak), 4.63 (d, *J* = 8.6 Hz, 1H), 4.47 (d, *J* = 7.8 Hz, 2H), 4.16 (d, *J* = 2.9 Hz, 1H), 4.03-3.99 (m, 1H), 3.96 (d, *J* = 8.4 Hz, 1H), 3.94-3.88 (m, 3H), 3.87-3.80 (m, 4H), 3.78-3.66 (m, 5H), 3.66-3.55 (m, 5H), 3.48-3.43 (m, 4H), 3.42-3.37 (m, 1H), 3.32 (t, *J* = 8.4 Hz, 1H), 3.13 (t, *J* = 6.2 Hz, 2H), 2.07 (s, 3H), 1.64-1.58 (m, 2H), 1.54-1.46 (m, 2H), 1.40-1.28 (m, 4H); ^{13}C NMR (126 MHz, D_2O) δ 175.51 (CO_{Ac}), 159.42 (CO_{Cbz}), 137.63, 129.73, 129.27, 128.54, 104.02, 103.94, 102.97, 102.03, 82.42, 79.97, 76.83, 76.73, 75.65, 75.44, 74.90, 74.81, 74.42, 73.85, 71.49, 70.97, 70.84, 70.26, 69.48, 69.28, 67.65, 66.70, 61.65, 61.34, 60.99, 56.46, 41.28, 29.62, 29.57, 26.45, 25.58, 23.37. HRMS (ESI) m/z of $C_{40}H_{63}N_5O_{22}$ $[M+H]^+$: calcd 965.3965, found 965.3967.

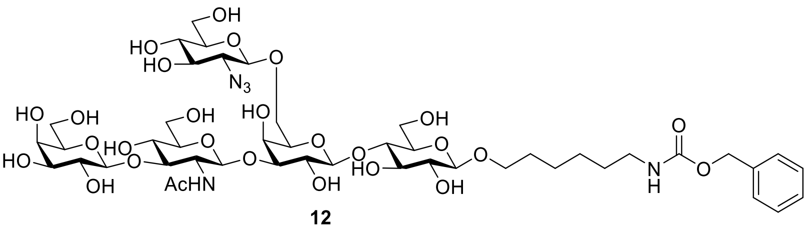

12

Compound 12. Compound **10** (58.0 mg, 60.1 µmol) and Gal (16.2 mg, 90.0 µmol) were used as the acceptor and the donor precursor, respectively, by following the general enzymatic synthetic procedure SNRS-G3. After being shaken for 2 h, the reaction was quenched and the mixture was purified by following general purification procedure 1. After purification, the product was lyophilized to give compound **12** in 97% yield (60.3 mg). R_f = 0.64 (EA/ MeOH/ H_2O/AcOH = 7/4/2/1 (v/v/v/v)); ^{1}H NMR (850 MHz, D_2O) δ 7.56-7.39 (m, 5H), 5.11 (s, 2H), 4.85 (d, *J* = 8.2 Hz, 1H), 4.73 (d, *J* = 8.5 Hz, 1H), 4.49 (d, *J* = 7.8 Hz, 1H), 4.46 (d, *J* = 7.7 Hz, 1H), 4.45 (d, *J* = 7.8 Hz, 1H), 4.17 (d, *J* = 3.1 Hz, 1H), 4.02 (dd, *J* = 12.3, 3.6 Hz, 1H), 3.99 (dd, 12.0, 8.2 Hz, 1H), 3.97-3.906 (m, 7H), 3.88 (t, *J* = 9.4 Hz, 1H), 3.82 (t, *J* = 9.4 Hz, 1H), 3.80-3.71 (m, 7H), 3.69-3.64 (m, 3H), 3.61 (dd, *J* = 9.5, 8.1 Hz, 1H), 3.58 (t, *J* = 9.7 Hz, 1H), 3.57-3.47 (m, 4H), 3.46-3.44 (m, 1H), 3.42 (t, *J* = 8.8 Hz, 1H), 3.36 (dd, *J* = 9.7, 8.3 Hz, 1H), 3.33 (t, *J* = 9.0 Hz, 1H), 3.12 (t, *J* = 6.3 Hz, 2H), 2.04 (s, 3H), 1.66-1.56 (m, 2H), 1,49 (quin, *J* = 7.2 Hz, 2H), 1.40-1.27 (4H); ^{13}C NMR (214 MHz, D_2O) δ 175.92, 159.41, 137.63, 129.73 (×2), 129.27, 128.54 (×2), 104.45, 103.55, 103.43, 103.04, 102.74, 83.02, 82.87, 77.88, 76.82, 76.24, 76.15, 75.82, 75.24, 75.17, 75.02, 74.01, 73.44, 71.65, 71.45, 70.96, 70.56, 69.83, 69.50, 69.41, 69.34, 67.64, 66.60, 61.99, 61.56, 64.47, 60.77, 55.67, 41.27, 29.63, 29.57, 26.46, 25.59, 23.19; HRMS (ESI) *m/z* calcd for $C_{46}H_{72}N_5O_{27}$ $[M-H]^-$:

1126.4415; found 1126.4385.

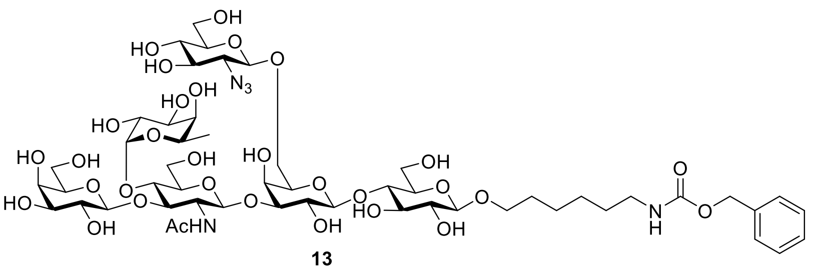

13

Compound 13. Compound **12** (36.4 mg, 32.3 μmol) and GDP-Fuc (30.7 mg, 48.4 μmol) were used as the acceptor and the donor, respectively, by following the general procedure for DUSD-F3/4. After being shaken for 23 h, the reaction was quenched and purified by following general purification procedure 1. After purification, the product was lyophilized to give compound **13** in 93% yield (38.3 mg). R_f = 0.21 (EA/ MeOH/ H_2O/AcOH = 10/4/2/1 (v/v/v/v)); ^1H NMR (850 MHz, D_2O) δ 7.55-7.37 (m, 5H), 5.11 (br, 2H), 5.04 (d, *J* = 3.9 Hz, 1H), 4.89 (q, *J* = 6.5 Hz, 1H), 4.85 (d, *J* = 8.2 Hz, 1H), 4.70 (d, *J* = 8.4 Hz, 1H), 4.51 (d, *J* = 7.7 Hz, 1H), 4.48 (d, *J* = 7.8 Hz, 1H), 4.46 (d, *J* = 7.9 Hz, 1H), 4.17 (d, *J* = 3.2 Hz, 1H), 4.09 (t, *J* = 9.8 Hz, 1H), 4.02 (dd, *J* = 12.2, 3.7 Hz, 1H), 3.99 (dd, *J* = 12.2, 8.2 Hz, 1H), 3.98-3.85 (m, 10H), 3.82-3.72 (m, 8H), 3.65 (t, *J* = 8.0 Hz, 2H), 3.63 (dd, *J* = 9.9, 3.4 Hz, 1H), 3.60 (dd, *J* = 9.6, 8.0 Hz, 1H), 3.58-3.54 (m, 3H), 3.53-3.49 (m, 2H), 3.47-3.44 (m, 1H), 3.42 (t, *J* = 8.8 Hz, 1H), 3.36 (dd, *J* = 9.8, 8.2 Hz, 1H), 3.33 (t, *J* = 8.9 Hz, 1H), 3.12 (t, *J* = 6.4 Hz, 2H), 2.04 (s, 3H), 1.67-1.56 (m, 2H), 1.49 (quin, *J* = 7.2, 2H), 1.43-1.28 (m, 4H), 1.19 (d, *J* = 6.5 Hz, 3H); ^{13}C NMR (214 MHz, D_2O) δ 175.73, 159.42, 137.63, 129.73 (×2), 129.26, 128.53 (×2), 103.82, 103.62, 103.45, 103.04, 102.73, 98.97, 82.97, 77.88, 76.87, 76.82, 76.18, 75.81, 75.78, 75.23, 75.13, 75.02, 74.01, 73.29, 73.07, 72.91, 71.47 (×2), 70.91, 70.56, 70.10, 69.80, 69.31, 68.76, 67.81, 67.80, 67.64, 66.61, 62.61, 61.56, 60.76, 60.58, 56.84, 41.27, 29.62, 29.57, 26.45, 25.59, 23.28, 16.33; HRMS (ESI) *m/z* calcd for $C_{52}H_{82}N_5O_{31}$ $[M-H]^-$: 1272.4994; found 1272.4984.

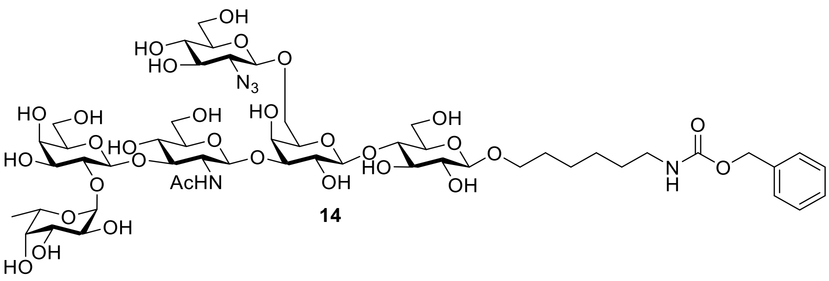

14

Compound 14. Compound **12** (44.3 mg, 39.3 μmol) and GDP-Fuc (37.3 mg, 58.9 μmol) were used as the acceptor and the donor, respectively, by following the general procedure for DUSD-F2. After being shaken for 18 h, the reaction was quenched and the mixture was purified by following **general purification procedure 1**. After purification, the product was

lyophilized to give compound **14** in 96% yield (47.8 mg). R_f = 0.5 (EA/ MeOH/ H_2O/AcOH = 7/4/2/1 (v/v/v/v));^{1}H NMR (850 MHz, D_2O) δ 7.46-7.40 (m, 5H), 5.20 (d, J = 4.1 Hz, 1H), 5.11 (br, 2H), 4.86 (d, J = 8.2 Hz, 1H), 4.66 (d, J = 7.7 Hz, 1H), 4.62 (d, J = 8.4 Hz, 1H), 4.47 (d, J = 7.8 Hz, 1H), 4.47 (d, J = 8.0 Hz, 1H), 4.30 (q, J = 6.6 Hz, 1H), 4.16 (d, J = 3.2 Hz, 1H), 4.03-3.98 (m, 3H), 3.96-3.87 (m, 8H), 3.85-3.83 (m, 2H), 3.82-3.77 (m, 4H), 3.76-3.72 (m, 4H), 3.69-3.64 (m, 4H), 3.60 (dd, J = 9.6, 7.9 Hz, 1H), 3.58-3.49 (m, 5H), 3.46-3.44 (m, 1H), 3.42 (dd, J = 9.6, 8.9 Hz, 1H), 3.36 (dd, J = 9.6, 8.5 Hz, 1H), 3.33 (dd, J = 8.9, 8.6 Hz, 1H), 3.12 (t, J = 6.4 Hz, 1H), 2.07 (s, 3H), 1.63-1.60 (m, 2H), 1.49 (quin, J = 7.1 Hz, 2H), 1.38-1.30 (m, 4H), 1.25 (d, J = 6.6 Hz, 3H); ^{13}C NMR (214 MHz, D_2O) δ 175.20, 159.43, 137.64, 129.74 (×2), 129.26, 128.53 (×2), 104.25, 103.48, 103.04, 102.75, 101.22, 100.46, 82.49, 78.14, 77.76, 77.62, 76.82, 76.22, 76.04, 75.83, 75.22, 75.15, 75.01, 74.46, 74.01, 72.81, 71.45, 71.16, 70.56, 70.40, 70.08, 69.85, 69.64, 69.43, 69.01, 67.65, 67.46, 66.60, 62.11, 61.56, 61.38, 60.76, 55.93, 41.27, 29.62, 29.57, 26.45, 25.59, 23.11, 16.22; HRMS (ESI) *m/z* calcd for $C_{52}H_{82}N_5O_{31}$ $[M-H]^-$: 1272.4994; found 1272.4990.

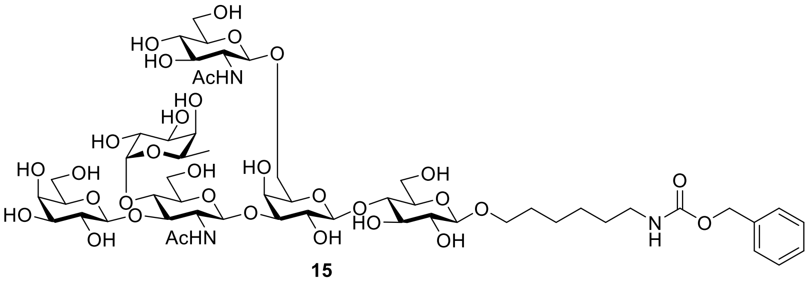

15

Compound 15. The CC1 procedure was applied on compound **13** (30 mg, 23.6 µmol) to give compound **15** in 92% yield (28 mg). R_f = 0.30 (EA/MeOH/H_2O/AcOH = 7/4/2/1 (v/v/v/v)); ^{1}H NMR (850 MHz, D_2O) δ 7.46-7.40 (m, 5H), 5.11 (br, 2H), 5.04 (d, J = 3.8 Hz, 1H), 4.88 (q, J = 6.7 Hz, 1H), 4.71 (d, J = 8.3 Hz, 1H), 4.62 (d, J = 8.5 Hz, 1H), 4.52 (d, J = 7.7 Hz, 1H), 4.47 (d, J = 7.8 Hz, 1H), 4.30 (d, J = 7.9 Hz, 1H), 4.15 (d, J = 2.9 Hz, 1H), 4.09 (t, J = 9.7 Hz, 1H), 4.00 (dd, J = 10.2, 2.7 Hz, 1H), 3.96-3.94 (m, 3H), 3.93 (dd, J = 12.4, 1.6 Hz, 1H), 3.90-3.87 (m, 4H), 3.85-3.69 (m, 11), 3.67-3.62 (m, 3H), 3.60-3.54 (m, 6H), 3.50 (dd, J = 9.5, 7.9 Hz, 1H), 3.47 (dd, J = 5.5, 1.7 Hz, 1H), 3.45 (t, J = 9.3 Hz, 1H), 3.31 (t, J = 8.7 Hz, 1H), 3.12 (t, J = 6.3 Hz, 1H), 2.06 (s, 3H), 2.04 (s, 3H), 1.62-1.60 (m, 2H), 1.49 (quin, J = 7.1 Hz, 2H), 1.36-1.32 (m, 4H), 1.19 (d, J = 6.5 Hz, 3H); ^{13}C NMR (214 MHz, D_2O) δ 175.70, 175.49, 159.42, 137.63, 129.73, 129.26, 128.52, 103.99, 103.82, 103.55, 102.96, 102.01, 98.97, 82.81, 79.94, 76.88, 76.83, 76.19, 75.78, 75.62, 75.44, 74.84, 74.42, 73.84, 73.29, 73.06, 72.91, 71.49, 71.46, 70.84, 70.77, 70.10, 69.58, 69.31, 68.76, 67.80, 67.64, 62.61, 61.65, 61.01, 60.57, 56.84, 56.46, 41.27, 29.61, 29.56, 26.45, 25.58, 23.38, 23.25, 16.33; HRMS (ESI) *m/z* calcd for $C_{54}H_{86}N_3O_{32}$ $[M-H]^-$: 1288.5194; found 1288.5264.

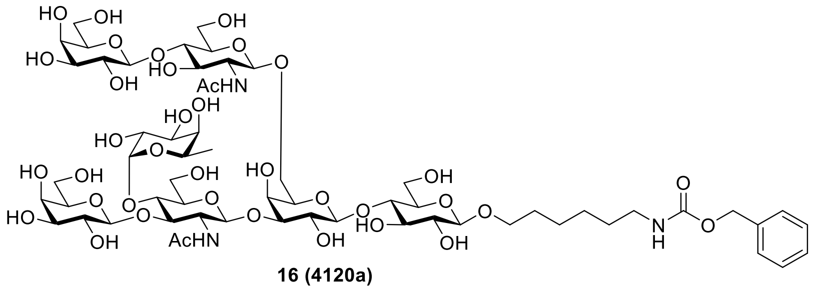

Compound 16 (4120a). Compound **15** (25.6 mg, 19.9 μmol) and UDP-Gal (16.9 mg, 29.9 μmol) were used as the acceptor and the donor, respectively, by following the **general procedure for DUSD-G4a**. After being shaken for 43.5 h, the reaction was quenched and the mixture was purified by following **general purification procedure 1**. After purification, the product was lyophilized to give compound **16** (4120a) in 91% yield (26.3 mg). R_f = 0.25 (EA/ MeOH/ H_2O/AcOH = 7/4/2/1 (v/v/v/v)); ^{1}H NMR (850 MHz, D_2O) δ 7.50-7.38 (m, 5H), 5.11 (br, 2H), 5.04 (d, J = 4.4 Hz, 1H), 4.89 (q, J = 6.7 Hz, 1H), 4.71 (d, J = 8.4 Hz, 1H), 4.64 (d, J = 8.1 Hz, 1H), 4.51 (d, J = 7.9 Hz, 1H), 4.47 (d, J = 7.9 Hz, 1H), 4.46 (d, J = 8.7 Hz, 1H), 4.43 (d, J = 7.9 Hz, 1H), 4.16 (d, J = 3.1 Hz, 1H), 4.09 (t, J = 10.0 Hz, 1H), 4.03-3.98 (m, 2H), 3.98-3.94 (m, 3H), 3.93 (d, J = 3.3 Hz, 1H), 3.92-3.86 (m, 4H), 3.86-3.69 (m, 16H), 3.69-3.53 (m, 11H), 3.49 (dd, J = 9.7, 7.8 Hz, 1H), 3.31 (t, J = 8.6 Hz, 1H), 3.12 (t, J = 6.4 Hz, 2H), 2.06 (s, 3H), 2.04 (s, 3H), 1.66-1.56 (m, 2H), 1.49 (quin, J = 7.0 Hz, 2H), 1.41-1.27 (m, 4H), 1.19 (d, J = 6.7 Hz, 3H); ^{13}C NMR (214 MHz, D_2O) δ 175.70, 175.44, 159.42, 137.64, 129.74 (×2), 129.26, 128.53 (×2), 103.99, 103.85, 103.82, 103.55, 102.96, 101.93, 98.99, 82.80, 79.93, 79.36, 76.88, 76.31, 76.19, 75.78, 75.72, 75.61, 75.44, 74.40, 73.84, 73.47, 73.41, 73.29, 73.07, 72.91, 71.92, 71.49, 71.47, 70.78, 70.10, 69.59, 69.52, 69.50, 69.32, 68.76, 67.80, 67.64, 62.61, 61.98, 60.99 (×2), 60.57, 56.84, 55.97, 41.26, 29.61, 29.56, 26.45, 25.58, 23.39, 23.25, 16.33; HRMS (ESI) m/z calcd for $C_{60}H_{96}N_3O_{37}$ $[M-H]^-$: 1450.5723; found 1450.5735.

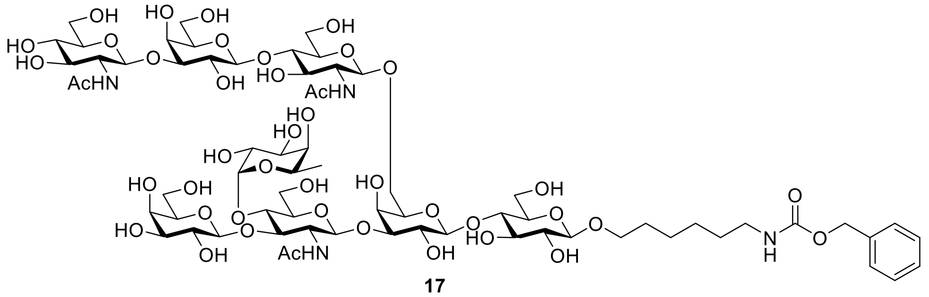

Compound 17. Compound **16** (4120a) (24.8 mg, 17.0 μmol) and UDP-GlcNAc (16.6 mg, 22.5 μmol) were used as the acceptor and the donor, respectively, by following the **general procedure for DUSD-NAc**. After being shaken for 7.5 h, the reaction was quenched, and the mixture was purified by following **general purification procedure 1**. After purification, the

product was lyophilized to give compound **17** in 97% yield (27.5 mg). R_f = 0.17 (EA/MeOH/H_2O/AcOH = 7/4/2/1 (v/v/v/v)); ^{1}H NMR (850 MHz, D_2O) δ 7.46-7.40 (m, 5H), 5.11 (br, 2H), 5.04 (d, *J* = 3.8 Hz, 1H), 4.88 (q, *J* = 6.7 Hz, 1H), 4.71 (d, *J* = 8.5 Hz, 1H), 4.69 (d, J = 8.5 Hz, 1H), 4.63 (d, *J* = 8.2 Hz, 1H), 4.51 (d, *J* = 7.7 Hz, 1H), 4.46 (d, *J* = 7.8 Hz, 1H), 4.42 (d, *J* = 7.8 Hz, 1H), 4.15 (br, 2H), 4.09 (t, *J* = 9.7 Hz, 1H), 4.00-3.95 (m, 5H), 3.91-3.87 (m, 5H), 3.86-3.69 (m, 19), 3.68-3.53 (m, 11H), 3.49 (dd, J = 9.5, 7.9 Hz, 1H), 3.477 (dd, *J* = 9.8, 8.8 Hz, 1H), 3.45 (ddd, J = 9.8, 5.2, 2.2 Hz, 1H), 3.31 (t, *J* = 8.7 Hz, 1H), 3.12 (t, *J* = 6.4 Hz, 1H), 2.06 (s, 3H), 2.045 (s, 3H), 2.04 (s, 3H), 1.64-1.59 (m, 2H), 1.49 (quin, *J* = 7.1 Hz, 2H), 1.38-1.28 (m, 4H), 1.19 (d, *J* = 6.5 Hz, 3H); ^{13}C NMR (214 MHz, D_2O) δ 175.89, 175.70, 175.44, 159.42, 137.64, 129.74 (×2), 129.26, 128.53 (×2), 103.99, 103.98, 103.88, 103.81, 103.55, 102.97, 101.93, 98.97, 82.92, 82.79, 79.91, 79.38, 76.87, 76.62, 76.19, 75.85, 75.78, 75.71, 75.61, 75.44, 74.53, 74.38, 73.84, 73.40, 73.29, 73.07, 72.91, 71.50, 71.47, 70.96, 70.78, 70.65, 70.10, 69.55, 69.31 (×3), 68.76, 67.80, 67.64, 62.61, 61.91, 61.44, 61.01, 60.99, 60.57, 56.84, 56.62, 55.92, 41.26, 29.61, 29.56, 26.44, 25.58, 23.38, 23.25, 23.12, 16.33; HRMS (ESI) *m/z* calcd for $C_{68}H_{109}N_4O_{42}$ $[M-H]^-$: 1653.6516; found 1653.6507.

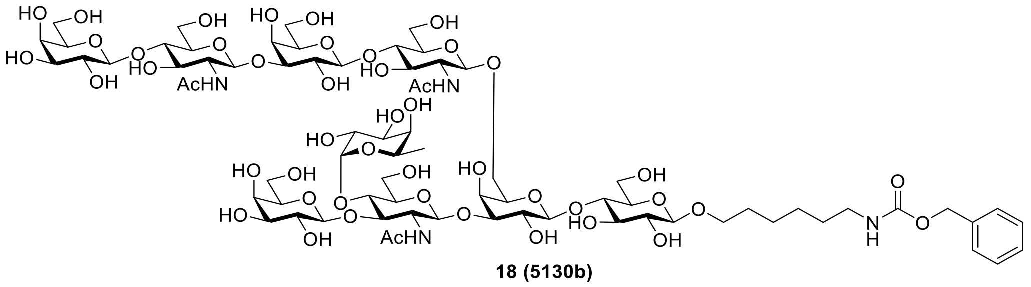

18 (5130b)

Compound 18 (5130b). Compound **17** (24.8 mg, 15.0 µmol) and UDP-Gal (12.7 mg, 22.5 µmol) were used as the acceptor and the donor, respectively, by following the **general procedure for DUSD-G4a**. After being shaken for 7 h, the reaction was quenched and purified by following **general purification procedure 1**. After purification, the product was lyophilized to give compound **18** (5130b) in 91% yield (24.7 mg); R_f = 0.26 (*n*-PrOH/H_2O/AcOH = 6/2/1 (v/v/v)). ^{1}H NMR (850 MHz, D_2O) δ 7.48-7.38 (m, 5H), 5.11 (br, 2H), 5.04 (d, *J* = 3.9 Hz, 1H), 4.89 (q, *J* = 6.5 Hz, 1H), 4.71 (d, *J* = 8.3 Hz, 1H), 4.70 (d, *J* = 8.3 Hz, 1H), 4.64 (d, *J* = 8.2 Hz, 1H), 4.51 (d, *J* = 7.7 Hz, 1H), 4.49 (d, *J* = 7.9 Hz, 1H), 4.46 (d, *J* = 7.8 Hz, 2H), 4.43 (d, *J* = 8.0 Hz, 1H), 4.16 (d, *J* = 3.2 Hz, 1H), 4.15 (d, *J* = 3.1 Hz, 1H), 4.09 (t, *J* = 9.8 Hz, 1H), 4.00-3.93 (m, 7H), 3.91-3.70 (m, 28H), 3.67 (dd, *J* = 10.0, 3.2 Hz, 1H), 3.66-3.54 (m, 12H), 3.49 (t, *J* = 8.1 Hz, 1H), 3.31 (t, *J* = 8.6 Hz, 1H), 3.12 (t, *J* = 6.4 Hz, 2H), 2.06 (s, 3H), 2.04 (s, 6H), 1.65-1.57 (m, 2H), 1.49 (quin, *J* = 7.0 Hz, 2H), 1.41-1.28 (m, 4H), 1.19 (d, *J* = 6.5 Hz, 3H); ^{13}C NMR (214 MHz, D_2O) δ 175.83, 175.70, 175.44, 159.42, 137.64, 129.74 (×2), 129.26, 128.52 (×2), 103.98, 103.88, 103.82 (x2), 103.70, 103.54, 102.96, 101.92, 98.97, 82.99, 82.79, 79.91, 79.37, 79.13, 76.87, 76.30, 76.18,

75.83, 75.79, 75.70, 75.60, 75.51, 75.43, 74.38, 73.83, 73.47, 73.39, 73.28, 73.14, 73.06, 72.91, 71.92, 71.49, 71.46, 70.91., 70.77, 70.10, 69.56, 69.51, 69.30 (×3), 68.75, 67.80, 67.64, 62.61, 61.98, 61.90, 60.98 (×2), 60.82, 60.57, 56.84, 56.14, 55.92, 41.26, 29.61, 29.56, 26.44, 25.58, 23.38, 23.24, 23.14, 16.32; HRMS (ESI) *m/z* calcd for $C_{74}H_{119}N_4O_{47}$ $[M-H]^-$: 1815.7047; found 1815.7039.

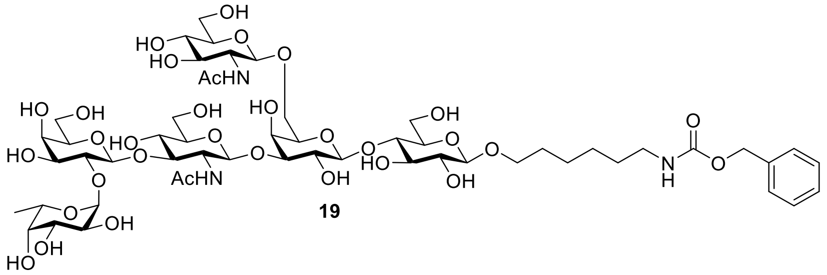

19

Compound 19. The CC1 procedure was applied on compound **14** (47.7 mg, 37.5 µmol) to give compound **19** in 82% yield (39.5 mg). R_f = 0.37 (EA/MeOH/H_2O/AcOH = 7/4/2/1 (v/v/v/v)); ^{1}H NMR (850 MHz, D_2O) δ 7.46-7.40 (m, 5H), 5.20 (d, *J* = 4.1 Hz, 1H), 5.11 (br, 2H), 4.66 (d, *J* = 7.7 Hz, 1H), 4.64 (d, *J* = 8.4 Hz, 1H), 4.62 (d, *J* = 8.7 Hz, 1H), 4.47 (d, *J* = 7.8 Hz, 1H), 4.42 (d, *J* = 7.9 Hz, 1H), 4.30 (q, *J* = 6.6 Hz, 1H), 4.14 (d, *J* = 3.2 Hz, 1H), 4.01 (q, *J* = 7.7 Hz, 1H), 4.00 (m, 1H), 3.96-3.89 (m, 5H), 3.85-3.74 (m, 11H), 3.72-3.62 (m, 6H), 3.61-3.53 (m, 6H), 3.51 (ddd, J = 9.9, 4.8, 2.4 Hz, 1H), 3.47 (dd, *J* = 5.4, 1.8 Hz, 1H), 3.46 (dd, *J* = 9.9, 8.5 Hz, 1H), 3.31 (t, *J* = 8.7 Hz, 1H), 3.12 (t, *J* = 6.3 Hz, 1H), 2.06 (s, 6H), 1.62-1.60 (m, 2H), 1.49 (quin, *J* = 7.1 Hz, 2H), 1.40-1.30 (m, 4H), 1.25 (d, *J* = 6.6 Hz, 3H); ^{13}C NMR (214 MHz, D_2O) δ 175.50, 175.16, 159.41, 137.63, 129.73 (×2), 129.26, 128.52 (×2), 104.16, 104.00, 102.95, 102.01, 101.20, 100.48, 82.29, 79.84, 78.12, 77.61, 76.82, 76.23, 76.03, 75.63, 75.42, 74.83, 74.45, 74.40, 73.85, 72.81, 71.49, 71.01, 70.83, 70.39, 70.10, 69.64 (×2), 69.41, 69.01, 67.64, 67.46, 62.10, 61.65, 61.37, 61.01, 56.46, 55.92, 41.26, 29.61, 29.56, 26.45, 25.58, 23.37, 23.10, 16.22; HRMS (ESI) *m/z* calcd for $C_{54}H_{86}N_3O_{32}$ $[M-H]^-$: 1288.5194; found 1288.5198.

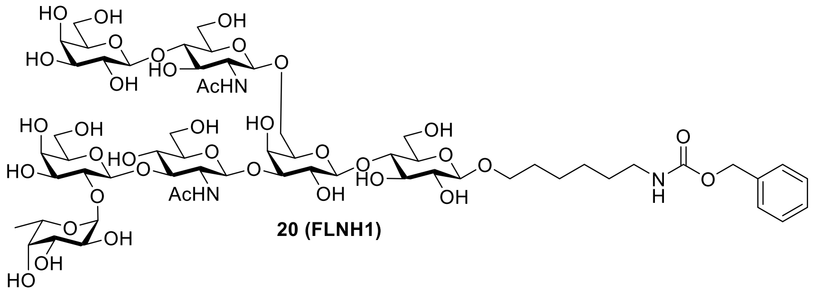

20 (FLNH1)

Compound 20 (FLNH1). Compound **19** (31.0 mg, 24.0 µmol) and Gal (8.6 mg, 47.8 µmol) were used as the acceptor and the donor, respectively, by following the **general procedure for SNRS-G4a**. After being shaken for 2 h, the reaction was quenched, and the mixture was

purified by following **general purification procedure 1**. After purification, the product was lyophilized to give compound **20** (FLNH1) in 95% yield (33.1 mg). R_f = 0.32 (EA/ MeOH/ H_2O/AcOH = 7/4/2/1 (v/v/v/v)); ^{1}H NMR (850 MHz, D_2O) δ 7.46-7.41 (m, 5H), 5.20 (d, J = 4.0 Hz, 1H), 5.11 (br, 2H), 4.66 (d, J = 7.7 Hz, 1H), 4.64 (d, J = 7.9 Hz, 1H), 4.63 (d, J = 8.0 Hz, 1H), 4.47 (d, J = 7.9 Hz, 1H), 4.46 (d, J = 8.7 Hz, 1H), 4.41 (d, J = 7.9 Hz, 1H), 4.30 (q, J = 6.6 Hz, 1H), 4.14 (d, J = 3.2 Hz, 1H), 4.01-3.99 (m, 3H), 3.97-3.95 (m, 2H), 3.93-3.89 (m, 3H), 3.86-3.83 (m, 5H), 3.82-3.77 (m, 5H), 3.76-3.74 (m, 4H), 3.73-3.71 (m, 4H), 3.69-3.65 (m, 4H), 3.64 (t, J = 8.7 Hz, 1H), 3.63-3.60 (m, 2H), 3.59-3.55 (m, 4H), 3.54 (t, J = 9.5 Hz, 1H), 3.50 (ddd, J = 9.9, 4.6, 2.3 Hz, 1H), 3.31 (t, J = 8.6 Hz, 1H), 3.12 (t, J = 6.4 Hz, 2H), 2.07 (s, 3H), 2.06 (s, 3H), 1.63-1.60 (m, 2H), 1.49 (quin, J = 7.1 Hz, 2H), 1.38-1.30 (m, 4H), 1.25 (d, J = 6.6 Hz, 3H); ^{13}C NMR (214 MHz, D_2O) δ 175.44, 175.16, 159.41, 137.63, 129.73 (×2), 129.26, 128.52 (×2), 104.16, 104.00, 103.85, 102.95, 101.93, 101.20, 100.47, 82.28, 79.82, 79.36, 78.12, 77.61, 76.30, 76.23, 76.03, 75.71, 75.62, 75.42, 74.45, 74.39, 73.84, 73.47, 73.40, 72.81, 71.91, 71.48, 71.01, 70.39, 70.10, 69.63, 69.51 (×2), 69.42 (×2), 69.01, 67.64, 67.44, 62.10, 61.97, 61.37, 60.99 (×2), 65.97, 55.97, 55.93, 41.26, 29.61, 29.56, 26.45, 25.58, 23.38, 23.10, 16.22; HRMS (ESI) m/z calcd for $C_{60}H_{96}N_3O_{37}$ $[M-H]^-$: 1450.5723; found 1450.5724.

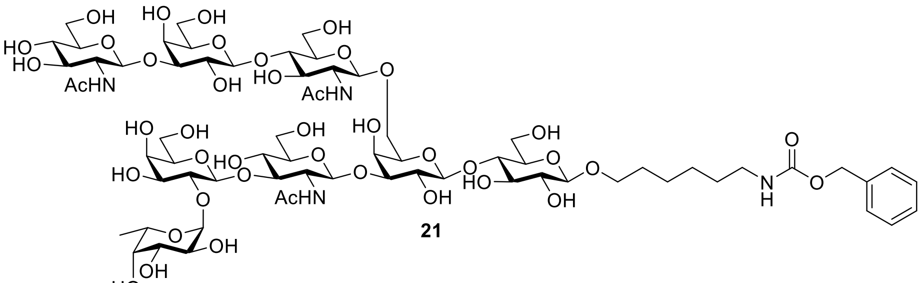

21

Compound 21. Compound **20** (FLNH1) (13.3 mg, 9.2 μmol) and GlcNAc (3.0 mg, 13.7 μmol) were used as acceptor and donor, respectively, by following the general procedure for SNRS-NAc. After being shaken for 6 h, the reaction was quenched and purified by following **general purification procedure 1**. After purification, the product was lyophilized to give compound **21** in 95% yield (14.4 mg); R_f = 0.25 (n-PrOH/ H_2O/AcOH = 6/2/1 (v/v/v)); ^{1}H NMR (850 MHz, D_2O) δ 7.48-7.40 (m, 5H), 5.21 (d, J = 4.1 Hz, 1H), 5.13 (br, 2H), 4.70 (d, J = 8.5 Hz, 1H), 4.66 (d, J = 7.7 Hz, 1H), 4.65 (d, J = 8.0 Hz, 1H), 4.64 (d, J = 8.3 Hz, 1H), 4.48 (d, J = 7.7 Hz, 1H), 4.47 (d, J = 7.9 Hz, 1H), 4.42 (d, J = 7.9 Hz, 1H), 4.31 (q, J = 6.7 Hz, 1H), 4.165 (d, J = 3.2 Hz, 1H), 4.144 (d, J = 3.2 Hz, 1H), 4.04-3.99 (m, 3H), 3.97 (dd, J = 12.3, 1.3 Hz, 1H), 3.94-3.90 (m, 4H), 3.87-3.83 (m, 5H), 3.83-3.78 (m, 6H), 3.78-3.712 (m, 11H), 3.71-3.67 (m, 3H), 3.66-3.56 (m, 9H), 3.547 (dd, J = 9.9, 8.8 Hz, 1H), 3.51 (ddd, J = 9.9, 4.6, 2.2 Hz, 1H), 3.489 (dd, J = 9.8, 8.7 Hz, 1H), 3.46 (ddd, J = 9.9, 5.3, 2.2 Hz, 1H), 3.321 (t, J = 8.7 Hz, 1H), 3.14 (t, J = 6.5 Hz, 2H), 2.07 (s, 3H), 2.068 (s, 3H), 2.055 (s, 3H),

1.64-1.60 (m, 2H), 1.50 (quin, *J* = 7.1 Hz, 2H), 1.39-1.30 (m, 4H), 1.25 (d, *J* = 6.6 Hz, 3H); ^{13}C NMR (MHz, D_2O) *δ* 175.89, 175.45, 175.17, 159.43, 137.65, 129.74 (×2), 129.26, 128.52 (×2), 104.16, 104.00, 103.88, 103.81, 102.96, 101.94, 101.21, 100.48, 82.92, 82.28, 79.81, 79.40, 78.14, 77.62, 76.63, 76.24, 76.04, 75.85, 75.71, 75.63, 75.42, 74.54, 74.46, 74.38, 73.85, 73.40, 72.82, 71.49, 71.02, 70.96, 70.66, 70.40, 70.10, 69.65, 69.63, 69.43, 69.30, 69.02, 67.65, 67.45, 62.10, 61.90, 61.45, 61.38, 61.01, 61.00, 56.62, 55.93 (×2),, 41.27, 29.60, 29.56, 26.44, 25.58, 23.38, 23.12, 23.11, 16.22; HRMS (ESI) *m/z* calcd for $C_{68}H_{109}N_4O_{42}$ [M-H]$^-$: 1653.6516; found 1653.6550.

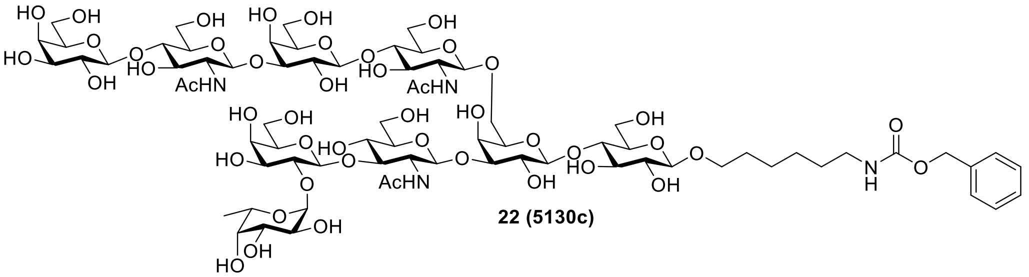

22 (5130c)

Compound 22 (5130c). Compound **21** (14.4 mg, 9.9 µmol) and Gal (2.7 mg, 14.8 µmol) were used as the acceptor and the donor, respectively, by following the **general procedure for SNRS-G4a**. After being shaken for 2 h, the reaction was quenched and the mixture was purified by following **general purification procedure 1**. After purification, the product was lyophilized to give compound **22** (5130c) in 95% yield (15.0 mg). R_f = 0.16 (*n*-PrOH/H_2O/AcOH = 6/2/1 (v/v/v)); ^{1}H NMR (850 MHz, D_2O) *δ* 7.46-7.40 (m, 5H), 5.20 (d, *J* = 4.0 Hz, 1H), 5.11 (br, 2H), 4.71 (d, *J* = 8.4 Hz, 1H), 4.65 (d, *J* = 7.7 Hz, 1H), 4.63 (d, *J* = 8.0 Hz, 1H), 4.63 (d, *J* = 8.3 Hz, 1H), 4.48 (d, *J* = 7.8 Hz, 1H), 4.461 (d, *J* = 8.3 Hz, 1H), 4.457 (d, *J* = 7.8 Hz, 1H), 4.41 (d, *J* = 7.9 Hz, 1H), 4.30 (q, *J* = 6.6 Hz, 1H), 4.16 (d, *J* = 3.1 Hz, 1H), 4.13 (d, *J* = 3.0 Hz, 1H), 4.01-3.98 (m, 3H), 3.97-3.95 (m, 2H), 3.93 (d, *J* = 3.3 Hz, 1H), 3.92-3.89 (m, 3H), 3.86-3.82 (m, 6H), 3.81-3.79 (m, 3H), 3.79-3.77 (m, 3H), 3.76-3.70 (m, 13H), 3.69-3.65 (m, 4H), 3.65-3.56 (m, 7H), 3.63 (t, *J* = 8.8 Hz, 1H), 3.549 (t, *J* = 10.4 Hz, 1H), 3.546 (t, *J* = 10.1 Hz, 1H), 3.53 (t, *J* = 9.2 Hz, 1H), 3.50 (ddd, *J* = 9.9, 4.5, 2.2 Hz, 1H), 3.31 (t, *J* = 8.7 Hz, 1H), 3.12 (t, *J* = 6.3 Hz, 2H), 2.062 (s, 3H), 2.055 (s, 3H), 2.04 (s, 3H), 1.63-1.60 (m, 2H), 1.49 (quin, *J* = 7.1 Hz, 2H), 1.38-1.30 (m, 4H), 1.24 (d, *J* = 6.6 Hz, 3H); ^{13}C NMR (214 MHz, D_2O) *δ* 175.83, 175.44, 175.16, 159.42, 137.64, 129.73 (×2), 129.26, 128.52 (×2), 104.15, 103.99, 103.88, 103.83, 103.70, 102.96, 101.93, 101.20, 100.47, 82.99, 82.27, 79.80, 79.38, 79.14, 78.12, 77.61, 76.31, 76.23, 76.03, 75.83, 75.70, 75.62, 75.51, 75.41, 74.45, 74.38, 73.84, 73.47, 73.39, 73.14, 72.81, 71.93, 71.48, 71.02, 70.92, 70.39, 70.09, 69.65, 69.62, 69.51, 69.42, 69.28, 69.01, 67.64, 67.45, 62.10, 61.98, 61.90, 61.37, 61.00, 60.98, 60.82, 56.15, 55.93 (×2), 41.26, 29.61, 29.56, 26.45, 25.58, 23.38, 23.13, 23.10, 16.22; HRMS (ESI) *m/z* calcd for $C_{74}H_{119}N_4O_{47}$ [M-H]$^-$: 1815.7045; found 1815.7040.

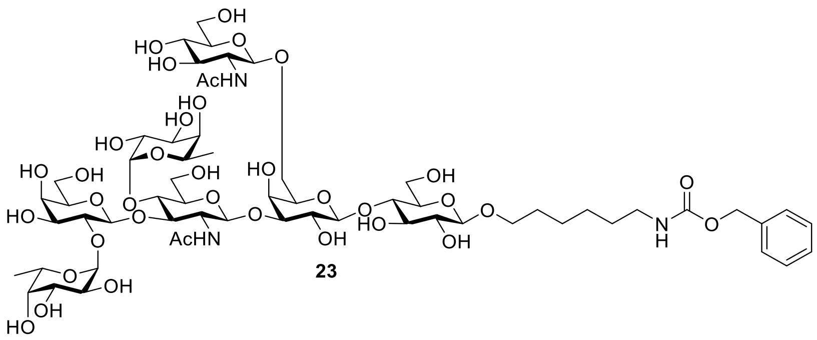

Compound 23. Compound **19** (42.7 mg, 33.1 µmol) and GDP-Fuc (25.2 mg, 39.7 µmol) were used as the acceptor and the donor, respectively, by following the **general procedure for DUSD-F3/4**. After being shaken for 18 h, the reaction was quenched and the mixture was purified by following **general purification procedure 1**. After purification, the product was lyophilized to give compound **23** in 96% yield (45.7 mg); R_f = 0.33 (EA/ MeOH/ H_2O/AcOH = 7/4/2/1 (v/v/v/v)); ^{1}H NMR (850 MHz, D_2O) δ 7.48-7.40 (m, 5H), 5.167 (d, *J* = 4.0 Hz, 1H), 5.12 (br, 2H), 5.044 (d, *J* = 3.8 Hz, 1H), 4.88 (q, *J* = 6.8 Hz, 1H), 4.67 (d, *J* = 7.7 Hz, 1H), 4.625 (d, *J* = 8.5 Hz, 1H), 4.614 (d, *J* = 8.5 Hz, 1H), 4.47 (d, *J* = 8.0 Hz, 1H), 4.42 (d, *J* = 7.9 Hz, 1H), 4.36 (q, *J* = 6.7 Hz, 1H), 4.144 (t, *J* = 9.9 Hz, 1H), 4.140 (d, *J* = 3.5 Hz, 1H), 4.01 (q, *J* = 8.6 Hz, 1H), 3.98-3.92 (m, 4H), 392-3.86 (m, 4H), 3.85-3.78 (m, 6H), 3.78-3.73 (m, 6H), 3.72-3.695 (m, 3H), 3.68-3.66 (m, 1H), 3.643 (t, *J* = 8.7 Hz, 1H), 3.622 (dd, *J* = 9.6, 7.8 Hz, 1H), 3.60-3.552 (m, 5H), 3.54 (dt, J = 9.6, 2.8 Hz, 1H), 3.48 (ddd, J = 9.9, 5.7, 2.2 Hz, 1H), 3.46 (dd, *J* = 9.7, 8.5 Hz, 1H), 3.314 (dd, J = 9.1, 8.2 Hz, 1H), 3.13 (t, *J* = 6.5 Hz, 2H), 2.075 (s, 3H), 2.07 (s, 3H), 1.65-1.60 (m, 2H), 1.50 (quin, *J* = 7.1 Hz, 2H), 1.39-1.31 (m, 4H), 1.29 (d, *J* = 6.7 Hz, 3H), 1.27 (d, *J* = 6.6 Hz, 3H); ^{13}C NMR (214 MHz, D_2O) δ 175.50, 175.10, 159.43, 137.64, 129.73 (×2), 129.26, 128.51 (×2), 104.17, 104.01, 102.94, 102.02, 101.59, 100.52, 98.74, 82.30, 79.83, 77.44, 76.82, 76.15, 75.72, 75.63, 75.44, 75.41, 74.84, 74.60, 74.39, 73.85, 72.95, 72.93, 72.73, 71.49, 70.96, 70.83, 70.40, 70.07, 69.70, 69.64 (×2), 69.23, 68.77, 67.97, 67.65, 67.20, 62.55, 61.65, 61.00, 60.43, 56.69, 56.45, 41.26, 29.60, 29.55, 26.43, 25.57, 23.37, 23.13, 16.32, 16.27; HRMS (ESI) *m/z* calcd for $C_{60}H_{96}N_3O_{36}$ $[M-H]^-$: 1434.5774; found 1434.5767

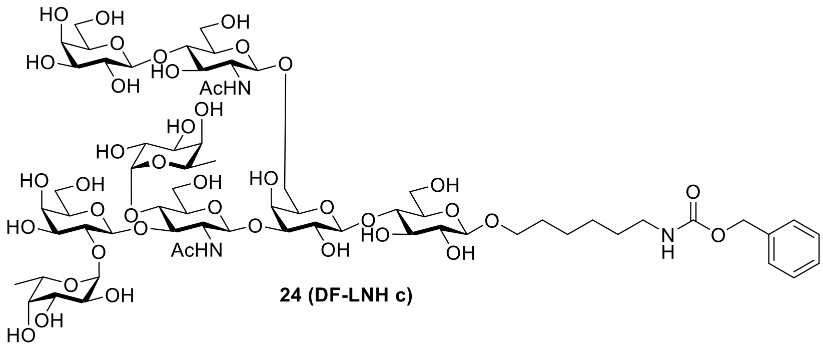

Compound 24 (DF-LNH c).

β1,4-galactosylation catalyzed by NmLgtB.

Compound **23** (36.5 mg, 25.4 μmol) and UDP-Gal (28.8 mg, 50.1 μmol) were used as the acceptor and the donor, respectively, by following the **general procedure for DUSD-G4a**. After being shaken for 47 h, the reaction was quenched, and the mixture was purified by following **general purification procedure 1**. After purification, the product was lyophilized to give compound **24** (DF-LNH c) in 47% yield (Brsm : based on recovered starting material, 76%, 19.2 mg). R_f = 0.28 (*n*-PrOH/ H_2O/AcOH = 6/2/1 (v/v/v));

β1,4-galactosylation catalyzed by HP0826.

Compound **23** (13.7 mg, 9.5 μmol) and Gal (2.1 mg, 11.5 μmol) were used as the acceptor and the donor precursor, respectively, by following the **general procedure for SNRS-G4b**. After being shaken for 30 min, the reaction was quenched and the mixture was purified by following **general purification procedure 1**. After purification, the product was lyophilized to give compound **24** (DF-LNH c) in 93% yield (15.6 mg). R_f = 0.14 (EA/MeOH/ H_2O = 4/2/1 (v/v/v)); ^{1}H NMR (850 MHz, D_2O) δ 7.47-7.40 (m, 5H), 5.16 (d, *J* = 4.0 Hz, 1H), 5.12 (br, 2H), 5.04 (d, *J* = 3.8 Hz, 1H), 4.88 (q, *J* = 6.7 Hz, 1H), 4.67 (d, *J* = 7.7 Hz, 1H), 4.64 (d, *J* = 8.1 Hz, 1H), 4.61 (d, *J* = 8.4 Hz, 1H), 4.48 (d, *J* = 7.8 Hz, 1H), 4.47 (d, *J* = 7.7 Hz, 1H), 4.41 (d, *J* = 7.9 Hz, 1H), 4.35 (q, *J* = 6.7 Hz, 1H), 4.14 (t, *J* = 9.8 Hz, 1H), 4.136 (d, J = 3.4 Hz, 1H), 4.01 (dd, *J* = 7.0, 2.5 Hz, 1H), 4.00 (dd, *J* = 9.7, 1.6 Hz, 1H) , 3.97-3.89 (m, 5H), 3.88-3.82 (m, 7H), 3.81-3.72 (m, 12H), 3.70 (dd, *J* = 10.2, 3.2 Hz, 2H), 3.69-3.60 (m, 4H), 3.67 (dd, *J* = 9.9, 3.4 Hz, 1H), 3.59-3.53 (m, 3H), 3.55 (dd, *J* = 9.4, 8.2 Hz, 2H), 3.53 (dt, *J* = 9.7, 2.4 Hz, 1H), 3.31 (t, *J* = 8.5 Hz, 1H), 3.13 (t, *J* = 6.4 Hz, 2H), 2.07 (s, 3H), 2.06 (s, 3H), 1.64-1.60 (m, 2H), 1.50 (quin, *J* = 7.1 Hz, 2H), 1.39-1.31 (m, 4H), 1.28 (d, *J* = 6.7 Hz, 3H), 1.27 (d, *J* = 6.7 Hz, 3H); ^{13}C NMR (214 MHz, D_2O) δ 175.49, 175.11, 159.45, 137.66, 129.74 (×2), 129.26, 128.52 (×2), 104.18, 104.02, 103.86, 102.96, 101.95, 101.60, 100.53, 98.75, 82.30, 79.82 , 79.37, 77.45, 76.32, 76.16, 75.73 (×2), 75.64, 75.45, 75.42, 74.60, 74.38, 73.85, 73.47, 73.42, 72.96, 72.94, 72.73, 71.92, 71.50, 70.97, 70.41, 70.07, 69.71, 69.67, 69.65, 69.52, 69.24, 68.78, 67.98, 67.65, 67.21, 62.56, 61.98 (×2), 61.00, 60.43, 56.70, 55.97, 41.26, 29.61, 29.56, 26.44, 25.58, 23.39, 23.14, 16.32, 16.28; HRMS (ESI) *m/z* calcd for $C_{66}H_{106}N_3O_{41}$ $[M-H]^-$: 1596.6302; found 1596.6303.

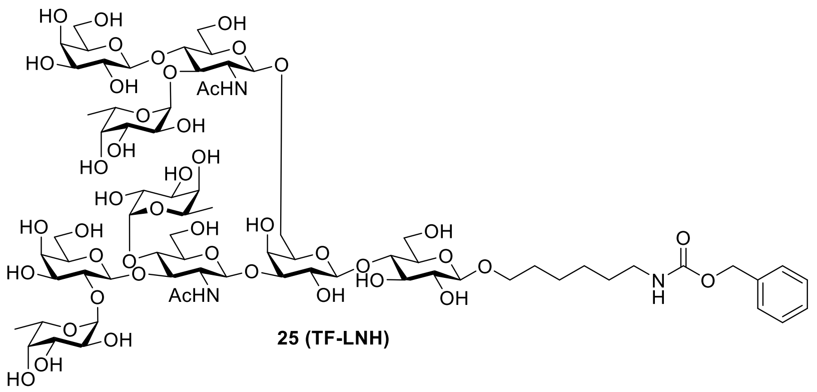

25 (TF-LNH)

Compound 25 (TF-LNH). Compound **20** (FLNH1) (14.7 mg, 10.1 μmol) and GDP-Fuc (14.1 mg, 22.3 μmol) were used as the acceptor and the donor precursor, respectively, by following the **general procedure for DUSD-F3/4**. After being shaken for 21 h, the reaction was quenched and the mixture was purified by following **general purification procedure 1**. After purification, the product was lyophilized to give compound **25** (TF-LNH) in 80% yield (14.1 mg). R_f = 0.23 (*n*-PrOH/ H_2O/AcOH = 6/2/1 (v/v/v)); ^{1}H NMR (850 MHz, D_2O) δ 7.46-7.40 (m, 5H), 5.16 (d, *J* = 4.0 Hz, 1H), 5.11 (br, 2H), 5.10 (d, *J* = 4.3 Hz, 1H), 5.03 (d, *J* = 3.7 Hz, 1H), 4.87 (q, *J* = 6.6 Hz, 1H), 4.83 (q, *J* = 6.6 Hz, 1H), 4.66 (d, *J* = 7.7 Hz, 1H), 4.64 (d, *J* = 7.6 Hz, 1H), 4.60 (d, *J* = 8.4 Hz, 1H), 4.46 (d, *J* = 8.3 Hz, 1H), 4.45(d, *J* = 7.7 Hz, 1H), 4.40 (d, *J* = 7.9 Hz, 1H), 4.35 (q, *J* = 6.6 Hz, 1H), 4.13 (t, *J* = 9.4 Hz, 1H), 4.12 (d, *J* = 2.7 Hz, 1H), 4.03-3.98 (m, 2H), 3.96-3.88 (m, 9H), 3.87-3.84 (m, 4H), 3.83-3.78 (m, 7H), 3.77-3.69 (m, 10H), 3.67-3.56 (m, 7H), 3.65 (dd, *J* = 9.7, 3.3 Hz, 1H), 3.63 (t, *J* = 10.4 Hz, 1H), 3.55 (dd, *J* = 9.9, 8.0 Hz, 1H), 3.53 (dt, *J* = 9.6, 2.7 Hz, 1H), 3.50 (dd, *J* = 9.7, 8.0 Hz, 1H), 3.30 (t, *J* = 8.7 Hz, 1H), 3.12 (t, *J* = 6.3 Hz, 2H), 2.07 (s, 3H), 2.05 (s, 3H), 1.63-1.59 (m, 2H), 1.49 (quin, *J* = 7.1 Hz, 2H), 1.38-1.30 (m, 4H), 1.28 (d, *J* = 6.6 Hz, 3H), 1.26 (d, *J* = 6.6 Hz, 3H), 1.18 (d, *J* = 6.6 Hz, 3H); ^{13}C NMR (214 MHz, D_2O) δ 175.22, 175.10, 159.42, 137.64, 129.73 (×2), 129.26, 128.52 (×2), 104.16, 104.09, 102.94, 102.82, 101.76, 101.59, 100.52, 99.60, 98.75, 82.28, 79.95, 77.44, 76.34, 76.15, 75.87, 75.81, 75.72, 75.63, 75.44 (×2), 74.60, 74.32, 74.31, 73.84, 73.42, 72.96, 72.93, 72.86, 72.73, 71.99, 71.50, 70.96, 70.41, 70.19, 70.07, 69.70, 69.66, 69.61, 69.30, 69.23, 68.77, 68.68, 67.97, 67.69, 67.64, 67.20, 62.56, 62.44, 61.01, 60.73, 60.43, 56.70, 56.61, 41.27, 29.62, 29.56, 26.45, 25.58, 23.47, 23.14, 16.32, 16.27, 16.26; HRMS (ESI) *m/z* calcd for $C_{72}H_{116}N_3O_{45}$ $[M-H]^-$: 1742.6881; found 1742.6879.

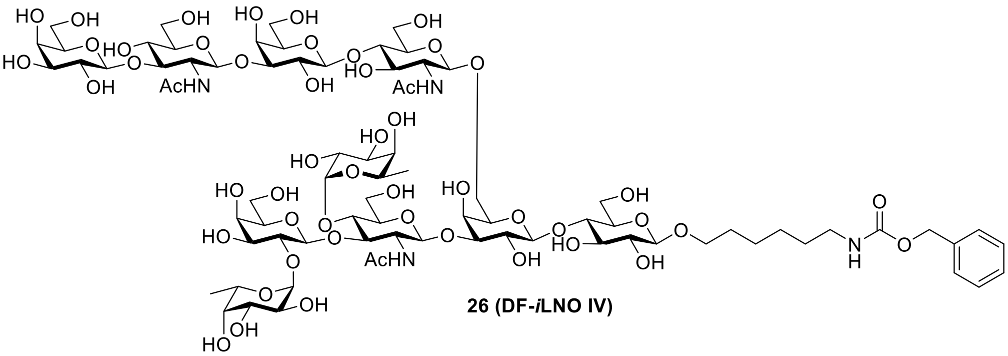

26 (DF-*i*LNO IV)

Compound 26 (DF-*i*LNO IV). Compound **24** (DF-LNH c) (15.6 mg, 8.9 μmol) and GlcNAc (2.4 mg, 10.7 μmol) were used as the acceptor and the donor precursor, respectively, by following the **general procedure for SNRS-NAc**. After being shaken for 15 h, the reaction was heated to 100 ^{o}C and quenched enzyme activity. The resulting product was used in next step without purification; R_f = 0.20 (*n*-PrOH/ H_2O/AcOH = 6/2/1 (v/v/v)). To the above solution and Gal (1.9 mg, 10.7 μmol) were used as the acceptor and the donor precursor,

respectively, by following the **general procedure for SNRS-G3**. After being shaken for 13 h, the reaction was quenched and the mixture was purified by following **general purification procedure 1**. After purification, the product was lyophilized to give compound **26** (DF-*i*LNO IV) in 98% yield for two steps (17.4 mg). R_f = 0.15 (*n*-PrOH/ H_2O/AcOH = 6/2/1 (v/v/v)); ^{1}H NMR (850 MHz, D_2O) δ 7.47-7.40 (m, 5H), 5.16 (d, J = 4.0 Hz, 1H), 5.11 (br, 2H), 5.04 (d, J = 3.8 Hz, 1H), 4.88 (q, J = 6.7 Hz, 1H), 4.74 (d, J = 8.5 Hz, 1H), 4.67 (d, J = 7.7 Hz, 1H), 4.64 (d, J = 8.1 Hz, 1H), 4.61 (d, J = 8.4 Hz, 1H), 4.47 (d, J = 7.9 Hz, 2H), 4.45 (d, J = 7.8 Hz, 1H), 4.41 (d, J = 7.9 Hz, 1H), 4.35 (q, J = 6.6 Hz, 1H), 4.16 (d, J = 3.3 Hz, 1H), 4.14 (t, J = 10.2 Hz, 1H), 4.13 (d, J = 3.3 Hz, 1H), 4.01-3.99 (m, 2H), 3.96-3.70 (m, 36H), 3.68-3.60 (m, 4H), 3.62 (dd, J = 8.6, 7.4 Hz, 2H), 3.59-3.57 (m, 4H), 3.55-3.53 (m, 1H), 3.55 (t, J = 10.0 Hz, 1H), 3.54 (dd, J = 9.8, 7.9 Hz, 1H), 3.49 (ddd, J = 9.8, 5.0, 2.3 Hz, 1H), 3.31 (t, J = 8.7 Hz, 1H), 3.13 (t, J = 6.4 Hz, 2H), 2.07 (s, 3H), 2.06 (s, 3H), 2.04 (s, 3H), 1.63-1.60 (m, 2H), 1.49 (quin, J = 7.1 Hz, 2H), 1.38-1.31 (m, 4H), 1.29 (d, J = 6.7 Hz, 3H), 1.27 (d, J = 6.6 Hz, 3H); ^{13}C NMR (214 MHz, D_2O) δ 175.91, 175.44, 175.10, 159.43, 137.65, 129.74 (×2), 129.26, 128.52 (×2), 104.45, 104.17, 104.01, 103.88, 103.52, 102.96, 101.93, 101.60, 100.53, 98.75, 83.04, 82.95, 82.28, 79.80, 79.41, 77.45, 76.24, 76.15 (×2), 75.85, 75.73, 75.71, 75.63, 75.44, 75.41, 74.60, 74.36, 73.85, 73.44, 73.41, 72.96, 72.93, 72.73, 71.64, 71.49, 70.97, 70.96, 70.41, 70.07, 69.70, 69.64, 69.62, 69.49, 69.41, 69.27, 69.23, 68.77, 67.98, 67.64, 67.20, 62.55, 61.99, 61.90, 61.45, 61.00 (×2), 60.43, 56.70, 55.92, 55.66, 41.26, 29.61, 29.56, 26.44, 25.58, 23.38, 23.19, 23.14, 16.32, 16.27; HRMS (ESI) *m/z* calcd for $C_{80}H_{129}N_4O_{51}$ $[M-H]^-$: 1961.7624.5723; found 1961.7617.

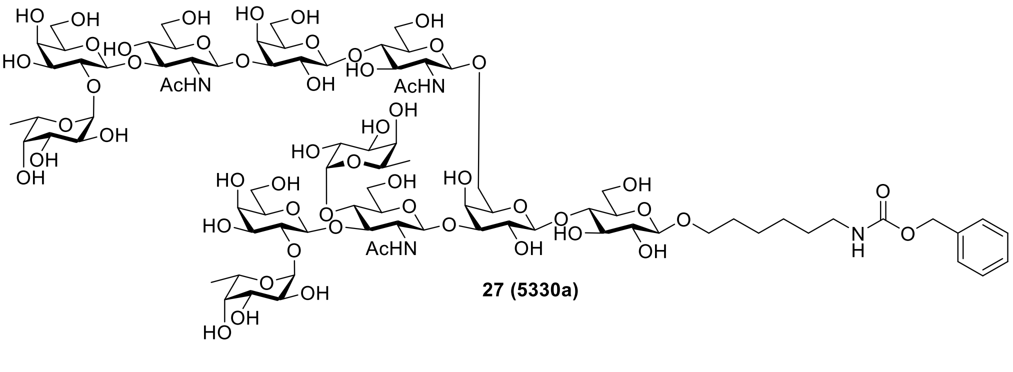

27 (5330a)

Compound 27 (5330a). Compound **26** (DF-*i*LNO IV) (14.3 mg, 7.3 µmol) and GDP-Fuc (6.9 mg, 11.0 µmol) were used as the acceptor and the donor, respectively, by following the **general procedure for DUSD-F2**. After being shaken for 6 h, the reaction was quenched, and the mixture was purified by following **general purification procedure 1**. After purification, the product was lyophilized to give compound **27** (5330a) in 80% yield (12.3 mg). R_f = 0.33 (*n*-PrOH/ H_2O/25% NH_4OH = 5/2/1 (v/v/v)); ^{1}H NMR (850 MHz, D_2O) δ 7.46-7.40 (m, 5H), 5.20 (d, J = 4.0 Hz, 1H), 5.16 (d, J = 3.9 Hz, 1H), 5.11 (br, 2H), 5.03 (d, J = 3.6 Hz, 1H), 4.87 (q, J = 6.7 Hz, 1H), 4.66 (d, J = 8.2 Hz, 1H), 4.65 (d, J = 7.9 Hz, 1H),

4.630 (d, J = 8.0 Hz, 1H), 4.625 (d, J = 8.4 Hz, 1H), 4.61 (d, J = 8.4 Hz, 1H), 4.46 (d, J = 7.8 Hz, 1H), 4.44 (d, J = 7.9 Hz, 1H), 4.41 (d, J = 7.9 Hz, 1H), 4.35 (q, J = 6.7 Hz, 1H), 4.30 (q, J = 6.7 Hz, 1H), 4.139 (d, J = 3.2 Hz, 1H), 4.135 (t, J = 10.1 Hz, 1H), 4.13 (d, J = 3.1 Hz, 1H), 4.00-3.98 (m, 3H), 3.96-3.92 (m, 3H), 3.91-80 (m, 16H), 3.79-3.69 (m, 19H), 3.67 (dd, J = 10.7, 3.1 Hz, 2H), 3.65-3.55 (m, 8H), 3.63 (t, J = 8.5 Hz, 1H), 3.53 (t, J = 9.5 Hz, 2H), 3.49 (ddd, J = 9.8, 4.6, 2.3 Hz, 1H), 3.31 (t, J = 8.7 Hz, 1H), 3.12 (t, J = 6.3 Hz, 2H), 2.07 (s, 3H), 2.06 (s, 3H), 2.05 (s, 3H), 1.63-1.60 (m, 2H), 1.49 (quin, J = 7.1 Hz, 2H), 1.38-1.30 (m, 4H), 1.28 (d, J = 6.7 Hz, 3H), 1.27 (d, J = 6.7 Hz, 3H), 1.24 (d, J = 6.7 Hz, 3H); ^{13}C NMR (214 MHz, D_2O) δ 175.43, 175.18, 175.10, 159.42, 137.64, 129.73 (×2), 129.26, 128.52 (×2), 104.21, 104.17, 104.01, 103.91, 102.96, 101.93, 101.59, 101.21, 100.52, 100.47, 98.74, 82.55, 82.27, 79.81, 79.29, 78.14, 77.62, 77.44, 76.21, 76.14, 76.03, 75.78, 75.72 (×2), 75.62, 75.43, 75.41, 74.60, 74.45, 74.34, 73.84, 73.37, 72.96, 72.93, 72.82, 72.72, 71.49, 71.14, 70.97, 70.40 (×2), 70.09, 70.07, 69.70, 69.63, 69.62, 69.53, 69.43, 69.23, 69.01, 68.77, 67.98, 67.64, 67.45, 67.20, 62.55, 62.10, 61.90, 61.35, 60.99 (×2), 60.43, 56.70, 55.92 (×2), 41.26, 29.61, 29.56, 26.44, 25.58, 23.38, 23.14, 23.10, 16.32, 16.27, 16.22; HRMS (ESI) *m/z* calcd for $C_{86}H_{139}N_4O_{55}$ [M-H]$^-$: 2107.8203; found 2107.8125.

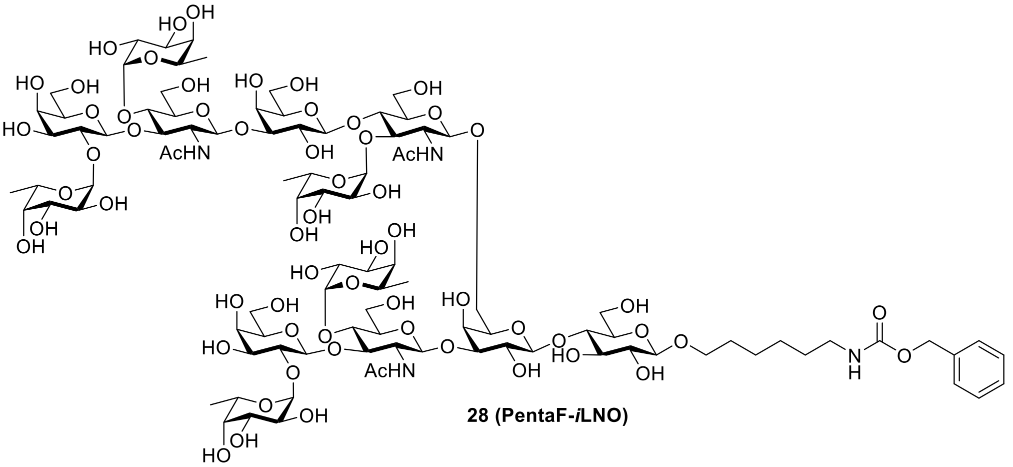

Compound 28 (PentaF-*i*LNO). Compound **27** (5330a) (9.3 mg, 4.4 µmol) and GDP-Fuc (11.7 mg, 18.5 µmol) were used as the acceptor and the donor, respectively, by following the **general procedure for DUSD-F3/4**. After being shaken for 31 h, the reaction was quenched and purified by following **general purification procedure 1**. After purification, the product was lyophilized to give compound **28** (PentaF-*i*LNO) in 95% yield (8.5 mg). R_f = 0.33 (*n*-PrOH/ H_2O/25% NH_4OH = 5/2/1 (v/v/v) stain run two time); ^{1}H NMR (850 MHz, D_2O) δ 7.46-7.40 (m, 5H), 5.16 (d, J = 4.0 Hz, 2H), 5.12 (br, 2H), 5.09 (d, J = 3.9 Hz, 1H), 5.04 (d, J = 3.6 Hz, 2H), 4.88 (q, J = 6.7 Hz, 1H), 4.87 (q, J = 6.7 Hz, 1H), 4.82 (q, J = 6.7 Hz, 1H), 4.671 (d, J = 7.6 Hz, 1H), 4.666 (d, J = 7.6 Hz, 1H), 4.64 (d, J = 7.7 Hz, 1H), 4.61 (d, J = 8.4 Hz, 1H), 4.47 (d, J = 7.8 Hz, 1H), 4.42 (d, J = 7.9 Hz, 1H), 4.41 (d, J = 7.9 Hz, 1H), 4.353 (q, J = 6.7 Hz, 1H), 4.352 (q, J = 6.7 Hz, 1H), 4.14 (t, J = 10.0 Hz, 2H), 4.13 (d, J = 2.7 Hz, 1H),

4.08 (d, J = 3.2 Hz, 1H), 4.01-3.98 (m, 2H), 3.96-3.72 (m, 38H), 3.71-3.68 (m, 6H), 3.67-3.51 (m, 9H), 3.64 (dd, J = 9.1, 8.7 Hz, 1H), 3.62 (dd, J = 9.1, 8.4 Hz, 2H), 3.56 (dd, J = 9.7, 8.1 Hz, 1H), 3.48 (dd, J = 9.2, 8.2 Hz, 1H), 3.30 (t, J = 8.7 Hz, 1H), 3.12 (t, J = 6.3 Hz, 2H), 2.07 (s, 3H), 2.06 (s, 3H), 2.05 (s, 3H), 1.63-1.60 (m, 2H), 1.49 (quin, J = 7.1 Hz, 2H), 1.38-1.31 (m, 4H), 1.28 (d, J = 6.7 Hz, 6H), 1.27 (d, J = 6.7 Hz, 6H), 1.16 (d, J = 6.7 Hz, 3H); ^{13}C NMR (214 MHz, D_2O) δ 175.25, 175.13, 175.11, 159.44, 137.65, 129.74 (×2), 129.26, 128.52 (×2), 104.18, 104.17, 104.10, 102.94, 102.77, 101.76, 101.60, 101.57, 100.53, 100.51, 99.80, 98.75, 98.73, 82.26, 82.17, 79.98, 77.45, 77.42, 76.33, 76.14, 76.08, 75.92, 75.73, 75.71, 75.63, 75.43 (×3), 75.32, 74.59 (×2), 74.27, 73.88, 73.83, 72.99, 72.96 (×2), 72.94, 72.83, 72.72, 72.71, 71.67, 71.50, 70.97, 70.41, 70.37, 70.18, 70.07 (×2), 69.70 (×2), 69.61 (×2), 69.50, 69.24, 69.11, 68.77 (×2), 68.59, 67.98 (×2), 67.67, 67.65, 67.20 (×2), 62.56 (×2), 62.42, 61.01, 60.72, 60.43, 60.41, 56.77, 56.70, 56.63, 41.26, 29.61, 29.56, 26.44, 25.57, 23.48, 23.14 (×2), 16.32 (×2), 16.30 (×2), 16.28; HRMS (ESI) *m/z* calcd for $C_{98}H_{159}N_4O_{63}$ $[M-H]^-$: 2399.9361; found 2399.9452.

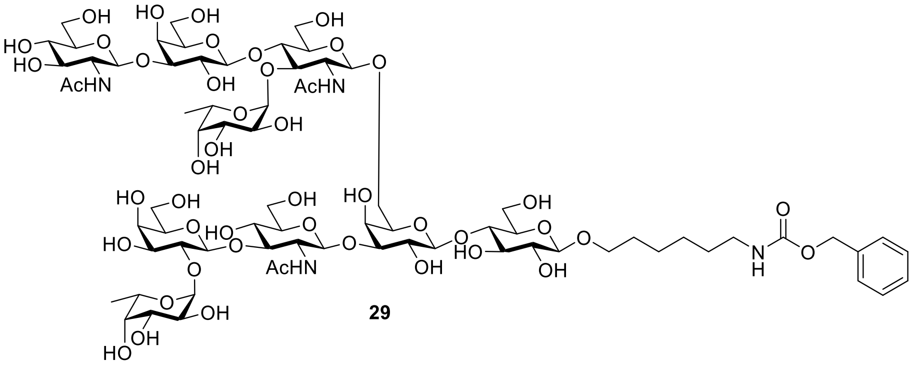

29

Compound 29. Compound **21** (20.8 mg, 12.6 μmol) and GDP-Fuc (8.4 mg, 13.3 μmol) were used as the acceptor and the donor, respectively, by following the **general procedure for DUSD-F3/4**. After being shaken for 6 h, the reaction was quenched and purified by following **general purification procedure 1**. After purification, the product was lyophilized to give compound **29** in 55% yield (brsm = 63%, 12.6 mg). R_f = 0.26 (*n*-PrOH/ H_2O/AcOH = 6/2/1 (v/v/v)); ^{1}H NMR (850 MHz, D_2O) δ 7.49-7.40 (m, 5H), 5.207 (d, J = 4.1 Hz, 1H), 5.13 (br, 2H), 5.103 (d, J = 4.0 Hz, 1H), 4.82 (q, J = 7.1 Hz, 1H, overlapping D_2O), 4.70 (d, J = 8.4 Hz, 1H), 4.66 (d, J = 7.7 Hz, 1H), 4.65 (d, J = 7.8 Hz, 1H), 4.64 (d, J = 8.4 Hz, 1H), 4.476 (d, J = 7.9 Hz, 1H), 4.45 (d, J = 7.9 Hz, 1H), 4.42 (d, J = 7.9 Hz, 1H), 4.31 (q, J = 6.7 Hz, 1H), 4.137 (d, J = 3.3 Hz, 1H), 4.108 (d, J = 3.5 Hz, 1H), 4.02-3.98 (m, 3H), 3.97 (dd, J = 12.4, 1.5 Hz, 1H), 3.94-3.89 (m, 7H), 3.88-3.83 (m, 5H), 3.83-3.78 (m, 6H), 3.78-3.74 (m, 4H), 3.74-3.706 (m, 4H), 3.70 (dd, J = 3.9, 1.3 Hz, 1H), 3.69 (d, J = 3.3 Hz, 1H), 3.677 (d, J = 3.3 Hz, 1H), 3.66-3.55 (m, 9H), 3.55-3.525 (m, 2H), 3.51 (ddd, J = 9.9, 4.7, 2.3 Hz, 1H), 3.49 (dd, J = 9.9, 8.8 Hz, 1H), 3.454 (ddd, J = 9.9, 5.2, 2.3 Hz, 1H), 3.315 (dd, J = 9.1, 8.3 Hz, 1H), 3.14 (t, J = 6.5 Hz, 2H), 2.07 (s, 3H), 2.06 (s, 3H), 2.05 (s, 3H), 1.65-1.60 (m, 2H), 1.49

(quin, *J* = 7.1 Hz, 2H), 1.40-1.30 (m, 4H), 1.25 (d, *J* = 6.6 Hz, 3H), 1.16 (d, *J* = 6.7 Hz, 3H); ^{13}C NMR (214 MHz, D_2O) δ 175.89, 175.25, 175.17, 159.44, 137.65, 129.74 (×2), 129.26, 128.51 (×2), 104.15, 104.07, 103.74, 102.93, 102.77, 101.74, 101.20, 100.47, 99.68, 82.47, 82.27, 79.96, 78.12, 77.61, 76.59, 76.32, 76.23, 76.03, 75.80, 75.63, 75.43, 75.41, 74.47 (×2), 74.46 (×2), 74.30, 74.06, 73.83, 72.81, 71.50, 71.01, 70.66, 70.39, 70.17, 70.09, 69.65, 69.59, 69.42, 69.21, 69.01, 68.62, 67.67, 67.66, 67.45, 62.38, 62.10, 61.43, 61.37, 61.02, 60.73, 56.59 (×2), 55.93, 41.26, 29.60, 29.55, 26.43, 25.57, 23.47, 23.10 (×2), 16.22 (×2); HRMS (ESI) *m/z* calcd for $C_{74}H_{120}N_4Na_1O_{46}$ $[M+Na]^+$: 1823.7071; found 1823.7073

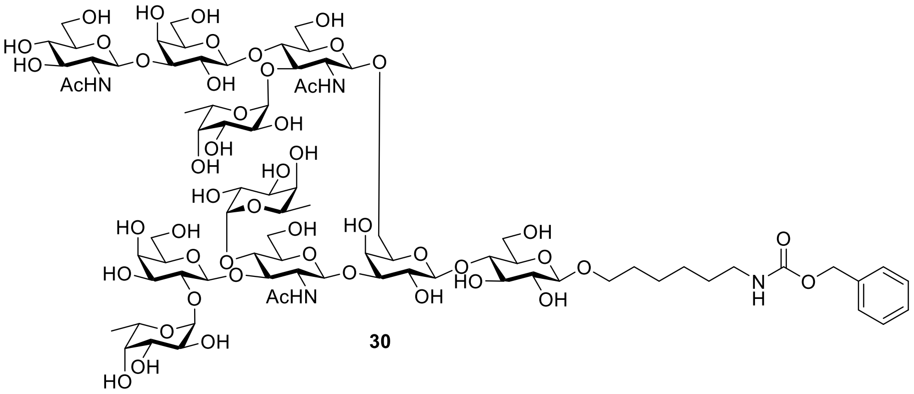

30

Compound 30. Compound **21** (11.4 mg, 6.9 µmol) and GDP-Fuc (9.2 mg, 14.5 µmol) were used as the acceptor and the donor, respectively, by following the **general procedure for DUSD-F3/4**. After being shaken for 26 h, the reaction was quenched and purified by following **general purification procedure 1**. After purification, the product was lyophilized to give compound **30** in 94% yield (12.6 mg). R_f = 0.19 (*n*-PrOH/ H_2O/AcOH = 6/2/1 (v/v/v)); ^{1}H NMR (850 MHz, D_2O) δ 7.50-7.40 (m, 5H), 5.17 (d, *J* = 3.9 Hz, 1H), 5.13 (br, 2H), 5.106 (d, *J* = 3.8 Hz, 1H), 5.05 (d, *J* = 3.6 Hz, 1H), 4.89 (q, *J* = 6.5 Hz, 1H), 4.83 (q, *J* = 6.6 Hz, 1H), 4.70 (d, J = 8.4 Hz, 1H), 4.68(d, J = 7.7 Hz, 1H), 4.65 (d, J = 7.7 Hz, 1H), 4.62 (d, *J* = 8.3 Hz, 1H), 4.48 (d, *J* = 7.7 Hz, 1H), 4.45 (d, *J* = 7.8 Hz, 1H), 4.42 (d, *J* = 7.9 Hz, 1H), 4.36 (q, *J* = 6.6 Hz, 1H), 4.15 (t, *J* = 9.9 Hz, 1H), 4.14 (d, *J* = 2.8 Hz, 1H), 4.11 (d, *J* = 2.7 Hz, 1H), 4.03-3.99 (m, 2H), 3.98-3.93 (m, 4H), 3.93-3.89 (m, 5H), 3.89-3.80 (m, 9H), 3.80-3.70 (m, 15H), 3.70-3.56 (m, 10H), 3.56-3.52 (m, 2H), 3.49 (t, *J* = 9.2 Hz, 1H), 3.46 (ddd, *J* = 9.9, 5.2, 2.0 Hz, 1H), 3.32 (t, *J* = 8.7 Hz, 1H), 3.14 (t, *J* = 6.2 Hz, 2H), 2.08 (s, 3H), 2.06 (s, 3H), 2.05 (s, 3H), 1.65-1.60 (m, 2H), 1.51 (quin, *J* = 7.1 Hz, 2H), 1.40-1.32 (m, 4H), 1.29 (d, *J* = 6.5 Hz, 3H), 1.28 (d, *J* = 6.5 Hz, 3H), 1.17 (d, *J* = 6.5 Hz, 3H); ^{13}C NMR (201 MHz, D_2O) δ 175.86, 175.25, 175.12, 159.46, 137.66, 129.75 (×2), 129.27, 128.53 (×2), 104.18, 104.10, 103.78, 102.95, 102.78, 101.77, 101.61, 100.54, 99.71, 98.76, 82.48, 82.27, 79.96, 77.45, 76.59, 76.33, 76.15, 75.83, 75.74, 75.64, 75.44, 75.43, 74.60, 74.48, 74.29, 74.05, 73.85, 72.97, 72.94, 72.82, 72.73, 71.52, 71.50, 70.98, 70.66, 70.41, 70.17, 70.07, 69.71, 69.64, 69.62, 69.45, 69.24 (×2), 68.78, 68.62, 68.00, 67.69, 67.66, 67.22, 62.57, 62.41, 61.42, 61.01,

60.72, 60.42, 56.71, 56.60 (×2), 41.27, 29.62, 29.57, 26.45, 25.59, 23.48, 23.14, 23.11, 16.33, 16.29, 16.24; HRMS (ESI) *m/z* calcd for $C_{80}H_{130}N_4Na_1O_{50}$ $[M+Na]^+$: 1969.7651; found 1969.7631

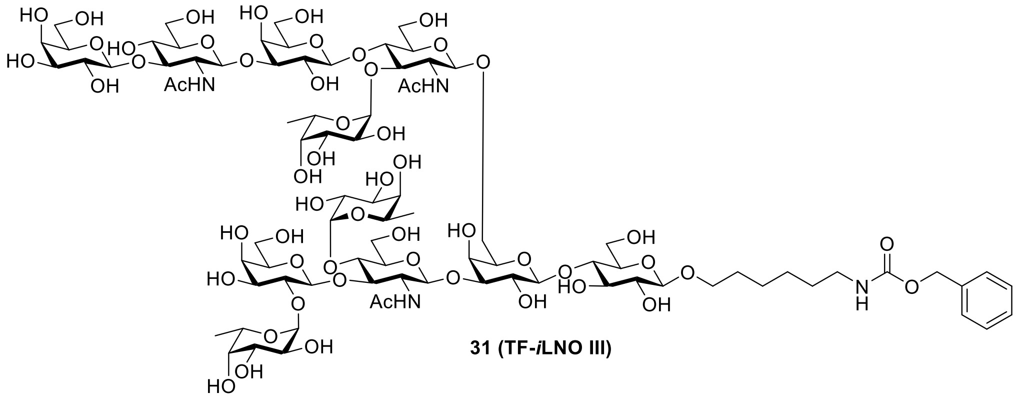

31 (TF-*i*LNO III)

Compound 31 (TF-*i*LNO III). Compound **21** (11.4 mg, 6.9 μmol) and GDP-Fuc (9.6 mg, 15.1 μmol) were used as the acceptor and the donor, respectively, by following the **general procedure for DUSD-F3/4**. After being shaken for 26 h, the reaction was quenched and the mixture was purified by following **general purification procedure 1**. After purification, the product was lyophilized to give compound **30** in 94% yield (12.6 mg). Compound **21** R_f = 0.27; Di-fuc product R_f = 0.23; compound **30** R_f = 0.20 (*n*-PrOH/ H_2O/AcOH = 6/2/1 (v/v/v)). Compound **30** (9.6 mg, 4.9 μmol) and Gal (1.0 mg, 5.4 μmol) were used as the acceptor and the donor precursor, respectively, by following the **general procedure for SNRS-G3**. After being shaken for 31 h, the reaction was quenched and the mixture was purified by following **general purification procedure 1**. After purification, the product was lyophilized to give compound **31** (TF-*i*LNO III) in 90% yield (9.3 mg). R_f = 0.20 (*n*-PrOH/ H_2O/AcOH = 5/2/1 (v/v/v), developed for twice); ^1H NMR (850 MHz, D_2O) δ 7.47-7.40 (m, 5H), 5.16 (d, *J* = 4.0 Hz, 1H), 5.12 (br, 2H), 5.10 (d, *J* = 3.9 Hz, 1H), 5.04 (d, *J* = 3.7 Hz, 1H), 4.88 (q, *J* = 6.7 Hz, 1H), 4.82 (q, *J* = 6.7 Hz, 1H), 4.73 (d, *J* = 8.5 Hz, 1H), 4.67 (d, *J* = 7.7 Hz, 1H), 4.64 (d, *J* = 7.7 Hz, 1H), 4.61 (d, *J* = 8.4 Hz, 1H), 4.47 (d, *J* = 8.2 Hz, 1H), 4.46 (d, *J* = 8.1 Hz, 1H), 4.44 (d, *J* = 8.3 Hz, 1H), 4.41 (d, *J* = 7.9 Hz, 1H), 4.35 (q, *J* = 6.7 Hz, 1H), 4.14 (t, *J* = 10.0 Hz, 1H), 4.13 (d, *J* = 2.8 Hz, 1H), 4.10 (d, *J* = 3.0 Hz, 1H), 4.01-3.98 (m, 2H), 3.96-3.69 (m, 37H), 3.68-3.52 (m, 15H), 3.49 (ddd, *J* = 9.7, 4.6, 2.3 Hz, 1H), 3.31 (t, *J* = 8.7 Hz, 1H), 3.13 (t, *J* = 6.3 Hz, 2H), 2.07 (s, 3H), 2.05 (s, 3H), 2.03 (s, 3H), 1.64-1.59 (m, 2H), 1.50 (quin, *J* = 7.1 Hz, 2H), 1.39-1.31 (m, 4H), 1.28 (d, *J* = 6.7 Hz, 3H), 1.27 (d, *J* = 6.7 Hz, 3H), 1.16 (d, *J* = 6.7 Hz, 3H); ^{13}C NMR (214 MHz, D_2O) δ 175.89, 175.24, 175.11, 159.45, 137.66, 129.74 (×2), 129.27, 128.52 (×2), 104.43, 104.17, 104.10, 103.47, 102.95, 102.77, 101.76, 101.60, 100.53, 99.70, 98.75, 82.97, 82.52, 82.28, 79.97, 77.45, 76.33, 76.24, 76.15, 76.12, 75.82, 75.73, 75.64, 75.44 (×2), 75.43, 74.60, 74.30, 74.07, 73.85, 73.43, 72.96, 72.94, 72.82, 72.73, 71.64, 71.51, 71.50, 70.97, 70.41, 70.17, 70.07, 69.71, 69.63, 69.62, 69.50, 69.40, 69.24,

69.19, 68.78, 68.63, 67.98, 67.68, 67.65, 67.21, 62.56, 62.39, 61.99, 61.44, 61.01, 60.73, 60.43, 56.71, 56.63, 55.65, 41.27, 29.61, 29.56, 26.44, 25.58, 23.47, 23.17, 23.14, 16.32, 16.28, 16.23; HRMS (ESI) *m/z* calcd for $C_{86}H_{140}N_4Na_1O_{55}$ $[M+Na]^+$: 2131.8179; found 2131.8179.

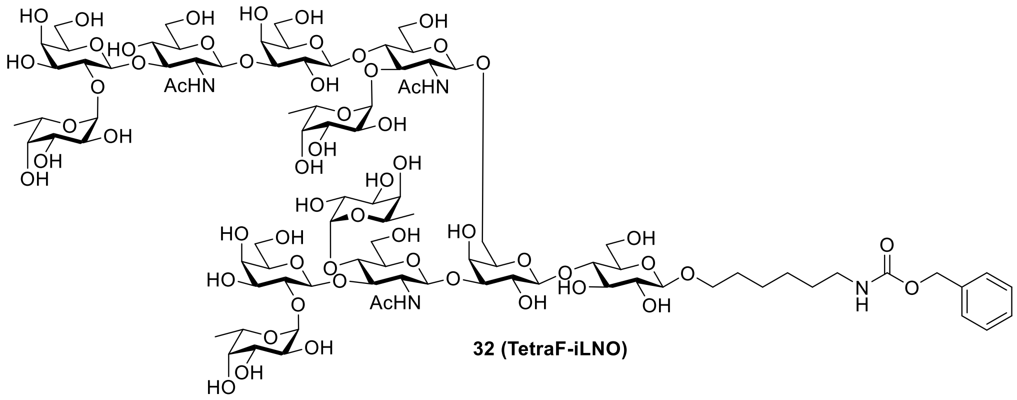

32 (TetraF-iLNO)

Compound 32 (TetraF-*i*LNO). Compound **30** (9.6 mg, 4.9 µmol) and Gal (1.1 mg, 5.9 µmol) were used as the acceptor and the donor precursor, respectively, by following the **general procedure for SNRS-G3**. After being shaken for 4 h, the reaction was heated to 100 ^{o}C and quenched enzyme activity. The resulting product compound **31** (TF-*i*LNO III) was directly used for next step without purification. R_f = 0.20 (*n*-PrOH/ H_2O/AcOH = 5/2/1 (v/v/v), stain run two times). Above resulting product solution and GDP-Fuc (4.7 mg, 7.4 µmol) were used as the acceptor and the donor, respectively, by following the **general procedure for DUSD-F2**. After being shaken for 11 h, the reaction was quenched and purified by following **general purification procedure 1**. After purification, the product was lyophilized to give compound **32** (TetraF-*i*LNO) in 85% yield (9.4 mg) for two steps. R_f = 0.12 (*n*-PrOH/ H_2O/25% NH_4OH = 5/2/1 (v/v/v) stain run two times); ^{1}H NMR (850 MHz, D_2O) δ 7.46-7.40 (m, 5H), 5.20 (d, *J* = 4.0 Hz, 1H), 5.16 (d, *J* = 4.0 Hz, 1H), 5.12 (br, 2H), 5.09 (d, *J* = 4.0 Hz, 1H), 5.04 (d, *J* = 3.8 Hz, 1H), 4.88 (q, *J* = 6.7 Hz, 1H), 4.82 (q, *J* = 6.7 Hz, 1H), 4.67 (d, *J* = 7.6 Hz, 1H), 4.66 (d, *J* = 7.6 Hz, 1H), 4.64 (d, *J* = 7.9 Hz, 1H), 4.63 (d, *J* = 8.5 Hz, 1H), 4.61 (d, *J* = 8.4 Hz, 1H), 4.47 (d, *J* = 7.8 Hz, 1H), 4.43 (d, *J* = 7.8 Hz, 1H), 4.41 (d, *J* = 7.9 Hz, 1H), 4.35 (q, *J* = 6.7 Hz, 1H), 4.30 (q, *J* = 6.7 Hz, 1H), 4.14 (t, *J* = 10.0 Hz, 1H), 4.13 (d, *J* = 2.9 Hz, 1H), 4.09 (d, *J* = 3.2 Hz, 1H), 4.01-3.98 (m, 3H), 3.96-3.68 (m, 40H), 3.66 (dd, *J* = 10.4, 3.3 Hz, 2H), 3.64 (t, *J* = 8.9 Hz, 1H), 3.62 (dd, *J* = 9.5, 8.2 Hz, 1H), 3.62-3.53 (m, 8H), 3.56 (dd, *J* = 9.6, 8.2 H, 1H), 3.50-3.48 (m, 2H), 3.31 (t, *J* = 8.7 Hz, 1H), 3.12 (t, *J* = 6.4 Hz, 2H), 2.07 (s, 3H), 2.06 (s, 3H), 2.05 (s, 3H), 1.63-1.60 (m, 2H), 1.49 (quin, *J* = 7.1 Hz, 2H), 1.38-1.30 (m, 4H), 1.28 (d, *J* = 6.7 Hz, 3H), 1.27 (d, *J* = 6.7 Hz, 3H), 1.24 (d, *J* = 6.7 Hz, 3H) 1.15 (d, *J* = 6.7 Hz, 3H); ^{13}C NMR (214 MHz, D_2O) δ 175.24, 175.16, 175.10, 159.43, 137.65, 129.74 (×2), 129.26, 128.52 (×2), 104.18, 104.16, 104.10, 102.94, 102.76, 101.75, 101.59, 101.21, 100.52, 100.47, 99.79, 98.74, 82.26, 82.18, 79.98, 78.14, 77.62, 77.44, 76.33,

76.15 (×2), 76.02, 75.92, 75.73, 75.63, 75.43 (×2), 75.33, 74.60, 74.45, 74.28, 73.89, 73.83, 72.96, 72.93, 72.84, 72.82, 72.73, 71.73, 71.50, 70.97, 70.41, 70.37, 70.18, 70.09, 70.07, 69.70, 69.60 (×2), 69.47, 69.42, 69.23, 68.92, 68.77, 68.59, 67.98, 67.67, 67.65, 67.43, 67.20, 62.55, 62.40, 62.11, 61.34, 61.01, 60.73, 60.43, 56.70, 56.63, 55.99, 41.26, 29.61, 29.56, 26.44, 25.57, 23.47, 23.13, 23.10, 16.32, 16.29, 16.27, 16.25; HRMS (ESI) *m/z* calcd for $C_{92}H_{149}N_4O_{59}$ $[M-H]^-$: 2253.8782; found 2253.8881.

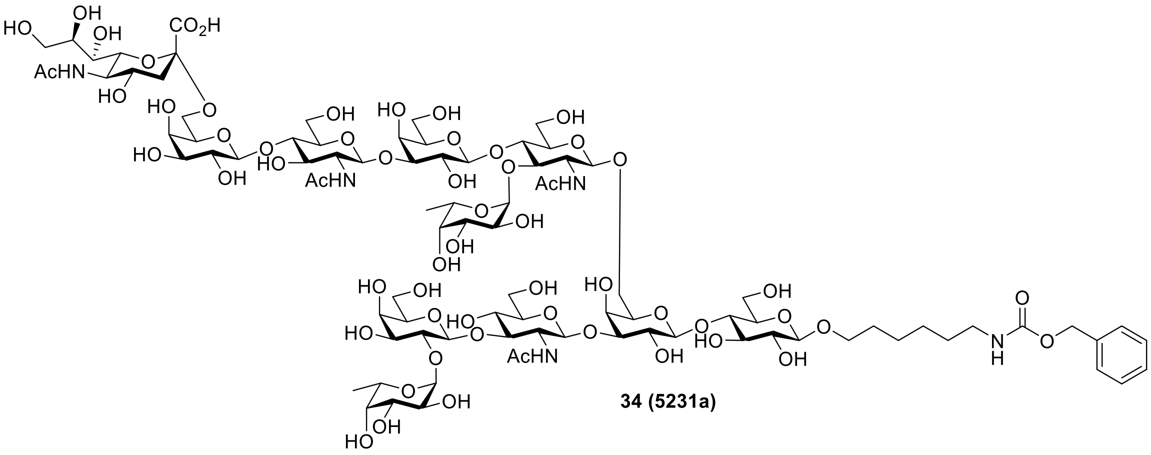

34 (5231a)

Compound 34 (5231a). Compound **29** (10 mg, 5.5 µmol) and Gal (1.1 mg, 6.1 µmol) were used as the acceptor and the donor precursor, respectively, by following the **general procedure for SNRS-G4b**. After being shaken for 30 min, the reaction was quenched and purified by following **general purification procedure 1**. After purification, the product was lyophilized to give intermediate compound **33** in 86% yield (9.4 mg). R_f = 0.17 (*n*-PrOH/ H_2O/AcOH = 6/2/1 (v/v/v)). Compound **33** (7.4 mg, 3.8 µmol) and Neu5Ac (1.8 mg, 6.1 µmol) were used as the acceptor and the donor precursor, respectively, by following the **general procedure for SOPE-S6a**. After being shaken for 2 h, the reaction was quenched and purified by following **general purification procedure 2**. After purification, the product was lyophilized to give compound **34** (5231a) in 64% separation yield (6.2 mg) (two steps 55%). R_f = 0.49 (*n*-PrOH/ H_2O/NH_4OH = 4/2/1 (v/v/v)); ^{1}H NMR (850 MHz, D_2O) δ 7.46-7.40 (m, 5H), 5.20 (d, *J* = 4.0 Hz, 1H), 5.12 (br, 2H), 5.09 (d, *J* = 3.8 Hz, 1H), 4.82 (q, *J* = 6.7 Hz, 1H, overlapping D_2O), 4.73 (d, *J* = 7.9 Hz, 1H), 4.65 (d, *J* = 7.7 Hz, 1H), 4.64 (d, *J* = 8.1 Hz, 1H), 4.63 (d, *J* = 8.5 Hz, 1H), 4.464 (d, *J* = 7.9 Hz, 1H), 4.460 (d, *J* = 7.8 Hz, 1H), 4.44 (d, *J* = 7.8 Hz, 1H), 4.41 (d, *J* = 7.8 Hz, 1H), 4.30 (q, *J* = 6.7 Hz, 1H,), 4.13 (d, *J* = 2.7 Hz, 1H), 4.10 (d, *J* = 2.8 Hz, 1H), 4.02-3.97 (m, 4H), 3.96-3.52 (m, 55H), 3.51-3.49 (m, 1H), 3.30 (t, *J* = 8.5 Hz, 1H), 3.12 (t, *J* = 6.2 Hz, 2H), 2.68 (dd, *J* = 12.4 , 4.5 Hz, 1H), 2.06 (s, 3H), 2.054 (s, 3H), 2.050 (s, 3H), 2.03 (s, 3H), 1.73 (t, *J* = 12.2 Hz, 1H), 1.63-1.60 (m, 2H), 1.49 (quin, *J* = 7.1 Hz, 2H), 1.38-1.30 (m, 4H), 1.24 (d, *J* = 6.7 Hz, 3H), 1.16 (d, *J* = 6.7 Hz, 3H); ^{13}C NMR (214 MHz, D_2O) δ 175.87, 175.82, 175.24, 175.17, 174.41, 159.45, 137.66, 129.74 (×2), 129.27, 128.52 (×2), 104.46, 104.16, 104.08, 103.48, 102.95, 102.78, 101.79, 101.21,

101.06, 100.48, 99.69, 82.56, 82.30, 81.48, 79.97, 78.13, 77.62, 76.32, 76.24, 76.04, 75.82, 75.64, 75.45, 75.42, 75.21, 74.66, 74.47, 74.31, 74.04, 73.84, 73.52, 73.40, 73.19, 72.82, 72.65, 71.71, 71.50, 71.45, 71.02, 70.40, 70.17, 70.10, 69.71, 69.59, 69.42, 69.38, 69.33, 69.20, 69.14, 69.02, 68.64, 67.89, 67.70, 67.65, 67.46, 64.31, 63.64, 62.42, 62.11, 61.37, 61.11, 61.04, 60.71, 56.63, 55.93, 55.87, 52.85, 41.27, 41.02, 29.61, 29.56, 26.44, 25.58, 23.46, 23.23, 23.11, 22.99, 16.23, 16.22; HRMS (ESI) *m/z* calcd for $C_{91}H_{146}N_5O_{59}$ $[M-H]^-$: 2252.8578; found 2252.8571.

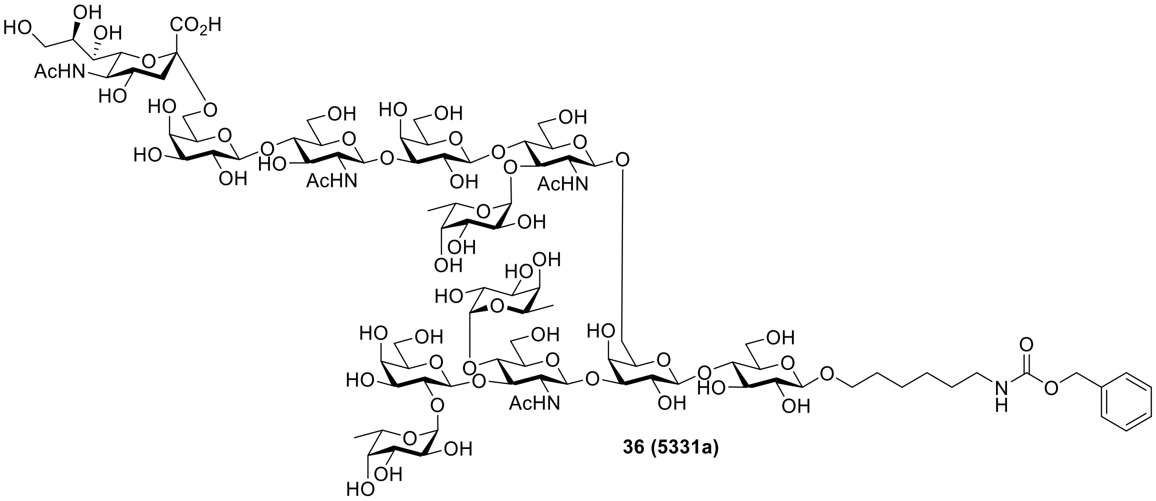

Compound 36 (5331a). Compound **30** (6 mg, 3.1 µmol) and Gal (0.7 mg, 3.7 µmol) were used as the acceptor and the donor precursor, respectively, by following the **general procedure for SNRS-G4b.** After being shaken for 30 min, the reaction was quenched and purified by following **general purification procedure 1**. After purification, the product was lyophilized to give intermediate compound **35** in 95% yield (6.0 mg). R_f = 0.14 (*n*-PrOH/ H_2O/AcOH = 5/2/1 (v/v/v)). Compound **35** (6 mg, 2.9 µmol) and Neu5Ac (1.3 mg, 4.3 µmol) were used as the acceptor and the donor precursor, respectively, by following the **general procedure for SOPE-S6a**. After being shaken for 2 h, the reaction was quenched and purified by following **general purification procedure 3**. After purification, the product was lyophilized to give compound **36** (5331a) in 80% yield (5.5 mg)(two steps 76%). R_f = 0.25 (*n*-PrOH/ H_2O/NH_4OH = 6/2/1 (v/v/v)); ^{1}H NMR (850 MHz, D_2O) δ 7.46-7.40 (m, 5H), 5.16 (d, J = 4.0 Hz, 1H), 5.12 (br, 2H), 5.09 (d, J = 3.8 Hz, 1H), 5.04 (d, J = 3.7 Hz, 1H), 4.87 (q, J = 6.7 Hz, 1H), 4.82 (q, J = 6.7 Hz, 1H, overlapping D_2O), 4.73 (d, J = 7.9 Hz, 1H), 4.66 (d, J = 7.7 Hz, 1H), 4.64 (d, J = 7.7 Hz, 1H), 4.61 (d, J = 8.3 Hz, 1H), 4.47 (d, J = 8.3 Hz, 1H), 4.46 (d, J = 7.8 Hz, 1H), 4.45 (d, J = 7.9 Hz, 1H), 4.41 (d, J = 7.9 Hz, 1H), 4.35 (q, J = 6.7 Hz, 1H), 4.14 (t, J = 10.0 Hz, 1H), 4.13 (d, J = 2.6 Hz, 1H), 4.10 (d, J = 3.1 Hz, 1H), 4.01-3.98 (m, 3H), 3.96-3.52 (m, 59H), 3.31 (t, J = 8.7 Hz, 1H), 3.12 (t, J = 6.3 Hz, 2H), 2.68 (dd, J = 12.4 , 4.6 Hz, 1H), 2.07 (s, 3H), 2.06 (s, 3H), 2.05 (s, 3H), 2.04 (s, 3H), 1.73 (t, J = 12.4 Hz, 1H), 1.63-1.60 (m, 2H), 1.49 (quin, J = 7.1 Hz, 2H), 1.38-1.30 (m, 4H), 1.28 (d, J =

6.7 Hz, 3H), 1.27 (d, J = 6.7 Hz, 3H), 1.16 (d, J = 6.7 Hz, 3H); ^{13}C NMR (214 MHz, D_2O) δ 175.87, 175.82, 175.23, 175.10, 174.49, 159.44, 137.66, 129.74 (×2), 129.27, 128.52 (×2), 104.45, 104.17, 104.09, 103.48, 102.94, 102.78, 101.80, 101.60, 101.11, 100.53, 99.68, 98.75, 82.55, 82.32, 81.47, 79.96, 77.45, 76.33, 76.15, 75.82, 75.73, 75.64, 75.44 (×2), 75.42, 75.21, 74.67, 74.60, 74.29, 74.04, 73.84, 73.51, 73.40, 73.19, 72.97, 72.93, 72.81, 72.73, 72.68, 71.71, 71.50, 71.45, 70.97, 70.41, 70.17, 70.07, 69.71, 69.69, 69.60, 69.38, 69.33, 69.24, 69.20, 69.17, 68.77, 68.63, 67.98, 67.70, 67.65, 67.21, 64.31, 63.63, 62.55, 62.42, 61.11, 61.02, 60.71, 60.43, 56.70, 56.62, 55.87, 52.86, 41.27, 41.05, 29.61, 29.56, 26.44, 25.58, 23.47, 23.23, 23.14, 22.98, 16.32, 16.28, 16.23; HRMS (ESI) *m/z* calcd for $C_{97}H_{156}N_5O_{63}$ $[M-H]^-$: 2398.9157; found 2398.8980.

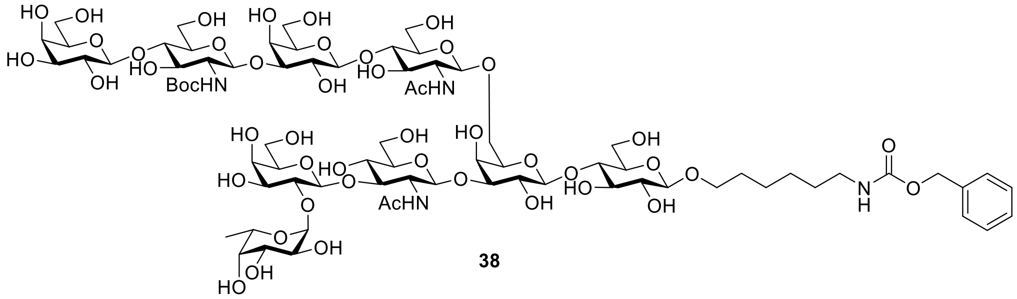

38

Compound 38. Compound **20** (FLNH1) (5.2 mg, 3.6 μmol) and GlcNTFA (1.5 mg, 5.4 μmol) were used as the acceptor and the donor precursor, respectively, by following the **general procedure for SNRS-NTFA**. After being shaken for 5 days, the reaction was quenched and the mixture was purified by following **general purification procedure 1**. After purification, the product was lyophilized to give intermediate compound **37**. R_f = 0.37 (*n*-PrOH/ H_2O/NH_4OH = 6/2/1 (v/v/v)). The **CC2 procedure** was applied on intermediate compound **37** to give GlcNBoc containing product. The above GlcNBoc containing product and Gal (0.6 mg, 3.4μmol) were used as the acceptor and the donor precursor, respectively, by following the **general procedure for SNRS-G4b**. After being shaken for 28 h, the reaction was lyophilized to give compound **38** in 73% for three-step yield (4.9 mg). GlcNBoc containing intermediate, R_f = 0.47 (*n*-PrOH/ H_2O/AcOH = 6/2/1 (v/v/v)), compound **38**, R_f = 0.38 (*n*-PrOH/ H_2O/AcOH = 6/2/1 (v/v/v)); ^{1}H NMR (850 MHz, D_2O) δ 7.48-7.40 (m, 5H), 5.205 (d, J = 4.1 Hz, 1H), 5.12 (br, 2H), 4.75 (d, J = 8.5 Hz, 1H), 4.66 (d, J = 7.7 Hz, 1H), 4.65 (d, J = 8.5 Hz, 1H), 4.64 (d, J = 8.5 Hz, 1H), 4.49 (d, J = 7.8 Hz, 2H), 4.47 (d, J = 8.0 Hz, 1H), 4.42 (d, J = 7.9 Hz, 1H), 4.31 (q, J = 6.7 Hz, 1H), 4.144 (d, J = 3.5 Hz, 1H), 4.14 (d, J = 3.5 Hz, 1H), 4.02-3.99 (m, 3H), 3.97 (dd, J = 12.6, 6.6 Hz, 1H), 3.97 (dd, J = 12.1, 6.1 Hz, 1H), 3.94 (d, J = 3.4 Hz, 1H), 3.93-3.89 (m, 3H), 3.87-3.83 (m, 6H), 3.825-3.71 (m, 18H), 3.705-3.632 (m, 7H), 3.63-3.53 (m, 8H), 3.506 (ddd, J = 9.9, 4.6, 2.2 Hz, 1H), 3.47 (dd, J = 9.9, 8.8 Hz, 1H), 3.32 (t, J = 8.6 Hz, 1H), 3.13 (t, J = 6.5 Hz, 2H), 2.07 (s, 3H), 2.065 (s, 3H), 1.65-1.60 (m, 2H), 1.50 (quin, J = 7.1 Hz, 2H),1.46 (s, 9H), 1.39-1.31 (m, 4H), 1.25 (d, J =

6.6 Hz, 3H),; ^{13}C NMR (214 MHz, D_2O) δ175.46, 175.17, 159.43, 159.19, 137.65, 129.74 (×2), 129.27, 128.52 (×2), 104.15, 103.98, 103.82 (×2), 103.46, 102.95, 101.94, 101.20, 100.47, 82.28, 82.21, 79.79, 79.39, 79.33, 78.12, 77.61, 76.33, 76.23, 76.03, 75.91, 75.69, 75.62, 75.56, 75.41, 74.45, 74.40, 73.85, 73.48, 73.40, 73.32, 72.81, 71.93, 71.49, 71.29, 71.01, 70.39, 70.09, 69.65, 69.63, 69.53, 69.42, 69.20, 69.01, 67.65, 67.45, 62.10, 62.01 61.91, 61.37, 61.03, 61.01, 60.90, 57.48, 55.93 (×2), 41.26, 29.60, 29.55, 28.63 (×3), 26.43, 25.57, 23.37, 23.10, 22.56, 16.21; HRMS (ESI) *m/z* calcd for $C_{77}H_{126}N_4O_{48}$ $[M+Na]^+$: 1897.7440; found 1897.7433.

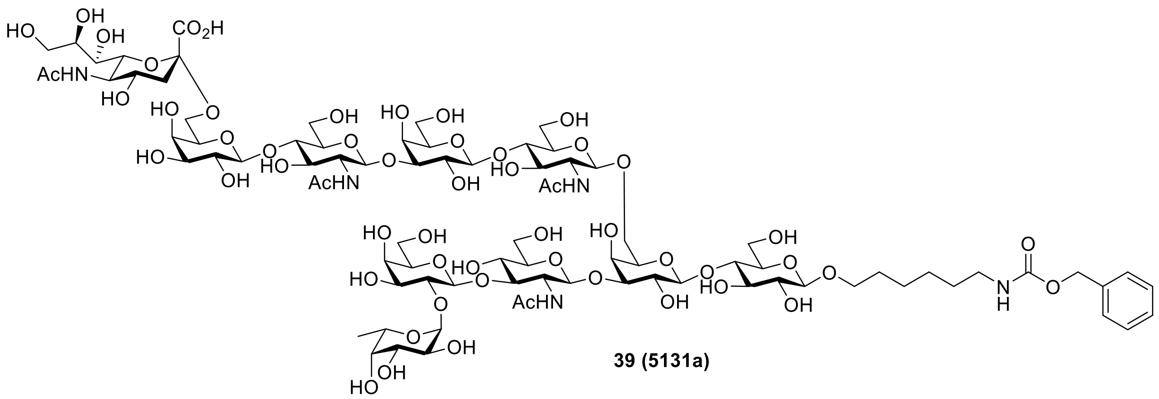

39 (5131a)

Compound 39 (5131a). Compound **38** (4.4 mg, 2.4 µmol) and Neu5Ac (1.1 mg, 3.5 µmol) were used as the acceptor and the donor precursor, respectively, by following the **general procedure for SOPE-6b**. After being shaken 1 h, the reaction was quenched and purified by following the **general purification procedure 3**. The **CC3 procedure** was applied on Neu5Ac product to give corresponding compound **39** (5131a) in 51% for two steps yield (2.5 mg). R_f = 0.12 (*n*-PrOH/ H_2O/ NH_4OH = 5.5/2/1 (v/v/v)); ^{1}H NMR (850 MHz, D_2O) δ 7.46-7.40 (m, 5H), 5.20 (d, *J* = 4.1 Hz, 1H), 5.11 (br, 2H), 4.74 (d, *J* = 8.1 Hz, 1H), 4.65 (d, *J* = 7.7 Hz, 1H), 4.633 (d, *J* = 8.0 Hz, 1H), 4.628 (d, *J* = 8.4 Hz, 1H), 4.463 (d, *J* = 7.7 Hz, 1H), 4.458 (d, *J* = 7.8 Hz, 2H), 4.41 (d, *J* = 7.9 Hz, 1H), 4.30 (q, *J* = 6.7 Hz, 1H), 4.16 (d, *J* = 3.1 Hz, 1H), 4.13 (d, *J* = 3.1 Hz, 1H), 4.01-3.87 (m, 11H), 3.93 (d, *J* = 3.2 Hz, 1H), 3.86-3.54 (m, 40H), 3.60 (t, *J* = 8.5 Hz, 2H), 3.53 (t, *J* = 8.5 Hz, 2H), 3.50 (ddd, *J* = 9.8, 4.3, 2.2 Hz, 1H), 3.31 (t, *J* = 8.6 Hz, 1H), 3.12 (t, *J* = 6.4 Hz, 2H), 2.68 (dd, *J* = 12.4, 4.6 Hz, 1H), 2.063 (s, 6H), 2.057 (s, 3H), 2.04 (s, 3H), 1.73 (t, *J* = 12.4 Hz, 1H), 1.63-1.60 (m, 2H), 1.49 (quin, *J* = 7.1 Hz, 2H), 1.38-1.30 (m, 4H), 1.24 (d, *J* = 6.7 Hz, 3H); ^{13}C NMR (214 MHz, D_2O) δ 175.86 (×2), 175.43, 175.15, 174.48, , 159.41, 137.64, 129.73 (×2), 129.26, 128.52 (×2), 104.42, 104.15, 103.98, 103.87, 103.53, 102.95, 101.96, 101.20, 101.10, 100.46, 82.94, 82.29, 81.42, 79.80, 79.38, 78.12, 77.61, 76.22, 76.03, 75.83, 75.70, 75.62, 75.41, 75.22, 74.65, 74.45, 74.37, 73.83, 73.50, 73.39 (×2), 73.19, 72.81, 72.67, 71.69, 71.48, 71.01, 70.93, 70.38, 70.09, 69.69, 69.61, 69.41, 69.37, 69.33, 69.26, 69.16, 69.01, 67.63, 67.44, 64.30, 63.62, 62.10, 61.91, 61.36, 61.10, 61.01, 60.97, 55.91 (×3), 52.85, 41.26, 41.04, 29.61, 29.55, 26.44,

25.57, 23.37, 23.24, 23.10, 22.98, 16.21; HRMS (ESI) *m/z* calcd for $C_{85}H_{137}N_5O_{55}$ $[M]^-$: 2107.8077; found 2107.8079.

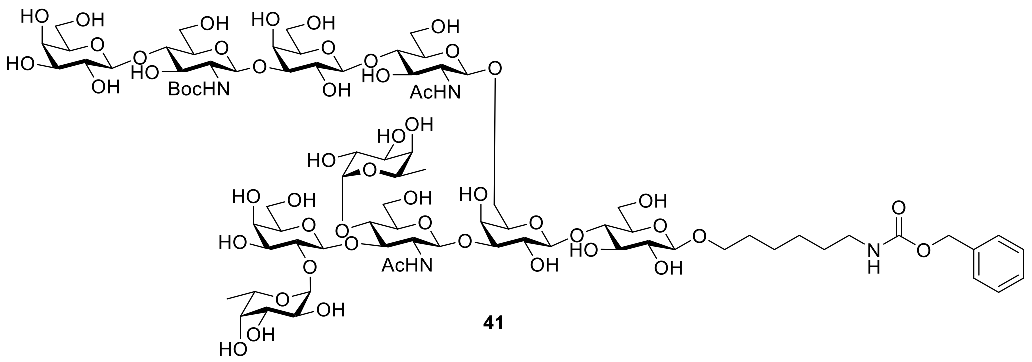

41

Compound 41. Compound **24** (DF-LNH c) (5 mg, 3.1 μmol) and GlcNTFA (1.3 mg, 4.7 μmol) were used as the acceptor and the donor precursor, respectively, by following the **general procedure for SNRS-NTFA**. After being shaken for 5 days, the reaction was quenched and the mixture was purified by following **general purification procedure 1**. After purification, the product was lyophilized to give intermediate compound **40**. R_f = 0.23 (*n*-PrOH/ H_2O/NH_4OH = 6/2/1 (v/v/v)). The **CC2 procedure** was applied on intermediate compound **40** to give corresponding GlcNBoc product. The above GlcNBoc containing product and Gal (0.7 mg, 3.6μmol) were used as the acceptor and the donor precursor, respectively, by following the **general procedure for SNRS-G4b**. After being shaken for 17 h, the reaction was lyophilized to give compound **41** in 53% for three steps yield (3.3 mg). GlcNBoc containing internediate, R_f = 0.38 (*n*-PrOH/ H_2O/AcOH = 6/2/1 (v/v/v)), compound **41**, R_f = 0.13 (*n*-PrOH/ H_2O/AcOH = 6/2/1 (v/v/v)); ^{1}H NMR (850 MHz, D_2O) δ 7.48-7.39 (m, 5H), 5.16 (d, *J* = 4.0 Hz, 1H), 5.12 (br, 2H), 5.04 (d, *J* = 3.8 Hz, 1H), 4.875 (q, *J* = 6.7 Hz, 1H), 4.74 (d, *J* = 8.4 Hz, 1H), 4.67 (d, *J* = 7.7 Hz, 1H), 4.64 (d, *J* = 8.1 Hz, 1H), 4.61 (d, *J* = 8.4 Hz, 1H), 4.48 (d, *J* = 7.8 Hz, 2H), 4.47 (d, *J* = 8.2 Hz, 1H), 4.41 (d, *J* = 7.9 Hz, 1H), 4.35 (q, *J* = 6.7 Hz, 1H), 4.16-4.12 (m, 3H), 4.02-3.98 (m, 2H), 3.98-3.92 (m, 5H), 3.91-3.82 (m, 10H), 3.81-3.69 (m, 21H), 3.68 (d, *J* = 4.1 Hz, 1H), 3.666 (d, *J* = 3.3 Hz, 1H), 3.66-3.60 (m, 5H), 3.60-3.52 (m, 7H), 3.47 (t, *J* = 9.4 Hz, 1H), 3.31 (t, *J* = 8.8 Hz, 1H), 3.13 (t, *J* = 6.4 Hz, 2H), 2.07 (s, 3H), 2.06 (s, 3H), 1.65-1.58 (m, 2H), 1.49 (quin, *J* = 7.1 Hz, 2H),1.45 (s, 9H), 1.40-1.30 (m, 4H), 1.28 (d, *J* = 6.6 Hz, 3H), 1.27 (d, *J* = 6.6 Hz, 3H); ^{13}C NMR (214 MHz, D_2O) δ 175.44, 175.10, 159.44, 159.21, 137.65, 129.74 (×2), 129.27, 128.53 (×2), 104.17, 104.00, 103.83 (×2), 103.80, 103.47, 102.96, 101.94, 101.60, 100.53, 98.75, 82.29, 82.20, 79.80, 79.40, 79.34, 77.45, 76.34 (×2), 76.16, 75.92, 75.73, 75.69, 75.63, 75.56, 75.44, 75.41, 74.60, 74.39, 73.85, 73.49, 73.41, 73.33, 72.96, 72.94, 72.73, 71.94, 71.49, 71.29, 70.97, 70.41, 70.07, 69.71, 69.65, 69.63, 69.54, 69.24, 69.21, 68.78, 67.98, 67.65, 67.21, 62.55, 62.01, 61.91, 61.05, 61.00, 60.90, 60.44, 57.49, 56.70, 55.94, 41.27, 29.61, 29.56, 28.63 (×3), 26.44, 25.58, 23.38, 23.14, 16.32, 16.27; HRMS (ESI) *m/z* calcd for $C_{83}H_{137}N_4Na_1O_{52}$

[M+Na]$^+$: 2044.8097; found 2044.8117

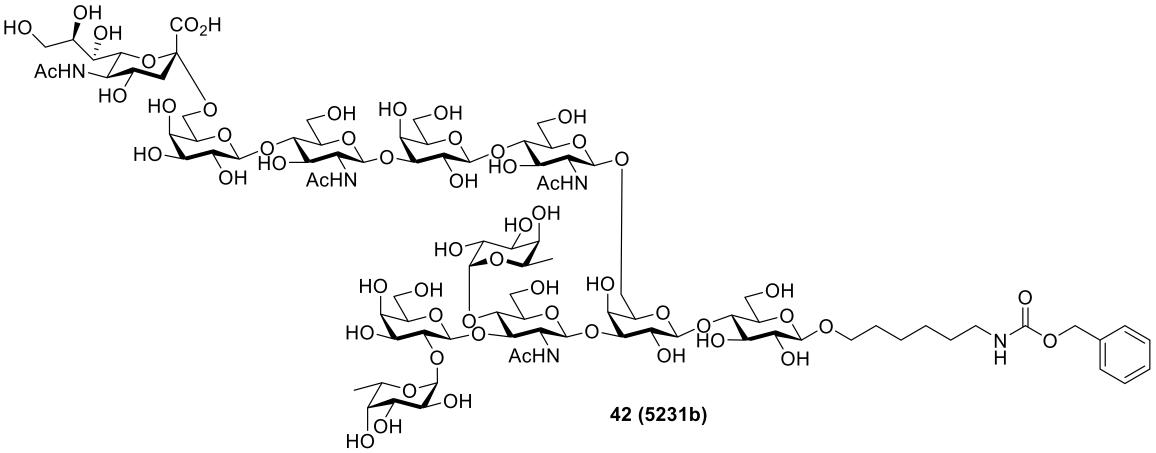

42 (5231b)

Compound 42 (5231b). Compound **41** (3.3 mg, 1.6 µmol) and Neu5Ac (0.8 mg, 2.5 µmol) were used as the acceptor and the donor precursor, respectively, by following the **general procedure for SOPE-6b**. After being shaken for 1 h, the reaction was quenched and purified by following the **general purification procedure 3**. The **CC3 procedure** was applied on Neu5Ac product to give corresponding compound **42** (5231b) in 88% for two steps yield (3.26mg). R_f = 0.07 (*n*-PrOH/ H_2O/ NH_4OH = 5.5/2/1 (v/v/v)); ^{1}H NMR (850 MHz, D_2O) δ 7.47-7.40 (m, 5H), 5.16 (d, *J* = 4.0 Hz, 1H), 5.12 (br, 2H), 5.04 (d, *J* = 3.8 Hz, 1H), 4.88 (q, *J* = 6.7 Hz, 1H), 4.74 (d, *J* = 8.1 Hz, 1H), 4.67 (d, *J* = 7.7 Hz, 2H), 4.64 (d, *J* = 8.2 Hz, 1H), 4.61 (d, *J* = 8.4 Hz, 1H), 4.47 (d, *J* = 7.7 Hz, 1H), 4.46 (d, *J* = 7.7 Hz, 1H), 4.41 (d, *J* = 7.9 Hz, 1H), 4.35 (q, *J* = 6.7 Hz, 1H), 4.16 (d, *J* = 3.1 Hz, 1H), 4.14 (t, *J* = 9.9 Hz, 1H), 4.13 (d, *J* = 3.1 Hz, 1H), 4.02-3.99 (m, 3H), 3.96-3.92 (m, 5H), 3.92-3.52 (m, 51H), 3.31 (t, *J* = 8.6 Hz, 1H), 3.13 (t, *J* = 6.4 Hz, 2H), 2.68 (dd, *J* = 12.4 , 4.6 Hz, 1H), 2.07 (s, 3H), 2.064 (s, 3H), 2.059 (s, 3H), 2.04 (s, 3H), 1.73 (t, *J* = 12.4 Hz, 1H), 1.64-1.60 (m, 2H), 1.50 (quin, *J* = 7.1 Hz, 2H), 1.39-1.31 (m, 4H), 1.28 (d, *J* = 6.7 Hz, 3H), 1.27 (d, *J* = 6.7 Hz, 3H); ^{13}C NMR (214 MHz, D_2O) δ 175.87 (×2), 175.45, 175.10, 174.50, 159.45, 137.66, 129.74 (×2), 129.27, 128.52 (×2), 104.44, 104.18, 104.01, 103.88, 103.54, 102.96, 101.98, 101.60, 101.11, 100.53, 98.75, 82.95, 82.33, 81.44, 79.81, 79.39, 77.45, 76.15, 75.85, 75.73 (×2), 75.64, 75.45, 75.42, 75.24, 74.67, 74.60, 74.38, 73.85, 73.51, 73.42, 73.40 , 73.21, 72.97, 72.93, 72.73, 72.68, 71.71, 71.50, 70.97, 70.94, 70.41, 70.07, 69.71 (×2), 69.63, 69.38, 69.34, 69.28, 69.24, 69.18, 68.77, 67.98, 67.65, 67.22, 64.31, 63.63, 62.55, 61.93, 61.11, 61.01, 60.98, 60.43, 56.70, 55.92 (×2), 52.86, 41.26, 41.06, 29.61, 29.56, 26.44, 25.58, 23.38, 23.25, 23.14, 22.98, 16.32, 16.27; HRMS (ESI) *m/z* calcd for $C_{91}H_{147}N_5O_{59}$ [M]$^-$: 2253.8656; found 2253.8658.

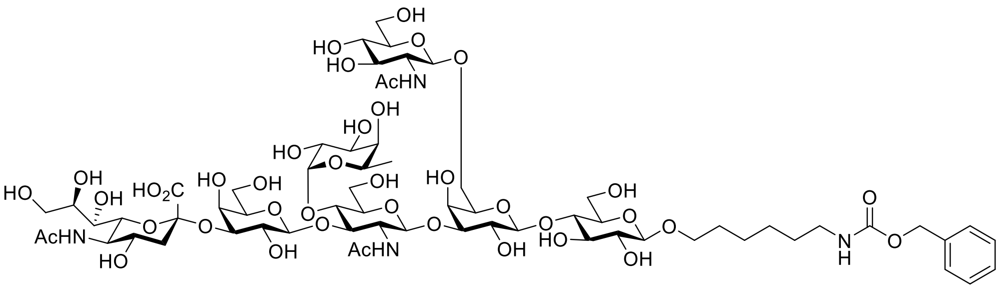

43

Compound 43. Compound **15** (52 mg, 40.3 µmol) and Neu5Ac (25 mg, 80.6 µmol) were used as the acceptor and the donor precursor, respectively, by following the **general procedure for SOPE-S3**. After being shaken for 31 h, the reaction was quenched and the mixture was purified by following the **general purification procedure 2**. After purification, the product was lyophilized to give compound **43** in 75% separation yield (42.5 mg). R_f = 0.25 (*n*-PrOH/ H_2O/NH_4OH = 6/2/1 (v/v/v)); ^1H NMR (850 MHz, D_2O) δ 7.48-7.39 (m, 5H), 5.12 (br, 2H), 5.03 (d, *J* = 3.8 Hz, 1H), 4.88 (q, *J* = 6.7 Hz, 1H), 4.72 (d, *J* = 8.3 Hz, 1H), 4.623 (d, *J* = 8.5 Hz, 1H), 4.56 (d, *J* = 7.7 Hz, 1H), 4.47 (d, *J* = 7.7 Hz, 1H), 4.43 (d, *J* = 7.8 Hz, 1H), 4.15 (d, *J* = 3.1 Hz, 1H), 4.10 (t, *J* = 10.1 Hz, 1H), 4.06 (dd, *J* = 9.8, 2.6 Hz, 1H), 4.00 (dd, *J* = 9.7, 1.5 Hz, 1H), 3.98-3.94 (m, 3H), 3.93-3.875 (m, 4H), 3.87-3.83 (m, 5H), 3.82-3.74 (m, 7H), 3.73-3.69 (m, 4H), 3.69-3.61 (m, 8H), 3.61-3.51 (m, 9H 可能含 Tris-HCl), 3.48 (ddd, *J* = 9.7, 5.2, 1.6 Hz, 1H), 3.46 (q, *J* = 9.0 Hz, 1H), 3.31 (t, *J* = 8.8 Hz, 1H), 3.13 (t, *J* = 6.5 Hz, 3H), 2.78 (dd, *J* = 12.3 , 4.4 Hz, 1H), 2.07 (s, 3H), 2.05 (s, 3H), 2.04 (s, 3H), 1.78 (t, *J* = 12.4 Hz, 1H), 1.64-1.59 (m, 2H), 1.50 (quin, *J* = 7.1 Hz, 2H), 1.40-1.29 (m, 4H), 1.19 (d, *J* = 6.5 Hz, 3H); ^{13}C NMR (214 MHz, D_2O) δ 175.91, 175.56, 175.51, 174.89, 161.78 (CO_3^-), 159.45, 137.66, 129.74 (×2), 129.26, 128.51 (×2), 104.00, 103.72, 103.47, 102.95, 102.02, 100.36, 98.96, 82.76, 79.97, 76.86, 76.82, 76.59, 76.24, 75.71, 75.62, 75.44, 74.85, 74.43, 73.85, 73.72, 73.01 (x2), 72.98, 72.91, 72.82, 71.50, 70.83, 70.78, 70.07, 69.77, 69.63, 69.37, 69.32, 69.00, 68.77, 67.90, 67.79, 67.66, 63.44 (Tris-HCl), 63.29, 62.62, 61.65, 61.00, 60.58, 56.80, 56.46, 52.64, 41.26, 40.97, 29.60, 29.55, 26.43, 25.57, 23.37 (×2), 23.00, 16.29; HRMS (ESI) *m/z* calcd for $C_{65}H_{103}N_4O_{40}$ [M-H]$^-$: 1579.6149; found 1579.6147.

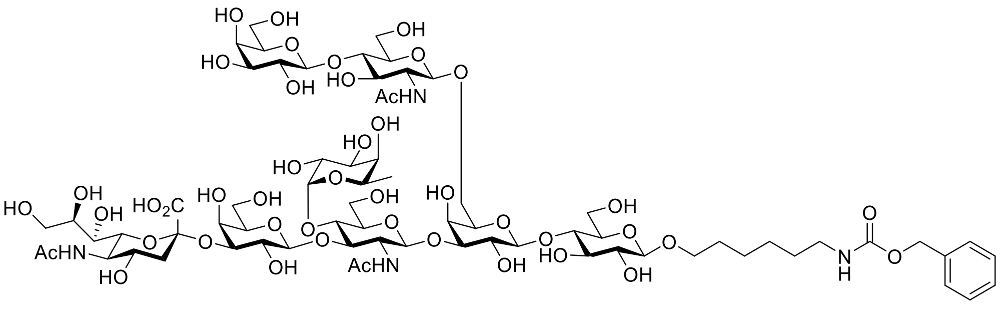

44 (FS-LNH IV)

Compound 44 (FS-LNH IV). Compound **43** (38.8 mg, 24.5 μmol) and Gal (5.3 mg, 29.5 μmol) were used as the acceptor and the donor precursor, respectively, by following the **general procedure for SNRS-G4b**. After being shaken for 2h, the reaction was quenched and the mixture was purified by following **general purification procedure 1**. After purification, the product was lyophilized to give compound **44** (FS-LNH IV) in 94% yield (40.4 mg). R_f = 0.22 (*n*-PrOH/ H_2O/AcOH = 6/2/1 (v/v/v)); ^{1}H NMR (850 MHz, D_2O) : δ 7.53-7.33 (m, 5H), 5.11 (br, 2H), 5.02 (d, *J* = 3.8 Hz, 1H), 4.88 (q, *J* = 6.5 Hz, 1H), 4.71 (d, *J* = 8.4 Hz, 1H), 4.64 (d, *J* = 8.2 Hz, 1H), 4.56 (d, *J* = 7.7 Hz, 1H), 4.47 (d, *J* = 7.8 Hz, 1H), 4.46 (d, *J* = 7.6 Hz, 1H), 4.42 (d, *J* = 7.8 Hz, 1H), 4.144 (d, *J* = 3.1 Hz, 1H), 4.10 (t, *J* = 9.7 Hz, 1H), 4.06 (dd, *J* = 9.8, 3.0 Hz, 1H), 3.996 (d, *J* = 9.7 Hz, 1H), 3.993 (d, *J* = 10.5 Hz, 1H), 3.98-3.93 (m, 3H), 3.93-3.91 (m, 2H), 3.91-3.81 (m, 9H), 3.81-3.78 (m, 4H), 3.78-3.76 (m, 1H), 3.76-3.74 (m, 2H), 3.74-3.69 (m, 7H), 3.69-3.63 (m, 5H), 3.63-3.59 (m, 3H), 3.59-3.56 (m, 2H), 3.544 (dd, *J* = 9.8, 7.7 Hz, 2H), 3.56-3.53 (m, 1H), 3.52 (dd, *J* = 9.4, 8.1 Hz, 1H), 3.31 (t, *J* = 8.6 Hz, 1H), 3.12 (t, *J* = 6.2 Hz, 2H), 2.77 (dd, *J* = 12.4, 4.4 Hz, 1H), 2.06 (s, 3H), 2.04 (s, 3H), 2.036 (s, 3H), 1.80 (t, *J* = 12.3 Hz, 1H), 1.66-1.56 (m, 2H), 1.49 (quin, *J* = 7.0 Hz, 2H), 1.39-1.29 (m, 4H), 1.18 (d, *J* = 6.5 Hz, 3H); ^{13}C NMR (214 MHz, D_2O) : δ 175.90, 175.57, 175.44, 174.53, 159.44, 137.65, 129.74 (×2), 129.27, 128.52 (×2), 104.00, 103.85, 103.70, 103.46, 102.96, 101.94, 100.19, 98.96, 82.76, 79.97, 79.35, 76.86, 76.58, 76.31, 76.24, 75.72, 75.68, 75.62, 75.46, 74.41, 73.84, 73.77, 73.47, 73.42, 72.98, 72.91, 72.70, 71.92, 71.49, 70.78, 70.08, 69.77, 69.65, 69.52, 69.32, 69.23, 69.03, 68.77, 67.93, 67.78, 67.64, 63.38, 62.58, 61.98, 60.99 (×2), 60.59, 56.80, 55.98, 52.64, 41.27, 40.81, 29.61, 29.56, 26.44, 25.58, 23.38, 23.37, 23.00, 16.29; HRMS (ESI) *m/z* calcd for $C_{71}H_{113}N_4O_{45}$ $[M-H]^-$: 1741.6677; found 1741.6683.

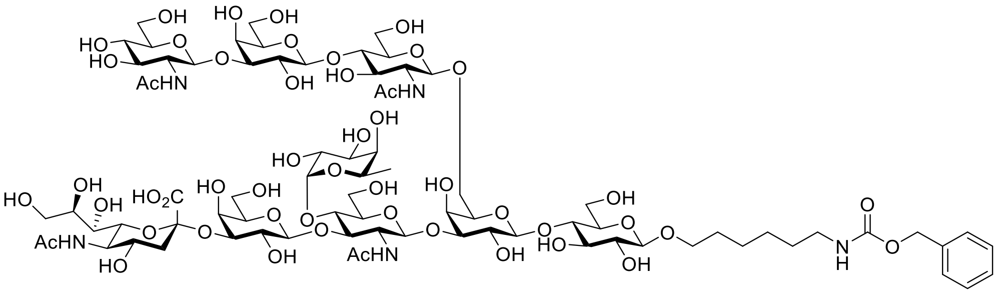

45

Compound 45. Compound **44** (FS-LNH IV) (15 mg, 8.6 μmol) and GlcNAc (6.7 mg, 10.3 μmol) were used as the acceptor and the donor precursor, respectively, by following the **general procedure for SNRS-NAc**. After being shaken for 41 h, the reaction was quenched and the mixture was purified by following the **general purification procedure 1**. After purification, the product was lyophilized to give compound **45** in quant yield (16.7 mg). R_f = 0.225 (*n*-PrOH/ H_2O/AcOH = 5.5/2/1 (v/v/v)); ^{1}H NMR (850 MHz, D_2O): δ 7.50-7.40 (m, 5H), 5.13 (br, 2H), 5.036 (d, *J* = 3.9 Hz, 1H), 4.89 (q, *J* = 6.7 Hz, 1H), 4.73 (d, *J* = 8.3 Hz),

4.70 (d, J = 8.5 Hz, 1H), 4.65 (d, J = 8.2 Hz, 1H), 4.57 (d, J = 7.7 Hz, 1H), 4.48 (d, J = 7.9 Hz, 1H), 4.47 (d, J = 7.8 Hz, 1H), 4.44 (d, J = 7.9 Hz, 1H), 4.165 (d, J = 3.5 Hz, 1H), 4.157 (d, J = 3.3 Hz, 1H), 4.106 (t, J = 8.8 Hz, 1H), 4.065 (dd, J = 9.9, 3.1 Hz, 1H), 4.02-3.99 (m, 2H), 3.99-3.94 (m, 3H), 3.94-3.83 (m, 12H), 3.825-3.70 (m, 18H), 3.70-3.57 (m, 13H), 3.57-3.54 (m, 2H), 3.528 (dd, J = 9.6, 7.8 Hz, 1H), 3.49 (dd, J = 9.8, 8.7 Hz, 1H), 3.46 (ddd, J = 9.9, 5.2, 2.3 Hz, 1H), 3.32 (t, J = 8.8 Hz, 1H), 3.137 (t, J = 6.4 Hz, 2H), 2.79 (dd, J = 12.4, 4.6 Hz, 1H), 2.07 (s, 3H), 2.056 (s, 6H), 2.049 (s, 3H), 1.789 (t, J = 12.2 Hz, 1H), 1.65-1.60 (m, 2H), 1.506 (quin, J = 7.1 Hz, 2H), 1.40-1.32 (m, 4H), 1.19 (d, J = 6.6 Hz, 3H); ^{13}C NMR (214 MHz, D_2O): δ 175.90 (×2), 175.56, 175.45, 174.89, 159.52, 137.66, 129.74 (×2), 129.26, 128.52 (×2), 104.00, 103.88, 103.81, 103.72, 103.45, 102.96, 101.94, 100.37, 98.96, 82.91, 82.74, 79.96, 79.38, 76.86, 76.62 (×2), 76.60, 76.25, 75.85, 75.71, 75.62, 75.46, 74.54, 74.41, 73.84, 73.72, 73.41, 73.00, 72.91, 72.82, 71.49, 70.97, 70.79, 70.66, 70.08, 69.77, 69.62, 69.45, 69.37, 69.32, 69.31, 69.01, 68.78, 67.91, 67.79, 67.65, 63.30, 62.61, 61.90 (×2), 61.44, 61.00, 60.59, 56.62, 55.94, 52.65, 41.27, 40.97, 29.60, 29.56, 26.43, 25.57, 23.38 (×2), 23.12, 23.00, 16.29; HRMS (ESI) *m/z* calcd for $C_{79}H_{126}N_5O_{50}$ $[M-H]^-$: 1944.7471; found 1944.7472

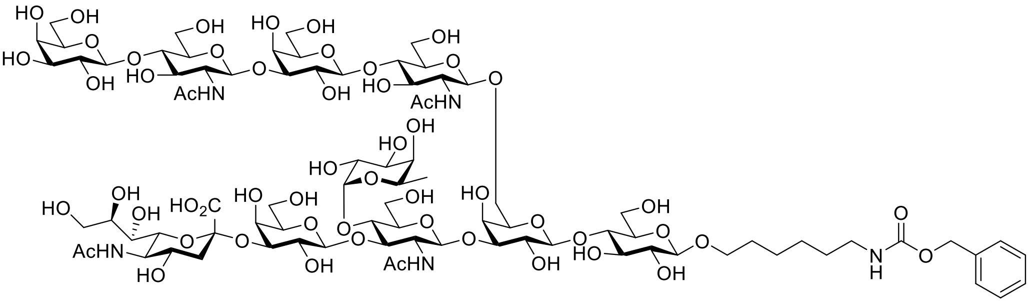

46 (FS-LNO I)

Compound 46 (FS-LNO I). Compound **45** (16 mg, 8.2 µmol) and Gal (1.8 mg, 9.9 µmol) were used as the acceptor and the donor precursor, respectively, by following the **general procedure for SNRS-G4b**. After being shaken for 1 h, the reaction was quenched and the mixture was purified by following **general purification procedure 1**. After purification, the product was lyophilized to give compound **46** (FS-LNO I) in 89% yield (15.4 mg). R_f = 0.16 (n-PrOH/ H_2O/NH_4OH = 6/2/1 (v/v/v)); ^{1}H NMR (850 MHz, D_2O): δ 7.52-7.33 (m, 5H), 5.12 (br, 2H), 5.02 (d, J = 3.8 Hz, 1H), 4.88 (q, J = 6.7 Hz, 1H), 4.71 (d, J = 8.5 Hz, 1H), 4.71 (d, J = 8.4 Hz, 1H), 4.64 (d, J = 8.1 Hz, 1H), 4.56 (d, J = 7.7 Hz), 4.48 (d, J = 7.9 Hz), 4.47 (d, J = 8.2 Hz, 1H), 4.46 (d, J = 7.7 Hz, 1H), 4.43 (d, J = 7.8 Hz, 1H), 4.16 (d, J = 3.1 Hz, 1H), 4.15 (d, J = 3.1 Hz, 1H), 4.09 (t, J = 9.6 Hz, 1H), 4.05 (dd, J = 9.8, 2.9 Hz, 1H), 3.99 (d, J = 10.6 Hz, 1H), 3.99 (d, J = 10.5 Hz, 1H), 3.98-3.92 (m, 5H), 3.92 (d, J = 2.7 Hz, 1H), 3.92-3.82 (m, 10H), 3.82-3.69 (m, 20H), 3.82-3.57 (m, 13H), 3.55 (dd, J = 9.6, 7.9 Hz, 1H), 3.57-3.50 (m, 2H), 3.52 (dd, J = 9.3, 8.1 Hz, 1H), 3.31 (t, J = 8.5 Hz, 1H), 3.13 (t, J = 6.3 Hz, 2H), 2.78 (dd, J = 12.3, 4.4 Hz, 1H), 2.06 (s, 3H), 2.04 (s, 3H), 2.04 (s, 3H), 2.04 (s, 3H),

1.78 (t, *J* = 12.3 Hz, 1H), 1.65-1.58 (m, 2H), 1.49 (quin, *J* = 7.0 Hz, 2H), 1.39-1.29 (m, 4H), 1.18 (d, *J* = 6.6 Hz, 3H); ^{13}C NMR (214 MHz, D_2O): δ 175.90, 175.85, 175.57, 175.45, 174.89, 159.44, 137.66, 129.74 (×2), 129.26, 128.52 (×2), 104.00, 103.89, 103.83, 103.73, 103.71, 103.47, 102.97, 101.94, 100.37, 98.96, 82.99, 82.74, 79.96, 79.36, 79.13, 76.85, 76.60, 76.31 (×2), 76.25, 75.84, 75.72, 75.62, 75.52, 75.45, 74.41, 73.84, 73.72, 73.48 (×2),, 73.41, 73.15, 72.99, 72.94, 72.82, 71.93, 71.49, 70.93, 70.79, 70.08, 69.77, 69.62, 69.52, 69.38, 69.33, 69.30, 69.01, 68.78, 67.91, 67.79, 67.65, 63.30, 62.62, 61.98 (×2), 61.91, 60.99, 60.82, 60.59, 56.80, 56.15 (×2), 55.94, 52.65, 41.27, 40.98, 29.60, 29.56, 26.44, 25.58, 23.38, 23.13, 23.00, 16.29; HRMS (ESI) *m/z* calcd for $C_{85}H_{137}N_5Na_2O_{55}$ [M+2Na]$^+$: 2153.7872; found 2153.7902.

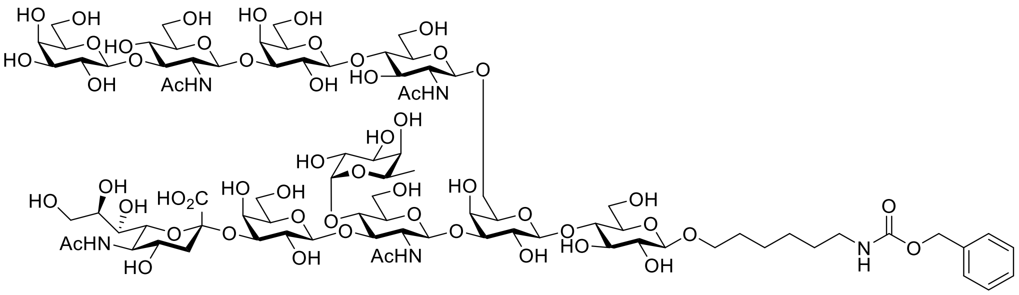

47 (FS-*i*LNO I)

Compound 47 (FS-*i*LNO I). Compound **45** (10 mg, 5.1 µmol) and Gal (1.0 mg, 5.5 µmol) were used as acceptor and donor, respectively, by following the general procedure for SNRS-G3. After being shaken for 3 h, the reaction was quenched and the mixture was purified by following **general purification procedure 1**. After purification, the product was lyophilized to give compound **47** (FS-*i*LNO I) in 98% yield (10.6 mg). R_f = 0.17 (*n*-PrOH/ H_2O/AcOH = 5.5/2/1 (v/v/v)); ^{1}H NMR (850 MHz, D_2O): δ 7.49-7.38 (m, 5H), 5.11 (br, 2H), 5.02 (d, *J* = 3.8 Hz, 1H), 4.88 (q, *J* = 6.5 Hz, 1H), 4.73 (d, *J* = 8.5 Hz), 4.71 (d, *J* = 8.3 Hz, 1H), 4.63 (d, *J* = 8.1 Hz, 1H), 4.55 (d, *J* = 7.7 Hz, 1H), 4.46 (d, *J* = 8.3 Hz, 1H), 4.46 (d, *J* = 7.9 Hz, 1H), 4.45 (d, *J* = 7.7 Hz, 1H), 4.42 (d, *J* = 7.9 Hz, 1H), 4.15 (d, *J* = 3.1 Hz, 1H), 4.14 (d, *J* = 3.0 Hz, 1H), 4.09 (t, *J* = 9.6 Hz, 1H), 4.05 (dd, *J* = 9.8, 2.9 Hz, 1H), 4.01-3.98 (m, 2H), 3.98-3.93 (m, 3H), 3.93-3.87 (m, 5H), 3.87-3.81 (m, 8H), 3.81-3.77 (m, 6H), 3.768-3.69 (m, 13H), 3.69-3.60 (m, 9H), 3.60-3.56 (m, 4H), 3.56-3.50 (m, 3H), 3.516 (dd, *J* = 9.5, 7.9 Hz, 1H), 3.48 (ddd, *J* = 9.8, 5.0, 2.3 Hz, 1H), 3.31 (t, *J* = 8.7 Hz, 1H), 3.12 (t, *J* = 6.3 Hz, 2H), 2.78 (dd, *J* = 12.4, 4.5 Hz, 1H), 2.06 (s, 3H), 2.04 (s, 3H), 2.03 (s, 3H), 2.03 (s, 3H), 1.78 (t, *J* = 12.4 Hz, 1H), 1.65-1.57 (m, 2H), 1.49 (quin, *J* = 7.0 Hz, 2H), 1.39-1.29 (m, 4H), 1.18 (d, *J* = 6.7 Hz, 3H); ^{13}C NMR (214 MHz, D_2O) : δ 175.92, 175.90, 175.56, 175.44, 174.80, 159.43, 137.65, 129.74 (×2), 129.26, 128.52 (×2), 104.45, 103.99, 103.88, 103.72, 103.53, 103.46, 102.96, 101.93, 100.32, 98.96, 83.04, 82.94, 82.74, 79.95, 79.38, 76.86, 76.59, 76.24 (×3), 76.15, 75.85, 75.71 (×2), 75.61, 75.45, 74.40, 73.84, 73.73, 73.43, 73.41, 72.99, 72.91, 72.79, 71.64, 71.49, 70.97, 70.79, 70.08, 69.77, 69.62, 69.49, 69.49, 69.41, 69.33, 69.32, 69.28,

69.01, 68.77, 67.91, 67.78, 67.65, 63.32, 62.61, 61.99 (×2), 61.60, 60.99, 60.59, 56.79, 55.93, 55.65, 52.65, 41.27, 40.93, 29.61, 29.56, 26.44, 25.28, 23.38, 23.19, 23.00, 16.29; HRMS (ESI) *m/z* calcd for $C_{85}H_{136}N_5O_{55}$ [M-H]$^-$: 2106.7999; found 2106.7999.

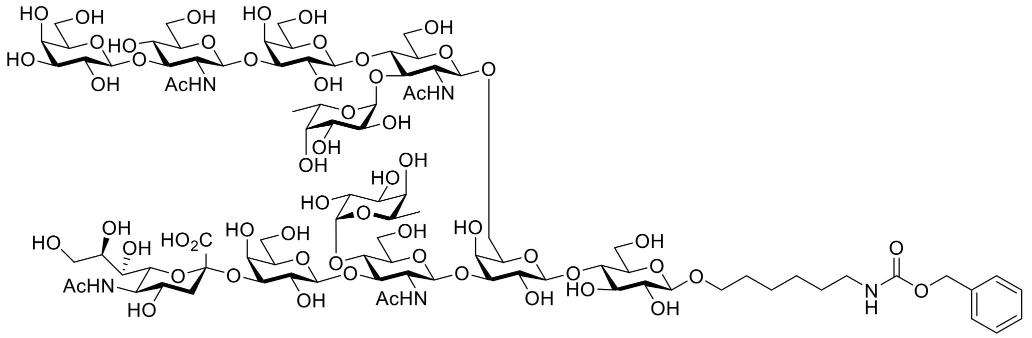

48 (DFS-*i*LNO I)

Compound 48 (DFS-*i*LNO I). Compound **45** (5 mg, 2.6 μmol) and GDP-Fuc (2.4 mg, 3.9 μmol) were used as the acceptor and the donor, respectively, by following the **general procedure for DUSD-F3/4**. After being shaken for 14 h, the reaction was quenched and the mixture was purified by following the **general purification procedure 1**. After purification, the product was lyophilized to give the fucosylated product in quant. yield (5.4 mg). R_f = 0.12 (*n*-PrOH/ H_2O/AcOH = 5/2/1 (v/v/v)). Above fucosylated product (5.4 mg, 2.6 μmol) and Gal (0.6 mg, 3.1 μmol) were used as the acceptor and the donor precursor, respectively, by following the **general procedure for SNRS-G3**. After being shaken for 4 h, the reaction was quenched and the mixture was purified by following the **general purification procedure 1**. After purification, the product was lyophilized to give compound **48** (DFS-*i*LNO I) in two steps 71% yield (4.1 mg). R_f = 0.14 (*n*-PrOH/ H_2O/NH_4OH = 5.5/2/1 (v/v/v)); ^{1}H NMR (850 MHz, D_2O): δ 7.46-7.40 (m, 5H), 5.12 (br, 2H), 5.09 (d, *J* = 4.0 Hz, 1H), 5.02 (d, *J* = 3.8 Hz, 1H), 4.88 (q, *J* = 6.7 Hz, 1H), 4.82 (q, *J* = 6.3 Hz, 1H, overlapping D_2O), 4.73 (d, *J* = 8.5 Hz, 1H), 4.72 (d, *J* = 8.3 Hz, 1H), 4.64 (d, *J* = 7.7 Hz, 1H), 4.56 (d, *J* = 7.7 Hz, 1H), 4.47 (d, *J* = 8.2 Hz, 1H), 4.46 (d, *J* = 8.0 Hz, 1H), 4.44 (d, *J* = 7.7 Hz, 1H), 4.42 (d, *J* = 7.9 Hz, 1H), 4.14 (d, *J* = 3.0 Hz, 1H), 4.103 (d, *J* = 3.3 Hz, 1H), 4.09 (t, *J* = 9.6 Hz, 1H), 4.06 (dd, *J* = 9.8 3.0 Hz, 1H), 4.00-3.98 (m, 2H), 3.98-3.93 (m, 3H), 3.93-3.87 (m, 9H), 3.87-3.77 (m, 12H), 3.77-3.67 (m, 10H), 3.67-3.63 (m, 4H), 3.63-3.56 (m, 7H), 3.55-3.50 (m, 4H), 3.49 (ddd, *J* = 9.9, 5.0, 2.4 Hz, 1H), 3.30 (t, *J* = 8.7 Hz, 1H), 3.12 (t, *J* = 6.4 Hz, 2H), 2.78 (dd, *J* = 12.4, 4.5 Hz, 1H), 2.05 (s, 3H), 2.04 (s, 3H), 2.035 (s, 3H), 2.03 (s, 3H), 1.78 (t, *J* = 12.4 Hz, 1H), 1.63-1.60 (m, 2H), 1.49 (quin, *J* = 7.1 Hz, 2H), 1.38-1.30 (m, 4H), 1.18 (d, *J* = 6.8 Hz, 3H), 1.16 (d, *J* = 6.7 Hz, 3H); ^{13}C NMR (214 MHz, D_2O): δ 175.90, 175.89, 175.56, 175.24, 174.85, 159.45, 137.66, 129.74 (×2), 129.27, 128.52 (×2), 104.43, 104.09, 103.73, 103.48, 103.47, 102.95, 102.77, 101.76, 100.35, 99.70, 98.97, 82.96, 82.73, 82.52, 80.12, 76.86, 76.60, 76.32, 76.24 (×2), 76.12, 75.82, 75.72, 75.62, 75.48, 75.42, 74.34, 74.06, 73.83, 73.73, 73.43, 72.99, 72.92, 72.81 (×2), 71.64, 71.50, 70.79, 70.17, 70.08, 69.77, 69.65, 69.50, 69.39,

69.36, 69.30, 69.20, 69.02, 68.78, 68.63, 67.91, 67.79, 67.68, 67.65, 63.31, 62.62, 62.39, 61.99 (×2), 61.44, 61.01, 60.73, 60.59, 56.80, 56.64, 55.65, 52.65, 41.27, 40.96, 29.61, 29.56, 26.44, 25.58, 23.47, 23.37, 23.17, 23.00, 16.30, 16.23; HRMS (ESI) *m/z* calcd for $C_{91}H_{146}N_5O_{59}$ [M-H]$^-$: 2252.8578; found 2252.8578.

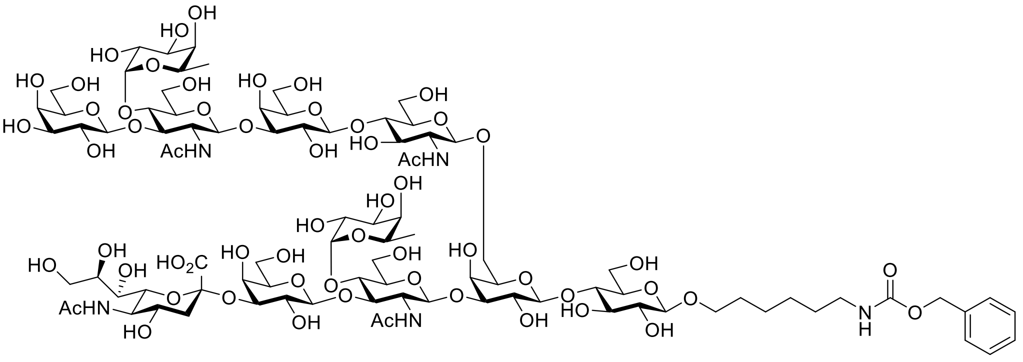

49 (DFS-*i*LNO II)

Compound 49 (DFS-*i*LNO II). Compound **47** (FS-*i*LNO I) (9.6 mg, 4.6 μmol) and GDP-Fuc (4.3 mg, 6.8 μmol) were used as the acceptor and the donor, respectively, by following the **general procedure for DUSD-F3/4**. After being shaken for 6 h, the reaction was quenched and the mixture was purified by following **general purification procedure 2**. After purification, the product was lyophilized to give compound **48** (DFS-*i*LNO I) in 24% yield (2.5 mg), and compound **49** (DFS-*i*LNO II) in 18% yield (1.8 mg). R_f = 0.14 (*n*-PrOH/ H_2O/NH_4OH = 5.5/2/1 (v/v/v)), and TFS-product **50** in 19% yield (2.1 mg). R_f = 0.09 (*n*-PrOH/ H_2O/NH_4OH = 5.5/2/1 (v/v/v)); ^{1}H NMR (850 MHz, D_2O) : δ 7.50-7.37 (m, 5H), 5.12 (br, 2H), 5.03 (d, *J* = 4.0 Hz, 1H), 5.02 (d, *J* = 3.8 Hz, 1H), 4.88 (q, *J* = 6.7 Hz, 1H,), 4.88 (q, *J* = 6.7 Hz, 1H), 4.71 (d, *J* = 7.7 Hz, 1H), 4.71 (d, *J* = 8.2 Hz, 1H), 4.64 (d, *J* = 8.1 Hz, 1H), 4.56 (d, *J* = 7.7 Hz, 1H), 4.51 (d, *J* = 7.7 Hz), 4.47 (d, *J* = 7.7Hz, 1H), 4.46 (d, *J* = 8.0 Hz, 1H), 4.43 (d, *J* = 7.9 Hz, 1H), 4.16 (d, *J* = 3.1 Hz, 1H), 4.16 (d, *J* = 3.1 Hz, 1H), 4.12-4.07 (m, 2H), 4.05 (dd, *J* = 9.8, 3.0 Hz, 1H), 3.99 (d, *J* = 10.2 Hz, 1H), 3.99 (d, *J* = 10.5 Hz, 1H), 3.98-3.93 (m, 4H), 3.92 (d, *J* = 2.8 Hz, 1H), 3.91-3.87 (m, 5H), 3.87-3.85 (m, 3H), 3.85-3.81 (m, 4H), 3.81-3.78 (m, 5H), 3.78-3.69 (m, 15H), 3.69-3.56 (m, 15H), 3.56-3.47 (m, 3H), 3.517 (dd, *J* = 9.6, 7.7 Hz, 1H), 3.493 (dd, *J* = 9.7, 7.9 Hz, 1H), 3.31 (t, *J* = 8.7 Hz, 1H), 3.13 (t, *J* = 6.5 Hz, 2H), 2.78 (dd, *J* = 12.5, 4.6 Hz, 1H), 2.06 (s, 3H), 2.05 (s, 3H), 2.04 (s, 3H), 2.04 (s, 3H), 1.78 (t, *J* = 12.3 Hz, 1H), 1.64-1.59 (m, 2H), 1.50 (quin, *J* = 7.0 Hz, 2H), 1.41-1.26 (m, 4H), 1.33 (d, *J* = 6.9 Hz, 3H), 1.19 (d, *J* = 6.3 Hz, 3H), 1.18 (d, *J* = 6.3 Hz, 3H); ^{13}C NMR (214 MHz, D_2O) : δ 175.91, 175.73, 175.57, 175.43, 174.90, 159.42, 137.66, 129.74 (×2), 129.26, 128.53 (×2), 104.00, 103.90, 103.83, 103.73, 103.61, 103.47, 102.97, 101.94, 100.37, 98.99, 98.97, 83.04, 82.74, 79.96, 79.38, 76.90, 76.86, 76.60, 76.25, 76.19, 75.84, 75.79, 75.72 (×2), 75.62, 75.46, 74.41, 73.84, 73.73, 73.41, 73.29, 73.08, 72.99, 72.92 (×2), 72.83, 71.50, 71.47, 70.93, 70.79, 70.11, 70.08, 69.77, 69.64, 69.45, 69.38, 69.32, 69.28,

69.02, 68.77, 67.91, 67.81, 67.80, 67.65, 63.30, 62.62, 62.61, 61.98, 61.91, 60.99, 60.60, 60.56, 56.83, 56.79, 55.94, 52.65, 41.26, 40.98, 29.60, 29.56, 26.44, 25.58, 23.38, 23.37, 23.25, 23.00, 16.33, 16.29; HRMS (ESI) *m/z* calcd for $C_{91}H_{147}N_5Na_2O_{59}$ $[M+2Na]^+$: 2299.8452; found 2299.8451.

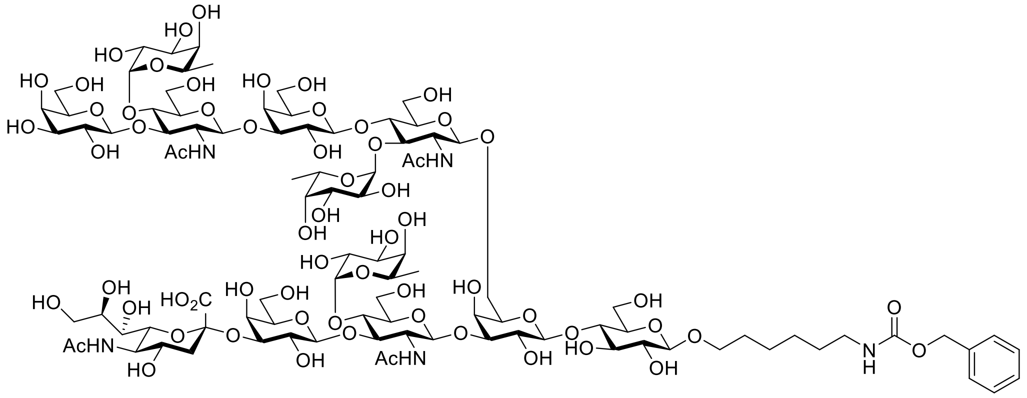

50

Compound 50. ^{1}H NMR (800 MHz, D_2O) : δ 7.47-7.38 (m, 5H), 5.10 (br, 2H), 5.08 (d, *J* = 3.9 Hz, 1H), 5.023 (d, *J* = 3.8 Hz, 1H), 5.01 (d, *J* = 3.7 Hz, 1H), 4.88 (q, *J* = 6.3 Hz, 1H), 4.87 (q, *J* = 6.3 Hz, 1H), 4.80 (q, *J* = 6.3 Hz, 1H, overlapping D_2O), 4.70 (d, *J* = 8.3 Hz, 1H), 4.68 (d, *J* = 8.5 Hz, 1H), 4.63 (d, *J* = 7.7 Hz, 1H), 4.54 (d, *J* = 7.8 Hz, 1H), 4.51 (d, *J* = 7.6 Hz), 4.45 (d, *J* = 7.4 Hz, 1H), 4.42 (d, *J* = 8.3 Hz, 1H), 4.41 (d, *J* = 8.3 Hz, 1H), 4.13 (d, *J* = 2.8 Hz, 1H), 4.10-4.05 (m, 2H), 4.088 (d, *J* = 3.0 Hz, 1H), 4.04 (dd, *J* = 10.0, 2.9 Hz, 1H), 4.00-3.91 (m, 8H), 3.91-3.82 (m, 14H), 3.82-3.76 (m, 9H), 3.76-3.71 (m, 5H), 3.71-3.59 (m, 14H), 3.59-3.54 (m, 6H), 3.54-3.504 (m, 4H), 3.50 (d, *J* = 8.4 Hz, 1H), 3.48 (dd, *J* = 9.6, 8.1 Hz, 1H), 3.29 (t, *J* = 8.7 Hz, 1H), 3.11 (t, *J* = 6.4 Hz, 2H), 2.76 (dd, *J* = 12.4, 4.5 Hz, 1H), 2.04 (s, 3H), 2.03 (s, 3H), 2.02 (s, 6H), 1.76 (t, *J* = 12.2 Hz, 1H), 1.63-1.57 (m, 2H), 1.48 (quin, *J* = 7.0 Hz, 2H), 1.38-1.28 (m, 4H), 1.172 (d, *J* = 6.4 Hz, 3H), 1.168 (d, *J* = 6.4 Hz, 3H), 1.14 (d, *J* = 6.5 Hz, 3H); ^{13}C NMR (201 MHz, D_2O) : δ 175.911, 175.70, 175.57, 175.26, 174.91 (x2), 159.47, 137.66, 129.75 (×2), 129.27, 128.53 (×2), 104.10, 103.83, 103.74, 103.57, 103.48, 102.95, 102.80, 101.78, 100.36, 99.71, 98.98 (×2), 82.73, 82.61, 80.12, 76.90, 76.86, 76.60, 76.32, 76.24, 76.15, 75.81, 75.78, 75.73, 75.63, 75.48, 75.42, 74.34, 74.06, 73.84, 73.73, 73.28, 73.07, 72.99, 72.92 (×2), 72.84, 72.82, 71.51, 71.47, 71.46, 70.81, 70.79, 70.17, 70.10, 70.08, 69.77, 69.65, 69.39, 69.33, 69.31, 69.21, 69.01, 68.78, 68.77, 68.62, 67.91, 67.81, 67.69, 67.66, 63.30, 62.64, 62.62, 62.41, 61.01, 60.72, 60.59, 60.56, 56.83, 56.65, 52.65, 41.27, 40.98, 29.62, 29.57, 26.45, 25.58, 23.47, 23.37, 23.24, 23.00, 16.33, 16.30, 16.23; HRMS (ESI) *m/z* calcd for $C_{97}H_{157}N_5NaO_{63}$ $[M+Na]^+$: 2422.9133; found 2422.9099.

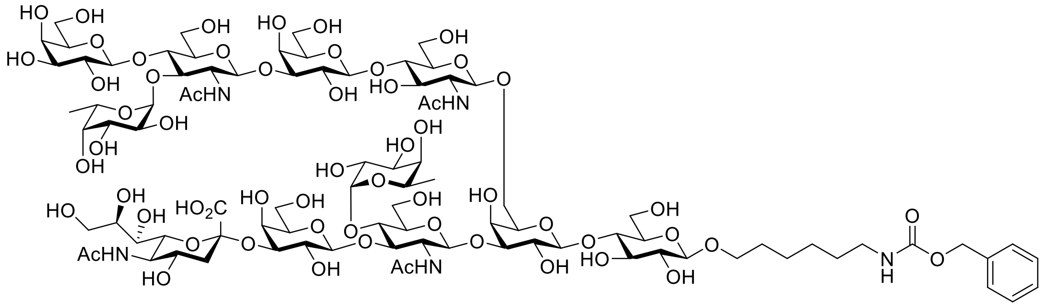

Compound 51 (DFS-LNO I). Compound **46** (FS-LNO I) (10.6 mg, 5.0 µmol) and GDP-Fuc (4.8 mg, 7.5 µmol) were used as the acceptor and the donor, respectively, by following the **general procedure for DUSD-F3/4**. After being shaken for 10 h, the reaction was quenched and the mixture was purified by following the **general purification procedure 1**. After purification, the product was lyophilized to give a 1:1 mixture containing compound **51** (DFS-LNO I) and compound **52** in 49% yield (5.5 mg, brsm = 52%, R_f = 0.13 (*n*-PrOH/ H_2O/NH_4OH = 5.5/2/1 (v/v/v)), and TFS-product **54** in 18% yield (1.8 mg, brsm = 20%, R_f = 0.10 (*n*-PrOH/ H_2O/NH_4OH = 5.5/2/1 (v/v/v)); ^{1}H NMR (850 MHz, D_2O): δ 7.48-7.40 (m, 5H), 5.14 (d, *J* = 3.9 Hz, 1H), 5.12 (br, 2H), 5.028 (d, *J* = 3.8 Hz, 1H), 4.88 (q, *J* = 6.7 Hz, 1H), 4.85 (q, *J* = 6.7 Hz, 1H), 4.722 (d, *J* = 8.1 Hz, 1H), 4.718 (d, *J* = 7.7 Hz, 1H), 4.64 (d, *J* = 8.1 Hz, 1H), 4.56 (d, *J* = 7.7 Hz), 4.47 (d, *J* = 7.7 Hz, 2H), 4.46 (d, *J* = 8.5 Hz, 1H), 4.43 (d, *J* = 7.9 Hz, 1H), 4.16 (d, *J* = 3.1 Hz, 1H), 4.15 (d, *J* = 2.9 Hz, 1H), 4.10 (t, *J* = 9.7 Hz, 1H), 4.06 (dd, *J* = 9.8, 2.8 Hz, 1H), 4.02-3.94 (m, 8H), 3.93-3.82 (m, 14H), 3.82-3.74 (m, 9H), 3.74-3.69 (m, 9H), 3.69-3.61 (m, 8H),3.61-3.57 (m, 6H), 3.55 (dd, *J* = 10.1, 3.5 Hz, 1H), 3.543 (t, *J* = 6.6 Hz, 1H), 3.52 (dd, *J* = 9.8, 7.9 Hz, 1H), 3.509 (dd, *J* = 9.9, 7.9 Hz, 1H), 3.31 (t, *J* = 8.5 Hz, 1H), 3.13 (t, *J* = 6.3 Hz, 2H), 2.78 (dd, *J* = 12.3, 4.4 Hz, 1H), 2.06 (s, 3H), 2.048 (s, 3H), 2.04 (s, 3H), 2.035 (s, 3H), 1.78 (t, *J* = 12.4 Hz, 1H), 1.65-1.58 (m, 2H), 1.50 (quin, *J* = 7.1 Hz, 2H), 1.39-1.30 (m, 4H), 1.187 (d, *J* = 6.7 Hz, 6H); ^{13}C NMR (214 MHz, D_2O): δ175.91, 175.66, 175.57, 175.46, 174.90, 159.46, 137.67, 129.74 (×2), 129.27, 128.52 (×2), 104.01, 103.89, 103.73, 103.52, 103.47, 102.97, 102.75, 101.94, 100.37, 99.57, 98.97, 83.02, 82.75, 79.97, 79.35, 76.86, 76.60, 76.25, 76.09, 75.88, 75.83, 75.72 (×2), 75.62, 75.46, 74.41, 74.02, 73.84, 73.73, 73.45, 73.42, 73.00, 72.92, 72.88, 72.83, 72.02, 71.50, 71.11, 70.93, 70.79, 70.17, 70.08, 69.77, 69.64, 69.38, 69.32 (×2), 69.28, 69.02, 68.78, 68.68, 67.91, 67.80, 67.66 (x2), 63.30, 62.62, 62.46, 61.98 (×2), 61.91, 61.00, 60.60, 56.92, 55.94 (×2), 52.65, 41.27, 40.98, 29.60, 29.56, 26.44, 25.58, 23.38, 23.37, 23.21, 23.00, 16.30, 16.27; HRMS (ESI) *m/z* calcd for $C_{91}H_{146}N_5O_{59}$ $[M-H]^-$: 2252.8578; found 2252.8567

Hydrolysis of compound 51 (DFS-LNO I) and compound 52 by β-galactosidase (*Aspergillus oryzae*). The pH of the buffer solution (100 mM Tris-HCl) containing 20 mM $MgCl_2$ was adjust to 7-7.5 by adding 4N $HCl_{(aq)}$. To the above solution were added 3 mM of a

difucosylated mixture (compound **51** (DFS-LNO I) and compound **52**) and 0.5 U/mL of β-galactosidase. The solution was incubated at 30 ^{o}C. The reaction progression was monitored by TLC analysis and stained with *para*-anisaldehyde stain. More enzymes were added if necessary. After being shaken for 24 h, the reaction was quenched and the mixture was purified by following **general purification procedure 1**. The fractions containing product were lyophilized to give compound **51** (DFS-LNO I) in 20% yield (2.3 mg, R_f = 0.15 (*n*-PrOH/ H_2O/NH_4OH = 5.5/2/1 (v/v/v)) and compound **53** in 25% yield (2.6 mg, R_f = 0.25 (*n*-PrOH/ H_2O/NH_4OH = 5.5/2/1 (v/v/v)).

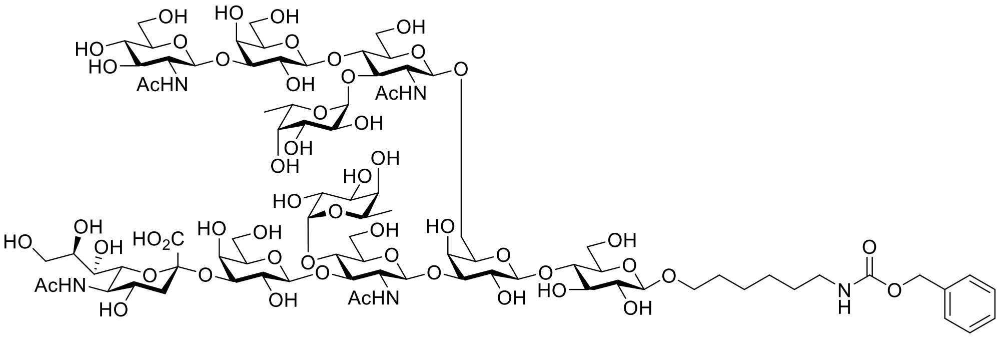

53

Compound 53. ^{1}H NMR (850 MHz, D_2O): δ 7.49-7.40 (m, 5H), 5.13 (br, 2H), 5.106 (d, *J* = 3.9 Hz, 1H), 5.036 (d, *J* = 3.9 Hz, 1H), 4.89 (q, *J* = 6.5 Hz, 1H), 4.83(q, *J* = 6.7 Hz, 1H), 4.725 (d, *J* = 8.2 Hz, 1H), 4.70 (d, *J* = 8.4 Hz, 1H), 4.65 (d, *J* = 7.4 Hz, 1H), 4.57 (d, *J* = 7.7 Hz, 1H), 4.48 (d, *J* = 8.0 Hz, 1H), 4.45 (d, *J* = 8.0 Hz, 1H), 4.44 (d, *J* = 7.9 Hz, 1H), 4.153 (d, *J* = 3.1 Hz, 1H), 4.11 (d, *J* = 3.1 Hz, 1H), 4.107 (t, *J* = 9.7 Hz, 1H), 4.066 (dd, *J* = 9.7, 2.8 Hz, 1H), 4.03-3.94 (m, 6H), 3.94-3.83 (m, 14H), 3.83-3.745 (m, 8H), 3.744-3.69 (m, 7H), 3.685-3.64 (m, 4H), 3.64-3.57 (m, 7H), 3.57-3.51 (m, 4H), 3.49 (dd, *J* = 9.5, 9.0 Hz, 1H), 3.456 (ddd, *J* = 9.7, 4.8, 2.0 Hz, 1H), 3.317 (t, *J* = 8.8 Hz, 1H), 3.138 (t, *J* = 6.5 Hz, 2H), 2.79 (dd, *J* = 12.5, 4.5 Hz, 1H), 2.063 (s, 3H), 2.056 (s,3H), 2.049 (s, 6H), 1.79 (t, *J* = 12.2 Hz, 1H), 1.65-1.60 (m, 2H), 1.506 (quin, *J* = 7.1 Hz, 2H), 1.40-1.31 (m, 4H), 1.19 (d, *J* = 6.5 Hz, 3H), 1.17 (d, *J* = 6.5 Hz, 3H); ^{13}C NMR (201 MHz, D_2O): δ 175.91, 175.87, 175.57, 175.26, 174.90, 159.47, 137.66, 129.75 (×2), 129.27, 128.53 (×2), 104.10, 103.78, 103.74, 103.48, 102.95, 102.79, 101.78, 100.36, 99.71, 98.98, 82.73, 82.47, 80.12, 76.59 (×2), 76.32, 76.25, 75.73, 75.63, 75.48, 75.42, 74.48 (×2), 74.34, 74.05, 73.84, 73.73, 72.99, 72.92, 72.83, 72.83, 71.51 (x2), 70.79, 70.67, 70.17, 70.08, 69.77, 69.65, 69.39 (×2), 69.31, 69.25, 69.01, 68.78, 68.63, 67.91, 67.80, 67.69, 67.66, 63.30, 62.64, 62.40, 61.42, 61.00, 60.73, 60.59, 56.80, 56.60 (x2), 52.65, 49.32, 49.22, 49.11, 49.00, 48.89, 48.78, 48.68, 41.27, 40.98, 29.61, 29.57, 26.45, 25.58, 23.47, 23.37, 23.11, 23.00, 16.30, 16.24; HRMS (ESI) *m/z* calcd for $C_{85}H_{137}N_5Na_1O_{54}$ $[M+Na]^+$: 2114.8026; found 2114.8038.

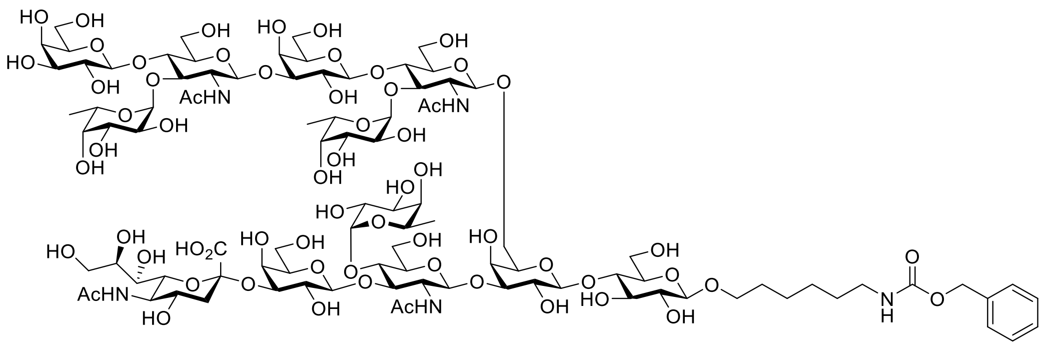

54

Compound 54. ^{1}H NMR (850 MHz, D_2O): δ 7.49-7.40 (m, 5H), 5.154 (d, J = 3.9 Hz, 1H), 5.13 (br, 2H), 5.105 (d, J = 3.9 Hz, 1H), 5.036 (d, J = 3.7 Hz, 1H), 4.89 (q, J = 6.7 Hz, 1H), 4.85 (q, J = 6.7 Hz, 1H), 4.82 (q, J = 6.7 Hz, 1H), 4.72 (d, J = 8.2 Hz, 2H), 4.65 (d, J = 7.3 Hz, 1H), 4.57 (d, J = 7.7 Hz, 1H), 4.48 (d, J = 7.7 Hz, 2H), 4.45 (d, J = 8.5 Hz, 1H), 4.44 (d, J = 8.5 Hz, 1H), 4.152 (d, J = 2.9 Hz, 1H), 4.11 (d, J = 3.1 Hz, 1H), 4.10 (t, J = 9.9 Hz, 1H), 4.066 (dd, J = 9.8, 2.9 Hz, 1H), 4.03-3.94 (m, 9H), 3.94-3.83 (m, 17H), 3.825-3.745 (m, 8H), 3.744-3.686 (m, 9H), 3.685-3.62 (m, 7H), 3.62-3.58 (m, 6H), 3.57-3.50 (m, 5H), 3.316 (t, J = 8.7 Hz, 1H), 3.137 (t, J = 6.2 Hz, 2H), 2.79 (dd, J = 12.5, 4.4 Hz, 1H), 2.062 (s, 3H), 2.056 (s, 3H), 2.048 (s, 3H), 2.037 (s, 3H), 1.79 (t, J = 12.2 Hz, 1H), 1.65-1.60 (m, 2H), 1.505 (quin, J = 7.1 Hz, 2H), 1.40-1.31 (m, 4H), 1.19 (d, J = 6.5 Hz, 6H), 1.16 (d, J = 6.5 Hz, 3H); ^{13}C NMR (201 MHz, D_2O): δ 175.90 , 175.62, 175.57, 175.25, 174.91, 159.45, 137.66, 129.75 (×2), 129.27, 128.53 (×2), 104.10, 103.74, 103.48 (×2), 102.95, 102.79, 102.72, 101.77, 100.36, 99.71, 99.56, 98.98, 82.73, 82.56, 80.12, 76.87, 76.60, 76.31, 76.24, 76.04, 75.88, 75.82, 75.73, 75.63, 75.48, 75.41, 74.33, 74.02, 73.99, 73.83, 73.72, 73.44, 72.99, 72.92, 72.88, 72.83, 72.82, 72.02, 71.51, 71.48, 70.79, 70.17 (×2), 70.08, 69.77, 69.64, 69.45, 69.39, 69.32, 69.30, 69.22, 69.01, 68.78, 68.68, 68.62, 67.91, 67.80, 67.68, 67.66 (×2), 63.30, 62.63, 62.47, 62.41, 61.00, 60.72, 60.59 (×2), 56.91, 56.79, 56.63, 52.65, 41.27, 40.98, 29.62, 29.57, 26.45, 25.58, 23.47, 23.37, 23.20, 23.00, 16.30, 16.27, 16.23; HRMS (ESI) *m/z* calcd for $C_{97}H_{157}N_5Na_2O_{63}$ $[M+2Na]^{+}$: 2445.9031; found 2445.9032.

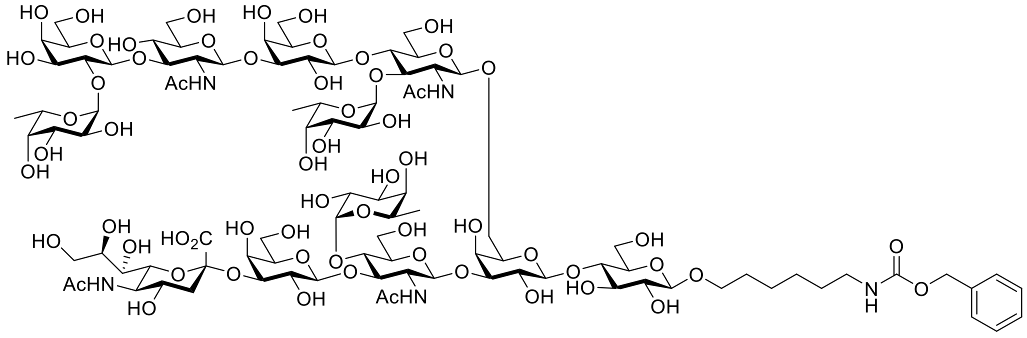

55 (TFS-*i*LNO)

Compound 55 (TFS-*i*LNO). Compound **48** (DFS-*i*LNO I) (3.0 mg, 1.3 μmol) and GDP-Fuc (1.3 mg, 2.0 μmol) were used as the acceptor and the donor, respectively, by following the

general procedure for DUSD-F2. After being shaken for 28 h, the reaction was quenched and the mixture was purified by following the **general purification procedure 1**. After purification, the product was lyophilized to give compound **48** (TFS-*i*LNO) in quant yield (3.2 mg). R_f = 0.1 (*n*-PrOH/ H_2O/25% NH_4OH = 5.5/2/1 (v/v/v)); ^1H NMR (850 MHz, D_2O): δ 7.51-7.36 (m, 5H), 5.20 (d, *J* = 4.1 Hz, 1H), 5.12 (br, 2H), 5.09 (d, *J* = 4.0 Hz, 1H), 5.02 (d, *J* = 3.8 Hz, 1H), 4.88 (q, *J* = 6.7 Hz, 1H), 4.82 (q, *J* = 6.7 Hz, 1H, overlapping D_2O), 4.71 (d, *J* = 8.2 Hz, 1H), 4.65 (d, *J* = 7.7 Hz, 1H), 4.64 (d, *J* = 8.2 Hz, 1H), 4.63 (d, *J* = 8.5 Hz, 1H), 4.56 (d, *J* = 7.7 Hz, 1H), 4.47 (d, *J* = 7.9 Hz, 1H), 4.43 (d, *J* = 7.7 Hz, 2H), 4.30 (q, *J* = 6.7 Hz, 1H), 4.14 (d, *J* = 3.1 Hz, 1H), 4.095 (t, *J* = 9.4 Hz, 1H), 4.086 (d, *J* = 3.2 Hz, 1H), 4.05 (dd, *J* = 9.8, 3.0 Hz, 1H), 4.02-3.98 (m, 3H), 3.98-3.93 (m, 3H), 3.93-3.87 (m, 9H), 3.87-3.81 (m, 8H), 3.81-3.75 (m, 10H), 3.75-3.67 (m, 11H), 3.67-3.63 (m, 5H), 3.63-3.57 (m, 8H), 3.57-3.51 (m, 4H), 3.51-3.47 (m, 2H), 3.30 (t, *J* = 8.8 Hz, 1H), 3.13 (t, *J* = 6.4 Hz, 2H), 2.78 (dd, *J* = 12.6, 4.6 Hz, 1H), 2.06 (s, 3H), 2.05 (s, 3H), 2.04 (s, 3H), 2.04 (s, 3H), 1.78 (t, *J* = 12.1 Hz, 1H), 1.64-1.58 (m, 2H), 1.49 (quin, *J* = 7.0 Hz, 2H), 1.39-1.28 (m, 4H), 1.33 (d, *J* = 6.9 Hz, 3H), 1.24 (d, *J* = 6.6 Hz, 3H), 1.18 (d, *J* = 6.5 Hz, 3H), 1.15 (d, *J* = 6.6 Hz, 3H); ^{13}C NMR (214 MHz, D_2O): δ 175.91, 175.57, 175.26, 175.17, 174.89, 159.45, 137.66, 129.74 (×2), 129.27, 128.52 (×2), 104.19, 104.09, 103.73, 103.46, 102.95, 102.76, 101.77, 101.21, 100.48, 100.37, 99.79, 98.97, 82.72, 82.19, 80.14, 79.14, 77.63, 76.86, 76.60, 76.33, 76.24, 76.16, 76.03, 75.92, 75.72, 75.62, 75.48, 75.33, 74.45, 74.32, 73.89, 72.83, 73.72, 73.00, 72.91, 72.83, 71.74, 71.50, 70.79, 70.38, 70.18, 70.09, 69.77, 69.65, 69.48, 69.45, 69.43, 69.38, 69.29, 69.01, 68.93, 68.78, 68.60, 67.91, 67.79, 67.67, 67.65, 67.43, 63.30, 62.62, 62.40, 62.11, 61.97, 61.34, 61.01, 60.73, 60.60, 56.80, 56.64, 56.00, 52.65, 41.26, 40.98, 29.61, 29.56, 26.44, 25.58, 23.47, 23.37, 23.10, 23.00, 21.00, 16.29, 16.25; HRMS (ESI) *m/z* calcd for $C_{97}H_{157}N_5O_{63}$ $[M]^+$: 2445.9031; found 2445.9032.

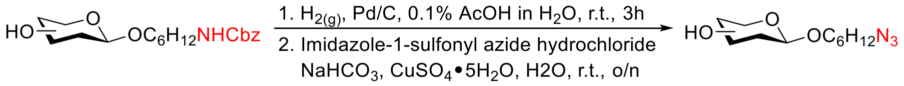

Scheme S4. General Procedure for conversion NHCbz to Azide

General Procedure for conversion NHCbz to Azide. The reaction solvent (H_2O with 0.1% AcOH, pH 3.25) was inflated with H_2(g). The above solvent was then introduced into a 1.5 mL microcentrifuge tubes which contain a compound with a Cbz protecting group, a stir bar, and a moderate amount of 10% Pd/C. The reaction tubes placed into a reaction chamber and stirred under one H_2 atmosphere for 3 hours. The resulting solution was filtered through celite and subjected to lyophilization to yield HMOs within the amino hexyl aglycon.

To an aqueous solution containing $NaHCO_3$ (5.0 eq., 125 mM) and imidazole-1-sulfonyl azide hydrochloride (3.0 eq., 75 mM) was added the amine intermediate (final concentration: 25 mM). Then, $CuSO_4{\cdot}5H_2O$ (0.1 eq., 2.5 mM) was added to the above solution and the

resulting solution was stirred at 500 rpm for overnight. Once the amine compound was fully consumed (checked by TLC), the resulting solution underwent purification using a C18 SPE column (Sep-Pak Vac C18 cartridge 1 cc/50 mg, 55-105 µm, Waters). The elution was performed stepwise with 100 µL H_2O with 0.1% AcOH, 200 µL H_2O, and 300 µL MeOH-H_2O co-solvent containing 30%, 50%, 75%, and 100% MeOH, respectively. Fractions containing the product were combined, and then concentrated using a centrifuge concentrator (Vacufuge plus, Eppendorf) followed by lyophilization. The yield and ESI-MS characterization of each glycan was shown in Table S2.

Table S2. The synthetic yields and m/z ESI-MA data of azido functionalized branched HMOs.

HMO	Weight of Cbz protected HMO	Weight of azido functionalized HMO	Yield	m/z calcd	m/z founded
**FLNH1**	1.5 mg	1.2 mg	85%	$[M+Na]^+$ 1366.5236	1366.5227
**5130c**	1.4 mg	1.0 mg	75%	$[M+Na]^+$ 1731.6558	1731.6566
**DF-LNHc**	2.7 mg	2.5 mg	quant.	$[M-H]^-$ 1488.5839	1488.5830
**TF-LNH**	3.0 mg	2.8 mg	quant.	$[M-H]^-$ 1634.6418	1634.6422
**DF-iLNO IV**	3.0 mg	2.4 mg	85%	$[M+Na]^+$ 1877.7137	1877.7136
**5330a**	1.6 mg	1.4 mg	92%	$[M+Na]^+$ 2023.7716	2023.7707
**TetraF-iLNO**	3.0 mg	2.7 mg	94%	$[M+Na]^+$ 2169.8295	2169.8295
**PentaF-iLNO**	3.0 mg	2.9 mg	quant.	$[M+Na]^+$ 2315.8874	2315.8875
**5131a**	1.0 mg	0.5 mg	53%	$[M-H+2Na]^+$ 2045.7410	2045.7410
**5231a**	2.0 mg	1.7 mg	89%	$[M-H+2Na]^+$ 2190.7910	2190.7910
**5231b**	1.0 mg	0.7 mg	74%	$[M-H+2Na]^+$ 2190.7910	2190.7911
**5331a**	3.0 mg	2.4 mg	84%	$[M-H+3Na]^{2+}$ 1180.9272	1180.9247
**FS-LNH IV**	3.0 mg	2.7 mg	96%	$[M-H+2Na]^+$ 1679.6009	1679.6009
**FS-iLNO I**	0.5 mg	0.4 mg	85%	$[M-H+2Na]^+$ 2045.7410	2045.7407
**DFS-iLNO I**	0.5 mg	0.5 mg	quant.	$[M-H+2Na]^+$ 2190.7910	2190.7866
**DFS-iLNO II**	0.7 mg	0.6 mg	90%	$[M-H+2Na]^+$ 2190.7910	2190.7912
**TFS-iLNO**	1.6 mg	1.2 mg	78%	$[M-H+2Na]^+$ 2336.8490	2336.8488
**FS-LNO I**	0.7 mg	0.6 mg	90%	$[M-H+2Na]^+$ 2044.7331	2044.7320

Fabrication of Glycan microarray. The alkynated slide was prepared by incubating Cu_2O@Ag glass slide[22] with alkynylated thiol linker HS-PEG-Alkyne, MPEG spacer, and zwitterionic linker (their structures are shown below) in the ratio of 49.5:49.5:1.0 mol%. The azide functionalized branched HMOs and control glycans (their structures are shown in Figure S14) (500 µM, in ddH_2O with 10% DMSO and 10 % glycerol) subsequently deposited onto the alkyne-functionalized slide using a robotic contact arrayer (AD 1500 Arrayer, biodot) with a drop volume of approximately 5-6 nL under a relative humidity of 88% and at 26 °C. The slides were then incubated for 20 hours in an environment with 70% relative humidity at 26 °C followed by rinsing with deionized water and dried by centrifugation.

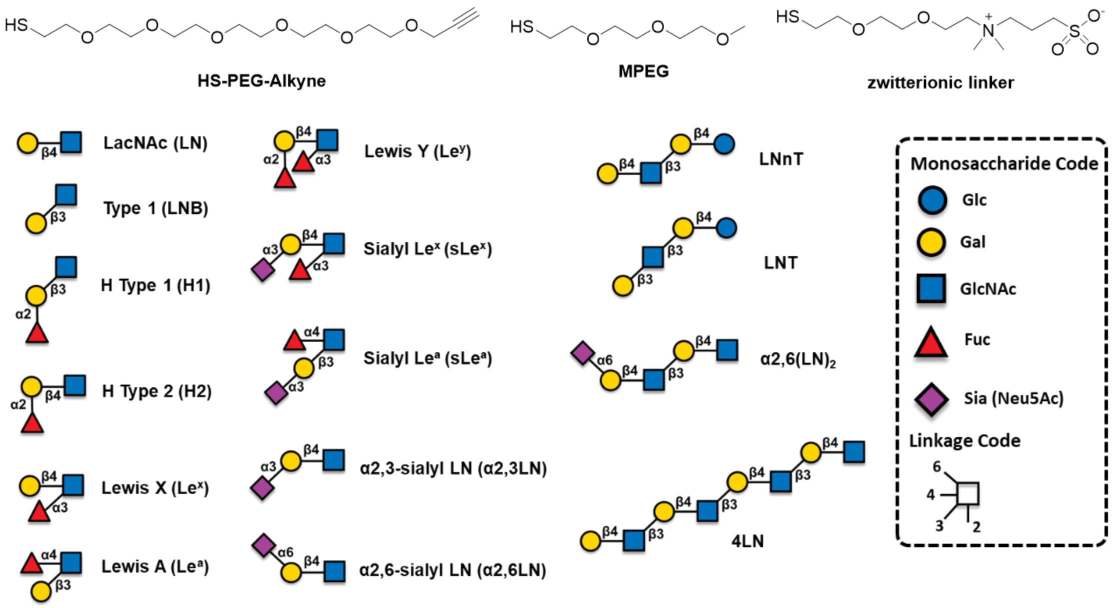

Figure S14. Additional glycan structures on glycan microarray.

Glycan microarray binding assay with GBPs.

Protein Materials. His tagged human galectins, DC-SIGN, and viral hemagglutinin (HA) were purchased from Sino Biological Inc. and ACROBiosystems. All of them were diluted into certain concentration, divided into aliquots, and stored at -80 °C as stock solutions by following the manufacture instructions. Cy3 conjugated mouse anti-6X His Tag Antibody was purchased from Rockland Immunochemicals.

The slides were rehydrated for 5 min in TSMW buffer (20 mM Tris-HCl, 150 mM NaCl, 2 mM $CaCl_2$, and 2 mM $MgCl_2$, 0.05% Tween-20, pH 7.4). Then, diluted galectins, DC-SIGN, and HA with various concentrations of proteins in 0.1 mL of TSMB buffer (20 mM Tris-HCl, 150 mM NaCl, 2 mM $CaCl_2$, and 2 mM $MgCl_2$, 0.05% Tween-20, 1% BSA, pH 7.4) were incubated respectively, with each subarray for 1 hour. It should be noted that 1 mM of TCEP (final conc.) was added to TSMB buffer for galectin binding due to the sensitivity of the galectin to be oxidative inactivation.[23] Subsequently, each subarrary was

washed with 0.1 mL of TSMW buffer and TSM buffer (20 mM Tris-HCl, 150 mM NaCl, 2 mM $CaCl_2$, and 2 mM $MgCl_2$, pH 7.4). 0.1 mL of Cy3 conjugated mouse anti-6X His Tag Antibody (5 μg/mL) was then added and incubated for 1 hour and washed as described above. The sides were subsequently washed by ddH_2O and dried by centrifugation and immediately scanned for fluorescence by SpinScan Microarray Scanner HC-BS01 (Caduceus Biotechnology Inc.). The fluorescence intensities of the spots were quantify using Image Lab software 6.0.1. The spot intensities subtracted background intensities to give corrected intensities which were further analyzed by using Microsoft excel. After removing the data of the lowest and highest values of the ten replicates, the mean fluorescent intensities and standard deviations (SD) were calculated (n=8) (Table S3). The data were graphically represented in bar charts using GraphPad Prism 9. Bar graphs represent the mean ± SD for each compound (Figure S15).

Table S3. Microarray Data. [a]Not assayed.

	hGal-1				hGal-3				hGal-7				DC-SIGN				HA of H1N1			
	25 µg/mL		12.5 µg/mL		25 µg/mL		12.5 µg/mL		25 µg/mL		12.5 µg/mL		20 µg/mL		10 µg/mL		50 µg/mL		25 µg/mL	
Glycan	Mean	SD	Mean	SD	Mean	SD	Mean	SD	Mean	SD	Mean	SD	Mean	SD	Mean	SD	Mean	SD	Mean	SD
LacNAc	401	29	297	16	732	58	196	16	212	24	196	16	708	329	114	37	0	0	0	0
LNB	643	131	262	33	1181	173	257	38	2548	618	257	38	1106	211	312	89	-	-	-	-
H1	302	76	145	35	1319	217	277	41	2174	509	277	41	719	231	281	83	-	-	-	-
H2	5	15	16	5	340	72	83	18	0	0	83	18	1294	415	363	137	-	-	-	-
Le^x	90	25	51	13	217	60	190	25	241	28	190	25	23625	4696	4900	1955	0	0	0	0
Le^a	108	16	62	21	170	70	116	25	257	45	116	25	23160	4382	4216	403	0	0	0	0
Le^y	156	28	59	21	134	73	152	52	228	81	152	52	25180	1767	5516	1227	0	0	0	0
α2,3LN	0	0	27	6	388	82	12	22	0	0	12	22	0	0	0	0	736	139	872	151
α2,6LN	0	0	1	10	-2	38	94	40	0	0	94	40	0	0	0	0	1694	242	2666	400
sialylLe^x	-[a]	-	-	-	-	-	-	-	-	-	-	-	-	-	-	-	663	94	704	232
sialylLe^a	0	0	0	0	20	148	14	50	0	0	14	50	5385	707	2826	621	552	80	825	76
LNnT	456	59	411	43	1610	204	478	80	1283	283	478	80	68	41	0	0	0	0	0	0
LNT	964	208	667	47	983	86	281	44	5949	675	281	44	530	240	300	30	0	0	0	0
4LN	409	70	323	14	7042	514	2049	168	1176	243	2049	168	-	-	-	-	-	-	-	-
α2,6(LN)$_2$	-	-	-	-	-	-	-	-	-	-	-	-	-	-	-	-	3207	595	6105	450
FLNH1 (20)	796	183	489	49	800	39	191	11	6137	860	191	11	436	70	52	22	0	0	0	0
5130c (22)	941	203	503	33	2160	178	625	86	5537	643	625	86	273	144	22	20	0	0	0	0
DF-LNHc (24)	241	52	102	7	190	92	19	14	0	0	19	14	9134	1689	2325	486	0	0	0	0
TF-LNH (25)	99	10	54	3	484	145	123	24	0	0	123	24	23143	2394	3676	843	0	0	0	0
DF-iLNO IV (26)	419	103	242	18	971	189	129	17	2529	504	129	17	4044	855	1072	228	0	0	0	0
5330a (27)	516	137	264	17	1142	92	118	18	3185	551	118	18	3310	753	855	144	0	0	0	0
TetraF-iLNO (32)	482	69	260	12	354	189	32	21	3124	320	32	21	3859	753	1026	340	0	0	0	0
PentaF-iLNO (28	82	17	42	6	404	122	146	38	0	0	146	38	16579	1764	3815	622	0	0	0	0
5131a (39)	142	11	65	9	1621	143	81	17	3059	775	81	17	266	95	0	0	2682	450	3229	453
5231a (34)	91	24	61	5	125	71	0	19	2079	649	0	19	844	146	332	132	3115	268	3571	675
5231b (42)	0	0	0	10	689	105	4	18	0	0	4	18	3080	407	1051	444	3320	484	3463	796
5331a (36)	0	0	0	5	15	104	37	20	0	0	37	20	3905	508	2176	627	3141	597	3761	1193
FS-LNH IV (44)	0	0	17	12	0	63	6	51	0	0	6	51	4494	672	1194	135	952	195	984	225
FS-iLNO I (47)	170	59	96	18	476	98	0	29	1003	379	0	29	4272	964	871	150	509	211	601	129
DFS-iLNO I (48)	108	23	93	14	-3	86	19	27	1457	527	19	27	7061	1039	1648	282	516	158	667	274
TFS-iLNO (55)	155	34	109	21	17	77	1	45	2075	139	1	45	4179	889	1123	114	531	198	644	142
DFS-iLNO II (49)	0	0	0	1	638	148	35	34	0	0	35	34	25309	1307	3310	203	424	144	697	217
FS-LNO I (46)	117	21	109	16	836	111	71	26	0	0	71	26	2940	417	874	59	646	67	898	202

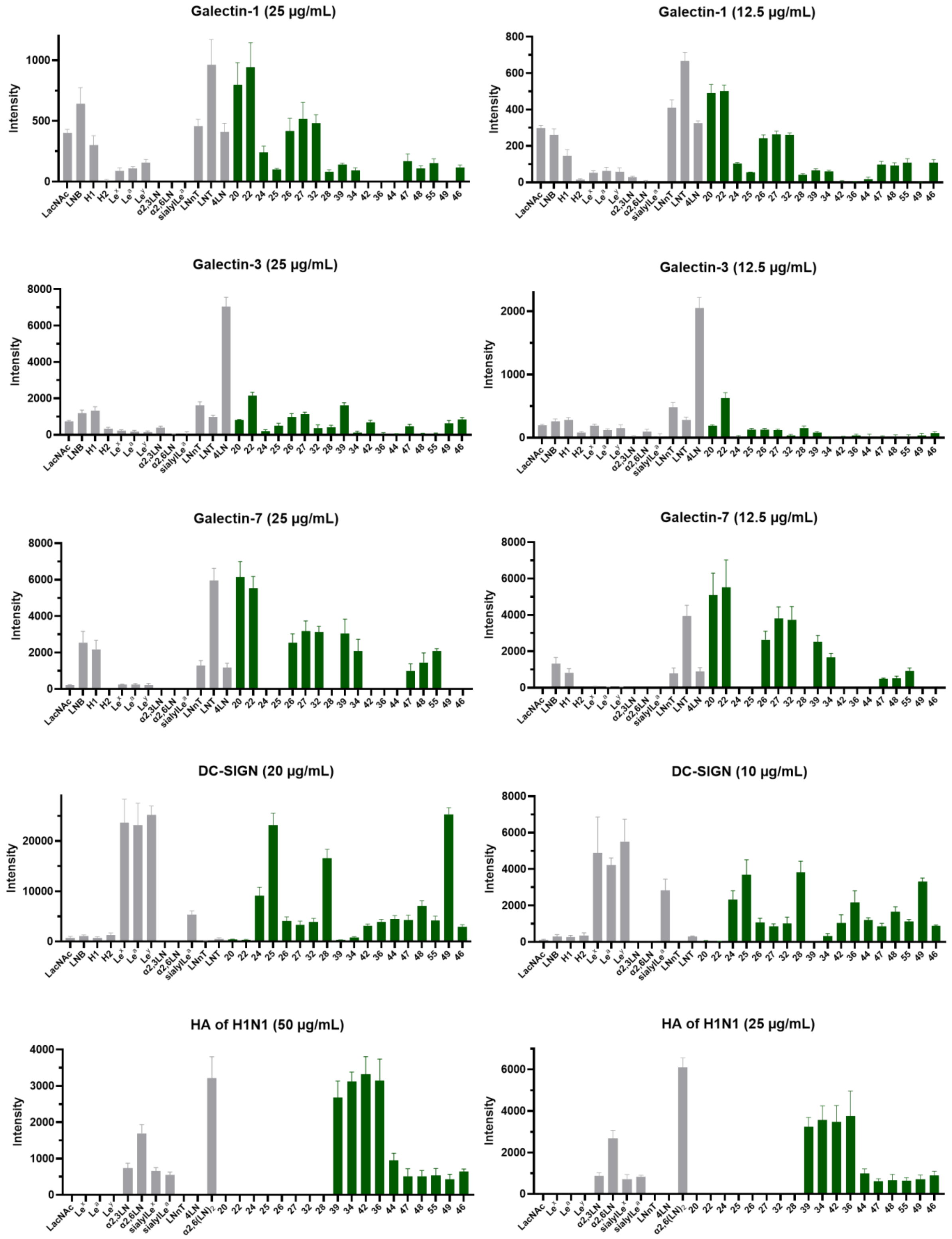

Figure S15. The binding profiles of different concentrations of GBPs with branched HMO microarray.

Detailed discussion of glycan microarray binding results with Galectins and DC-SIGN.

The binding profile for hGal-1 revealed that the presence of terminal Neu5Ac would significantly diminish the binding affinity. Compound **34** (5231a) and compound **39** (5131a) showed very weak binding signals due to the presence of α2,6-sialic acid. FS-series HMOs mostly showed lower binding signals because of sLe^a motif at β3 arm. Additionally, further assemblies of internal/terminal Fuc also led to reduce affinity for hGal-1. For example, further α1,4-fucosylated glycans, compound **24** (DF-LNH c) and compound **25** (TF-LNH), gave weaker binding singals. Moreover, compound **26** (DF-*i*LNO IV) containing LNB at its β6 arm and Le^b its β3 arm led to significantly lower affinity. Interestingly, compound **27** (5330a) and compound **32** (TetraF-*i*LNO) containing additional type 1 H motif and inner Le^x motif showed similar binding affinities as that of compound **26** (DF-*i*LNO IV). However, compound **28** (PentaF-*i*LNO) which contains Le^b motif at both arms significantly reduced the binding affinity. Similarly, compound **36** (5331a) and compound **42** (5231b) with Le^b motif at β3 arm fully abolished the affinity. By contrast, compound **44** (FS-LNH IV), lacking an extended sugar chain at the β6 arm, and compound **49** (DFS-*i*LNO II) which carries Le^a motif at the β6 arm were unable to bind with hGal-1.

Although the hGal-3 preferred **4LN** over than other glycans, our binding results showed that hGal-3 exhibited different binding affinities toward these branched glycans. Compound **39** (5131a), derived from distal α2,6-sialylation on diLacNAc of compound **22** (5130c), generates comparable binding affinity to LNnT. Compound **42** (5231b) with Le^b motif rather than type 1 H motif at β3 arm would reduce the interaction. Additionally, compound **34** (5231a) and compound **36** (5331a) bearing extra inner Le^x motif abolished the affinity. The binding strengths of compound **22** (5130c) and compound **39** (5131a) were higher than those of compound **20** (FLNH1), compound **24** (DF-LNH c), and compound **25** (TF-LNH), manifesting the necessity of longer LacNAc. The binding signals ofcompound **26** (DF-*i*LNO IV) and compound **27** (5330a) were similar with that of LNT; however, the other *i*LNO based branched HMOs compound **28** (PentaF-*i*LNO) and compound **32** (TetraF-*i*LNO) including inner Le^x showed weaker signals. This phenomenon can also be observed in FS-series HMOs. No binding signals were detected for compound **48** (DFS-*i*LNO I) and compound **54** (TFS-*i*LNO).

The binding profile for hGal-7 showed that LNT was the better ligand than LNnT, consistent with previous reports. The α1,2-fucosylated LNB generating type 1 H motif would not affect the binding affinity. Branched HMOs compound **20** (FLNH1) and its β6 arm extended derivative compound **22** (5130c) exhibited the strongest binding intensity with hGal-7. This indicated that longer glycan at β6 arm would not affect the interaction, and binding affinity majorly resulted from type 1 H motif at β3 arm. Both compound **24** (DF-LNH c) and compound **25** (TF-LNH) containing Le^b motif were not the ligands for hGal-7. Intriguingly, further LNB extension at β6 arm yielding compound **26** (DF-*i*LNO IV),

compound **27** (5330a), and compound **32** (TetraF-*i*LNO) would regain the hGal-7 binding affinity. Compound **27** (5330a) and compound **32** (TetraF-*i*LNO) exhibited stronger signals attributed to the presence of type 1 H motif at β6 arm. Again, compound **28** (PentaF-*i*LNO) failed to bind with hGal-7 due to the presence of two Le^b motifs which blocked the interaction. Compared to compound **34** (5231a) and compound **39** (5131a), the presence of Le^b motifs at two distal locations in compound **36** (5331a) and compound **42** (5231b) would also disrupted the binding interaction. For FS-series glycans, sLe^a motif would significantly reduced the binding signals. Thus, the exclusive presence of LNB at the β6 arm or further modified with α1,2-Fuc or inner α1,3-Fuc served as ligands for hGal-7.

Our glycan microarray binding results of DC-SIGN showed that glycans containing Le^x, Le^a, or Le^y motif provided extremely strong signals. Neither type 1 H nor type 2 H motif contributed to the binding with DC-SIGN. Consequently, compound **20** (FLNH1) and compound **22** (5130c) showed no binding signals. Compound **25** (TF-LNH), compound **28** (PentaF-*i*LNO), and compound **49** (DFS-*i*LNO II) with two terminal Le^x, Le^a, and/or Le^b motifs at both arms, exhibited strongest signals among tested branched HMOs. Compound **24** (DF-LNH c), compound **26** (DF-*i*LNO IV), compound **27** (5330a), and compound **32** (TetraF-*i*LNO) containing one Le^b motif showed weaker signals, albeit compound **32** (TetraF-*i*LNO) has an internal Le^x motif. This also indicated that the longer glycan present in the β6 arm would decrease the binding affinity (compound **26** (DF-*i*LNO IV), compound **27** (5330a), and compound **32** (TetraF-*i*LNO) vs compound **24** (DF-LNH c)). For α2,6-sialylated glycans, compound **36** (5331a) and compound **42** (5231b) binded with DC-SIGN while compound **34** (5231a) and compound **39** (5131a), lack of Le^b motif, could not bind. All FS-series HMOs having sLe^a motif at their β3 arm could also serve as weaker ligands for DC-SIGN. Compound **48** (DFS-*i*LNO I) and compound **49** (DFS-*i*LNO II) within internal Le^x motif could slightly enhance this binding compared to compound **47** (FS-*i*LNO I), but additional decoration of type 1 H motif on compound **55** (TFS-*i*LNO) cuased the reduce of binding affinity.

Reference

1. Chien, W. T.; Liang, C. F.; Yu, C. C.; Lin, C. H.; Li, S. P.; Primadona, I.; Chen, Y. J.; Mong, K. K.; Lin, C. C. Sequential one-pot enzymatic synthesis of oligo-*N*-acetyllactosamine and its multisialylated extensions. *Chem. Commun.* **2014**, *50*, 5786-5789.
2. Guan, W.; Cai, L.; Wang, P. G. Highly efficient synthesis of UDP-GalNAc/GlcNAc analogues with promiscuous recombinant human UDP-GalNAc pyrophosphorylase AGX1. *Chem. Eur. J.* **2010**, *16*, 13343-13345.
3. Blixt, O.; van Die, I.; Norberg, T.; van den Eijnden, D. H. High-level expression of the Neisseria meningitidis lgtA gene in Escherichia coli and characterization of the encoded Nacetylglucosaminyltransferase as a useful catalyst in the synthesis of GlcNAc

β1-->3Gal and GalNAc β1-->3Gal linkages. *Glycobiology* **1999**, *9*, 1061-1071.

4. Fang, J.-L.; Tsai, T.-W.; Liang, C.-Y.; Li, J.-Y.; Yu, C.-C. Enzymatic Synthesis of Human Milk Fucosides α1,2-Fucosyl para-Lacto-*N*-Hexaose and its Isomeric Derivatives. *Adv. Synth. Catal.* **2018**, *360*, 3213-3219.
5. Li, S.-P.; Hsiao, W.-C.; Yu, C.-C.; Chien, W.-T.; Lin, H.-J.; Huang, L.-D.; Lin, C.-H.; Wu, W.-L.; Wu, S.-H.; Lin, C.-C. Characterization of Meiothermus taiwanensis Galactokinase and its Use in the OnePot Enzymatic Synthesis of Uridine Diphosphate-Galactose and the Chemoenzymatic Synthesis of the Carbohydrate Antigen Stage Specific Embryonic Antigen-3. *Adv. Synth. Catal.* **2014**, *356*, 3199- 3213.
6. Litterer, L. A.; Schnurr, J. A.; Plaisance, K. L.; Storey, K. K.; Gronwald, J. W.; Somers, D. A. Characterization and expression of Arabidopsis UDP-sugar pyrophosphorylase. *Plant Physiol. Biochem.* **2006**, *44*, 171-180.
7. Liu, X. W.; Xia, C.; Li, L.; Guan, W. Y.; Pettit, N.; Zhang, H. C.; Chen, M.; Wang, P. G. Characterization and synthetic application of a novel β1,3-galactosyltransferase from Escherichia coli O55:H7. *Bioorg. Med. Chem.* **2009**, *17*, 4910-4905.
8. McArthur, J. B.; Yu, H.; Chen, X. A Bacterial β1–3-Galactosyltransferase Enables Multigram-Scale Synthesis of Human Milk Lacto-*N*-tetraose (LNT) and Its Fucosides. *ACS Catal.* **2019**, *9*, 10721- 10726.
9. Tsai, T.-I.; Lee, H.-Y.; Chang, S.-H.; Wang, C.-H.; Tu, Y.-C.; Lin, Y.-C.; Hwang, D.-R.; Wu, C.-Y.; Wong, C.-H. Effective Sugar Nucleotide Regeneration for the Large-Scale Enzymatic Synthesis of Globo H and SSEA4. *J. Am. Chem. Soc.* **2013**, *135*, 14831-14839.
10. Hou, K.-L.; Chiang, P.-Y.; Lin, C.-H.; Li, B.-Y.; Chien, W.-T.; Huang, Y.-T.; Yu, C.-C.; Lin, C.-C. Water-Soluble Sulfo-Fluorous Affinity (SOFA) Tag-Assisted Enzymatic Synthesis of Oligosaccharides. *Adv. Synth. Catal.* **2018**, *360*, 2313-2323.
11. Yu, H.; Li, Y.; Wu, Z.; Li, L.; Zeng, J.; Zhao, C.; Wu, Y.; Tasnima, N.; Wang, J.; Liu, H.; Gadi, M. R.; Guan, W.; Wang, P. G.; Chen, X. *H. pylori* α1-3/4-fucosyltransferase (Hp3/4FT)-catalyzed onepot multienzyme (OPME) synthesis of Lewis antigens and human milk fucosides. *Chem. Commun.* **2017**, *53*, 11012-11015.
12. Chiang, P. Y.; Adak, A. K.; Liang, W. L.; Tsai, C. Y.; Tseng, H. K.; Cheng, J. Y.; Hwu, J. R.; Yu, A. L.; Hung, J. T.; Lin, C. C. Chemoenzymatic Synthesis of Globo-series Glycosphingolipids and Evaluation of Their Immunosuppressive Activities. *Chem. Asian J.* **2022**, *17*, e202200403.
13. Yu, C. C.; Lin, P. C.; Lin, C. C. Site-specific immobilization of CMP-sialic acid synthetase on magnetic nanoparticles and its use in the synthesis of CMP-sialic acid. *Chem. Commun.* **2008**, *11*, 1308-1310.
14. Yu, C. C.; Kuo, Y. Y.; Liang, C. F.; Chien, W. T.; Wu, H. T.; Chang, T. C.; Jan, F. D.; Lin, C. C. Sitespecific immobilization of enzymes on magnetic nanoparticles and their use in

organic synthesis. *Bioconjugate Chem.* **2012**, *23*, 714-724.

15. Vireak Thon, Yanhong Li, Hai Yu, Kam Lau, Xi Chen. PmST3 from *Pasteurella multocida* encoded by *Pm1174* gene is a monofunctional α2-3-sialyltransferase. *Appl. Microbiol. Biotechnol.* **2012**, *94*, 977-985.
16. Go Sugiarto, Kam Lau, Jingyao Qu, Yanhong Li, Sunghyuk Lim, Shengmao Mu, James B. Ames, Andrew J. Fisher, and Xi Chen. A Sialyltransferase Mutant with Decreased Donor Hydrolysis and Reduced Sialidase Activities for Directly Sialylating Lewis^{x} *ACS Chem. Biol.* **2012**, *7*, 1232-1240.
17. Chiu, C. P.; Lairson, L. L.; Gilbert, M.; Wakarchuk, W. W.; Withers, S. G.; Strynadka, N. C. Structural analysis of the alpha-2,3-sialyltransferase Cst-I from Campylobacter jejuni in apo and substrateanalogue bound forms. *Biochemistry* **2007**, *46*, 7196-7204.
18. Zhang, Z. Y.; Ollmann, I. R.; Ye, X. S.; Wischnat, R.; Baasov, T.; Wong, C. H. Programmable one-pot oligosaccharide synthesis. *J. Am. Chem. Soc.* **1999**, *121*, 734-753.
19. Grann Hansen, S.; Skrydstrup, T. Studies directed to the synthesis of oligochitosans–preparation of building blocks and their evaluation in glycosylation studies. *Eur. J.Org. Chem.* **2007**, *2007*, 3392-3401.
20. Forman, A.; Pfoh, R.; Eddenden, A.; Howell, P. L.; Nitz, M. Synthesis of defined mono-de-*N*-acetylated β(1-->6)-*N*-acetyl-d-glucosamine oligosaccharides to characterize PgaB hydrolase activity. *Org. Biomol. Chem.* **2019**, *17*, 9456-9466.
21. Hsu, C. H.; Chu, K. C.; Lin, Y. S.; Han, J. L.; Peng, Y. S.; Ren, C. T.; Wu, C. Y.; Wong, C. H. Highly alpha-selective sialyl phosphate donors for efficient preparation of natural sialosides. *Chem. Eur. J.* **2010**, *16*, 1754-1760.
22. Fan, C. Y.; Kawade, S. K.; Adak, A. K.; Cho, C.; Tan, K. T.; Lin, C. C., Silver-Coated CuO Nanoparticle Substrates for Surface Azide-Alkyne Cycloaddition. *ACS Appl. Nano. Mater.* **2021**, *4*, 1558-1566.
23. Arthur, C. M.; Rodrigues, L. C.; Baruffi, M. D.; Sullivan, H. C.; Heimburg-Molinaro, J.; Smith, D. F.; Cummings, R. D.; Stowell, S. R. Examining galectin binding specificity using glycan microarrays. *Methods Mol. Biol.* **2015**, *1207*, 115-131.

NMR Spectra

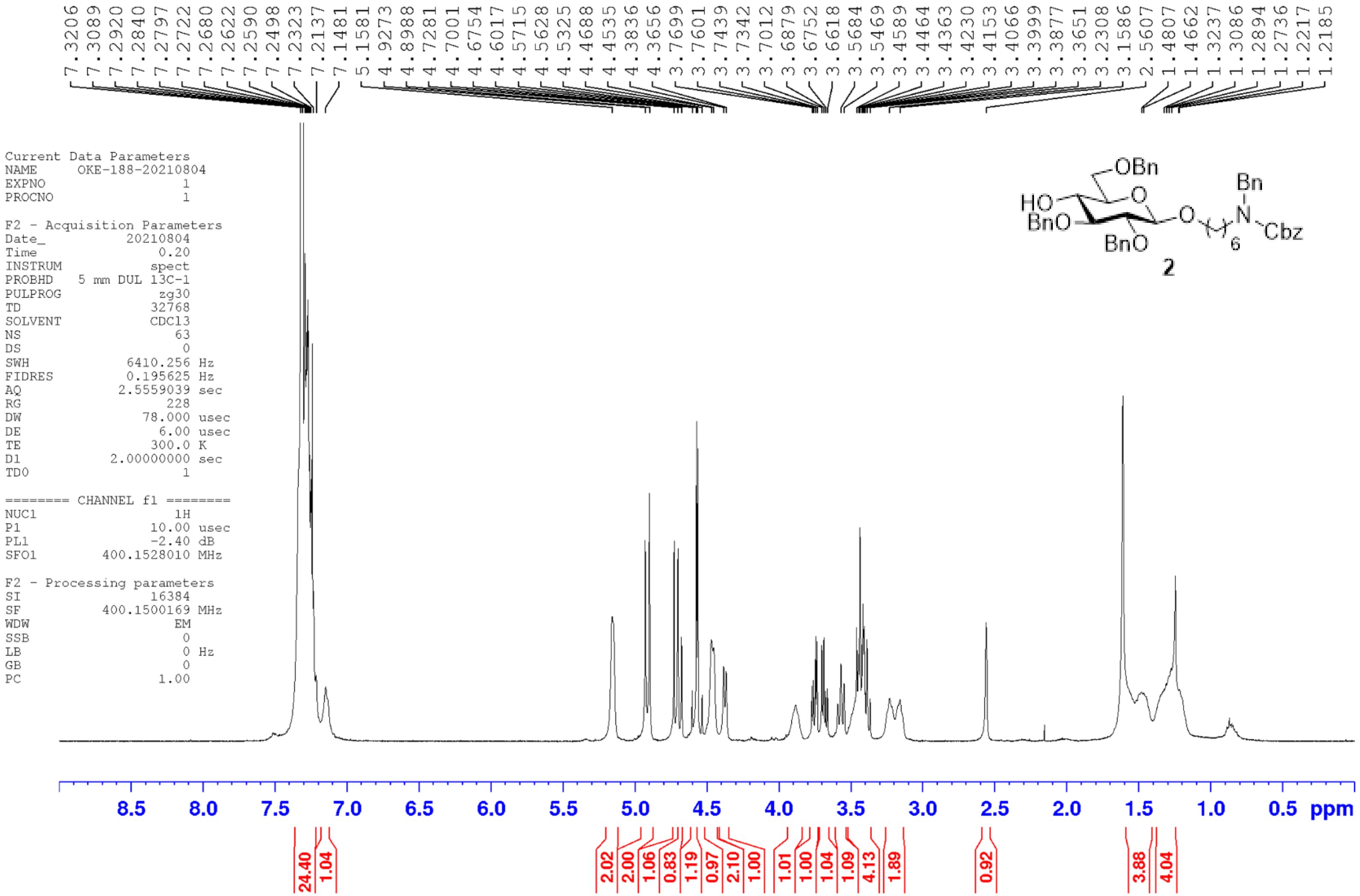

^{1}H NMR spectrum of Compound **2** (400 MHz $CDCl_3$).

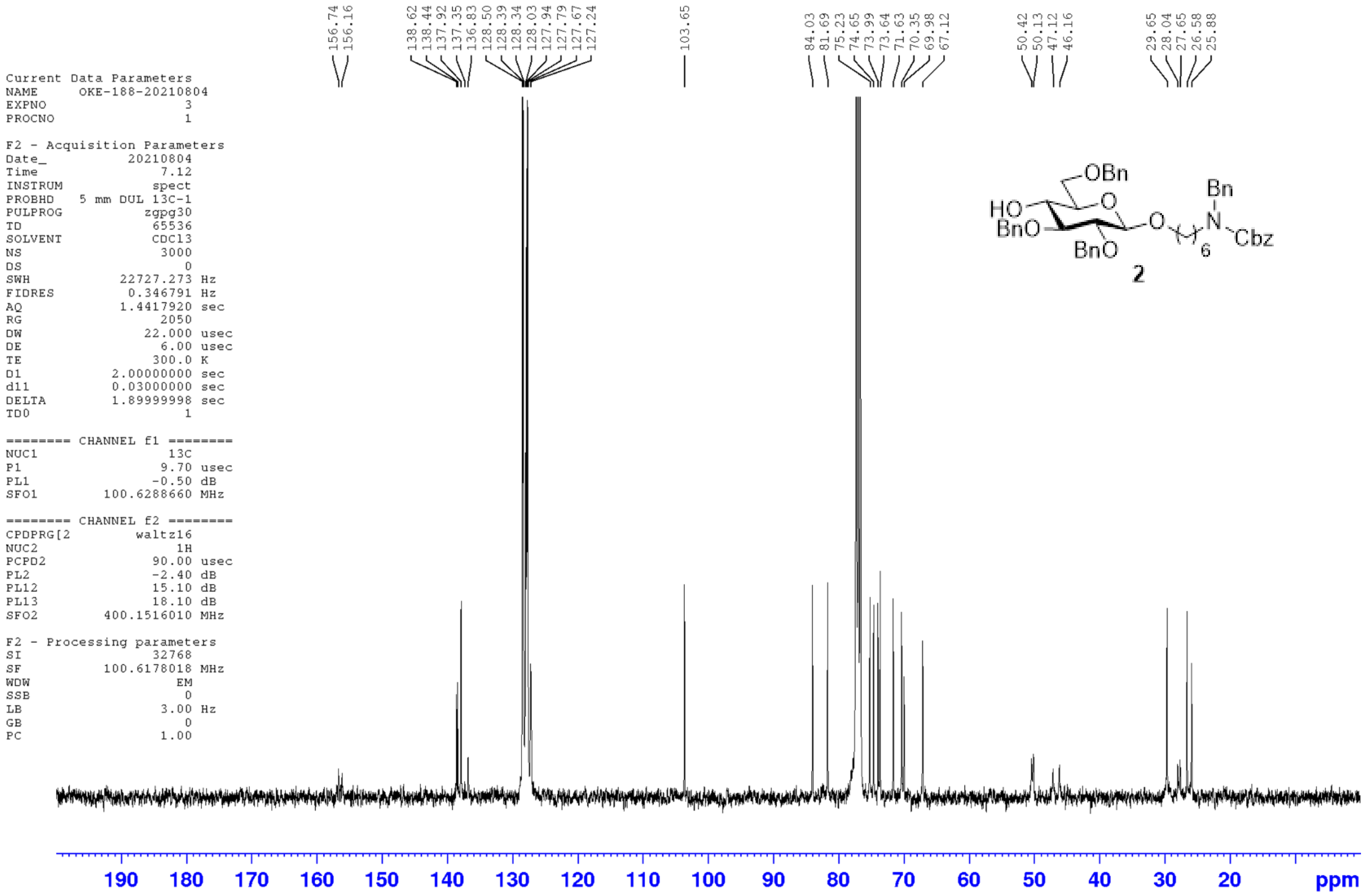

^{13}C NMR spectrum of Compound **2** (100 MHz $CDCl_3$).

```
Current Data Parameters
NAME     OKE-188-COSY-20220525
EXPNO                    2
PROCNO                   1

F2 - Acquisition Parameters
Date_           20220525
Time               14.16
INSTRUM            spect
PROBHD   5 mm DUL 13C-1
PULPROG         cosyqf90
TD                  2048
SOLVENT            CDCl3
NS                     4
DS                    16
SWH             5197.505 Hz
FIDRES          2.537844 Hz
AQ             0.1970176 sec
RG                   128
DW                96.200 usec
DE                  6.00 usec
TE                 300.0 K
d0            0.00000300 sec
D1            2.00000000 sec
IN0           0.00019240 sec

======== CHANNEL f1 ========
NUC1                  1H
P1                 10.00 usec
PL1                -2.40 dB
SFO1         400.1520008 MHz

F1 - Acquisition parameters
TD                   256
SFO1             400.152 MHz
FIDRES         40.605511 Hz
SW                12.989 ppm
FnMODE                QF

F2 - Processing parameters
SI                  1024
SF           400.1500194 MHz
WDW                 SINE
SSB                    0
LB                     0 Hz
GB                     0
PC                  1.40

F1 - Processing parameters
SI                  1024
MC2                   QF
SF           400.1500197 MHz
WDW                 SINE
SSB                    0
LB                     0 Hz
GB                     0
```

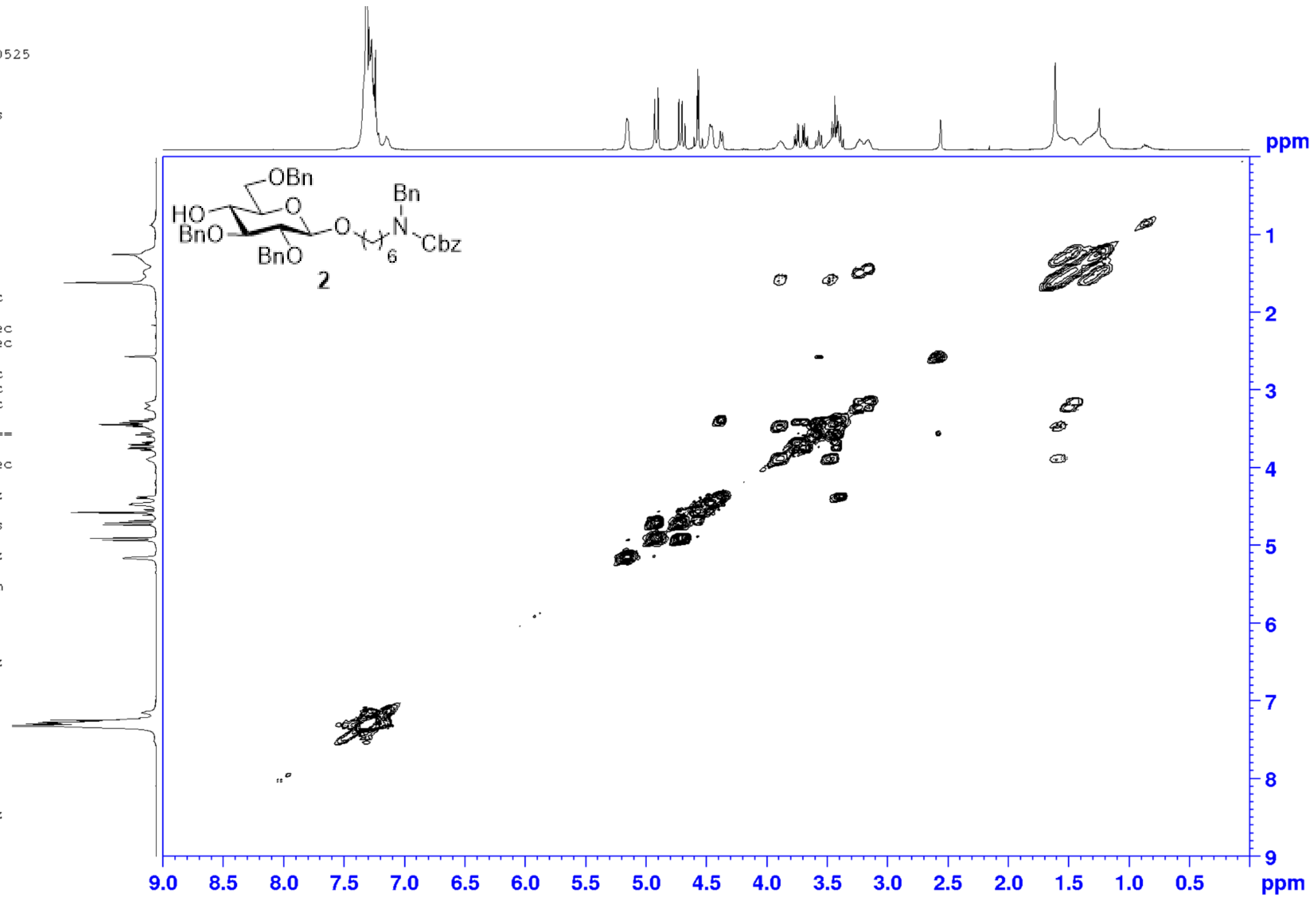

^1H-^1H COSY NMR spectrum of Compound **2** (400 MHz $CDCl_3$).

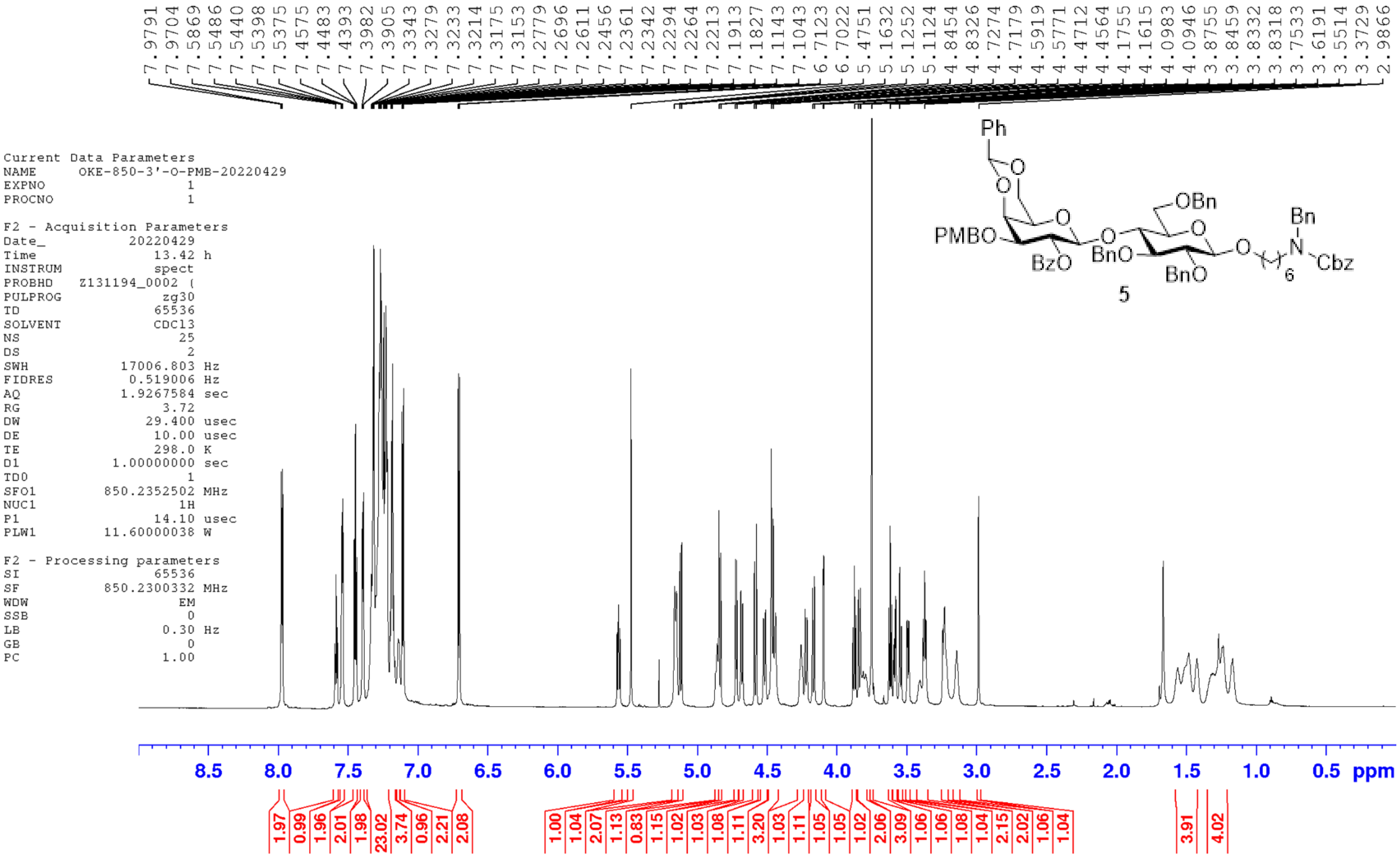

^{1}H NMR spectrum of Compound **5** (850 MHz $CDCl_3$).

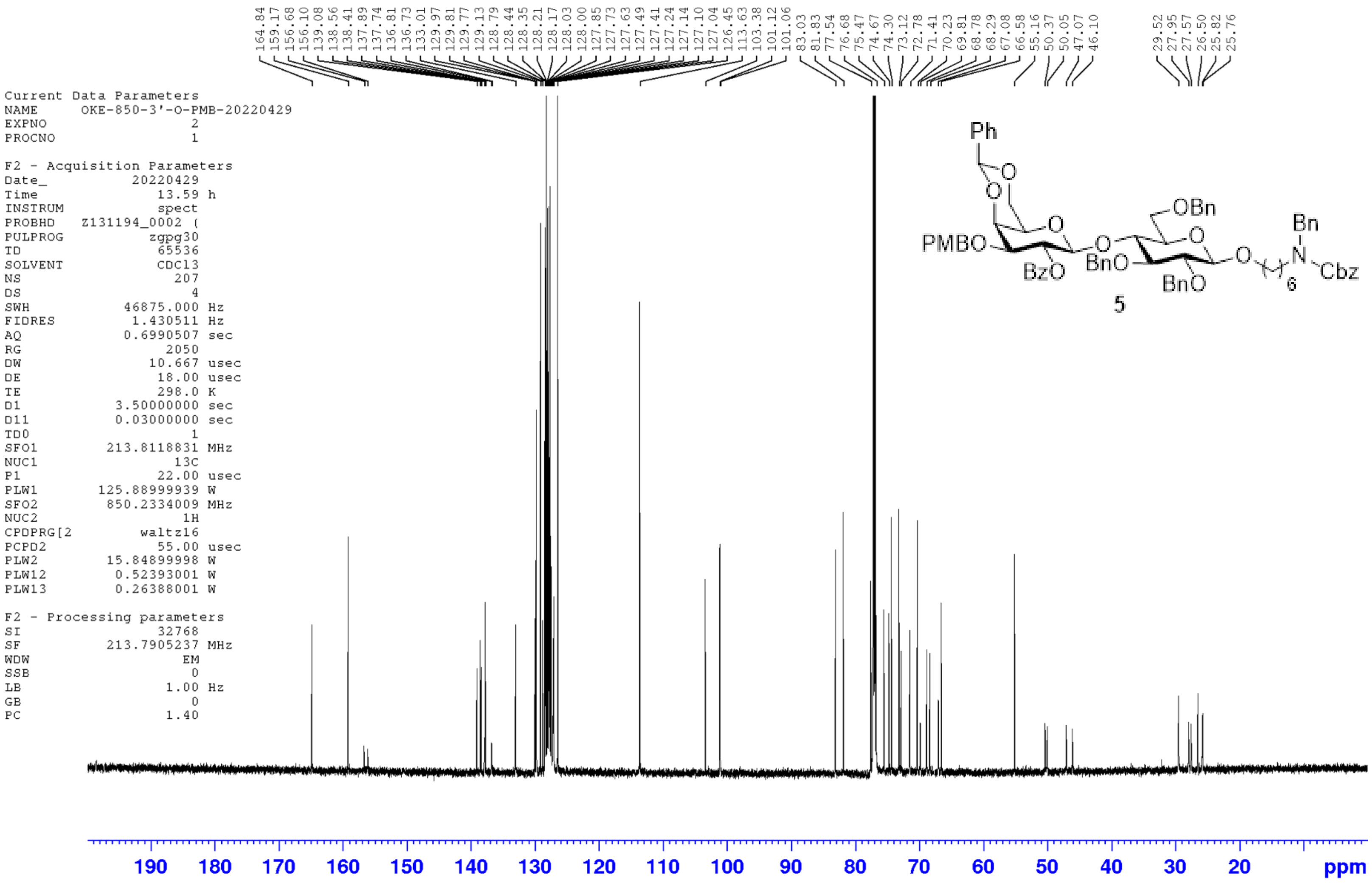

^{13}C NMR spectrum of Compound **5** (214 MHz $CDCl_3$).

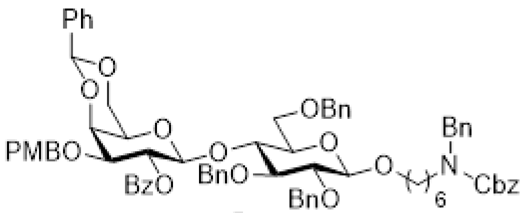

5

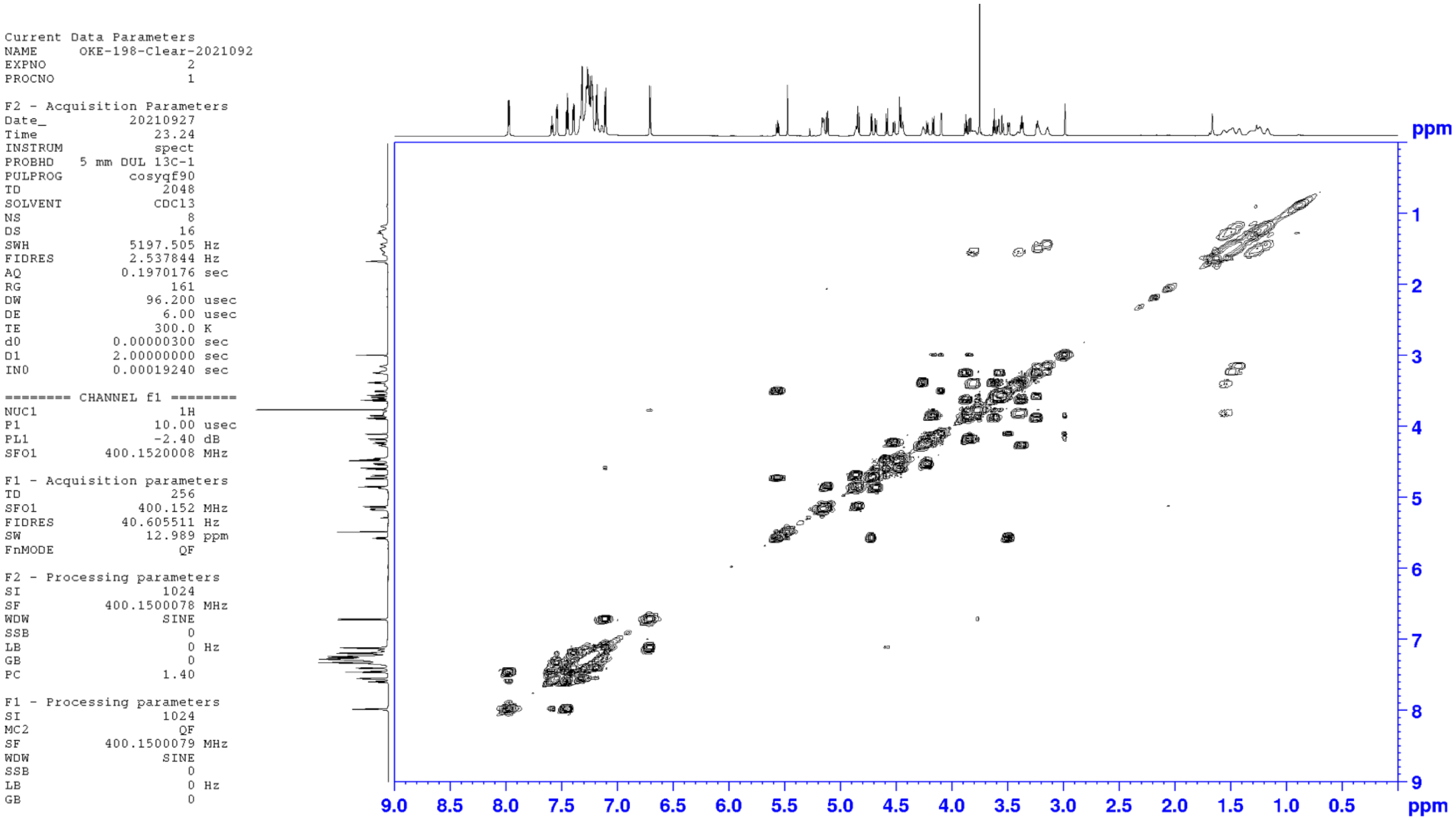

^{1}H-^{1}H COSY NMR spectrum of Compound **5** (850 MHz $CDCl_3$).

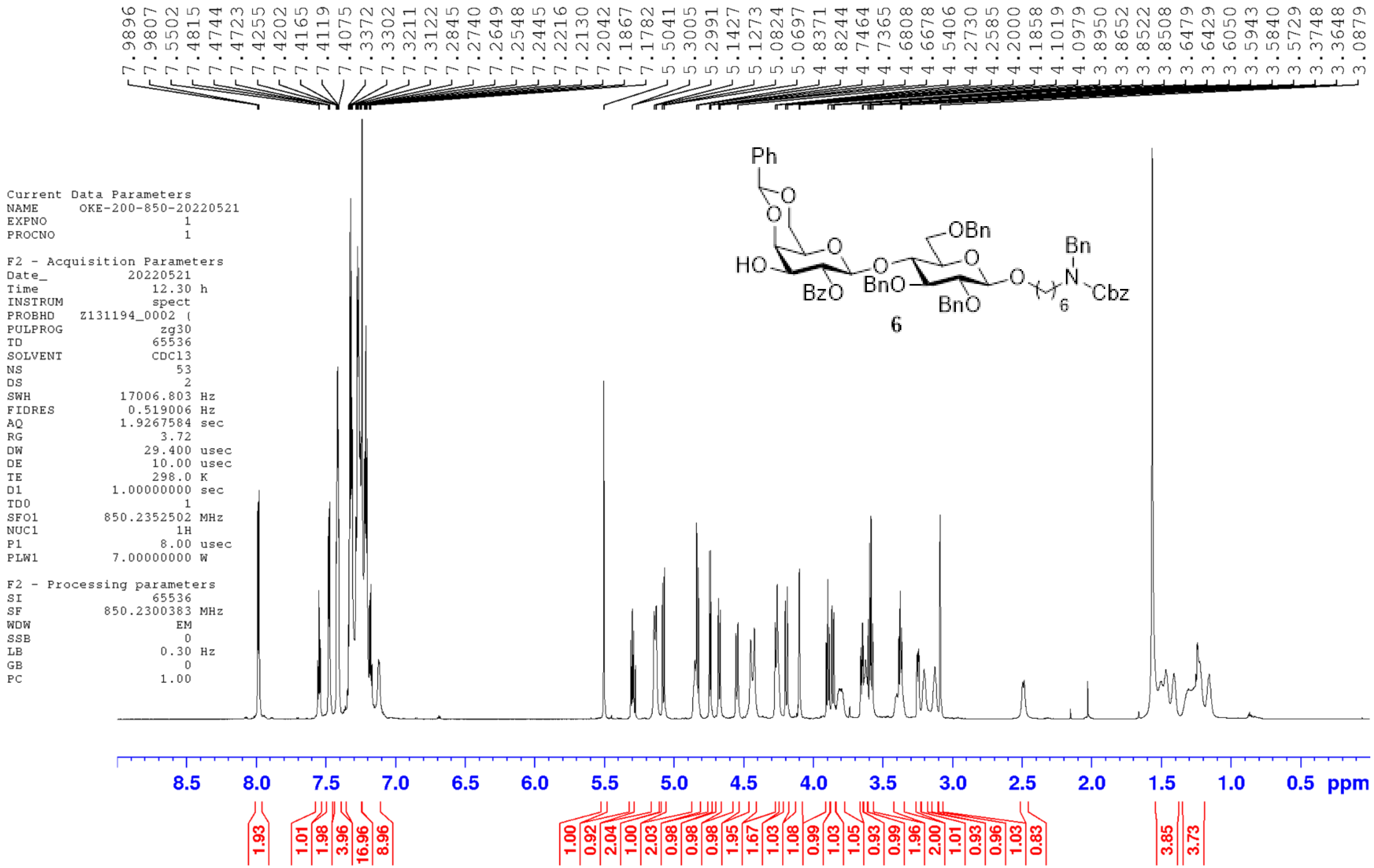

^{1}H NMR spectrum of Compound **6** (850 MHz $CDCl_3$).

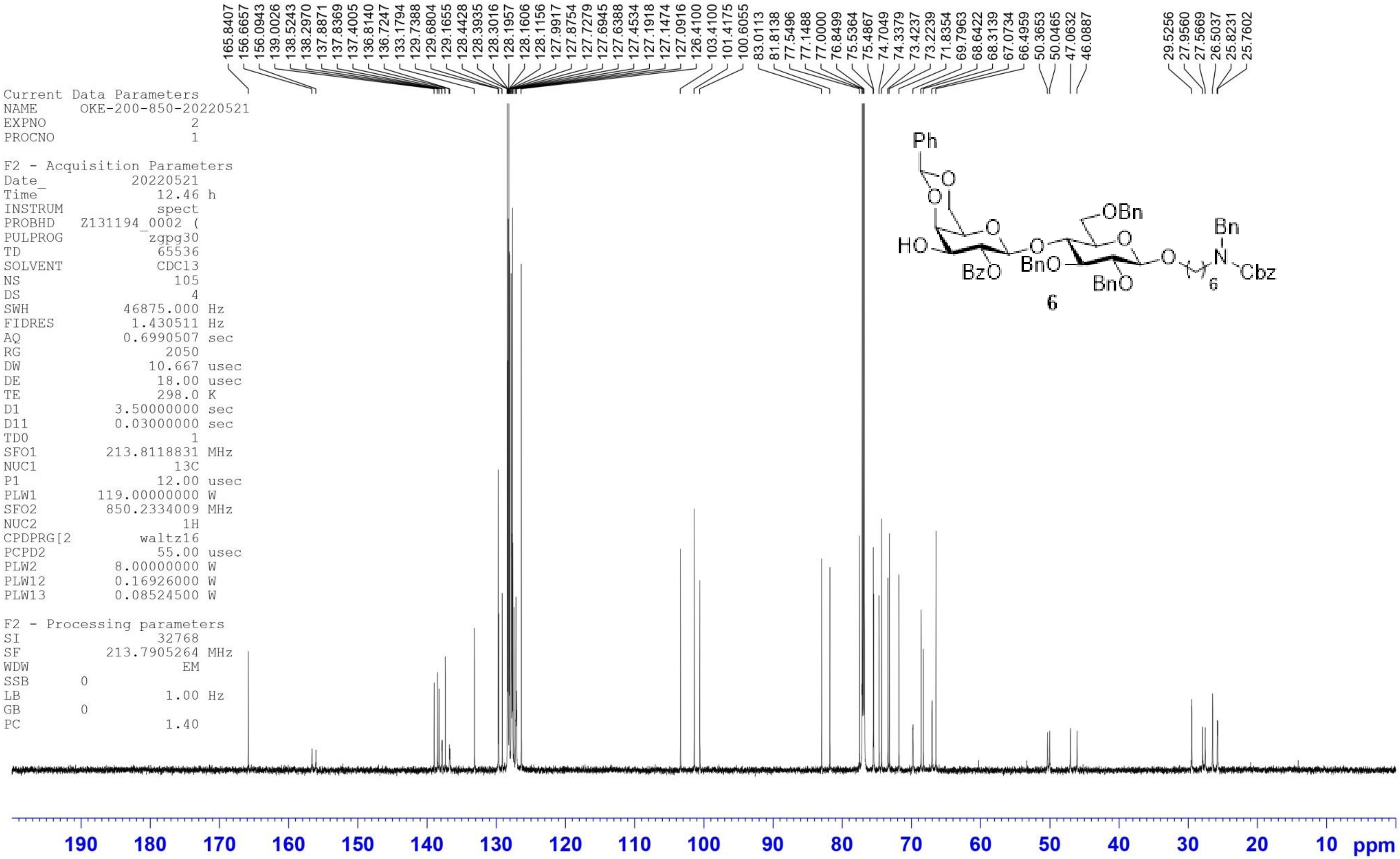

^{13}C NMR spectrum of Compound **6** (214 MHz $CDCl_3$).

```
Current Data Parameters
NAME      OKE-200-COSY-20
EXPNO                   1
PROCNO                  1

F2 - Acquisition Paramet
Date_            20210731
Time                 0.23
INSTRUM             spect
PROBHD   5 mm DUL 13C-1
PULPROG          cosyqf90
TD                   2048
SOLVENT             CDCl3
NS                     16
DS                     16
SWH              5197.505
FIDRES           2.537844
AQ              0.1970176
RG                    362
DW                 96.200
DE                   6.00
TE                  300.0
d0             0.00000300
D1             2.00000000
IN0            0.00019240

======== CHANNEL f1 ====
NUC1                   1H
P1                  10.00
PL1                 -2.40
SFO1          400.1520008

F1 - Acquisition paramet
TD                    256
SFO1              400.152
FIDRES          40.605511
SW                 12.989
FnMODE                 QF

F2 - Processing paramete
SI                   1024
SF            400.1500167
WDW                  SINE
SSB                     0
LB                      0
GB                      0
PC                   1.40

F1 - Processing paramete
SI                   1024
MC2                    QF
SF            400.1500172
WDW                  SINE
SSB                     0
LB                      0
GB                      0
```

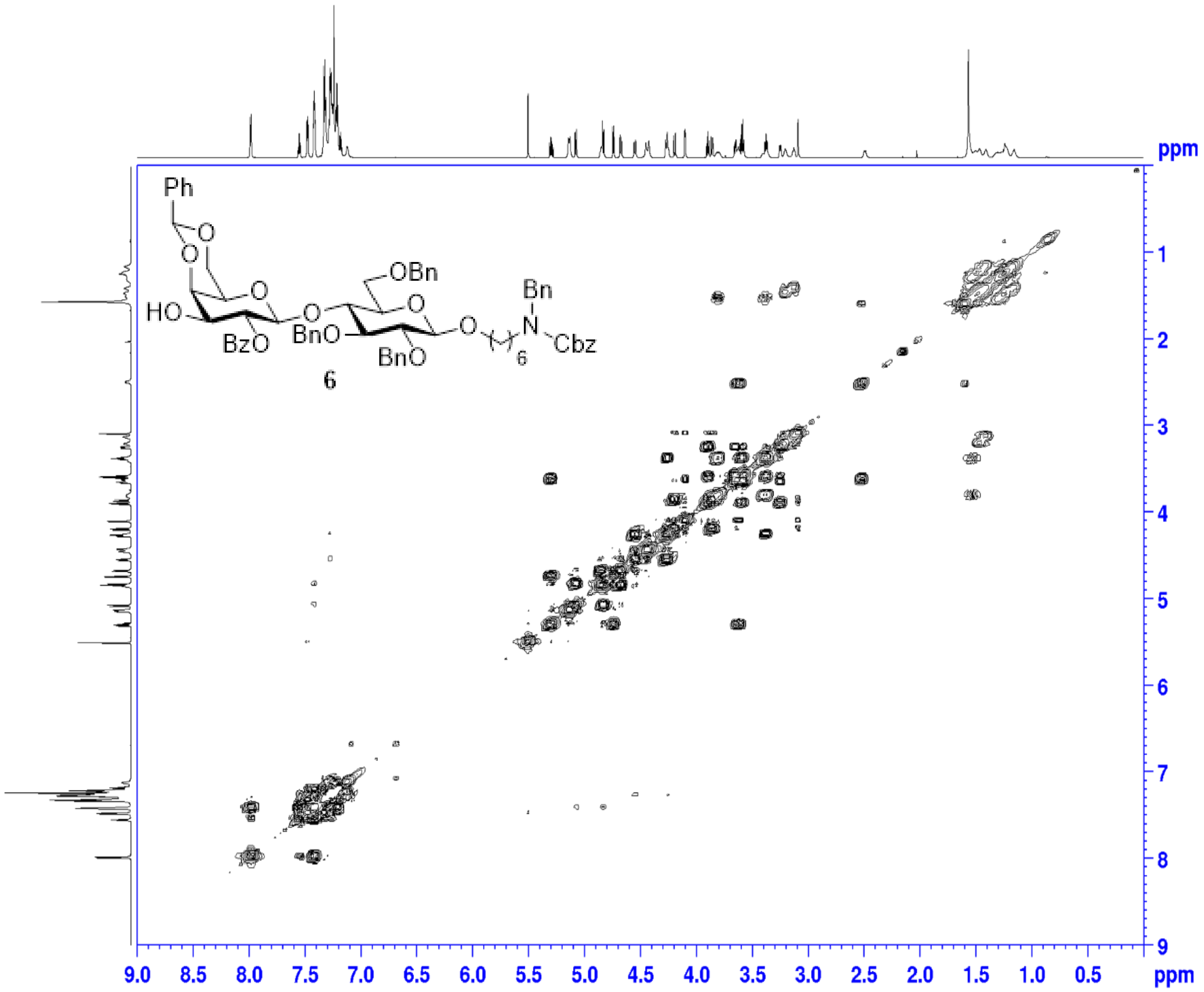

^{1}H-^{1}H COSY NMR spectrum of Compound **6** (850 MHz $CDCl_3$).

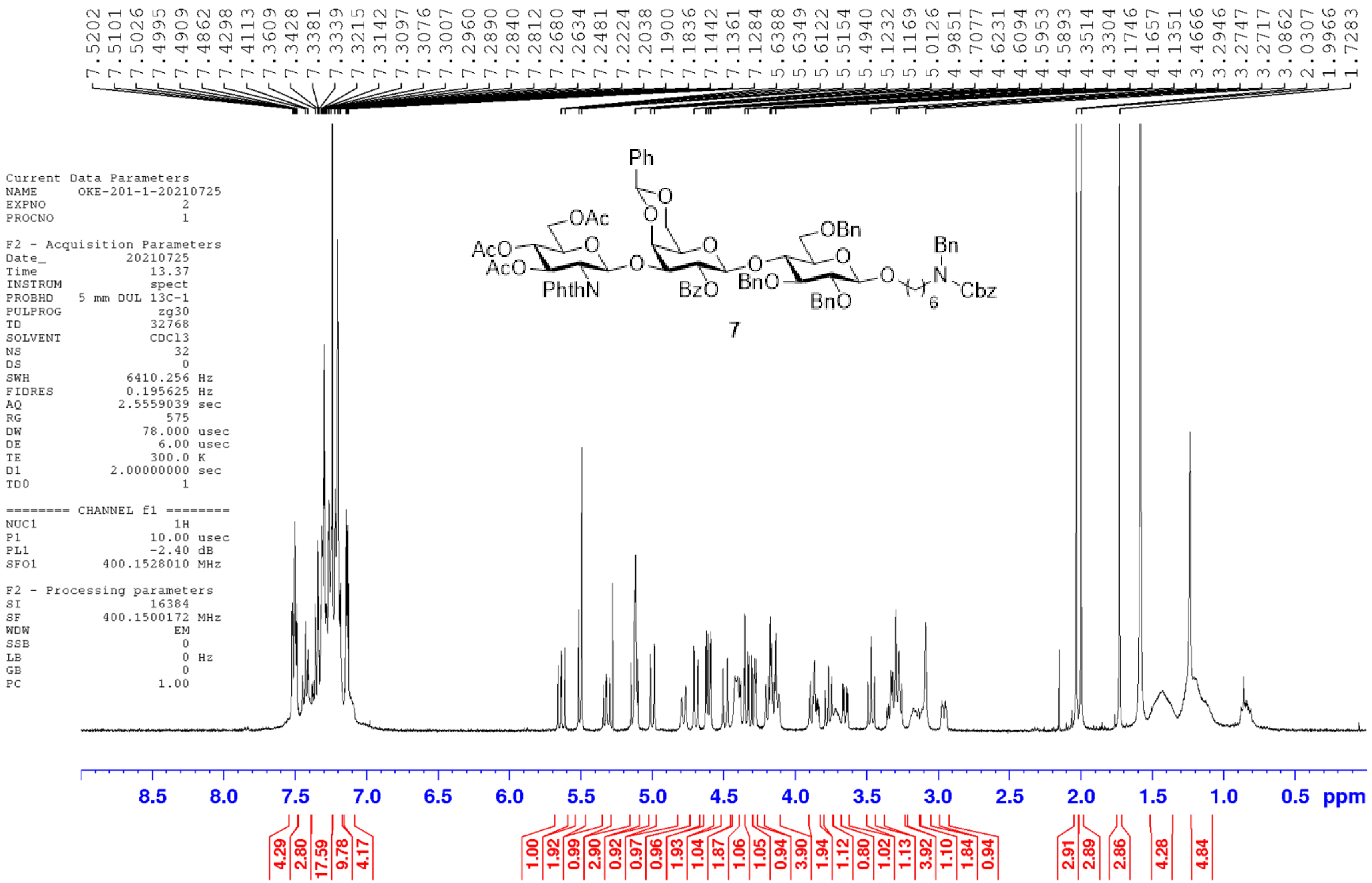

^{1}H NMR spectrum of Compound **7** (400 MHz, $CDCl_3$).

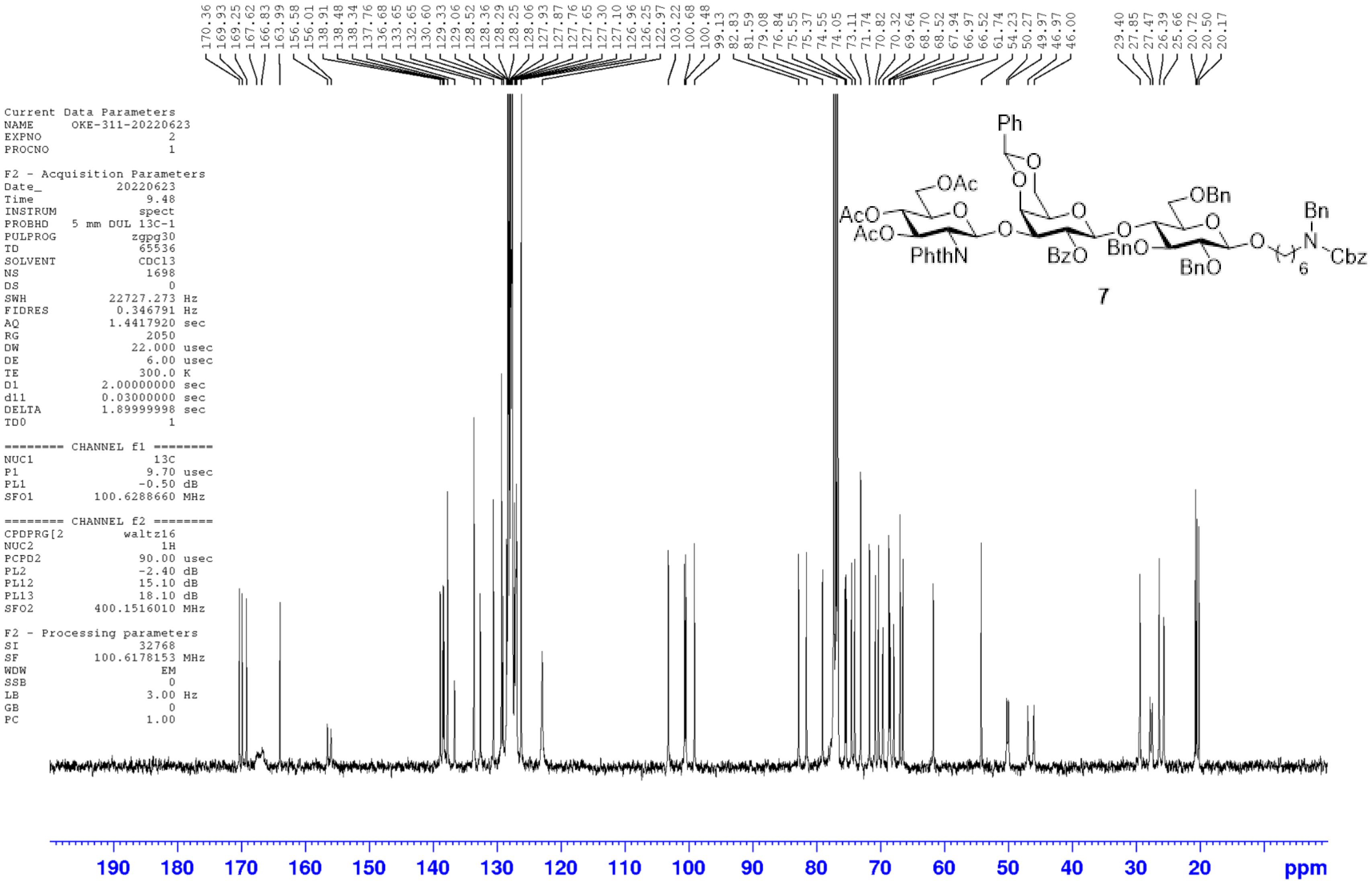

^{13}C NMR spectrum of Compound **7** (100 MHz, $CDCl_3$).

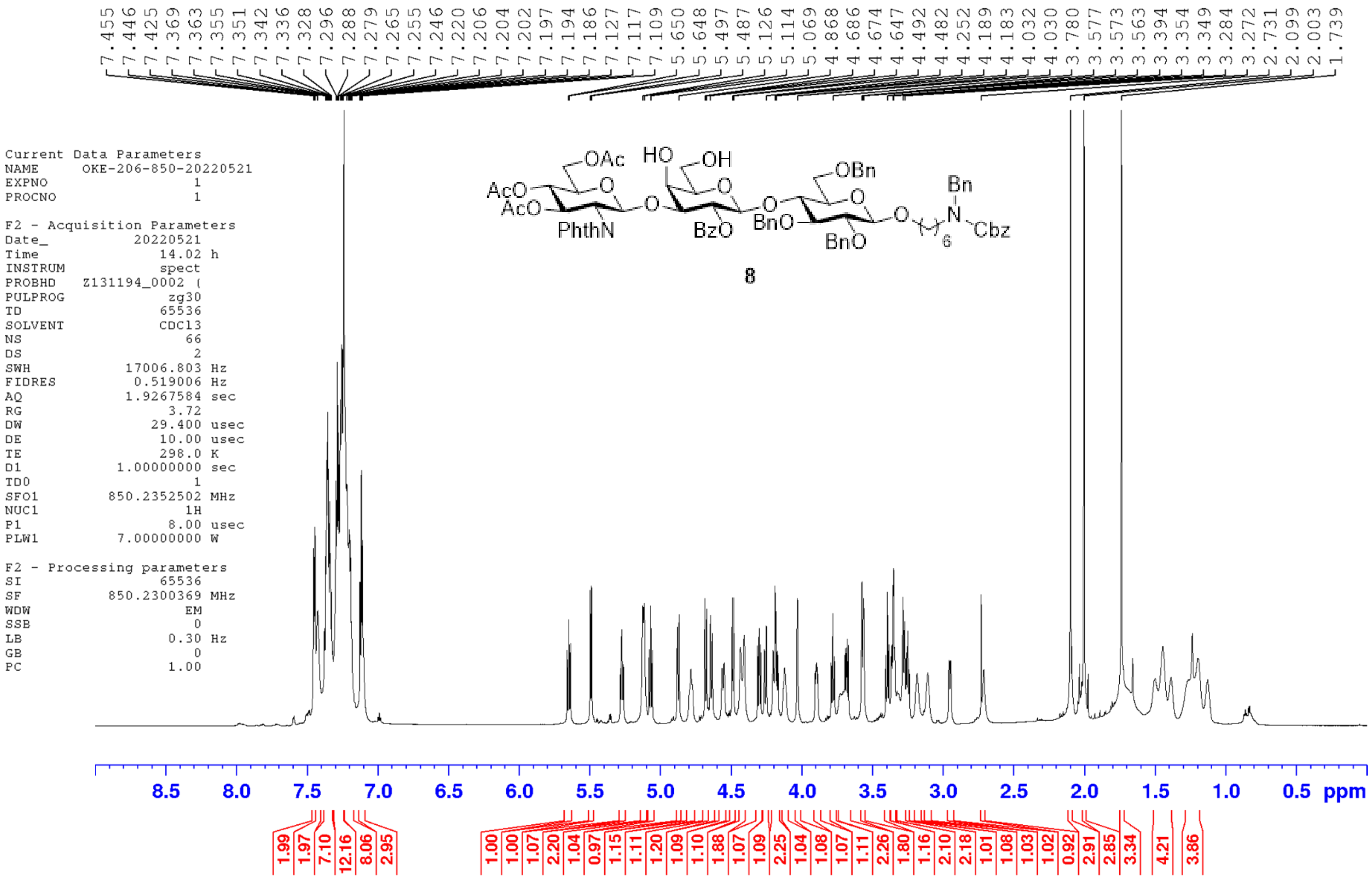

^{1}H NMR spectrum of Compound **8** (850 MHz, $CDCl_3$).

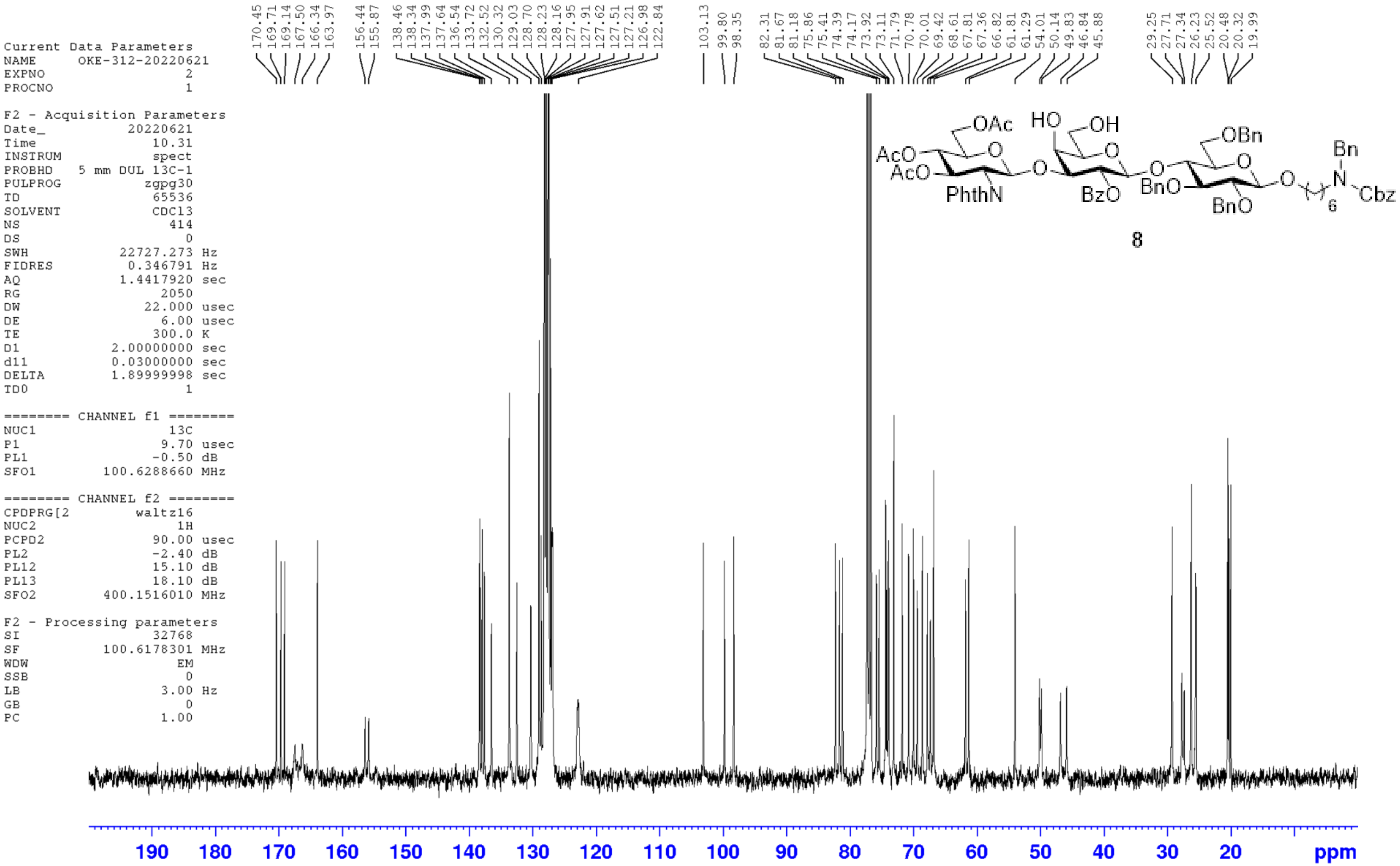

^{13}C NMR spectrum of Compound **8** (214 MHz, $CDCl_3$).

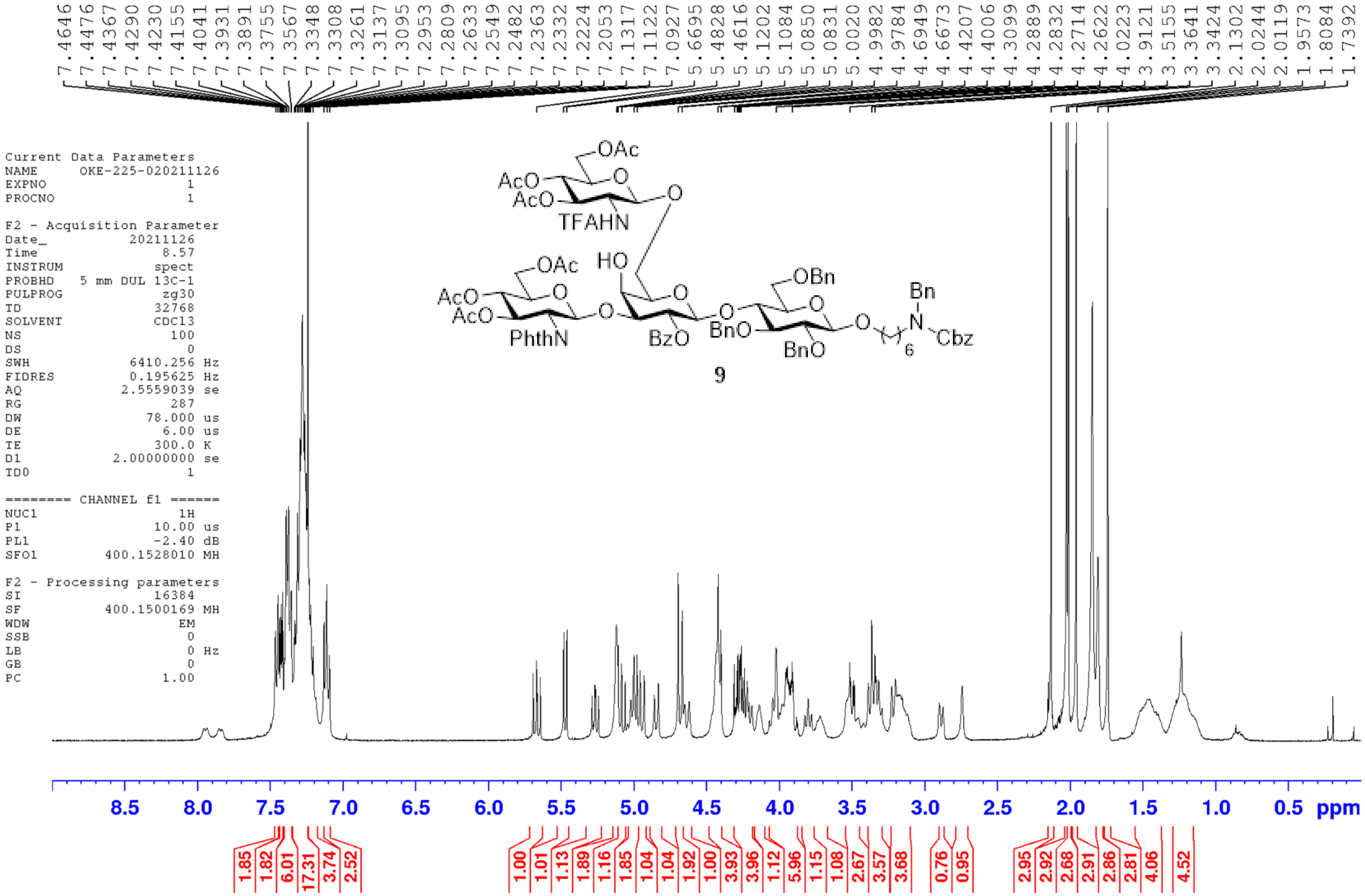

^{1}H NMR spectrum of Compound **9** (400 MHz, $CDCl_3$).

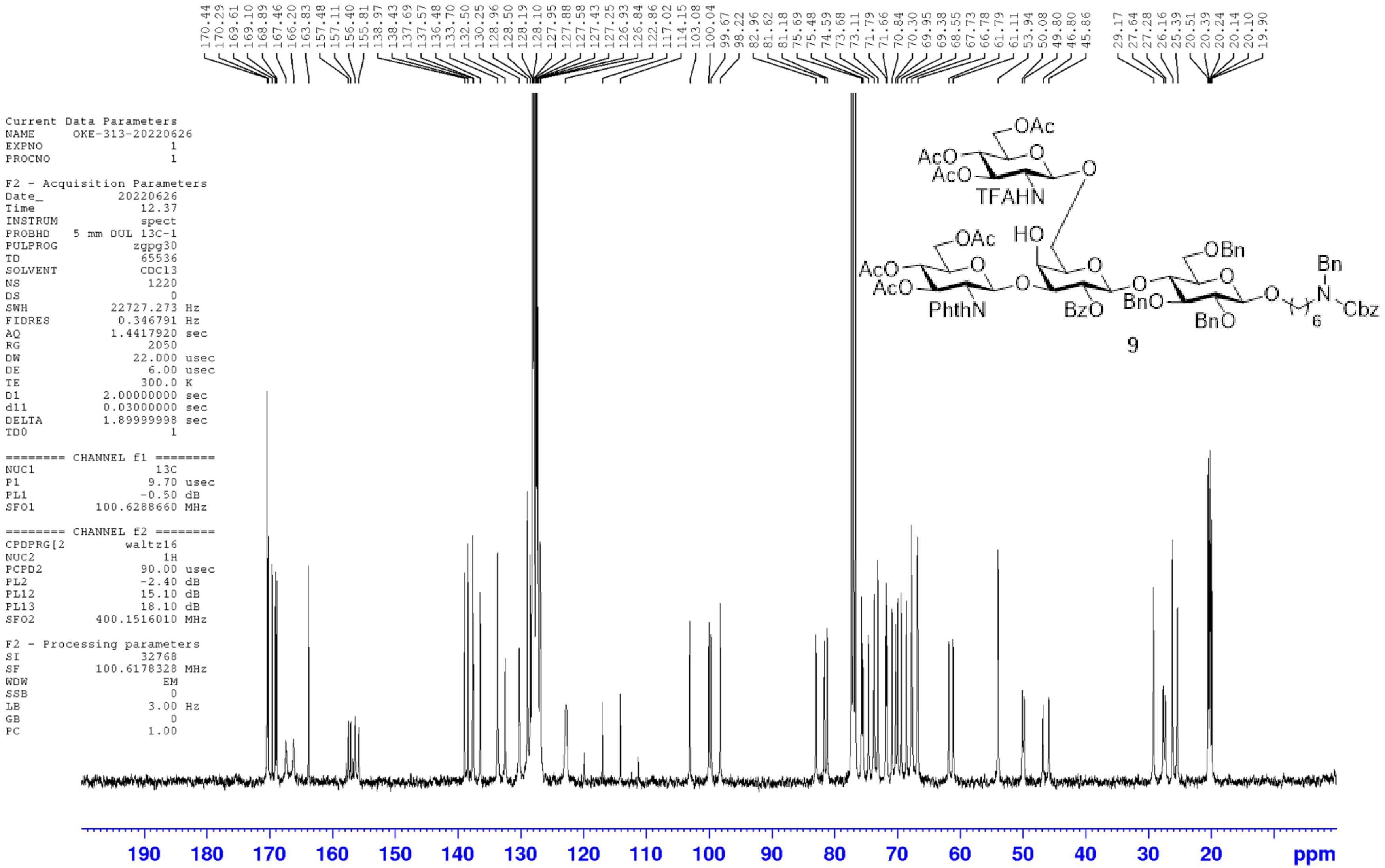

^{13}C NMR spectrum of Compound **9** (100 MHz, $CDCl_3$).

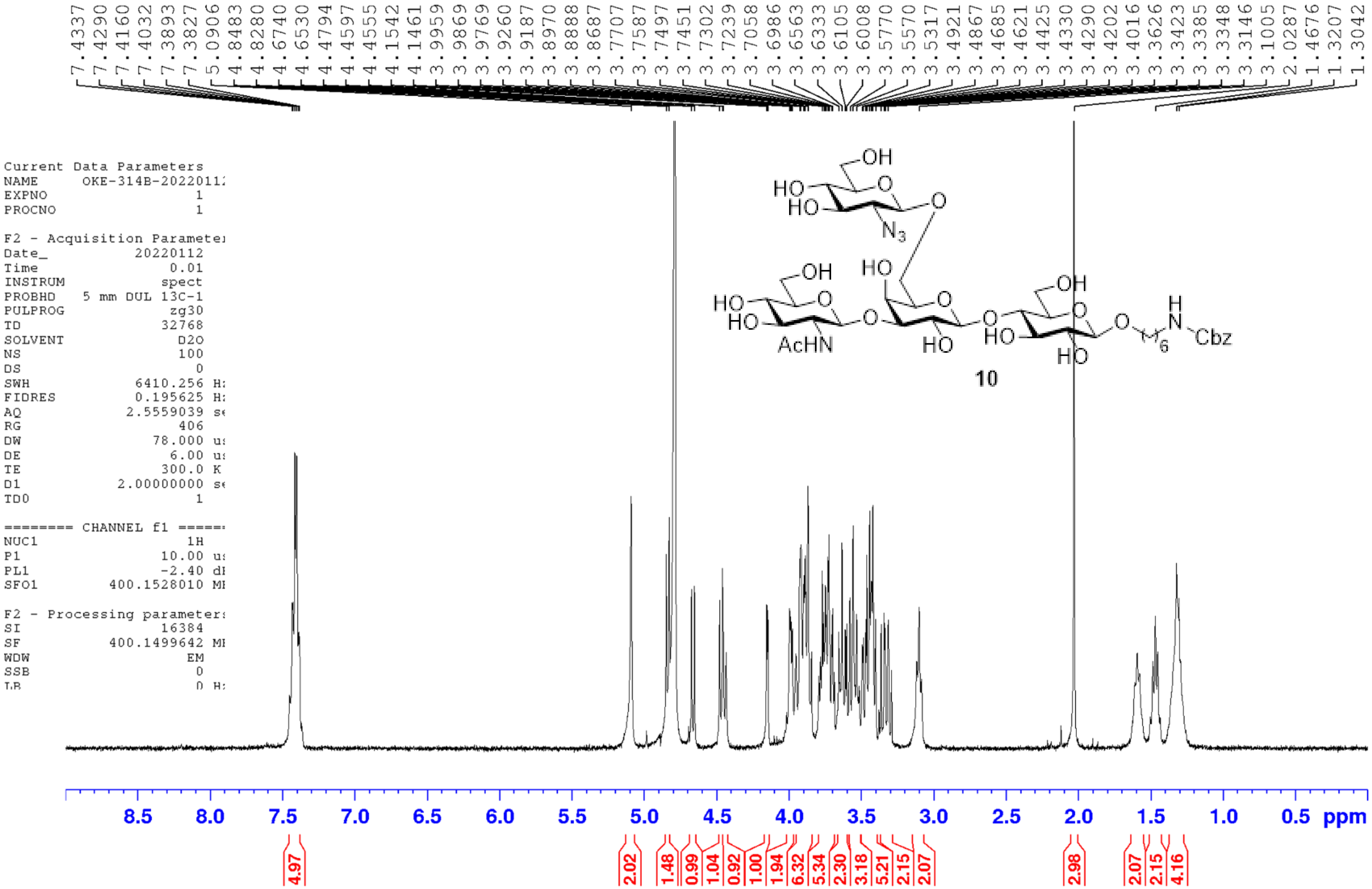

^{1}H NMR spectrum of Compound **10** (400 MHz, D_2O).

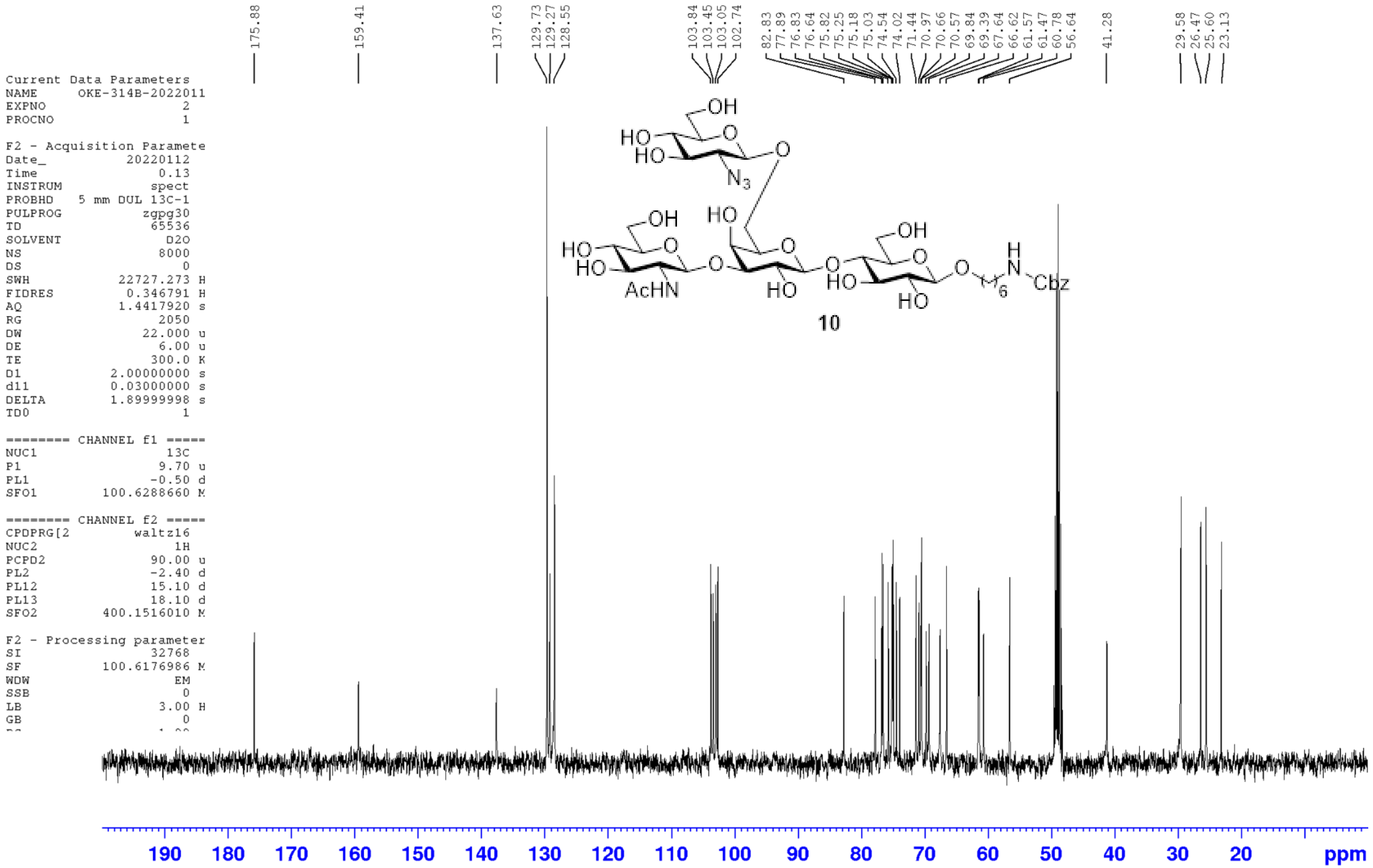

^{13}C NMR spectrum of Compound **10** (100 MHz, D_2O).

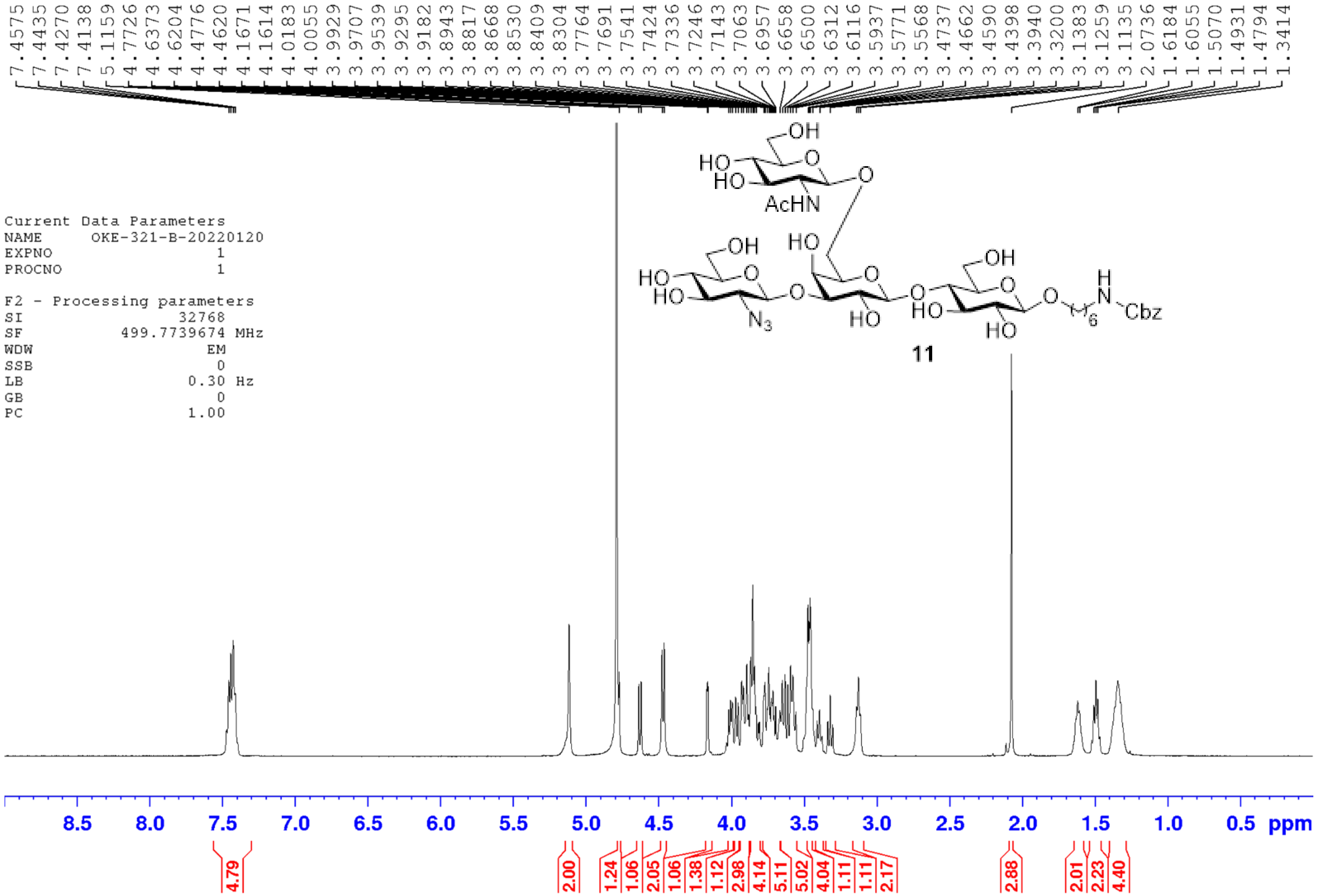

^{1}H NMR spectrum of Compound **11** (500 MHz, D_2O).

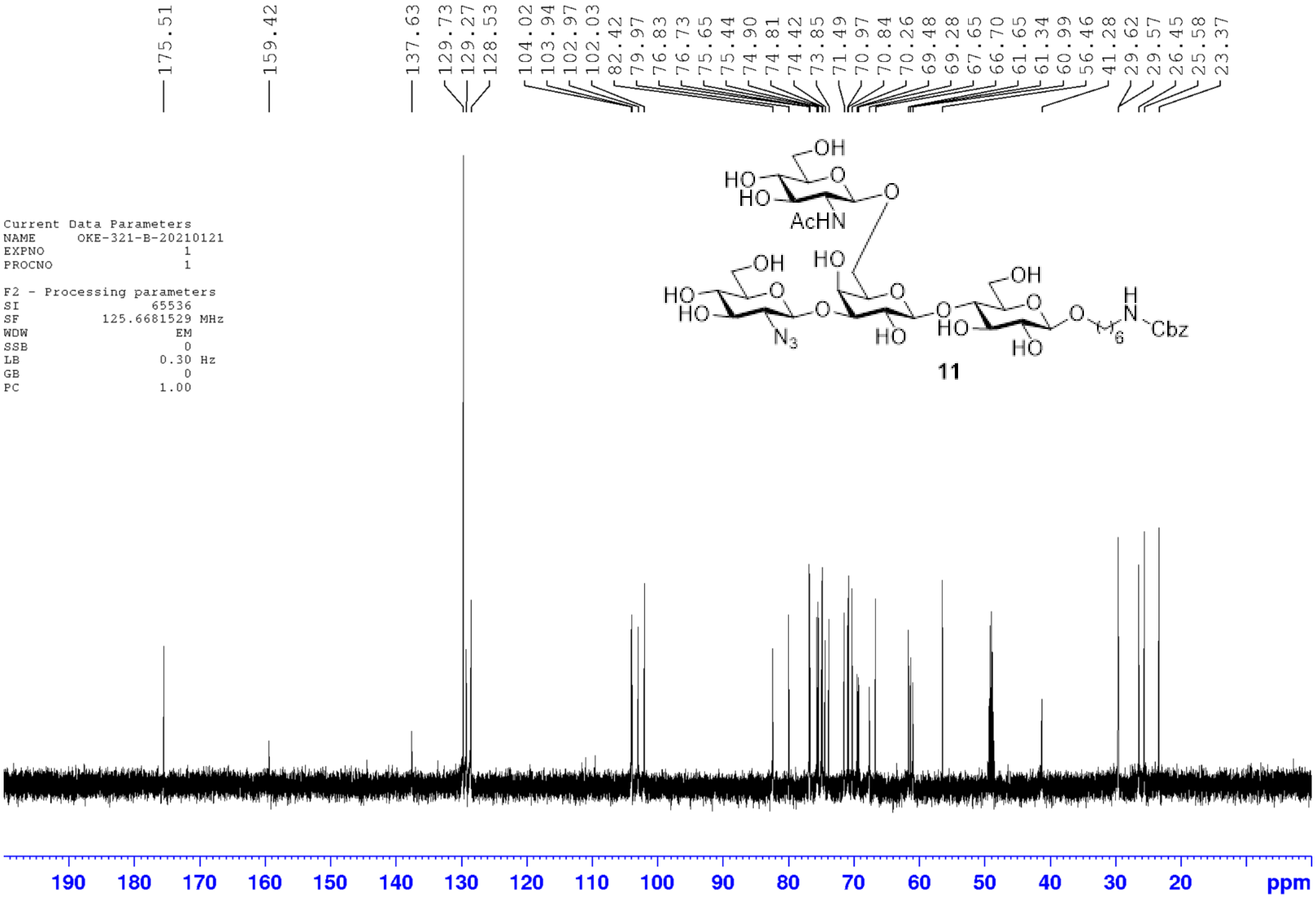

^{13}C NMR spectrum of Compound **11** (125 MHz, D_2O).

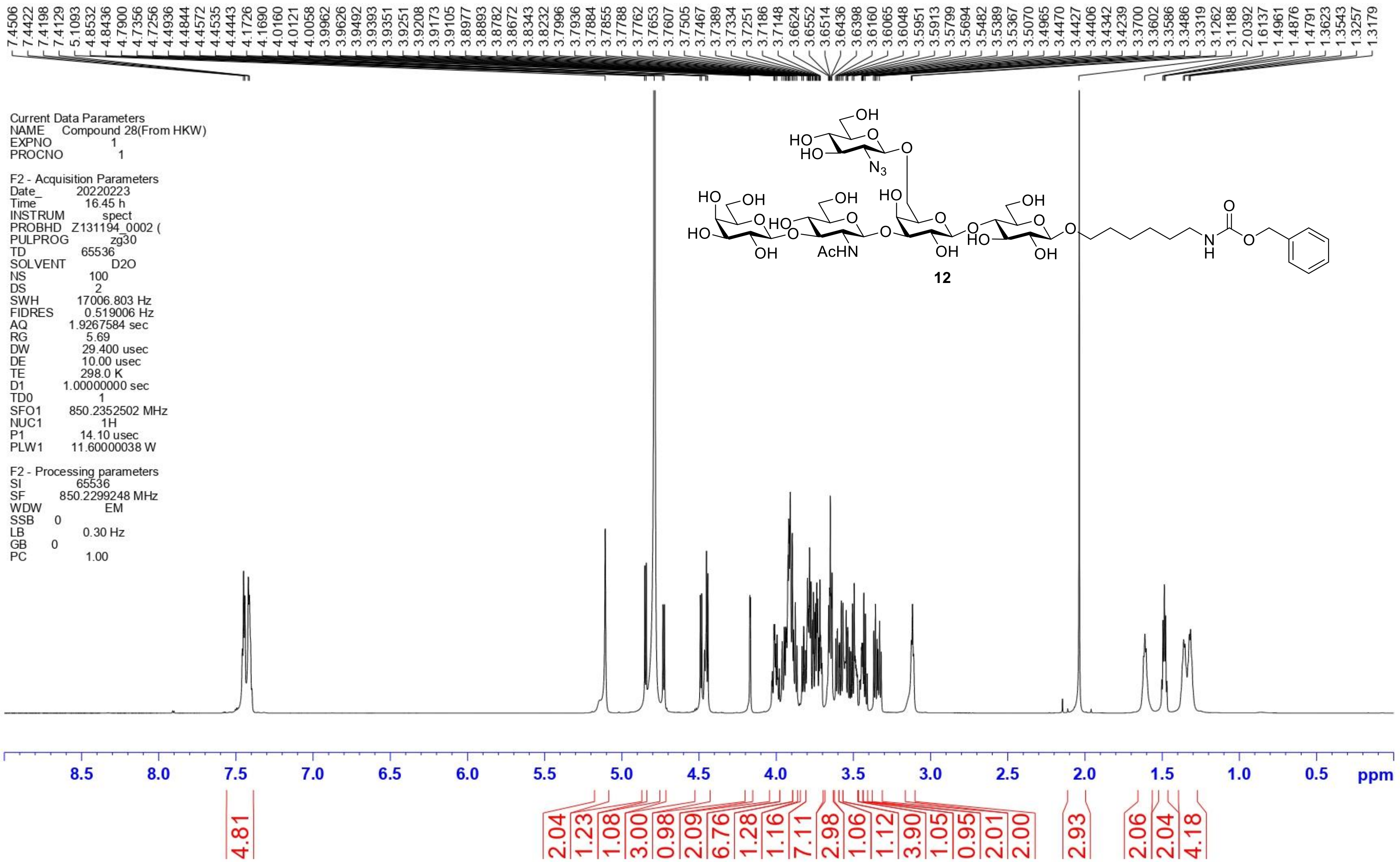

^{1}H NMR spectrum of compound **12** (850 MHz, D_2O)

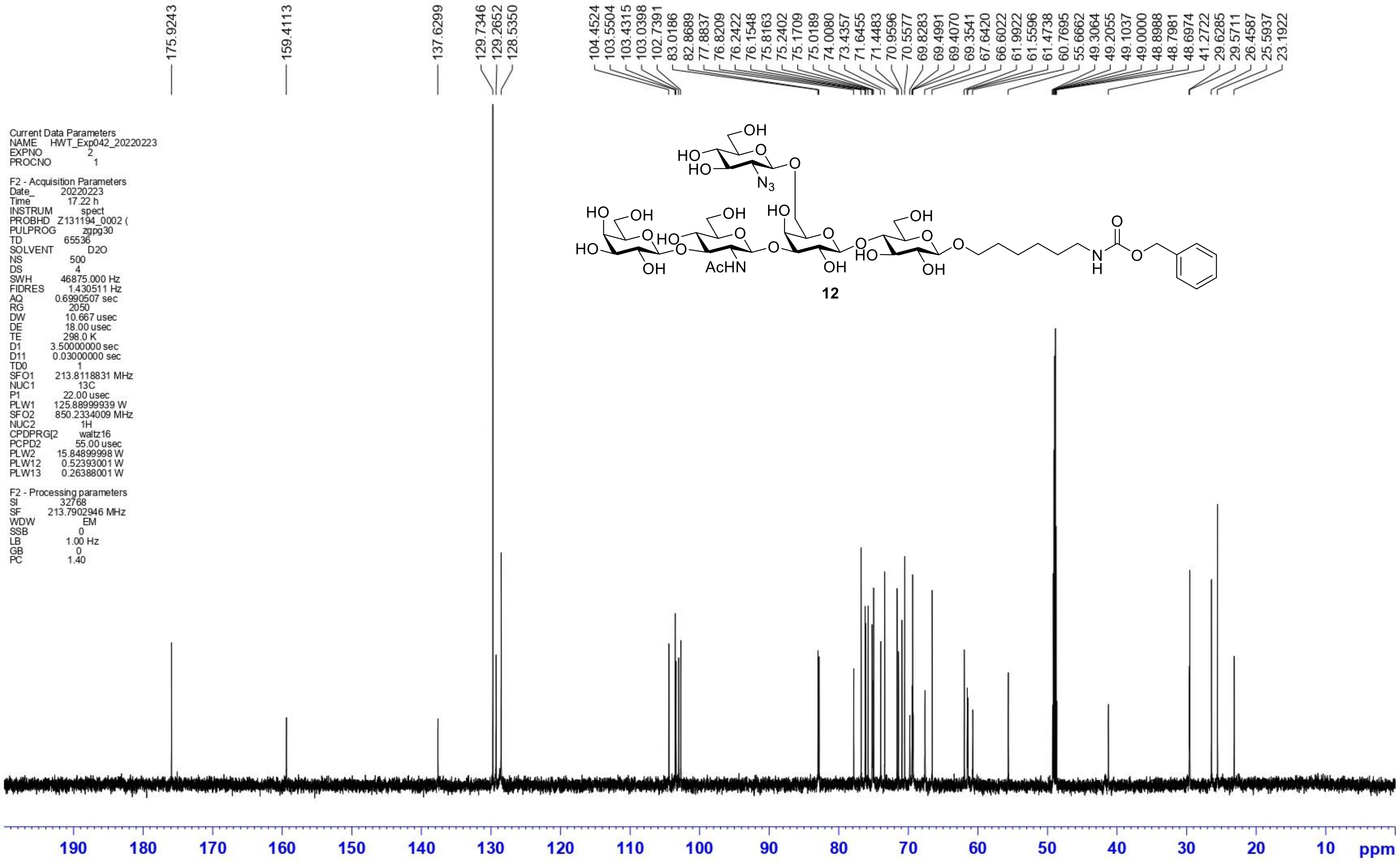

^{13}C NMR spectrum of compound **12** (214 MHz, D_2O)

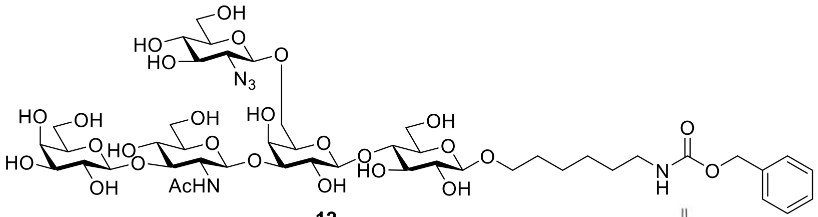

12

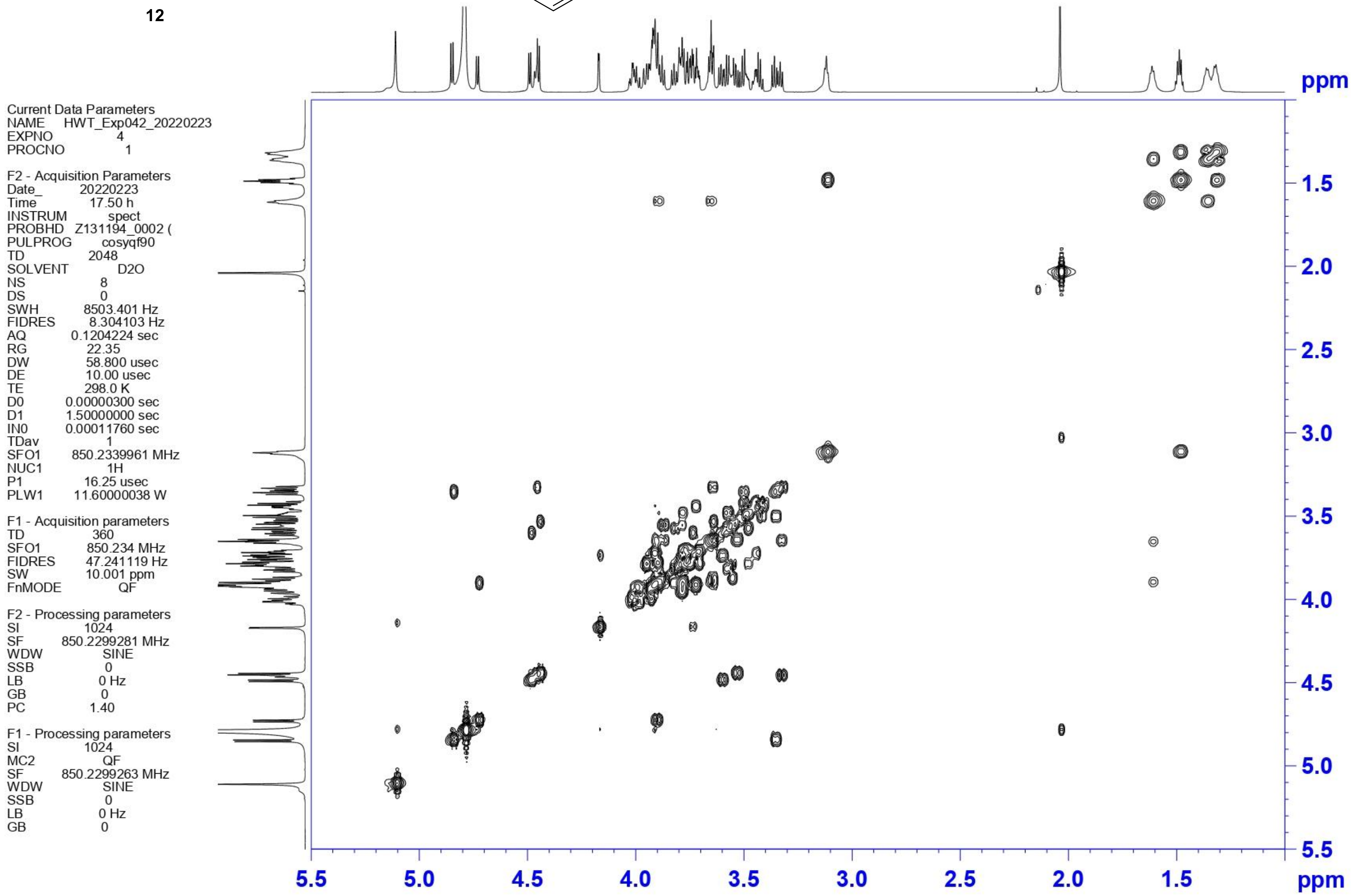

^{1}H-^{1}H COSY NMR spectrum of Compound **12** (850 MHz, D_2O)

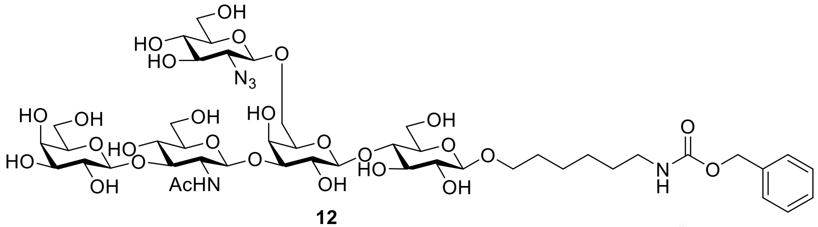

12

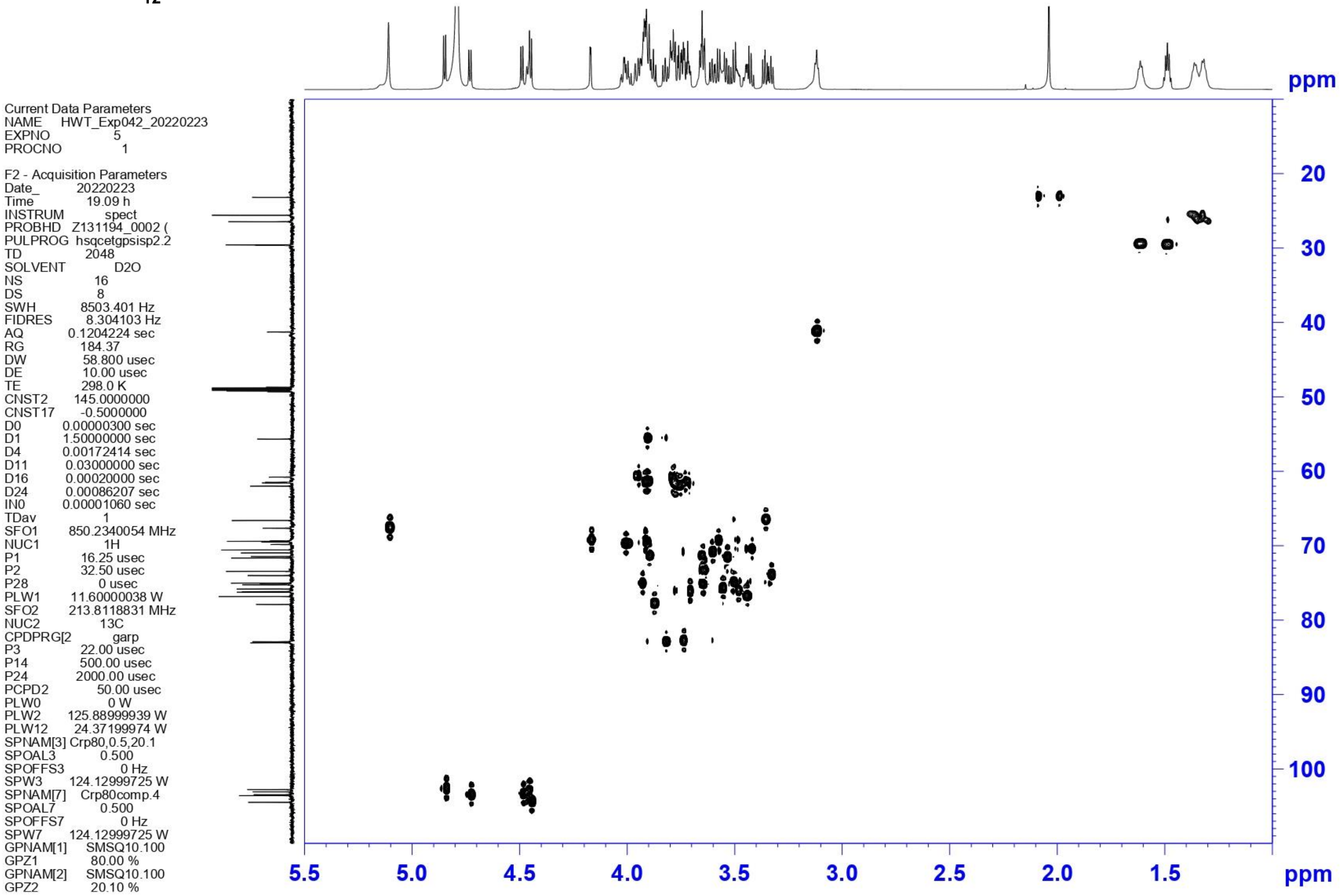

^{1}H-^{13}C HSQC NMR spectrum of Compound **12** (850 MHz/214 MHz D_2O)

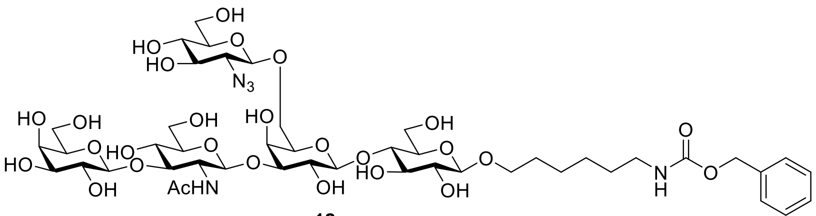

12

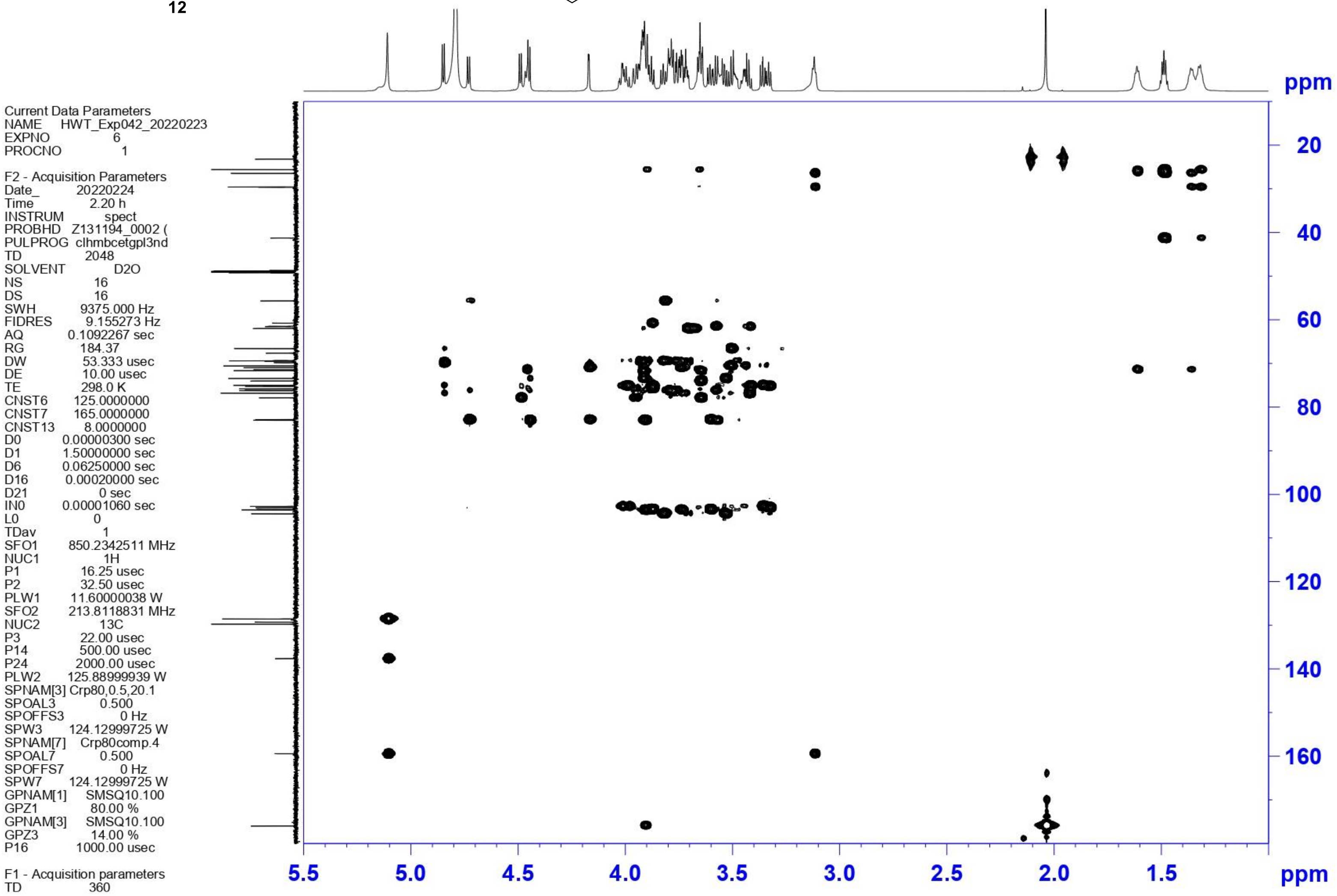

^{1}H-^{13}C HMBC NMR spectrum of Compound **12** (850 MHz/214 MHz D_2O)

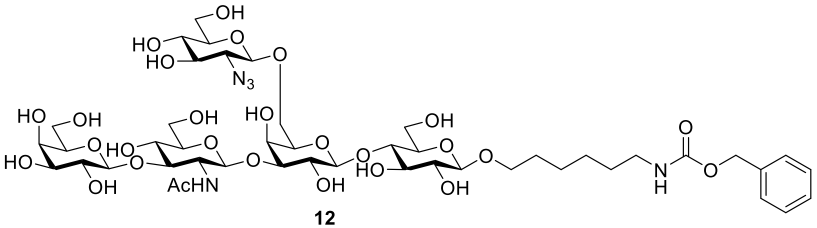

12

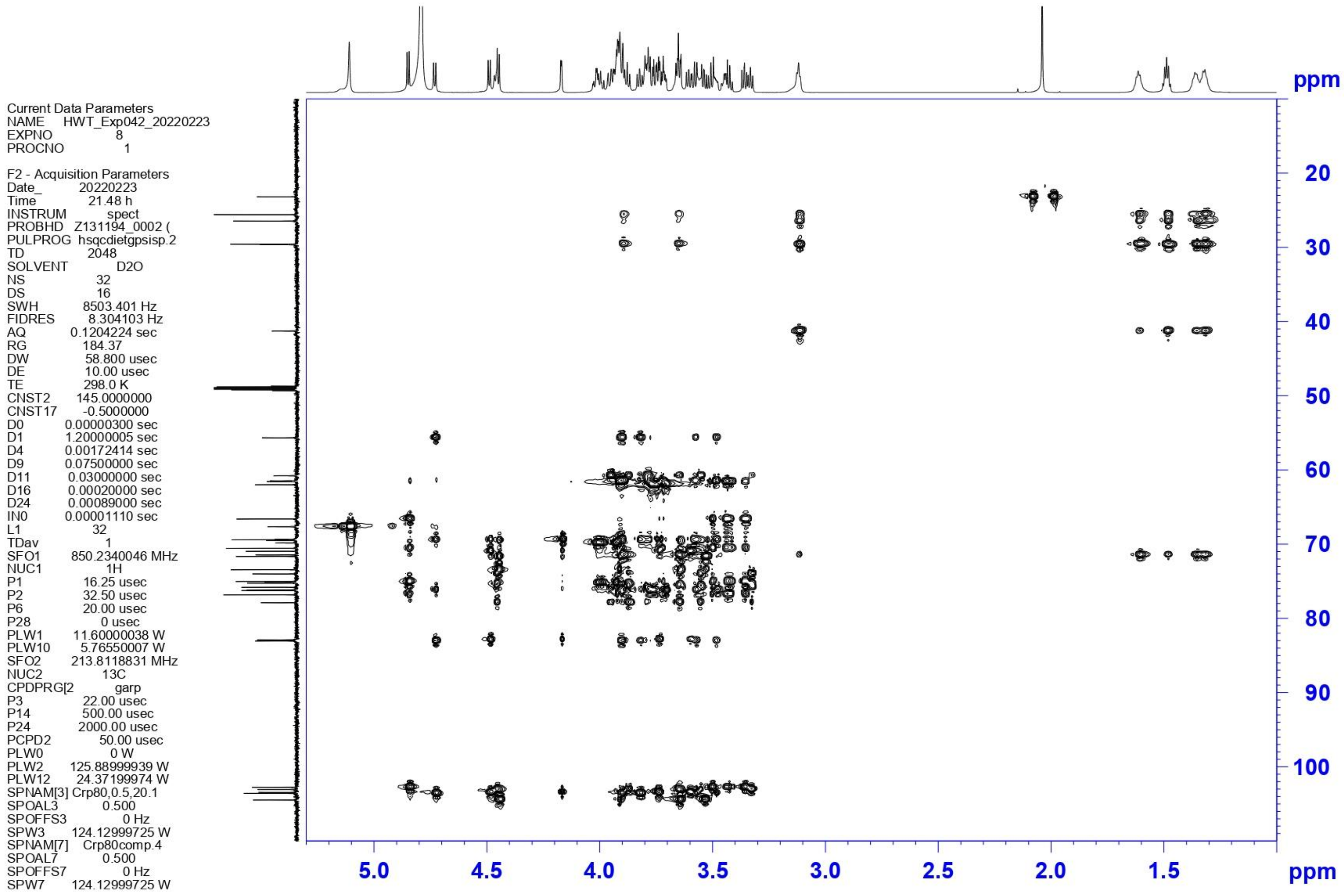

^{1}H-^{13}C HSQC-TOCSY NMR spectrum of Compound **12** (850 MHz/214 MHz D_2O)

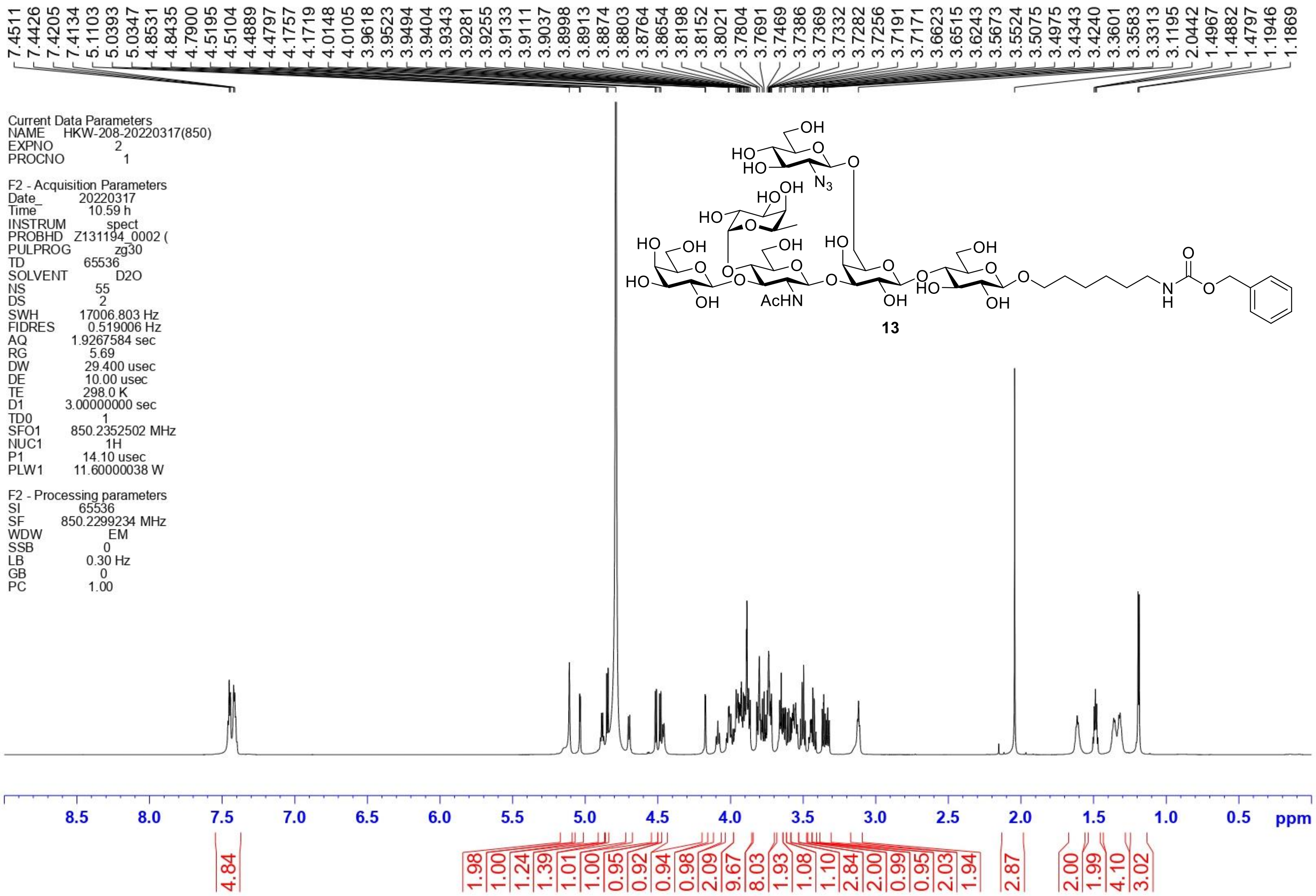

^{1}H NMR spectrum of Compound **13** (850 MHz, D_2O)

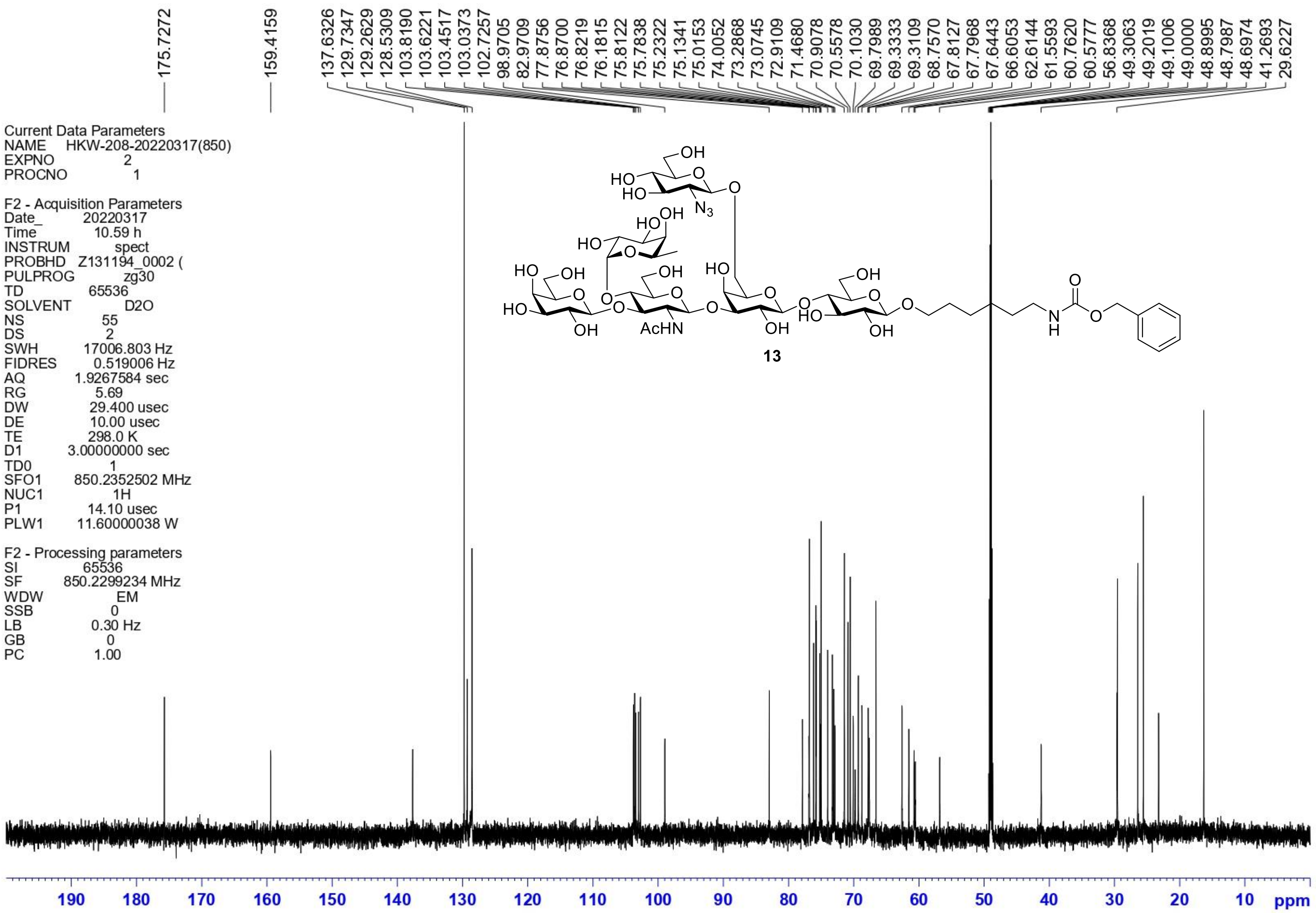

^{13}C NMR spectrum of Compound **13** (214 MHz, D_2O)

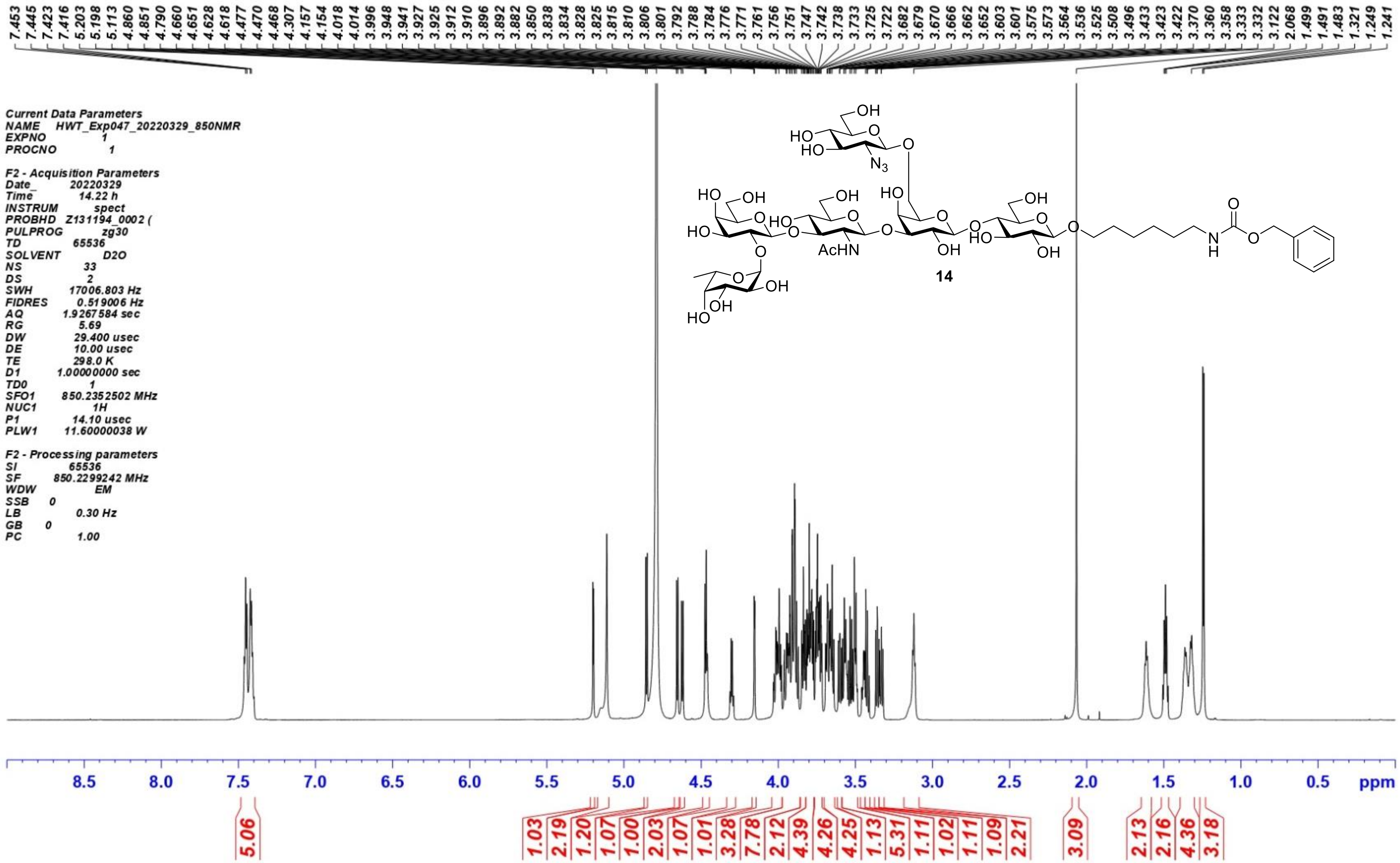

^{1}H NMR spectrum of Compound **14** (850 MHz D_2O)

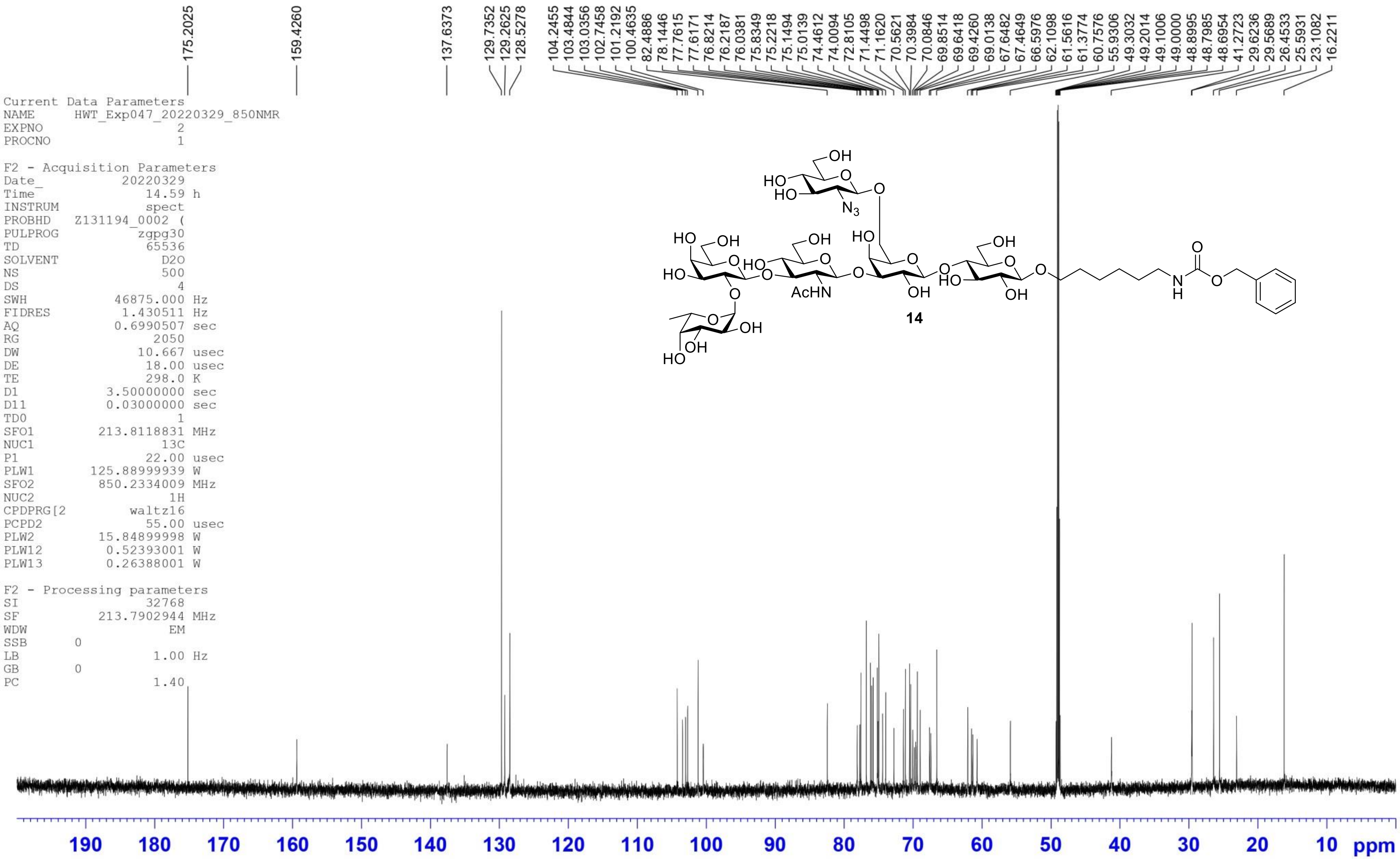

^{13}C NMR spectrum of compound **14** (214 MHz D_2O)

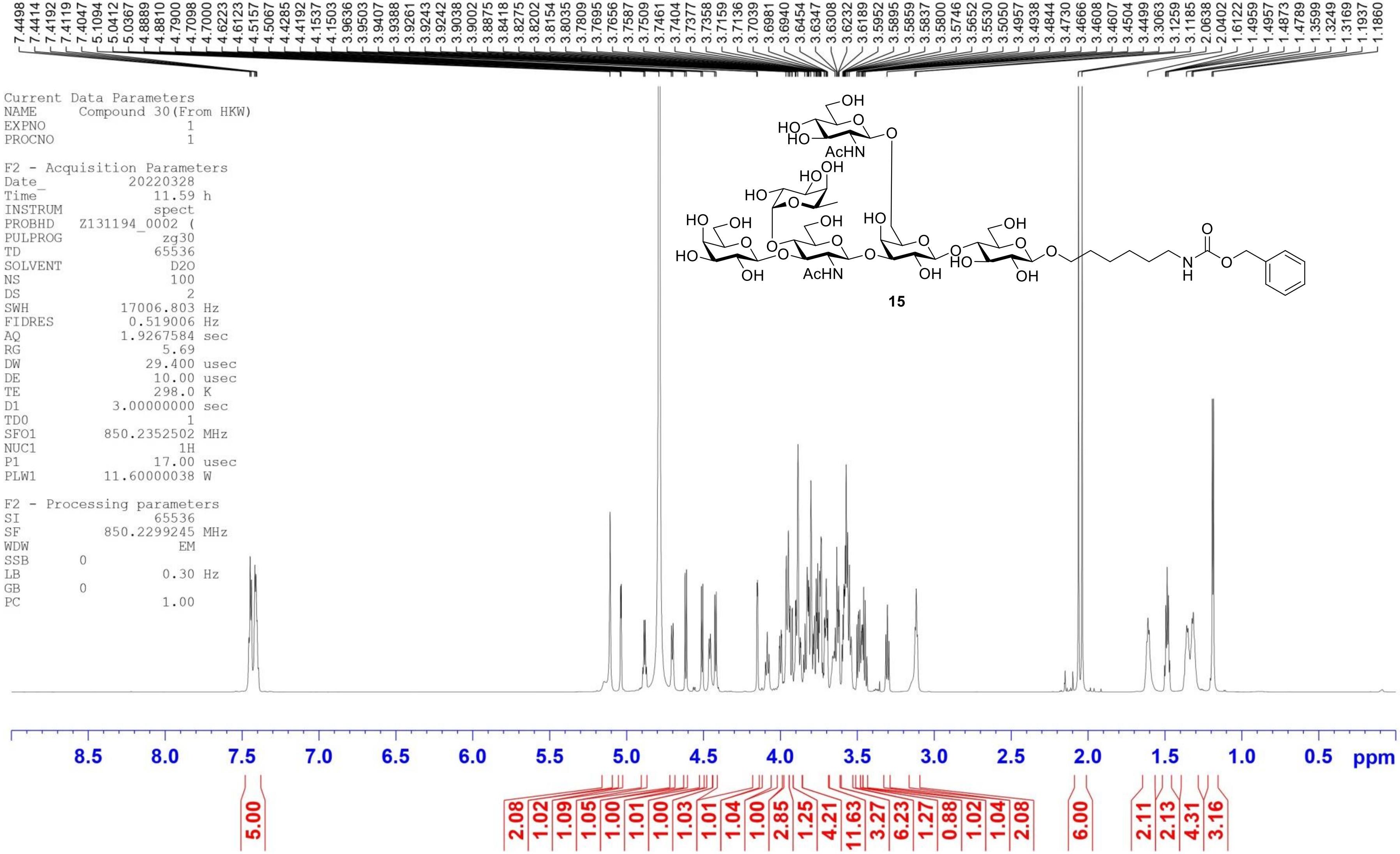

^{1}H NMR spectrum of Compound **15** (850 MHz D_2O)

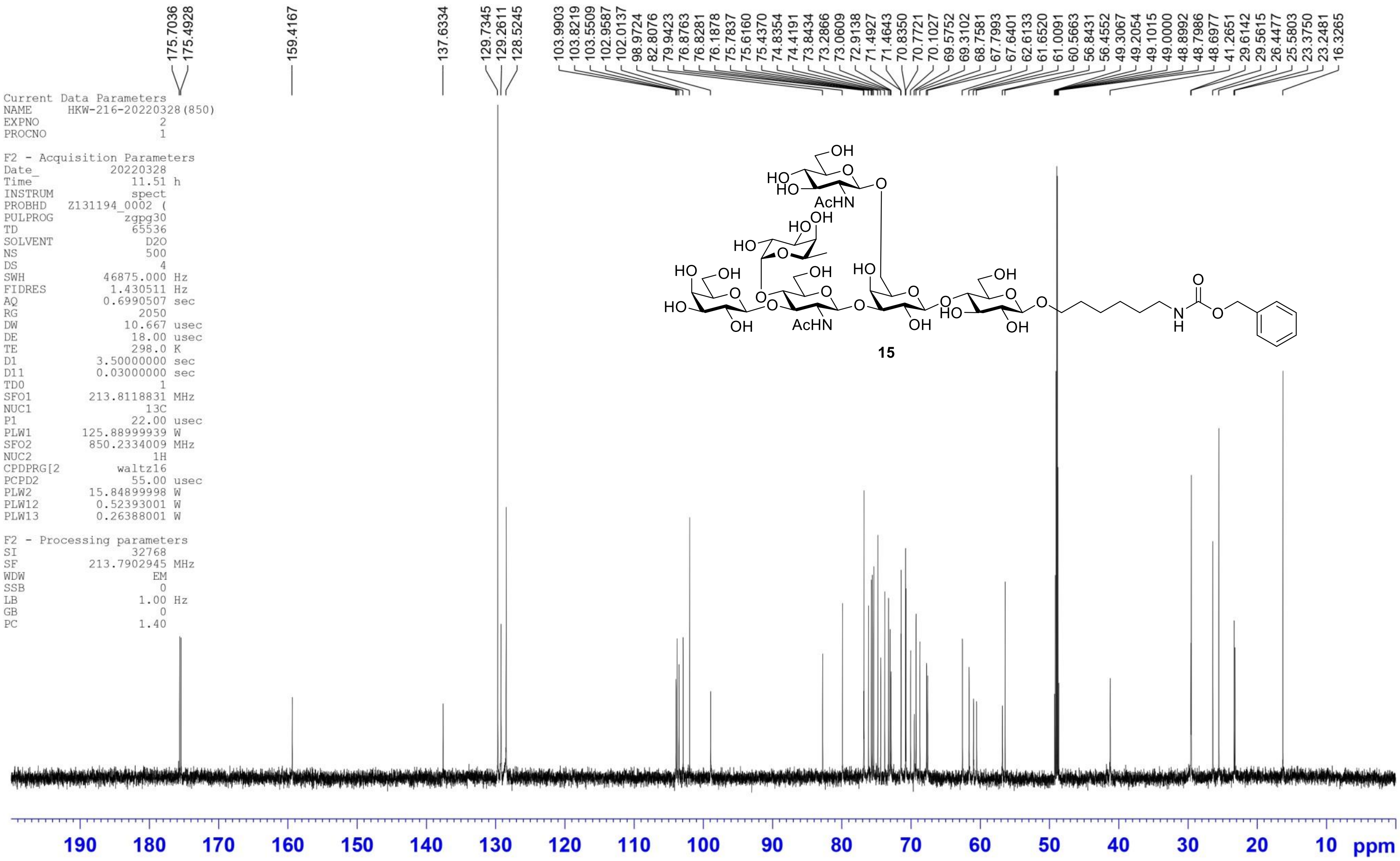

^{13}C NMR spectrum of Compound **15** (214 MHz D_2O)

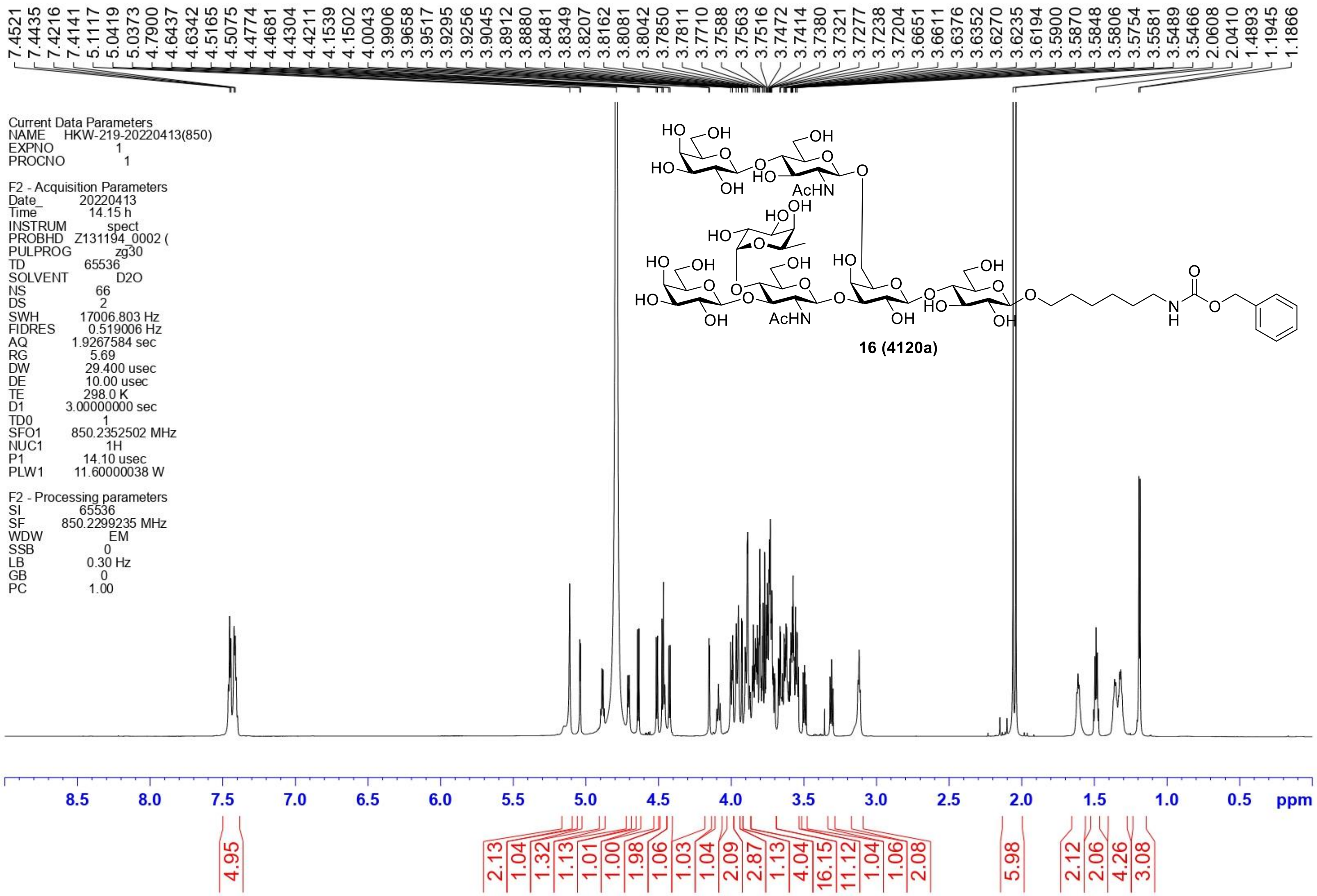

^{1}H NMR spectrum of compound **16** (4120a) (850 MHz, D_2O)

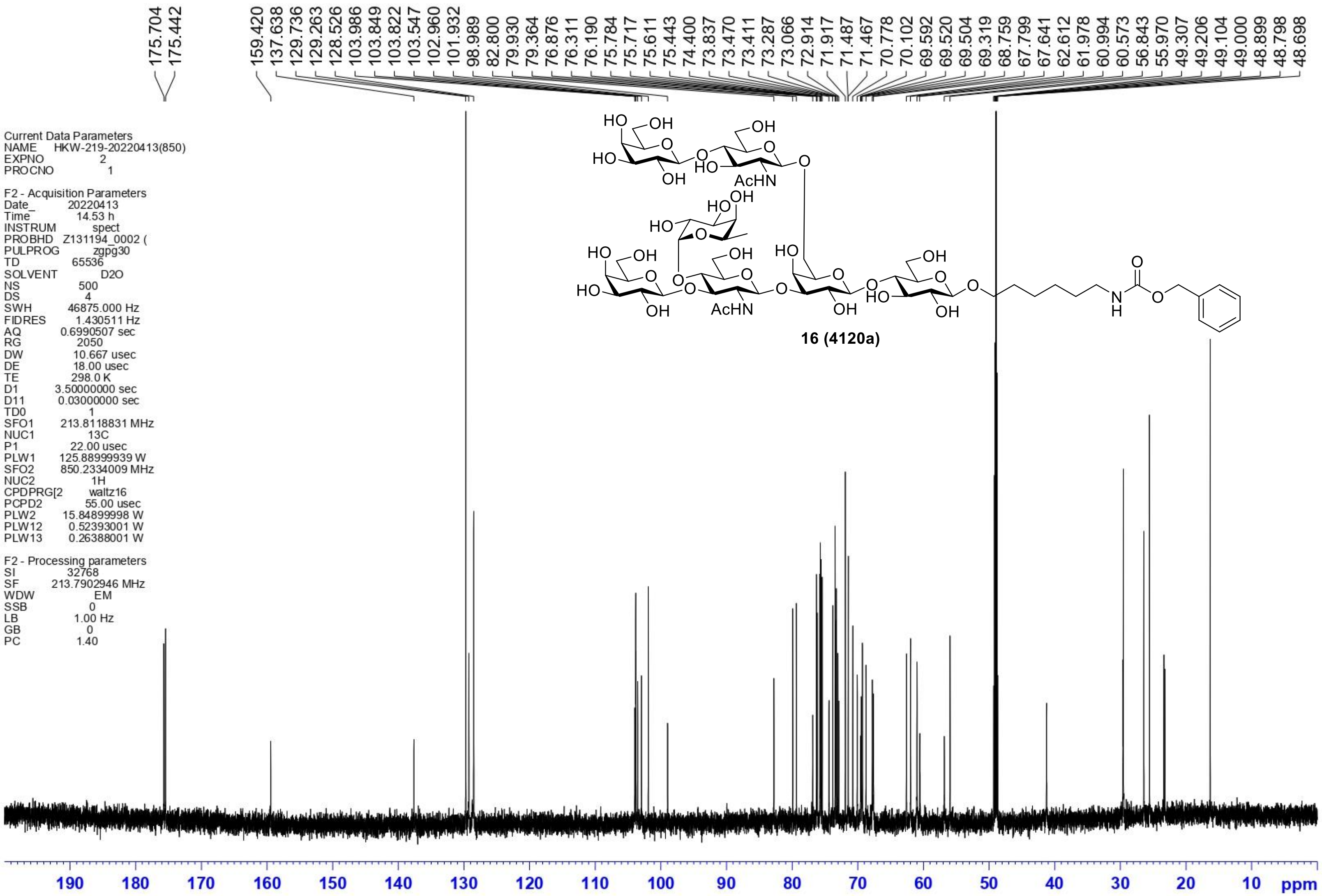

^{13}C NMR spectrum of compound **16** (4120a) (214 MHz, D_2O)

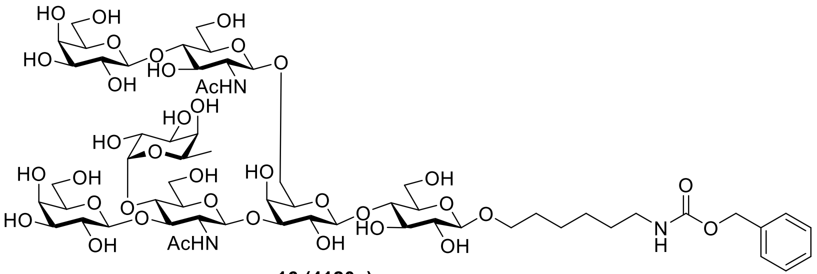

16 (4120a)

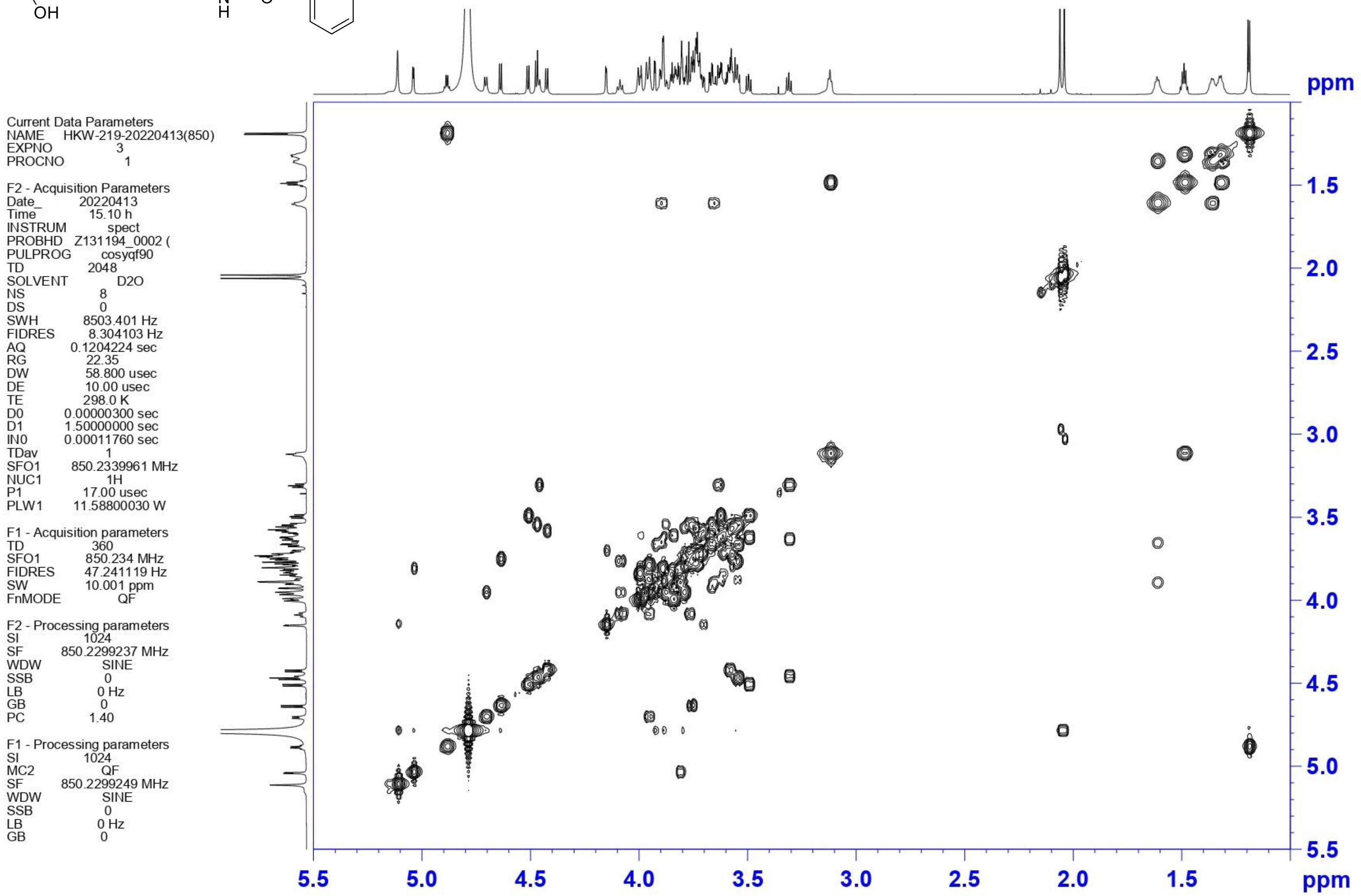

^1H-^1H COSY NMR spectrum of compound **16** (4120a) (850 MHz, D_2O)

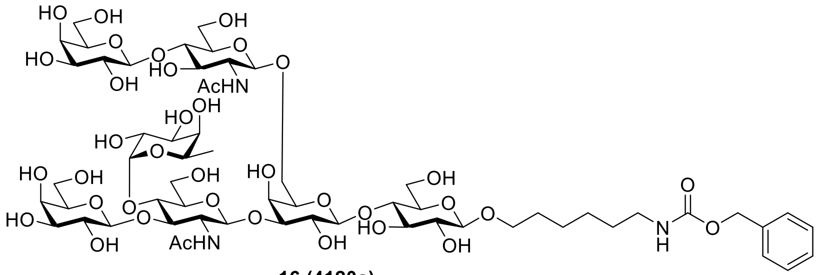

16 (4120a)

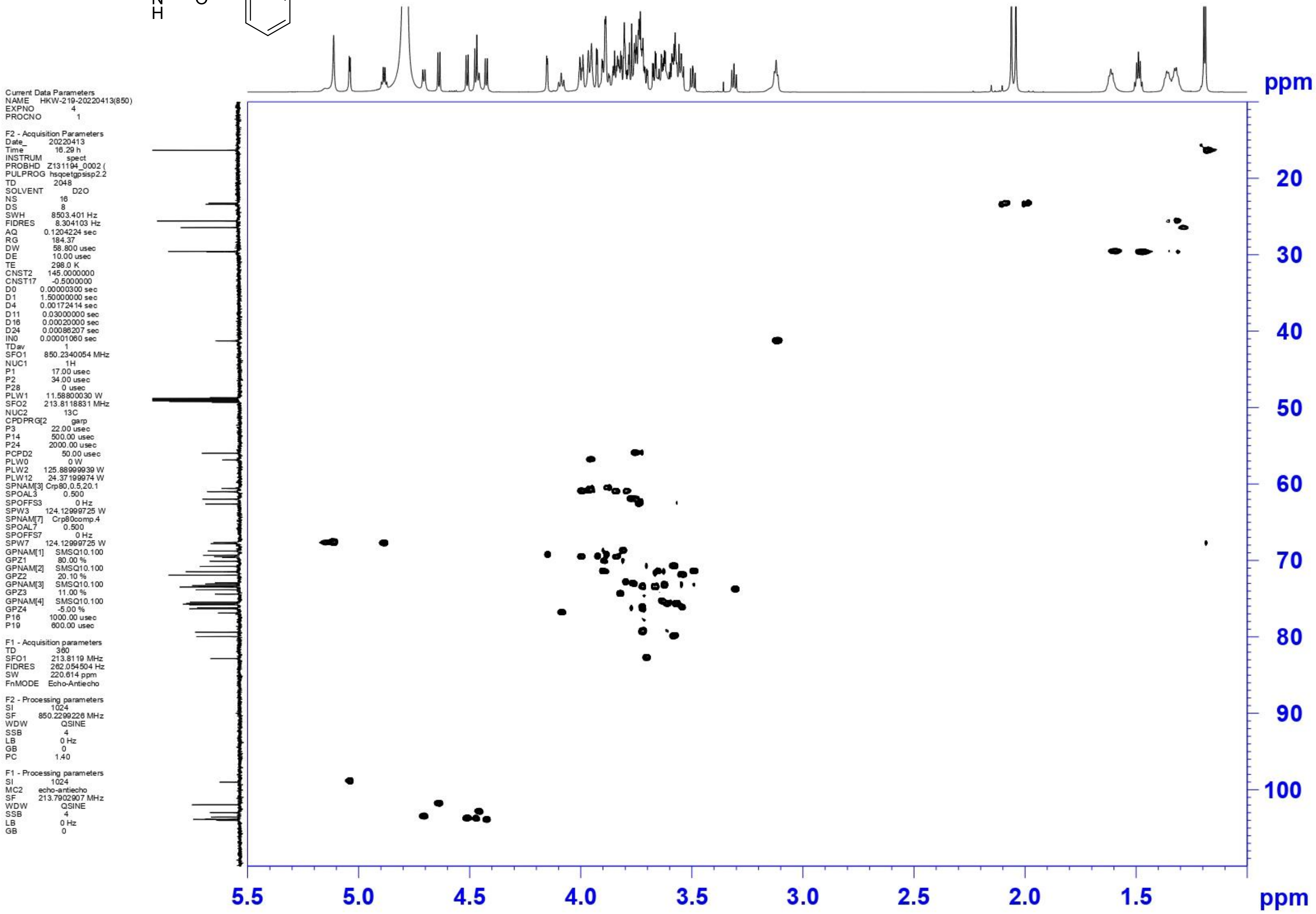

^{1}H-^{13}C HSQC NMR spectrum of Compound **16** (4120a) (850 MHz/214 MHz, D_2O)

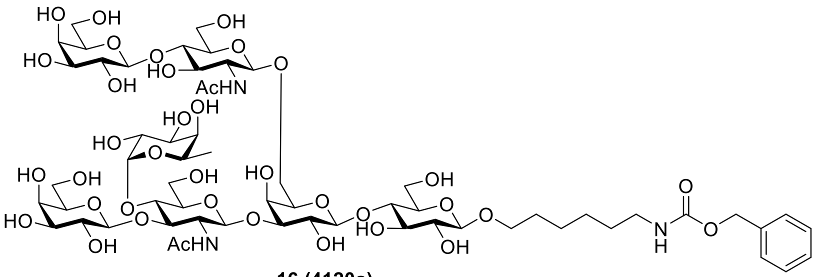

16 (4120a)

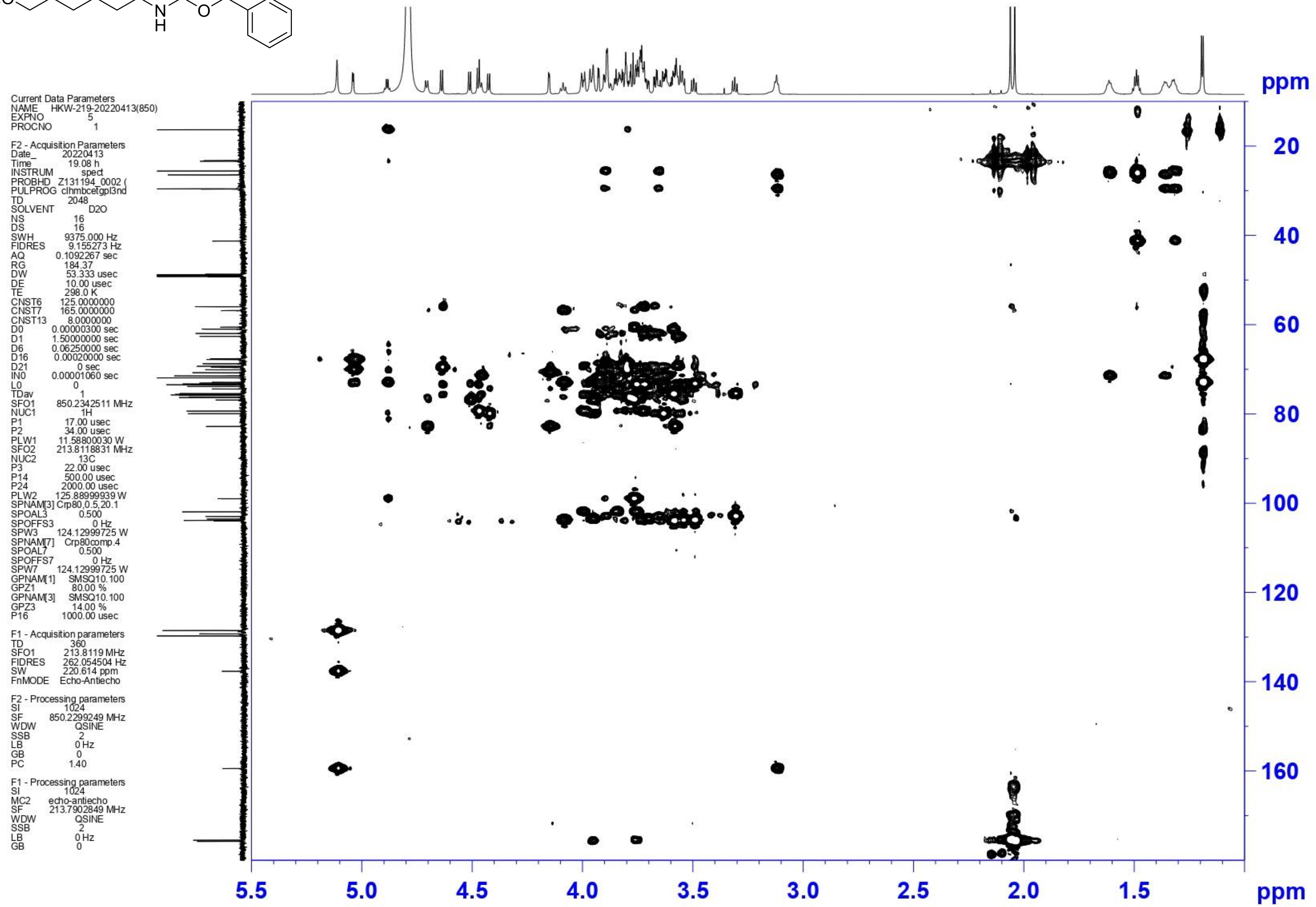

^{1}H-^{13}C HMBC NMR spectrum of Compound **16** (4120a) (850 MHz/214 MHz, D_2O)

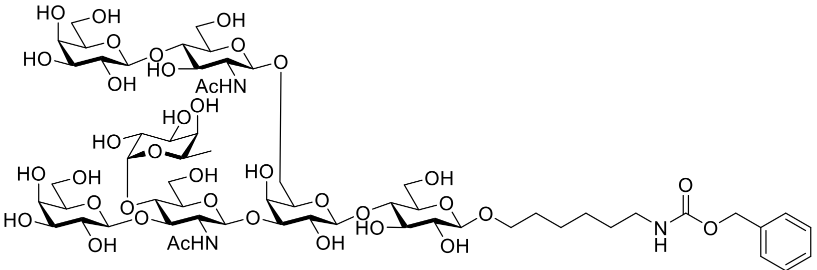

16 (4120a)

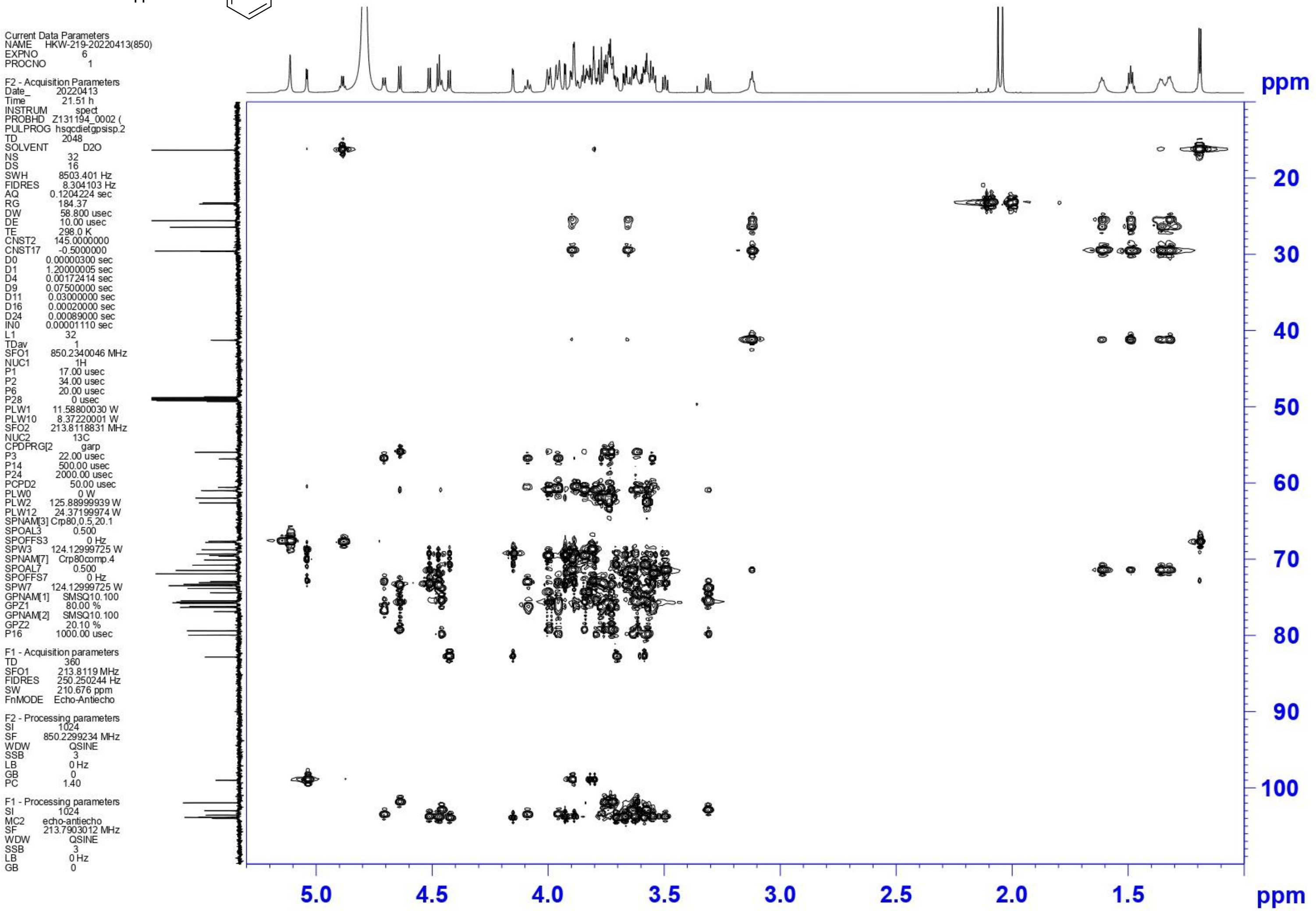

^{1}H-^{13}C HSQC-TOSCY NMR spectrum of compound **16** (4120a) (850 MHz/214 MHz, D_2O)

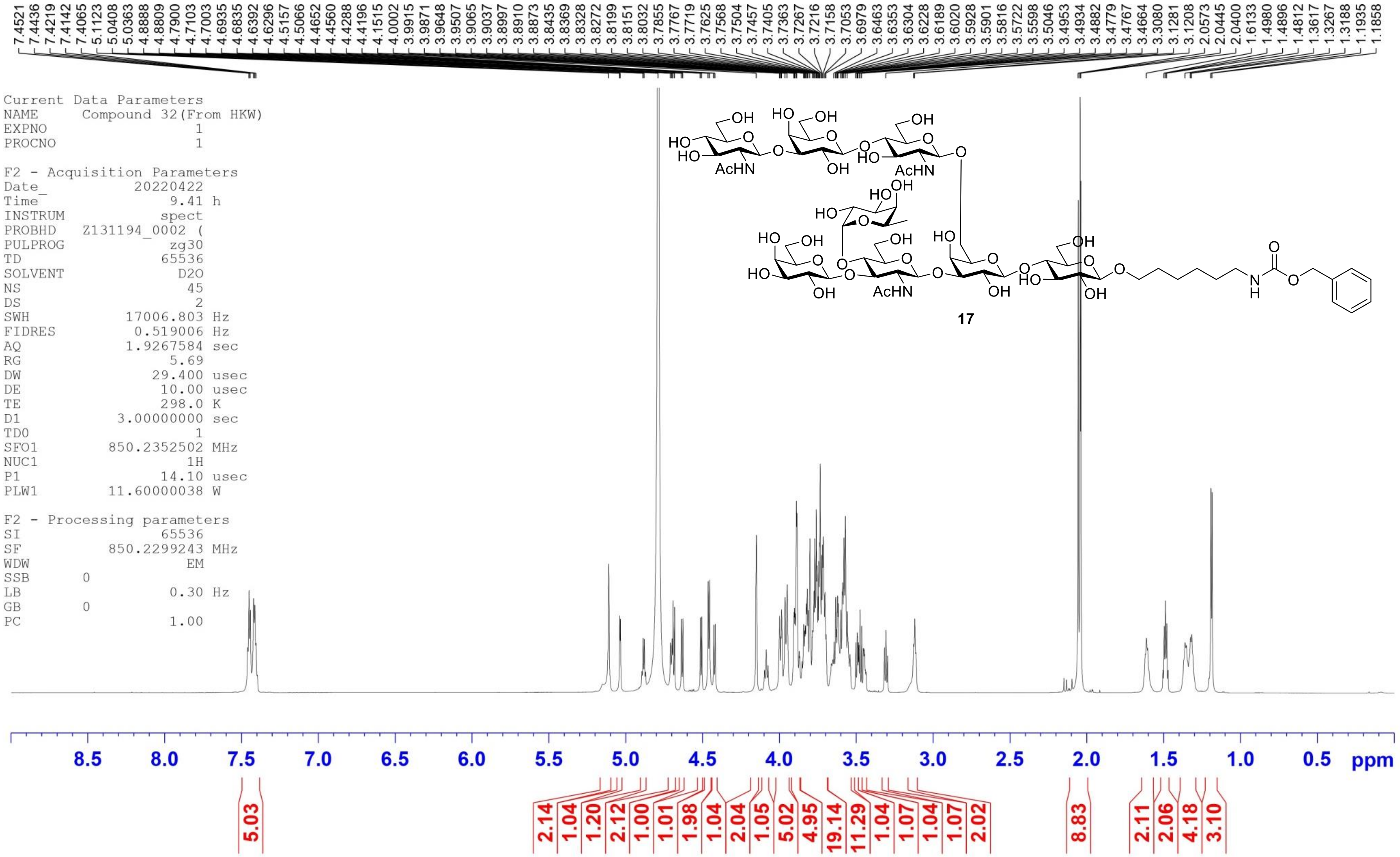

^{1}H NMR spectrum of Compound **17** (850 MHz D_2O)

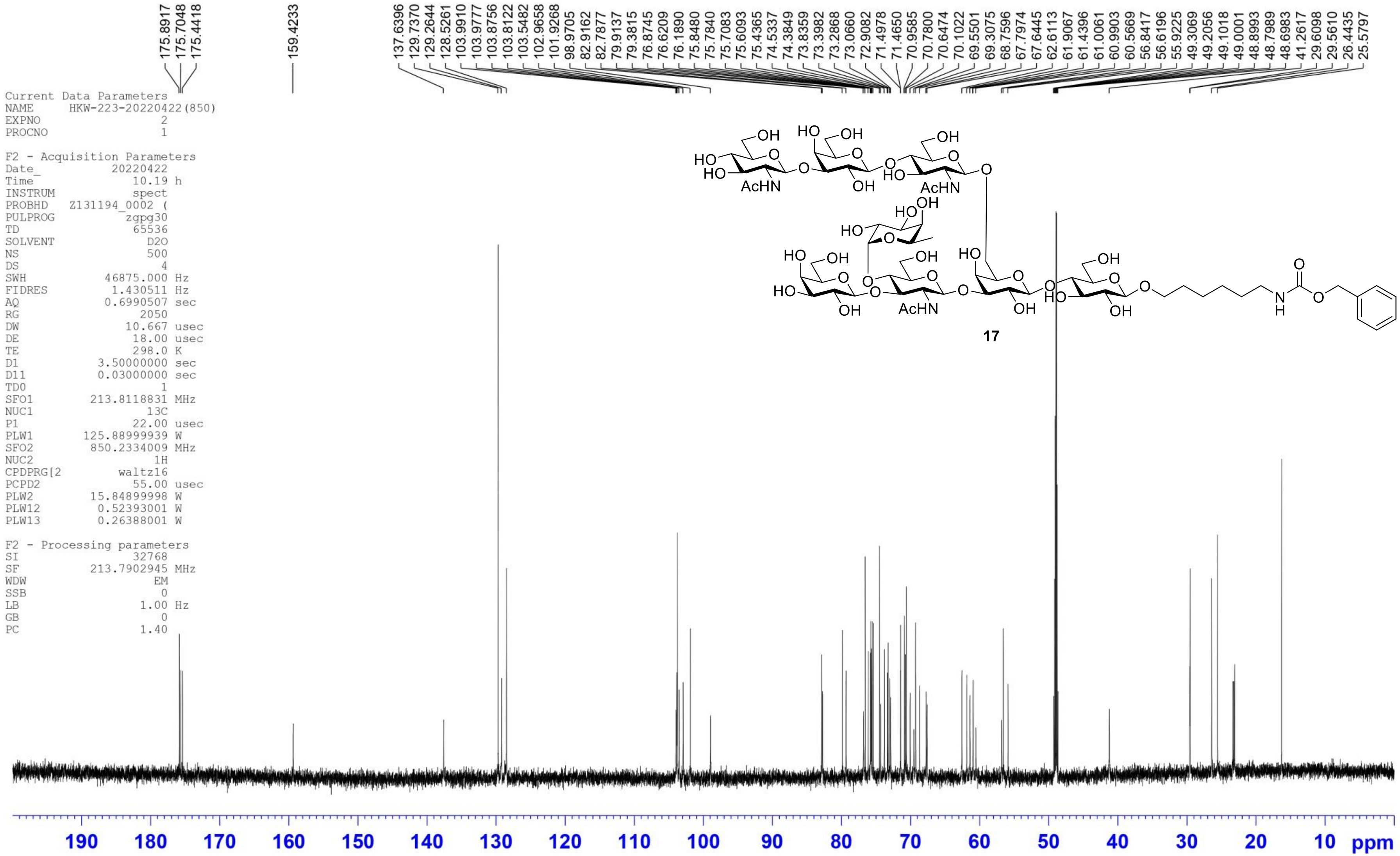

^{13}C NMR spectrum of Compound **17** (214 MHz D_2O)

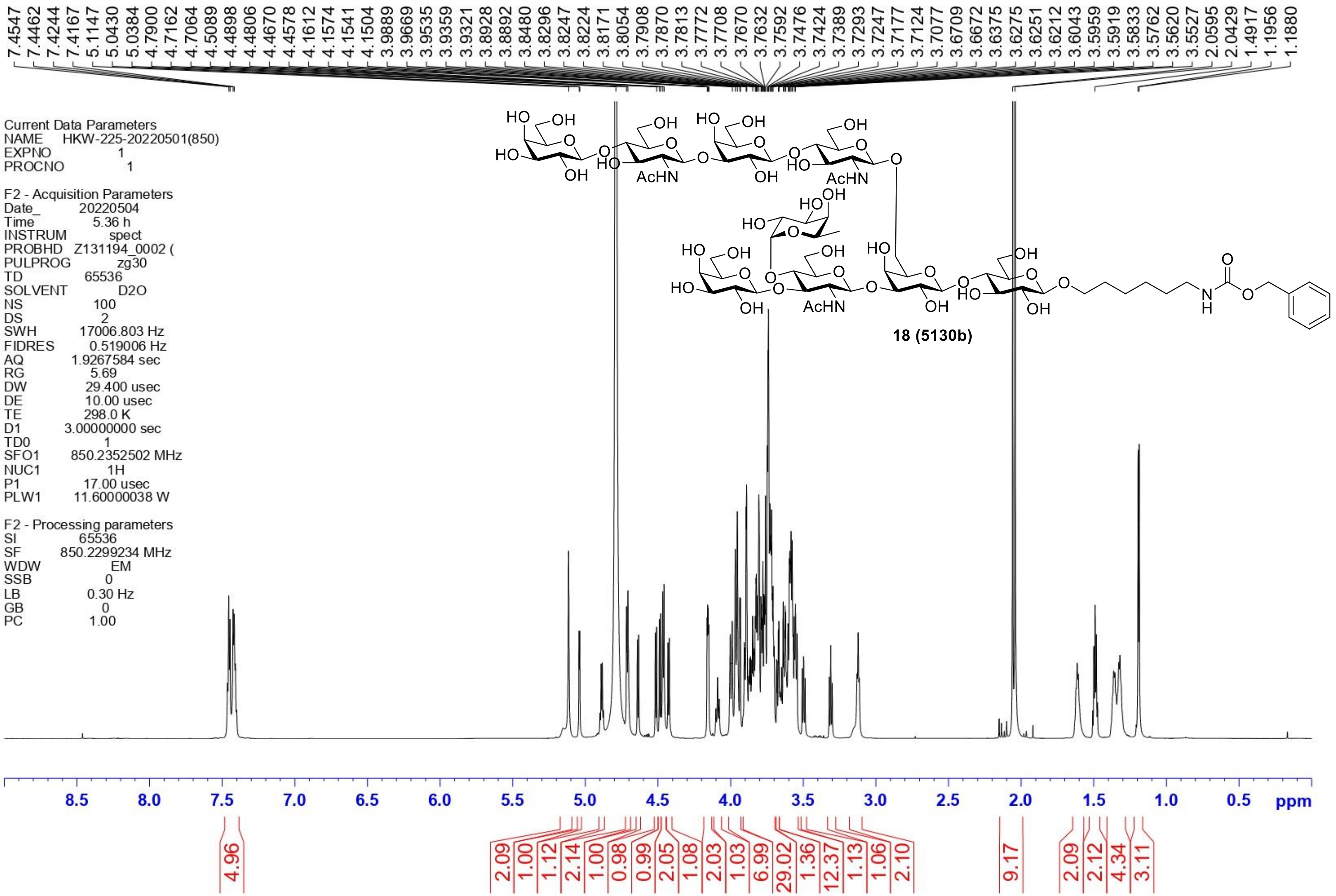

^{1}H NMR spectrum of **18** (5130b) (850 MHz, D_2O)

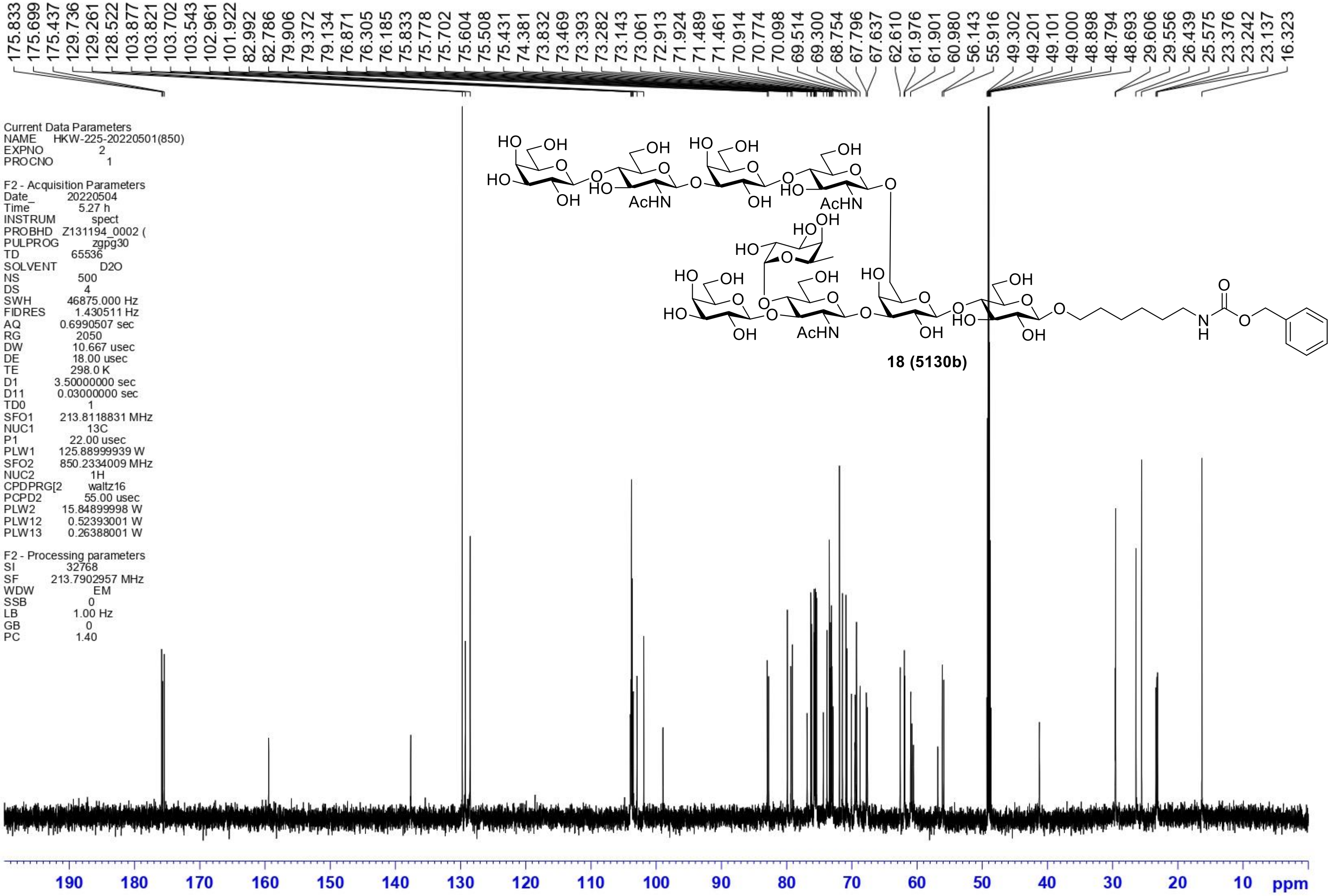

^{13}C NMR spectrum of **18** (5130b) (214 MHz, D_2O)

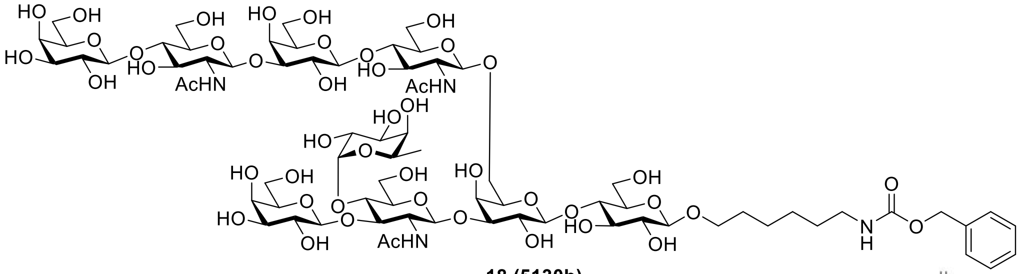

18 (5130b)

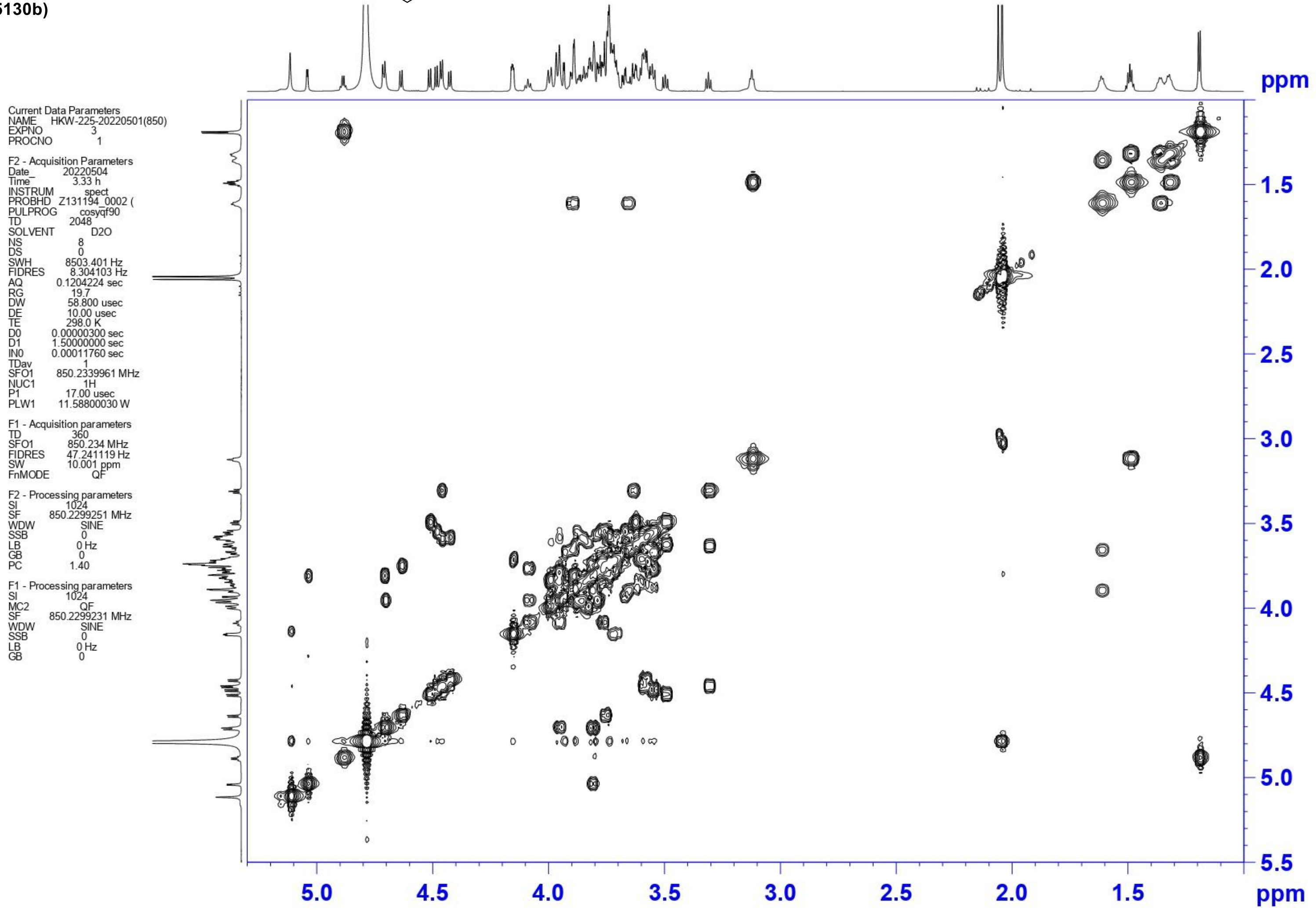

^{1}H-^{1}H COSY NMR spectrum of **18** (5130b) (850 MHz, D_2O)

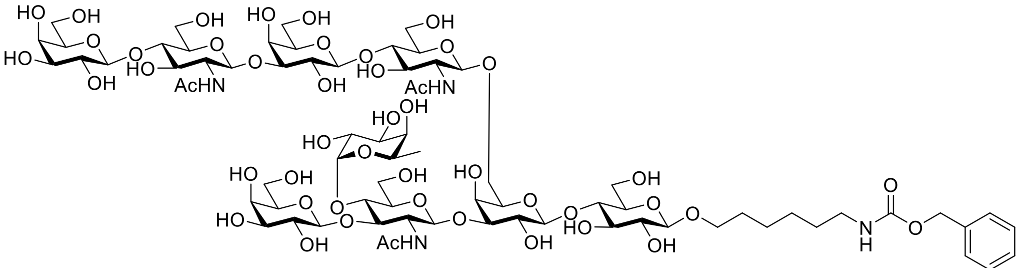

18 (5130b)

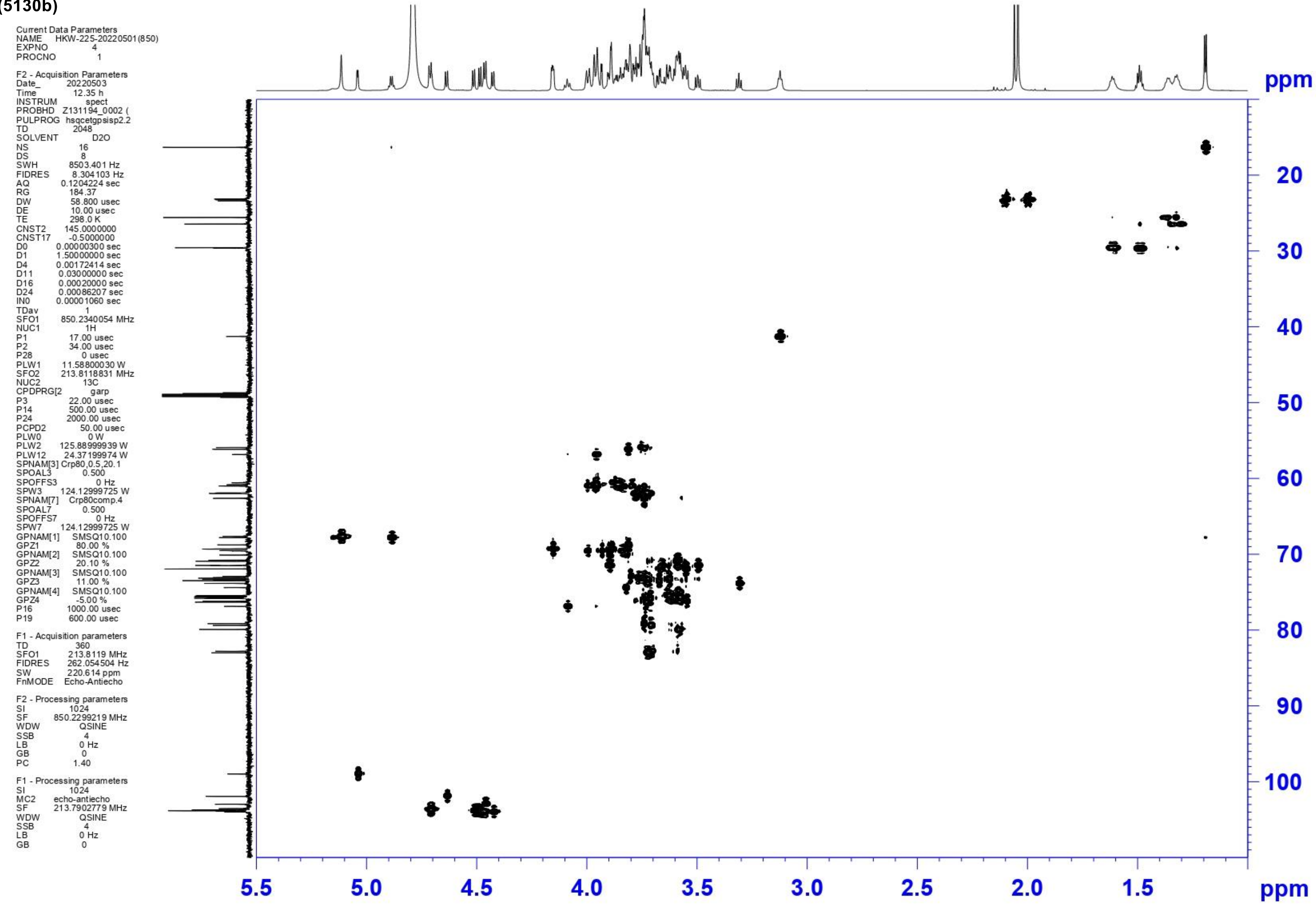

^{1}H-^{13}C HSQC NMR spectrum of **18** (5130b) (850 MHz/214 MHz, D_2O)

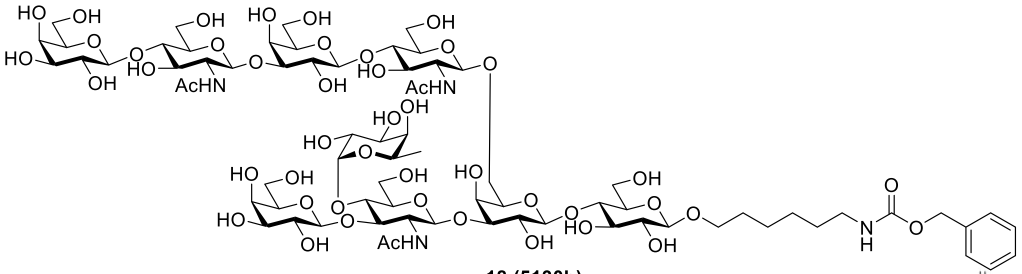

18 (5130b)

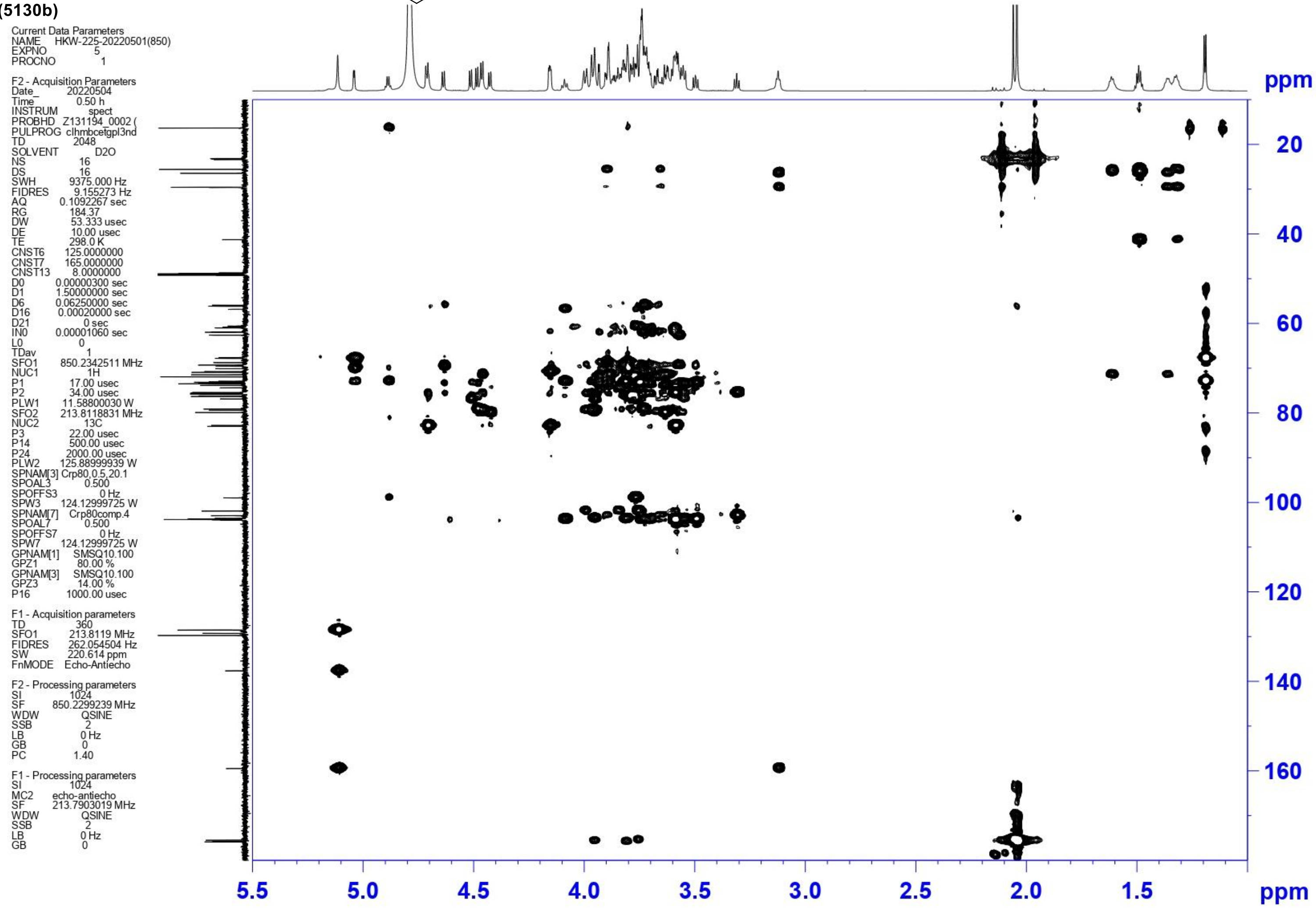

^{1}H-^{13}C HMBC NMR spectrum of **18** (5130b) (850 MHz/214 MHz, D_2O)

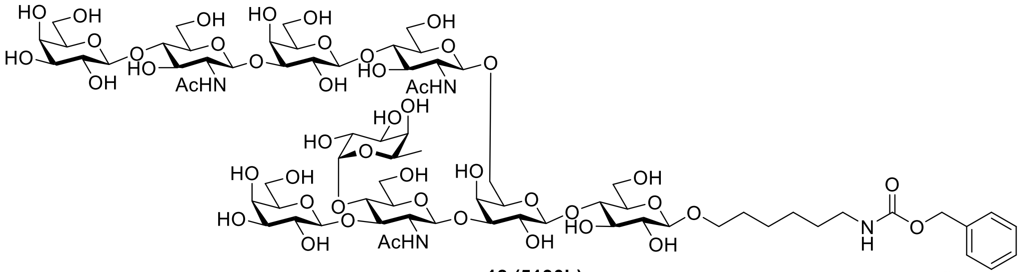

18 (5130b)

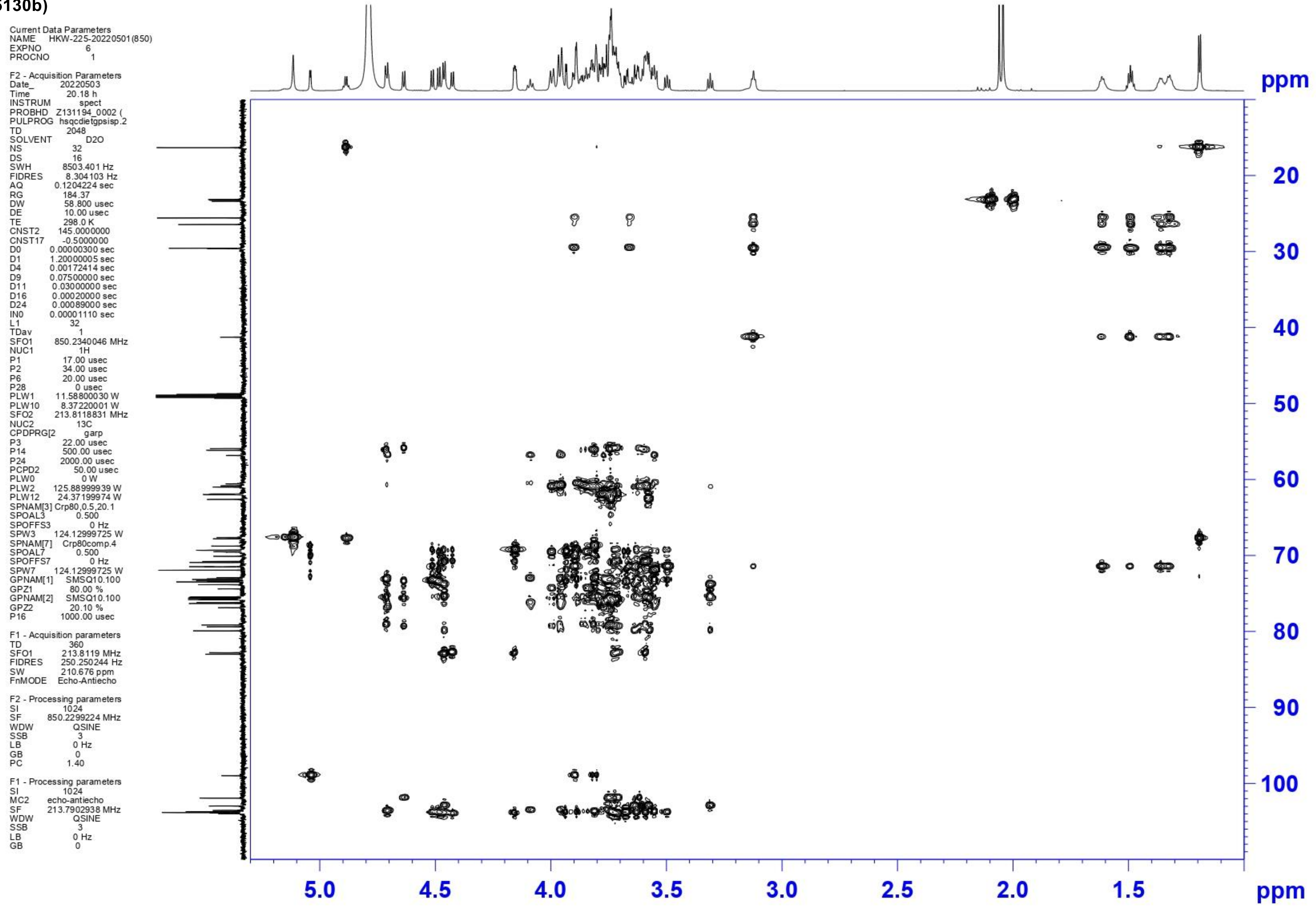

^{1}H-^{13}C HSQC-TOSCY NMR spectrum of **18** (5130b) (850 MHz/214 MHz, D_2O)

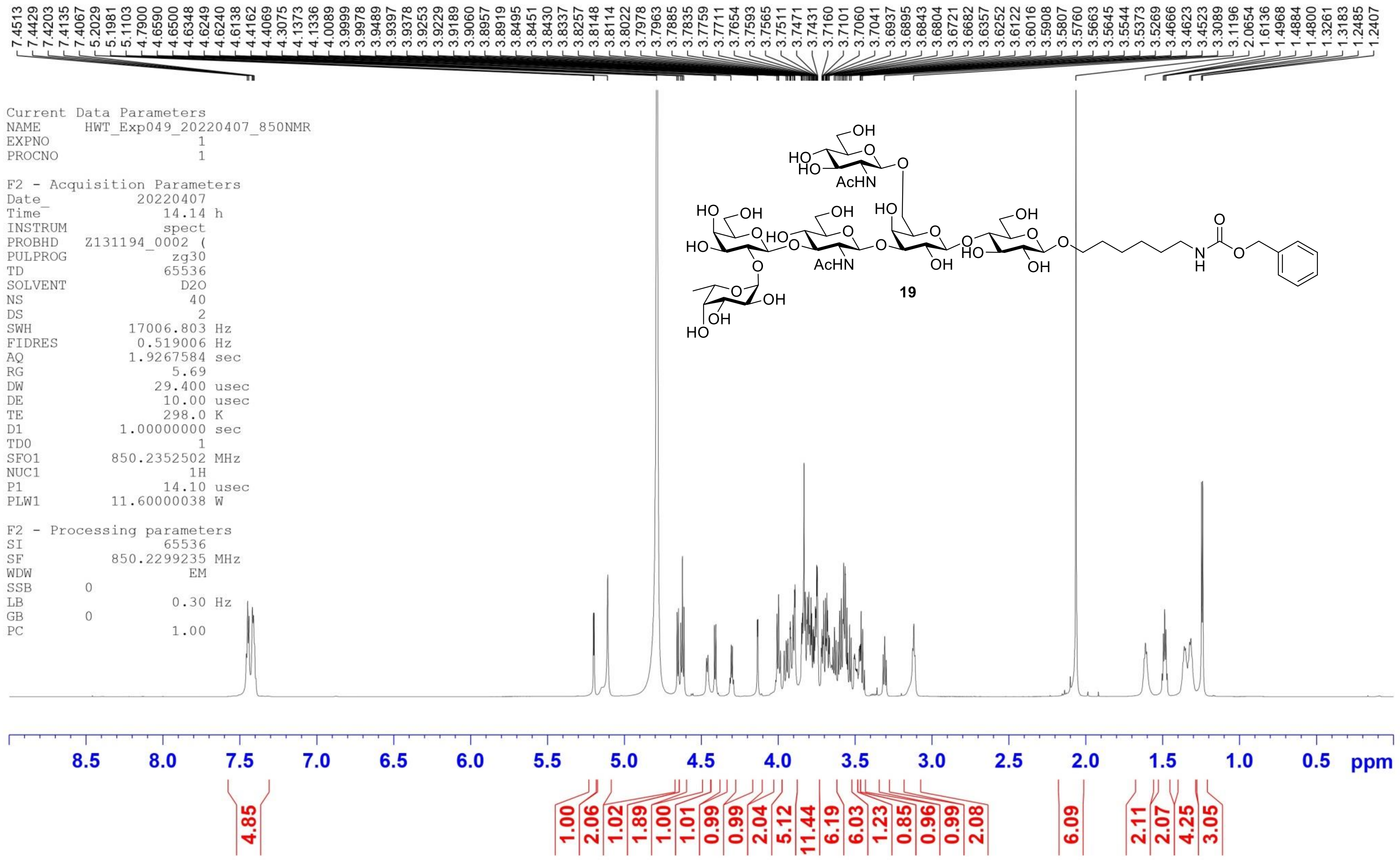

^{1}H NMR spectrum of Compound **19** (850 MHz D_2O)

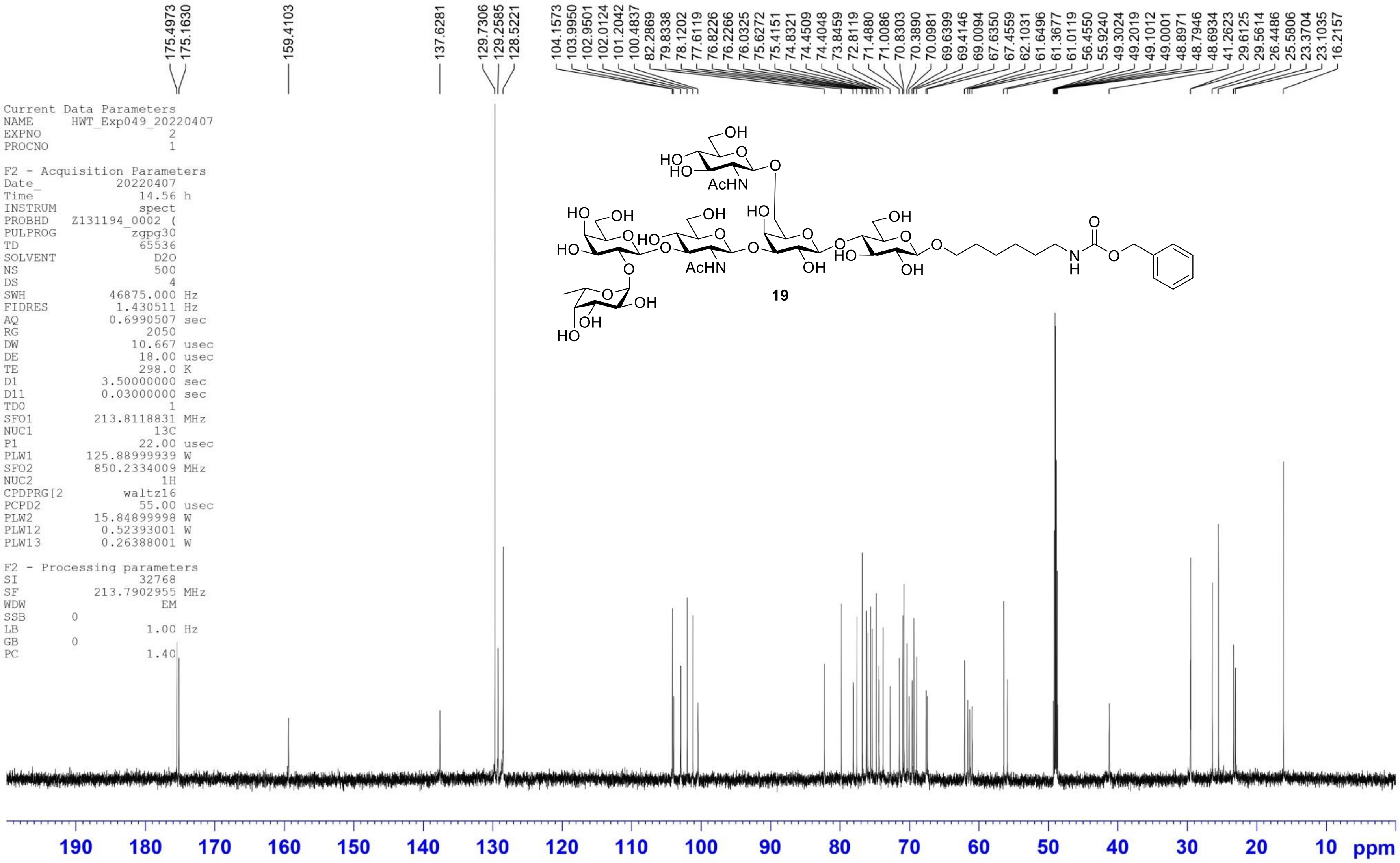

^{13}C NMR spectrum of Compound **19** (214 MHz D_2O)

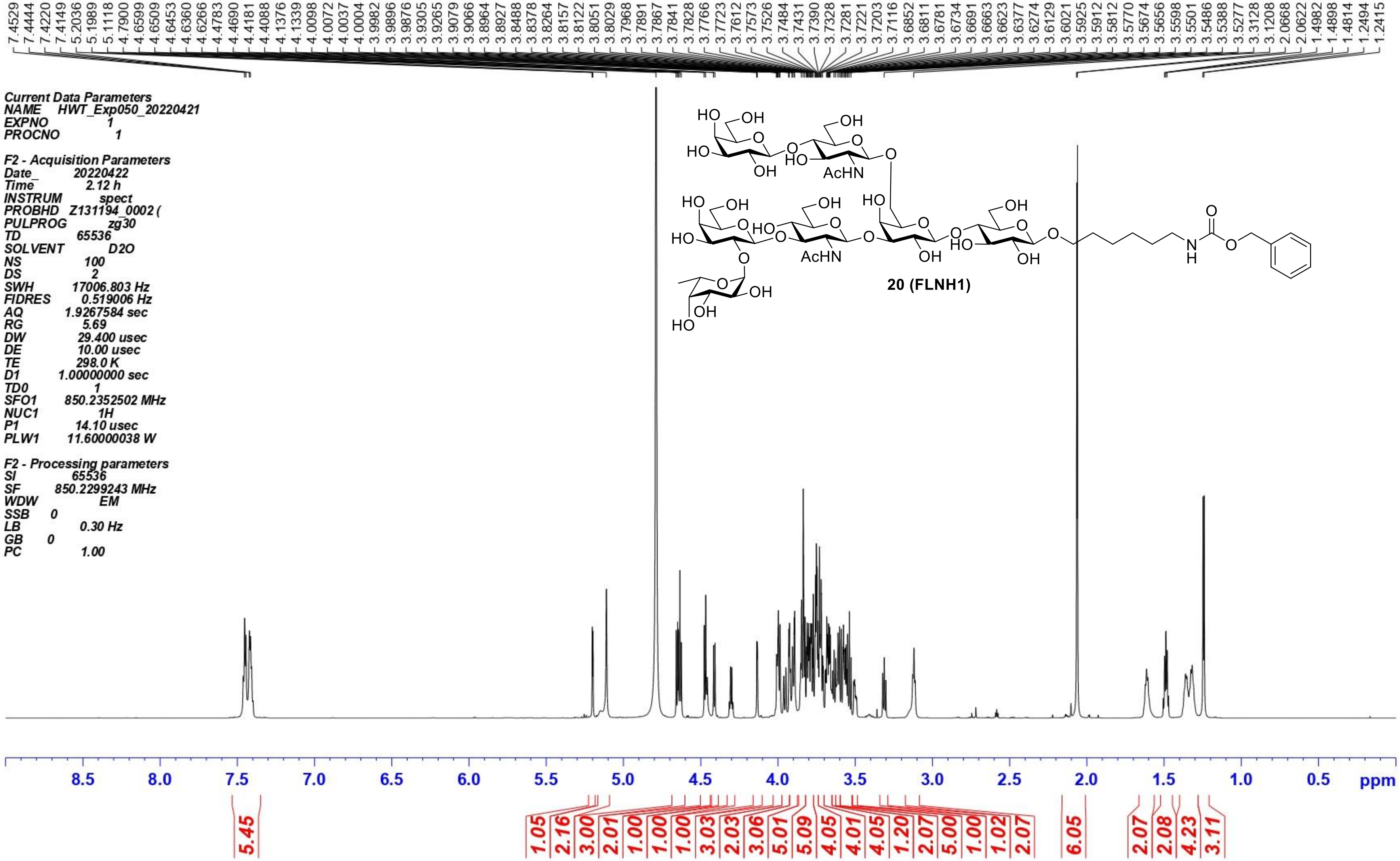

^{1}H NMR spectrum of **20** (FLNH1) (850 MHz, D_2O)

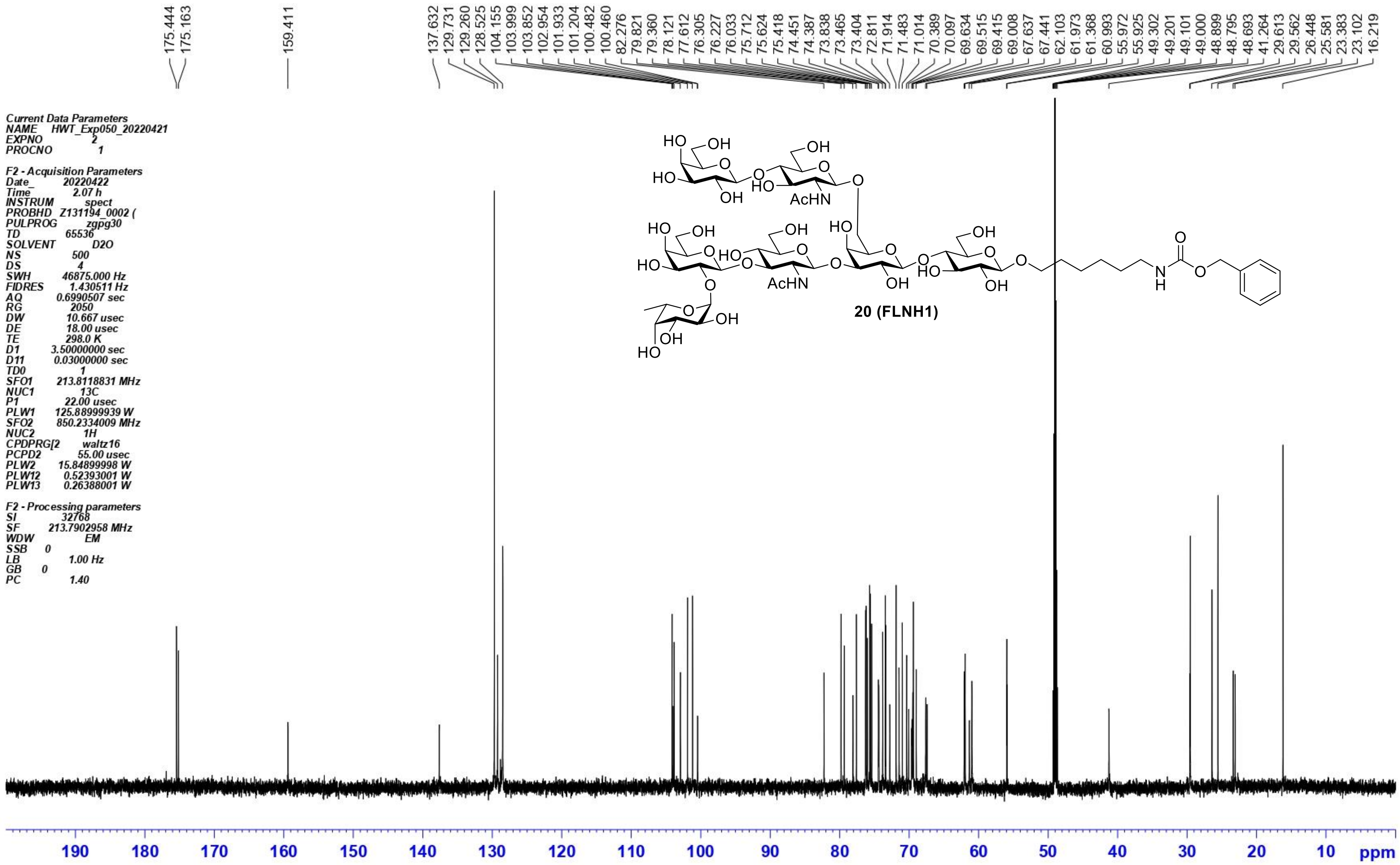

^{13}C NMR spectrum of **20** (FLNH1) (214 MHz, D_2O)

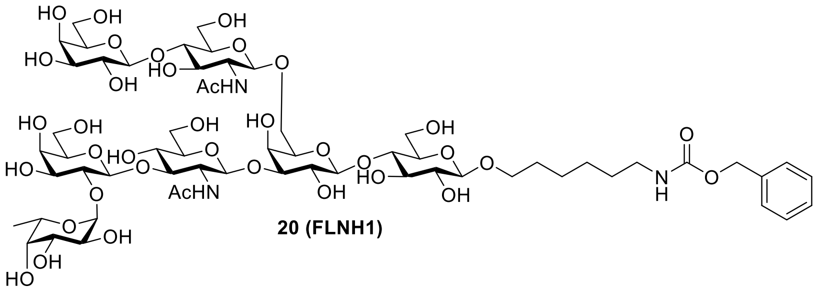

20 (FLNH1)

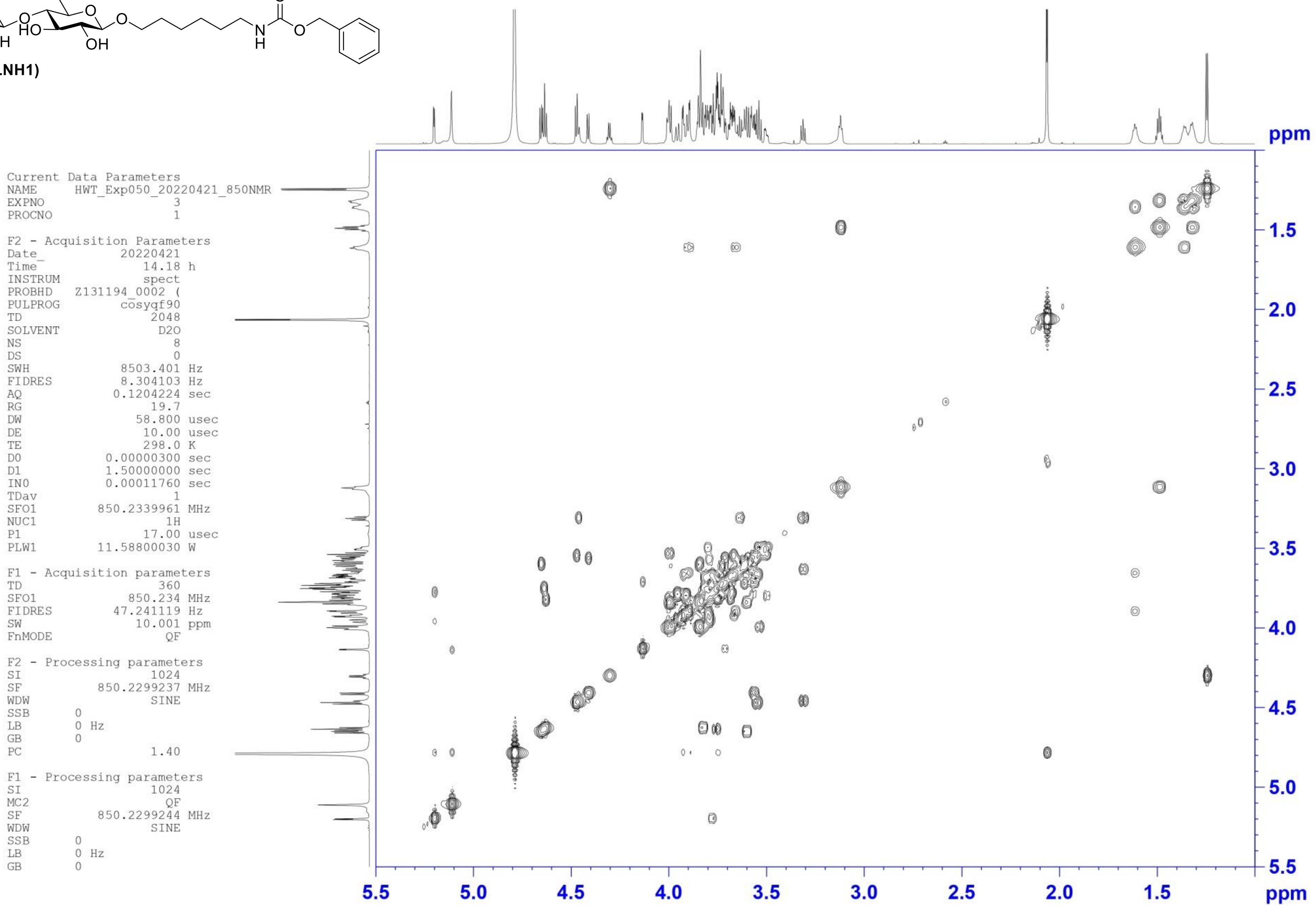

^1H-^1H COSY NMR spectrum of **20** (FLNH1) (850 MHz, D_2O)

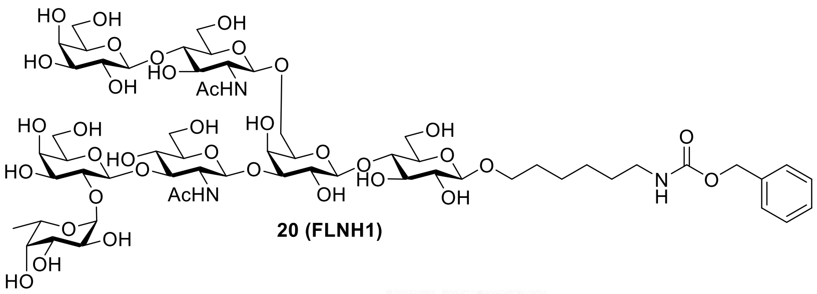

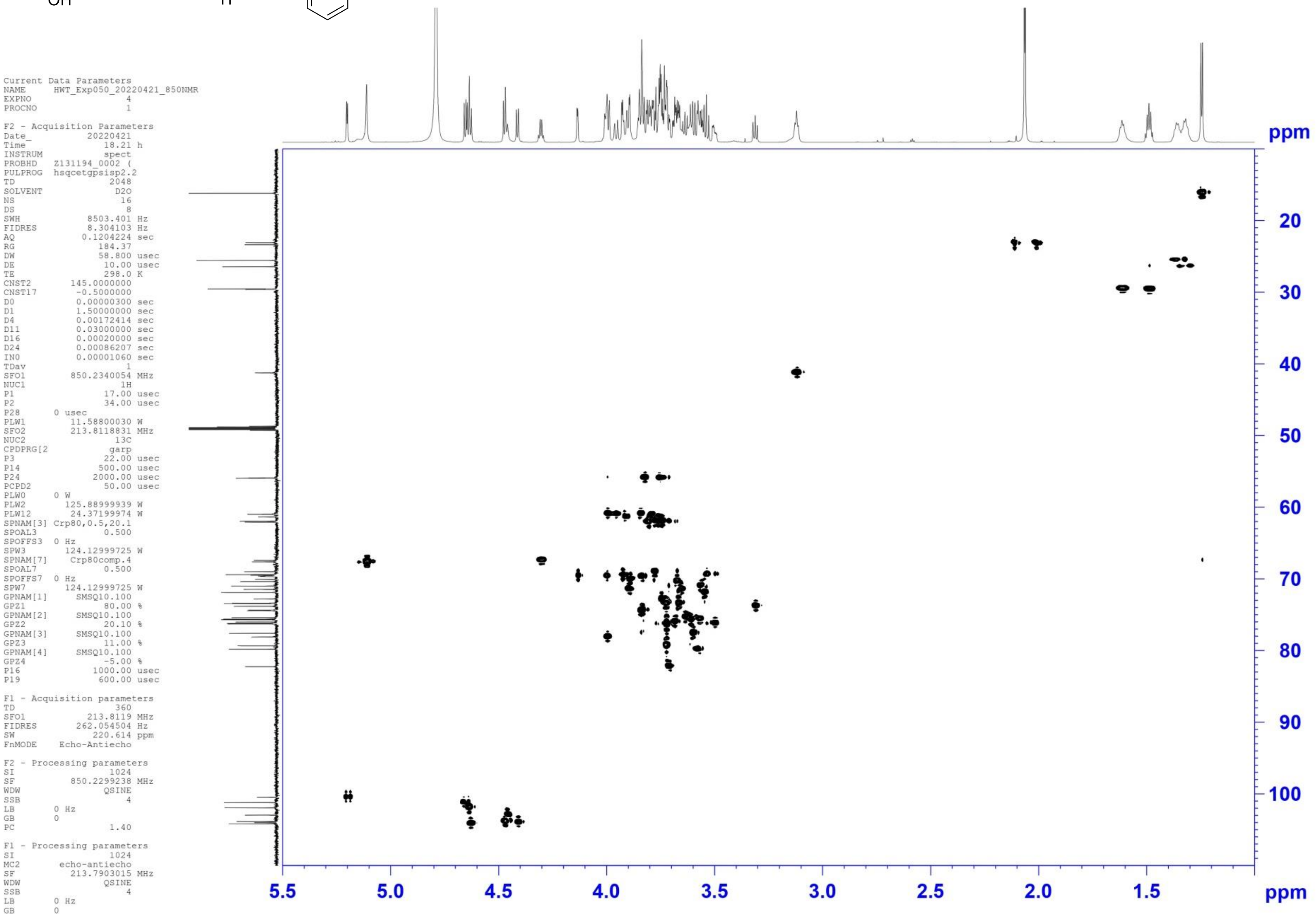

^{1}H-^{13}C HSQC NMR spectrum of **20** (FLNH1) (850 MHz/214 MHz, D_2O)

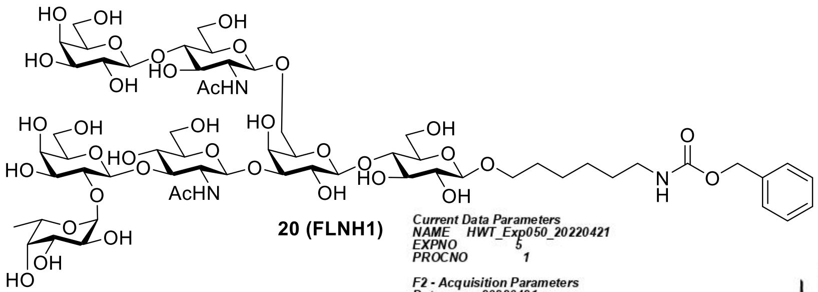

20 (FLNH1)

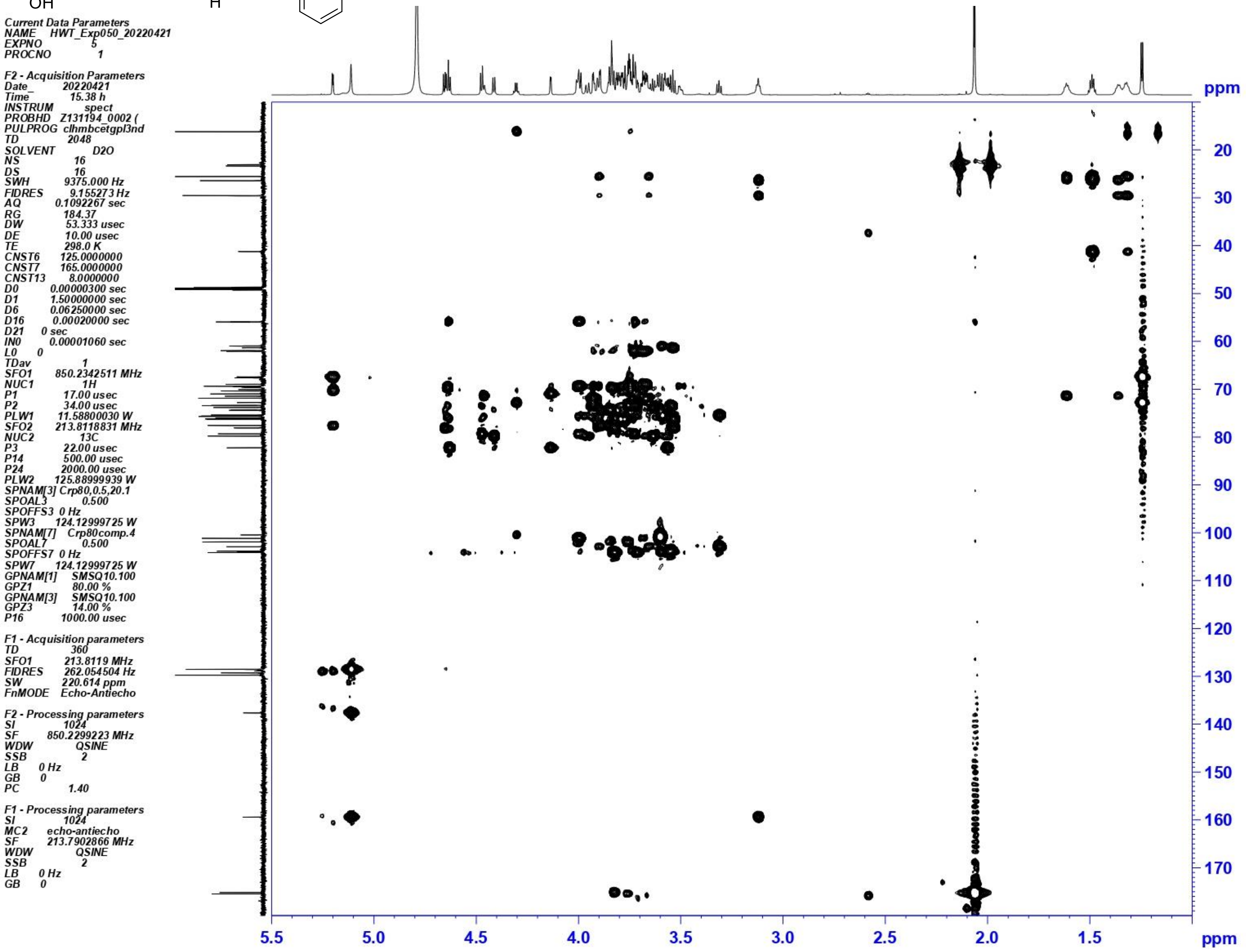

^{1}H-^{13}C HMBC NMR spectrum of **20** (FLNH1) (850 MHz/214 MHz, D_2O)

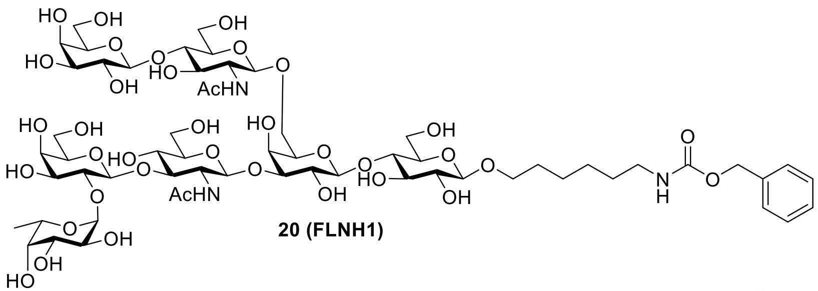

20 (FLNH1)

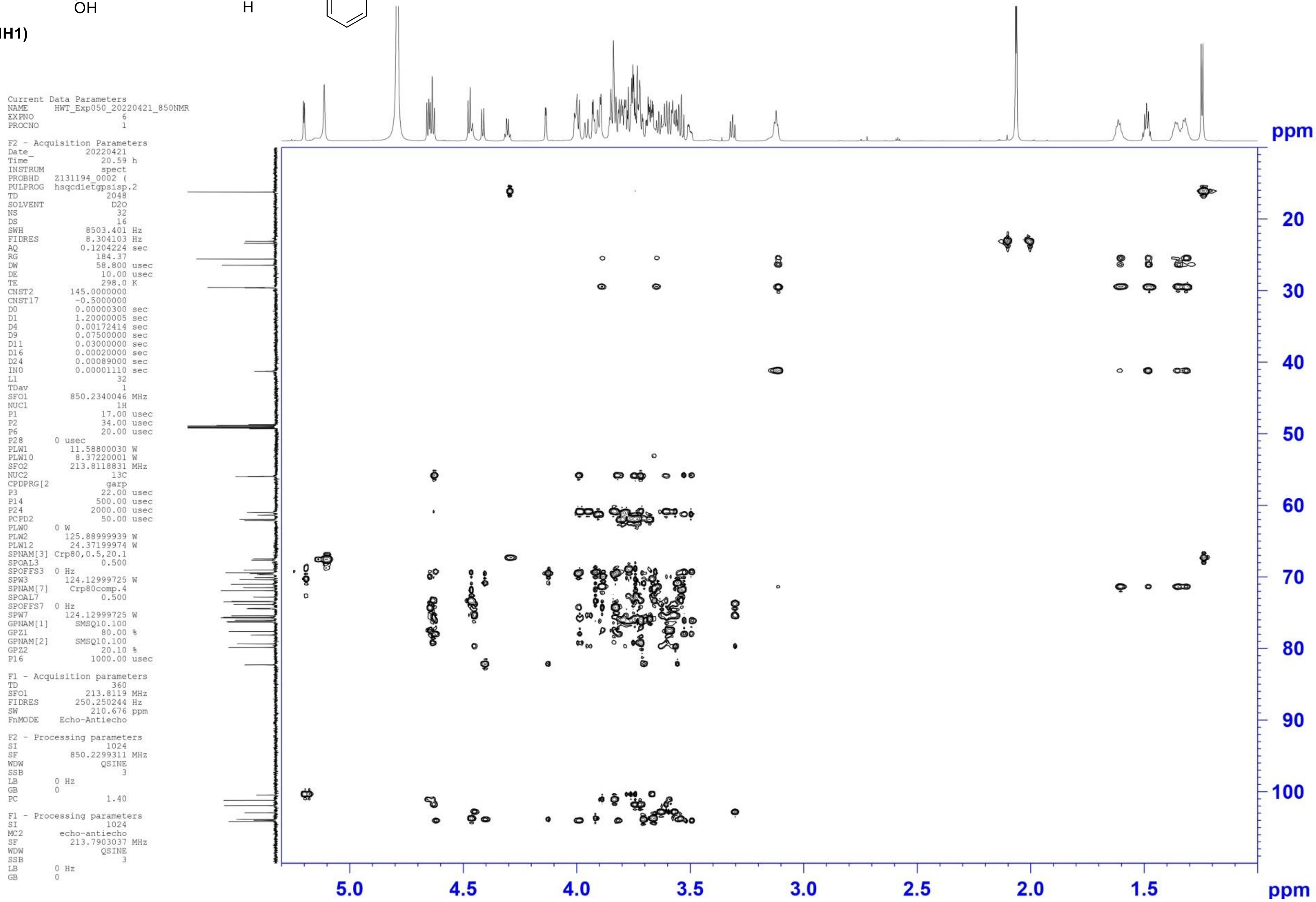

^{1}H-^{13}C HSQC-TOSCY NMR spectrum of **20** (FLNH1) (850 MHz/214 MHz, D_2O)

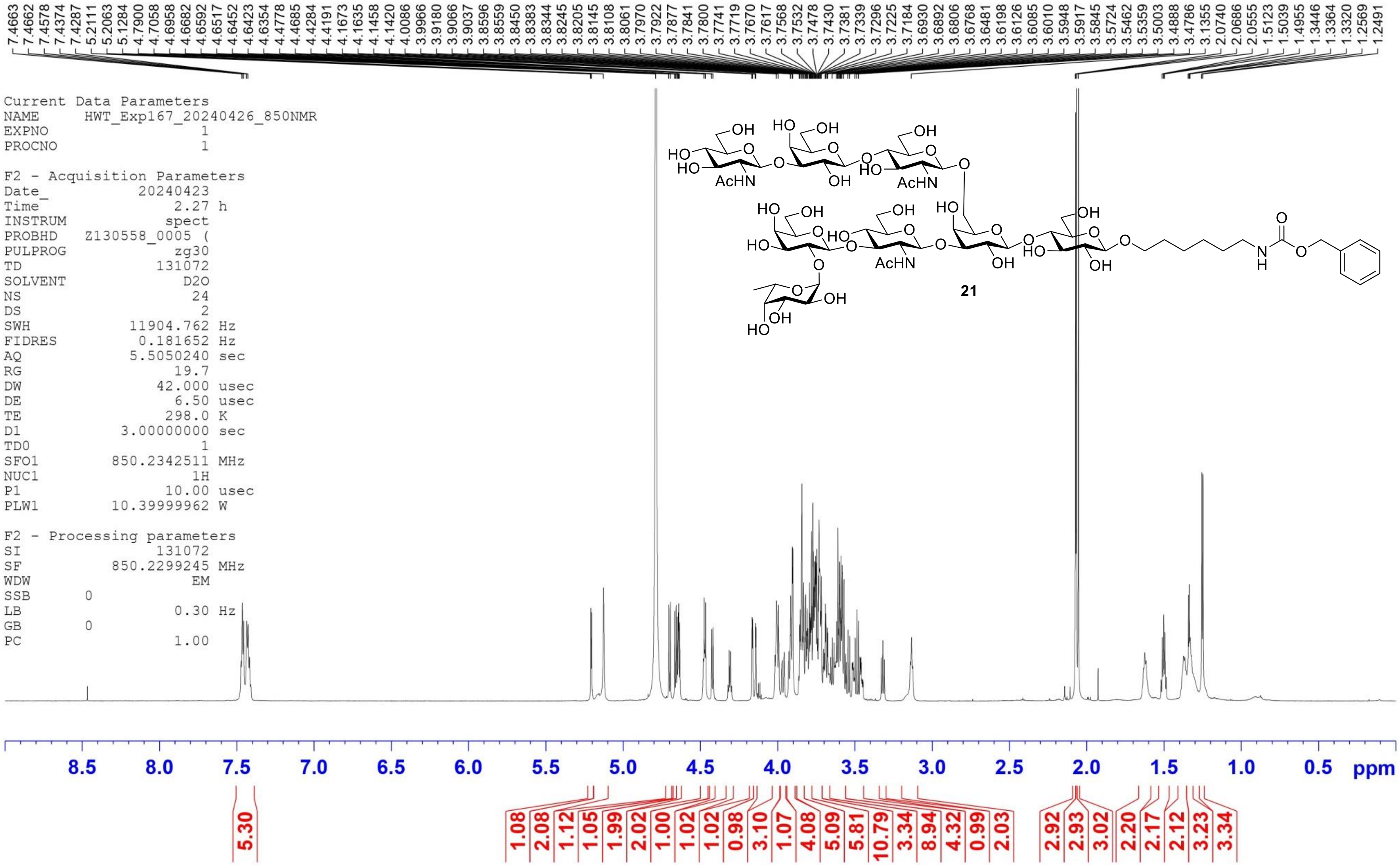

^{1}H NMR spectrum of Compound **21** (850 MHz D_2O)

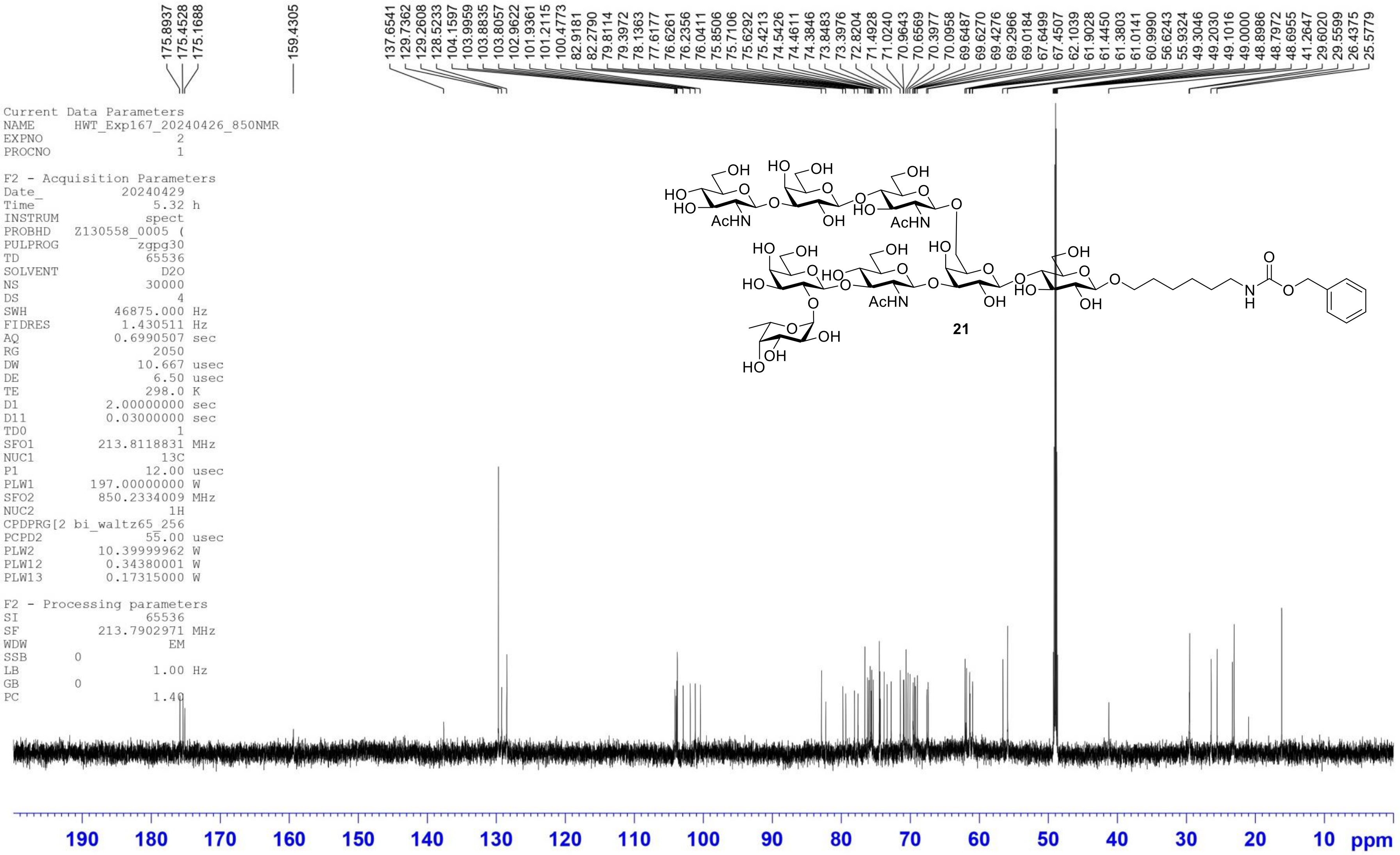

^{13}C NMR spectrum of Compound **21** (214 MHz D_2O)

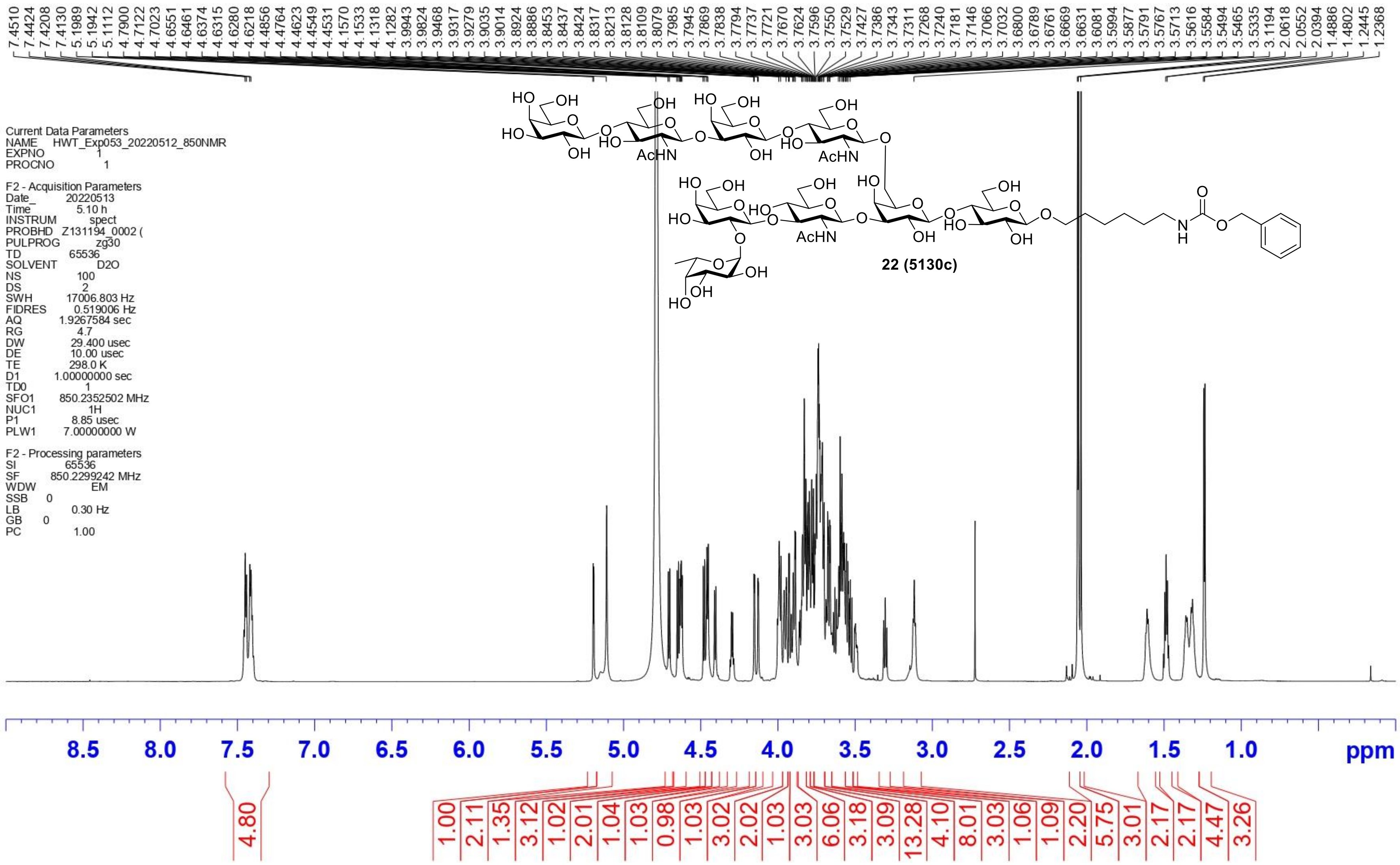

^{1}H NMR spectrum of **22** (5130c) (850 MHz, D_2O)

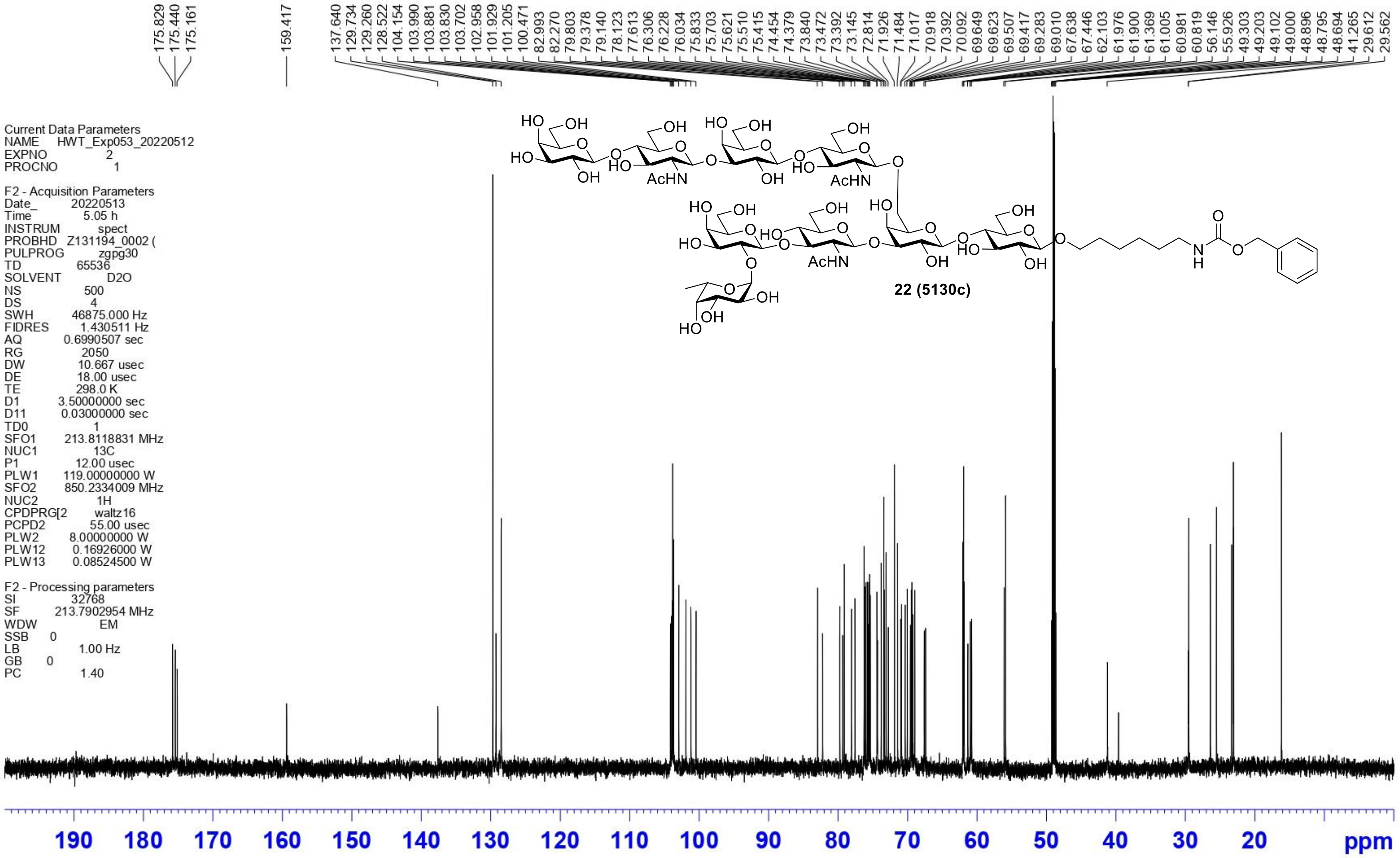

^{13}C NMR spectrum of **22** (5130c) (214 MHz, D_2O)

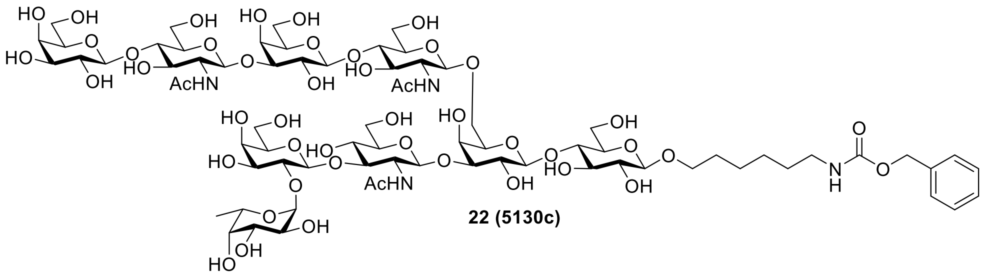

22 (5130c)

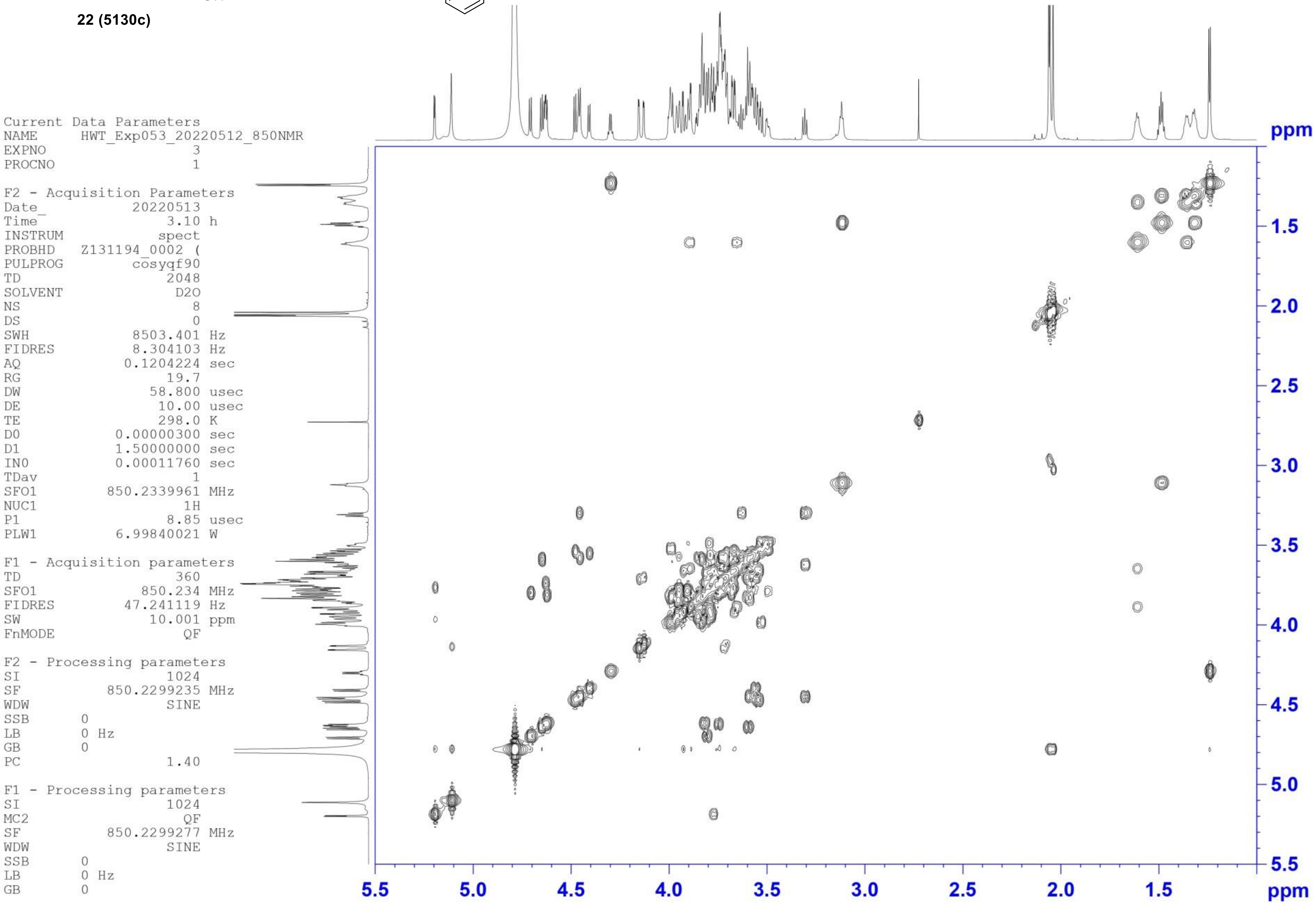

^{1}H-^{1}H COSY NMR spectrum of **22** (5130c) (850 MHz, D_2O)

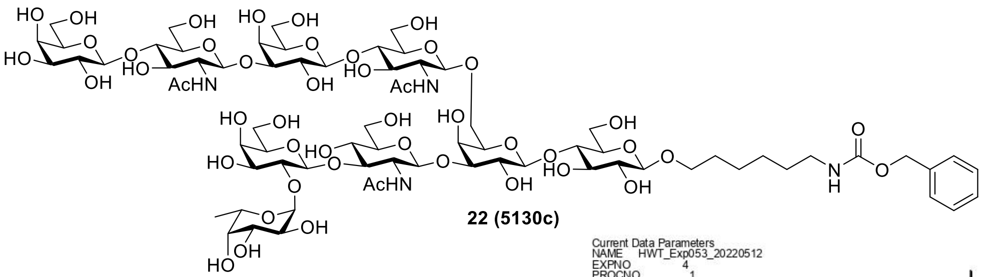

22 (5130c)

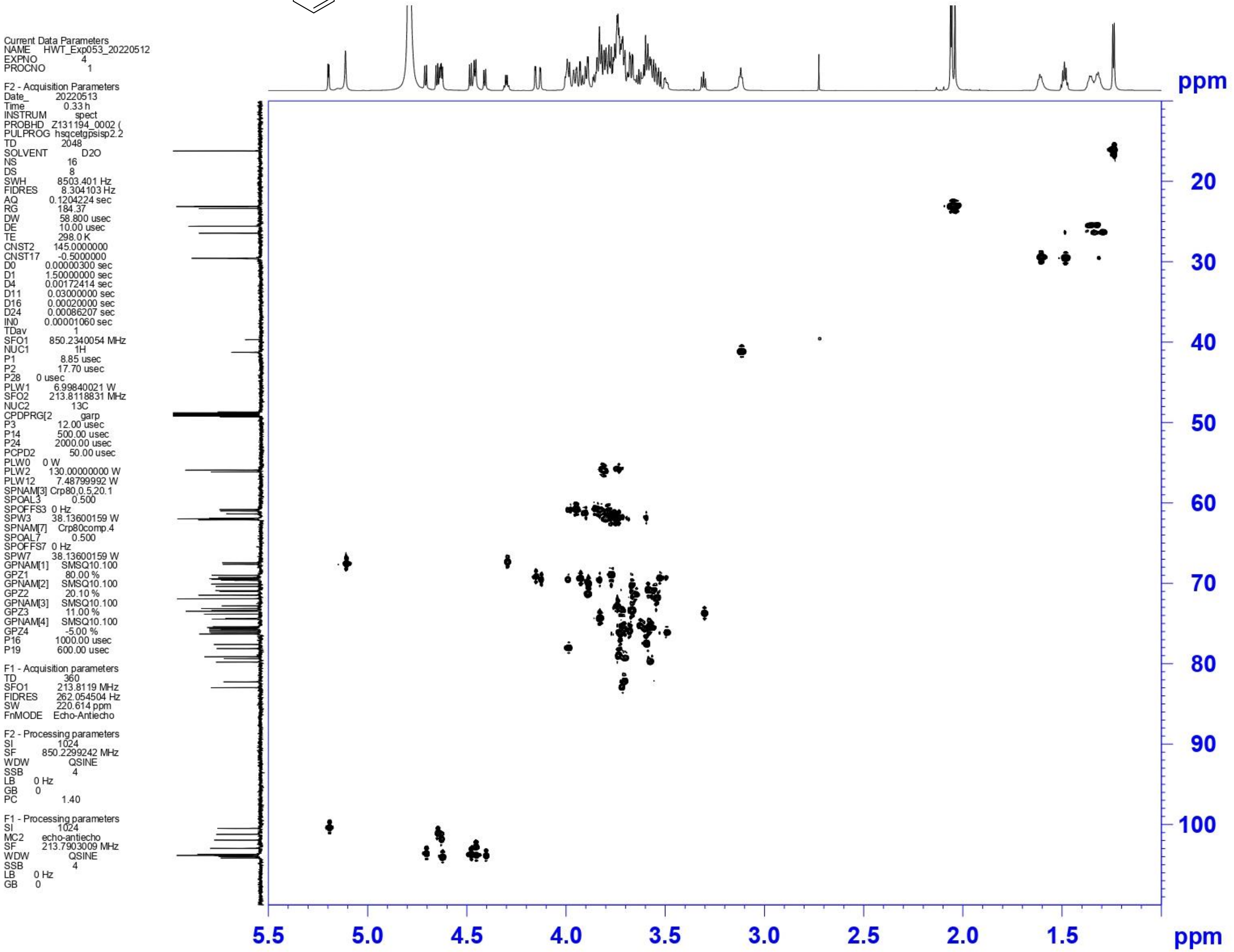

^{1}H-^{13}C HSQC NMR spectrum of **22** (5130c) (850 MHz/214 MHz, D_2O)

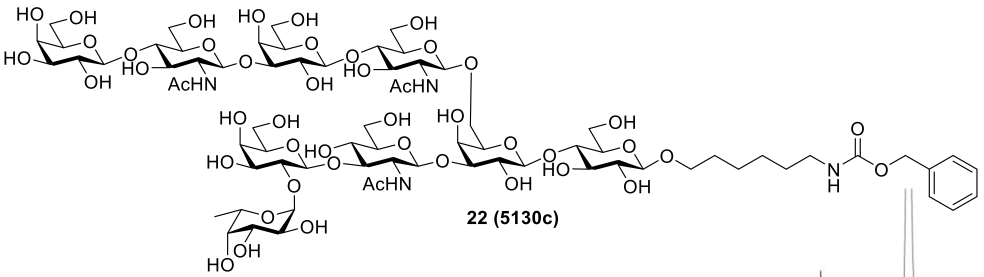

22 (5130c)

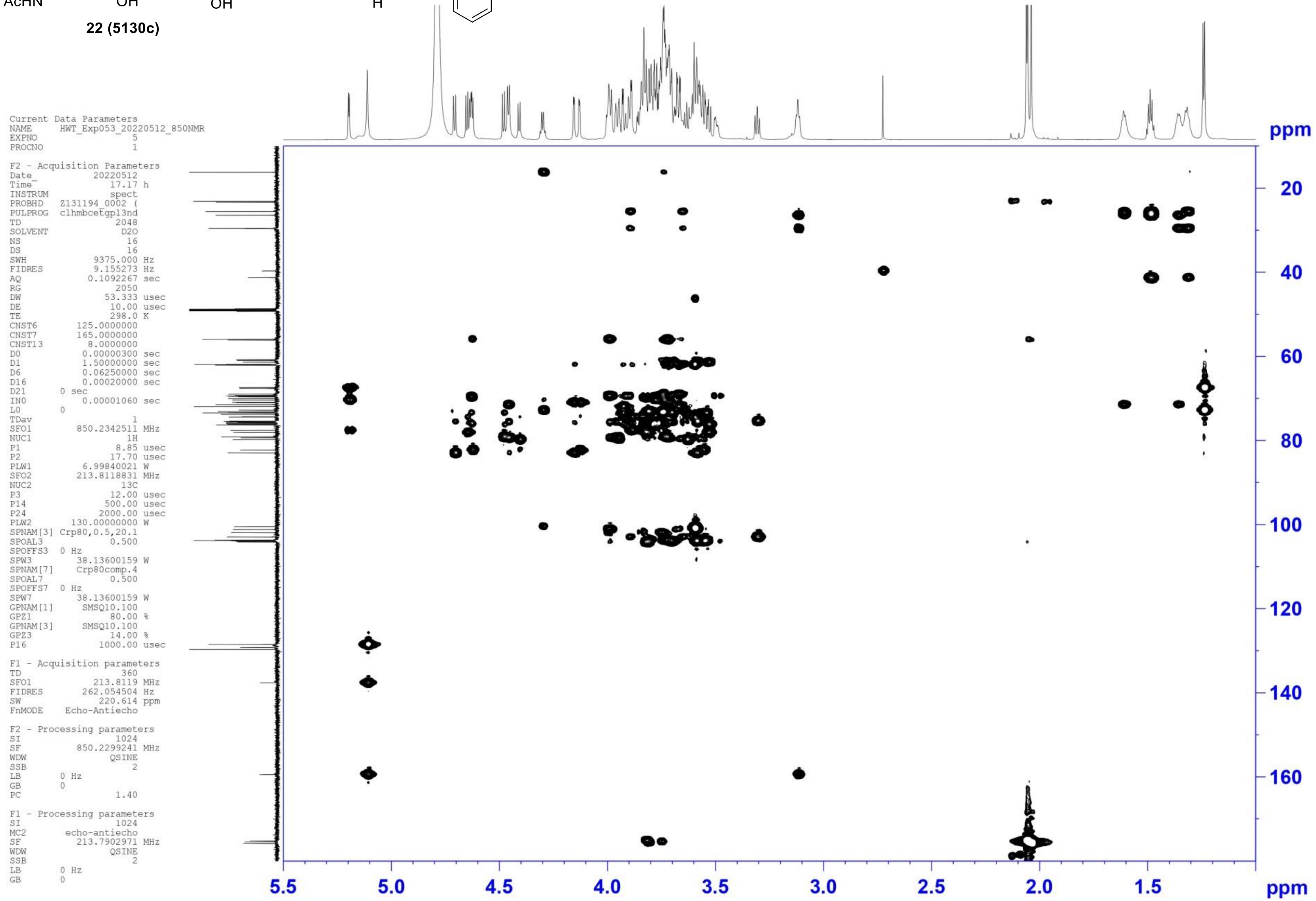

^{1}H-^{13}C HMBC NMR spectrum of **22** (5130c) (850 MHz/214 MHz, D_2O)

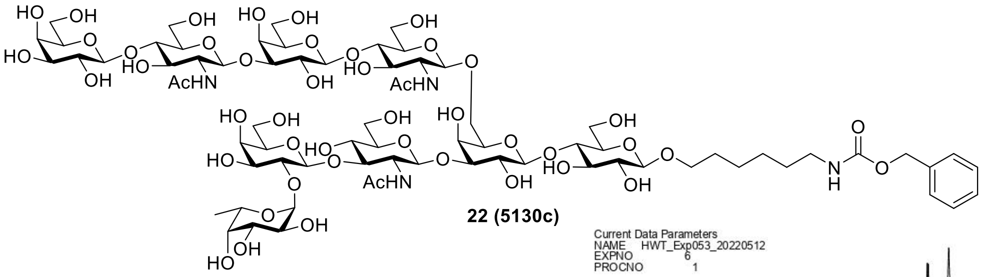

22 (5130c)

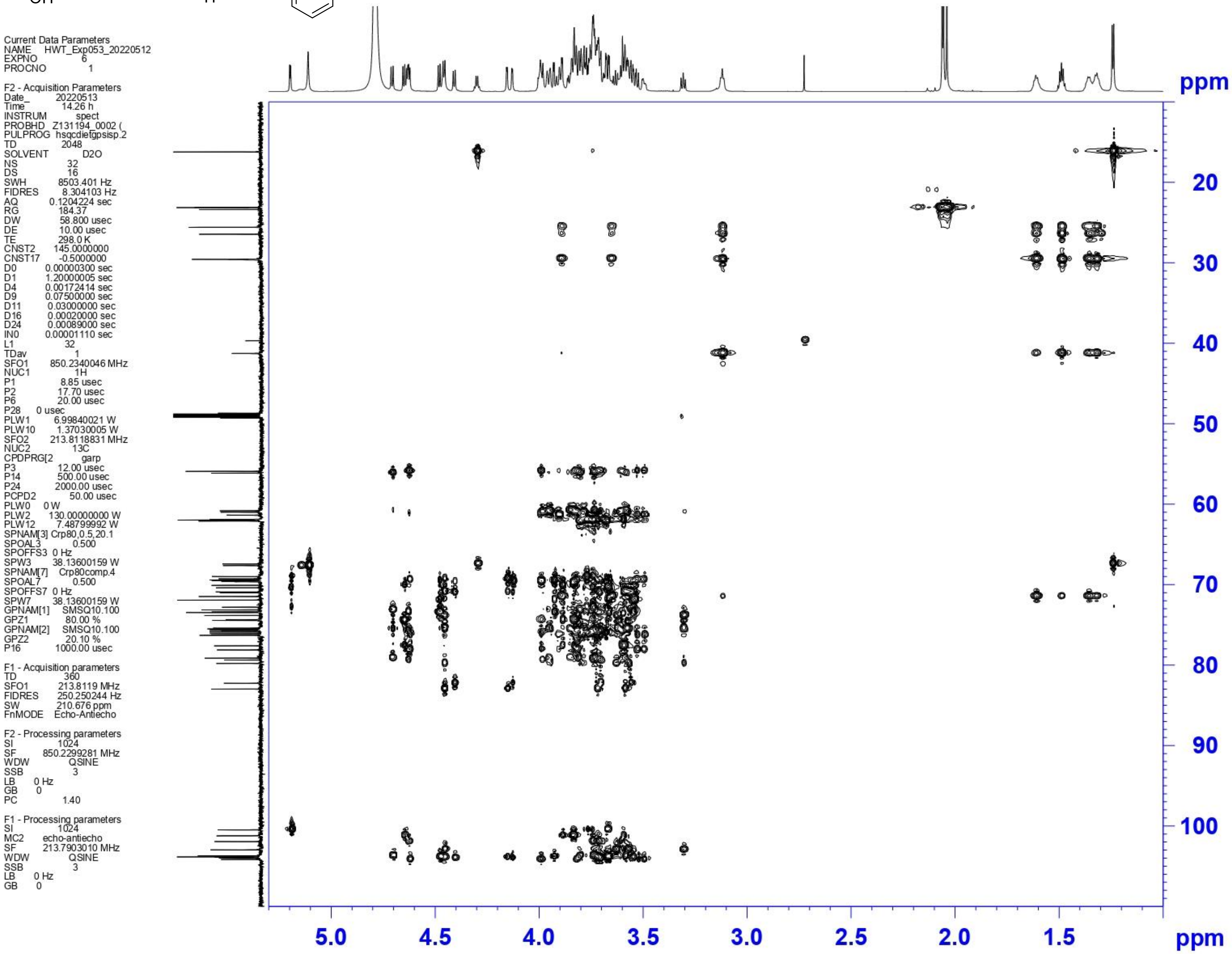

^{1}H-^{13}C HSQC-TOSCY NMR spectrum of **22** (5130c) (850 MHz/214 MHz, D_2O)

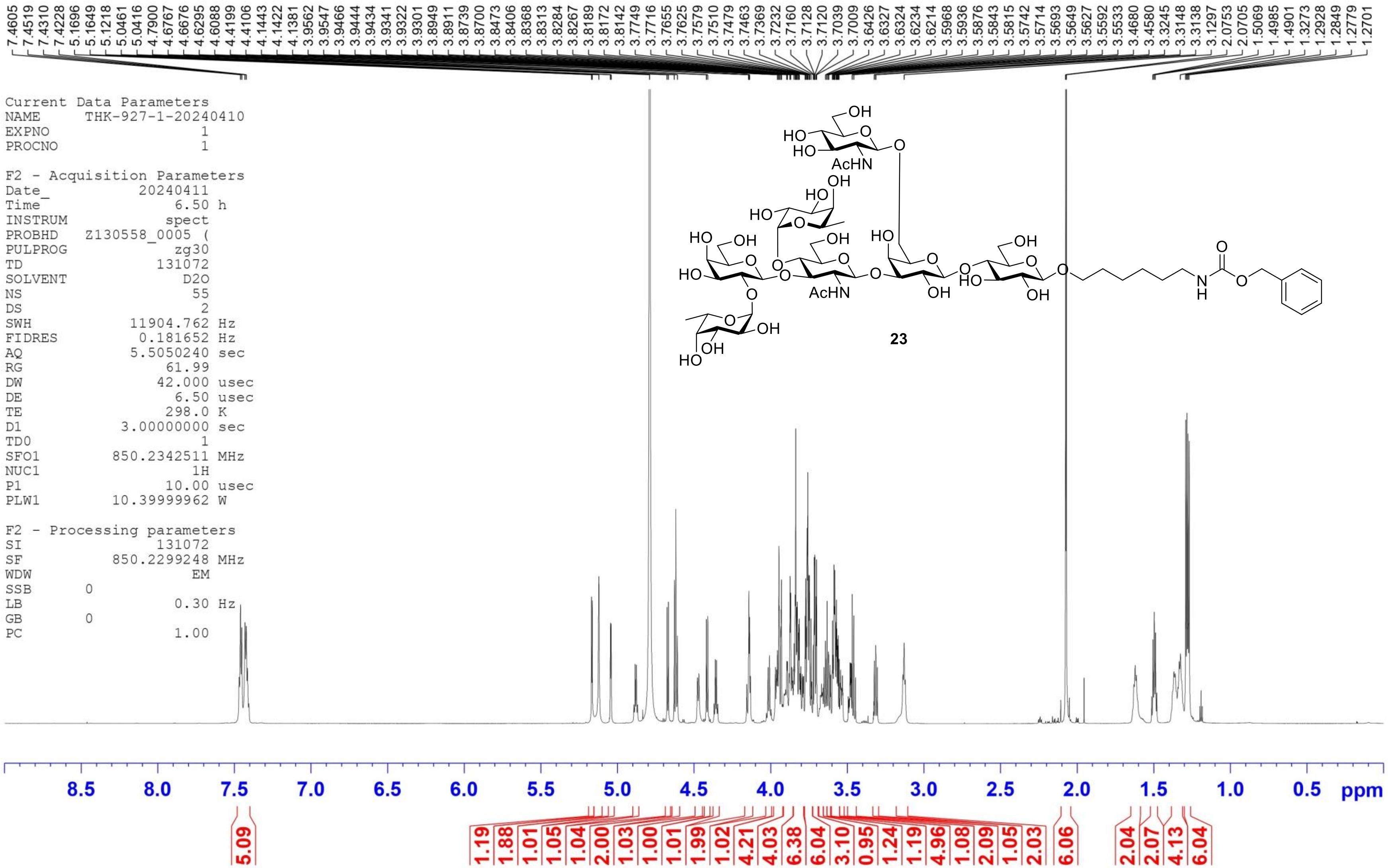

^{1}H NMR spectrum of Compound **23** (850 MHz D_2O)

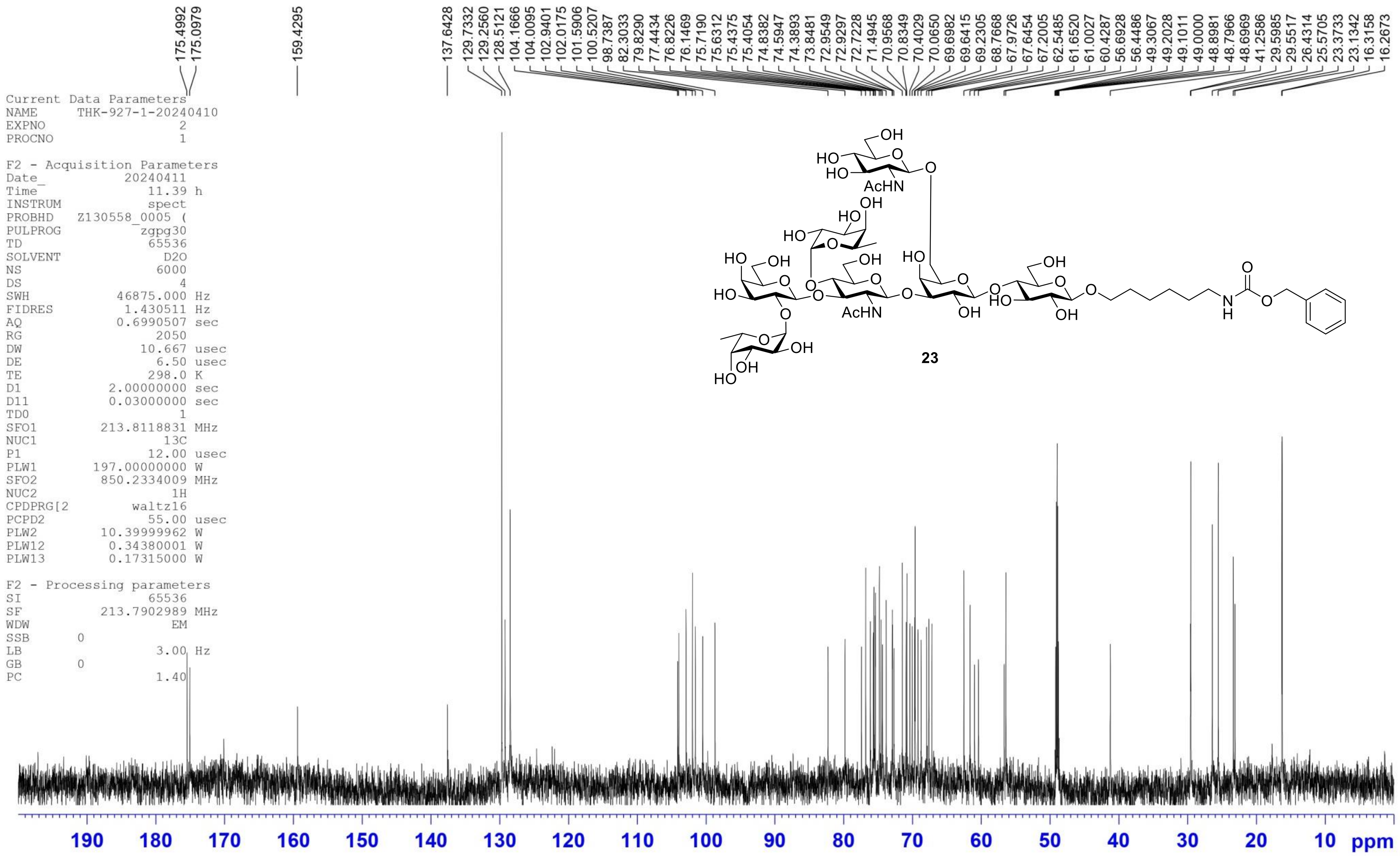

^{13}C NMR spectrum of Compound **23** (214 MHz D_2O)

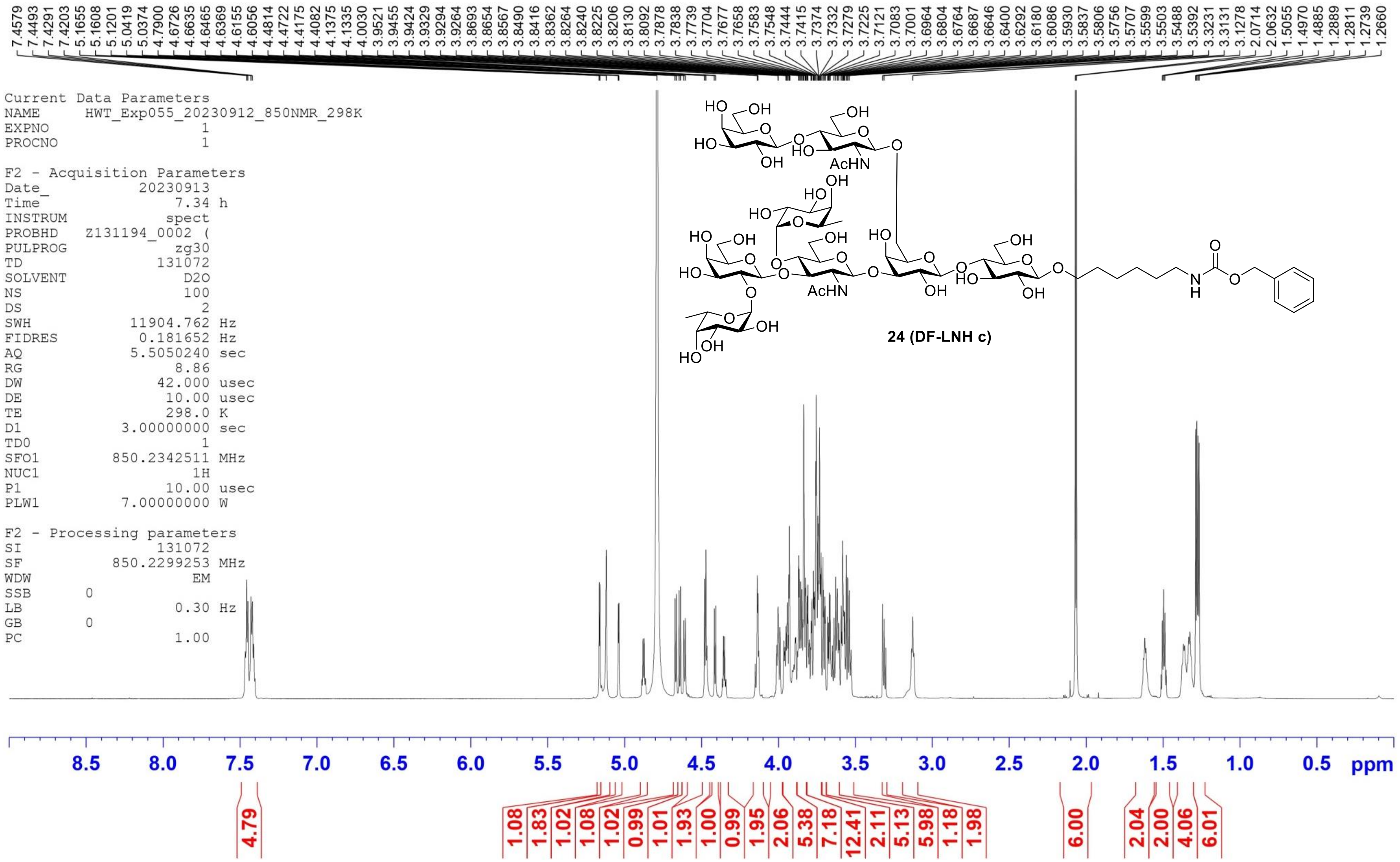

^{1}H NMR spectrum of **24** (DF-LNH c) (850 MHz, D_2O)

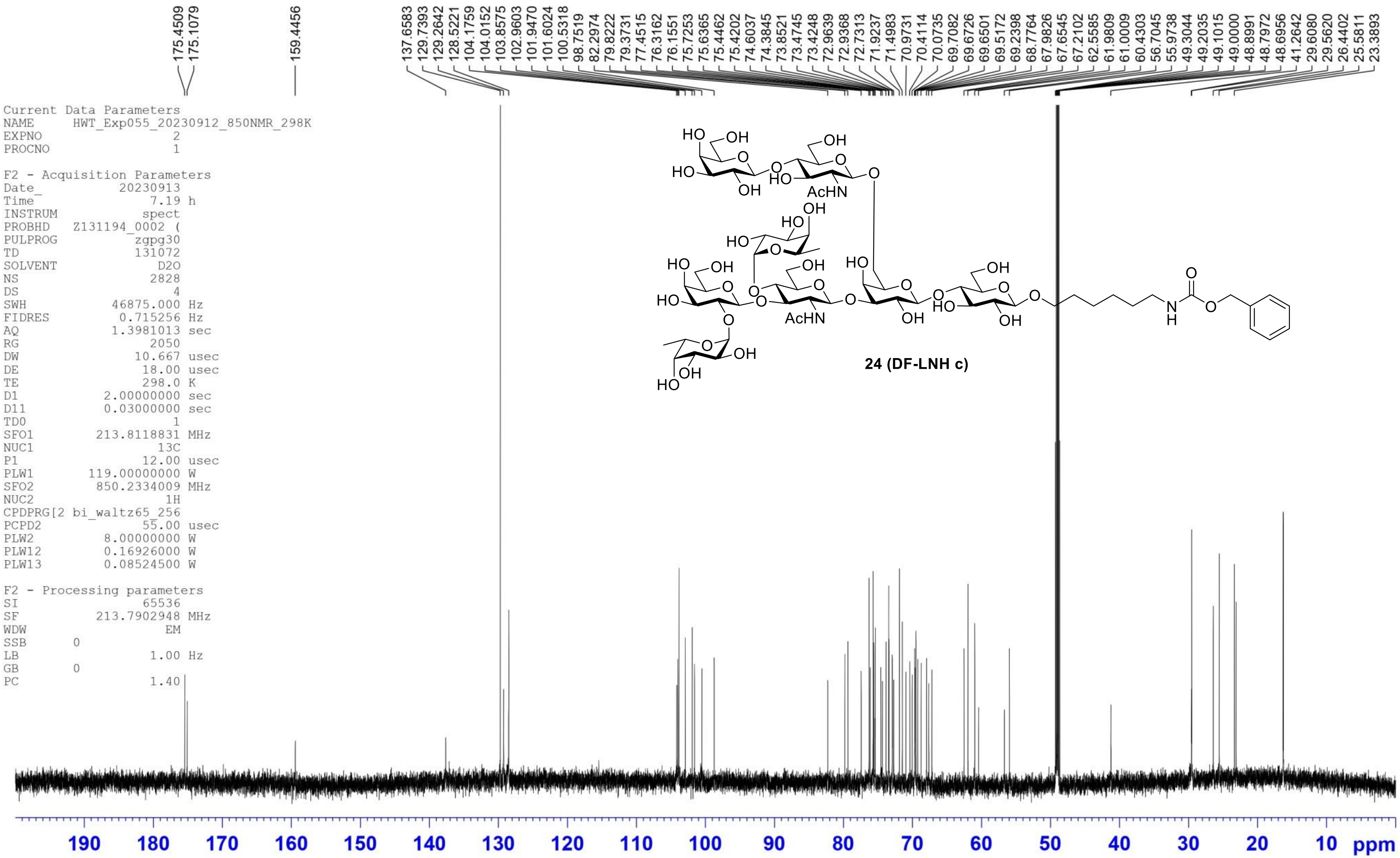

^{13}C NMR spectrum of **24** (DF-LNH c) (214 MHz, D_2O)

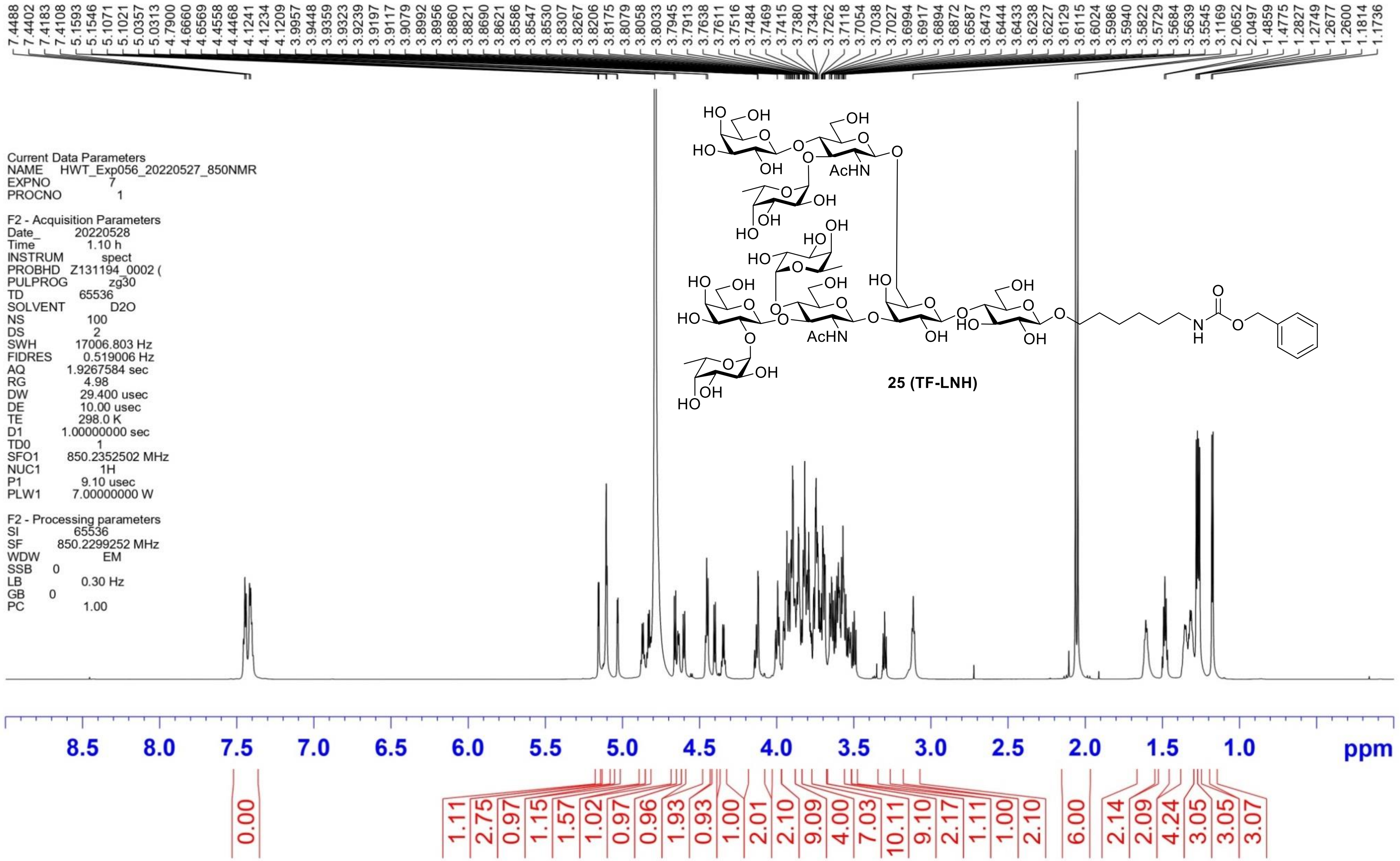

^{1}H NMR spectrum of **25** (TF-LNH) (850 MHz, D_2O)

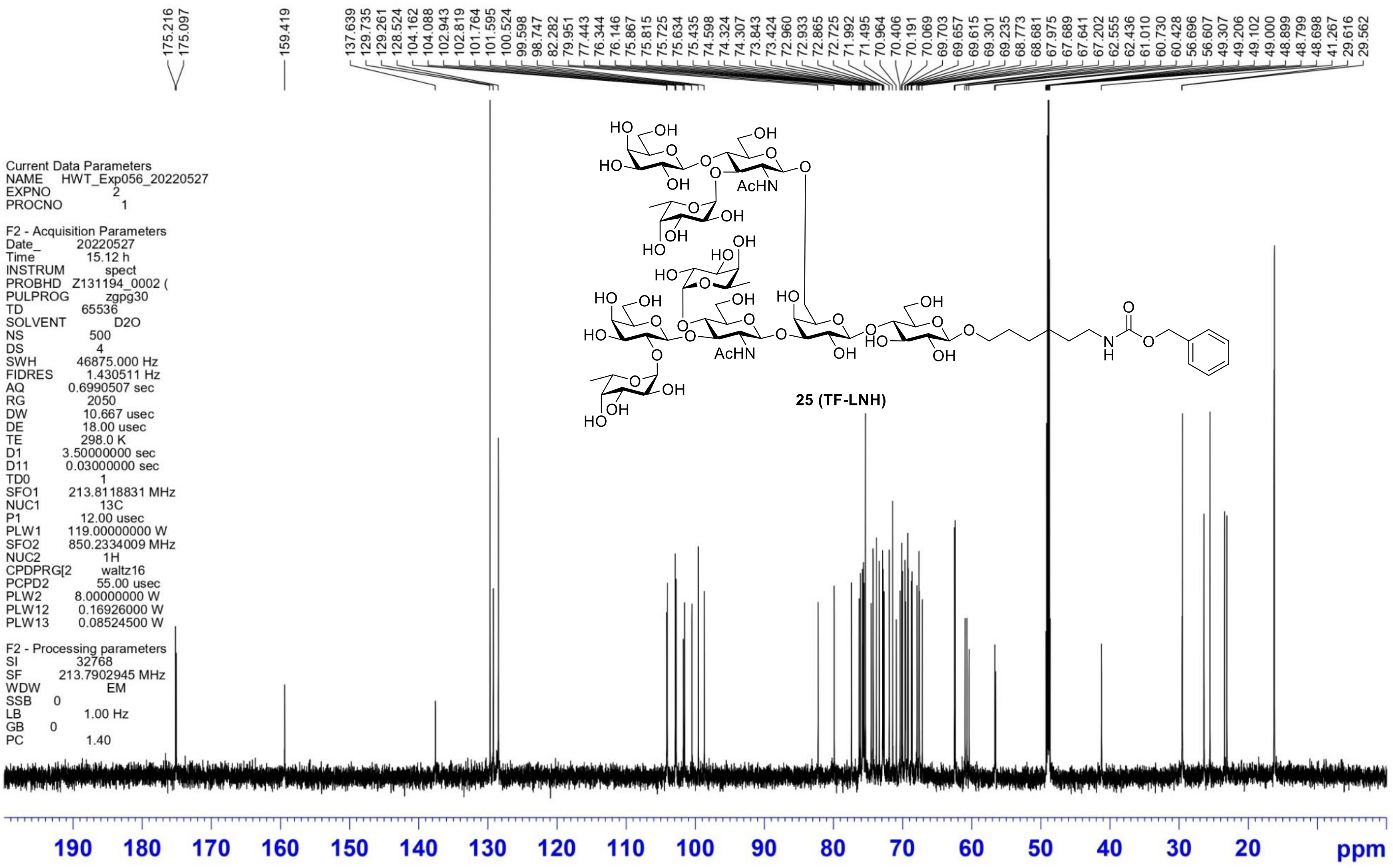

^{13}C NMR spectrum of **25** (TF-LNH) (214 MHz, D_2O)

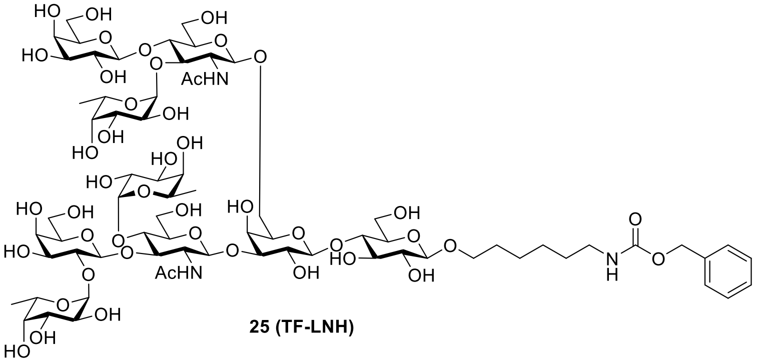

25 (TF-LNH)

```
Current Data Parameters
NAME     HWT_Exp056_20220527_850NMR
EXPNO                   3
PROCNO                  1

F2 - Acquisition Parameters
Date_            20220527
Time                23.46 h
INSTRUM             spect
PROBHD    Z131194_0002 (
PULPROG          cosyqf90
TD                   2048
SOLVENT               D2O
NS                      8
DS                      0
SWH              8503.401 Hz
FIDRES           8.304103 Hz
AQ              0.1204224 sec
RG                  13.07
DW                 58.800 usec
DE                  10.00 usec
TE                  298.0 K
D0             0.00000300 sec
D1             1.50000000 sec
IN0            0.00011760 sec
TDav                    1
SFO1          850.2339961 MHz
NUC1                   1H
P1                   9.10 usec
PLW1           6.99840021 W

F1 - Acquisition parameters
TD                    360
SFO1              850.234 MHz
FIDRES          47.241119 Hz
SW                 10.001 ppm
FnMODE                 QF

F2 - Processing parameters
SI                   1024
SF            850.2299240 MHz
WDW                  SINE
SSB      0
LB       0 Hz
GB       0
PC                   1.40

F1 - Processing parameters
SI                   1024
MC2                    QF
SF            850.2299266 MHz
WDW                  SINE
SSB      0
LB       0 Hz
GB       0
```

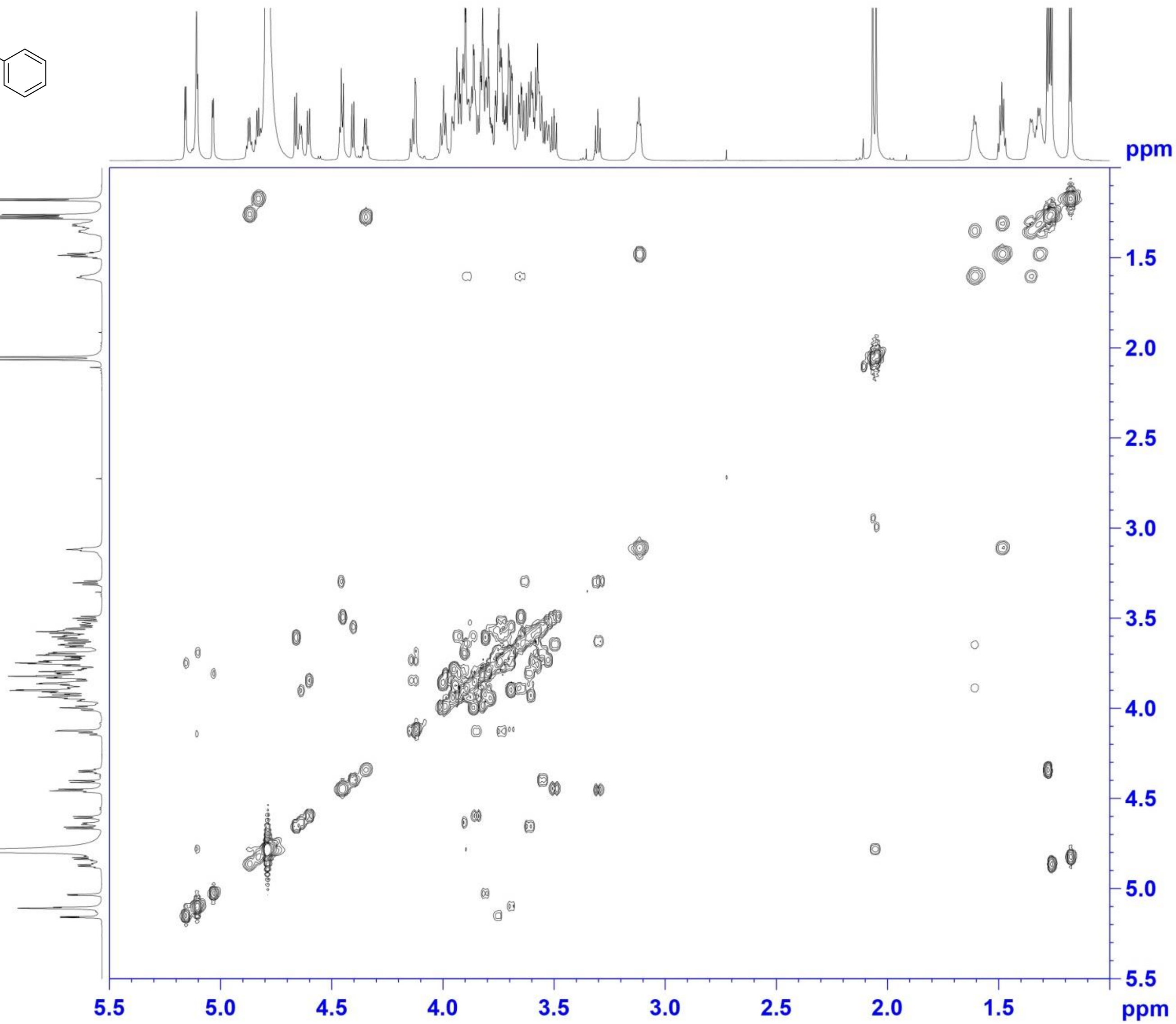

^{1}H-^{1}H COSY NMR spectrum of **25** (TF-LNH) (850 MHz, D_2O)

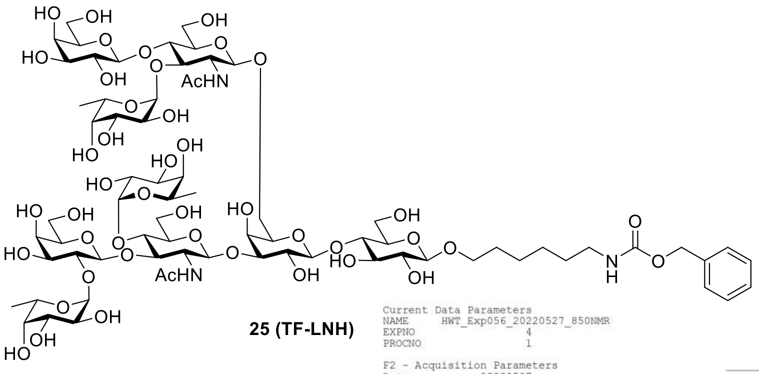

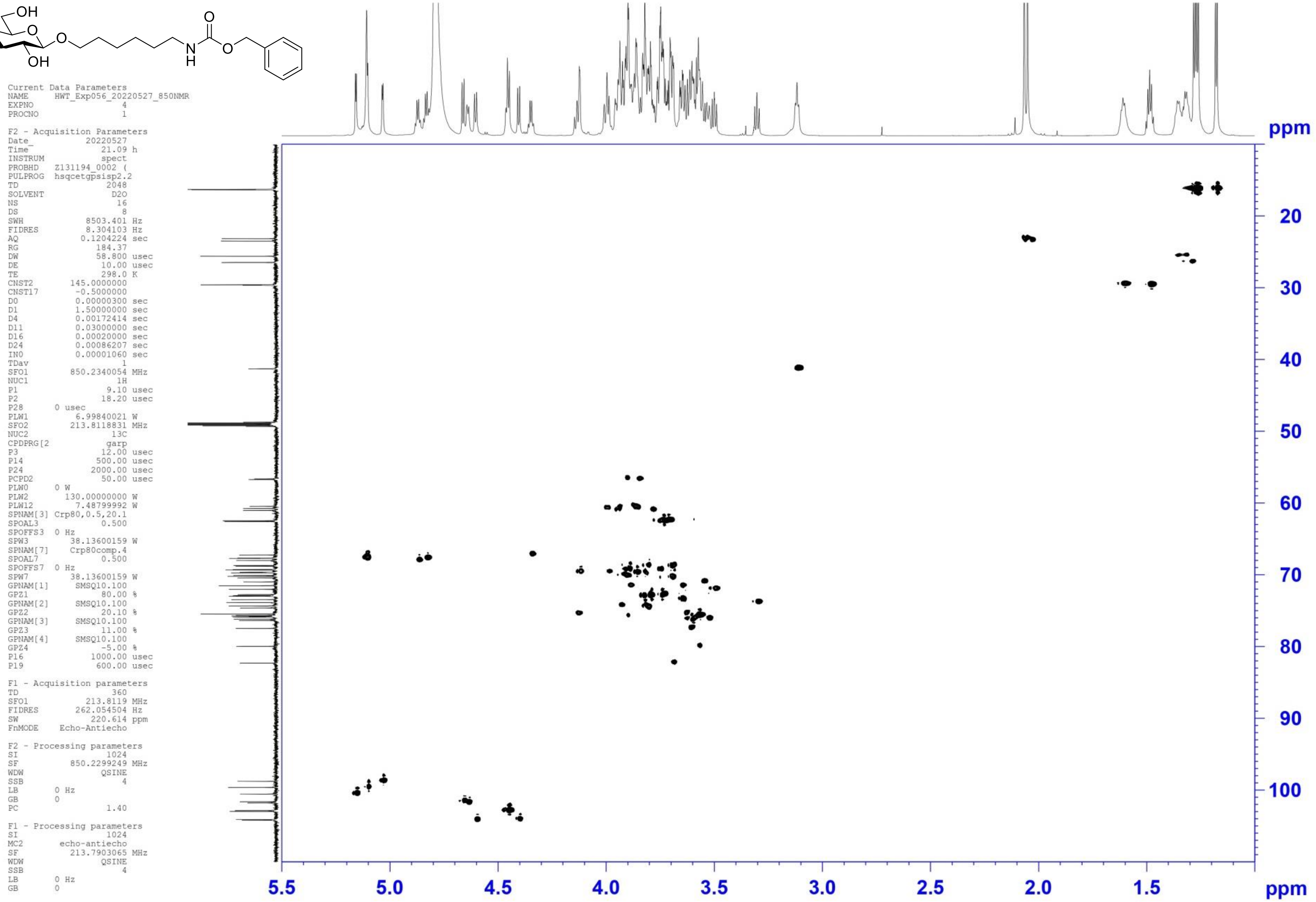

^{1}H-^{13}C HSQC NMR spectrum of **25** (TF-LNH) (850 MHz/214 MHz, D_2O)

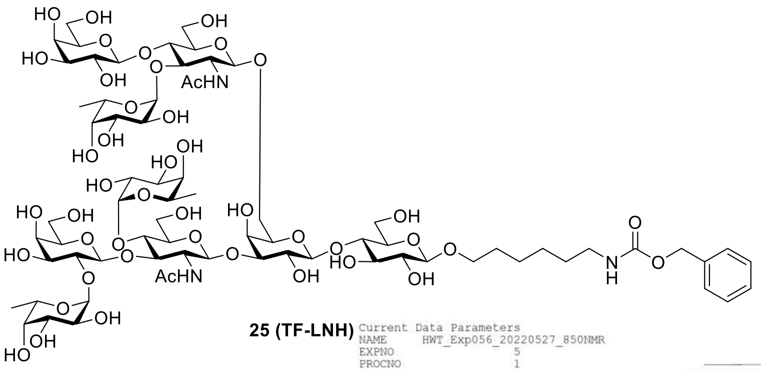

25 (TF-LNH)

```
Current Data Parameters
NAME      HWT_Exp056_20220527_850NMR
EXPNO                     5
PROCNO                    1

F2 - Acquisition Parameters
Date_              20220527
Time                  18.26 h
INSTRUM               spect
PROBHD    Z131194_0002 (
PULPROG   clhmbcetgpl3nd
TD                     2048
SOLVENT                 D2O
NS                       16
DS                       16
SWH                9375.000 Hz
FIDRES             9.155273 Hz
AQ                0.1092267 sec
RG                     2050
DW                   53.333 usec
DE                    10.00 usec
TE                    298.0 K
CNST6           125.0000000
CNST7           165.0000000
CNST13            8.0000000
D0               0.00000300 sec
D1               1.50000000 sec
D6               0.06250000 sec
D16              0.00020000 sec
D21       0 sec
IN0              0.00001060 sec
L0        0
TDav                      1
SFO1            850.2342511 MHz
NUC1                     1H
P1                     9.10 usec
P2                    18.20 usec
PLW1             6.99840021 W
SFO2            213.8118831 MHz
NUC2                    13C
P3                    12.00 usec
P14                  500.00 usec
P24                 2000.00 usec
PLW2           130.00000000 W
SPNAM[3]  Crp80,0.5,20.1
SPOAL3                0.500
SPOFFS3   0 Hz
SPW3            38.13600159 W
SPNAM[7]      Crp80comp.4
SPOAL7                0.500
SPOFFS7   0 Hz
SPW7            38.13600159 W
GPNAM[1]        SMSQ10.100
GPZ1                  80.00 %
GPNAM[3]        SMSQ10.100
GPZ3                  14.00 %
P16                 1000.00 usec

F1 - Acquisition parameters
TD                      360
SFO1               213.8119 MHz
FIDRES           262.054504 Hz
SW                  220.614 ppm
FnMODE      Echo-Antiecho

F2 - Processing parameters
SI                     1024
SF              850.2299275 MHz
WDW                   QSINE
SSB                       2
LB        0 Hz
GB        0
PC                     1.40

F1 - Processing parameters
SI                     1024
MC2          echo-antiecho
SF              213.7903037 MHz
WDW                   QSINE
SSB                       2
LB        0 Hz
GB        0
```

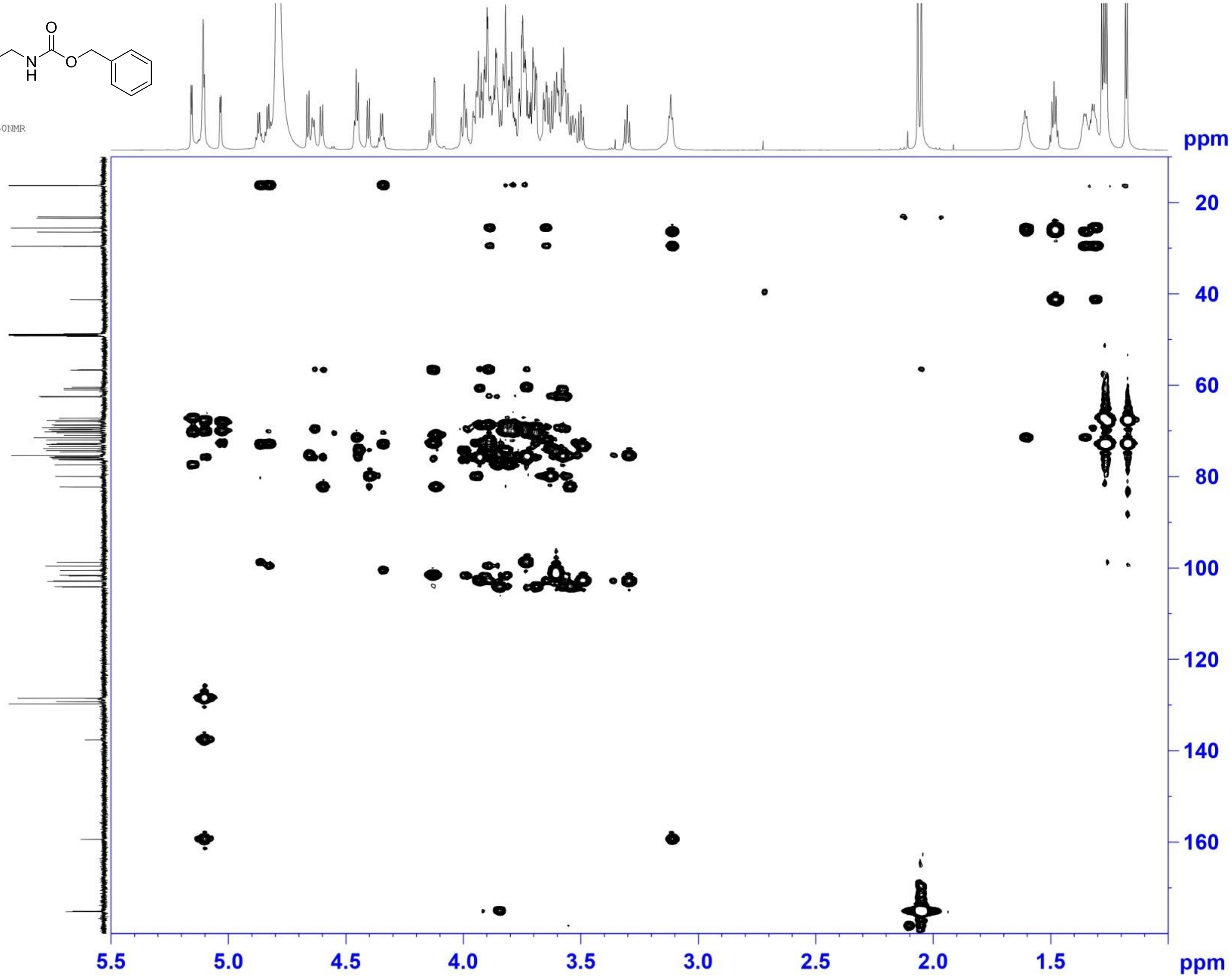

^{1}H-^{13}C HMBC NMR spectrum of **25** (TF-LNH) (850 MHz/214 MHz, D_2O)

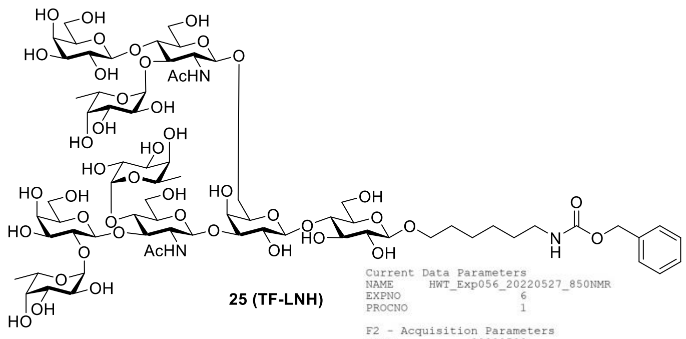

25 (TF-LNH)

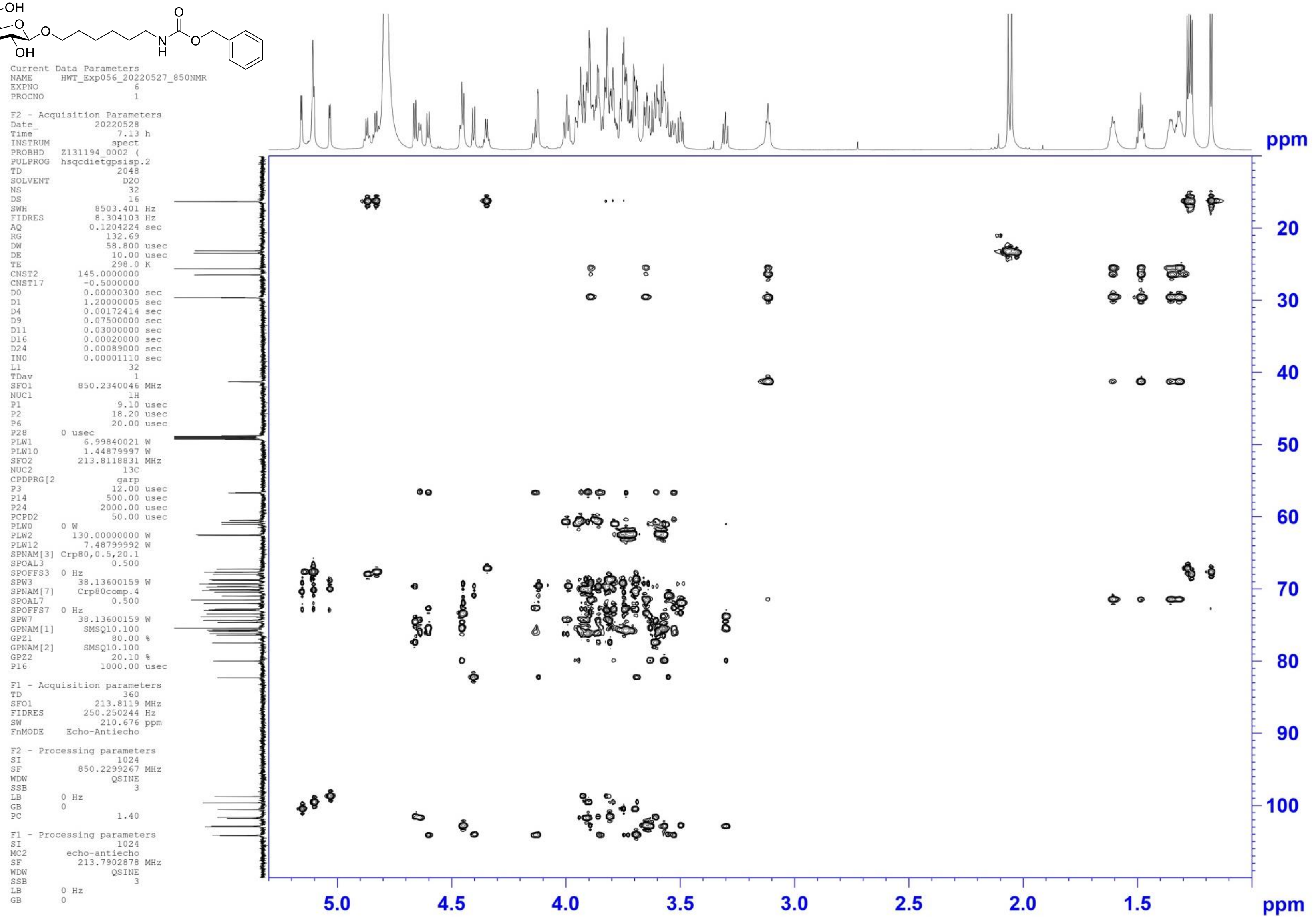

^{1}H-^{13}C HSQC-TOSCY NMR spectrum of **25** (TF-LNH) (850 MHz/214 MHz, D_2O)

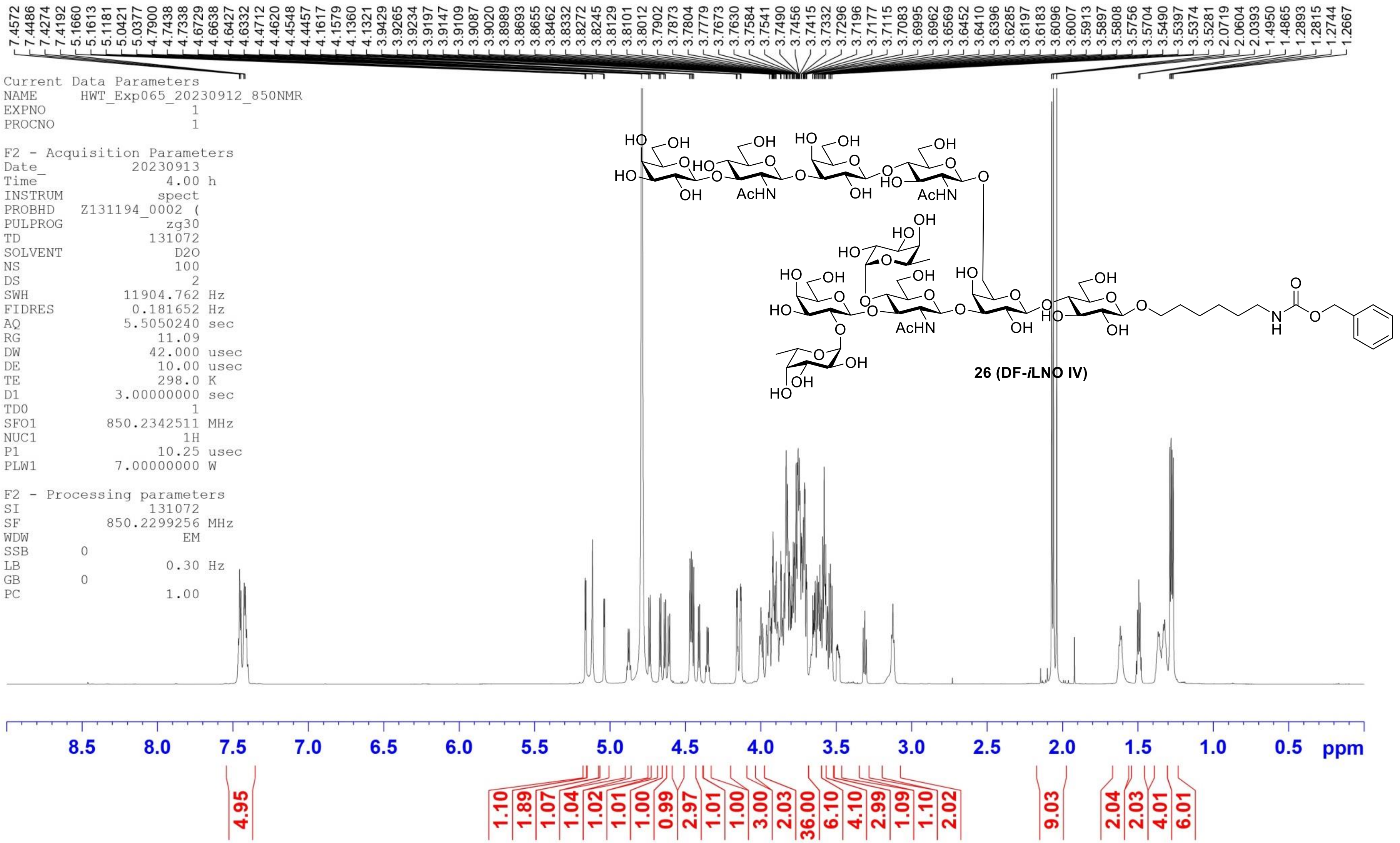

^{1}H NMR spectrum of **26** (DF-*i*LNO IV) (850 MHz, D_2O)

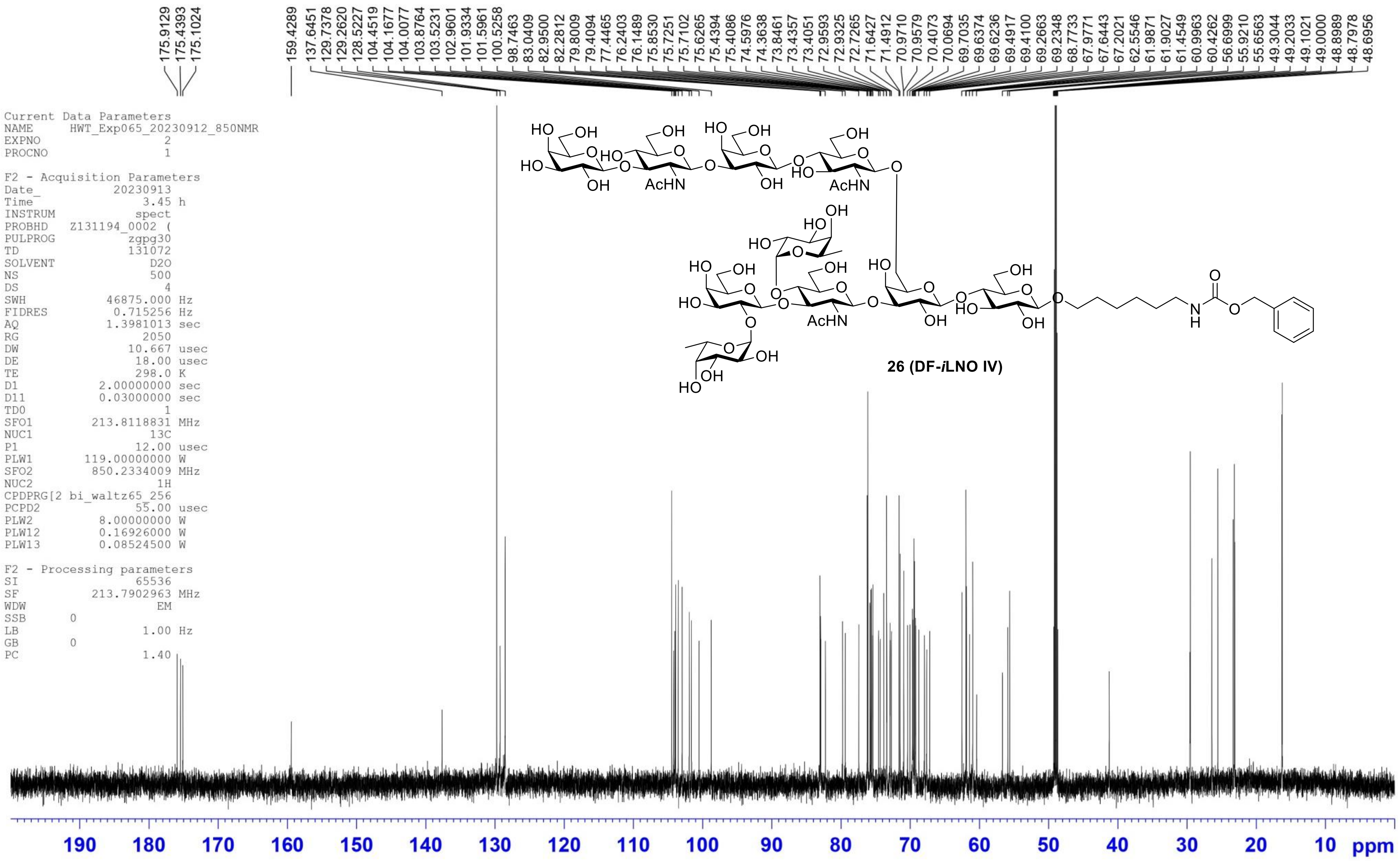

^{13}C NMR spectrum of **26** (DF-*i*LNO IV) (214 MHz, D_2O)

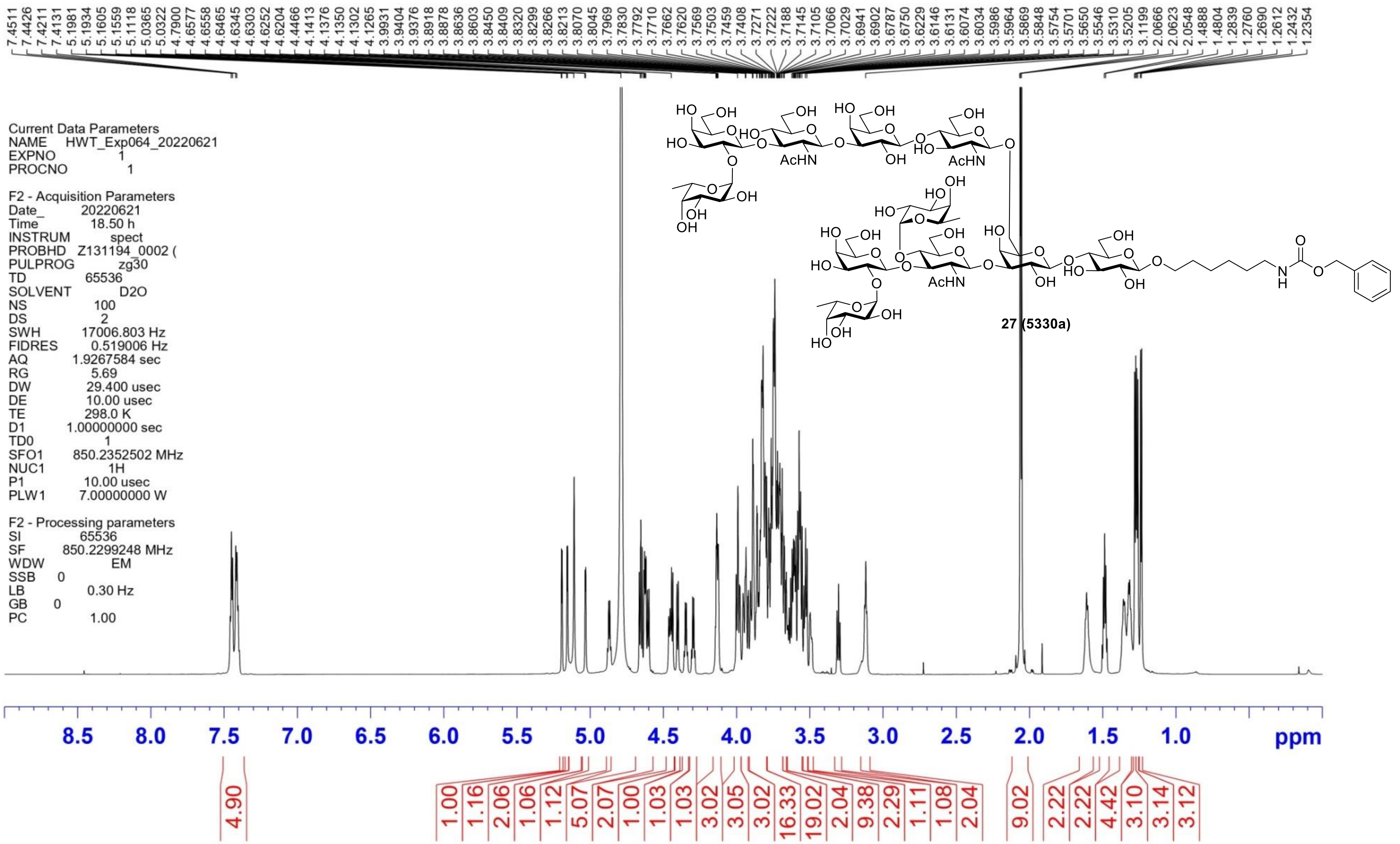

^{1}H NMR spectrum of **27** (5330a) (850 MHz, D_2O)

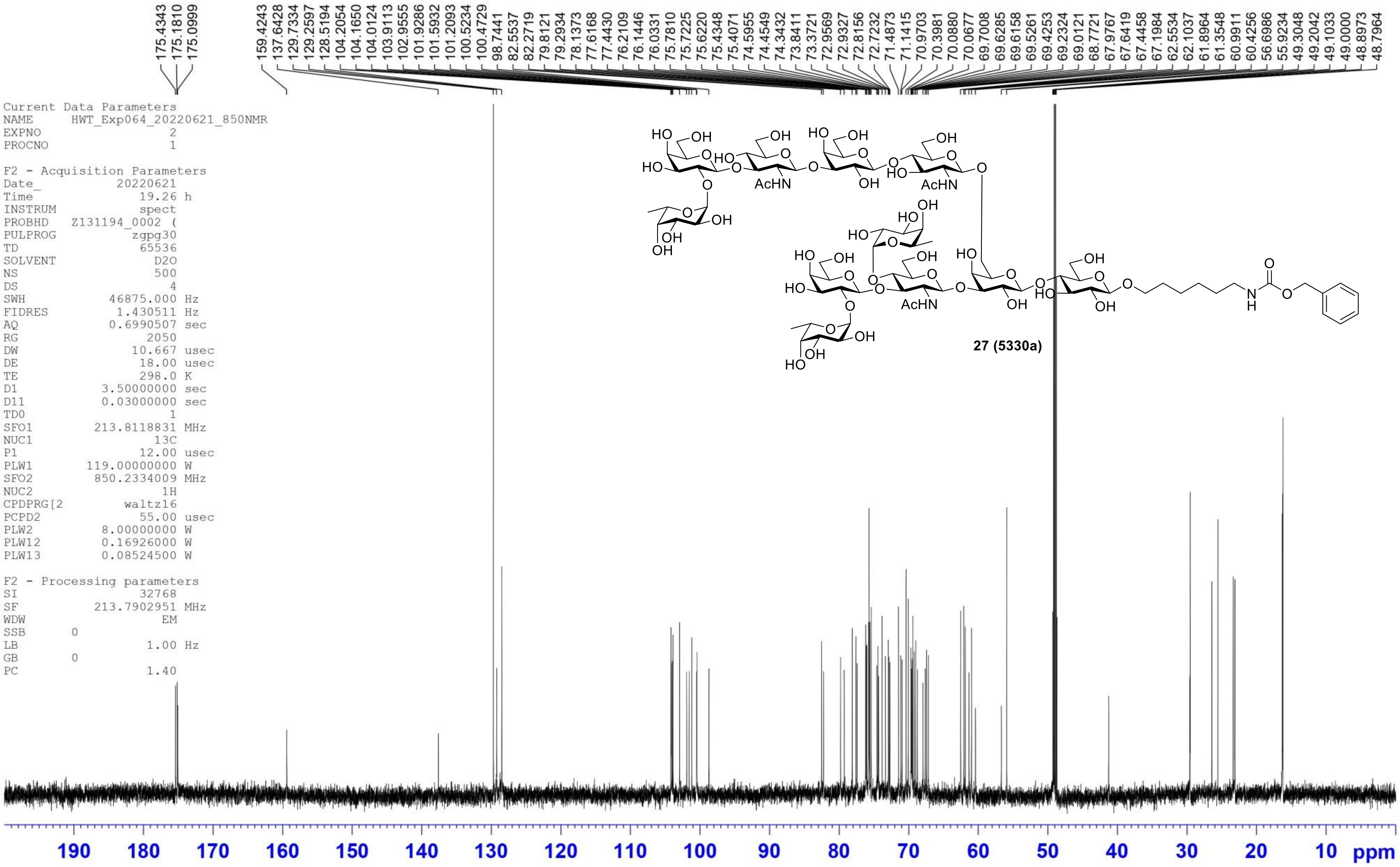

^{13}C NMR spectrum of **27** (5330a) (214 MHz, D_2O)

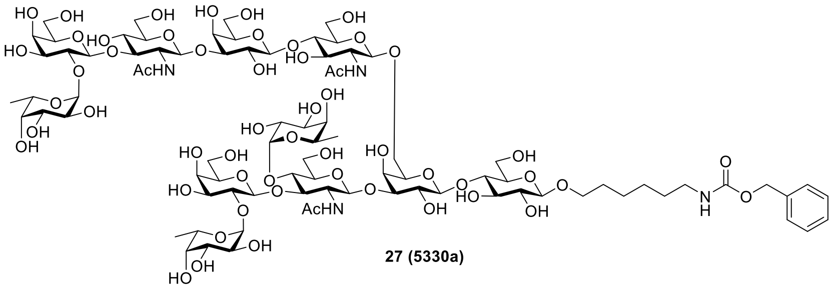

27 (5330a)

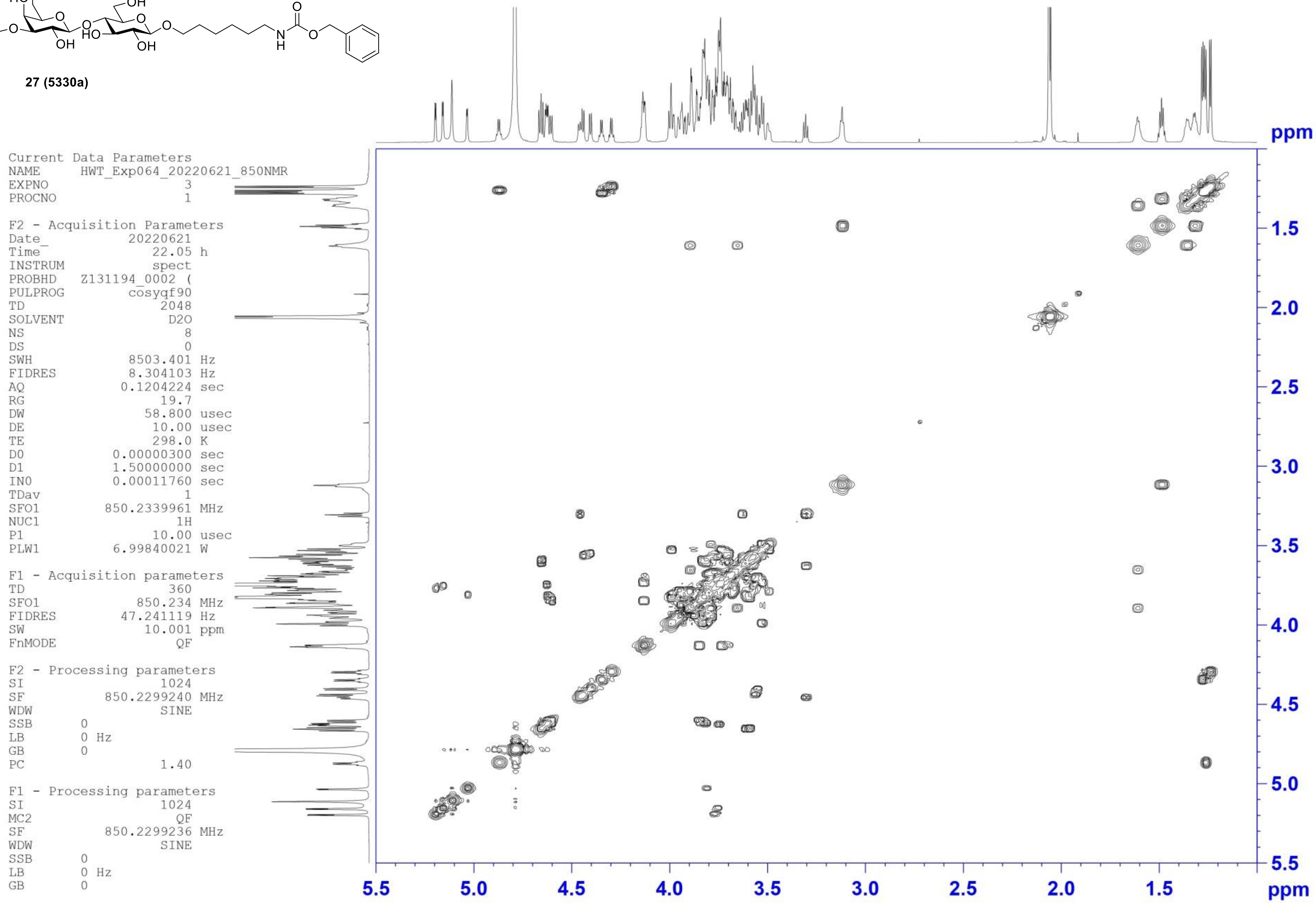

^{1}H-^{1}H COSY NMR spectrum of **27** (5330a) (850 MHz, D_2O)

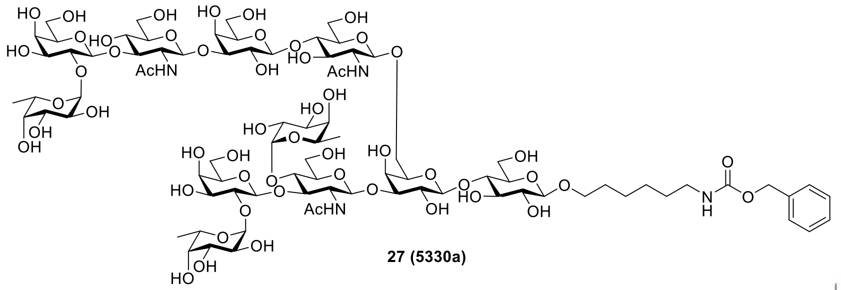

27 (5330a)

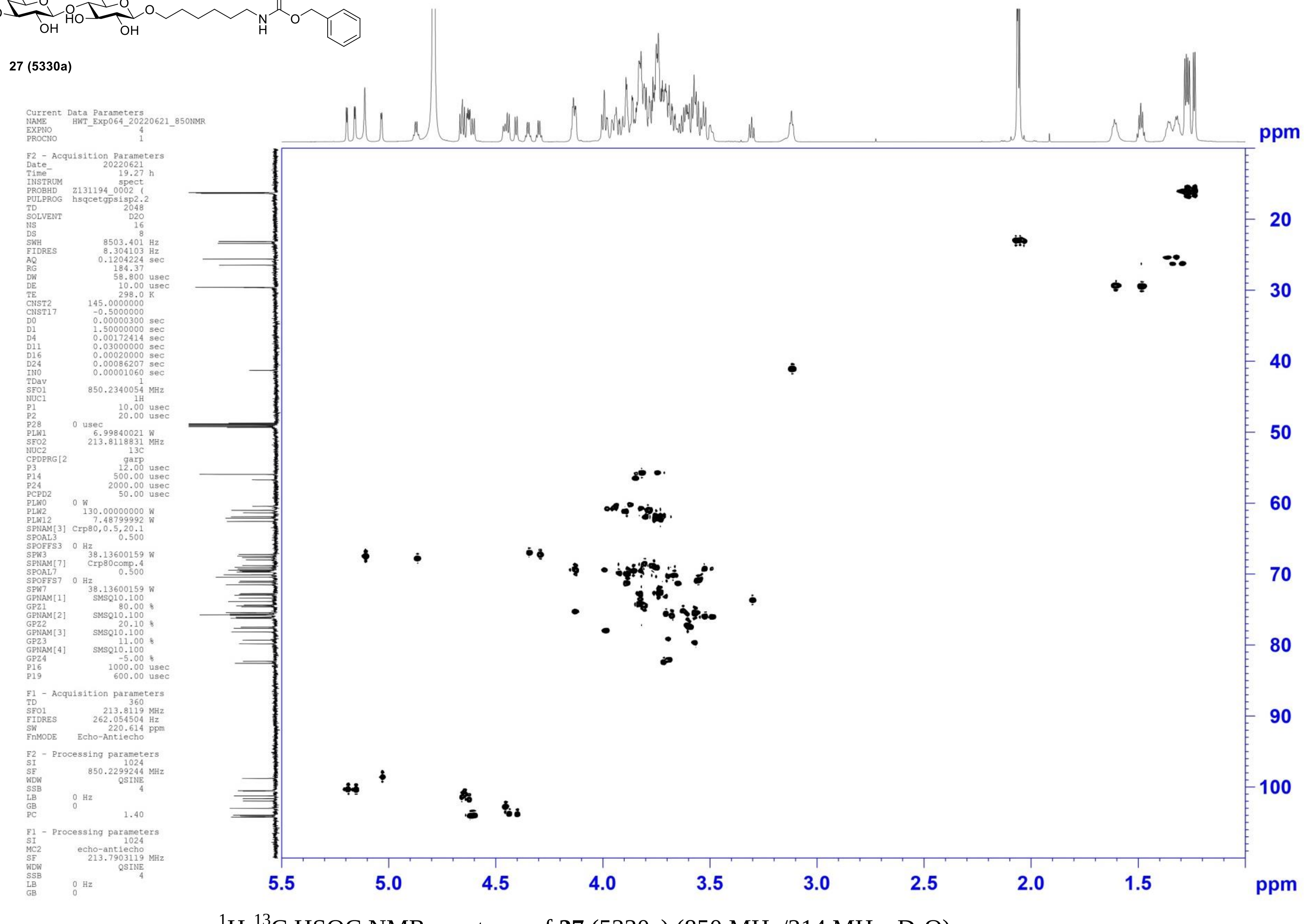

^{1}H-^{13}C HSQC NMR spectrum of **27** (5330a) (850 MHz/214 MHz, D_2O)

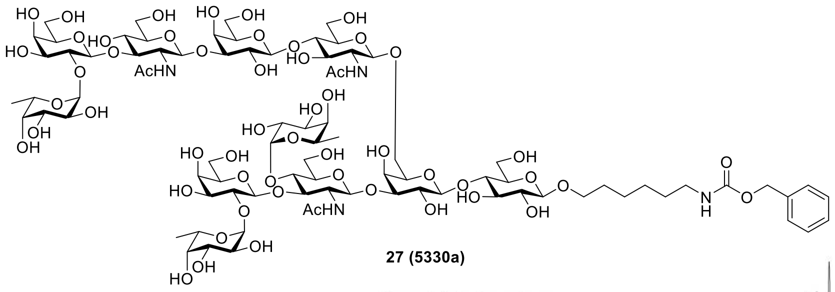

27 (5330a)

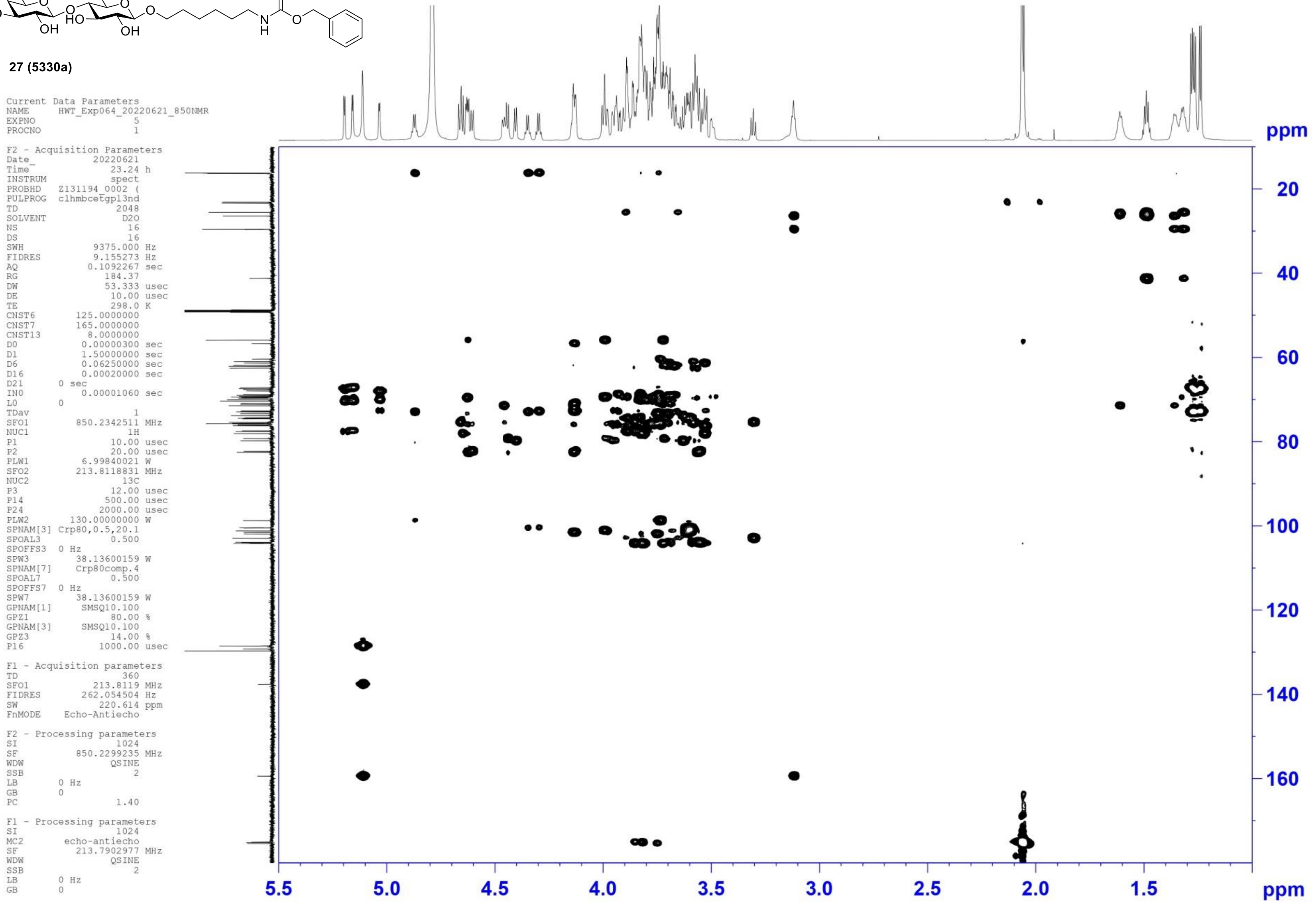

^{1}H-^{13}C HMBC NMR spectrum of **27** (5330a) (850 MHz/214 MHz, D_2O)

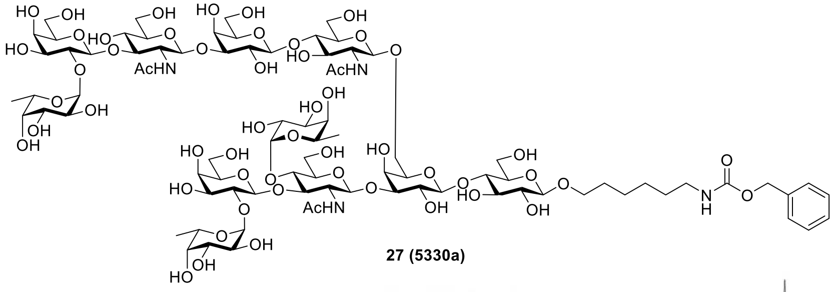

27 (5330a)

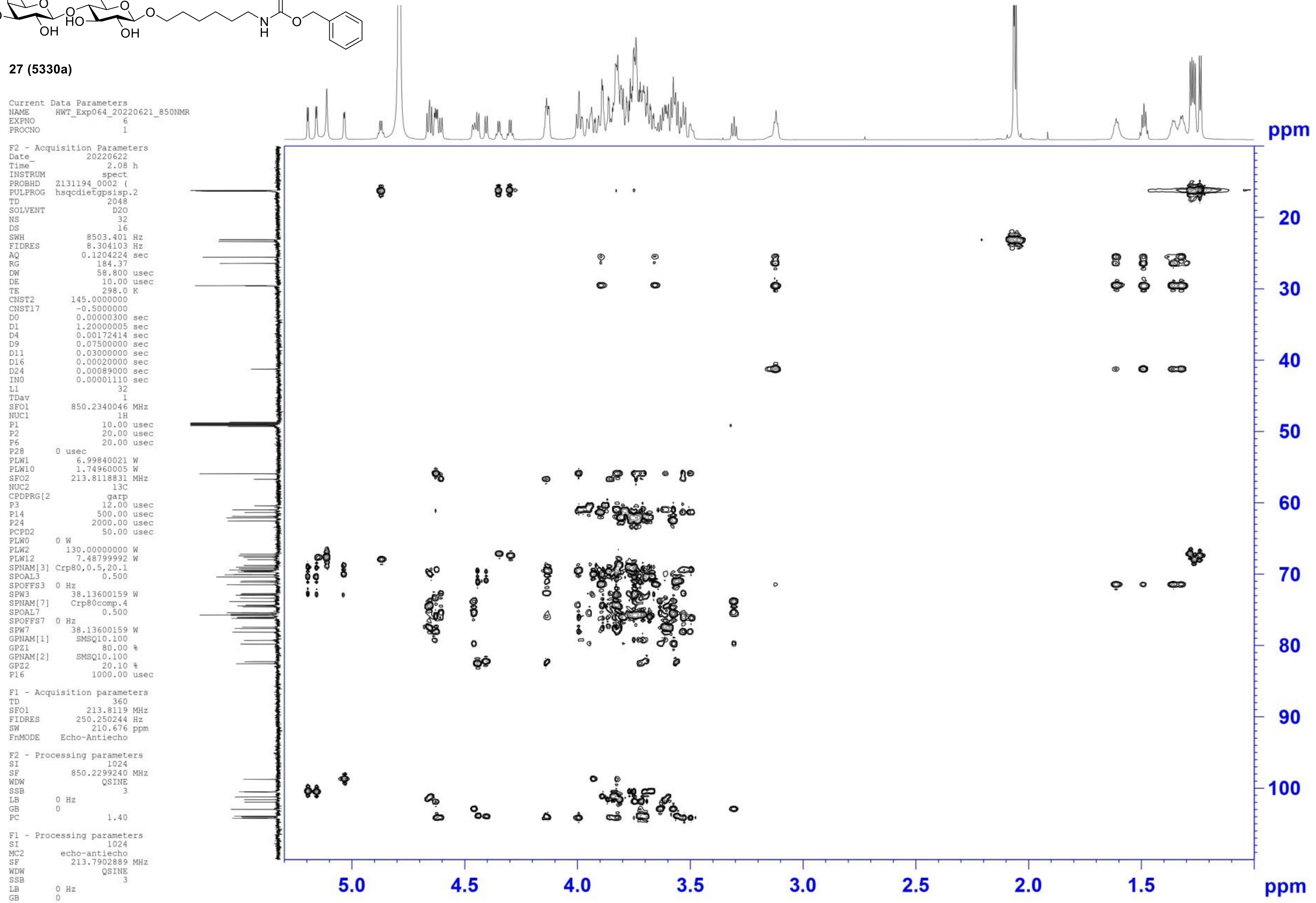

^{1}H-^{13}C HSQC-TOSCY NMR spectrum of **27** (5330a) (850 MHz/214 MHz, D_2O)

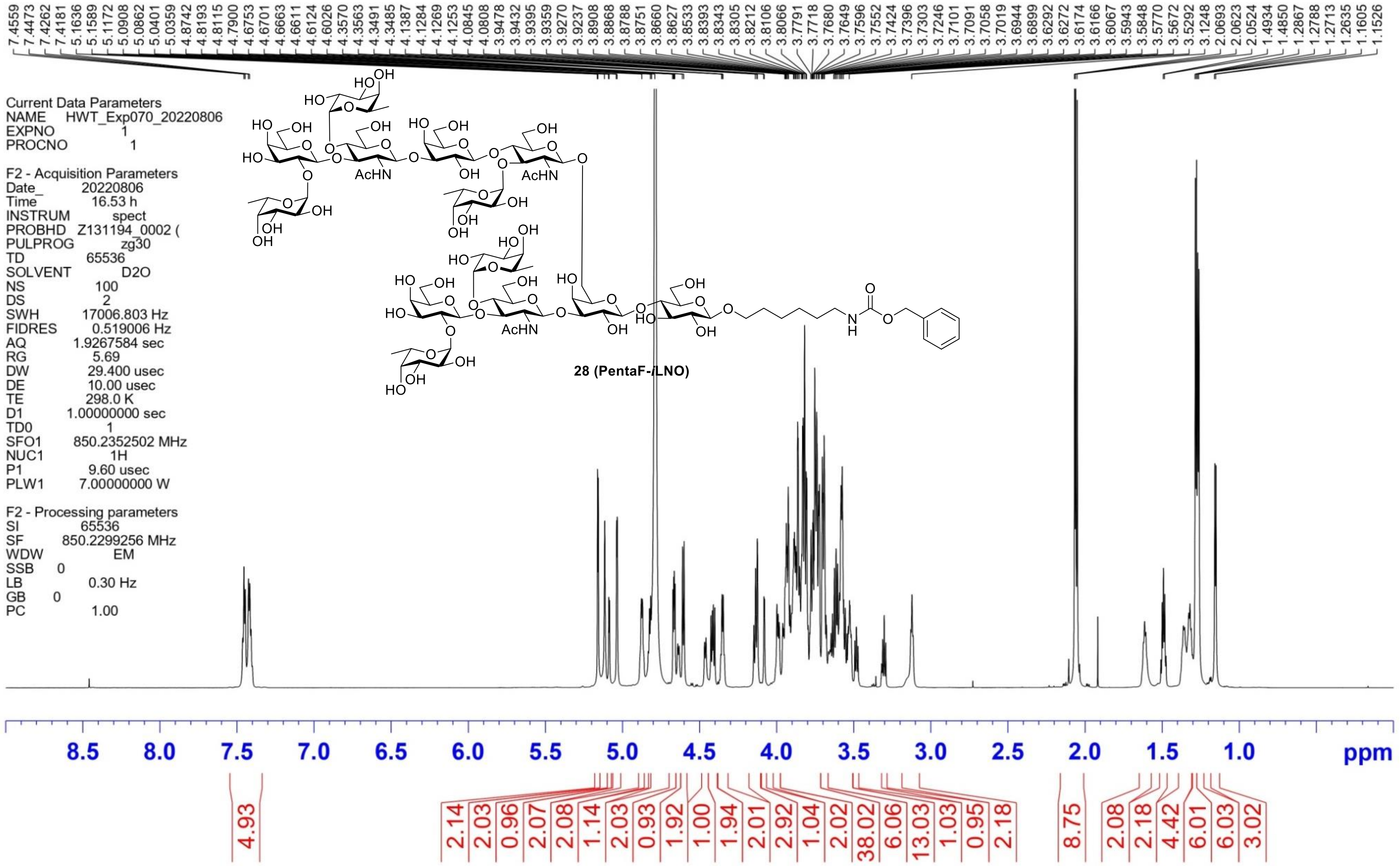

^{1}H NMR spectrum of **28** (PentaF-*i*LNO) (850 MHz, D_2O)

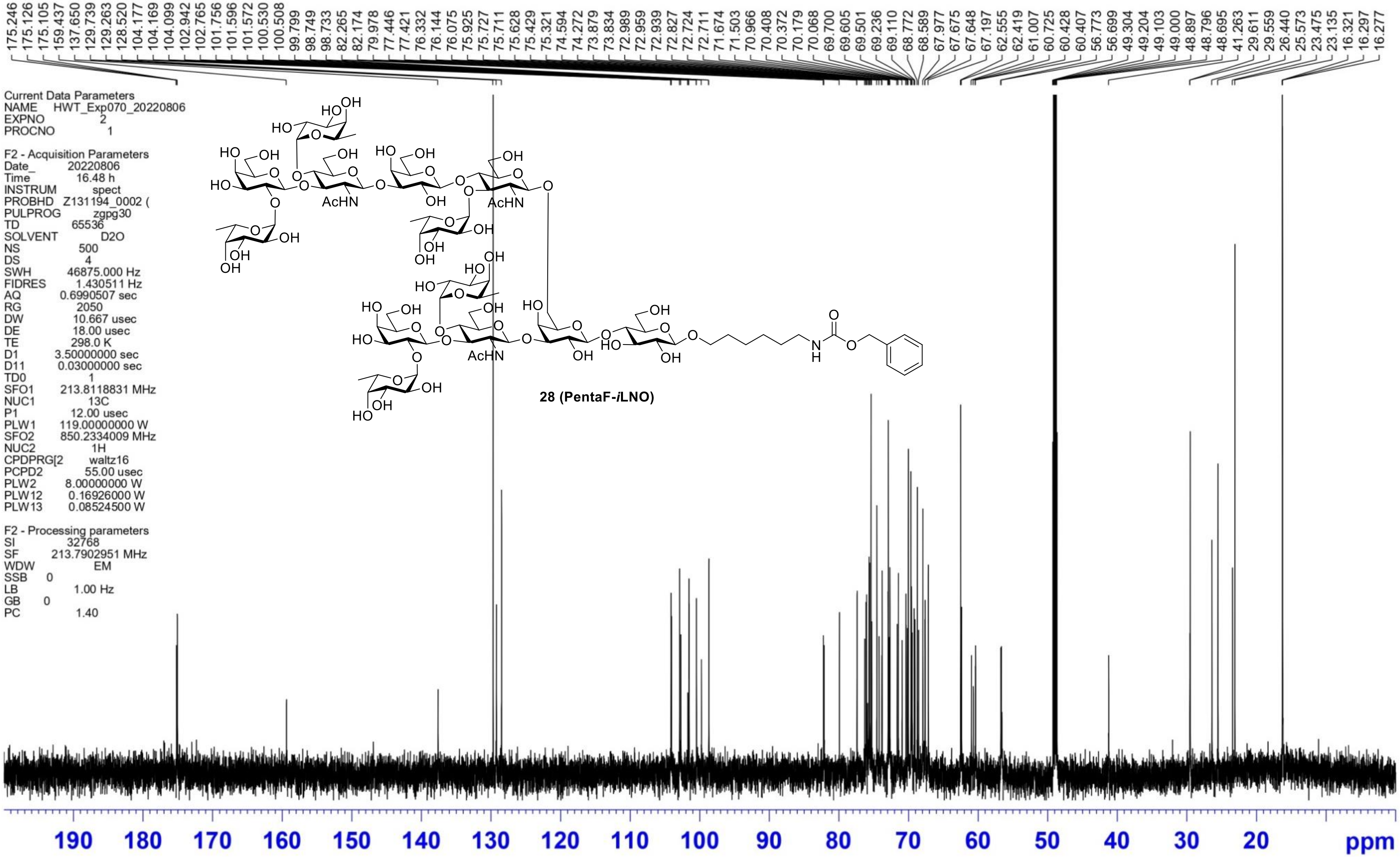

^{13}C NMR spectrum of **28** (PentaF-*i*LNO) (214 MHz, D_2O)

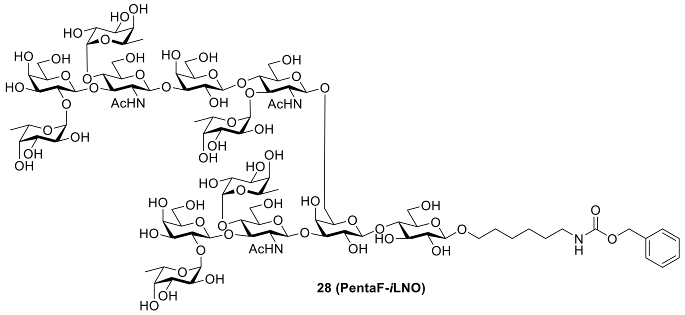

28 (PentaF-*i*LNO)

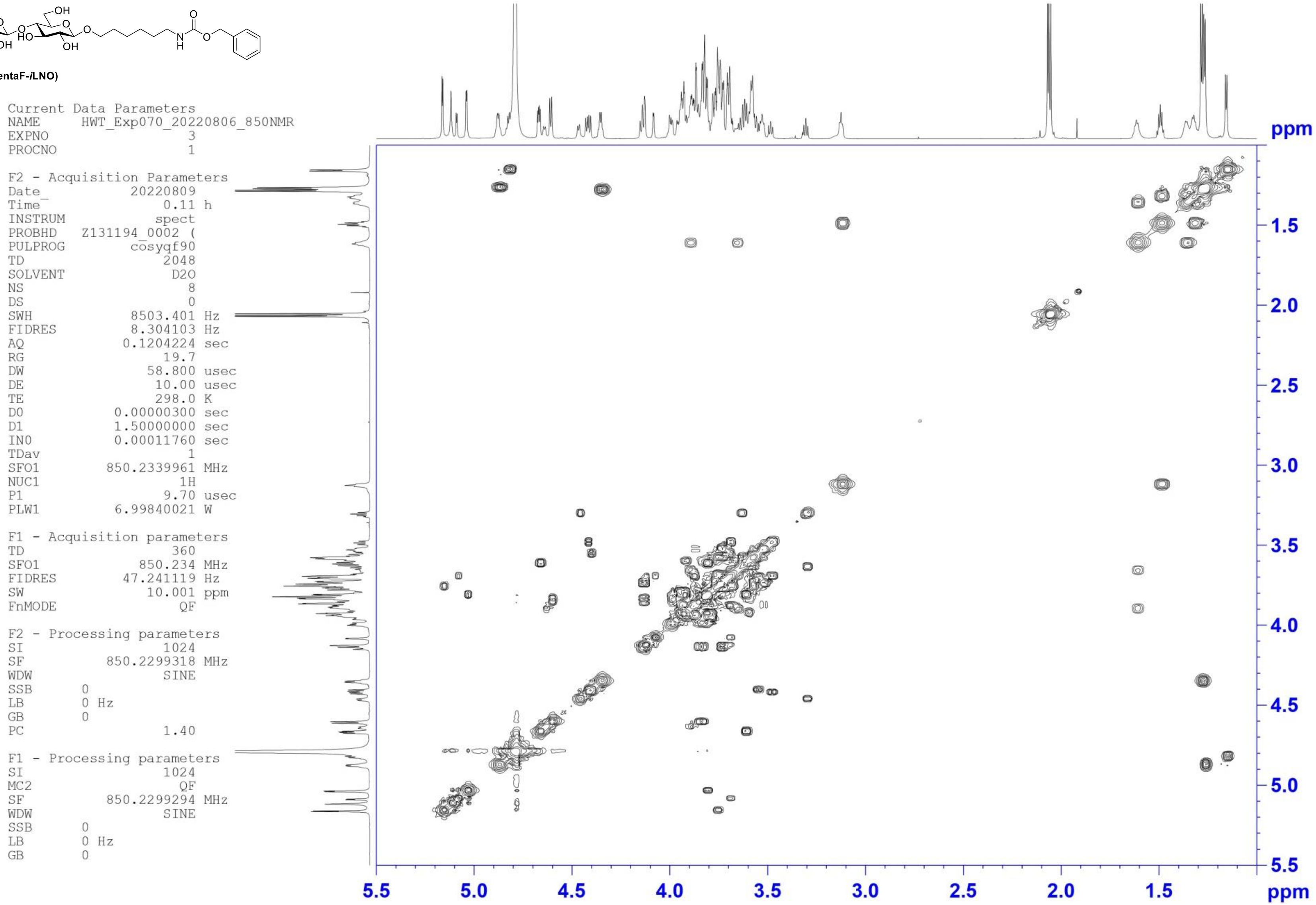

^{1}H-^{1}H COSY NMR spectrum of **28** (PentaF-*i*LNO) (850 MHz, D_2O)

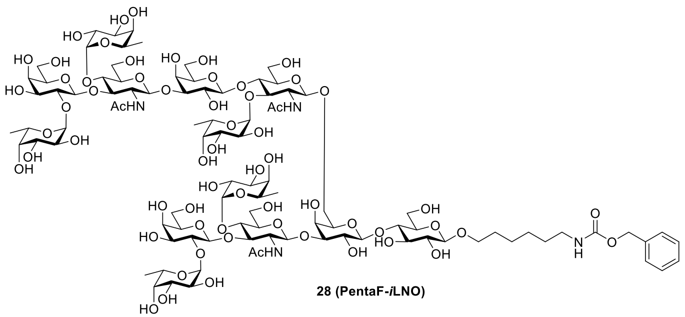

28 (PentaF-*i*LNO)

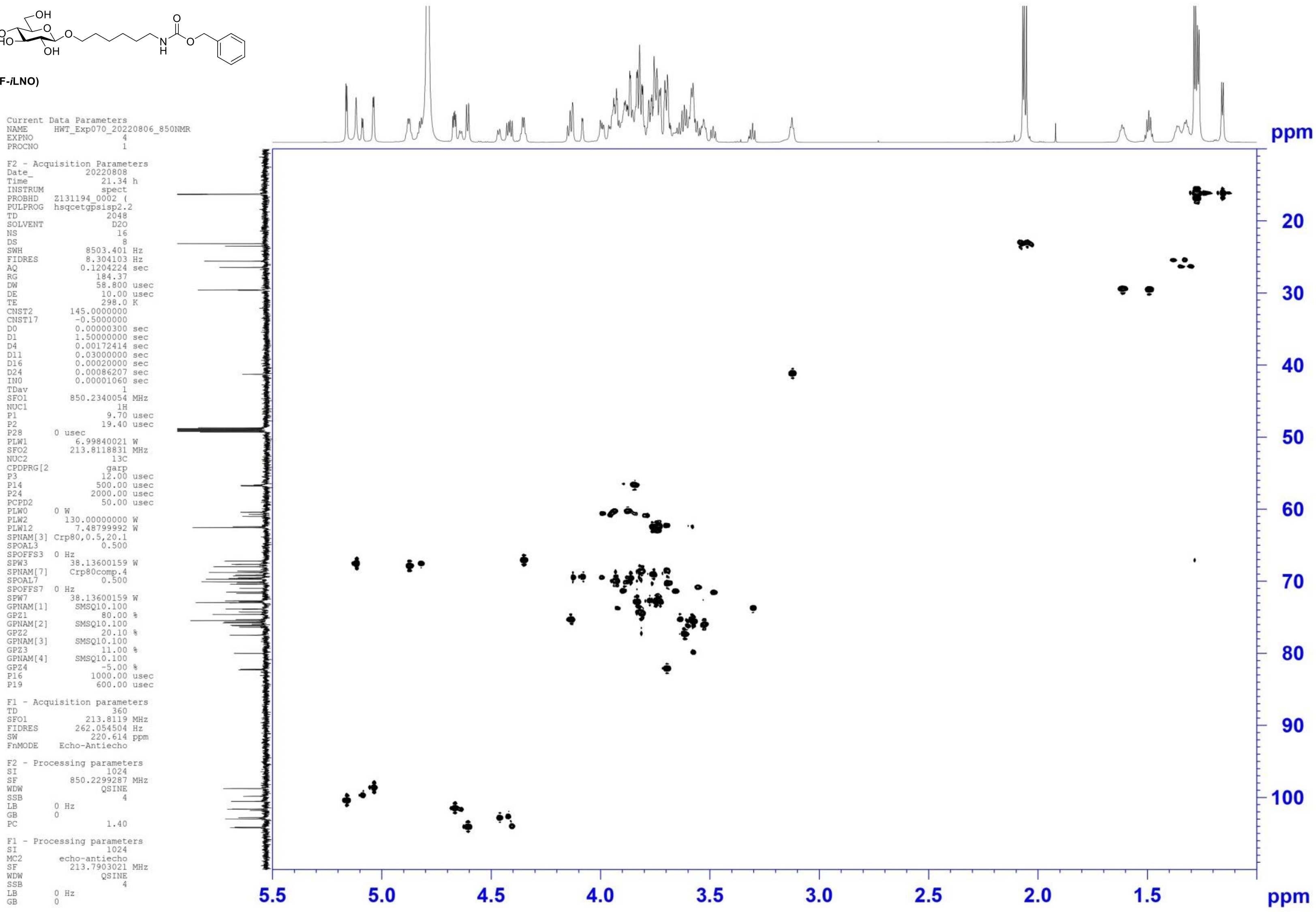

^{1}H-^{13}C HSQC NMR spectrum of **28** (PentaF-*i*LNO) (850 MHz/214 MHz, D_2O)

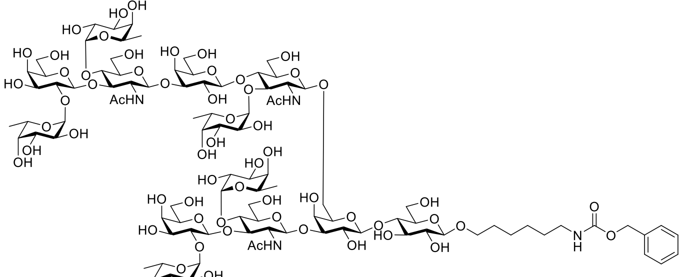

28 (PentaF-*i*LNO)

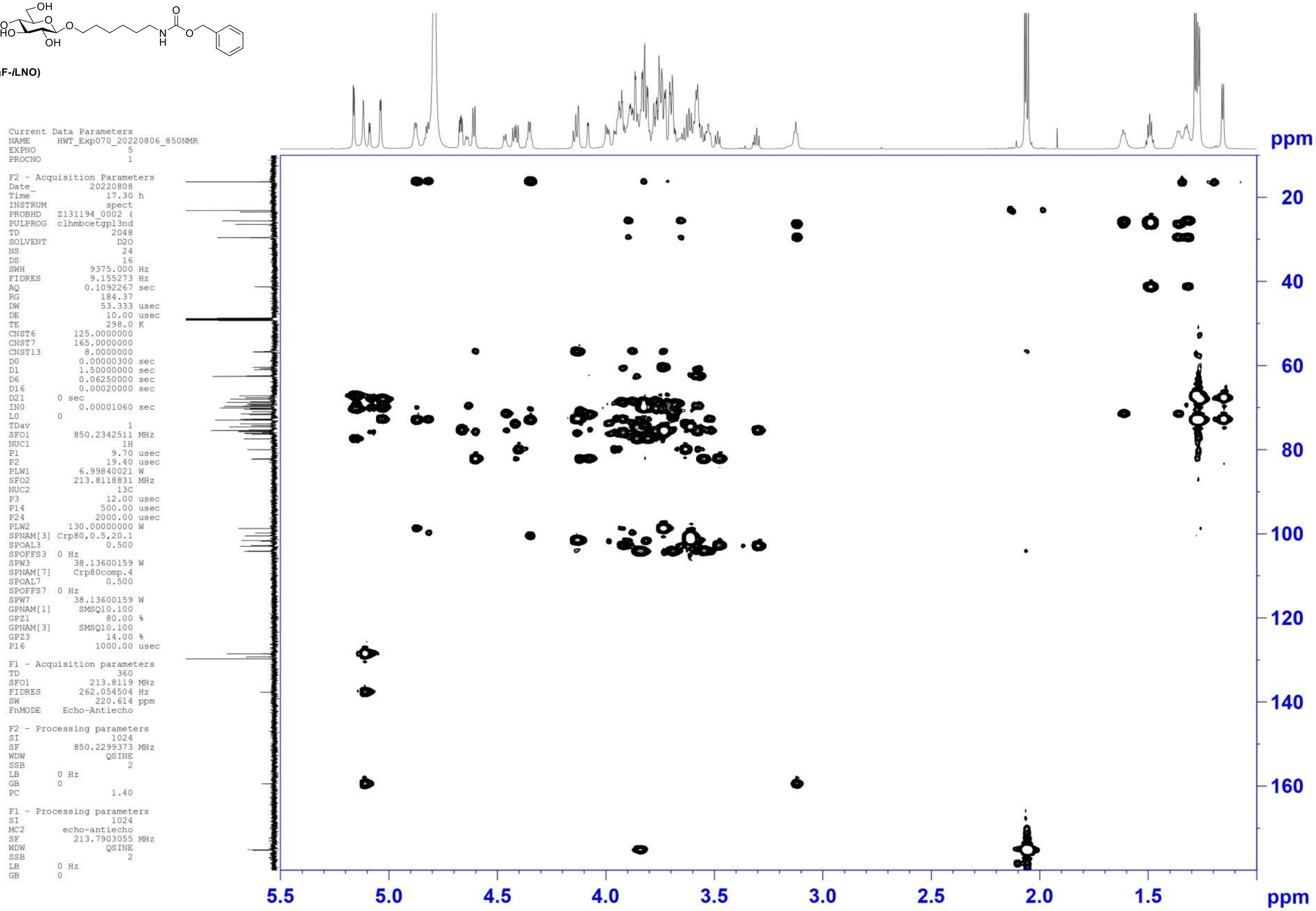

^{1}H-^{13}C HMBC NMR spectrum of **28** (PentaF-*i*LNO) (850 MHz/214 MHz, D_2O)

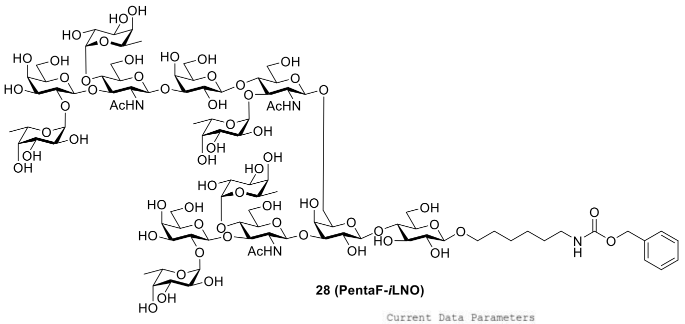

28 (PentaF-*i*LNO)

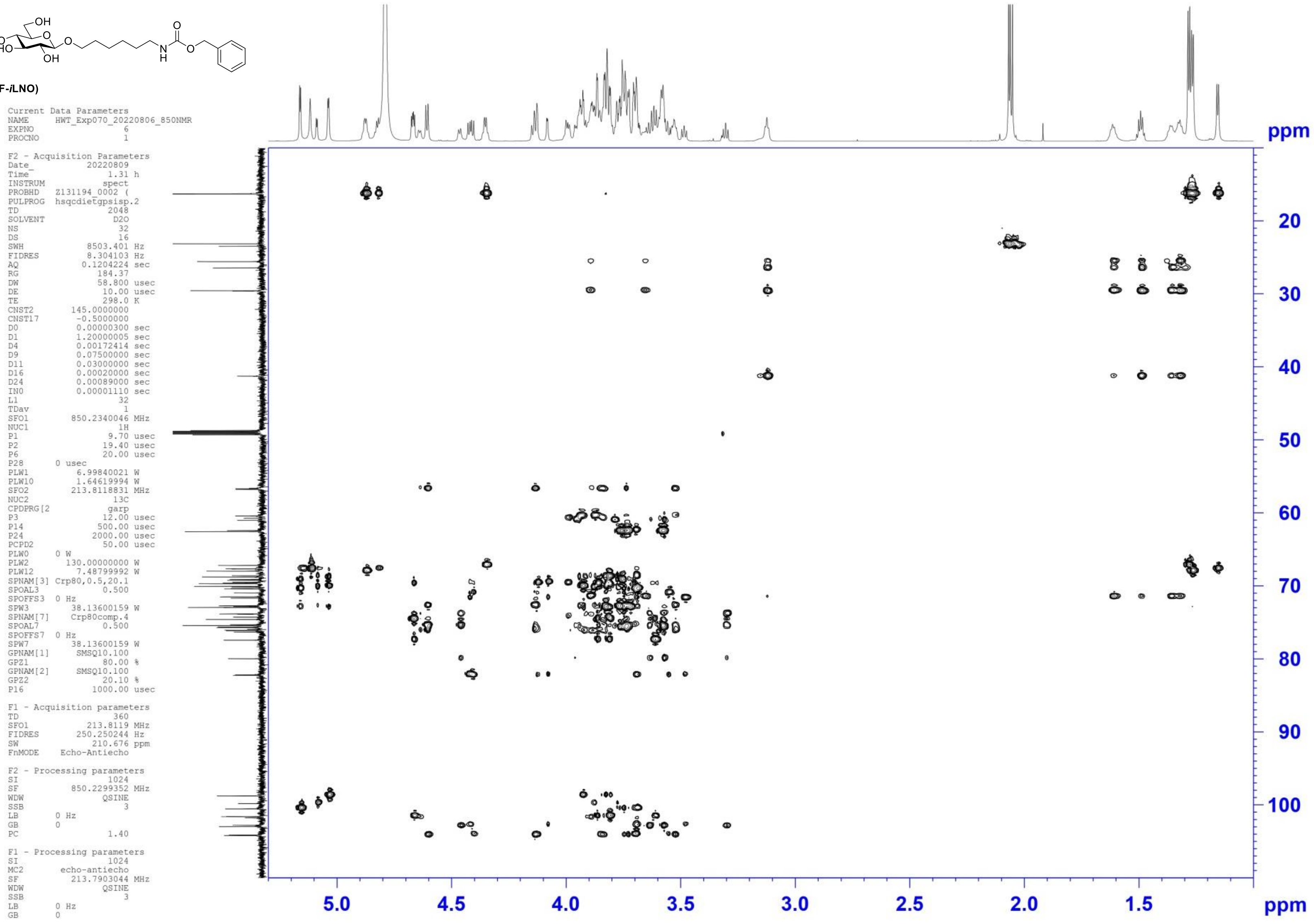

^{1}H-^{13}C HSQC-TOSCY NMR spectrum of **28** (PentaF-*i*LNO) (850 MHz/214 MHz, D_2O)

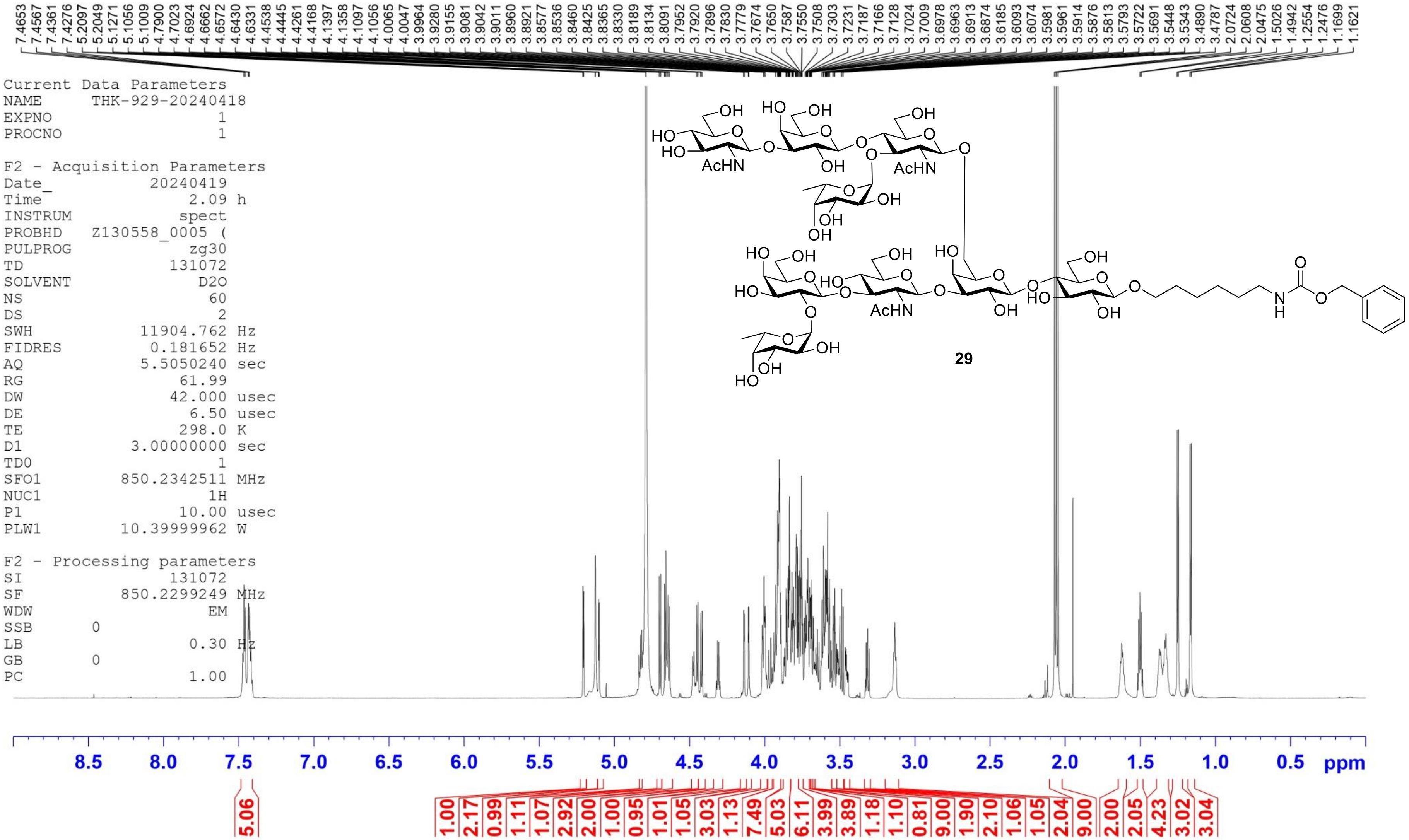

^{1}H NMR spectrum of Compound **29** (850 MHz D_2O)

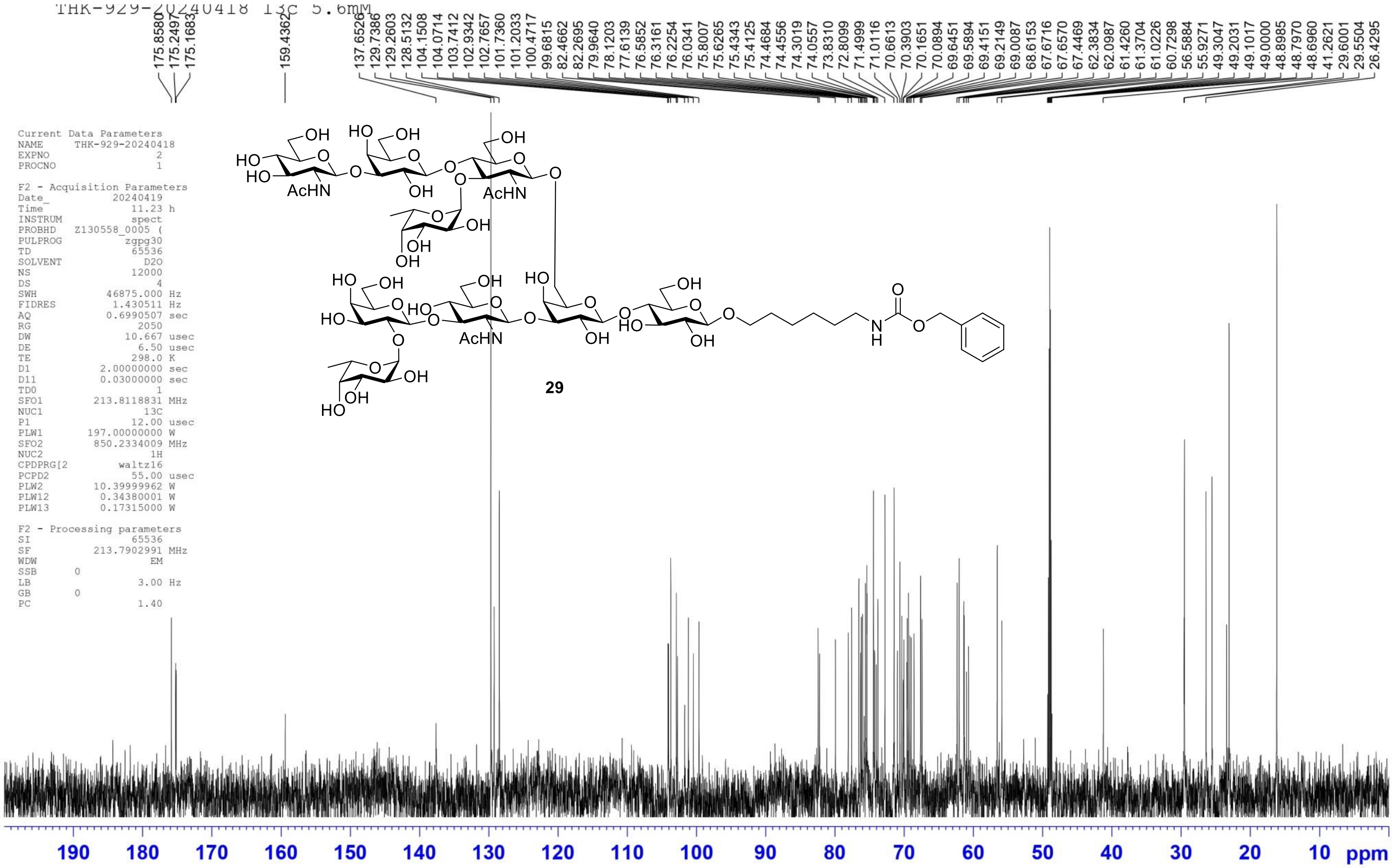

^{13}C NMR spectrum of Compound **29** (214 MHz D_2O)

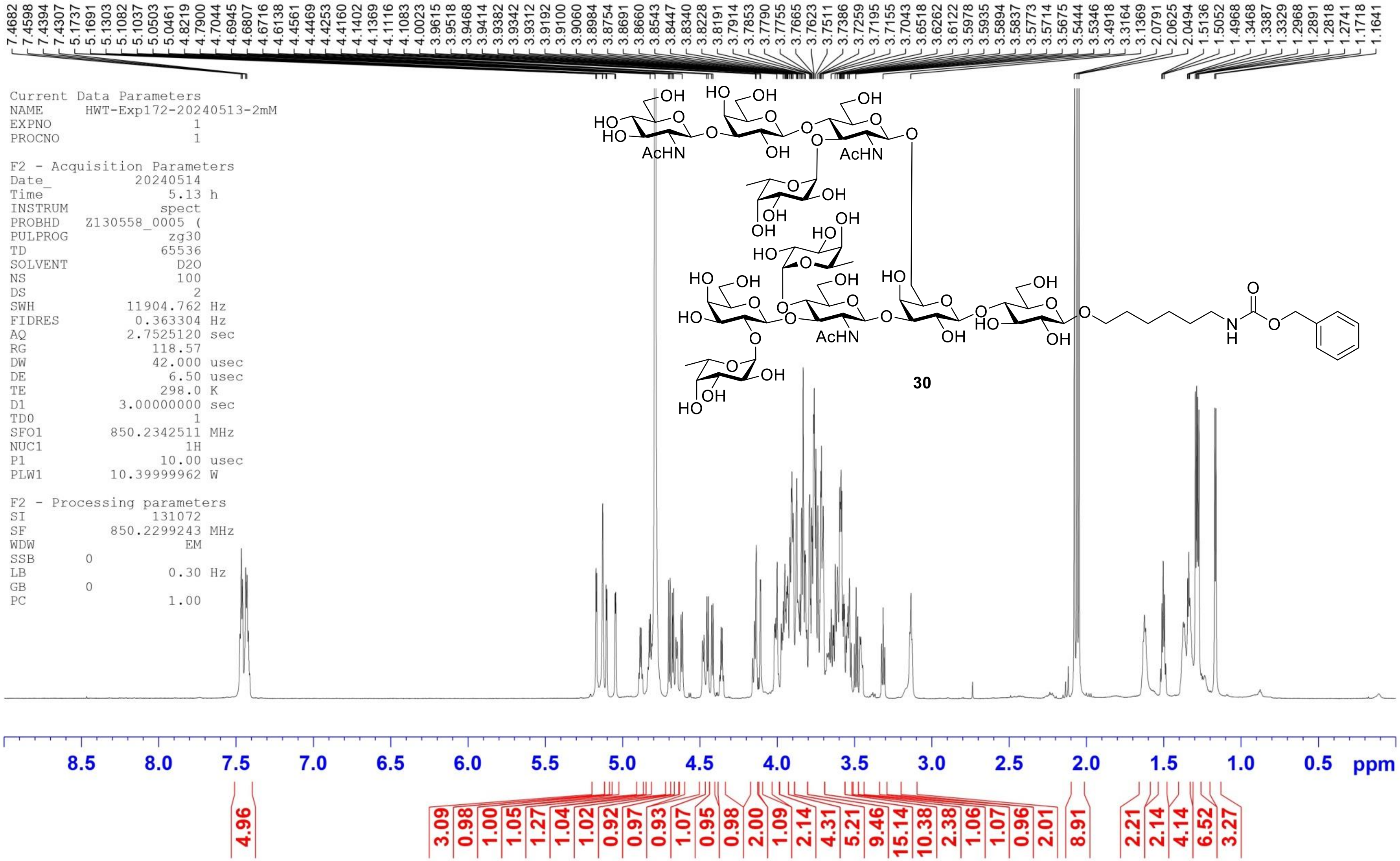

^{1}H NMR spectrum of Compound **30** (850 MHz D_2O)

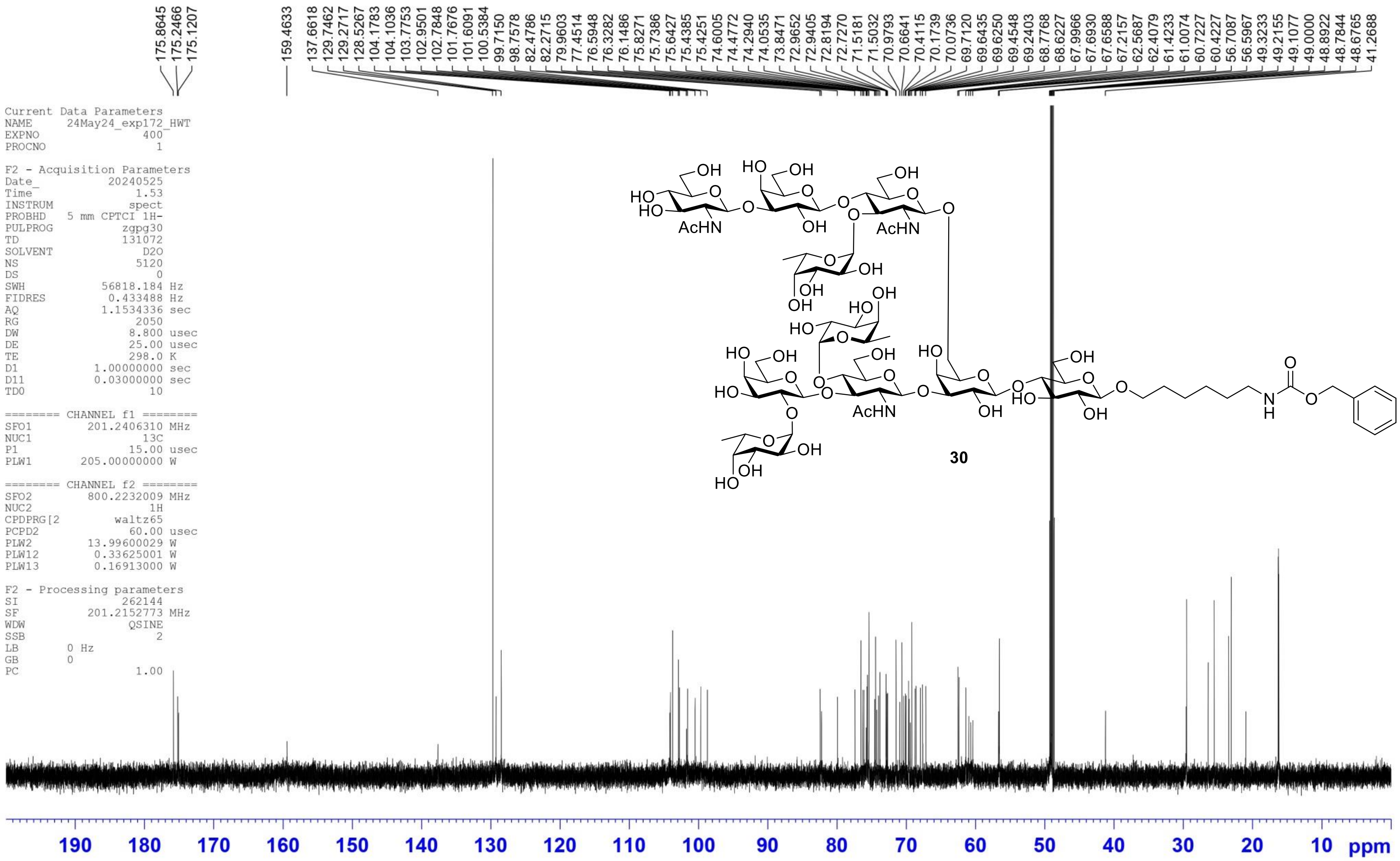

^{13}C NMR spectrum of Compound **30** (201 MHz D_2O)

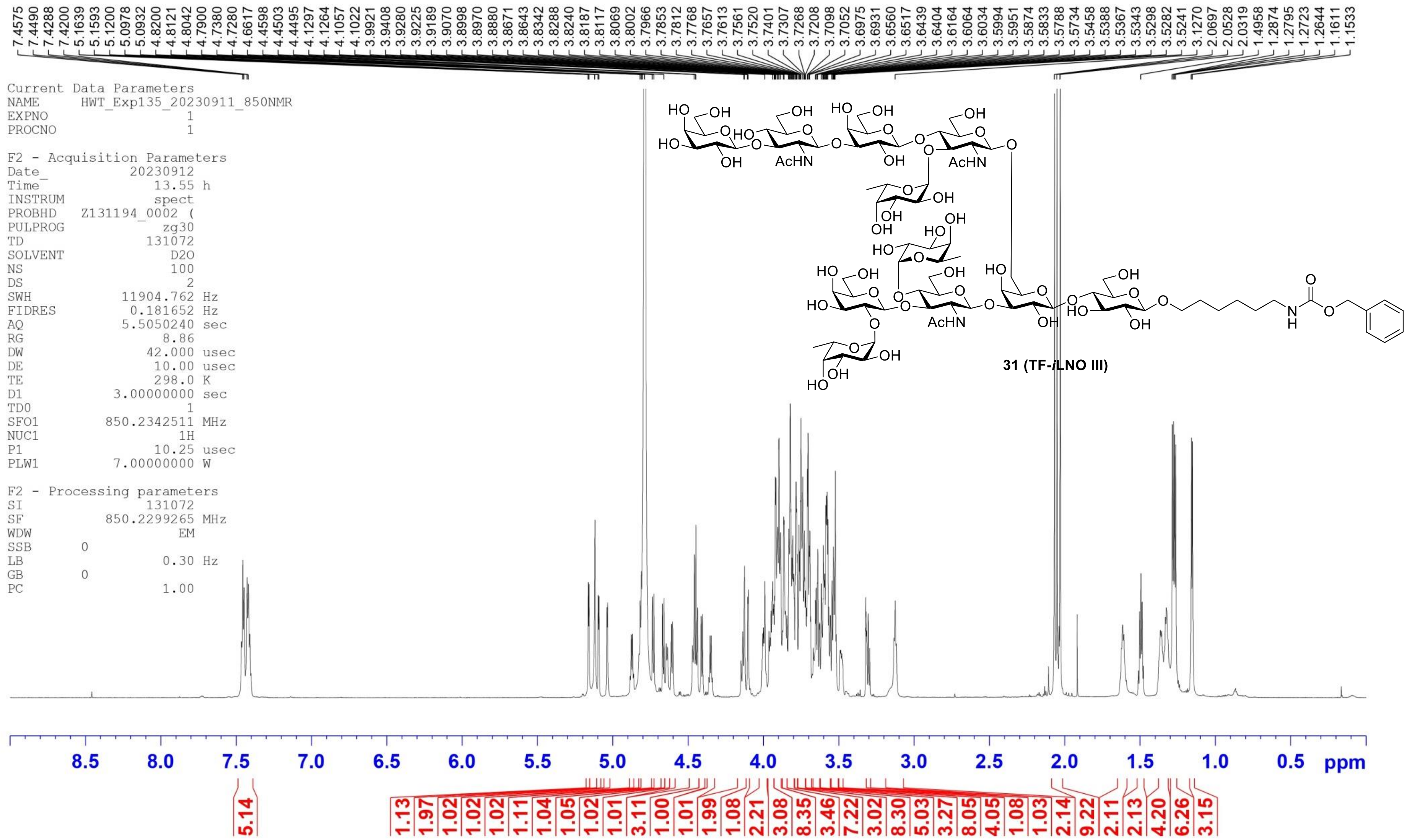

^{1}H NMR spectrum of **31** (TF-*i*LNO III) (850 MHz, D_2O)

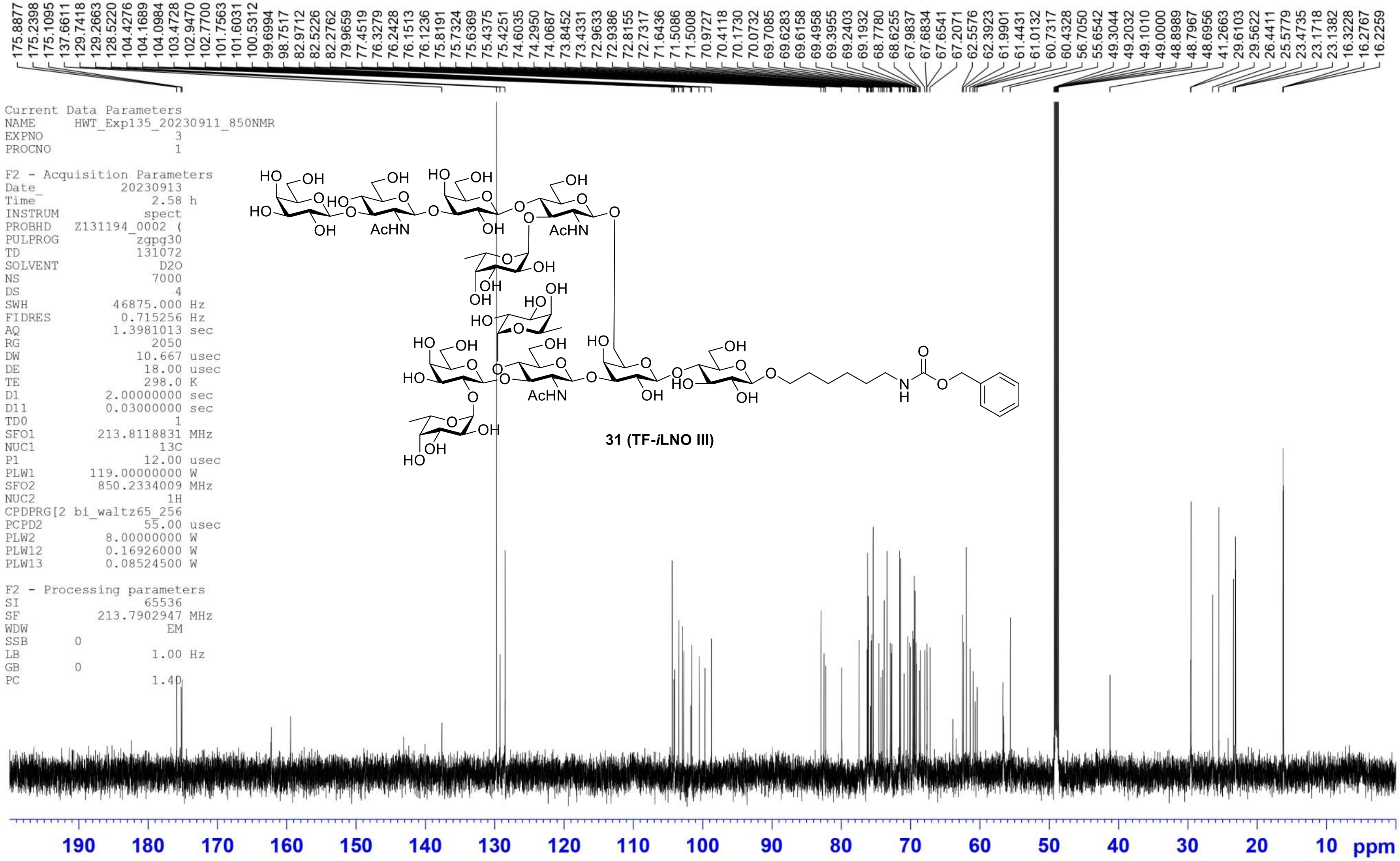

^{13}C NMR spectrum of **31** (TF-*i*LNO III) (214 MHz, D_2O)

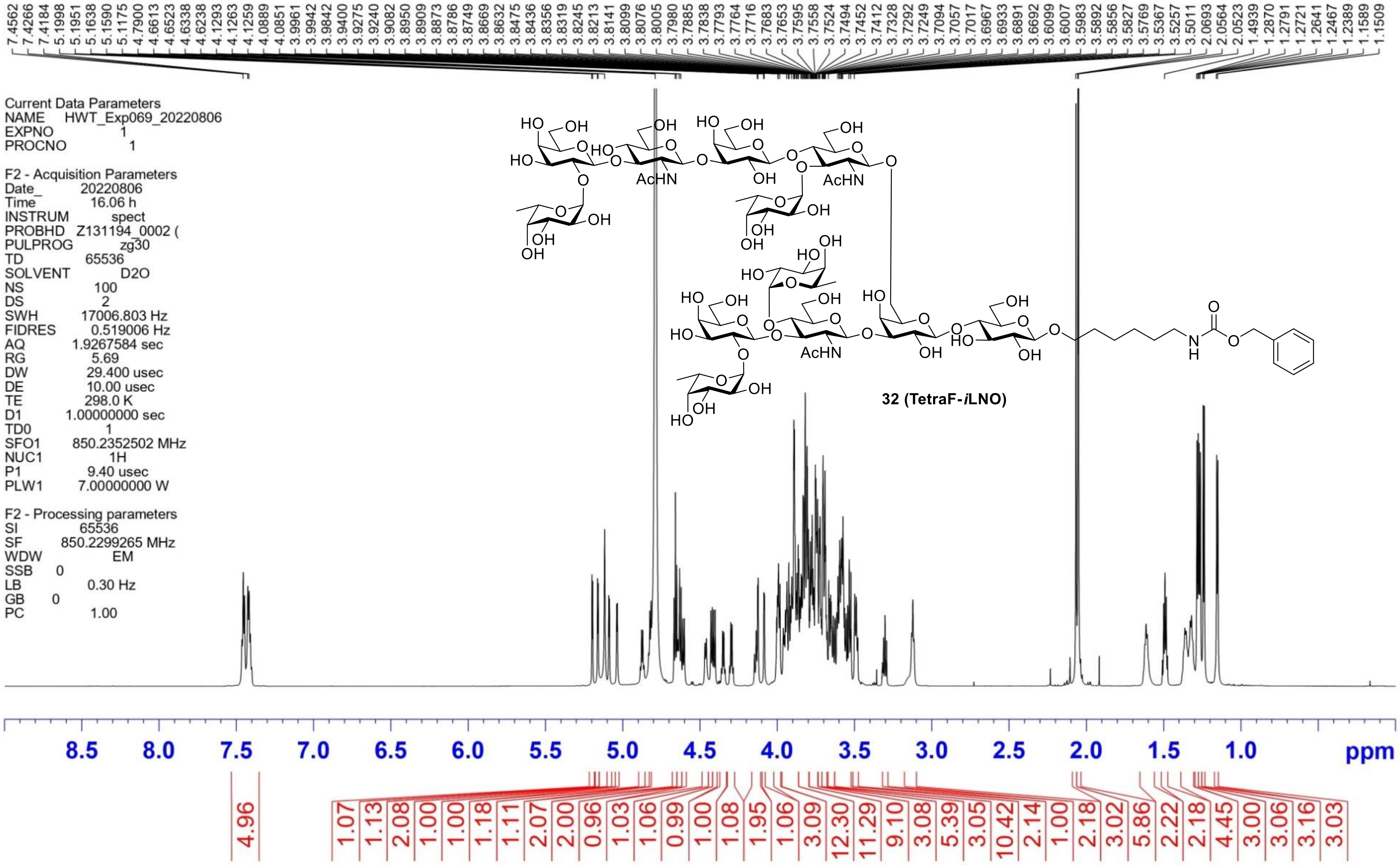

^{1}H NMR spectrum of **32** (TetraF-*i*LNO) (850 MHz, D_2O)

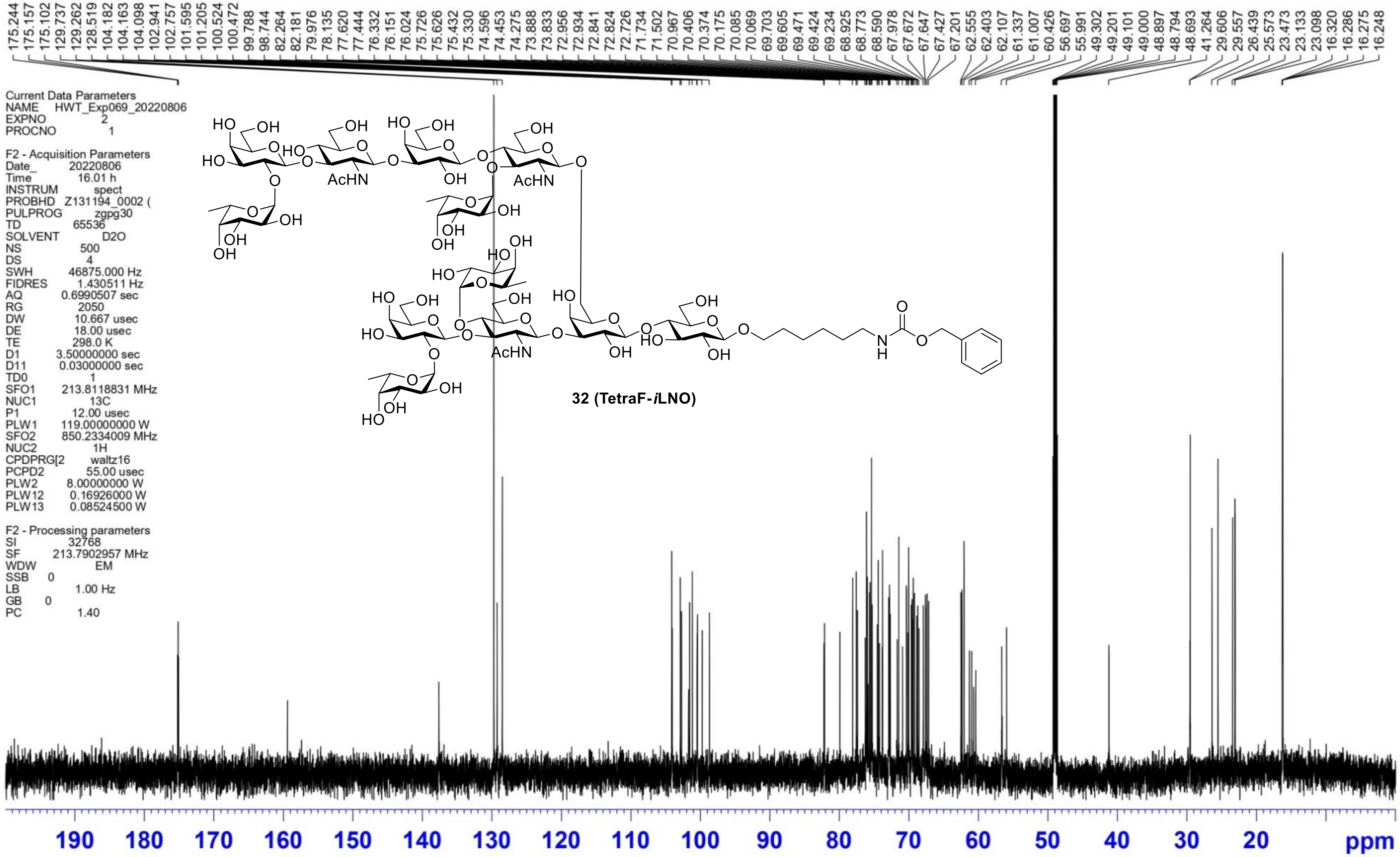

^{13}C NMR spectrum of **32** (TetraF-*i*LNO) (214 MHz, D_2O)

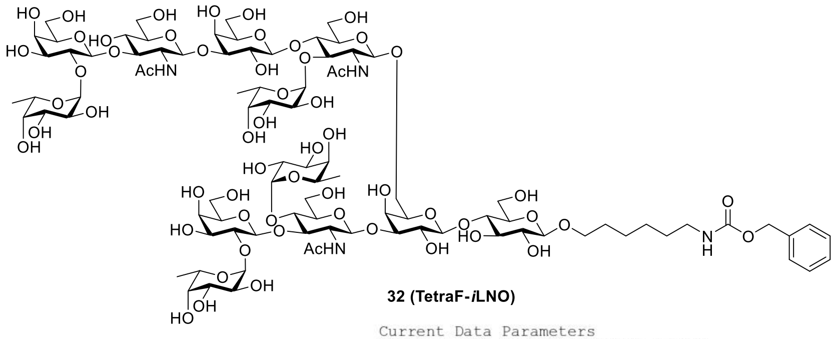


```
Current Data Parameters
NAME      HWT_Exp069_20220806_850NMR
EXPNO              3
PROCNO             1

F2 - Acquisition Parameters
Date_          20220807
Time              21.11 h
INSTRUM           spect
PROBHD   Z131194_0002 (
PULPROG         cosyqf90
TD                 2048
SOLVENT             D2O
NS                    8
DS                    0
SWH            8503.401 Hz
FIDRES         8.304103 Hz
AQ            0.1204224 sec
RG                16.45
DW               58.800 usec
DE                10.00 usec
TE                298.0 K
D0           0.00000300 sec
D1           1.50000000 sec
IN0          0.00011760 sec
TDav                  1
SFO1        850.2339961 MHz
NUC1                 1H
P1                 9.40 usec
PLW1         6.99840021 W

F1 - Acquisition parameters
TD                  360
SFO1            850.234 MHz
FIDRES        47.241119 Hz
SW               10.001 ppm
FnMODE               QF

F2 - Processing parameters
SI                 1024
SF          850.2299235 MHz
WDW                SINE
SSB      0
LB       0 Hz
GB       0
PC                 1.40

F1 - Processing parameters
SI                 1024
MC2                  QF
SF          850.2299235 MHz
WDW                SINE
SSB      0
LB       0 Hz
GB       0
```

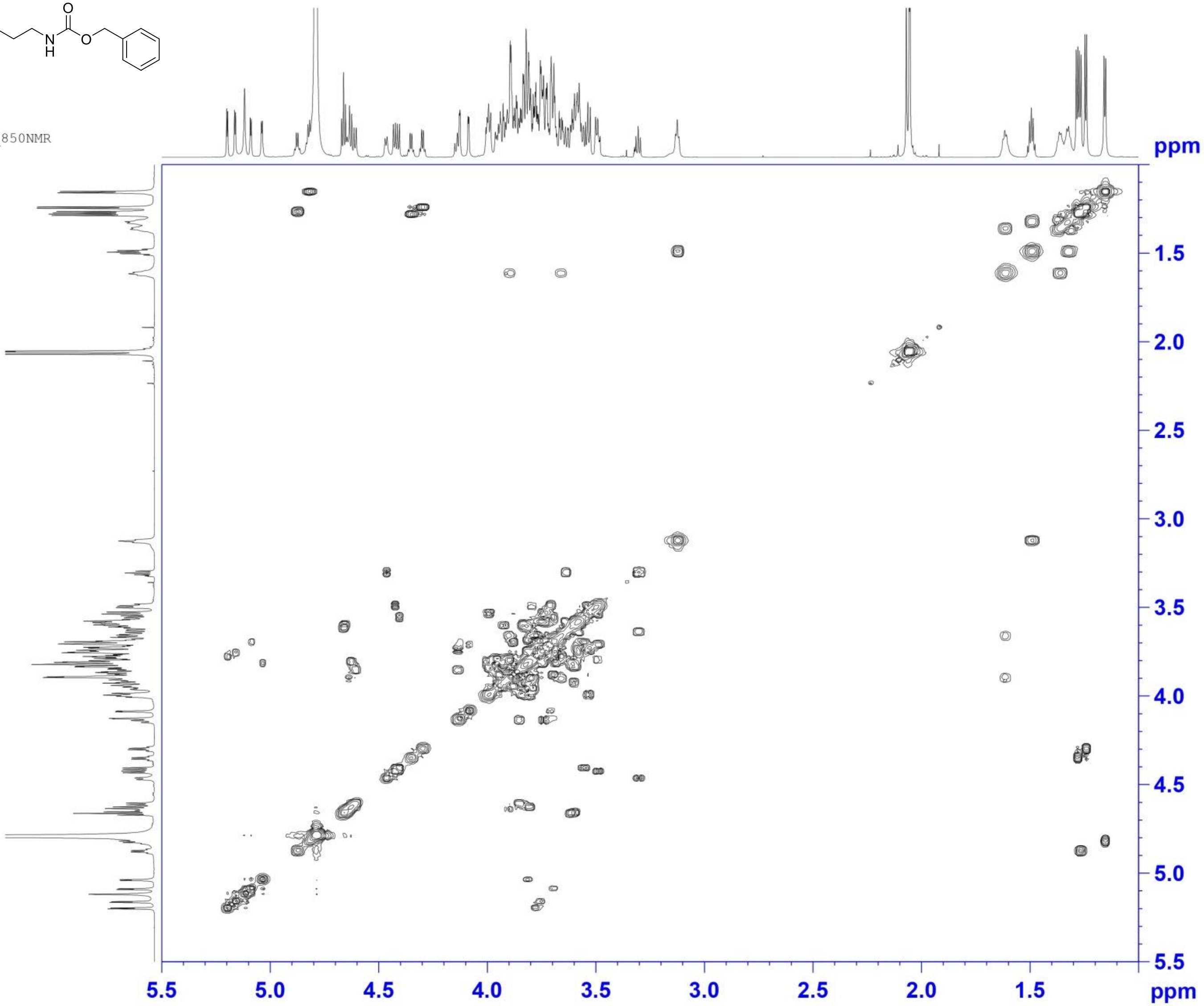

^{1}H-^{1}H COSY NMR spectrum of **32** (TetraF-*i*LNO) (850 MHz, D_2O)

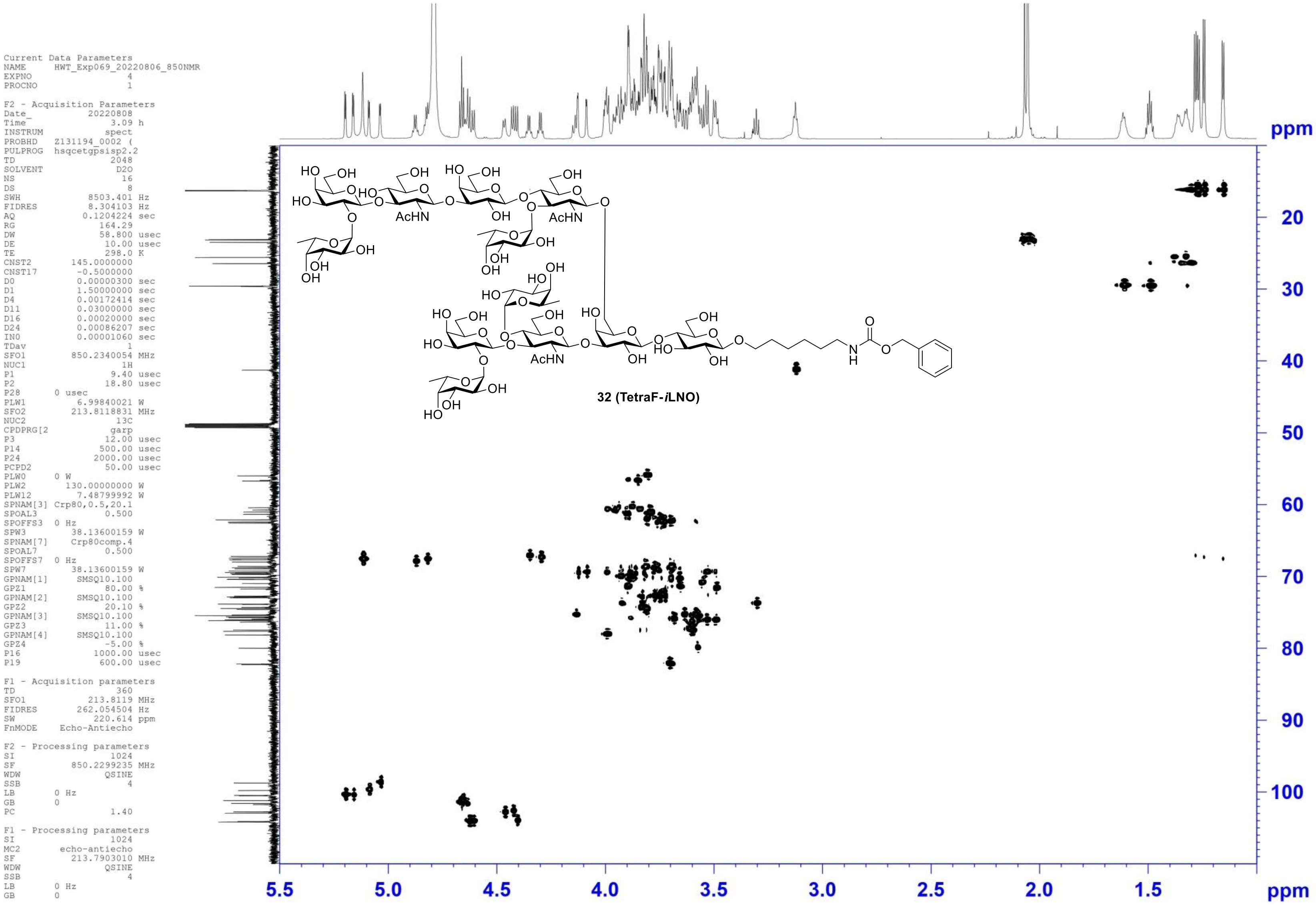

^{1}H-^{13}C HSQC NMR spectrum of **32** (TetraF-*i*LNO) (850 MHz/214 MHz, D_2O)

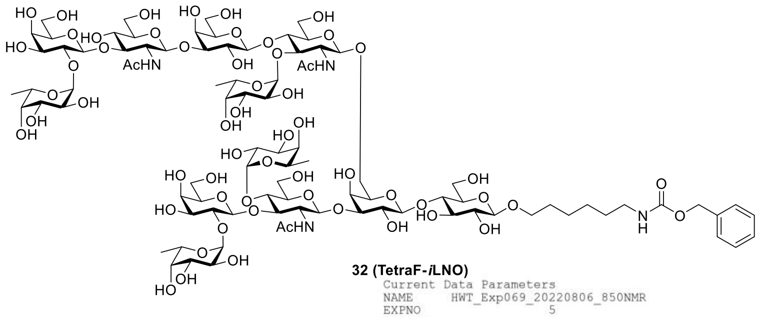

32 (TetraF-*i*LNO)

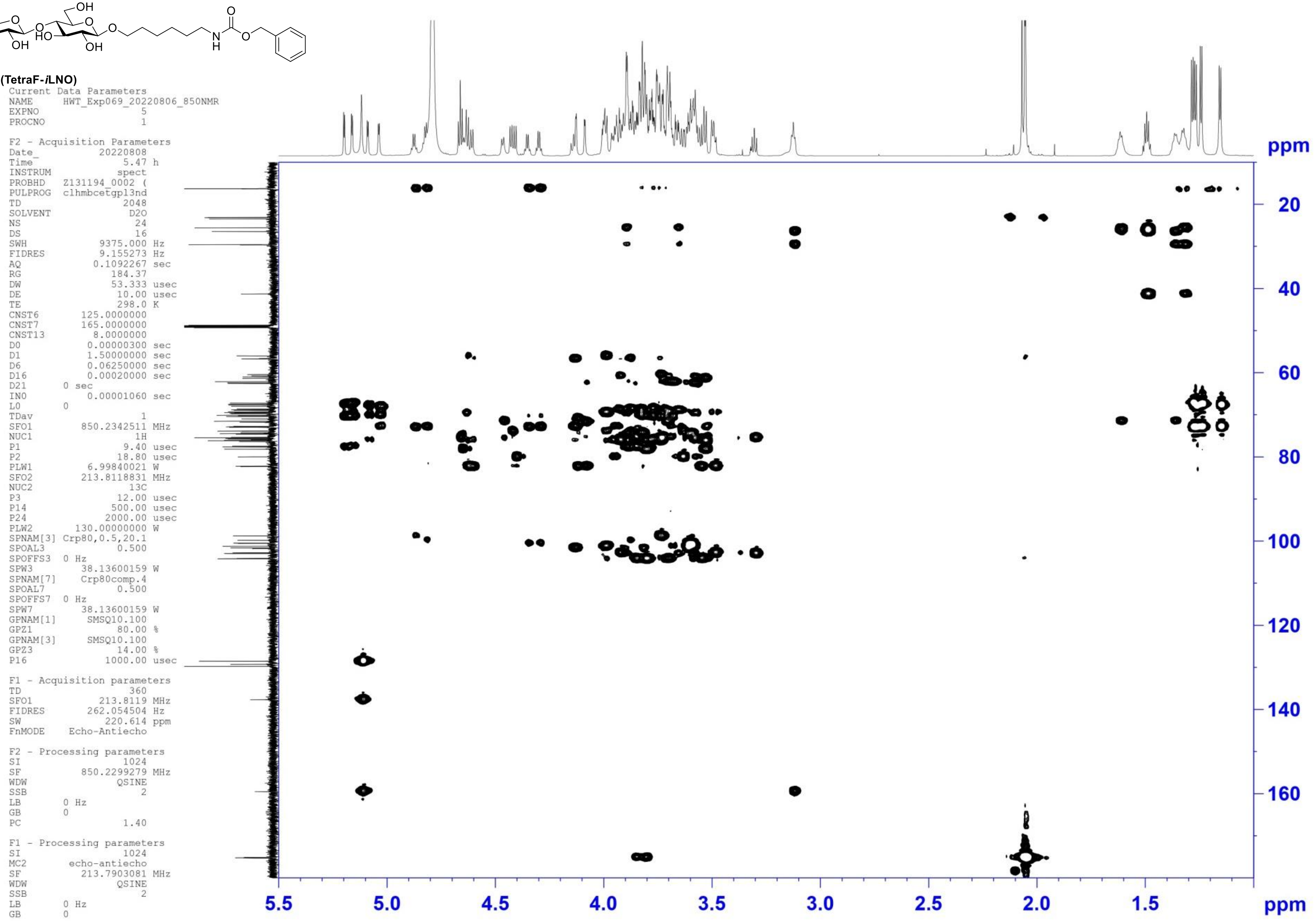

^{1}H-^{13}C HMBC NMR spectrum of **32** (TetraF-*i*LNO) (850 MHz/214 MHz, D_2O)

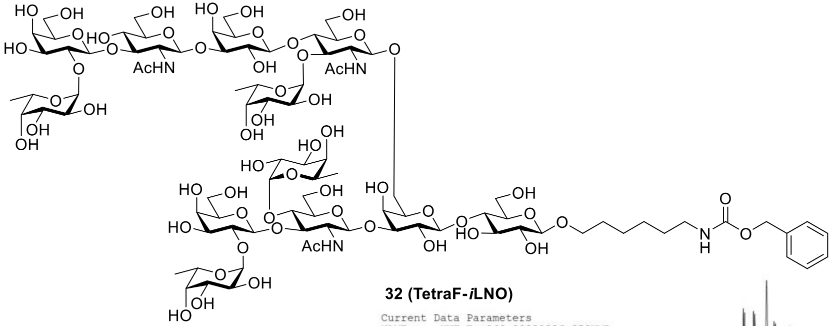

32 (TetraF-*i*LNO)

```
Current Data Parameters
NAME     HWT_Exp069_20220806_850NMR
EXPNO              6
PROCNO             1

F2 - Acquisition Parameters
Date_          20220807
Time              22.31 h
INSTRUM           spect
PROBHD   Z131194_0002 (
PULPROG  hsqcdietgpsisp.2
TD                 2048
SOLVENT             D2O
NS                   32
DS                   16
SWH            8503.401 Hz
FIDRES         8.304103 Hz
AQ            0.1204224 sec
RG               184.37
DW               58.800 usec
DE                10.00 usec
TE                298.0 K
CNST2       145.0000000
CNST17       -0.5000000
D0           0.00000300 sec
D1           1.20000005 sec
D4           0.00172414 sec
D9           0.07500000 sec
D11          0.03000000 sec
D16          0.00020000 sec
D24          0.00089000 sec
IN0          0.00001110 sec
L1                   32
TDav                  1
SFO1        850.2340046 MHz
NUC1                 1H
P1                 9.40 usec
P2                18.80 usec
P6                20.00 usec
P28      0 usec
PLW1         6.99840021 W
PLW10        1.54600000 W
SFO2        213.8118831 MHz
NUC2                13C
CPDPRG[2           garp
P3                12.00 usec
P14              500.00 usec
P24             2000.00 usec
PCPD2             50.00 usec
PLW0     0 W
PLW2       130.00000000 W
PLW12        7.48799992 W
SPNAM[3] Crp80,0.5,20.1
SPOAL3            0.500
SPOFFS3  0 Hz
SPW3        38.13600159 W
SPNAM[7]    Crp80comp.4
SPOAL7            0.500
SPOFFS7  0 Hz
SPW7        38.13600159 W
GPNAM[1]     SMSQ10.100
GPZ1              80.00 %
GPNAM[2]     SMSQ10.100
GPZ2              20.10 %
P16             1000.00 usec

F1 - Acquisition parameters
TD                  360
SFO1           213.8119 MHz
FIDRES       250.250244 Hz
SW              210.676 ppm
FnMODE    Echo-Antiecho

F2 - Processing parameters
SI                 1024
SF          850.2299276 MHz
WDW               QSINE
SSB                   3
LB       0 Hz
GB       0
PC                 1.40

F1 - Processing parameters
SI                 1024
MC2        echo-antiecho
SF          213.7903040 MHz
WDW               QSINE
SSB                   3
LB       0 Hz
GB       0
```

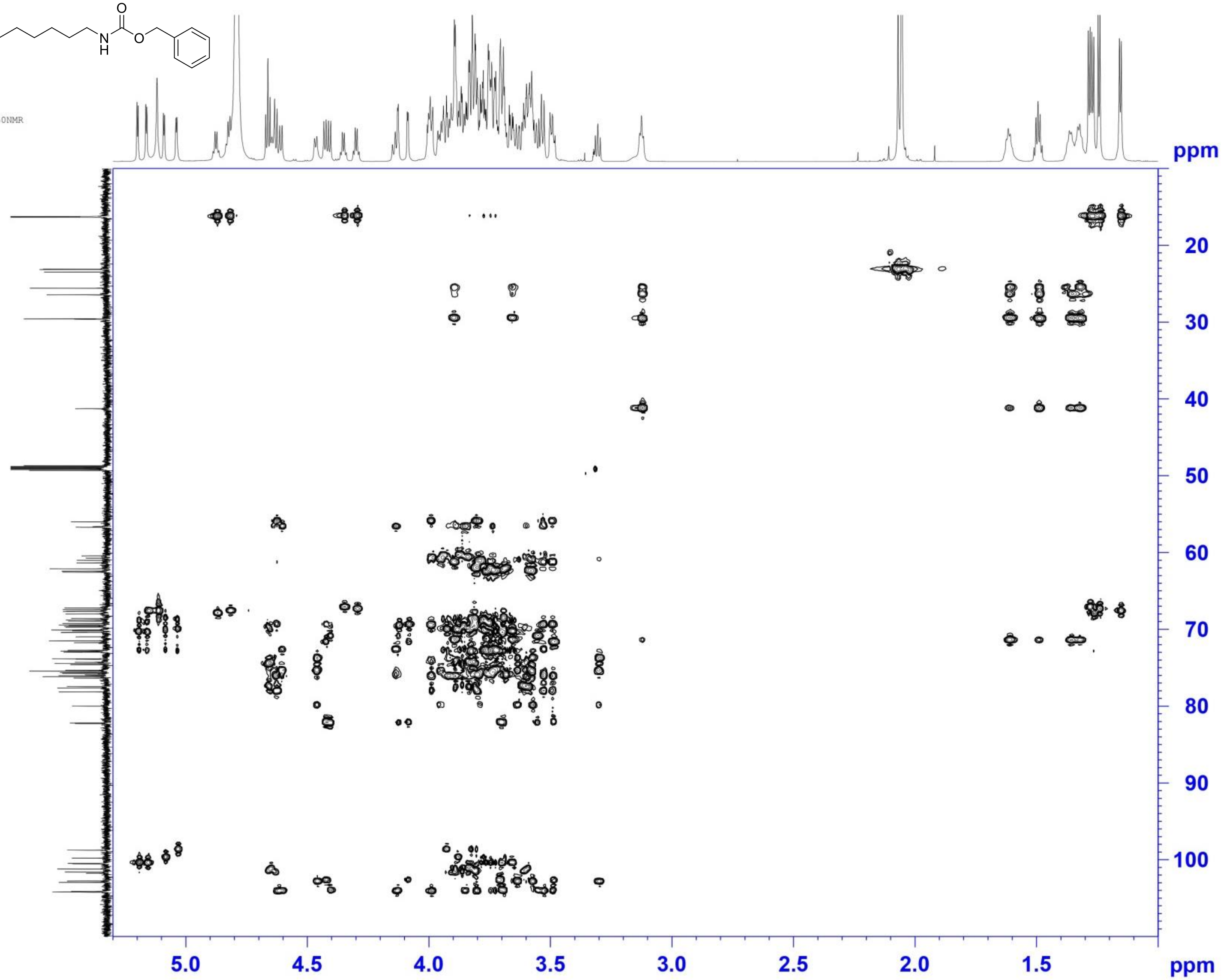

^{1}H-^{13}C HSQC-TOSCY NMR spectrum of **32** (TetraF-*i*LNO) (850 MHz/214 MHz, D_2O)

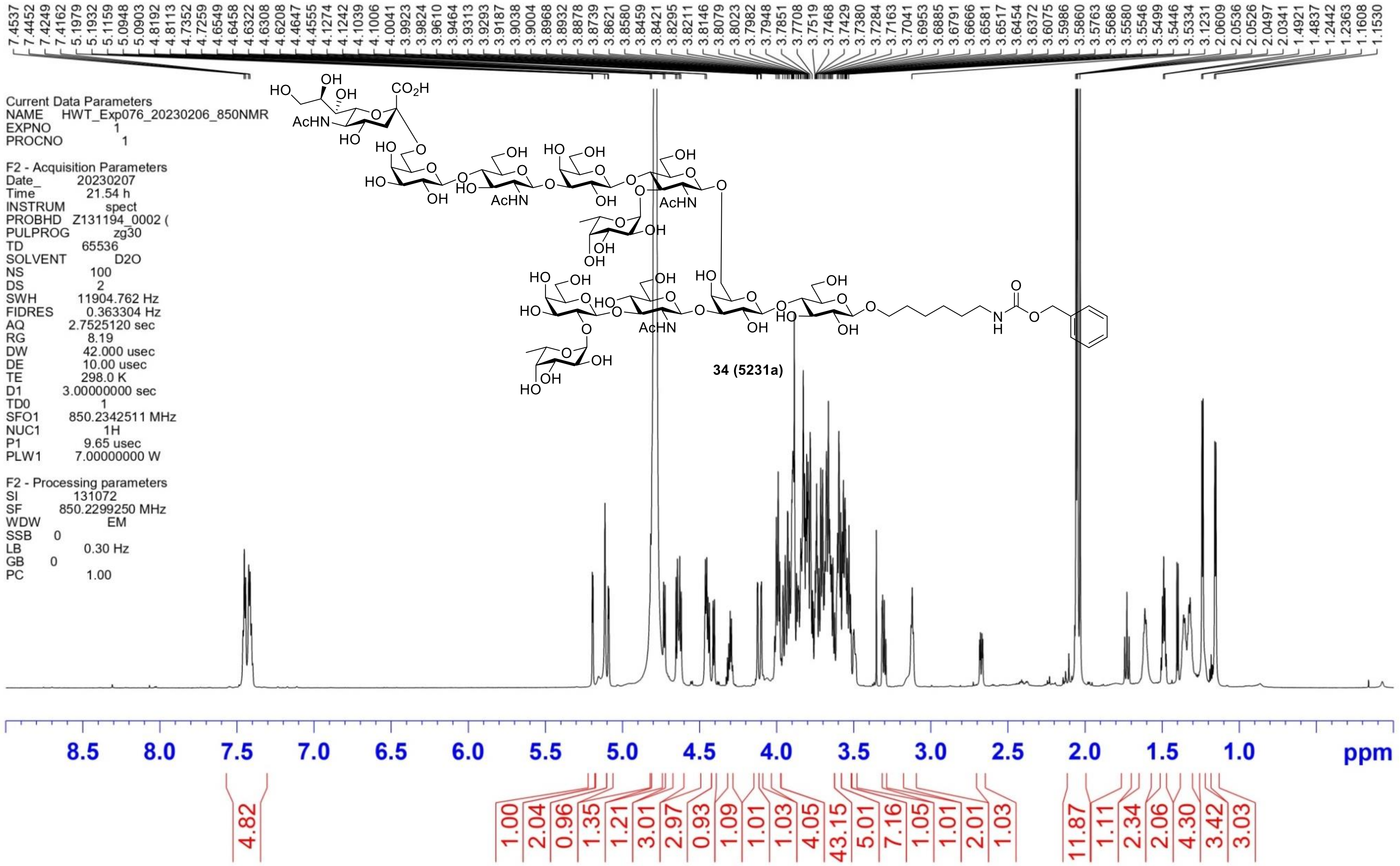

^{1}H NMR spectrum of **34** (5231a) (850 MHz, D_2O)

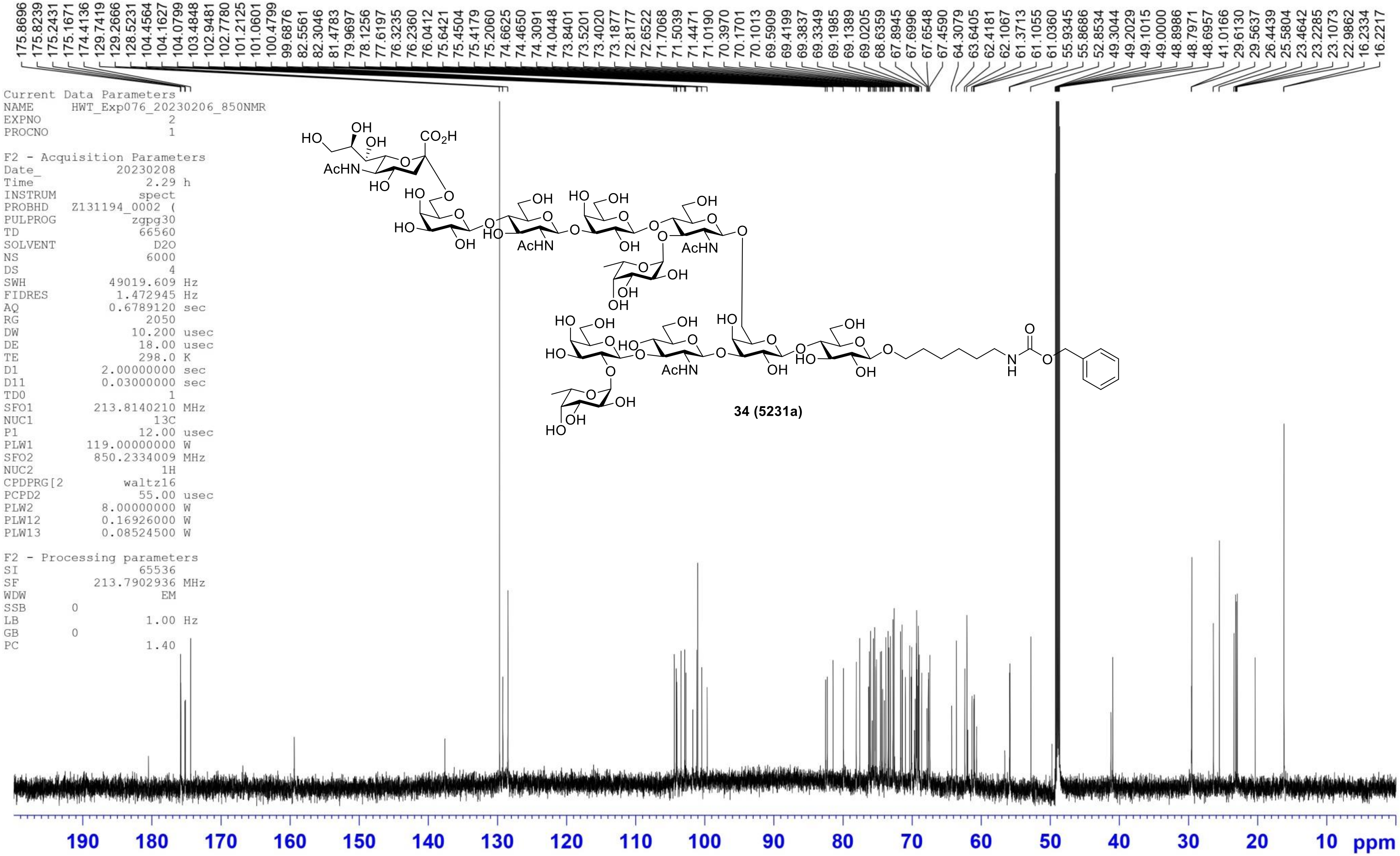

34 (5231a)

^{13}C NMR spectrum of **34** (5231a) (214 MHz, D_2O)

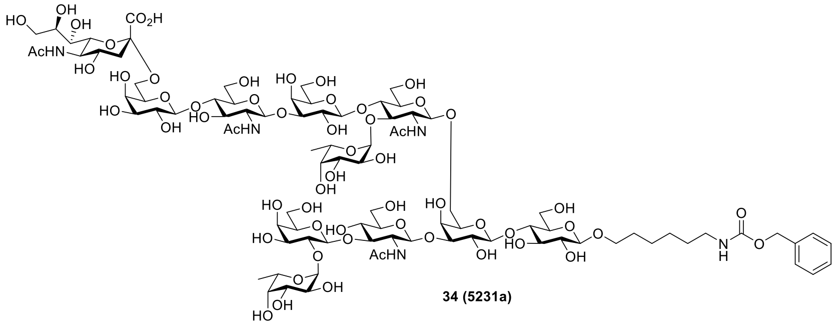

34 (5231a)

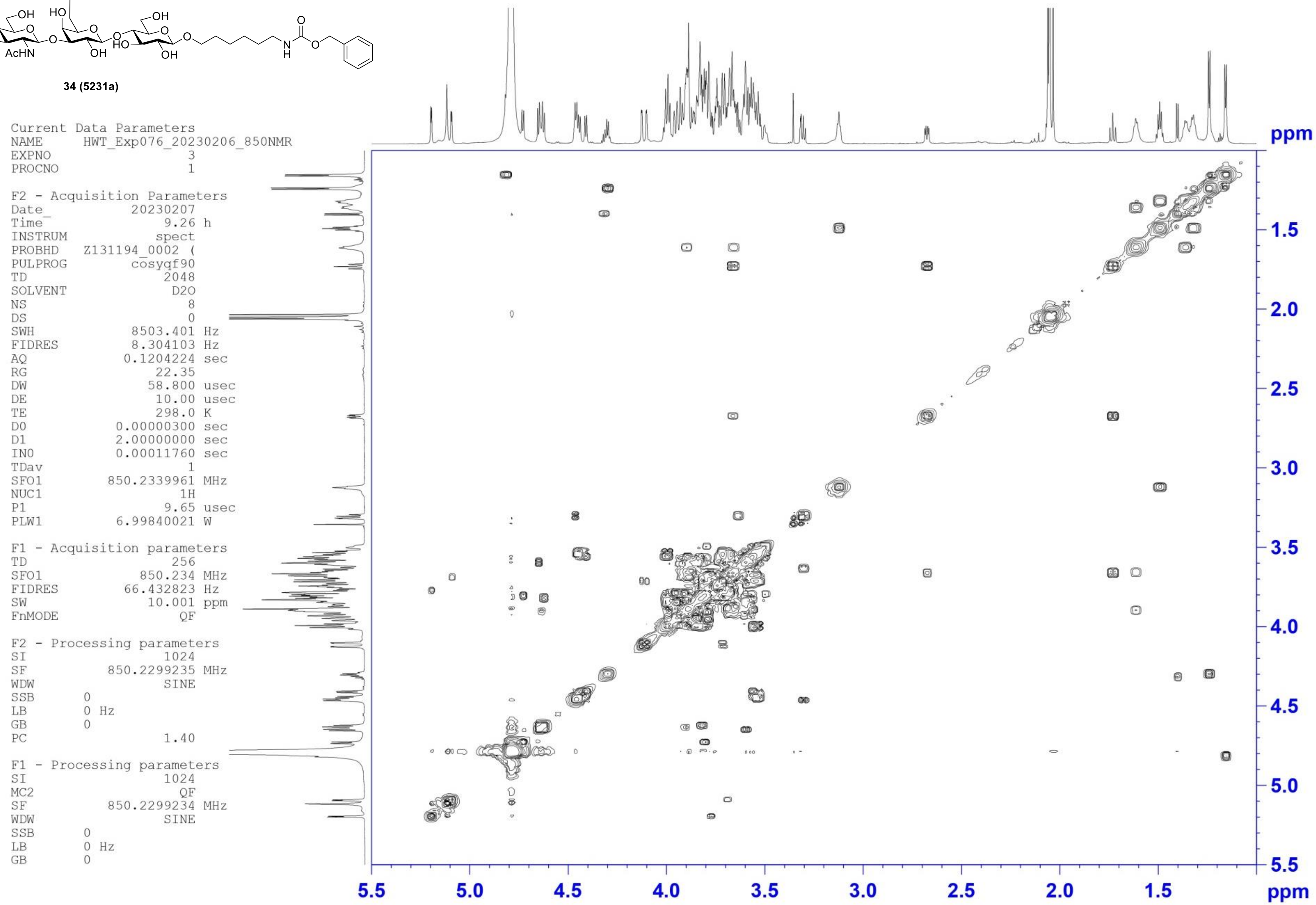

^{1}H-^{1}H COSY NMR spectrum of **34** (5231a) (850 MHz, D_2O)

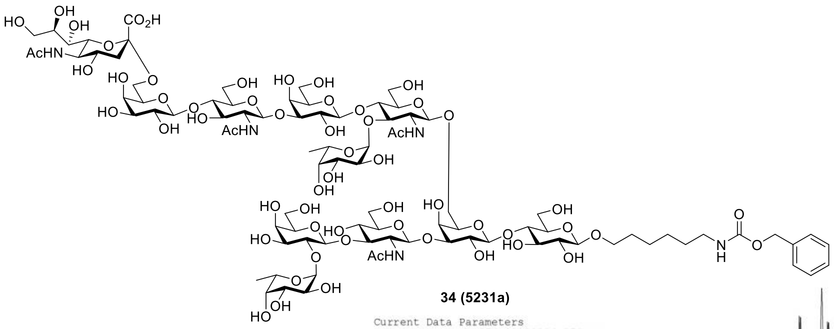

34 (5231a)

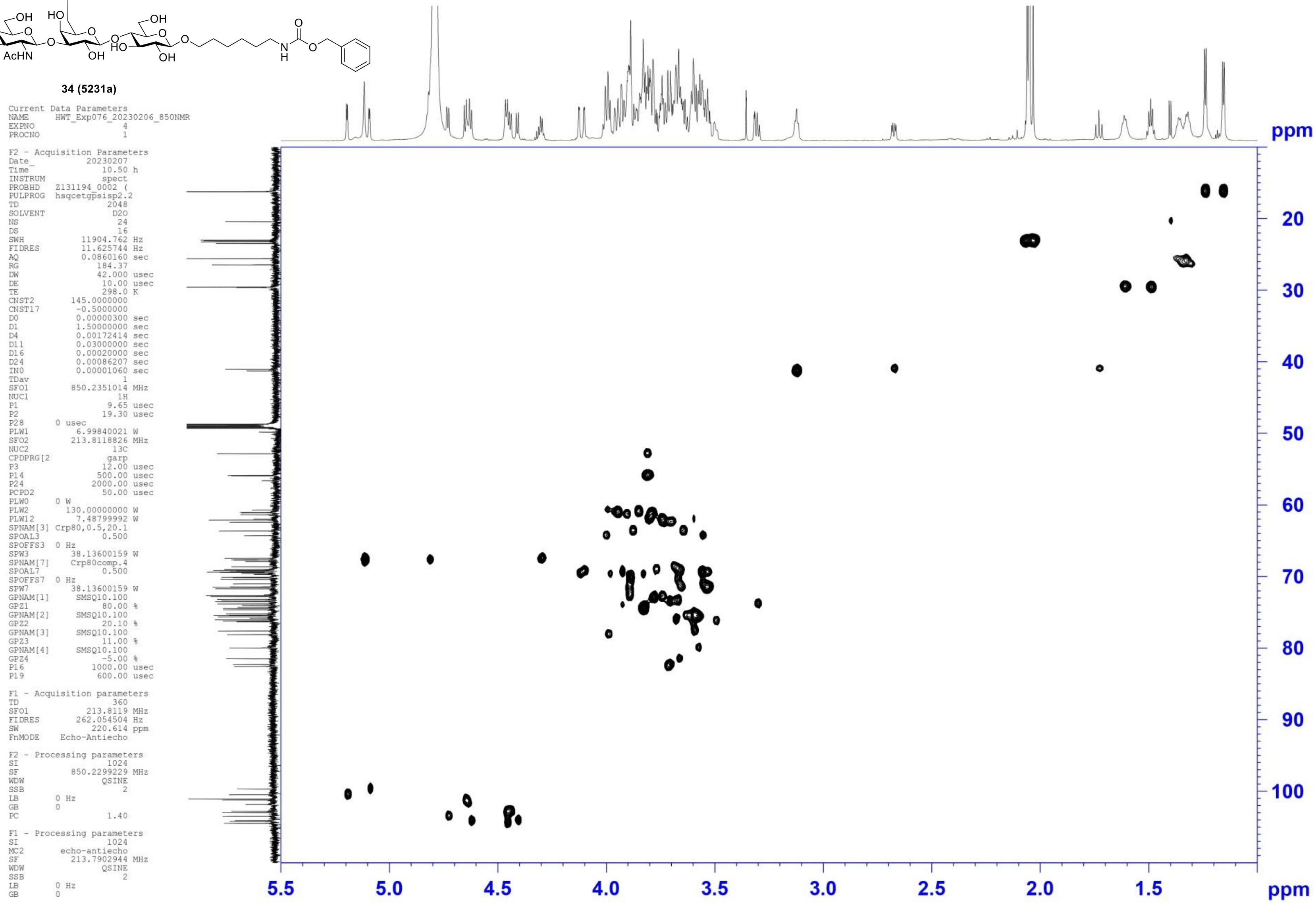

^{1}H-^{13}C HSQC NMR spectrum of **34** (5231a) (850 MHz/214 MHz, D_2O)

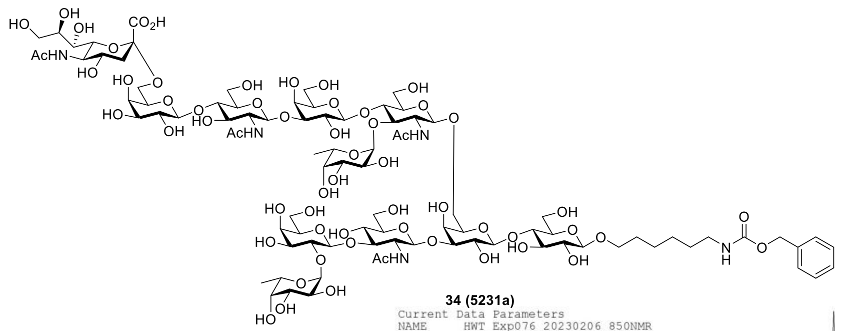

34 (5231a)

Current Data Parameters
NAME HWT_Exp076_20230206_850NMR
EXPNO 5
PROCNO 1

F2 - Acquisition Parameters
Date_ 20230208
Time 2.30 h
INSTRUM spect
PROBHD Z131194_0002 (
PULPROG clhmbcetgpl3nd
TD 2048
SOLVENT D2O
NS 32
DS 16
SWH 8503.401 Hz
FIDRES 8.304103 Hz
AQ 0.1204224 sec
RG 184.37
DW 58.800 usec
DE 10.00 usec
TE 298.0 K
CNST6 125.0000000
CNST7 165.0000000
CNST13 8.0000000
D0 0.00000300 sec
D1 1.50000000 sec
D6 0.06250000 sec
D16 0.00020000 sec
D21 0 sec
IN0 0.00001170 sec
L0 0
TDav 1
SFO1 850.2339961 MHz
NUC1 1H
P1 9.65 usec
P2 19.30 usec
PLW1 6.99840021 W
SFO2 213.8118831 MHz
NUC2 13C
P3 12.00 usec
P14 500.00 usec
P24 2000.00 usec
PLW2 130.00000000 W
SPNAM[3] Crp80,0.5,20.1
SPOAL3 0.500
SPOFFS3 0 Hz
SPW3 38.13600159 W
SPNAM[7] Crp80comp.4
SPOAL7 0.500
SPOFFS7 0 Hz
SPW7 38.13600159 W
GPNAM[1] SMSQ10.100
GPZ1 80.00 %
GPNAM[3] SMSQ10.100
GPZ3 14.00 %
P16 1000.00 usec

F1 - Acquisition parameters
TD 360
SFO1 213.8119 MHz
FIDRES 237.416901 Hz
SW 199.872 ppm
FnMODE Echo-Antiecho

F2 - Processing parameters
SI 1024
SF 850.2299238 MHz
WDW QSINE
SSB 2
LB 0 Hz
GB 0
PC 1.40

F1 - Processing parameters
SI 1024
MC2 echo-antiecho
SF 213.7902984 MHz
WDW QSINE
SSB 2
LB 0 Hz
GB 0

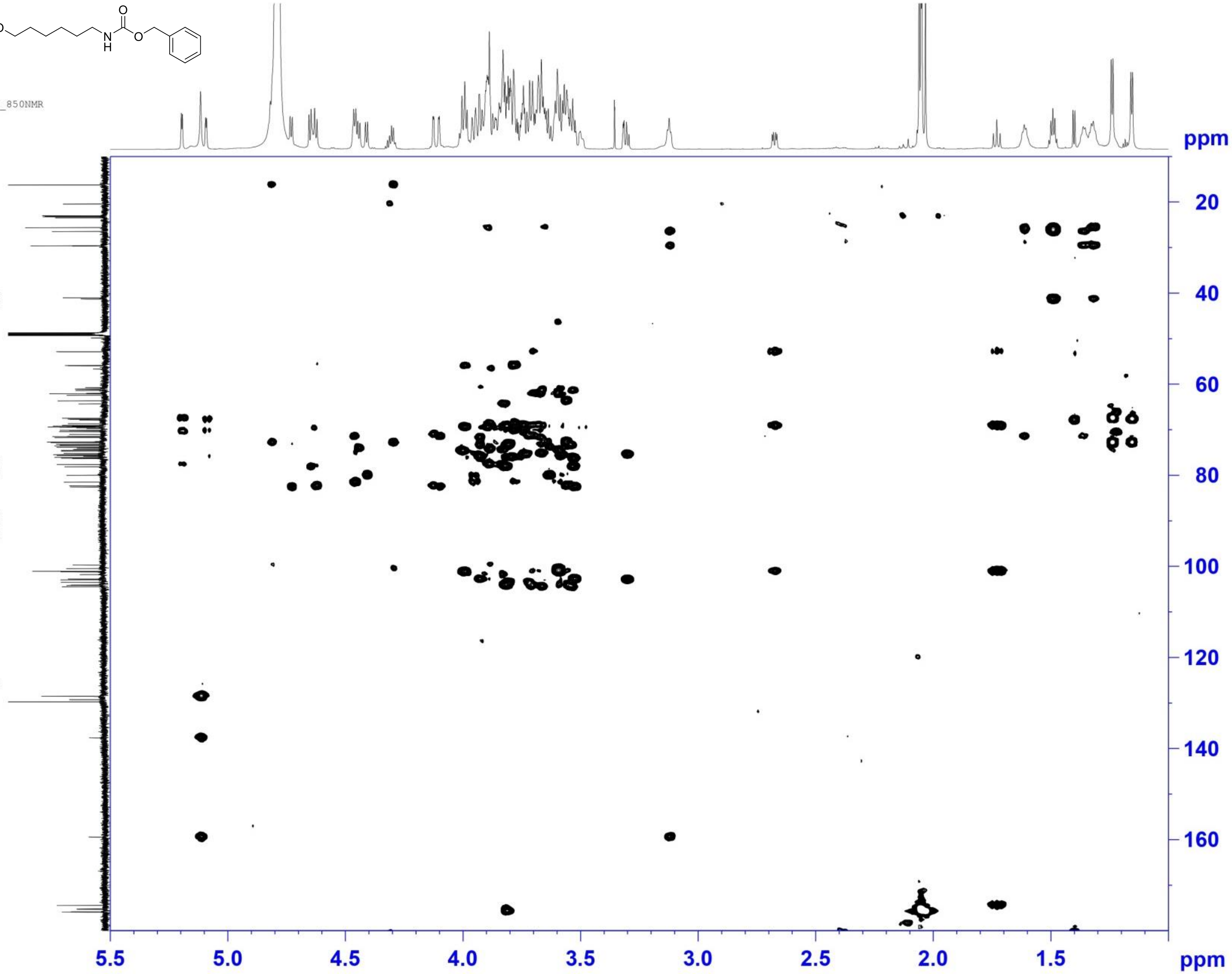

^{1}H-^{13}C HMBC NMR spectrum of **34** (5231a) (850 MHz/214 MHz, D_2O)

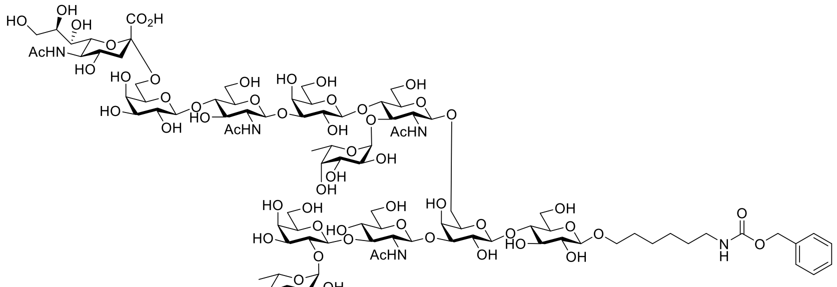

34 (5231a)

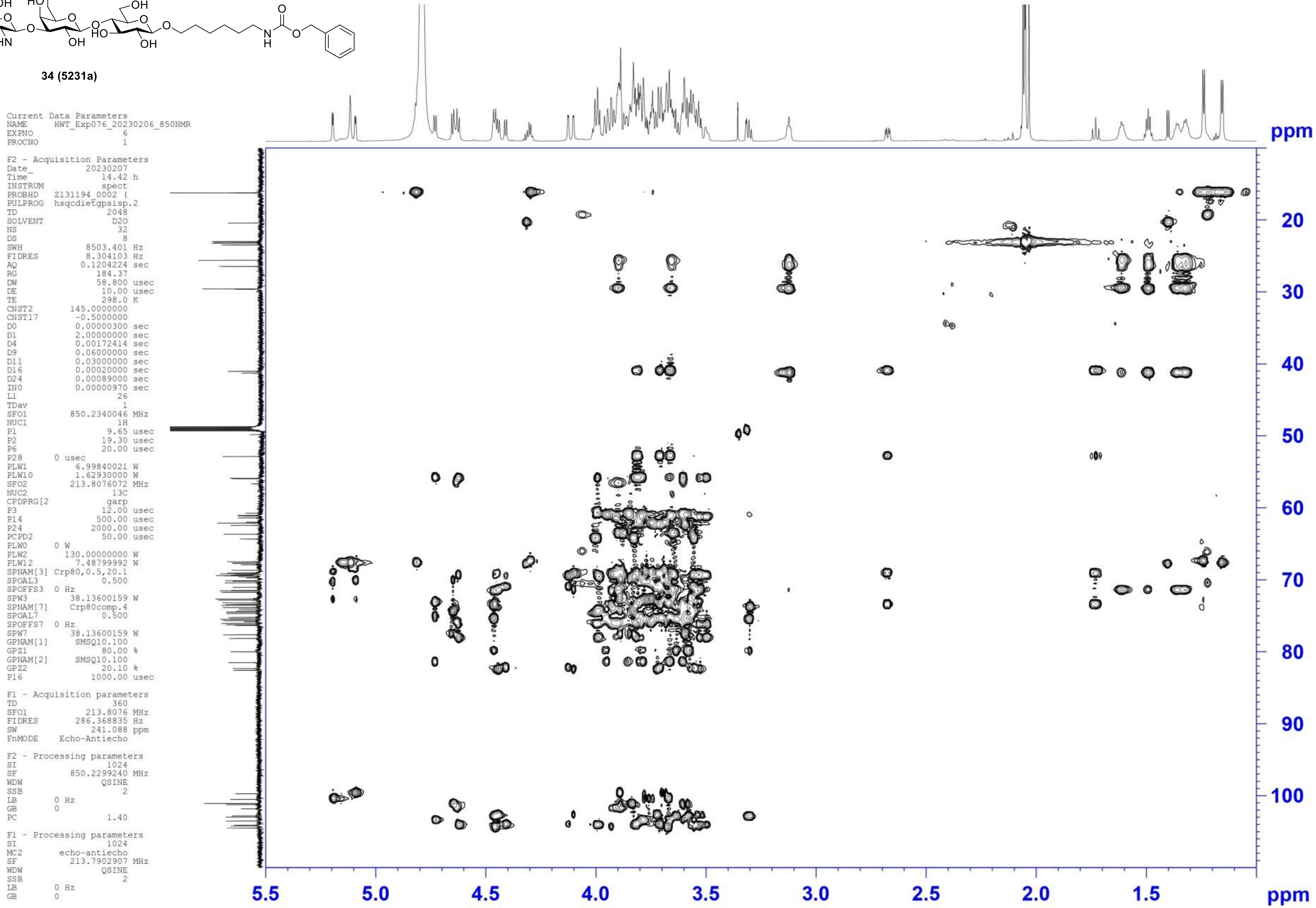

^{1}H-^{13}C HSQC-TOSCY NMR spectrum of **34** (5231a) (850 MHz/214 MHz, D_2O)

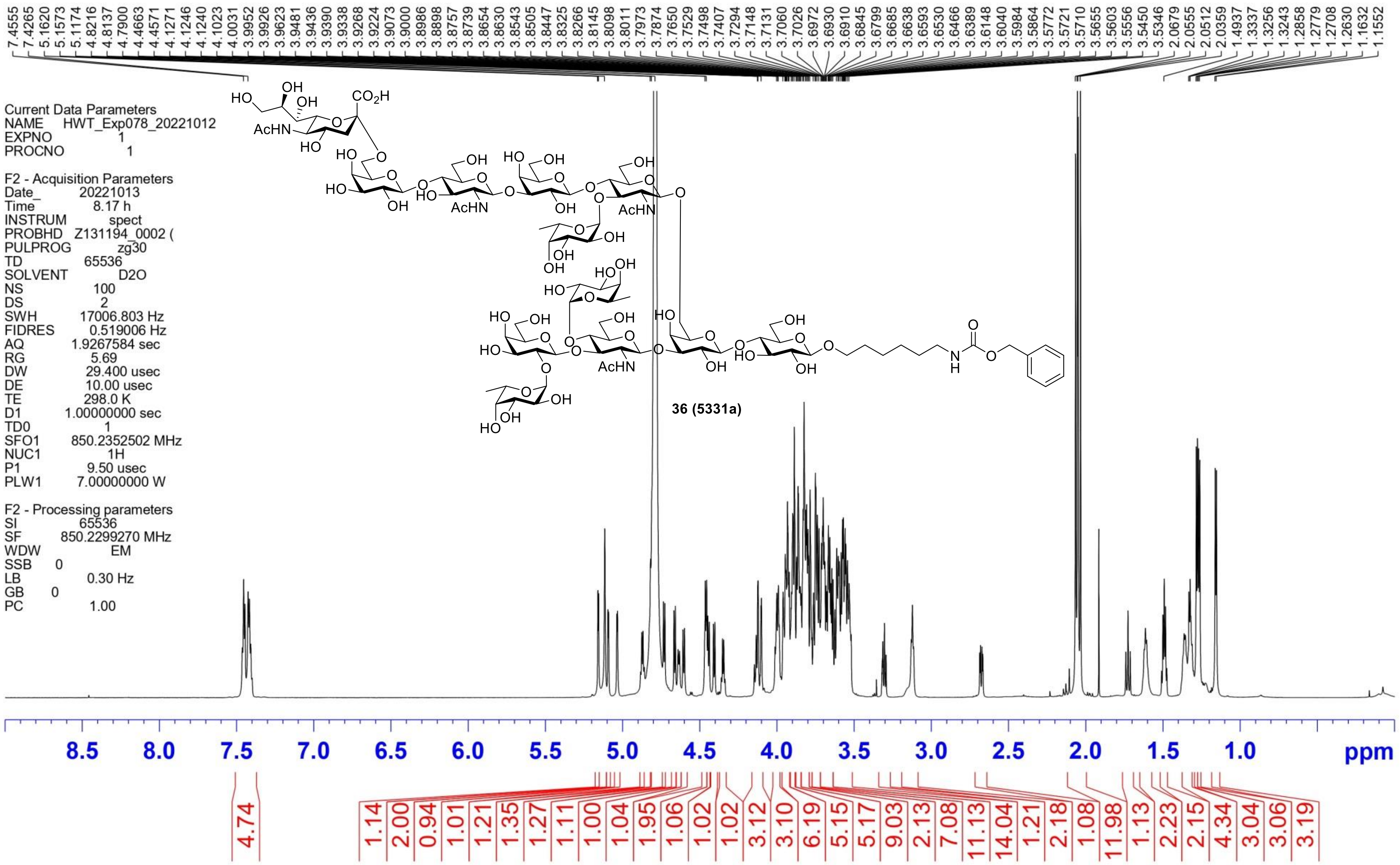

^{1}H NMR spectrum of **36** (5331a) (850 MHz, D_2O)

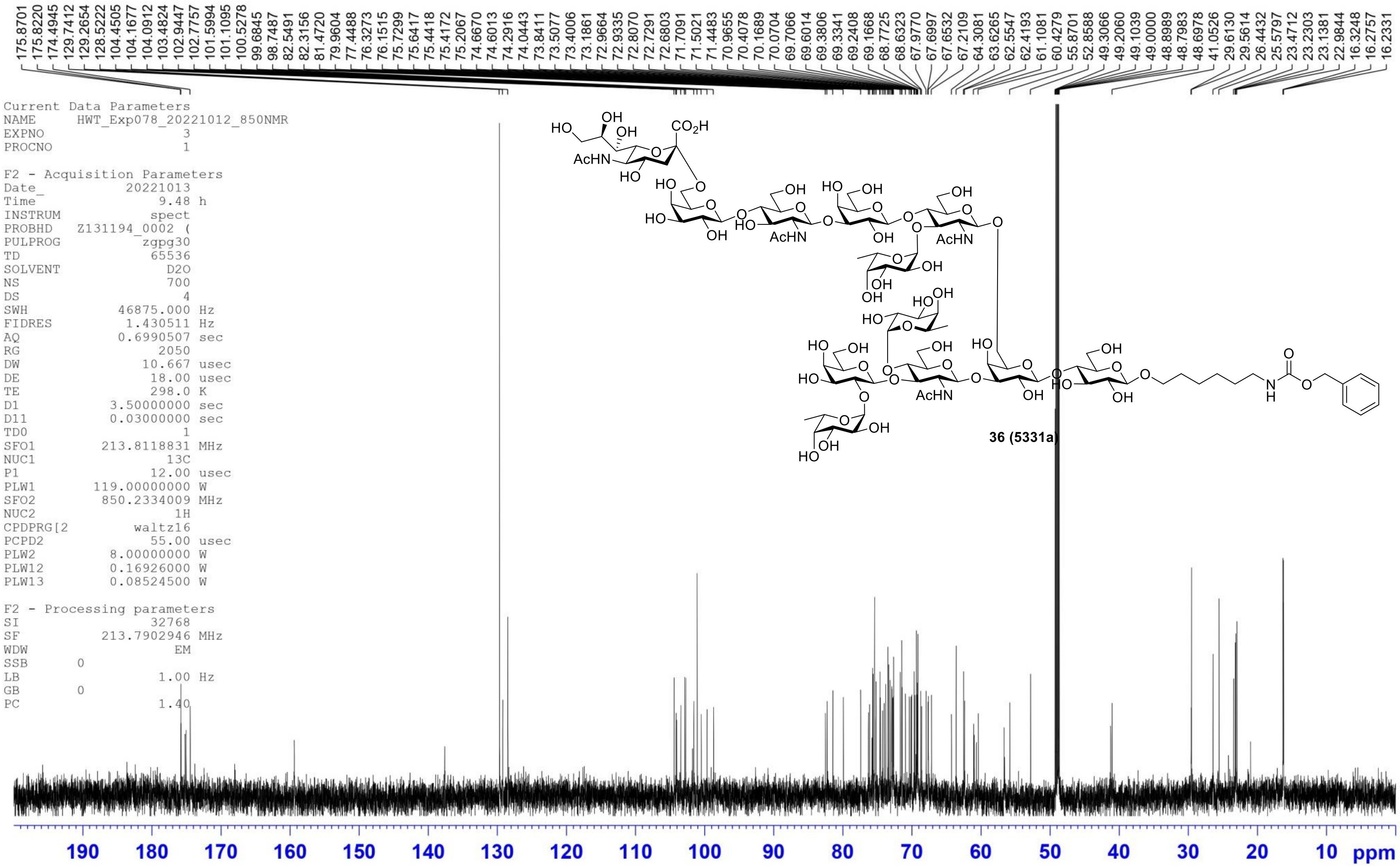

^{13}C NMR spectrum of **36** (5331a) (214 MHz, D_2O)

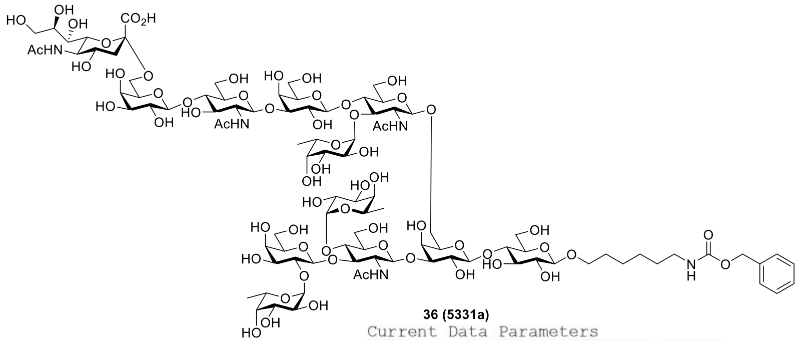

36 (5331a)

```
Current Data Parameters
NAME      HWT_Exp078_20221012_850NMR
EXPNO                      4
PROCNO                     1

F2 - Acquisition Parameters
Date_             20221013
Time                6.53 h
INSTRUM            spect
PROBHD    Z131194_0002 (
PULPROG          cosyqf90
TD                  2048
SOLVENT              D2O
NS                     8
DS                     0
SWH             8503.401 Hz
FIDRES          8.304103 Hz
AQ             0.1204224 sec
RG                 16.45
DW                58.800 usec
DE                 10.00 usec
TE                 298.0 K
D0            0.00000300 sec
D1            1.50000000 sec
IN0           0.00011760 sec
TDav                   1
SFO1         850.2339961 MHz
NUC1                  1H
P1                  9.50 usec
PLW1          6.99840021 W

F1 - Acquisition parameters
TD                   360
SFO1             850.234 MHz
FIDRES         47.241119 Hz
SW                10.001 ppm
FnMODE                QF

F2 - Processing parameters
SI                  1024
SF           850.2299278 MHz
WDW                 SINE
SSB       0
LB        0 Hz
GB        0
PC                  1.40

F1 - Processing parameters
SI                  1024
MC2                   QF
SF           850.2299277 MHz
WDW                 SINE
SSB       0
LB        0 Hz
GB        0
```

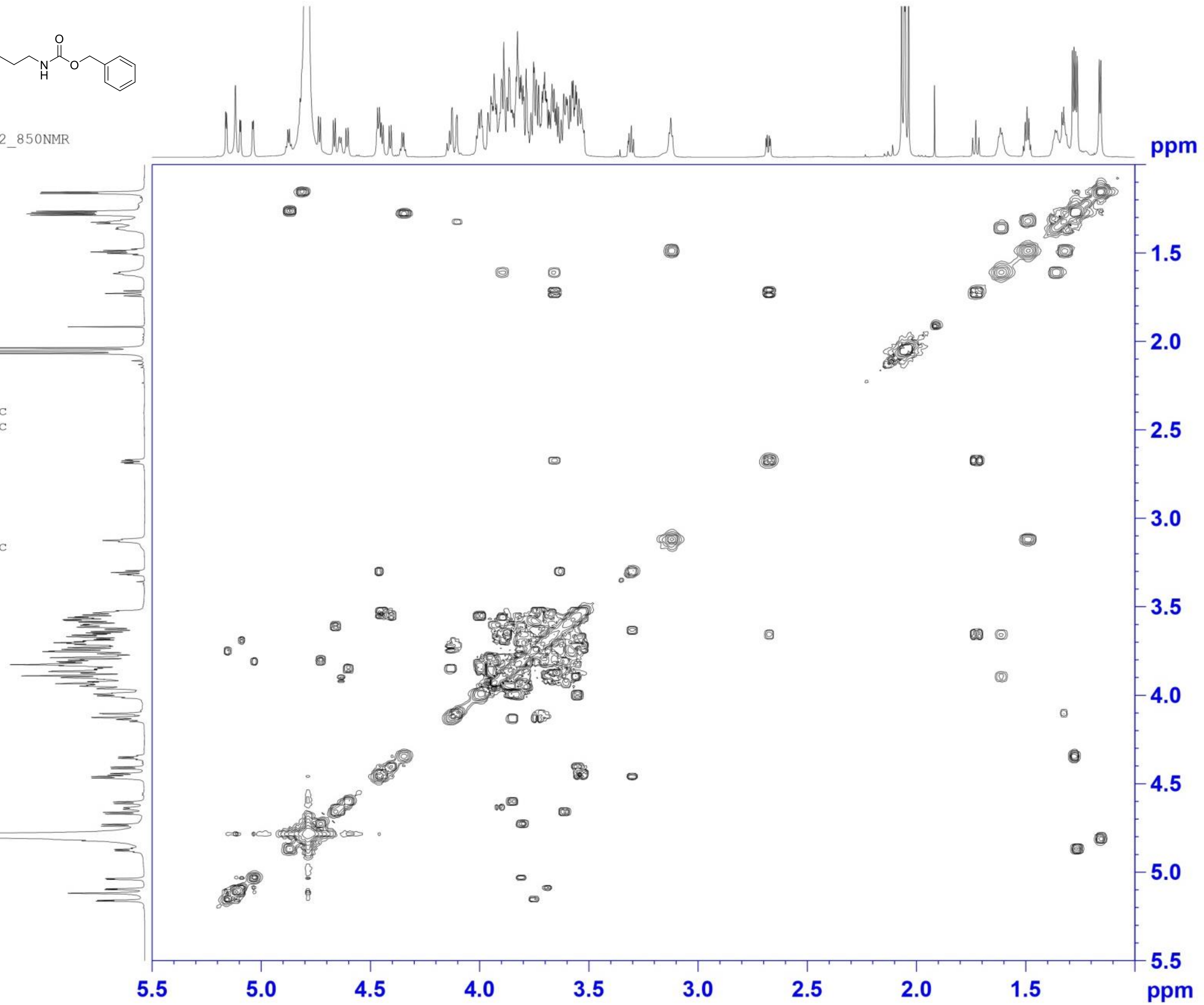

^{1}H-^{1}H COSY NMR spectrum of **36** (5331a) (850 MHz, D_2O)

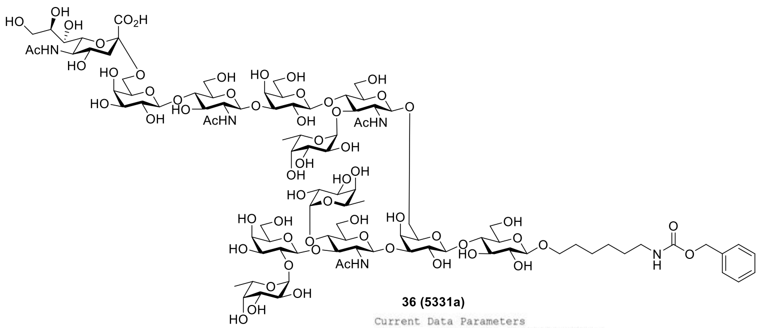


```
Current Data Parameters
NAME     HWT_Exp078_20221012_850NMR
EXPNO                   5
PROCNO                  1

F2 - Acquisition Parameters
Date_            20221013
Time                 4.16 h
INSTRUM             spect
PROBHD   Z131194_0002 (
PULPROG  hsqcetgpsisp2.2
TD                   2048
SOLVENT               D2O
NS                     16
DS                      8
SWH              8503.401 Hz
FIDRES           8.304103 Hz
AQ              0.1204224 sec
RG                 184.37
DW                 58.800 usec
DE                  10.00 usec
TE                  298.0 K
CNST2         145.0000000
CNST17         -0.5000000
D0             0.00000300 sec
D1             1.50000000 sec
D4             0.00172414 sec
D11            0.03000000 sec
D16            0.00020000 sec
D24            0.00086207 sec
IN0            0.00001060 sec
TDav                    1
SFO1          850.2340054 MHz
NUC1                   1H
P1                   9.50 usec
P2                  19.00 usec
P28      0 usec
PLW1           6.99840021 W
SFO2          213.8118831 MHz
NUC2                  13C
CPDPRG[2             garp
P3                  12.00 usec
P14                500.00 usec
P24               2000.00 usec
PCPD2               50.00 usec
PLW0     0 W
PLW2         130.00000000 W
PLW12          7.48799992 W
SPNAM[3] Crp80,0.5,20.1
SPOAL3              0.500
SPOFFS3  0 Hz
SPW3          38.13600159 W
SPNAM[7]     Crp80comp.4
SPOAL7              0.500
SPOFFS7  0 Hz
SPW7          38.13600159 W
GPNAM[1]      SMSQ10.100
GPZ1                80.00 %
GPNAM[2]      SMSQ10.100
GPZ2                20.10 %
GPNAM[3]      SMSQ10.100
GPZ3                11.00 %
GPNAM[4]      SMSQ10.100
GPZ4                -5.00 %
P16               1000.00 usec
P19                600.00 usec

F1 - Acquisition parameters
TD                    360
SFO1             213.8119 MHz
FIDRES         262.054504 Hz
SW                220.614 ppm
FnMODE     Echo-Antiecho

F2 - Processing parameters
SI                   1024
SF            850.2299289 MHz
WDW                 QSINE
SSB                     4
LB       0 Hz
GB       0
PC                   1.40

F1 - Processing parameters
SI                   1024
MC2        echo-antiecho
SF            213.7903003 MHz
WDW                 QSINE
SSB                     4
LB       0 Hz
GB       0
```

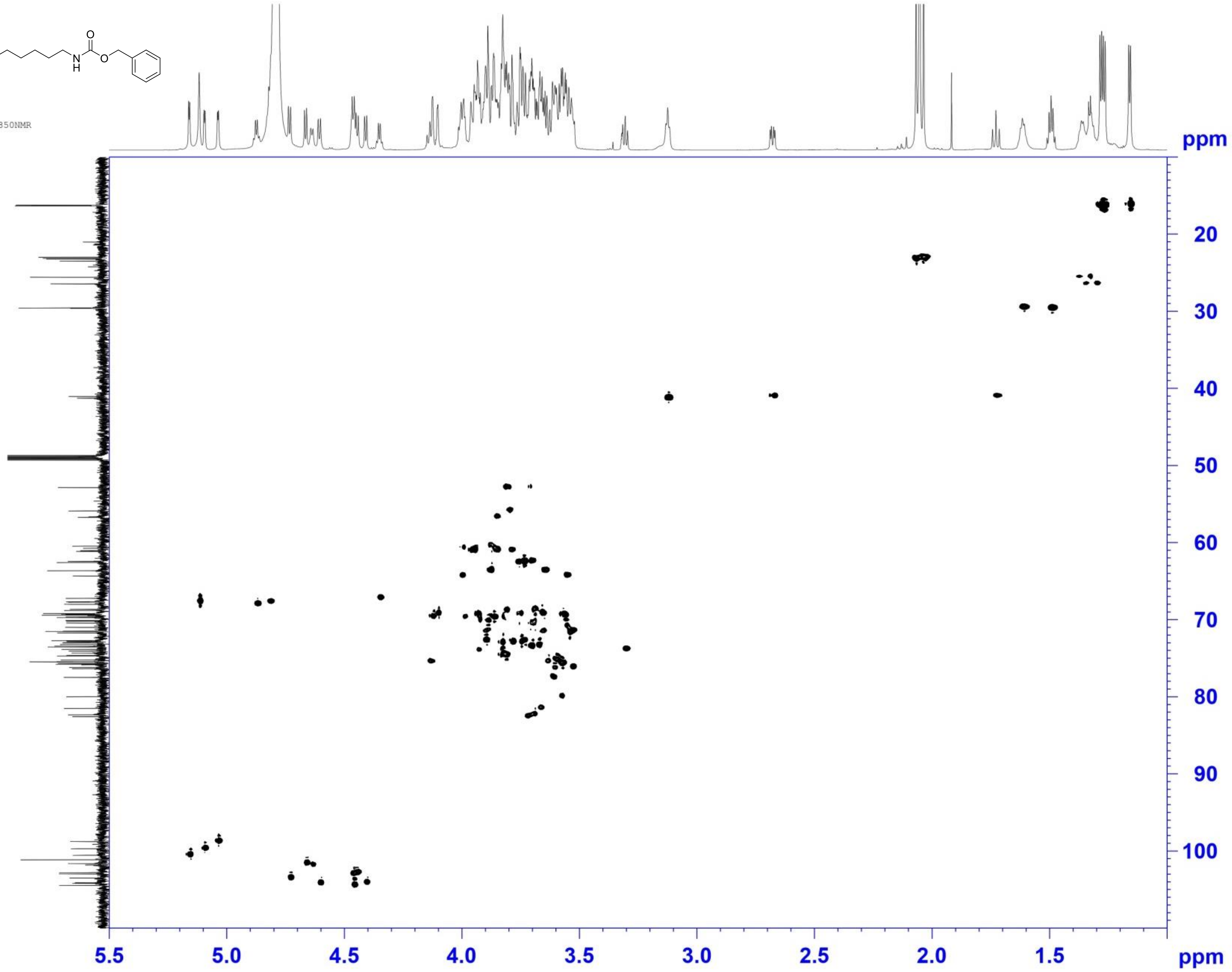

^{1}H-^{13}C HSQC NMR spectrum of **36** (5331a) (850 MHz/214 MHz, D_2O)

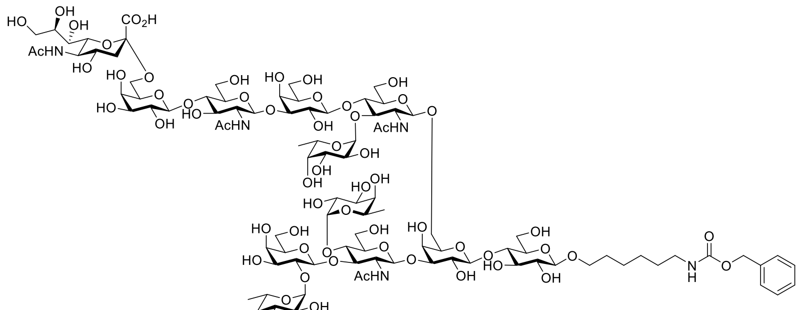

36 (5331a)

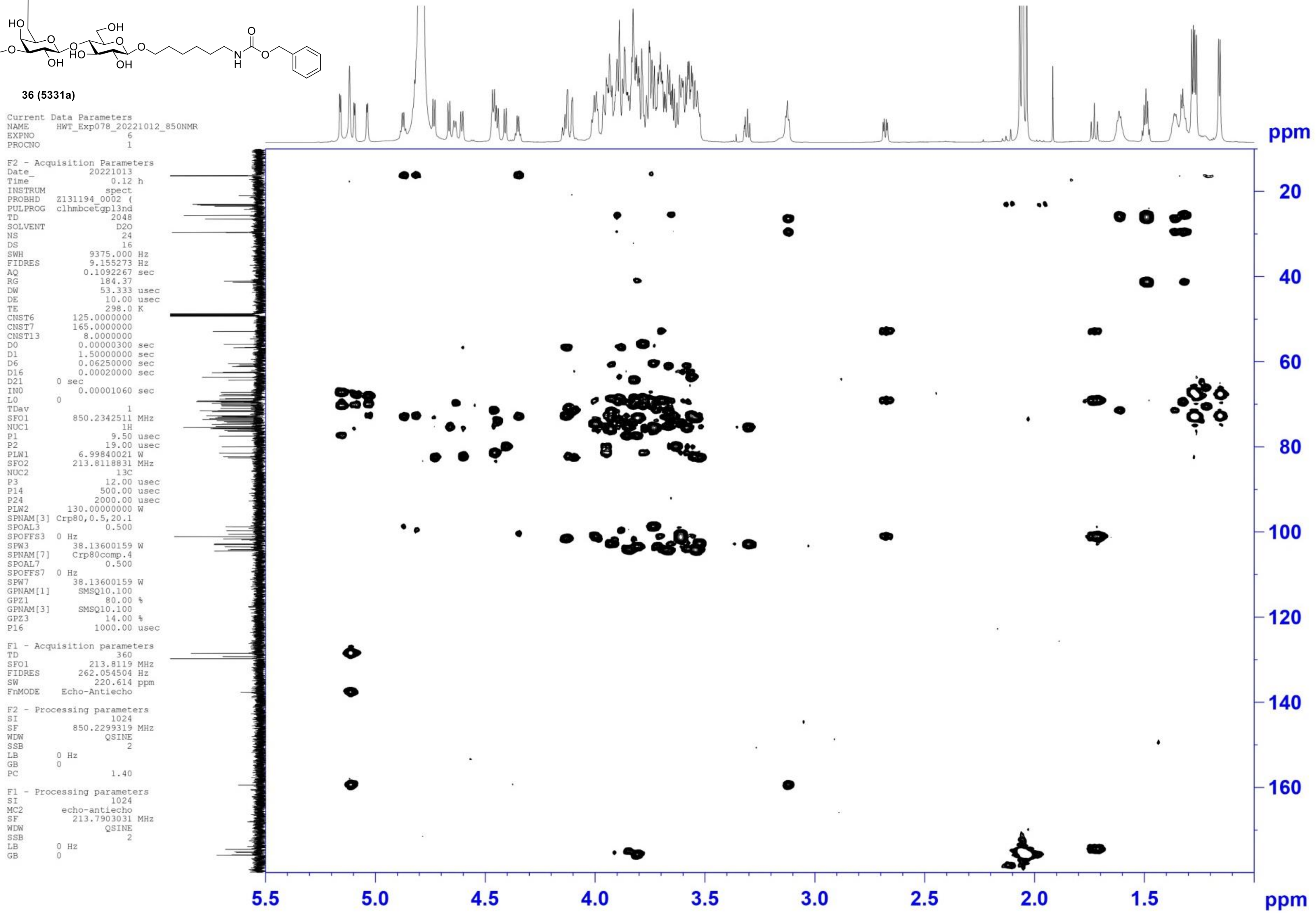

^{1}H-^{13}C HMBC NMR spectrum of **36** (5331a) (850 MHz/214 MHz, D_2O)

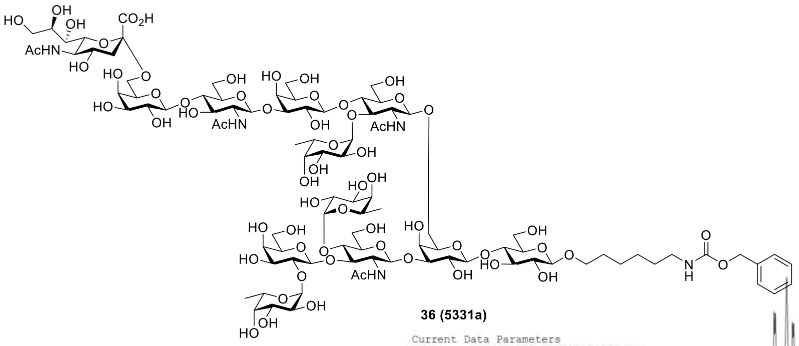

36 (5331a)

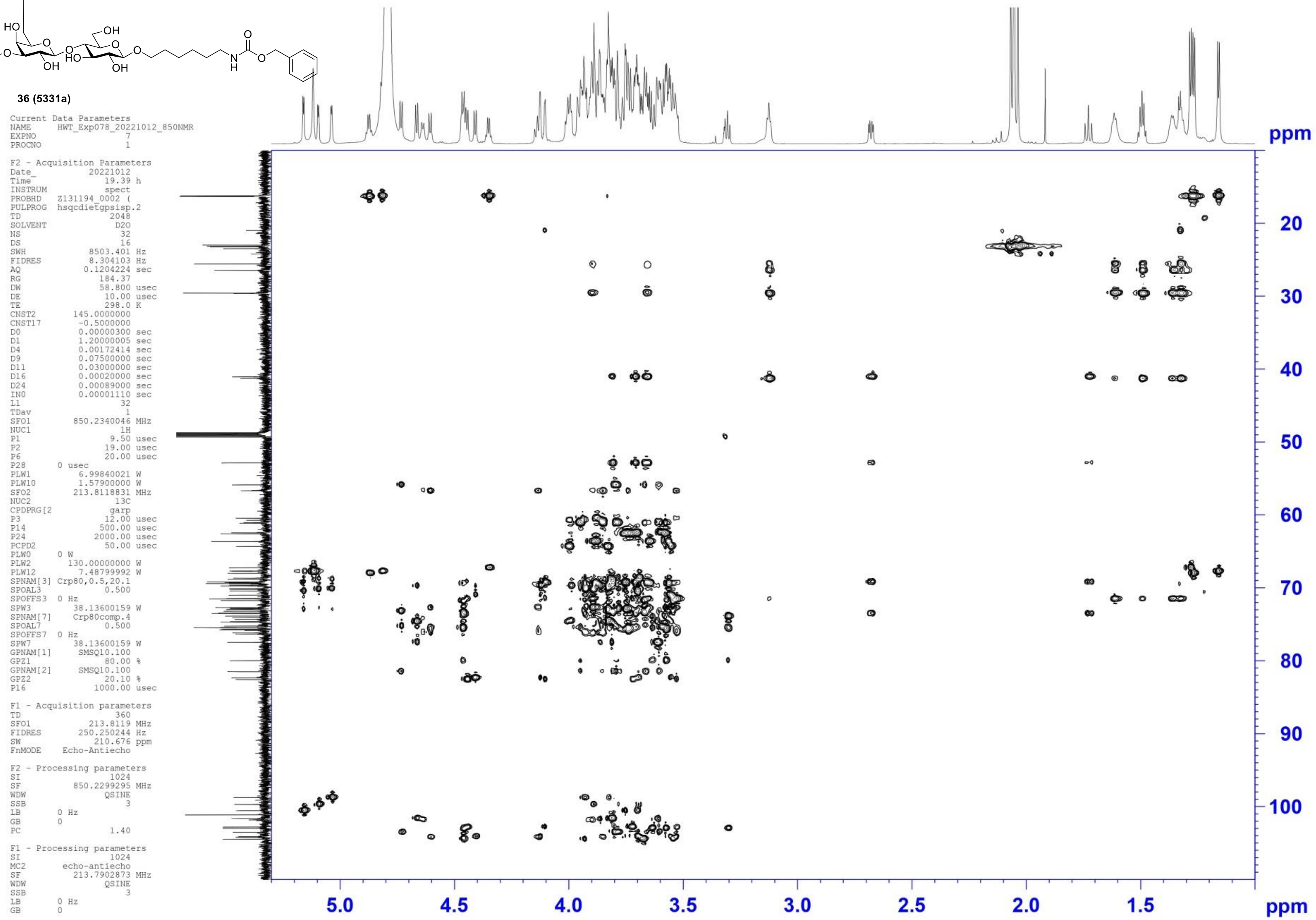

^{1}H-^{13}C HSQC-TOSCY NMR spectrum of **36** (5331a) (850 MHz/214 MHz, D_2O)

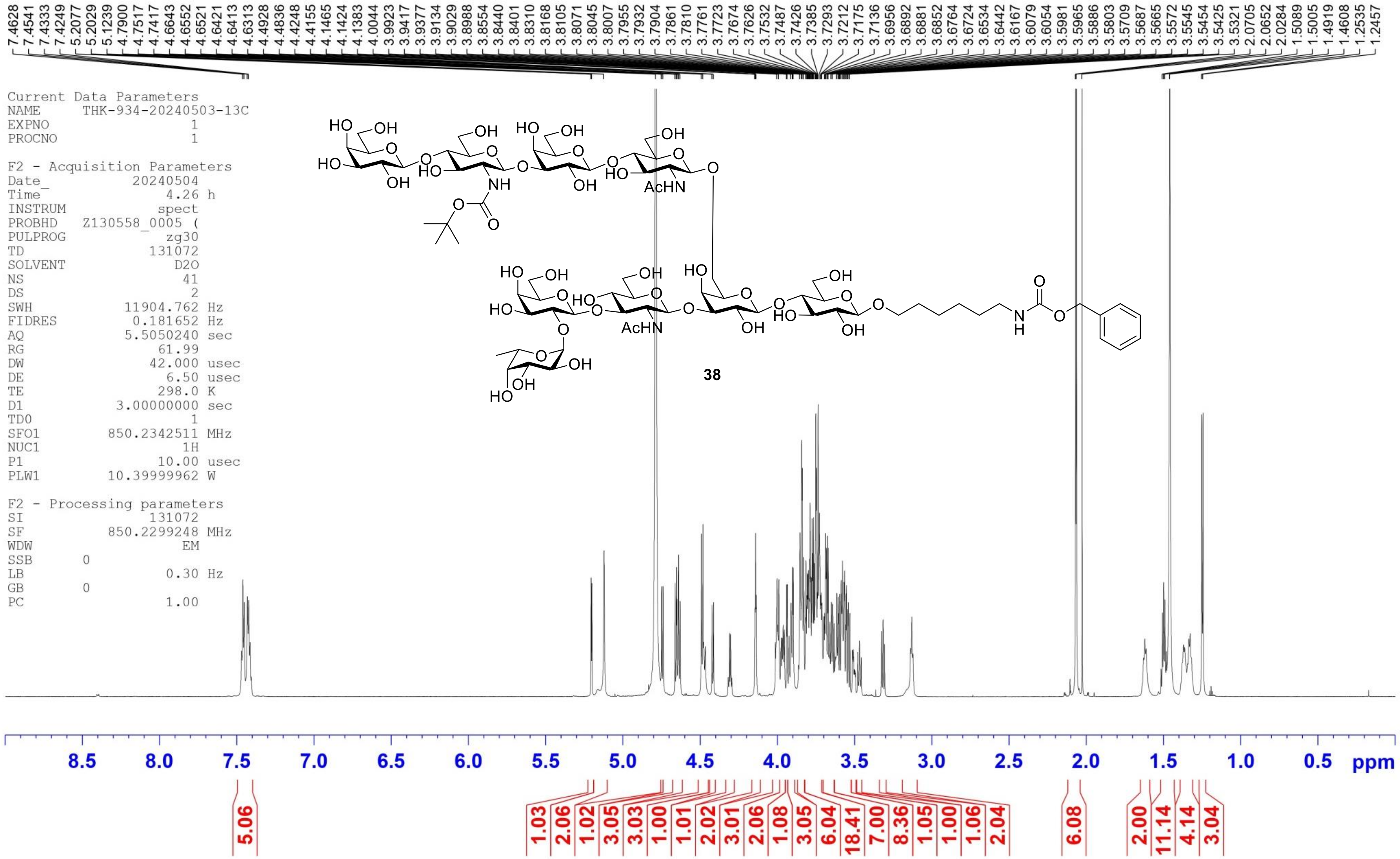

^{1}H NMR spectrum of Compound **38** (850 MHz D_2O)

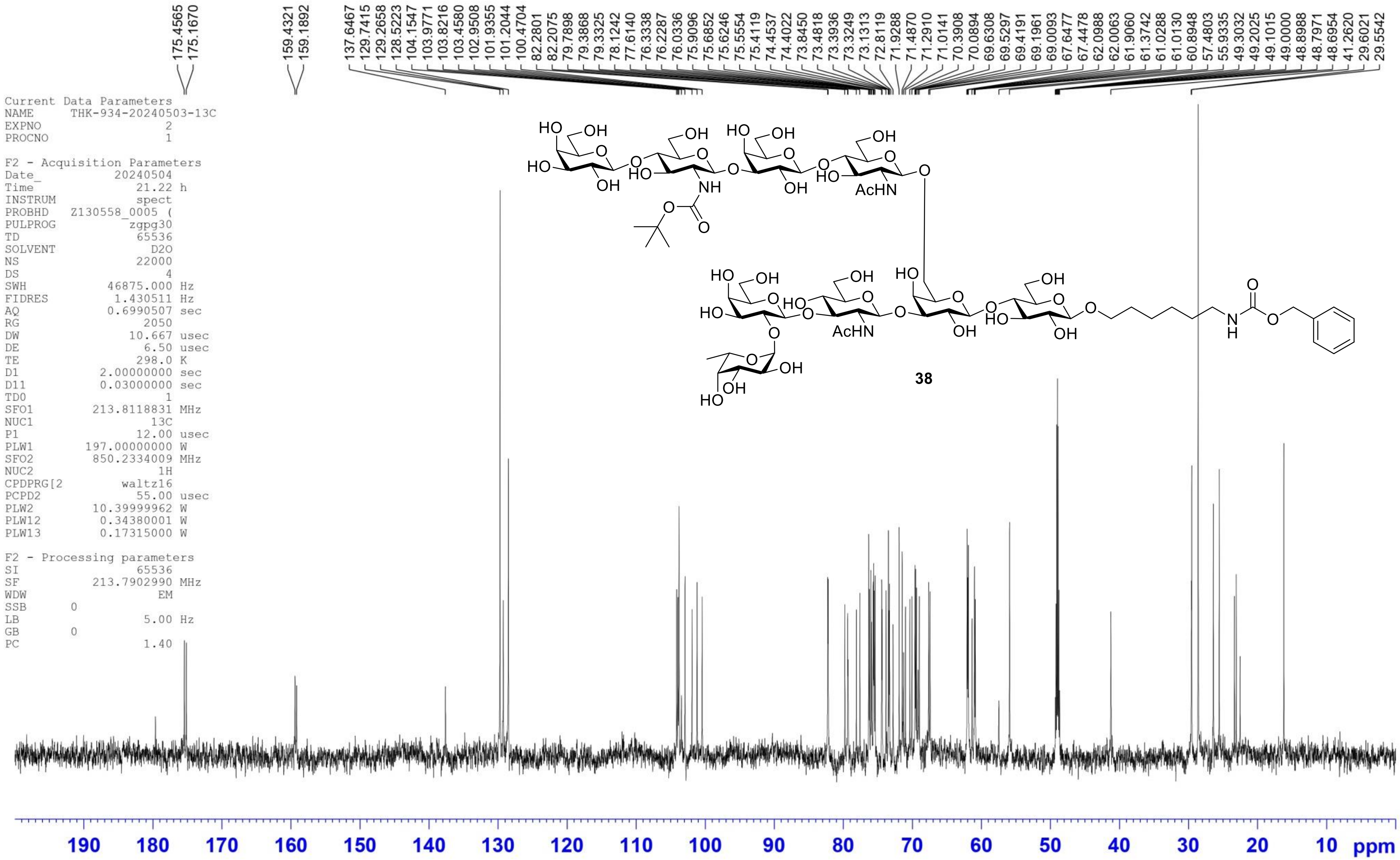

^{13}C NMR spectrum of Compound **38** (214 MHz D_2O)

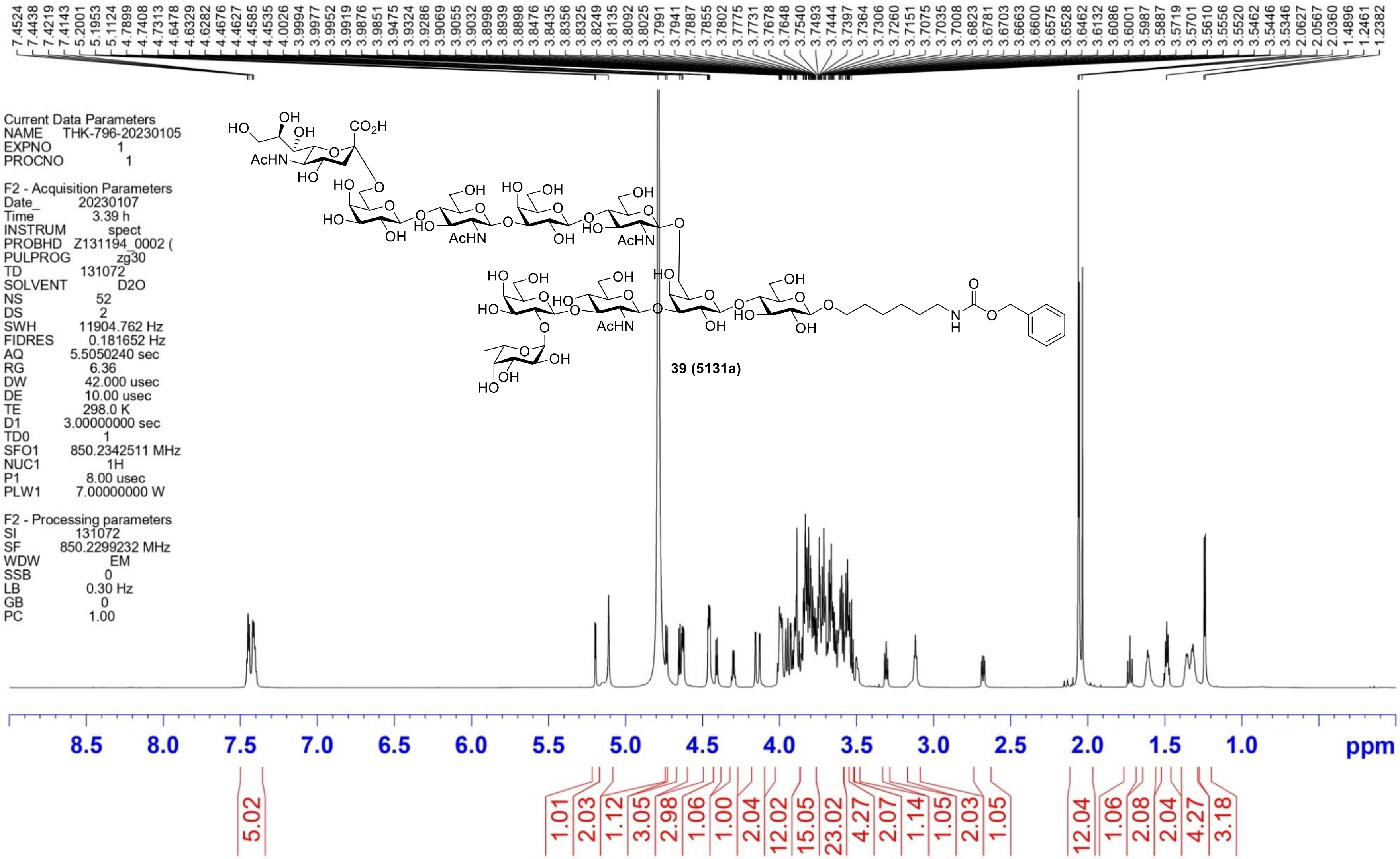

^{1}H NMR spectrum of **39** (5131a) (850 MHz, D_2O)

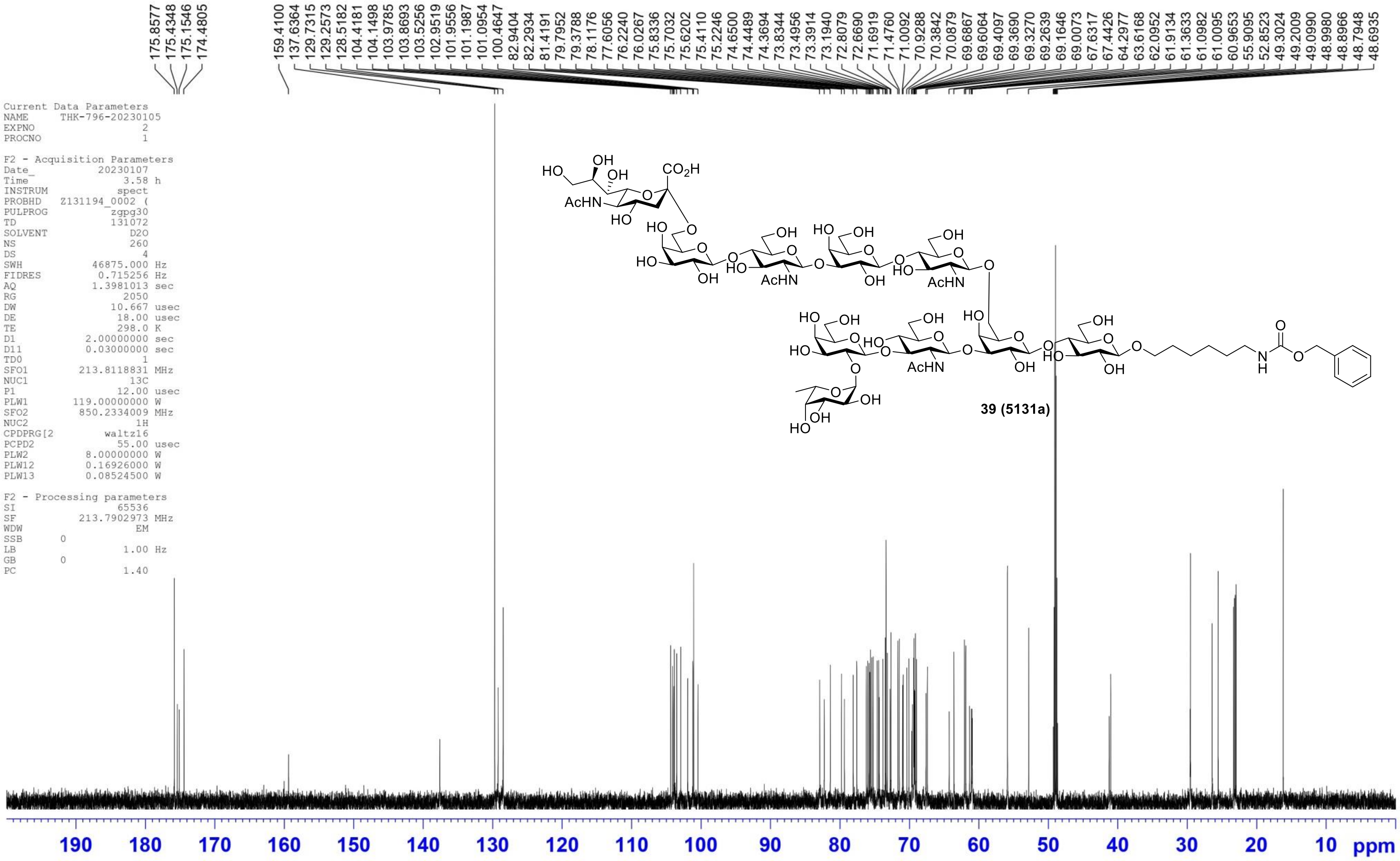

^{13}C NMR spectrum of **39** (5131a) (214 MHz, D_2O)

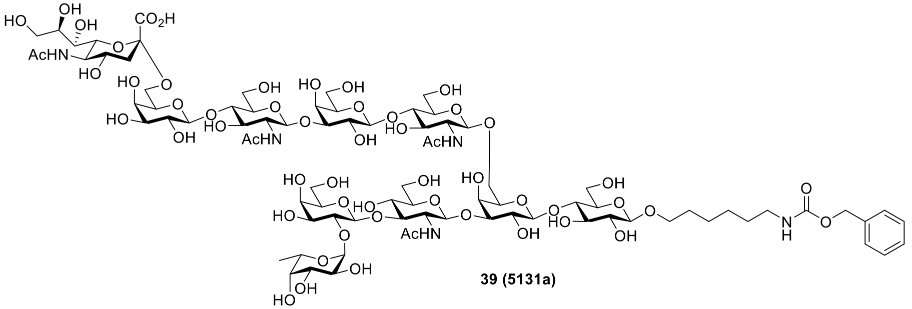

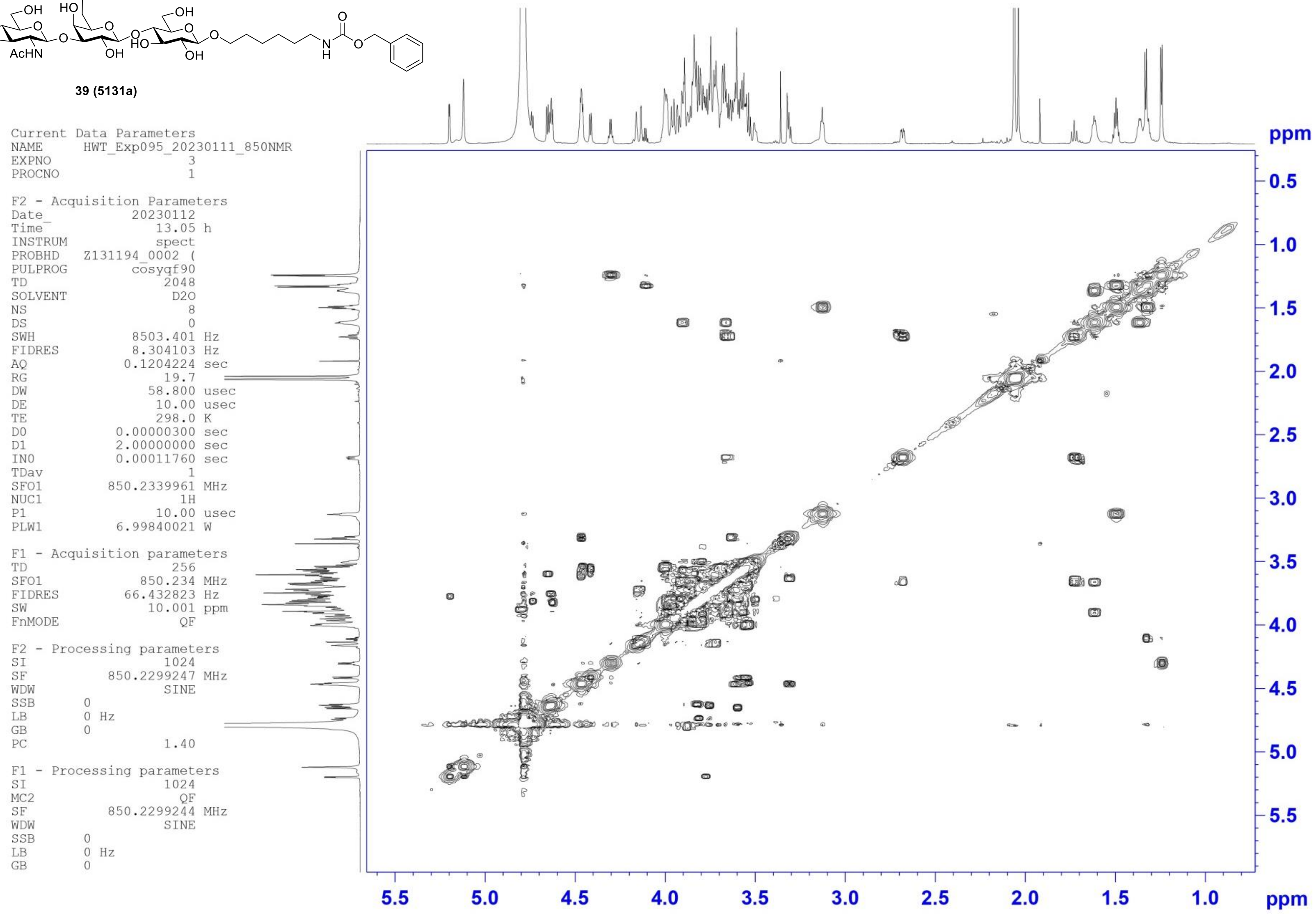

^1H-^1H COSY NMR spectrum of **39** (5131a) (850 MHz, D_2O)

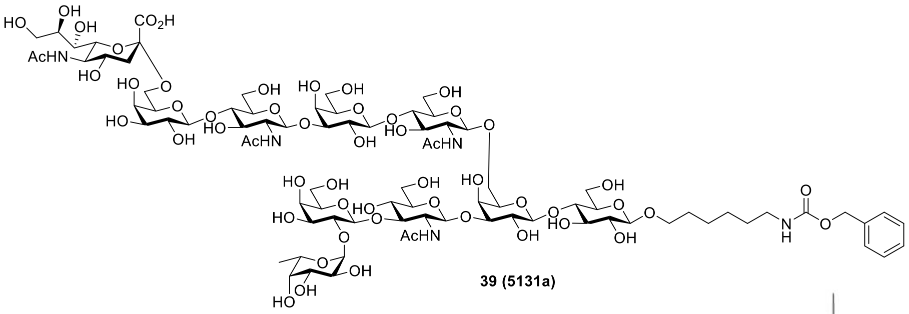

39 (5131a)

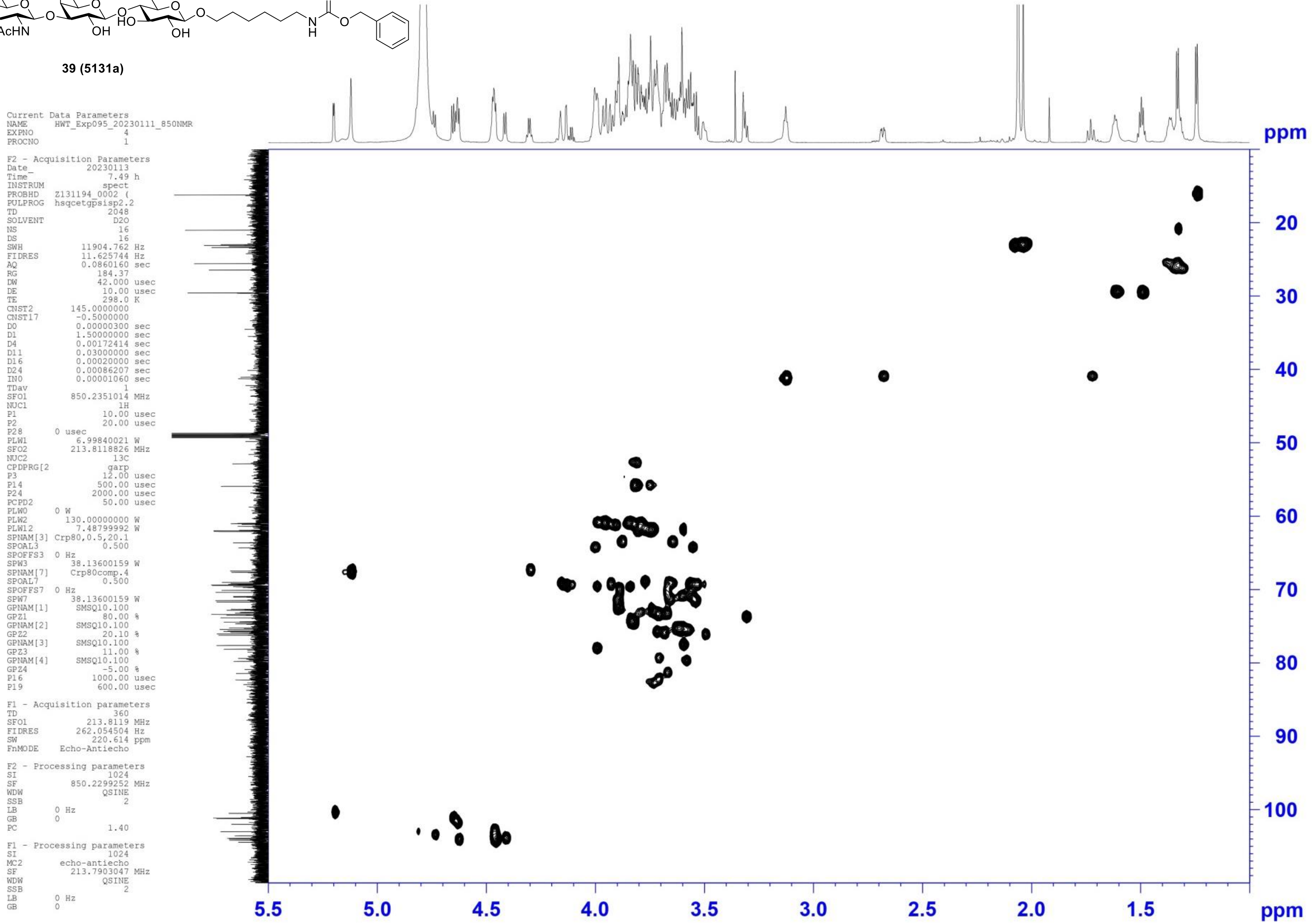

^{1}H-^{13}C HSQC NMR spectrum of **39** (5131a) (850 MHz/214 MHz, D_2O)

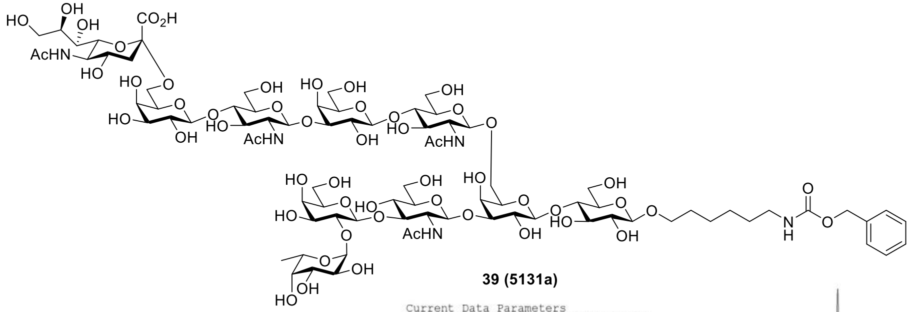

39 (5131a)

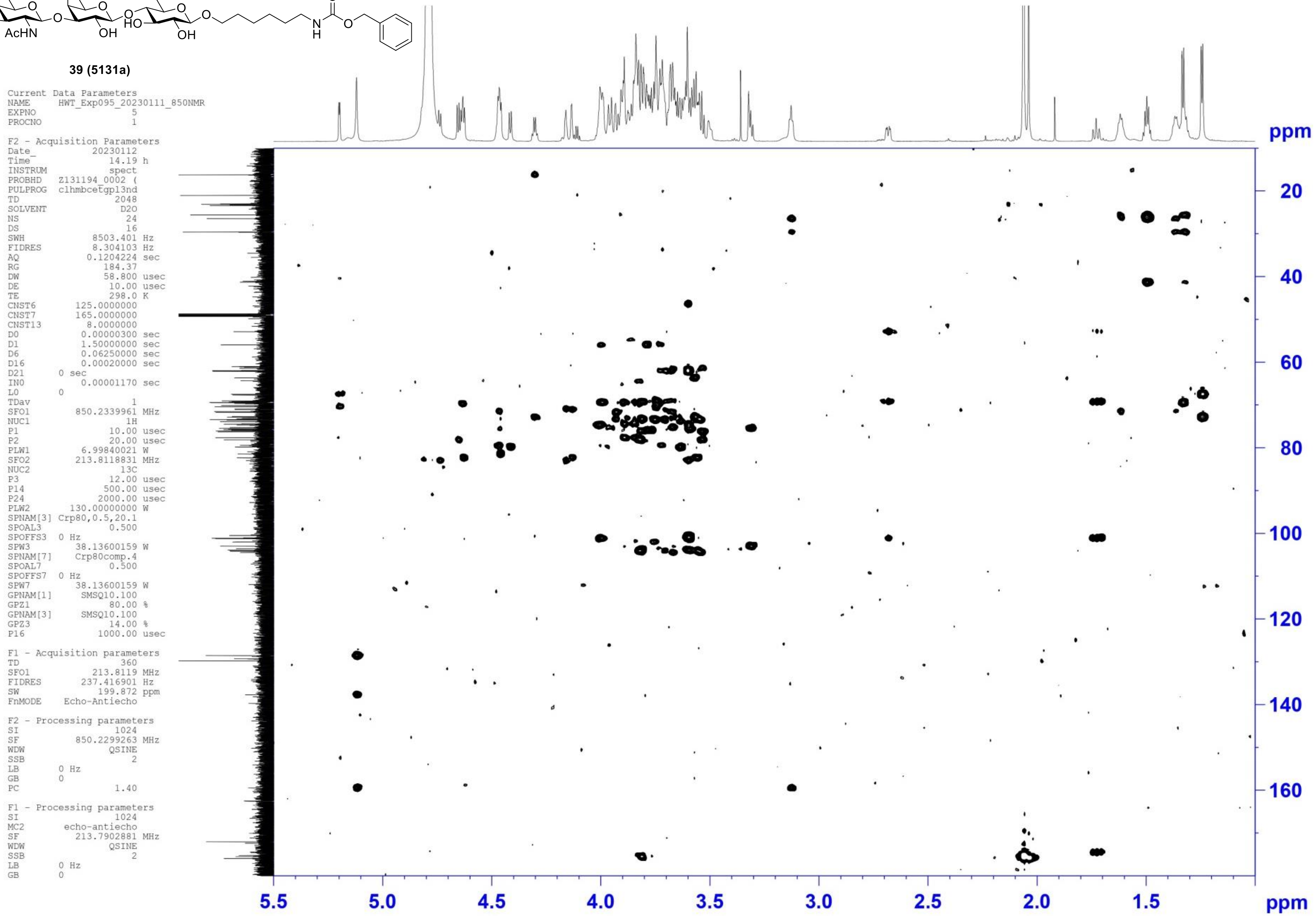

^{1}H-^{13}C HMBC NMR spectrum of **39** (5131a) (850 MHz/214 MHz, D_2O)

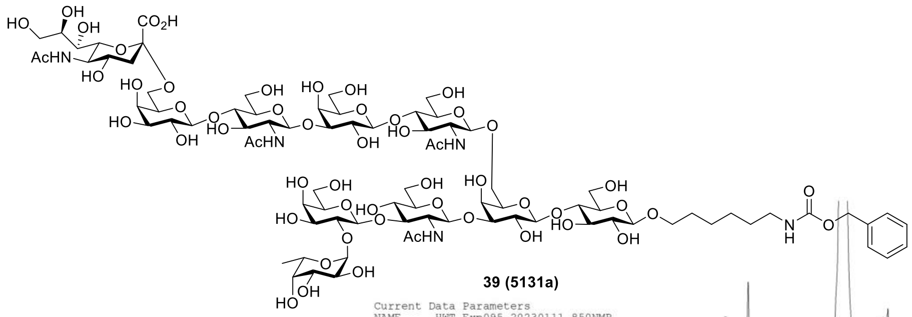

39 (5131a)

```
Current Data Parameters
NAME      HWT_Exp095_20230111_850NMR
EXPNO                     6
PROCNO                    1

F2 - Acquisition Parameters
Date_              20230112
Time                  18.25 h
INSTRUM               spect
PROBHD    Z131194_0002 (
PULPROG   hsqcdietgpsisp.2
TD                     2048
SOLVENT                 D2O
NS                       32
DS                        8
SWH                8503.401 Hz
FIDRES             8.304103 Hz
AQ                0.1204224 sec
RG                   184.37
DW                   58.800 usec
DE                    10.00 usec
TE                    298.0 K
CNST2           145.0000000
CNST17           -0.5000000
D0               0.00000300 sec
D1               2.00000000 sec
D4               0.00172414 sec
D9               0.06000000 sec
D11              0.03000000 sec
D16              0.00020000 sec
D24              0.00089000 sec
IN0              0.00000970 sec
L1                       26
TDav                      1
SFO1            850.2340046 MHz
NUC1                     1H
P1                    10.00 usec
P2                    20.00 usec
P6                    20.00 usec
P28       0 usec
PLW1             6.99840021 W
PLW10            1.74960005 W
SFO2            213.8076072 MHz
NUC2                    13C
CPDPRG[2               garp
P3                    12.00 usec
P14                  500.00 usec
P24                 2000.00 usec
PCPD2                 50.00 usec
PLW0      0 W
PLW2           130.00000000 W
PLW12            7.48799992 W
SPNAM[3] Crp80,0.5,20.1
SPOAL3                0.500
SPOFFS3   0 Hz
SPW3            38.13600159 W
SPNAM[7]     Crp80comp.4
SPOAL7                0.500
SPOFFS7   0 Hz
SPW7            38.13600159 W
GPNAM[1]       SMSQ10.100
GPZ1                  80.00 %
GPNAM[2]       SMSQ10.100
GPZ2                  20.10 %
P16                 1000.00 usec

F1 - Acquisition parameters
TD                      360
SFO1               213.8076 MHz
FIDRES           286.368835 Hz
SW                  241.088 ppm
FnMODE      Echo-Antiecho

F2 - Processing parameters
SI                     1024
SF              850.2299257 MHz
WDW                   QSINE
SSB                       2
LB        0 Hz
GB        0
PC                     1.40

F1 - Processing parameters
SI                     1024
MC2          echo-antiecho
SF              213.7902993 MHz
WDW                   QSINE
SSB                       2
LB        0 Hz
GB        0
```

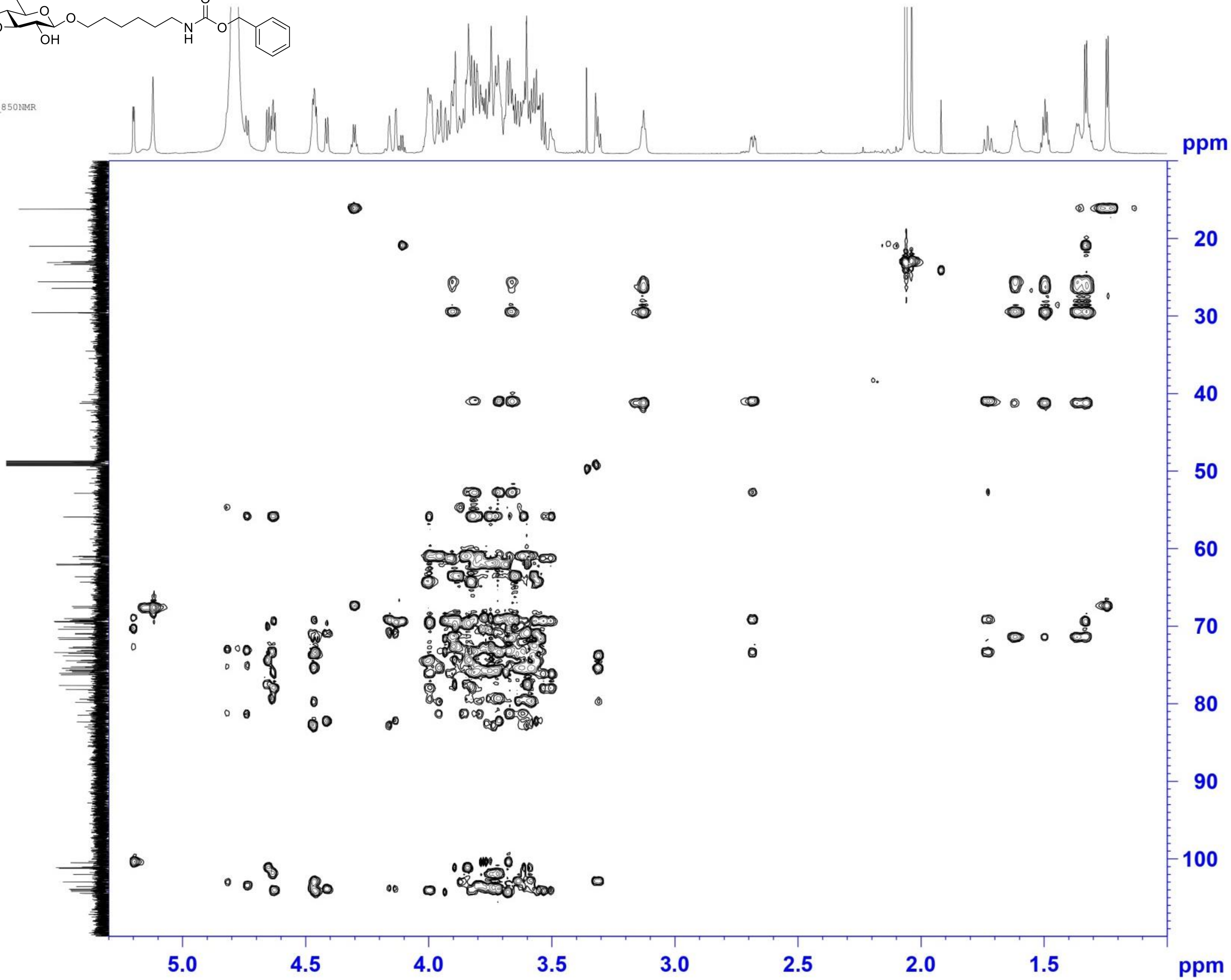

^{1}H-^{13}C HSQC-TOSCY NMR spectrum of **39** (5131a) (850 MHz/214 MHz, D_2O)

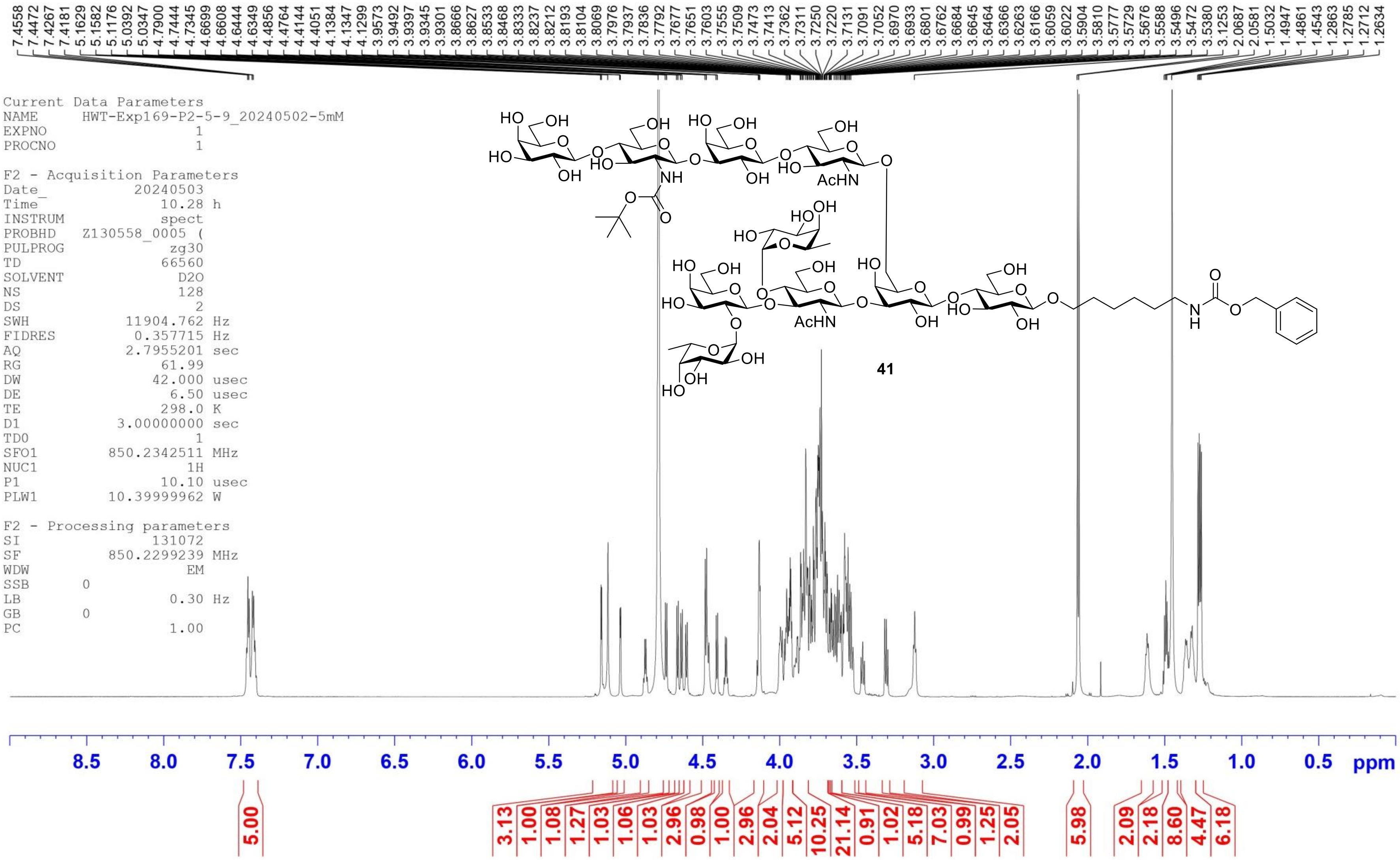

^{1}H NMR spectrum of Compound **41** (850 MHz D_2O)

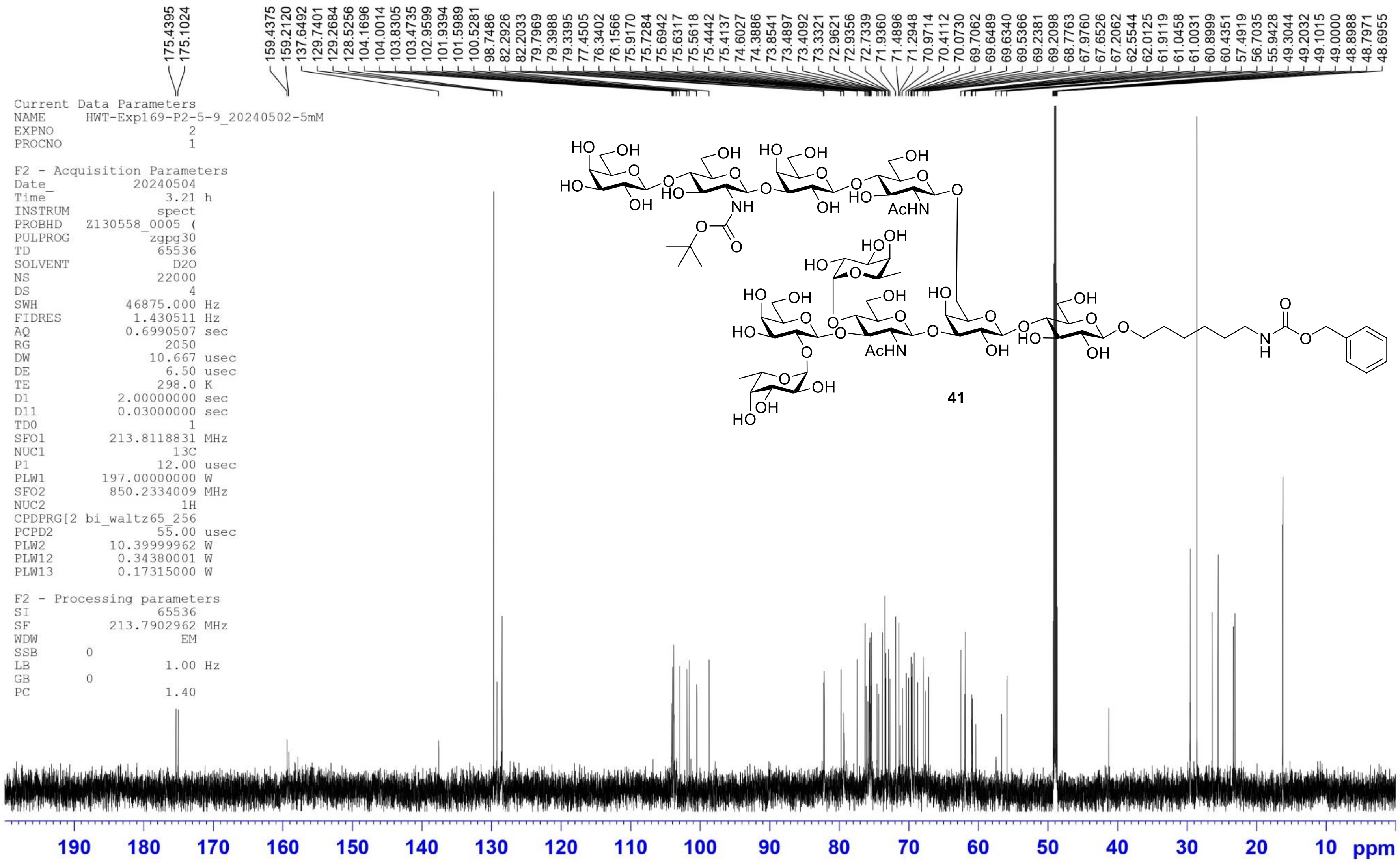

^{13}C NMR spectrum of Compound **41** (214 MHz D_2O)

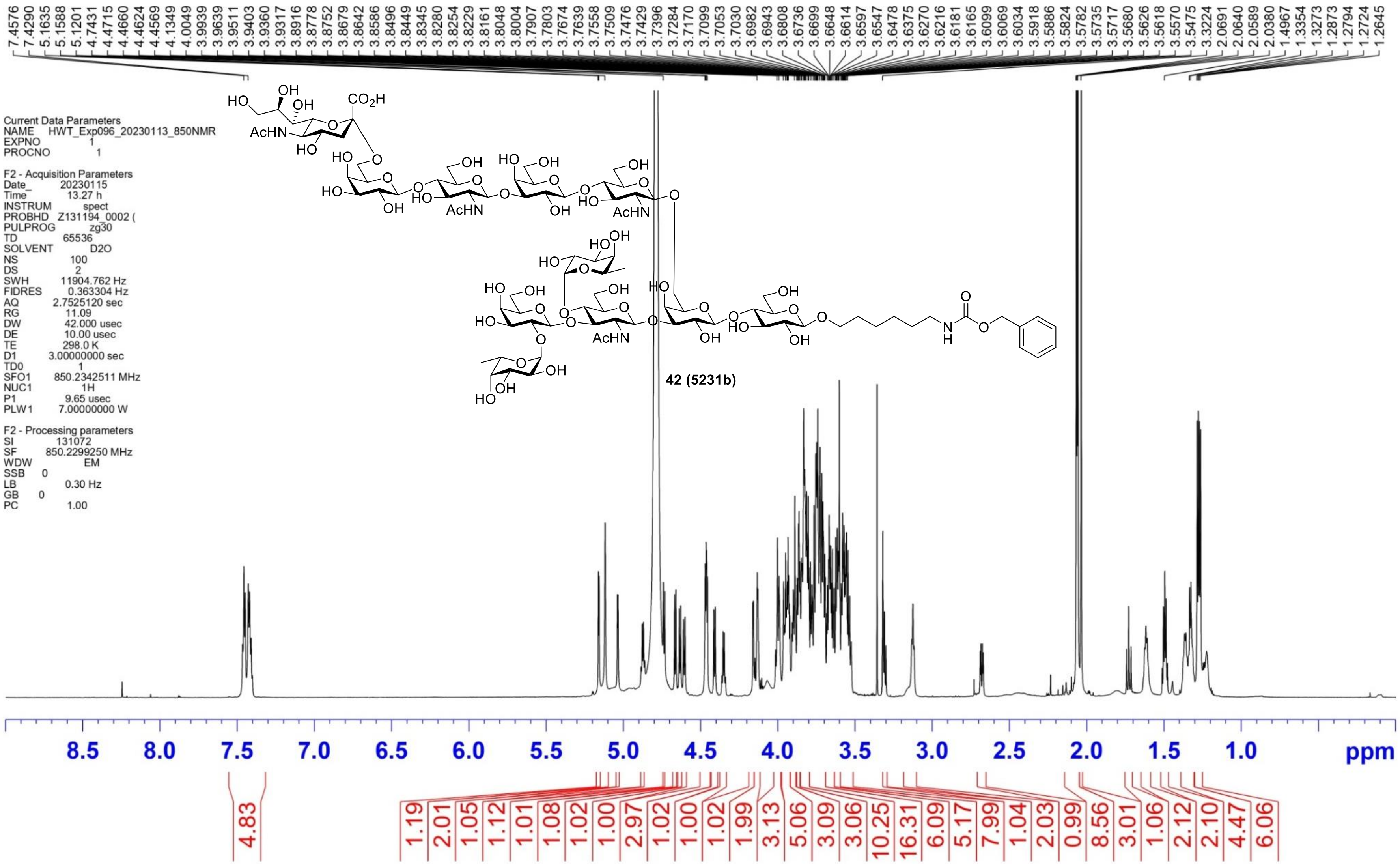

^{1}H NMR spectrum of **42** (5231b) (850 MHz, D_2O)

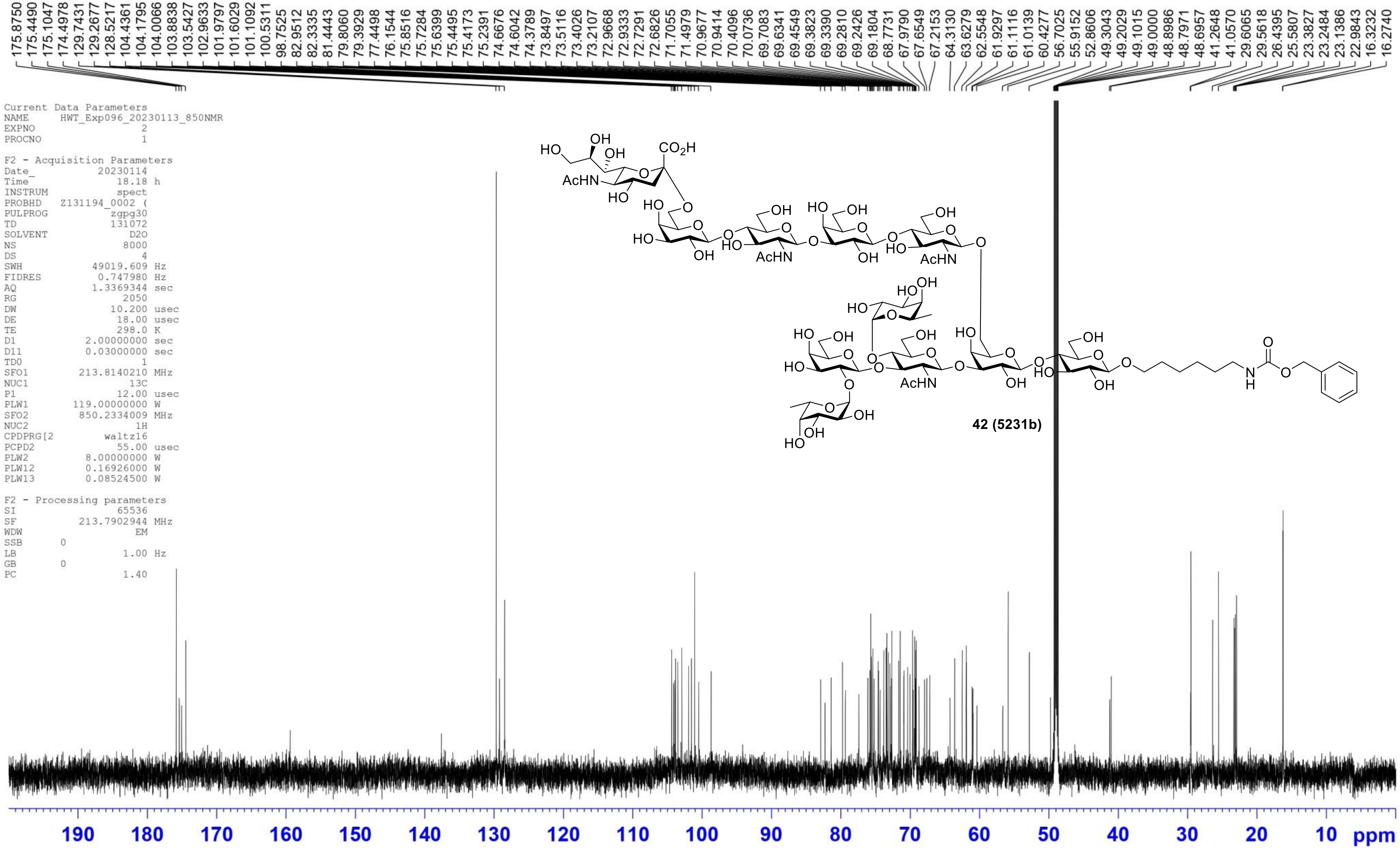

^{13}C NMR spectrum of **42** (5231b) (214 MHz, D_2O)

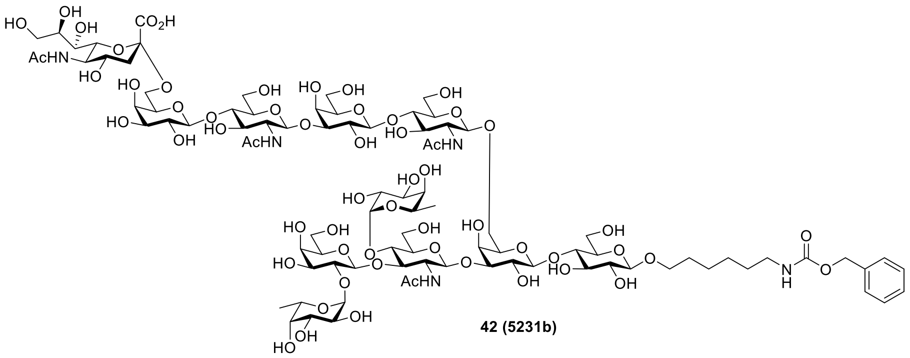

42 (5231b)

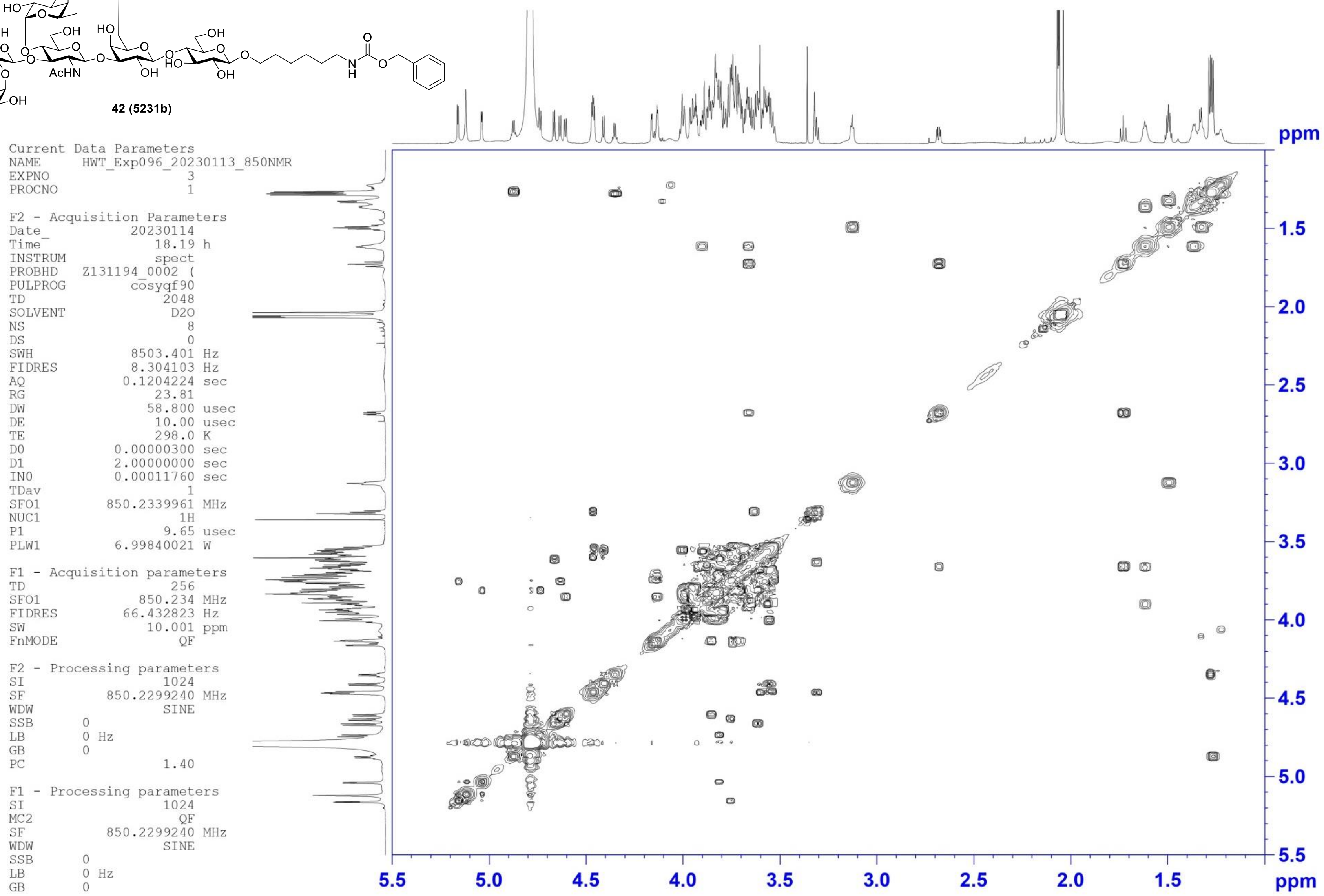

^{1}H-^{1}H COSY NMR spectrum of **42** (5231b) (850 MHz, D_2O)

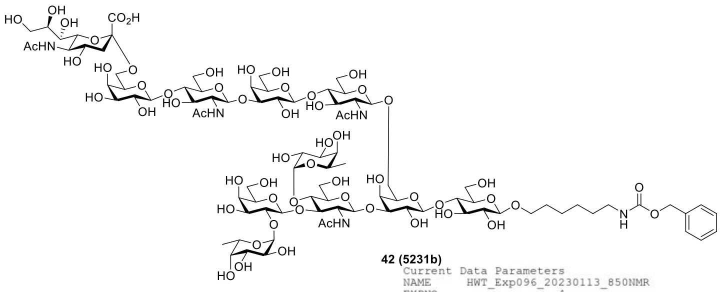

42 (5231b)

```
Current Data Parameters
NAME      HWT_Exp096_20230113_850NMR
EXPNO                  4
PROCNO                 1

F2 - Acquisition Parameters
Date_           20230114
Time               19.33 h
INSTRUM            spect
PROBHD   Z131194_0002 (
PULPROG  hsqcetgpsisp2.2
TD                  2048
SOLVENT              D2O
NS                    24
DS                    16
SWH            11904.762 Hz
FIDRES         11.625744 Hz
AQ             0.0860160 sec
RG                184.37
DW                42.000 usec
DE                 10.00 usec
TE                 298.0 K
CNST2        145.0000000
CNST17        -0.5000000
D0            0.00000300 sec
D1            1.50000000 sec
D4            0.00172414 sec
D11           0.03000000 sec
D16           0.00020000 sec
D24           0.00086207 sec
IN0           0.00001060 sec
TDav                   1
SFO1        850.2351014 MHz
NUC1                  1H
P1                  9.65 usec
P2                 19.30 usec
P28      0 usec
PLW1          6.99840021 W
SFO2        213.8118826 MHz
NUC2                 13C
CPDPRG[2            garp
P3                 12.00 usec
P14               500.00 usec
P24              2000.00 usec
PCPD2              50.00 usec
PLW0     0 W
PLW2        130.00000000 W
PLW12         7.48799992 W
SPNAM[3] Crp80,0.5,20.1
SPOAL3             0.500
SPOFFS3  0 Hz
SPW3         38.13600159 W
SPNAM[7]    Crp80comp.4
SPOAL7             0.500
SPOFFS7  0 Hz
SPW7         38.13600159 W
GPNAM[1]     SMSQ10.100
GPZ1               80.00 %
GPNAM[2]     SMSQ10.100
GPZ2               20.10 %
GPNAM[3]     SMSQ10.100
GPZ3               11.00 %
GPNAM[4]     SMSQ10.100
GPZ4               -5.00 %
P16              1000.00 usec
P19               600.00 usec

F1 - Acquisition parameters
TD                   360
SFO1         213.8119 MHz
FIDRES       262.054504 Hz
SW              220.614 ppm
FnMODE    Echo-Antiecho

F2 - Processing parameters
SI                  1024
SF          850.2299225 MHz
WDW                QSINE
SSB                    2
LB       0 Hz
GB       0
PC                  1.40

F1 - Processing parameters
SI                  1024
MC2       echo-antiecho
SF          213.7902922 MHz
WDW                QSINE
SSB                    2
LB       0 Hz
GB       0
```

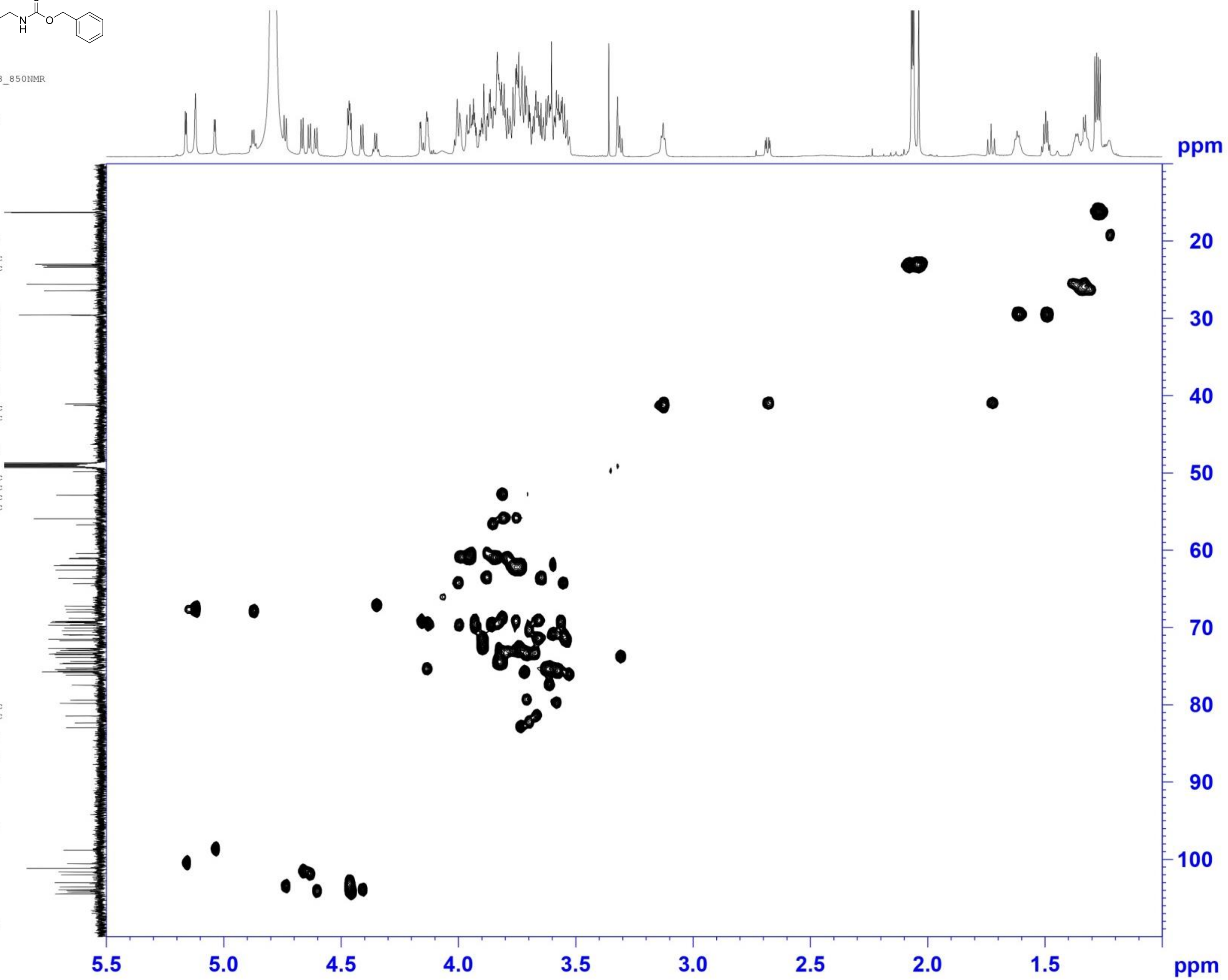

^{1}H-^{13}C HSQC NMR spectrum of **42** (5231b) (850 MHz/214 MHz, D_2O)

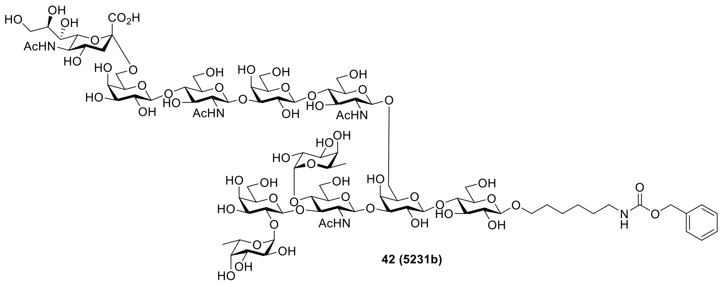

42 (5231b)

```
Current Data Parameters
NAME      HWT_Exp096_20230113_850NMR
EXPNO                  5
PROCNO                 1

F2 - Acquisition Parameters
Date_           20230114
Time               23.26 h
INSTRUM            spect
PROBHD    Z131194_0002 (
PULPROG   clhmbcetgpl3nd
TD                  2048
SOLVENT              D2O
NS                    40
DS                    16
SWH             8503.401 Hz
FIDRES          8.304103 Hz
AQ             0.1204224 sec
RG                184.37
DW                58.800 usec
DE                 10.00 usec
TE                 298.0 K
CNST6        125.0000000
CNST7        165.0000000
CNST13         8.0000000
D0            0.00000300 sec
D1            1.50000000 sec
D6            0.06250000 sec
D16           0.00020000 sec
D21       0 sec
IN0           0.00001170 sec
L0        0
TDav                   1
SFO1         850.2339961 MHz
NUC1                  1H
P1                  9.65 usec
P2                 19.30 usec
PLW1          6.99840021 W
SFO2         213.8118831 MHz
NUC2                 13C
P3                 12.00 usec
P14               500.00 usec
P24              2000.00 usec
PLW2        130.00000000 W
SPNAM[3]  Crp80,0.5,20.1
SPOAL3             0.500
SPOFFS3   0 Hz
SPW3         38.13600159 W
SPNAM[7]     Crp80comp.4
SPOAL7             0.500
SPOFFS7   0 Hz
SPW7         38.13600159 W
GPNAM[1]      SMSQ10.100
GPZ1               80.00 %
GPNAM[3]      SMSQ10.100
GPZ3               14.00 %
P16              1000.00 usec

F1 - Acquisition parameters
TD                   360
SFO1            213.8119 MHz
FIDRES        237.416901 Hz
SW               199.872 ppm
FnMODE     Echo-Antiecho

F2 - Processing parameters
SI                  1024
SF           850.2299254 MHz
WDW                QSINE
SSB                    2
LB        0 Hz
GB        0
PC                  1.40

F1 - Processing parameters
SI                  1024
MC2        echo-antiecho
SF           213.7902964 MHz
WDW                QSINE
SSB                    2
LB        0 Hz
GB        0
```

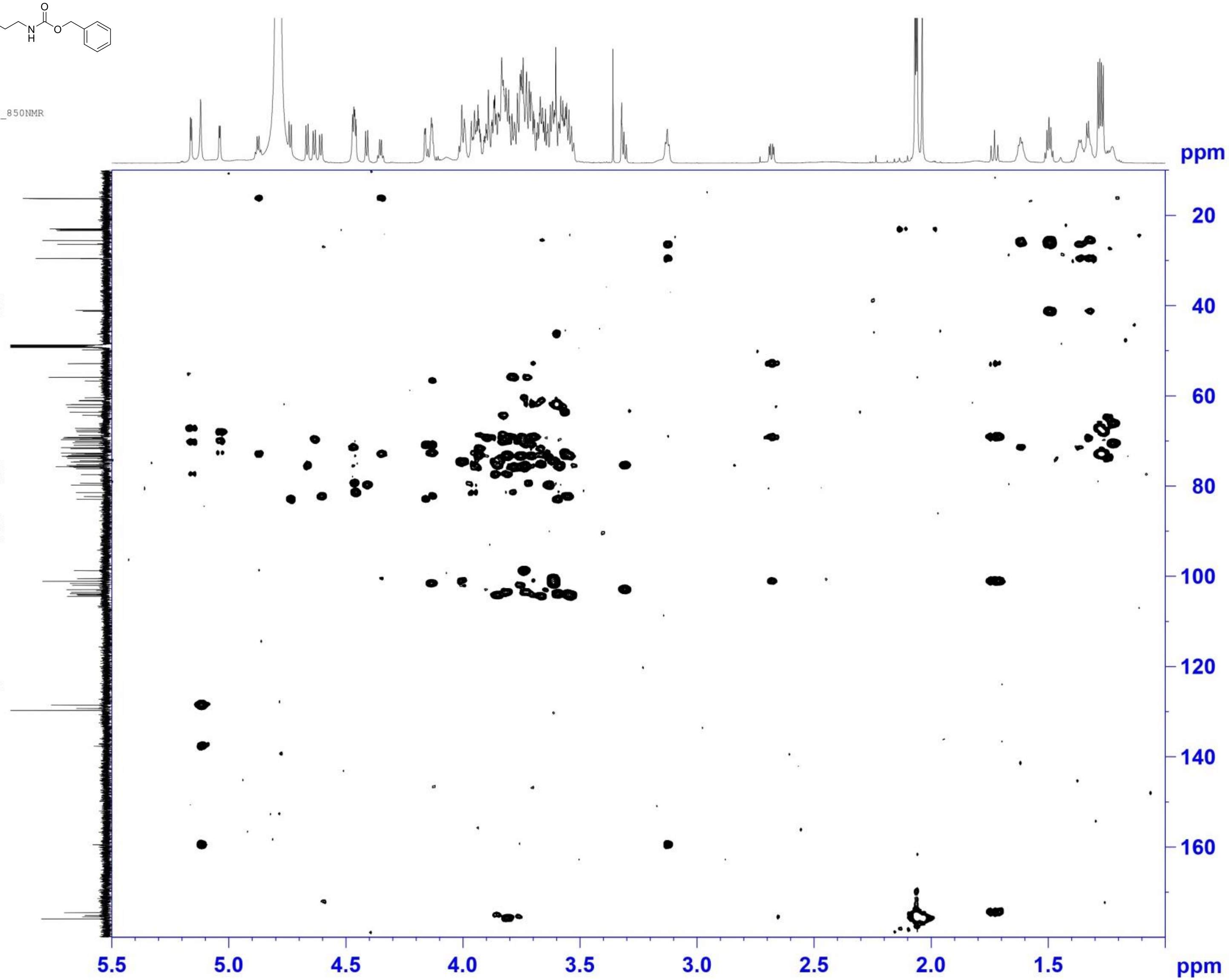

^{1}H-^{13}C HMBC NMR spectrum of **42** (5231b) (850 MHz/214 MHz, D_2O)

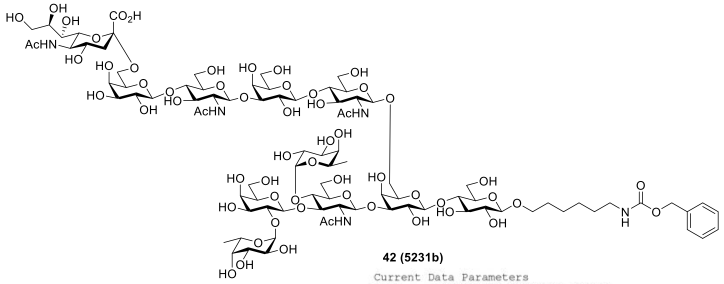

42 (5231b)

```
Current Data Parameters
NAME      HWT_Exp096_20230113_850NMR
EXPNO                    6
PROCNO                   1

F2 - Acquisition Parameters
Date_             20230115
Time                  6.15 h
INSTRUM              spect
PROBHD    Z131194_0002 (
PULPROG   hsqcdietgpsisp.2
TD                    2048
SOLVENT                D2O
NS                      32
DS                       8
SWH               8503.401 Hz
FIDRES            8.304103 Hz
AQ               0.1204224 sec
RG                  184.37
DW                  58.800 usec
DE                   10.00 usec
TE                   298.0 K
CNST2          145.0000000
CNST17          -0.5000000
D0              0.00000300 sec
D1              2.00000000 sec
D4              0.00172414 sec
D9              0.06000000 sec
D11             0.03000000 sec
D16             0.00020000 sec
D24             0.00089000 sec
IN0             0.00000970 sec
L1                      26
TDav                     1
SFO1           850.2340046 MHz
NUC1                    1H
P1                    9.65 usec
P2                   19.30 usec
P6                   20.00 usec
P28        0 usec
PLW1            6.99840021 W
PLW10           1.62930000 W
SFO2           213.8076072 MHz
NUC2                   13C
CPDPRG[2              garp
P3                   12.00 usec
P14                 500.00 usec
P24                2000.00 usec
PCPD2                50.00 usec
PLW0       0 W
PLW2          130.00000000 W
PLW12           7.48799992 W
SPNAM[3] Crp80,0.5,20.1
SPOAL3                0.500
SPOFFS3  0 Hz
SPW3           38.13600159 W
SPNAM[7]      Crp80comp.4
SPOAL7                0.500
SPOFFS7  0 Hz
SPW7           38.13600159 W
GPNAM[1]       SMSQ10.100
GPZ1                 80.00 %
GPNAM[2]       SMSQ10.100
GPZ2                 20.10 %
P16                1000.00 usec

F1 - Acquisition parameters
TD                     360
SFO1              213.8076 MHz
FIDRES          286.368835 Hz
SW                 241.088 ppm
FnMODE     Echo-Antiecho

F2 - Processing parameters
SI                    1024
SF             850.2299265 MHz
WDW                  QSINE
SSB                      2
LB         0 Hz
GB         0
PC                    1.40

F1 - Processing parameters
SI                    1024
MC2         echo-antiecho
SF             213.7902909 MHz
WDW                  QSINE
SSB                      2
LB         0 Hz
GB         0
```

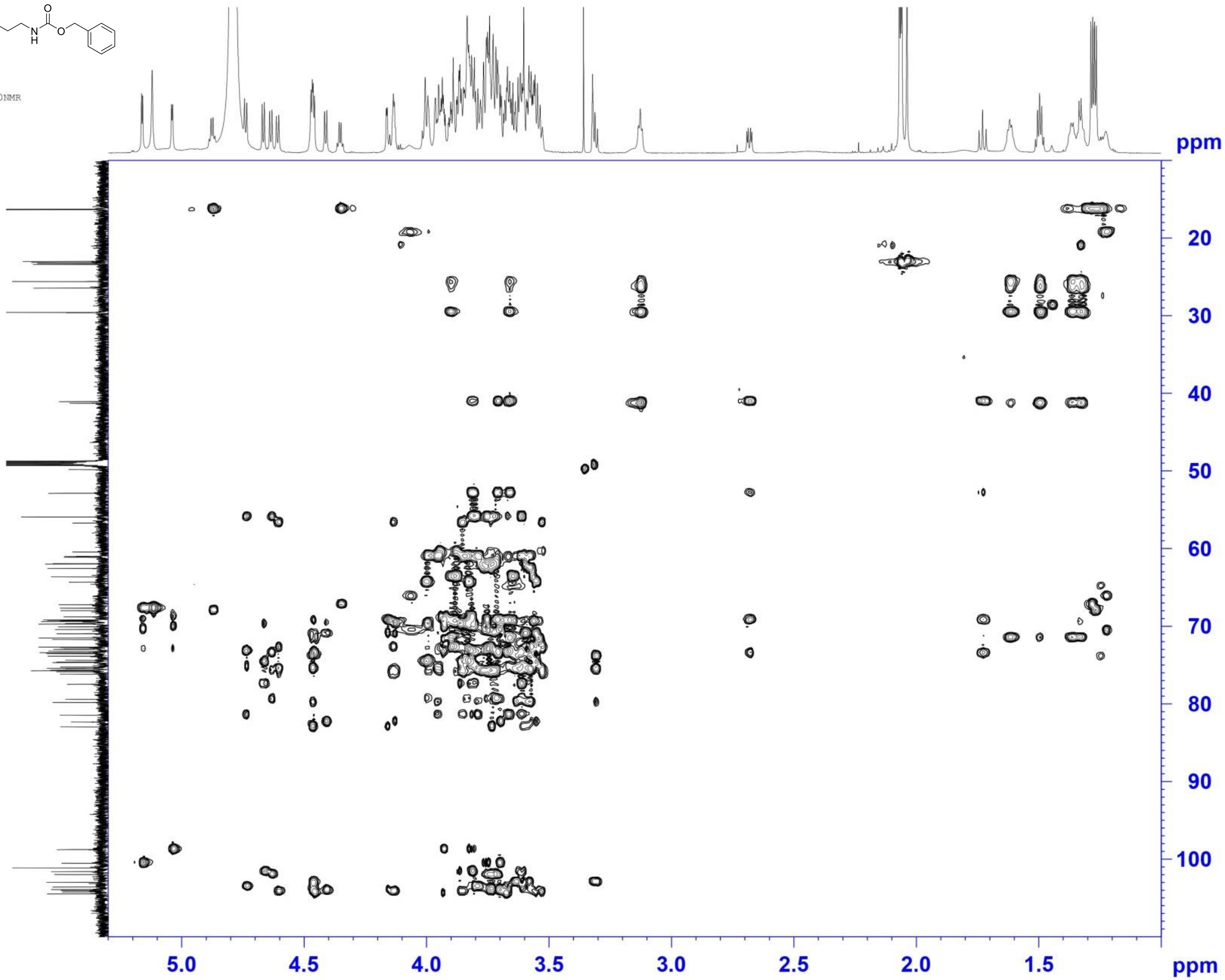

^{1}H-^{13}C HSQC-TOSCY NMR spectrum of **42** (5231b) (850 MHz/214 MHz, D_2O)

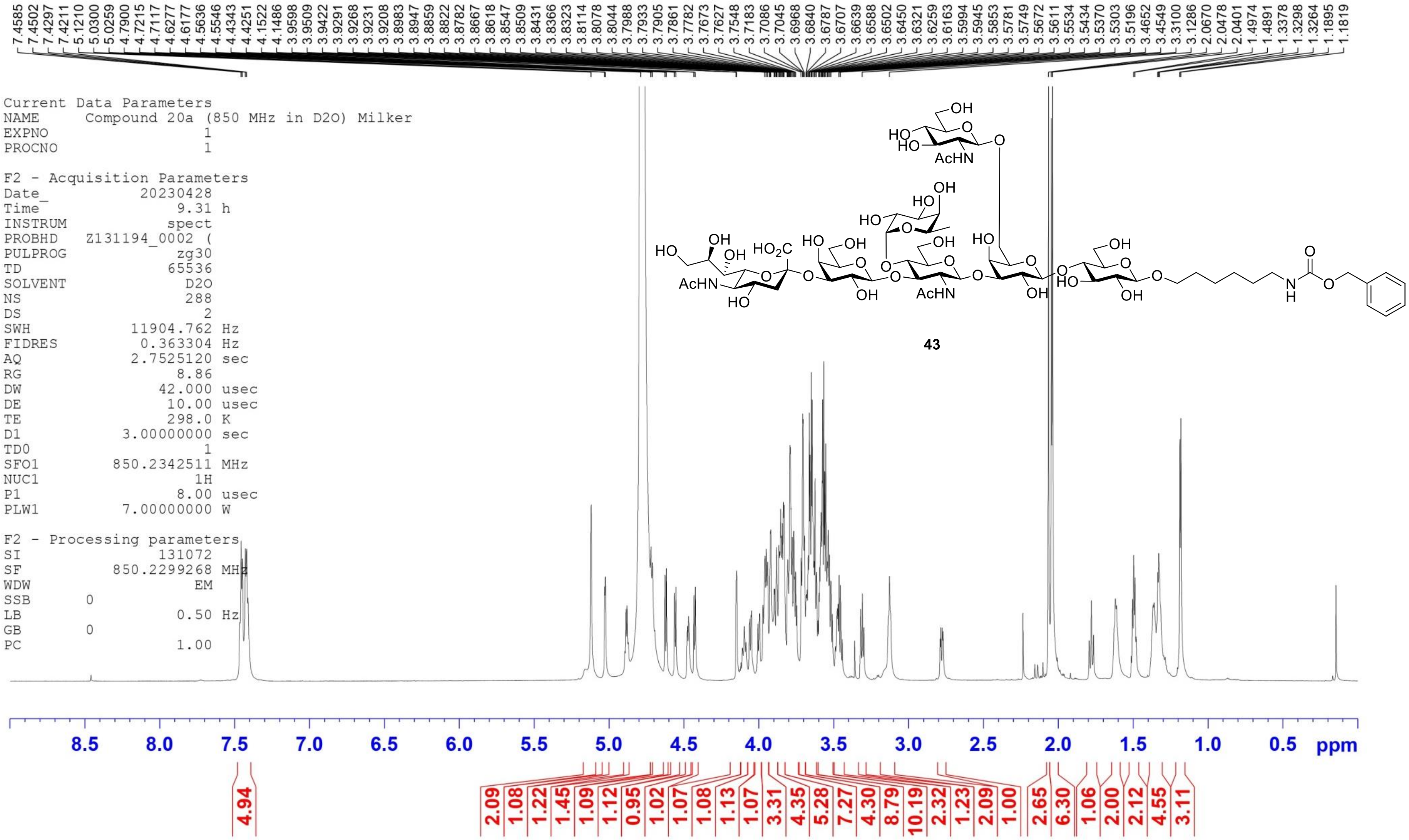

^{1}H NMR spectrum of **43** (850 MHz, D_2O)

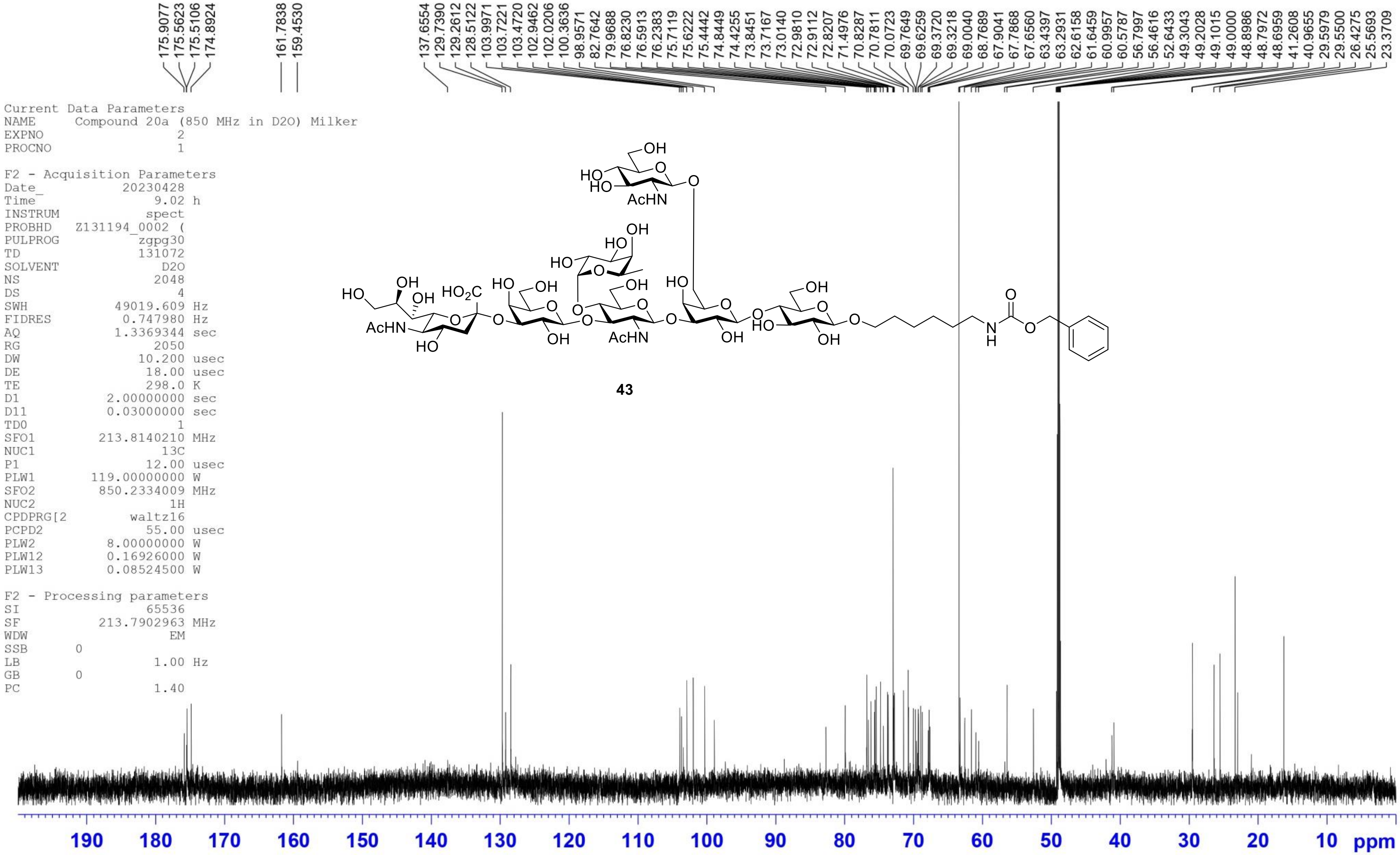

^{13}C NMR spectrum of **43** (214 MHz, D_2O)

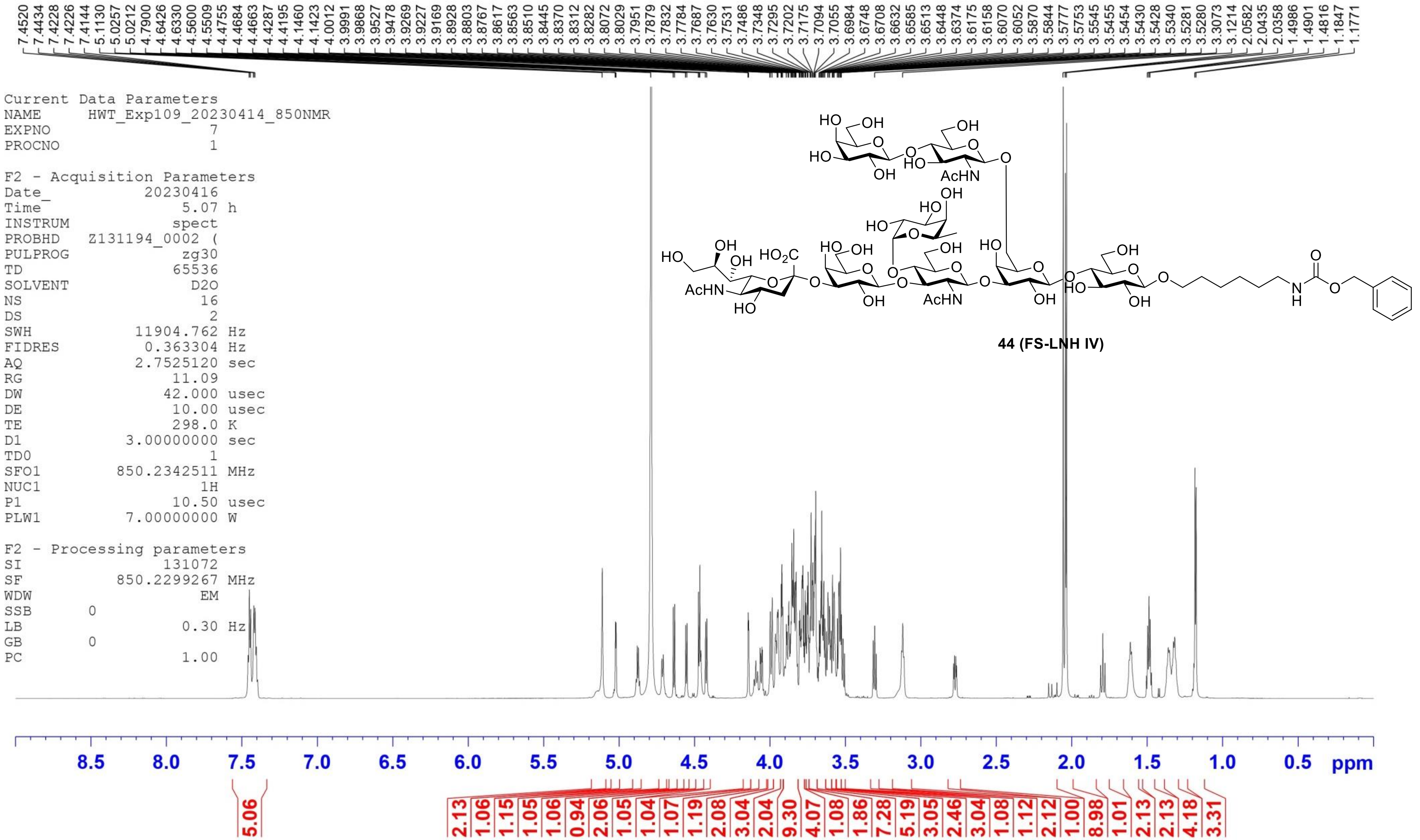

^{1}H NMR spectrum of **44** (FS-LNH IV) (850 MHz, D_2O)

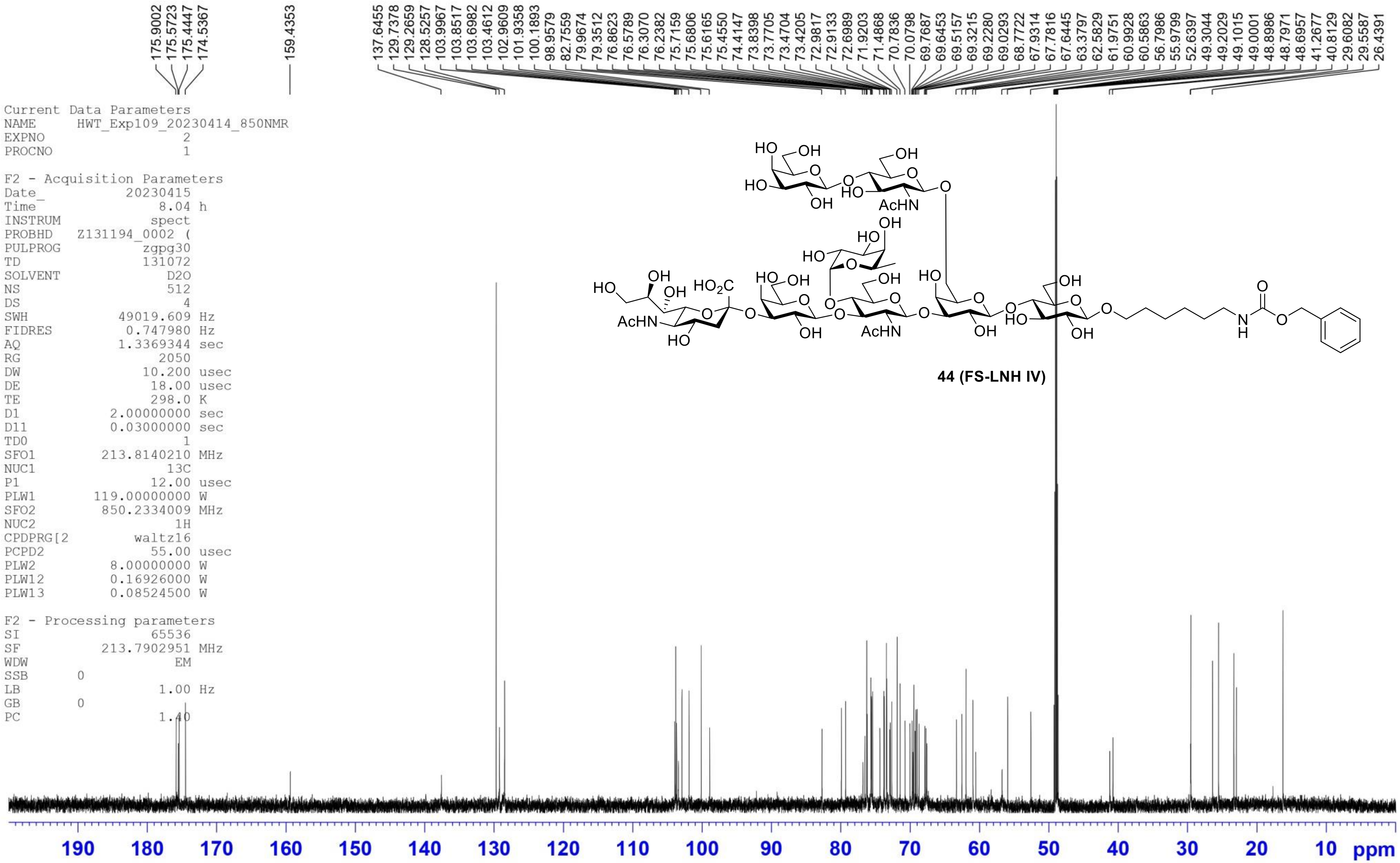

^{13}C NMR spectrum of **44** (FS-LNH IV) (214 MHz, D_2O)

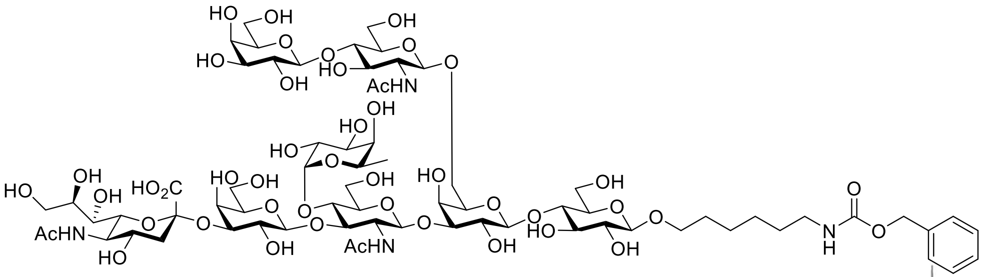

44 (FS-LNH IV)

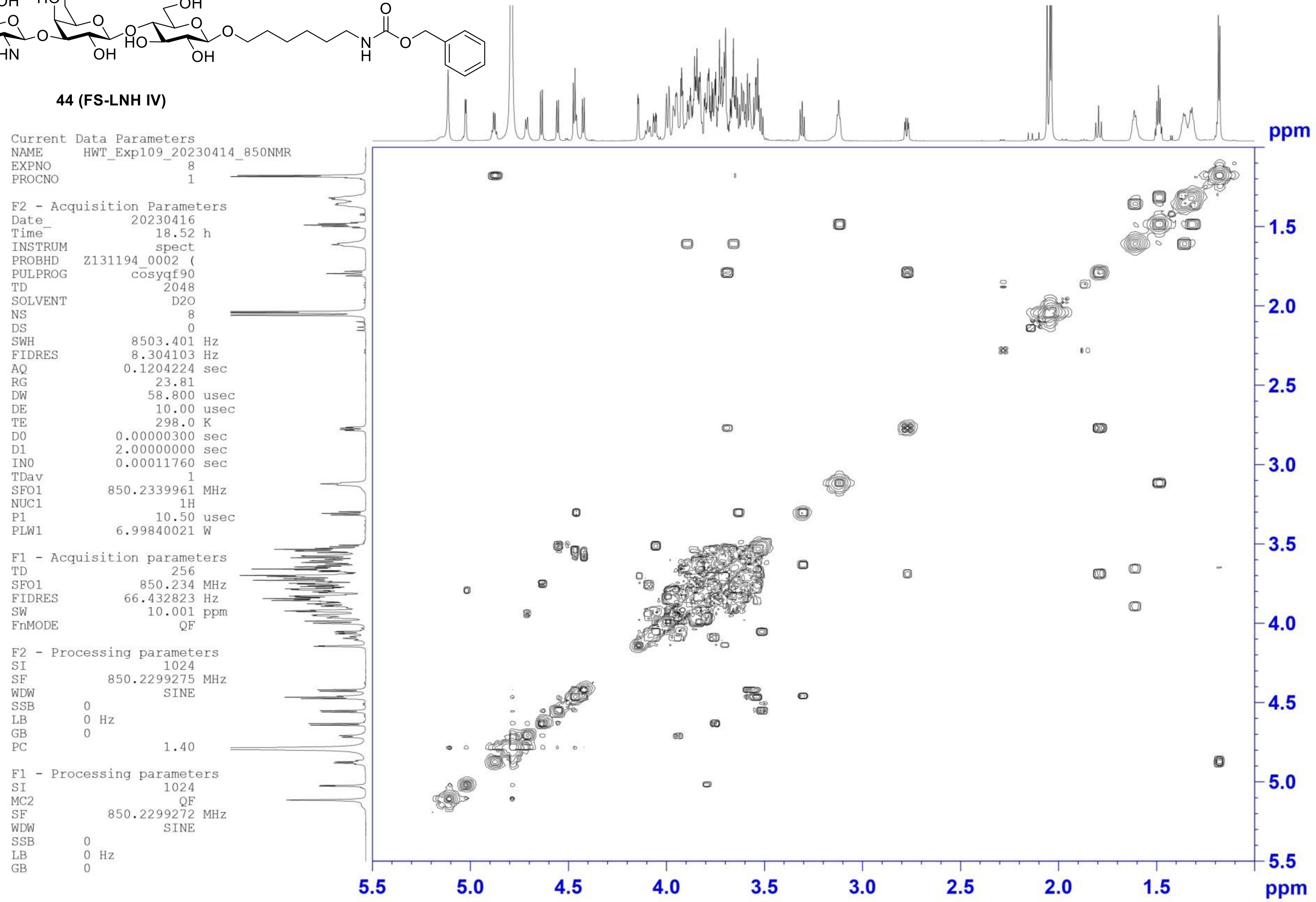

^1H-^1H COSY NMR spectrum of **44** (FS-LNH IV) (850 MHz, D_2O)

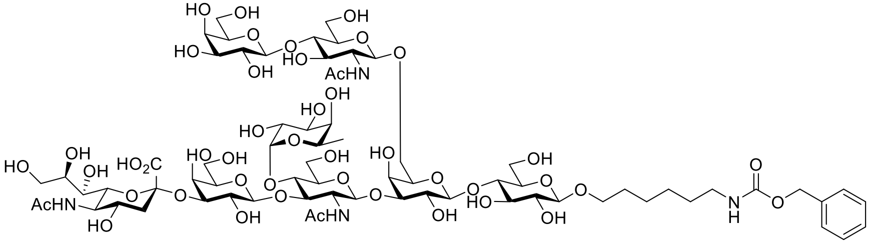

44 (FS-LNH IV)

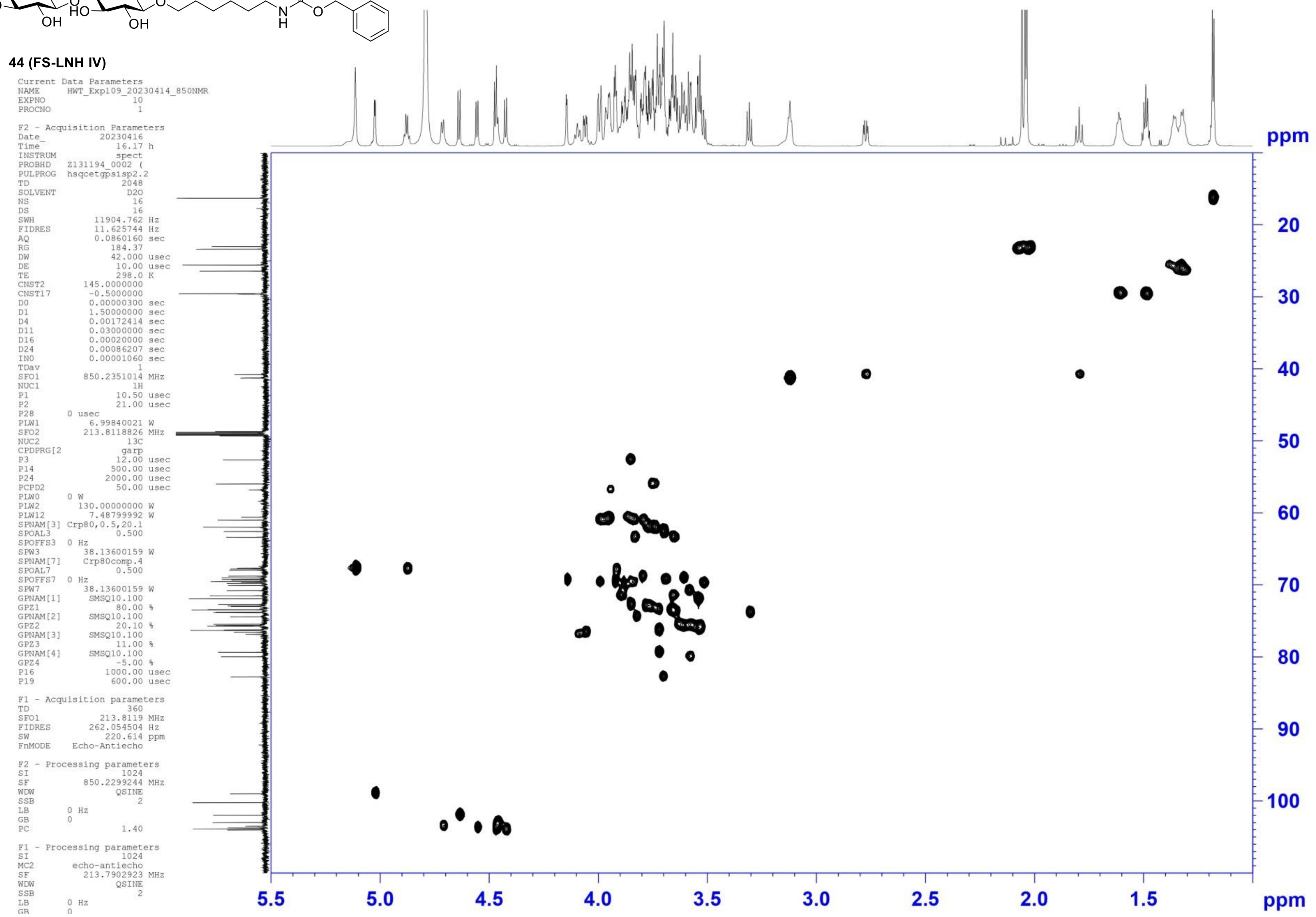

^{1}H-^{13}C HSQC NMR spectrum of **44** (FS-LNH IV) (850 MHz/214 MHz, D_2O)

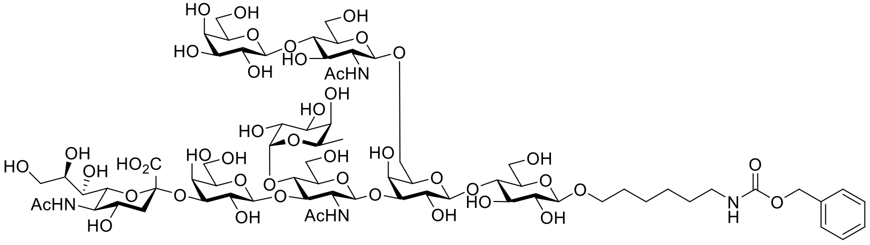

44 (FS-LNH IV)

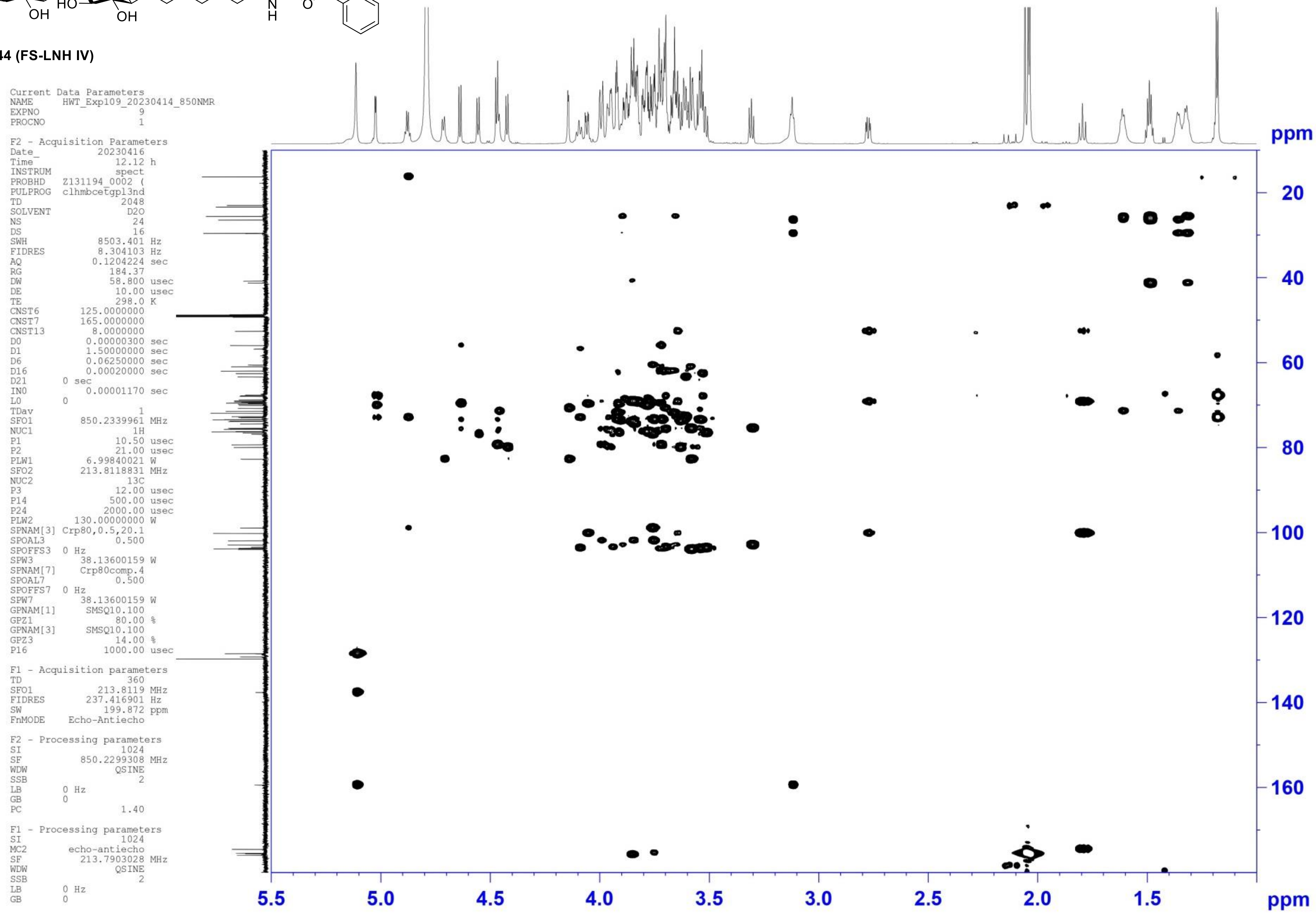

^{1}H-^{13}C HMBC NMR spectrum of **44** (FS-LNH IV) (850 MHz/214 MHz, D_2O)

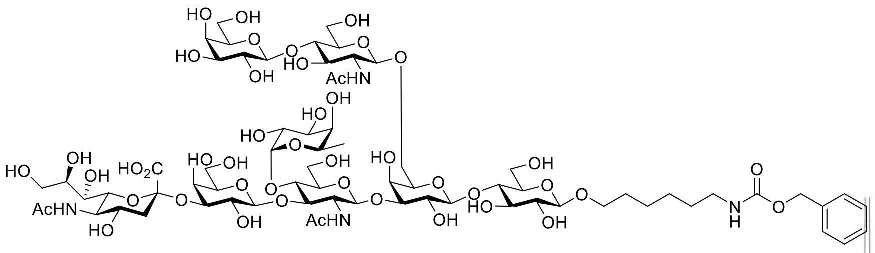

44 (FS-LNH IV)

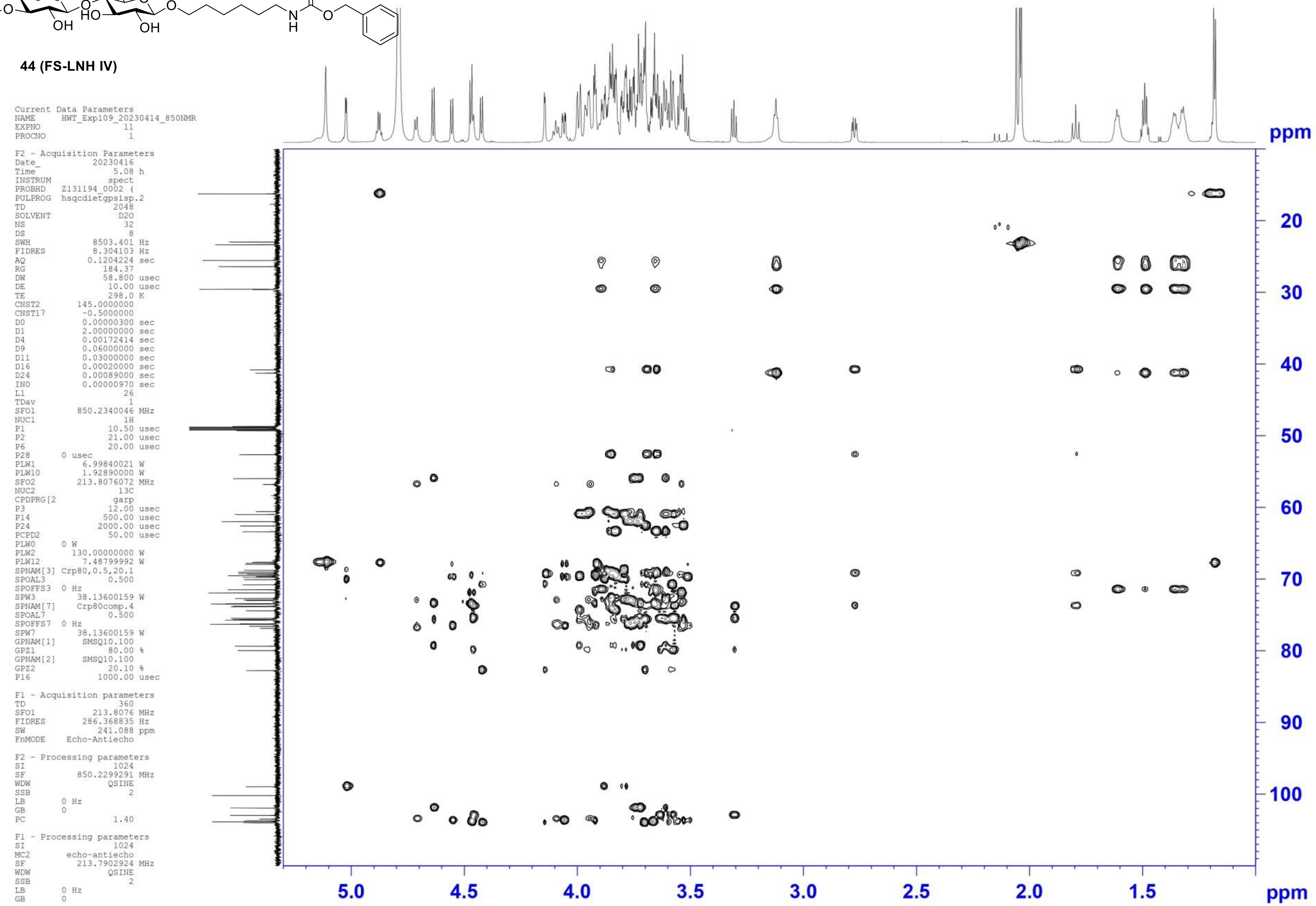

^{1}H-^{13}C HSQC-TOSCY NMR spectrum of **44** (FS-LNH IV) (850 MHz/214 MHz, D_2O)

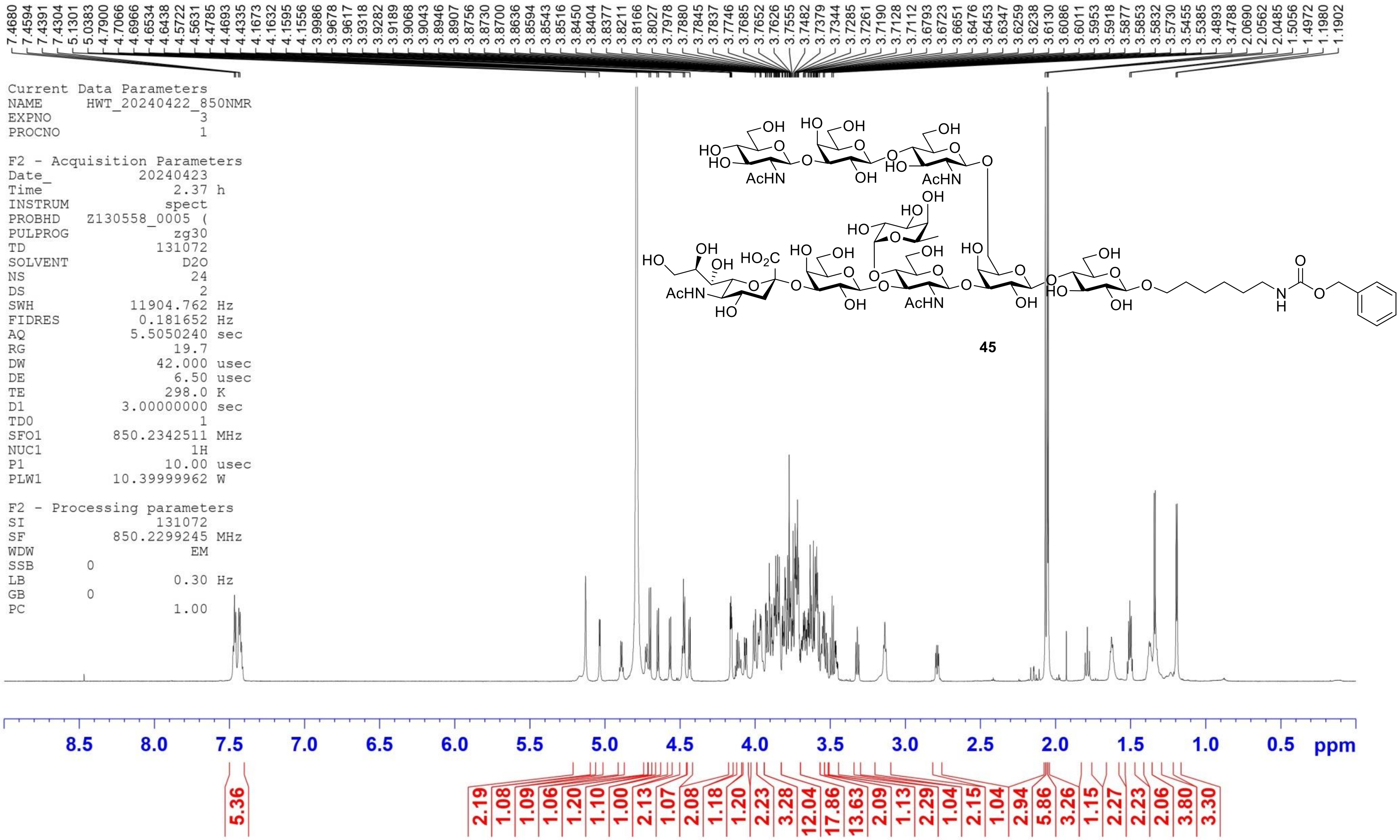

^{1}H NMR spectrum of Compound **45** (850 MHz D_2O)

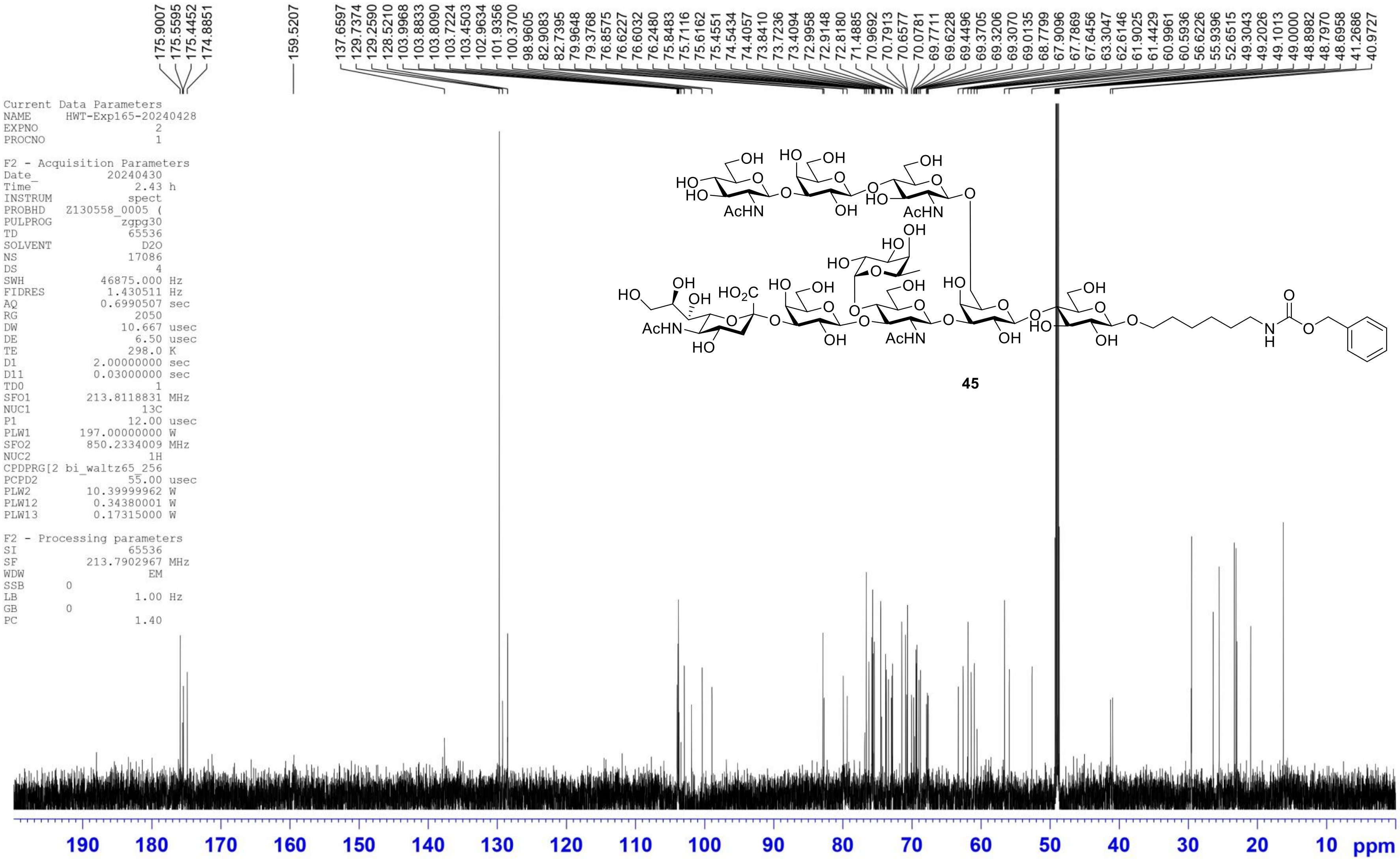

^{13}C NMR spectrum of Compound **45** (214 MHz D_2O)

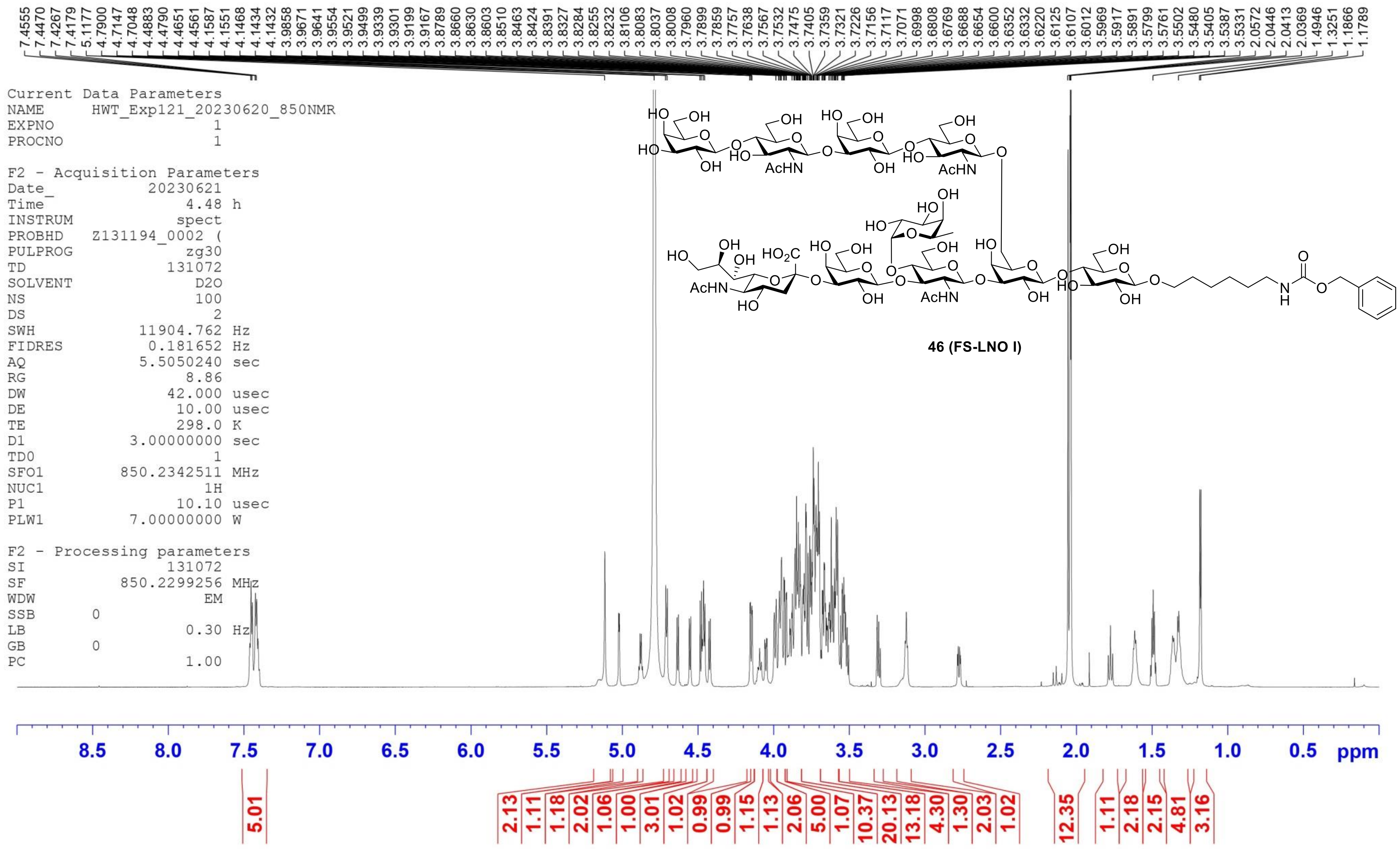

^{1}H NMR spectrum of **46** (FS-LNO I) (850 MHz, D_2O)

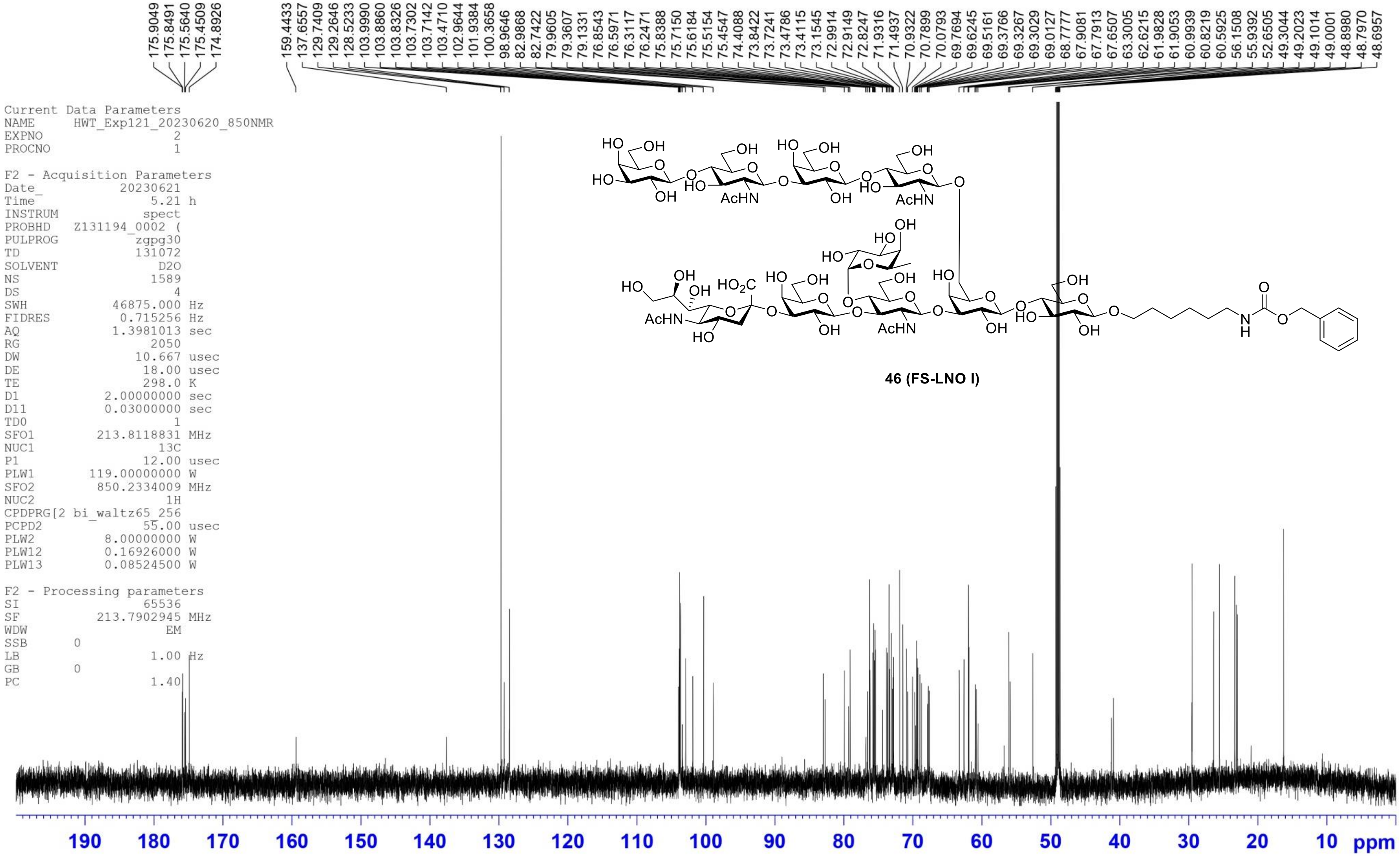

^{13}C NMR spectrum of **46** (FS-LNO I) (214 MHz, D_2O)

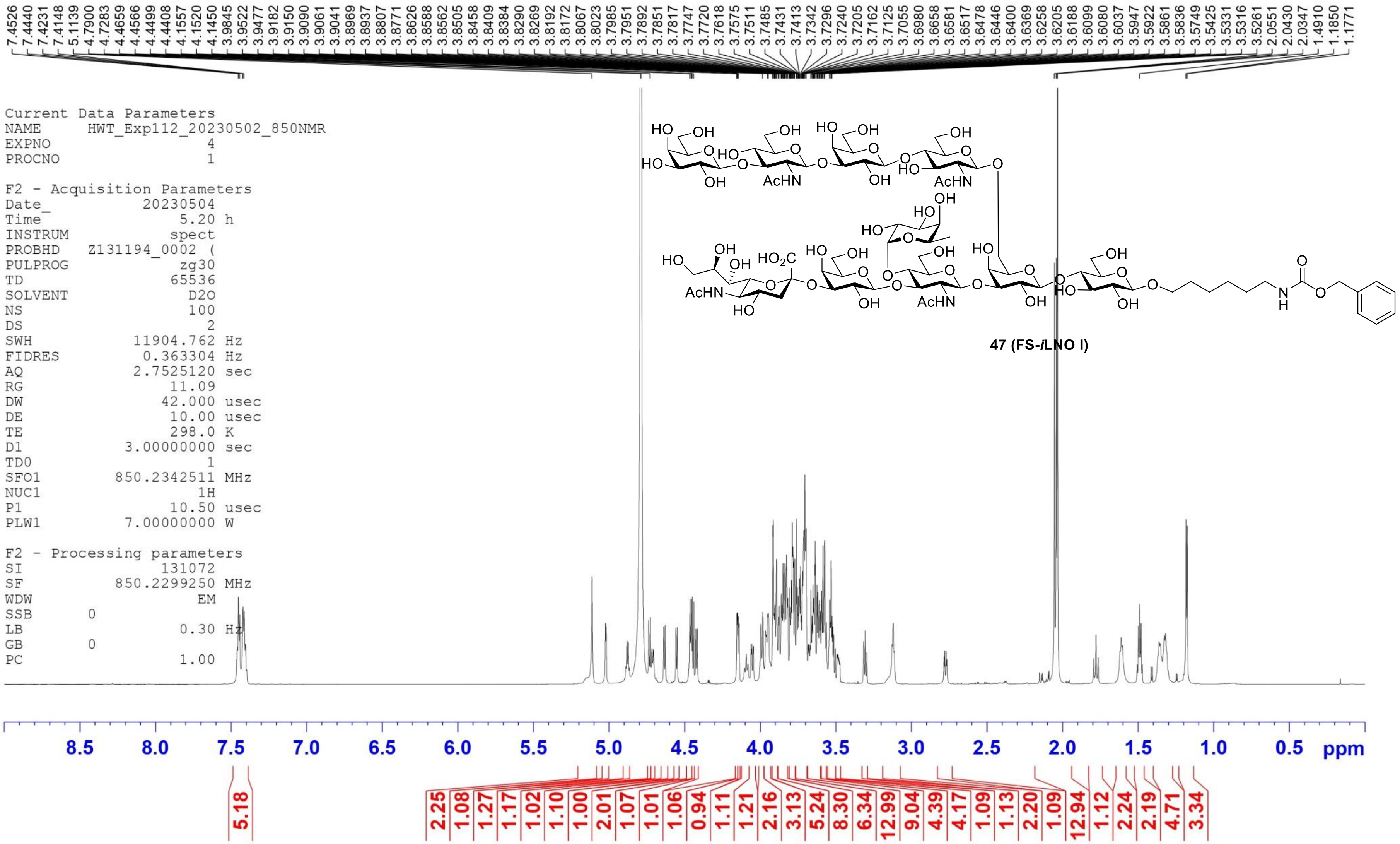

^{1}H NMR spectrum of **47** (FS-*i*LNO I) (850 MHz, D_2O)

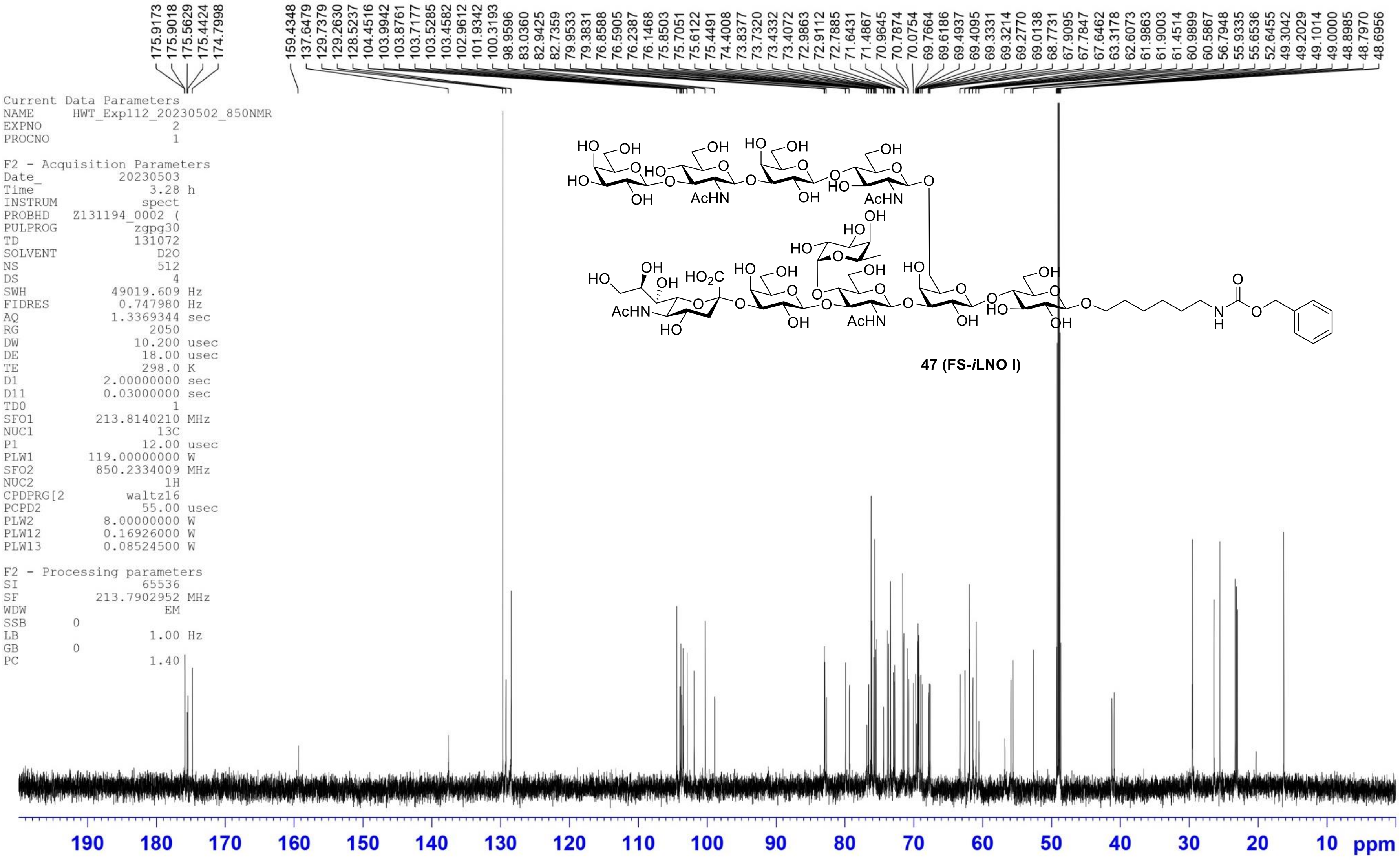

^{13}C NMR spectrum of **47** (FS-*i*LNO I) (214 MHz, D_2O)

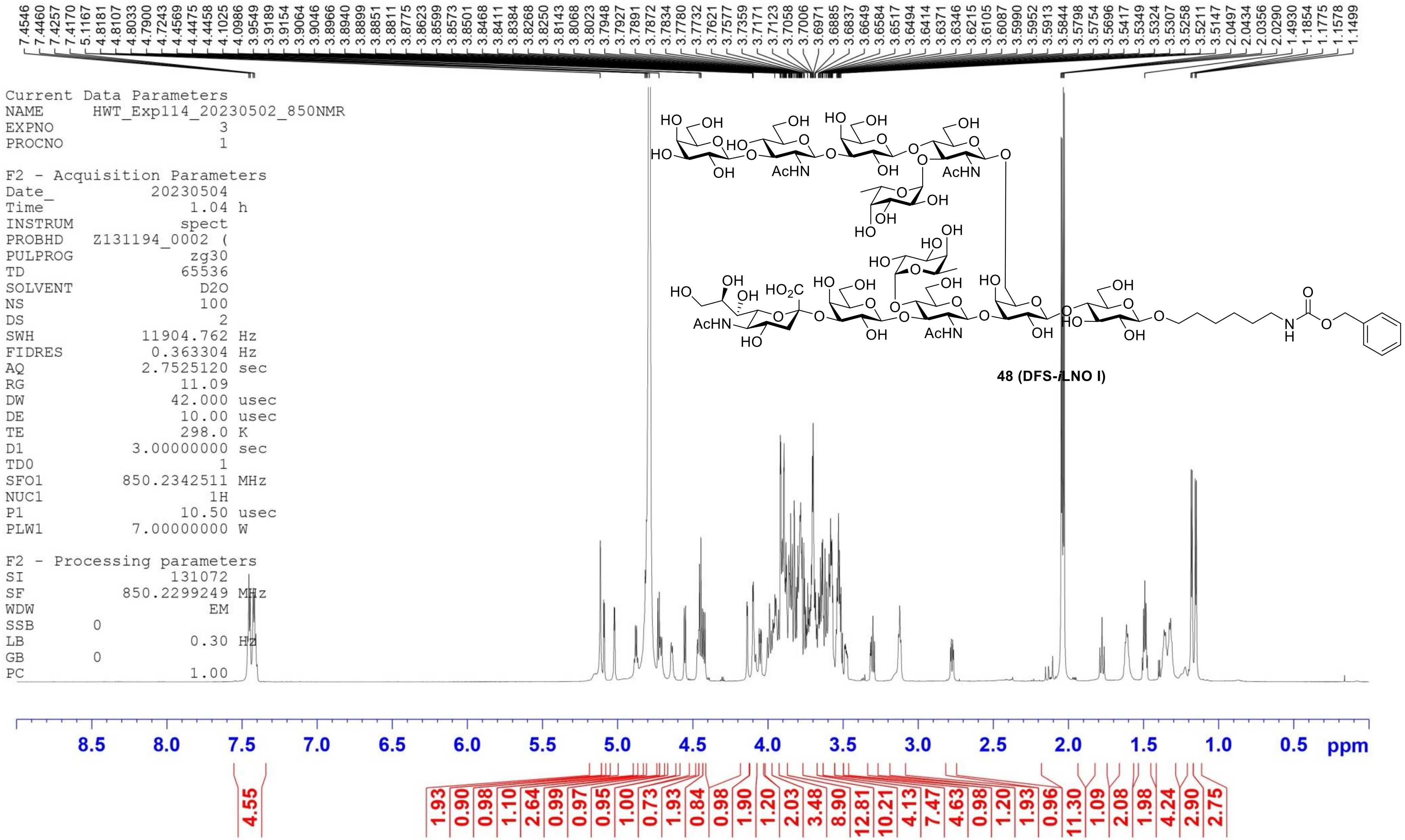

^{1}H NMR spectrum of **48** (DFS-*i*LNO I) (850 MHz, D_2O)

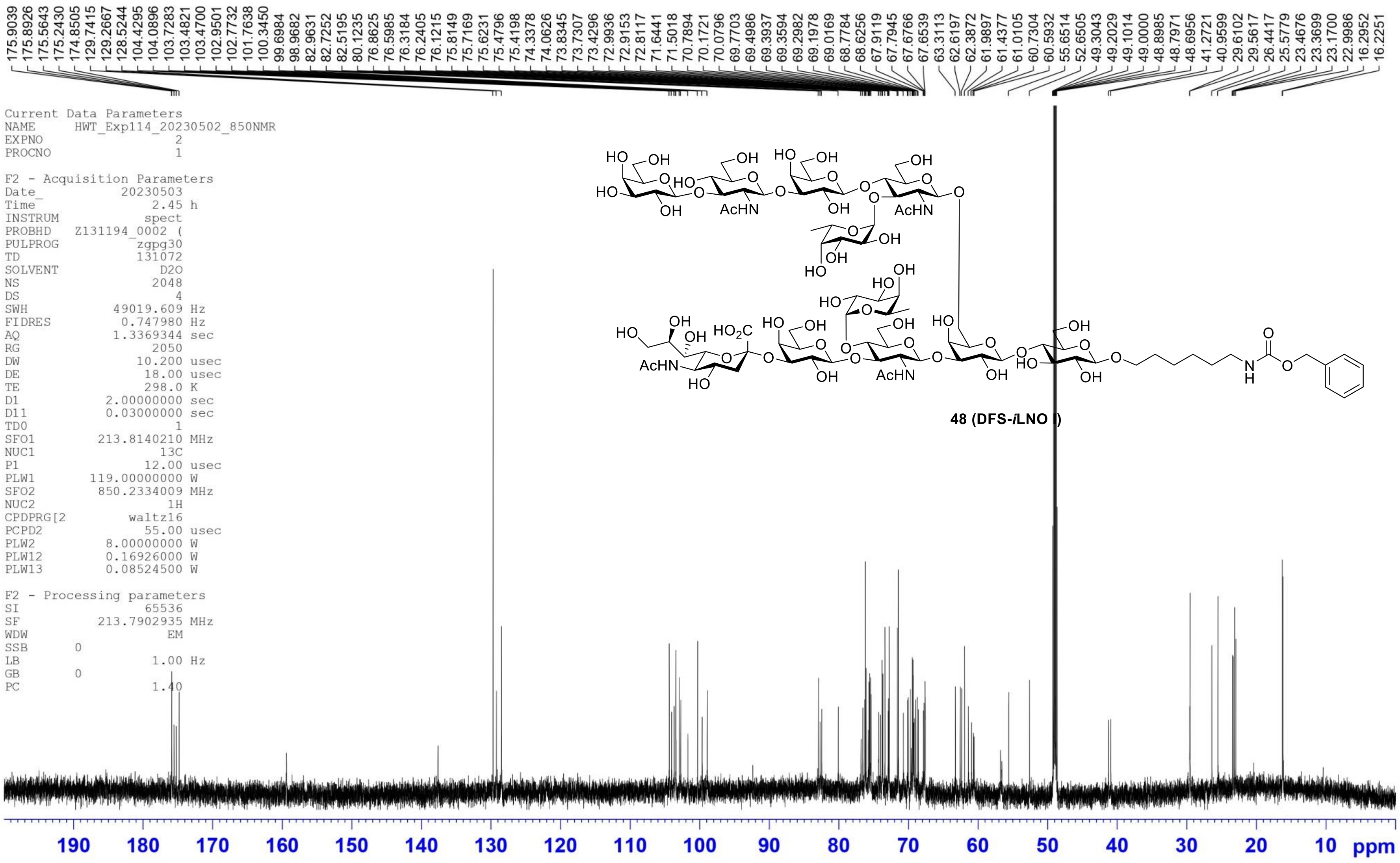

^{13}C NMR spectrum of **48** (DFS-*i*LNO I) (214 MHz, D_2O)

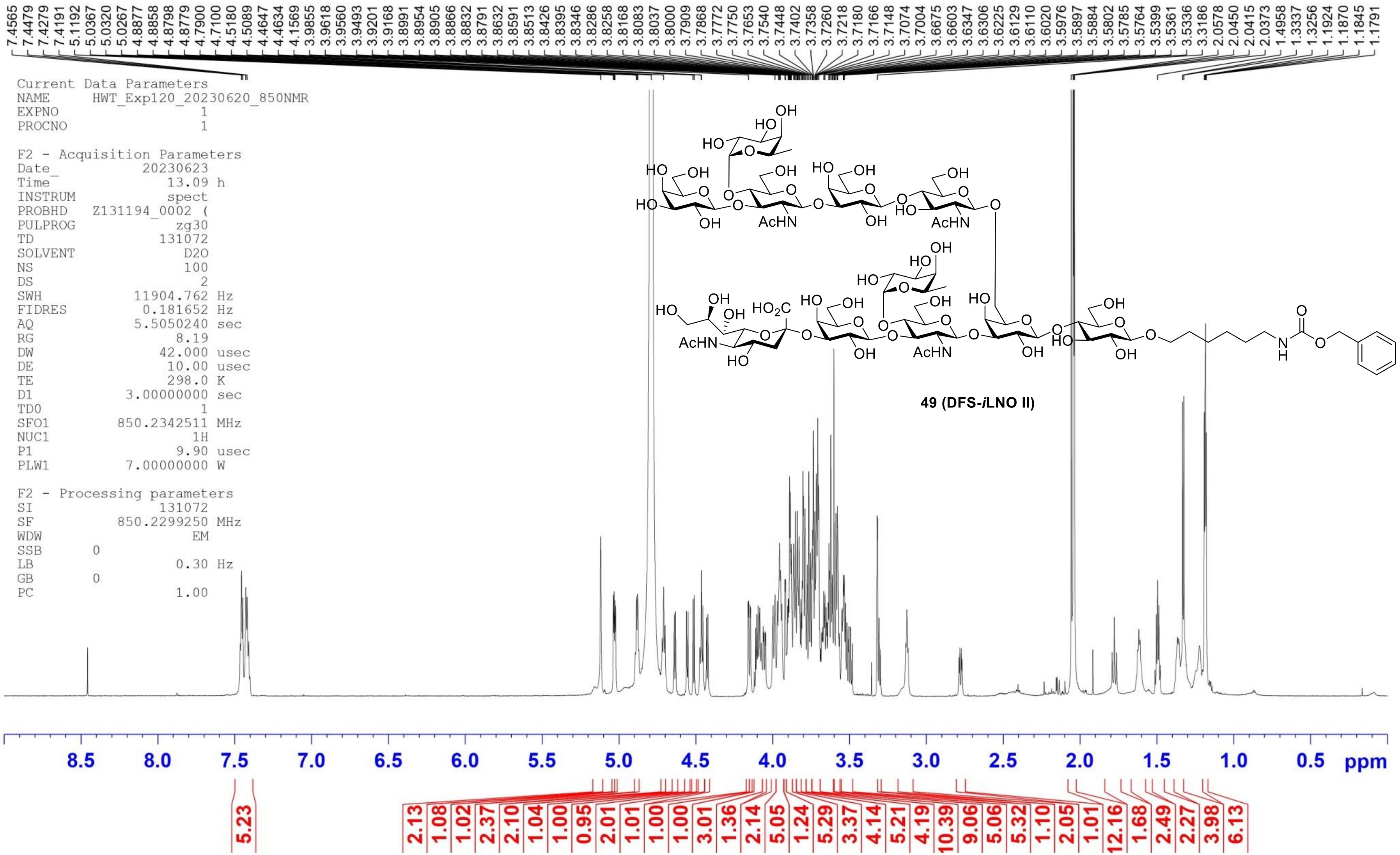

^{1}H NMR spectrum of **49** (DFS-*i*LNO II) (850 MHz, D_2O)

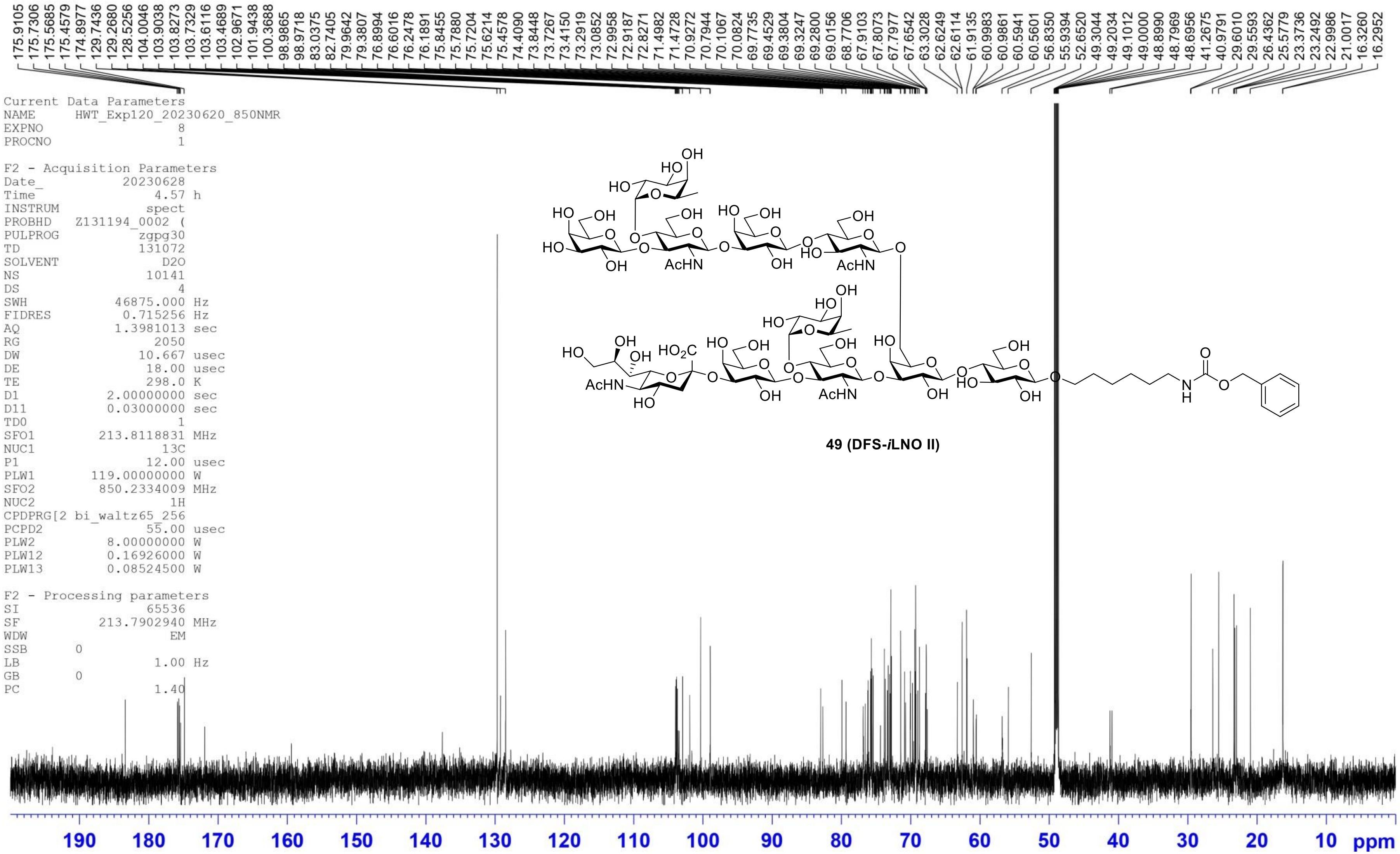

^{13}C NMR spectrum of **49** (DFS-*i*LNO II) (214 MHz, D_2O)

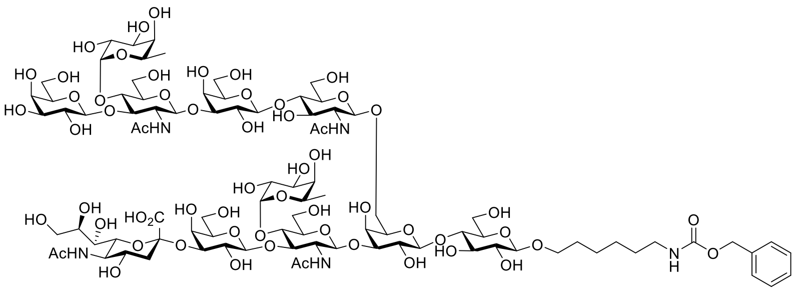

49 (DFS-*i*LNO II)

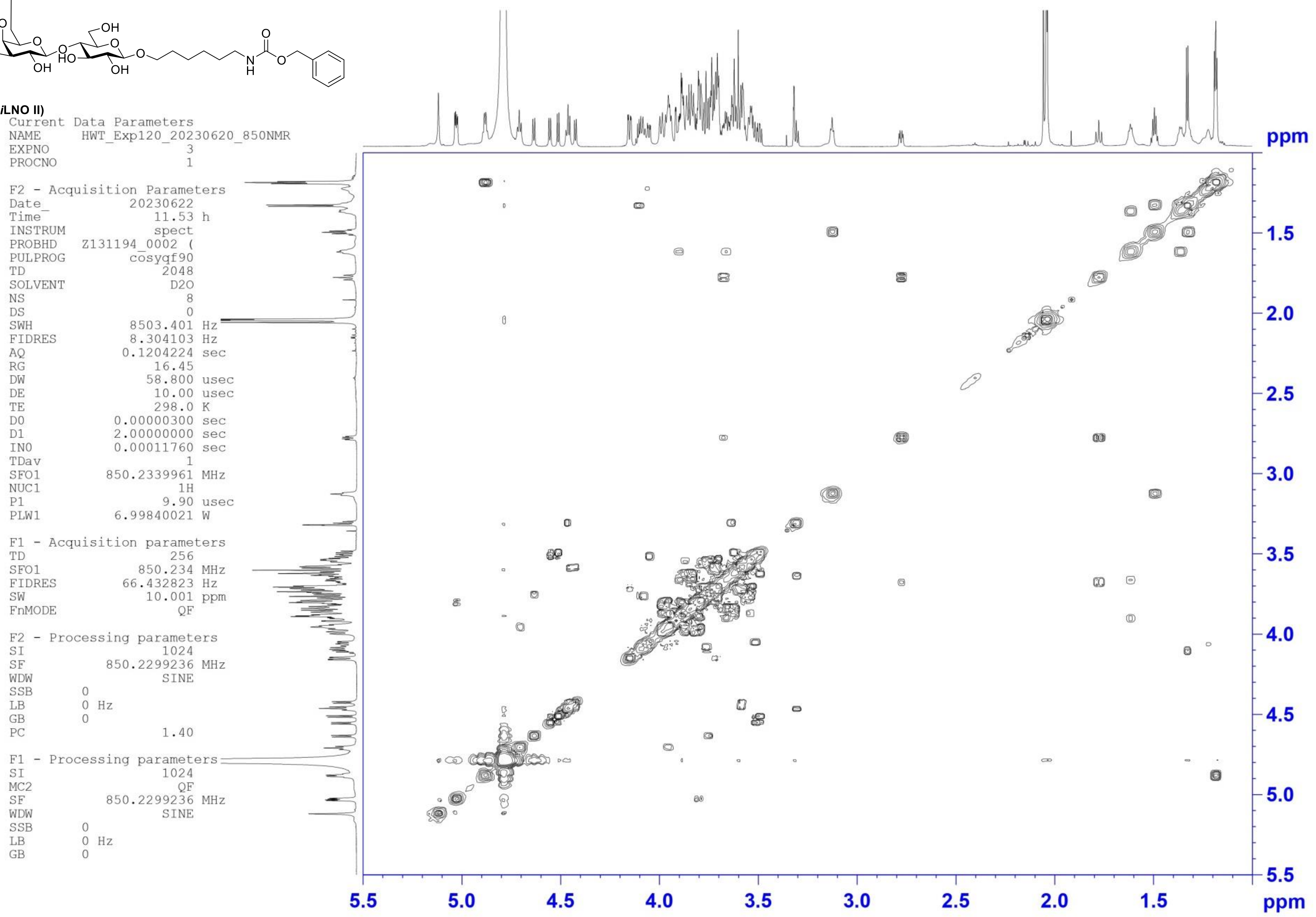

^{1}H-^{1}H COSY NMR spectrum of **49** (DFS-*i*LNO II) (850 MHz, D_2O)

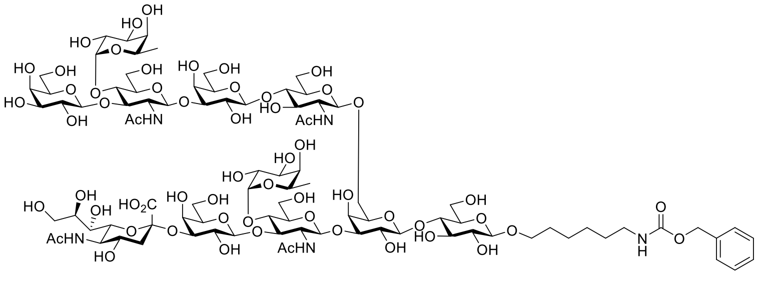

49 (DFS-*i*LNO II)

```
Current Data Parameters
NAME     HWT_Exp120_20230620_850NMR
EXPNO                 4
PROCNO                1

F2 - Acquisition Parameters
Date_          20230622
Time              19.22 h
INSTRUM           spect
PROBHD   Z131194_0002 (
PULPROG  hsqcetgpsisp2.2
TD                 2048
SOLVENT             D2O
NS                   24
DS                   16
SWH          11904.762 Hz
FIDRES       11.625744 Hz
AQ           0.0860160 sec
RG               184.37
DW               42.000 usec
DE                10.00 usec
TE                298.0 K
CNST2       145.0000000
CNST17       -0.5000000
D0           0.00000300 sec
D1           1.50000000 sec
D4           0.00172414 sec
D11          0.03000000 sec
D16          0.00020000 sec
D24          0.00086207 sec
IN0          0.00001060 sec
TDav                  1
SFO1        850.2351014 MHz
NUC1                 1H
P1                 9.90 usec
P2                19.80 usec
P28      0 usec
PLW1         6.99840021 W
SFO2        213.8118826 MHz
NUC2                13C
CPDPRG[2           garp
P3                12.00 usec
P14              500.00 usec
P24             2000.00 usec
PCPD2             50.00 usec
PLW0     0 W
PLW2       130.00000000 W
PLW12        7.48799992 W
SPNAM[3] Crp80,0.5,20.1
SPOAL3            0.500
SPOFFS3  0 Hz
SPW3        38.13600159 W
SPNAM[7]    Crp80comp.4
SPOAL7            0.500
SPOFFS7  0 Hz
SPW7        38.13600159 W
GPNAM[1]     SMSQ10.100
GPZ1              80.00 %
GPNAM[2]     SMSQ10.100
GPZ2              20.10 %
GPNAM[3]     SMSQ10.100
GPZ3              11.00 %
GPNAM[4]     SMSQ10.100
GPZ4              -5.00 %
P16             1000.00 usec
P19              600.00 usec

F1 - Acquisition parameters
TD                  360
SFO1        213.8119 MHz
FIDRES    262.054504 Hz
SW           220.614 ppm
FnMODE   Echo-Antiecho

F2 - Processing parameters
SI                 1024
SF          850.2299264 MHz
WDW               QSINE
SSB                   2
LB       0 Hz
GB       0
PC                 1.40

F1 - Processing parameters
SI                 1024
MC2       echo-antiecho
SF          213.7902922 MHz
WDW               QSINE
SSB                   2
LB       0 Hz
GB       0
```

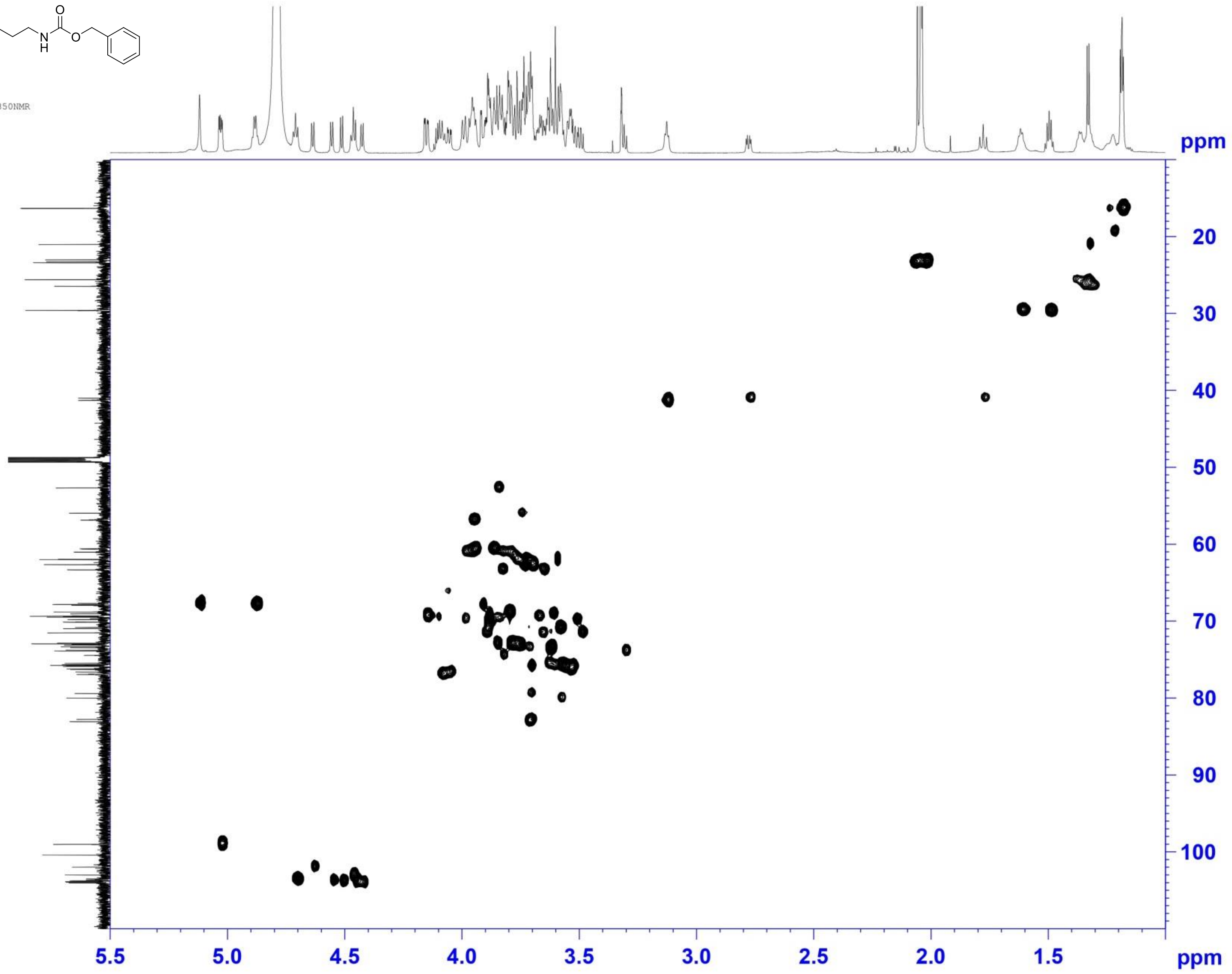

^{1}H-^{13}C HSQC NMR spectrum of **49** (DFS-*i*LNO II) (850 MHz/214 MHz, D_2O)

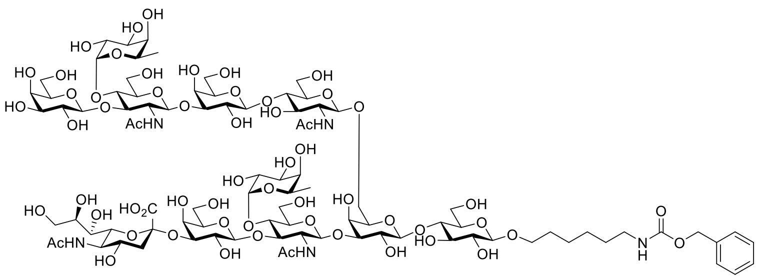

49 (DFS-*i*LNO II)

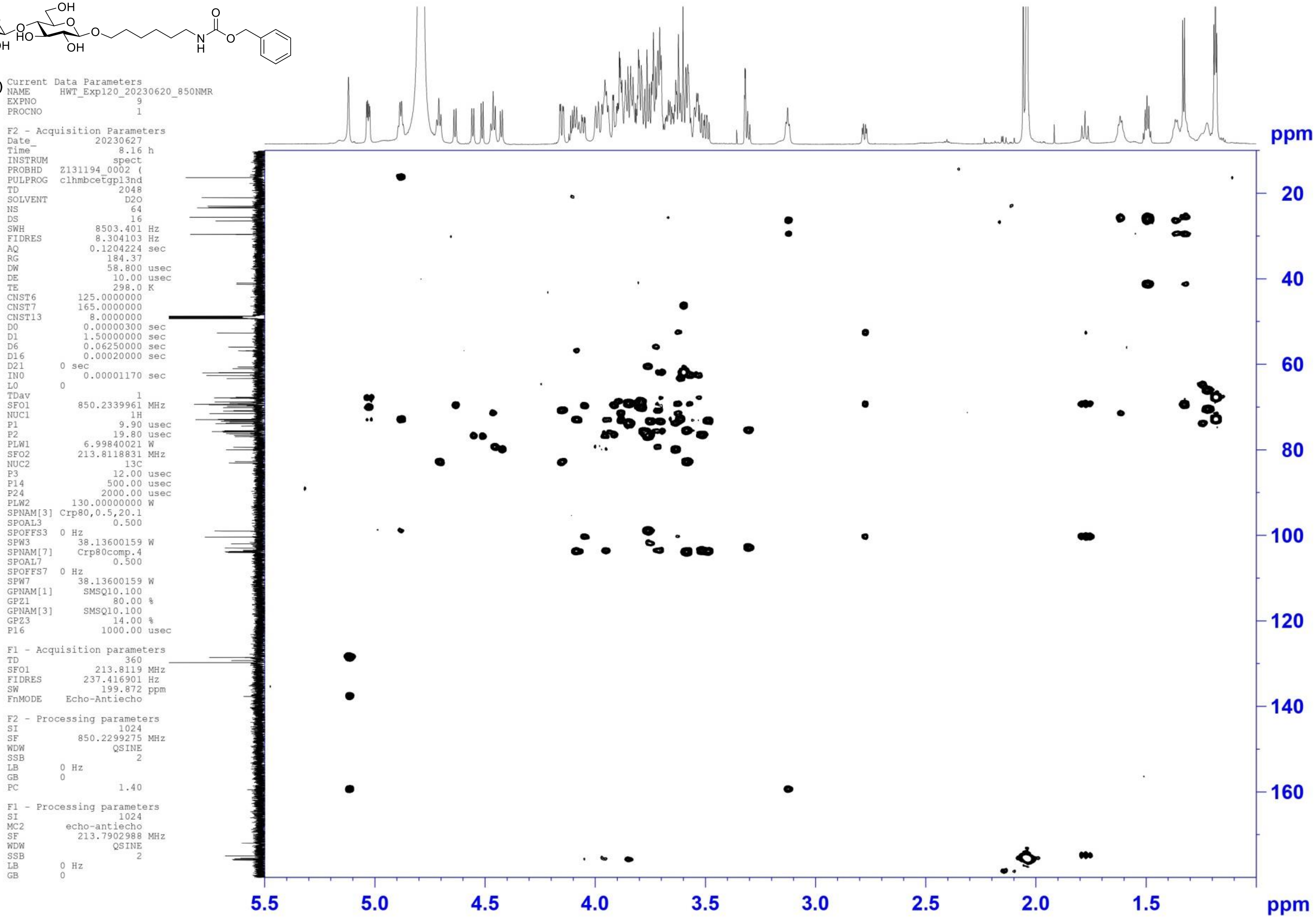

^{1}H-^{13}C HMBC NMR spectrum of **49** (DFS-*i*LNO II) (850 MHz/214 MHz, D_2O)

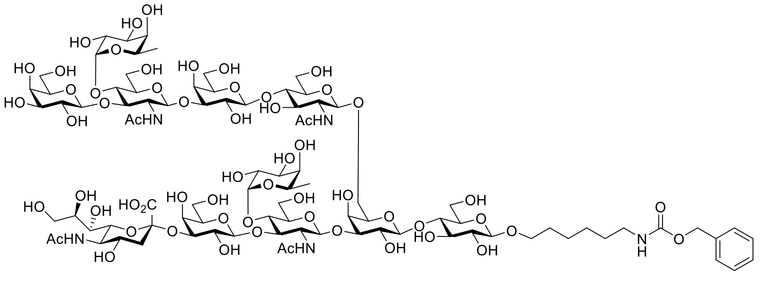

49 (DFS-*i*LNO II)

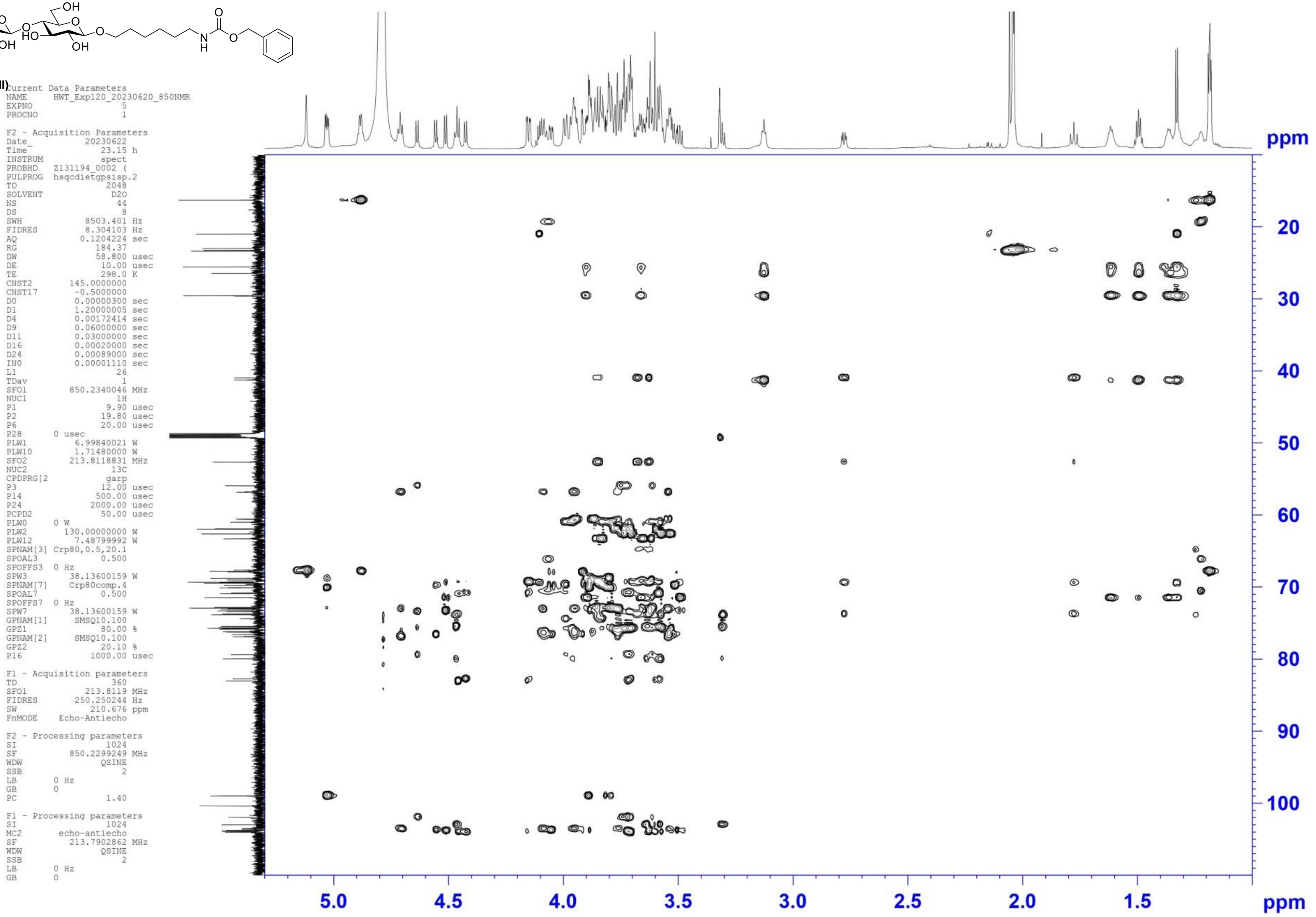

^{1}H-^{13}C HSQC-TOSCY NMR spectrum of **49** (DFS-*i*LNO II) (850 MHz/214 MHz, D_2O)

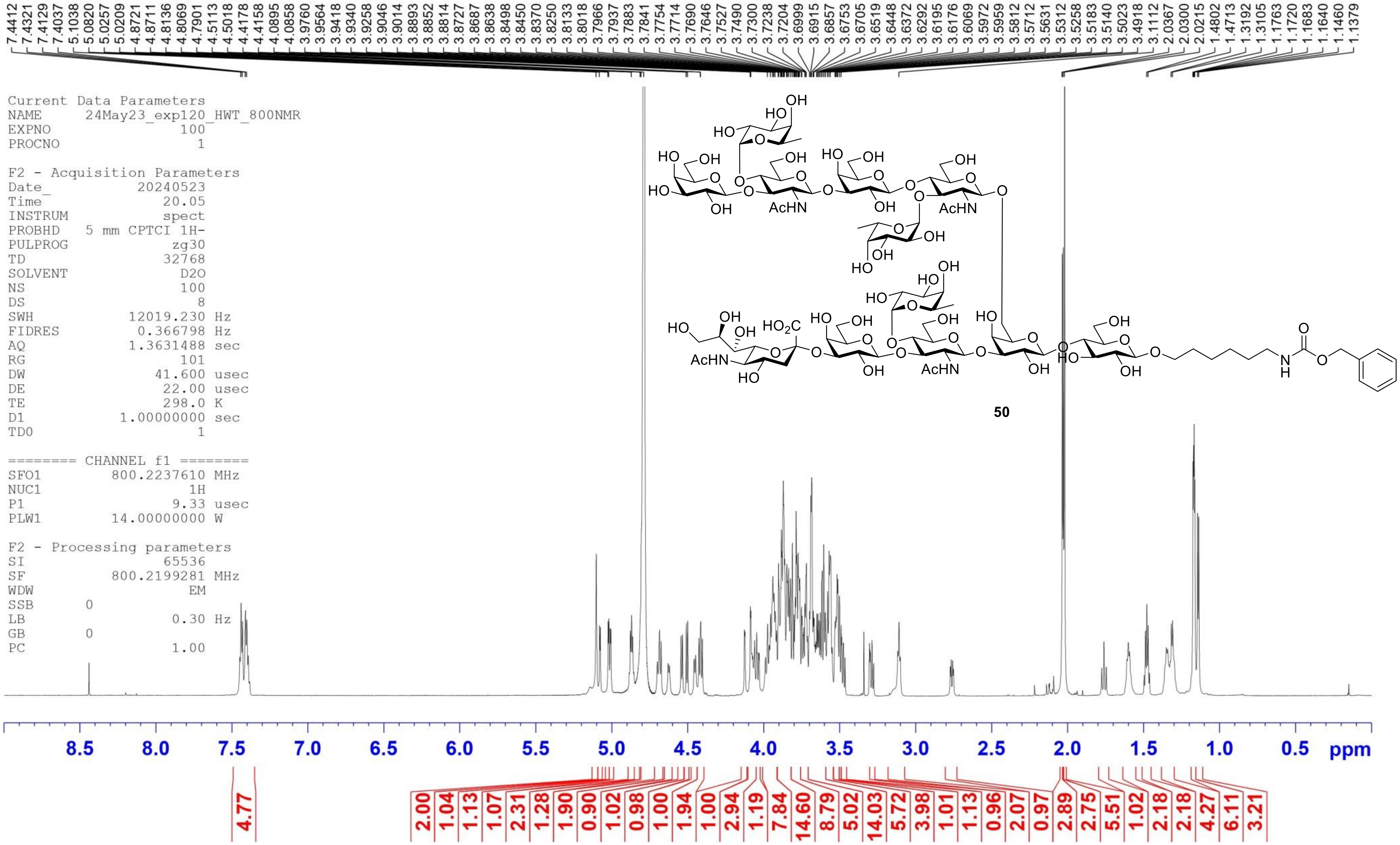

^{1}H NMR spectrum of **50** (800 MHz, D_2O)

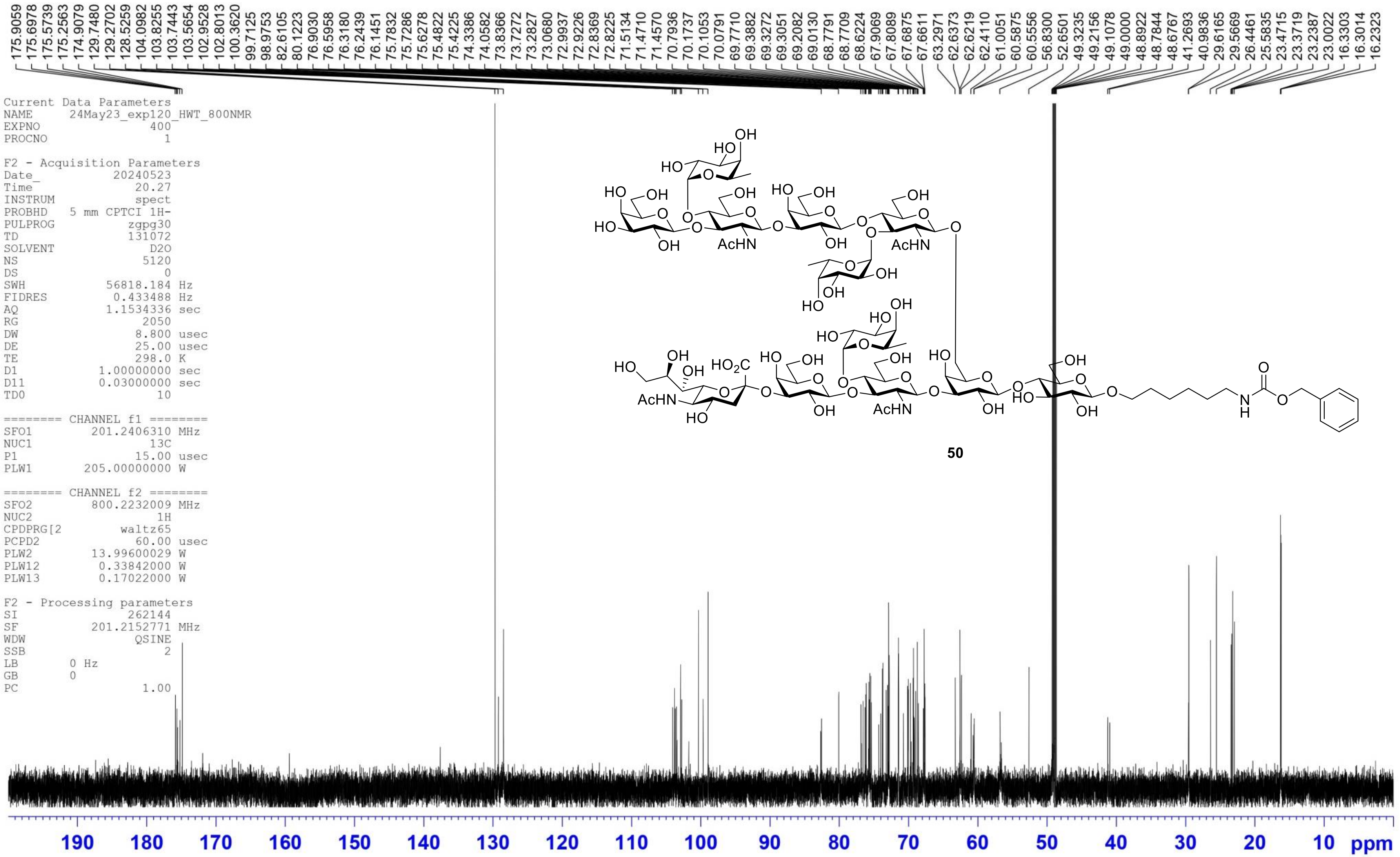

^{13}C NMR spectrum of **50** (201 MHz, D_2O)

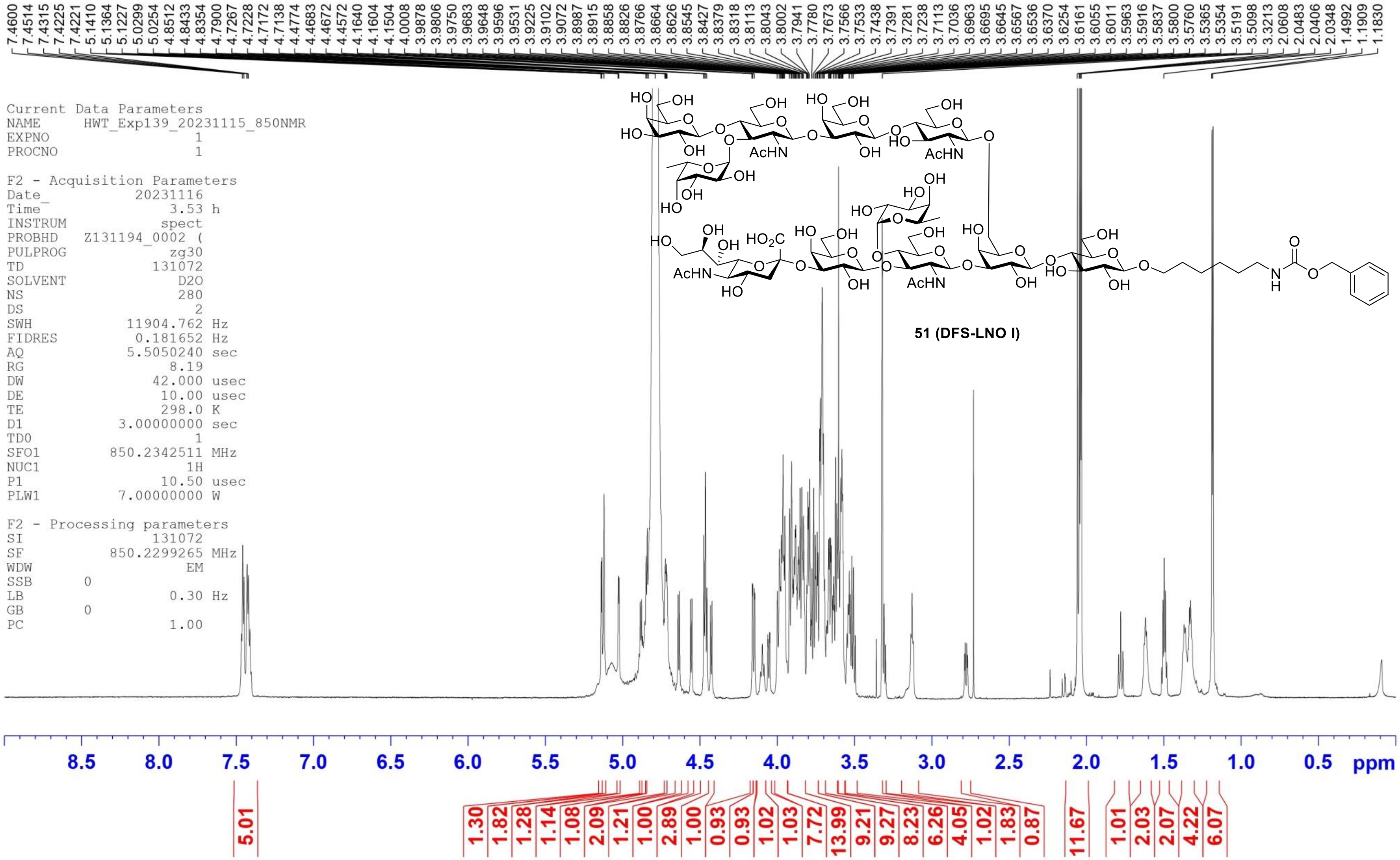

^{1}H NMR spectrum of **51** (DFS-LNO I) (850 MHz, D_2O)

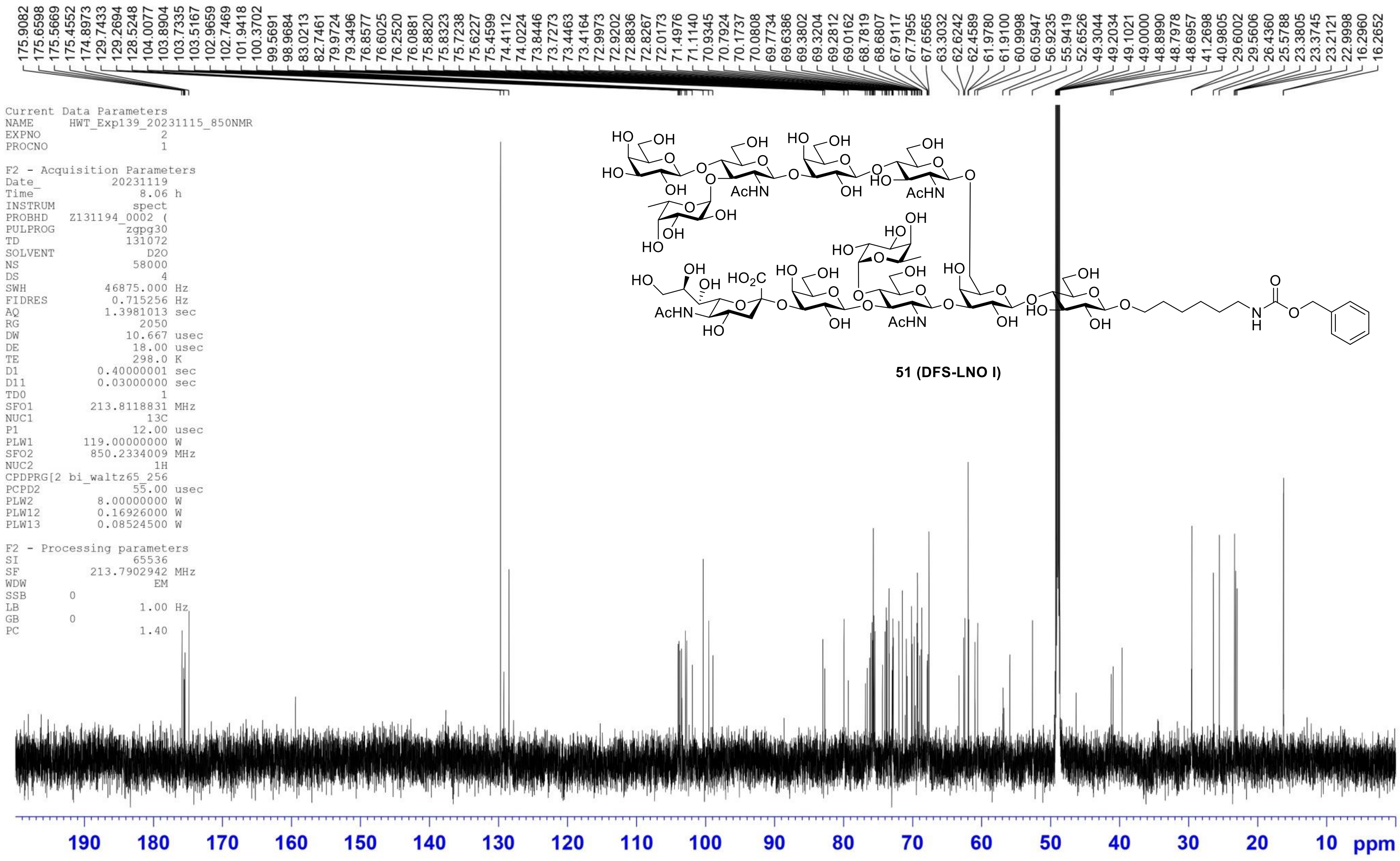

^{13}C NMR spectrum of **51** (DFS-LNO I) (214 MHz, D_2O)

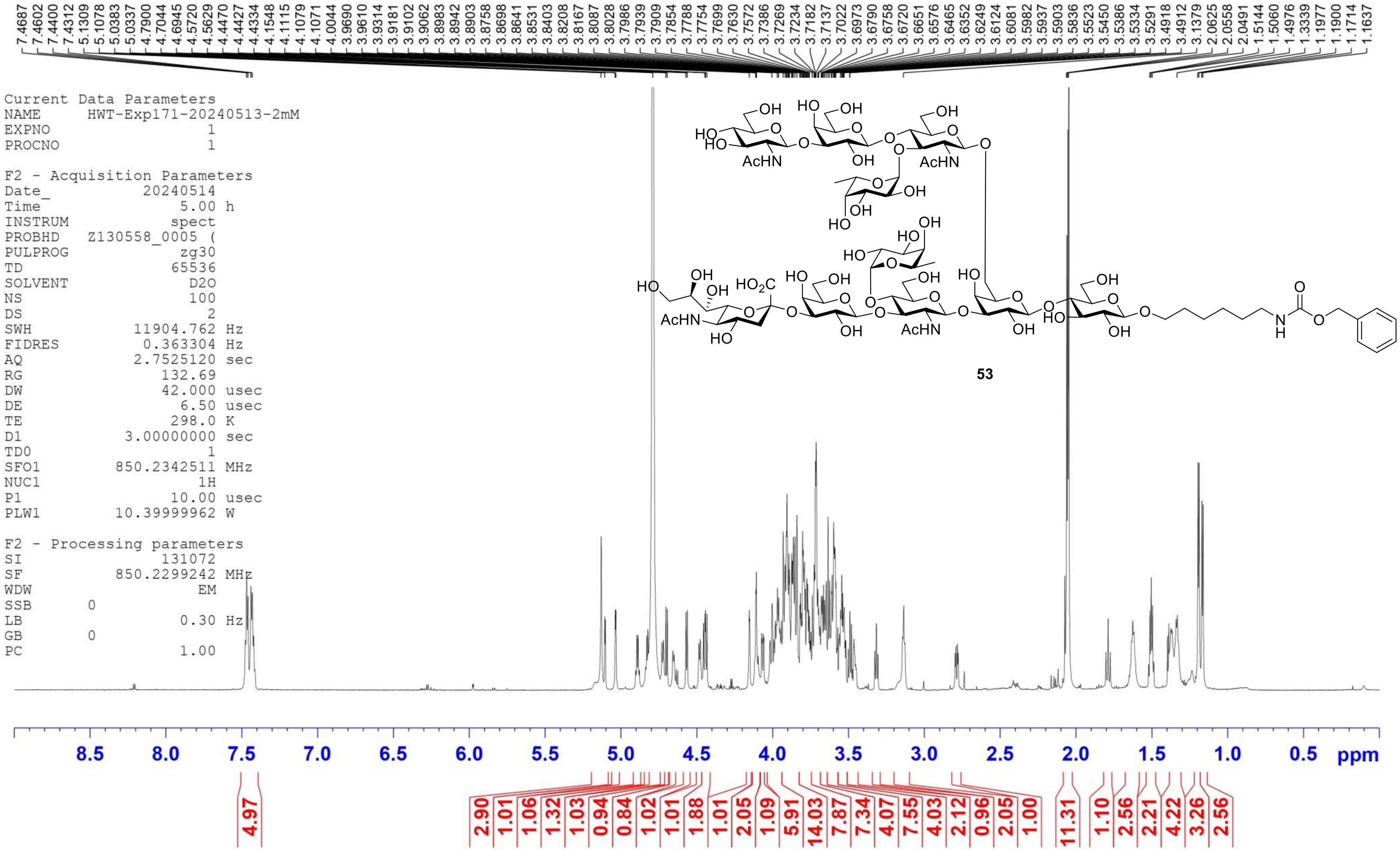

^{1}H NMR spectrum of Compound **53** (850 MHz D_2O)

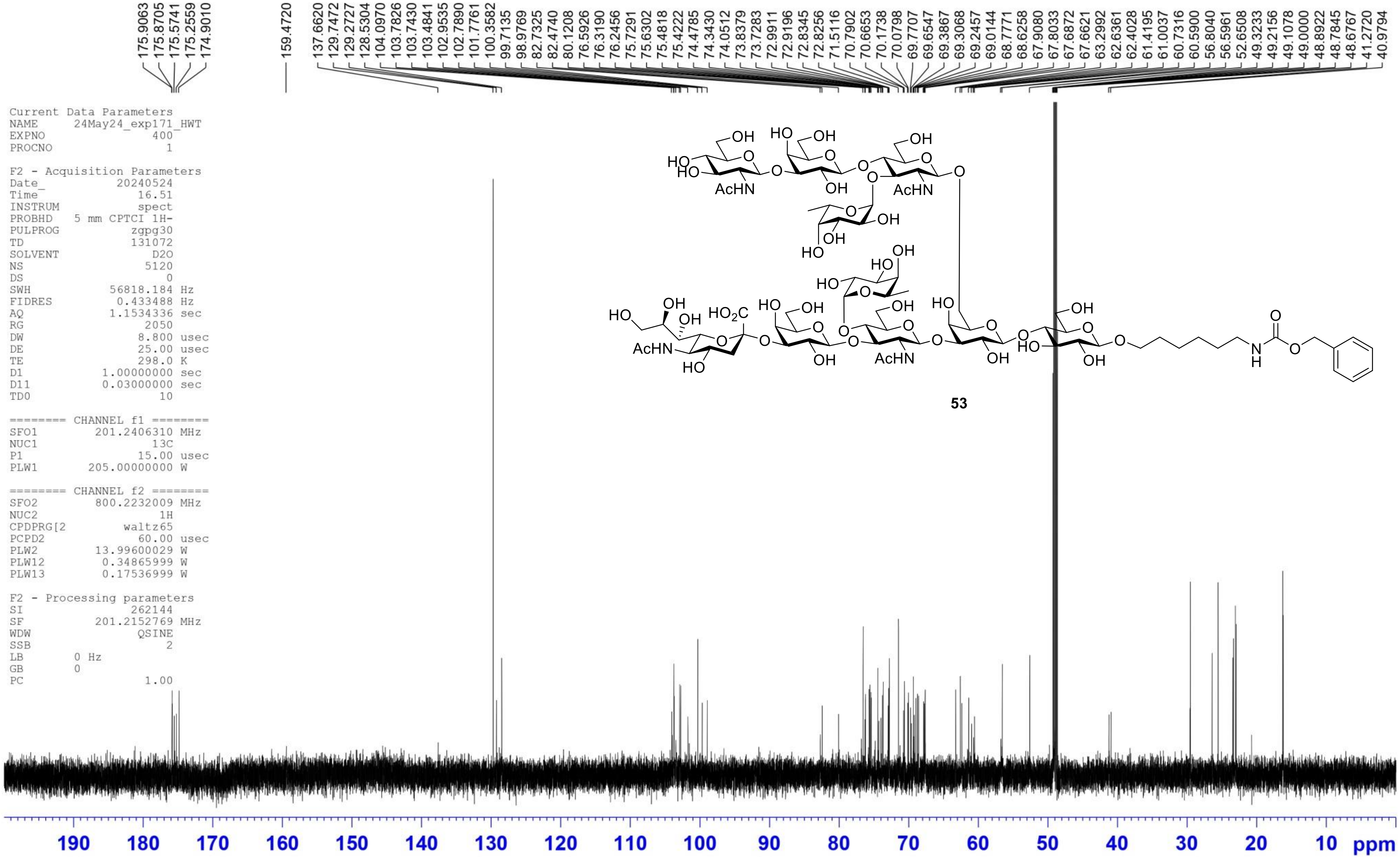

^{13}C NMR spectrum of Compound **53** (201 MHz D_2O)

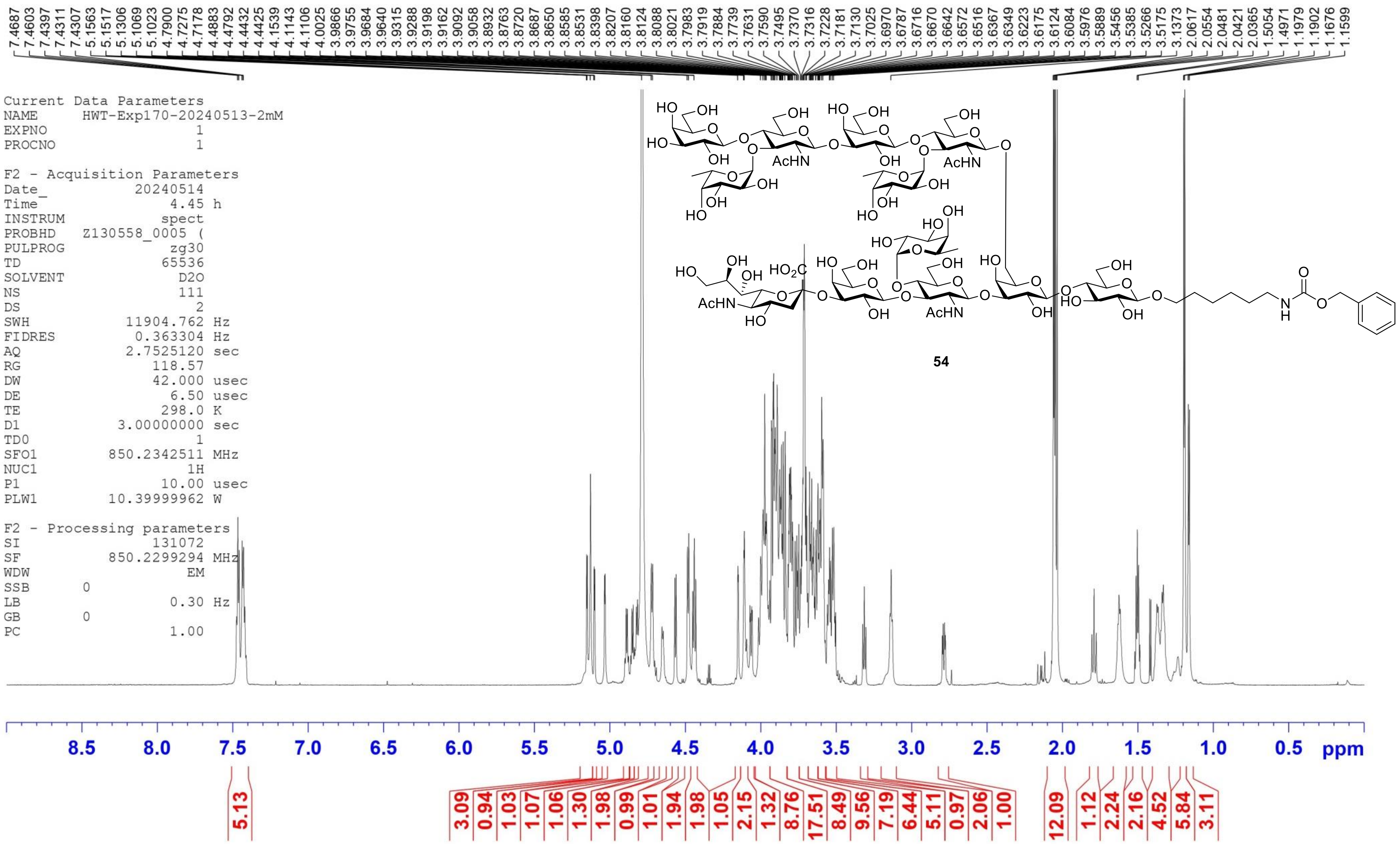

^{1}H NMR spectrum of Compound **54** (850 MHz D_2O)

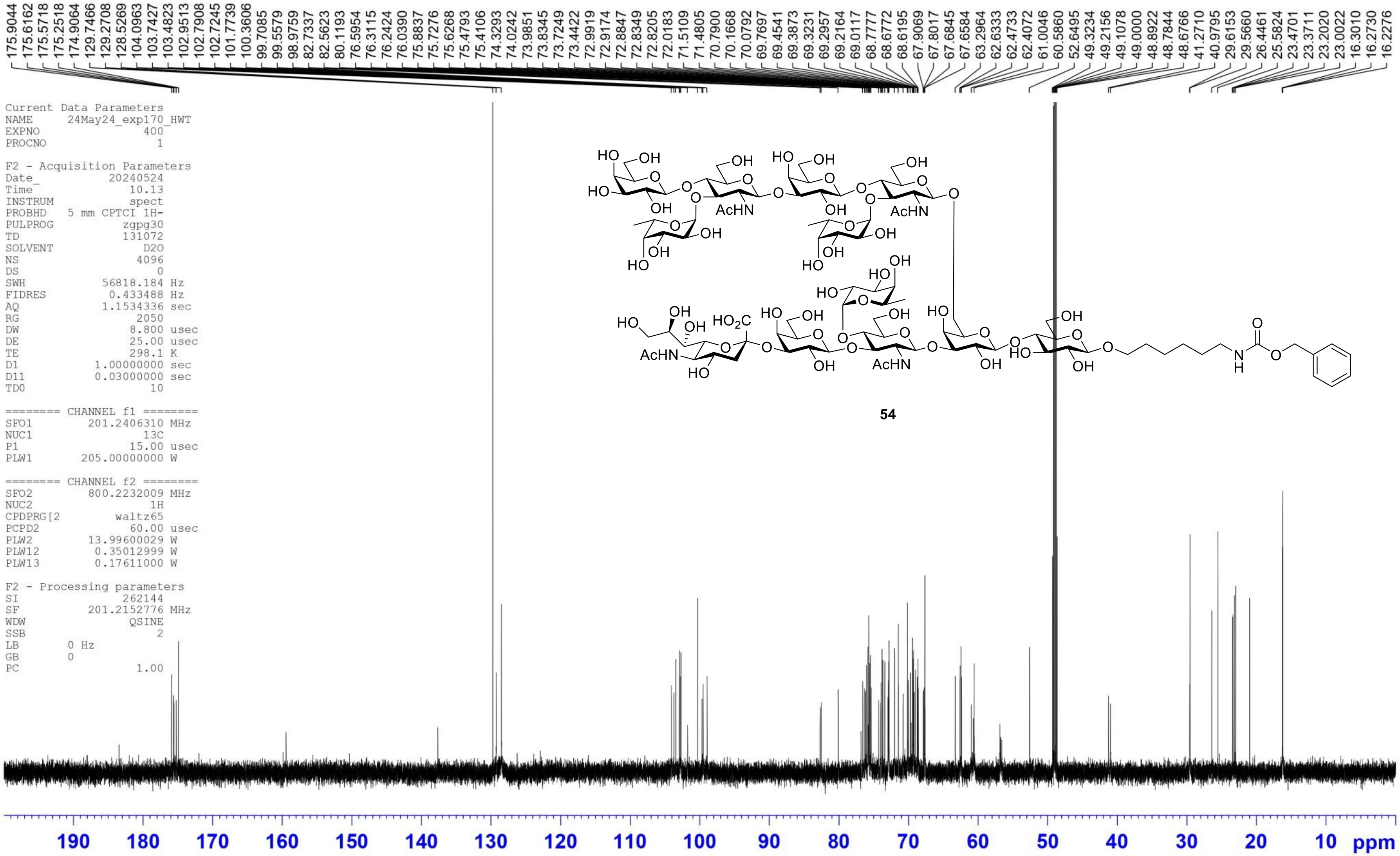

^{13}C NMR spectrum of Compound **54** (201 MHz D_2O)

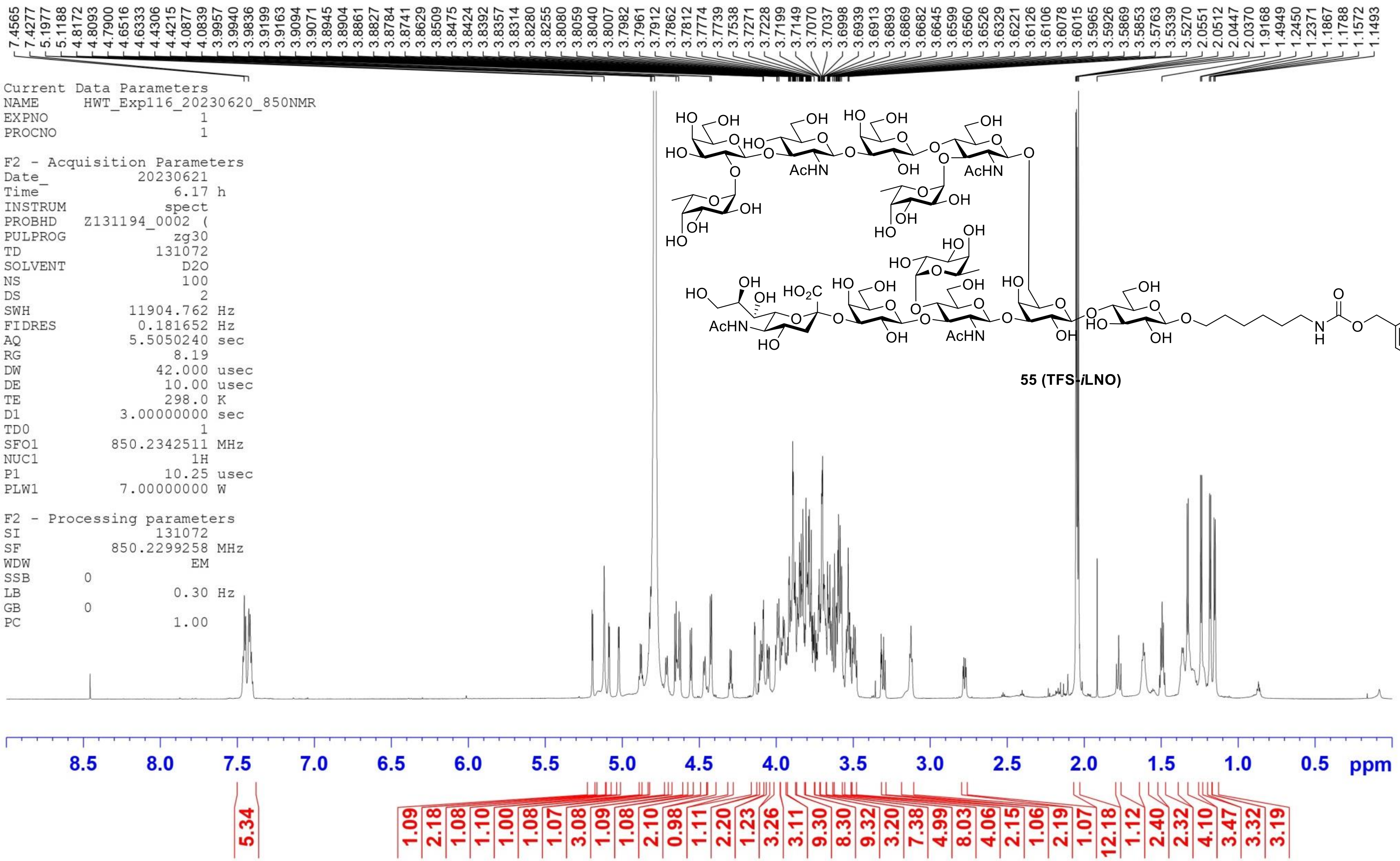

^{1}H NMR spectrum of **55** (TFS-*i*LNO) (850 MHz, D_2O)

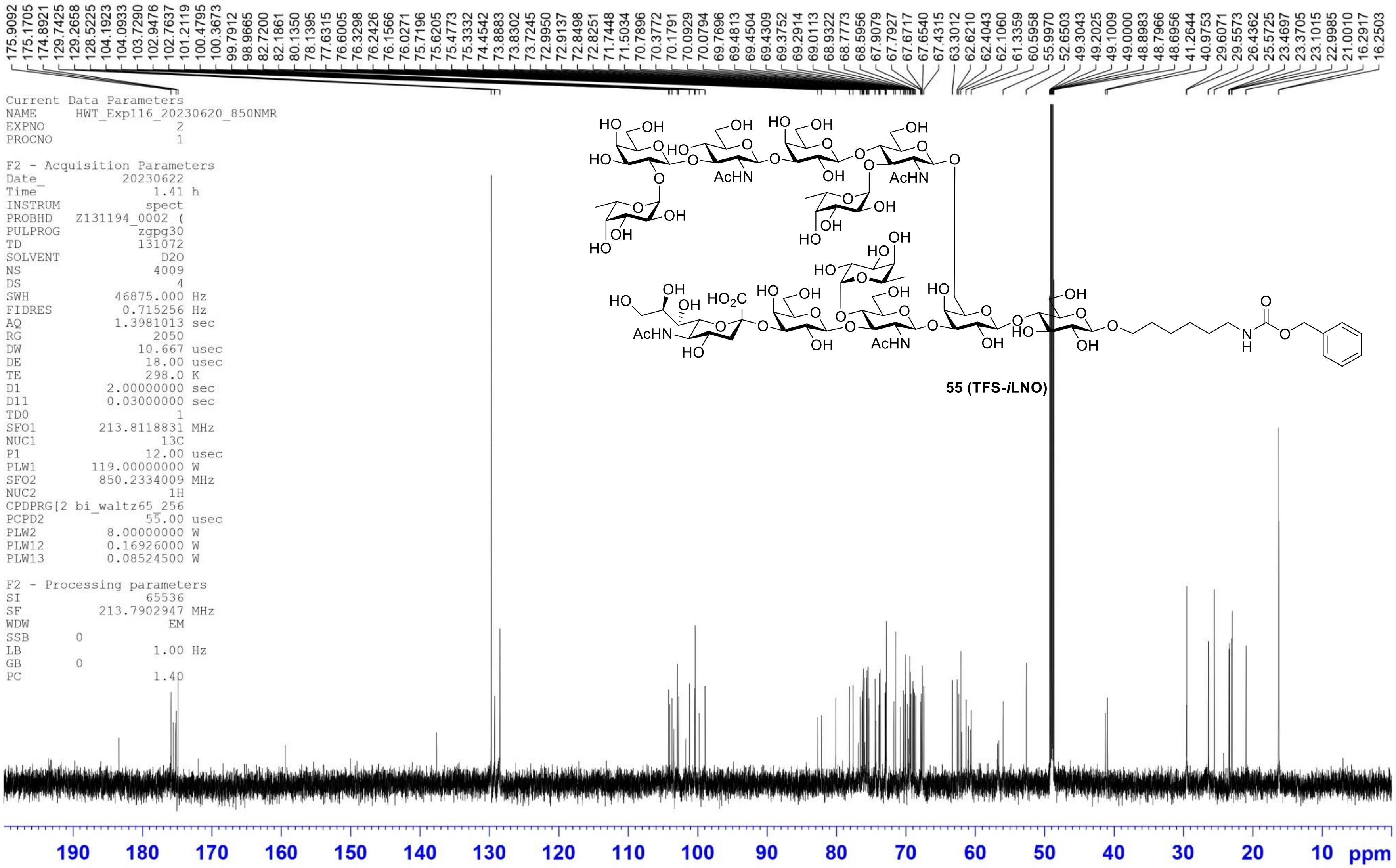

^{13}C NMR spectrum of **55** (TFS-*i*LNO) (214 MHz, D_2O)

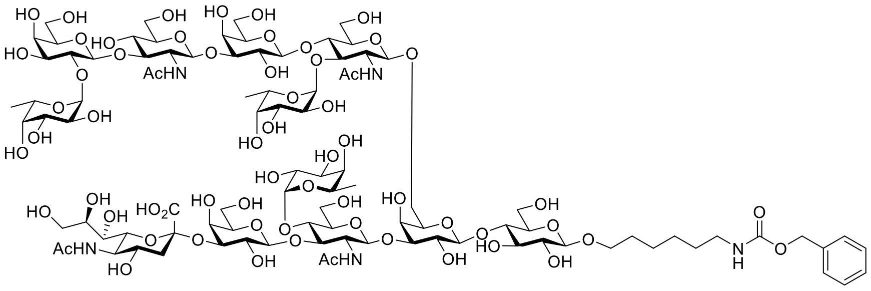

55 (TFS-*i*LNO)

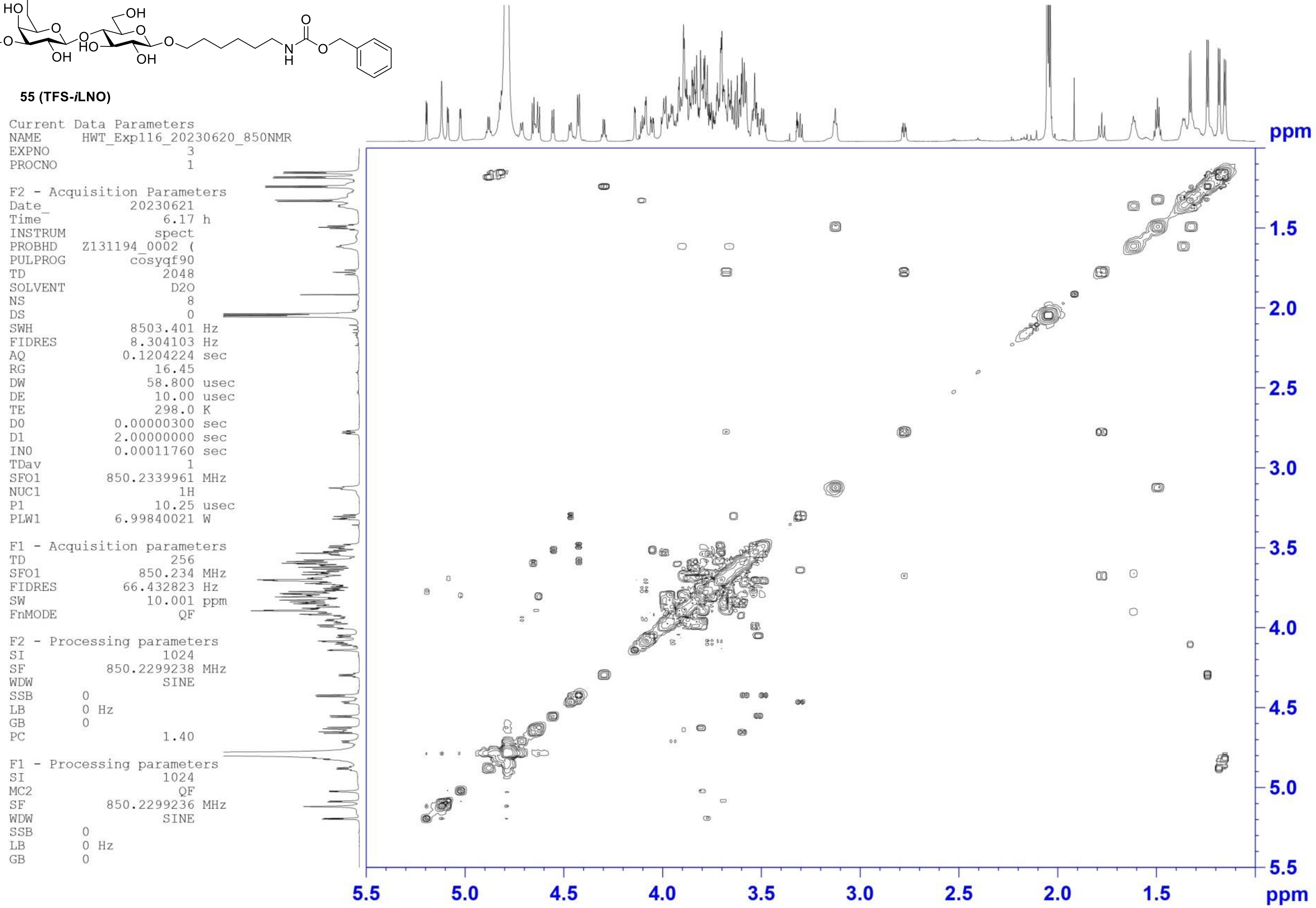

^{1}H-^{1}H COSY NMR spectrum of **55** (TFS-*i*LNO) (850 MHz, D_2O)

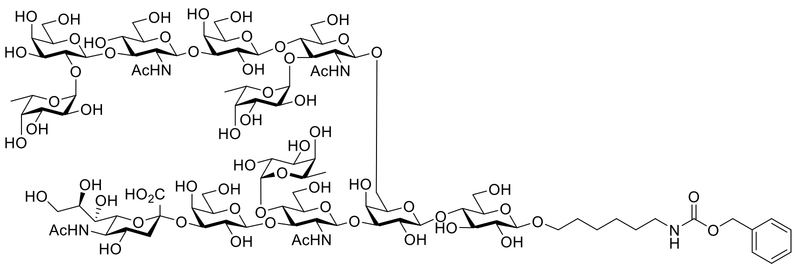

55 (TFS-*i*LNO)

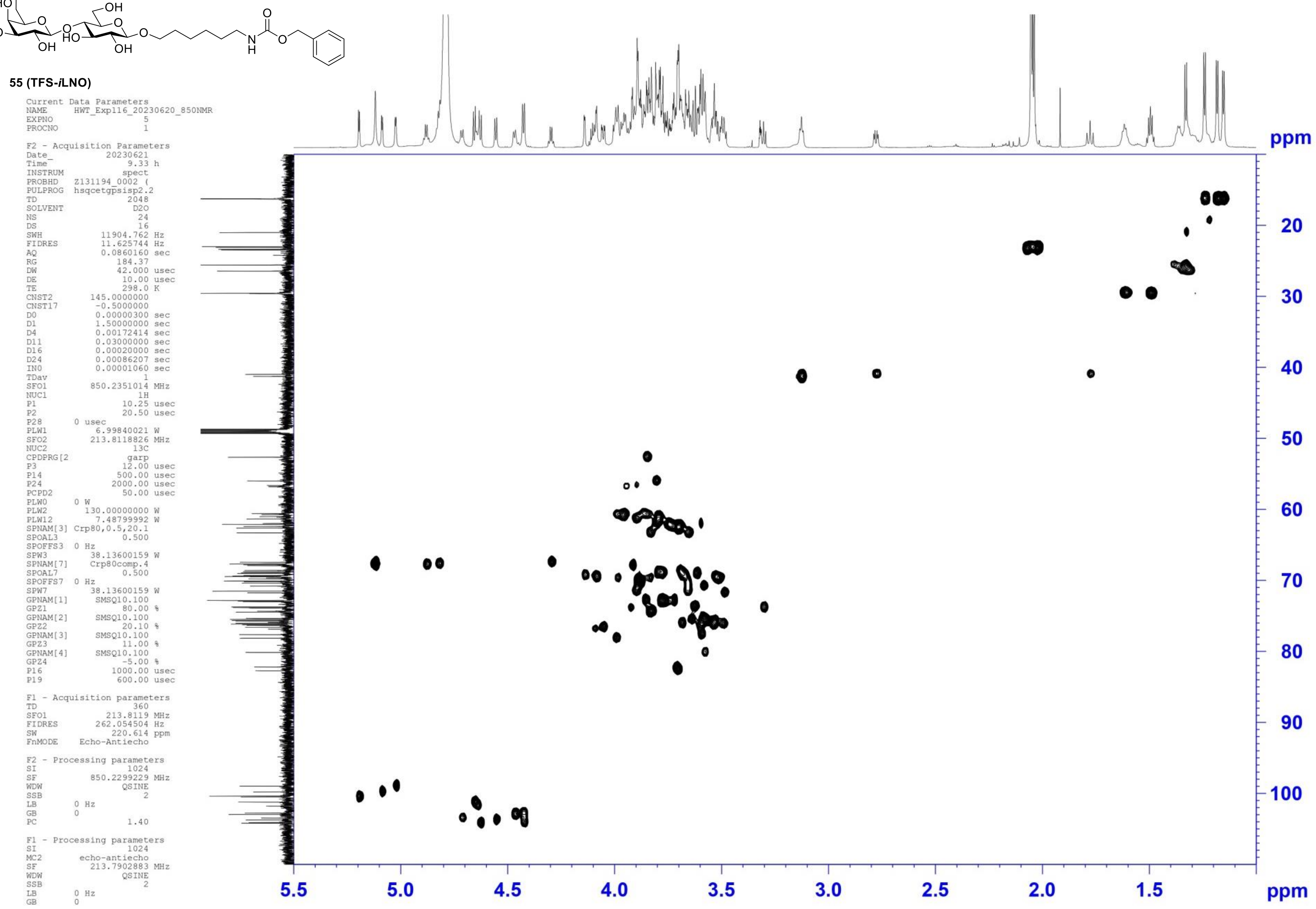

^{1}H-^{13}C HSQC NMR spectrum of **55** (TFS-*i*LNO) (850 MHz/214 MHz, D_2O)

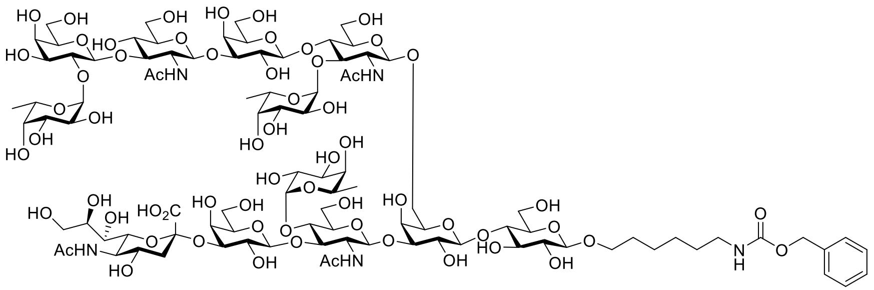

55 (TFS-*i*LNO)

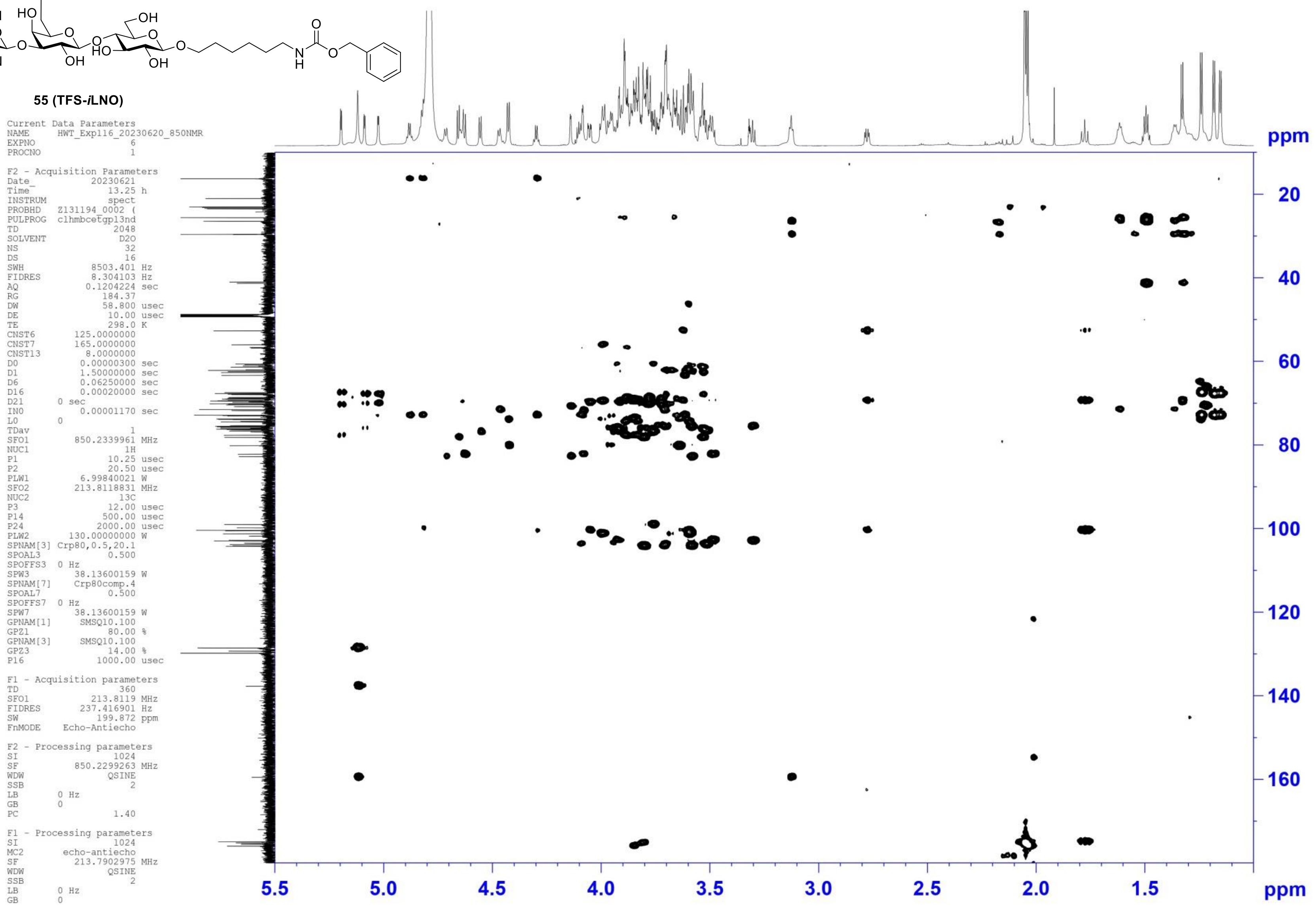

^{1}H-^{13}C HMBC NMR spectrum of **55** (TFS-*i*LNO) (850 MHz/214 MHz, D_2O)

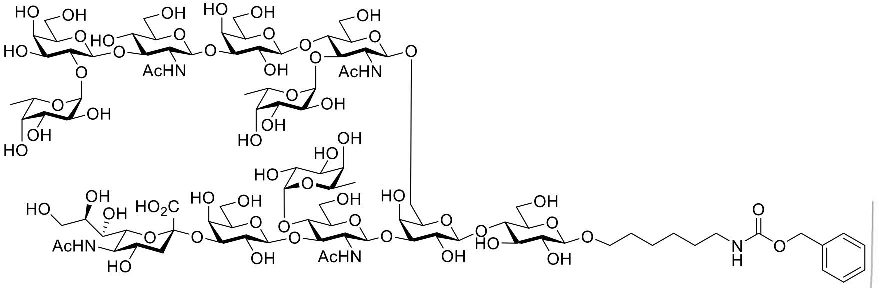

55 (TFS-*i*LNO)

```
Current Data Parameters
NAME     HWT_Exp116_20230620_850NMR
EXPNO                     4
PROCNO                    1

F2 - Acquisition Parameters
Date_              20230621
Time                  18.52 h
INSTRUM               spect
PROBHD   Z131194_0002 (
PULPROG  hsqcdietgpsisp.2
TD                     2048
SOLVENT                 D2O
NS                       32
DS                        8
SWH                8503.401 Hz
FIDRES             8.304103 Hz
AQ                0.1204224 sec
RG                   184.37
DW                   58.800 usec
DE                    10.00 usec
TE                    298.0 K
CNST2       145.0000000
CNST17       -0.5000000
D0           0.00000300 sec
D1           1.20000005 sec
D4           0.00172414 sec
D9           0.06000000 sec
D11          0.03000000 sec
D16          0.00020000 sec
D24          0.00089000 sec
IN0          0.00001110 sec
L1                       26
TDav                      1
SFO1        850.2340046 MHz
NUC1                     1H
P1                    10.25 usec
P2                    20.50 usec
P6                    20.00 usec
P28      0 usec
PLW1         6.99840021 W
PLW10        1.83819997 W
SFO2        213.8118831 MHz
NUC2                    13C
CPDPRG[2               garp
P3                    12.00 usec
P14                  500.00 usec
P24                 2000.00 usec
PCPD2                 50.00 usec
PLW0     0 W
PLW2       130.00000000 W
PLW12        7.48799992 W
SPNAM[3] Crp80,0.5,20.1
SPOAL3                0.500
SPOFFS3  0 Hz
SPW3        38.13600159 W
SPNAM[7]     Crp80comp.4
SPOAL7                0.500
SPOFFS7  0 Hz
SPW7        38.13600159 W
GPNAM[1]      SMSQ10.100
GPZ1                  80.00 %
GPNAM[2]      SMSQ10.100
GPZ2                  20.10 %
P16                 1000.00 usec

F1 - Acquisition parameters
TD                      360
SFO1           213.8119 MHz
FIDRES       250.250244 Hz
SW              210.676 ppm
FnMODE    Echo-Antiecho

F2 - Processing parameters
SI                     1024
SF          850.2299242 MHz
WDW                   QSINE
SSB                       2
LB       0 Hz
GB       0
PC                     1.40

F1 - Processing parameters
SI                     1024
MC2          echo-antiecho
SF          213.7902867 MHz
WDW                   QSINE
SSB                       2
LB       0 Hz
GB       0
```

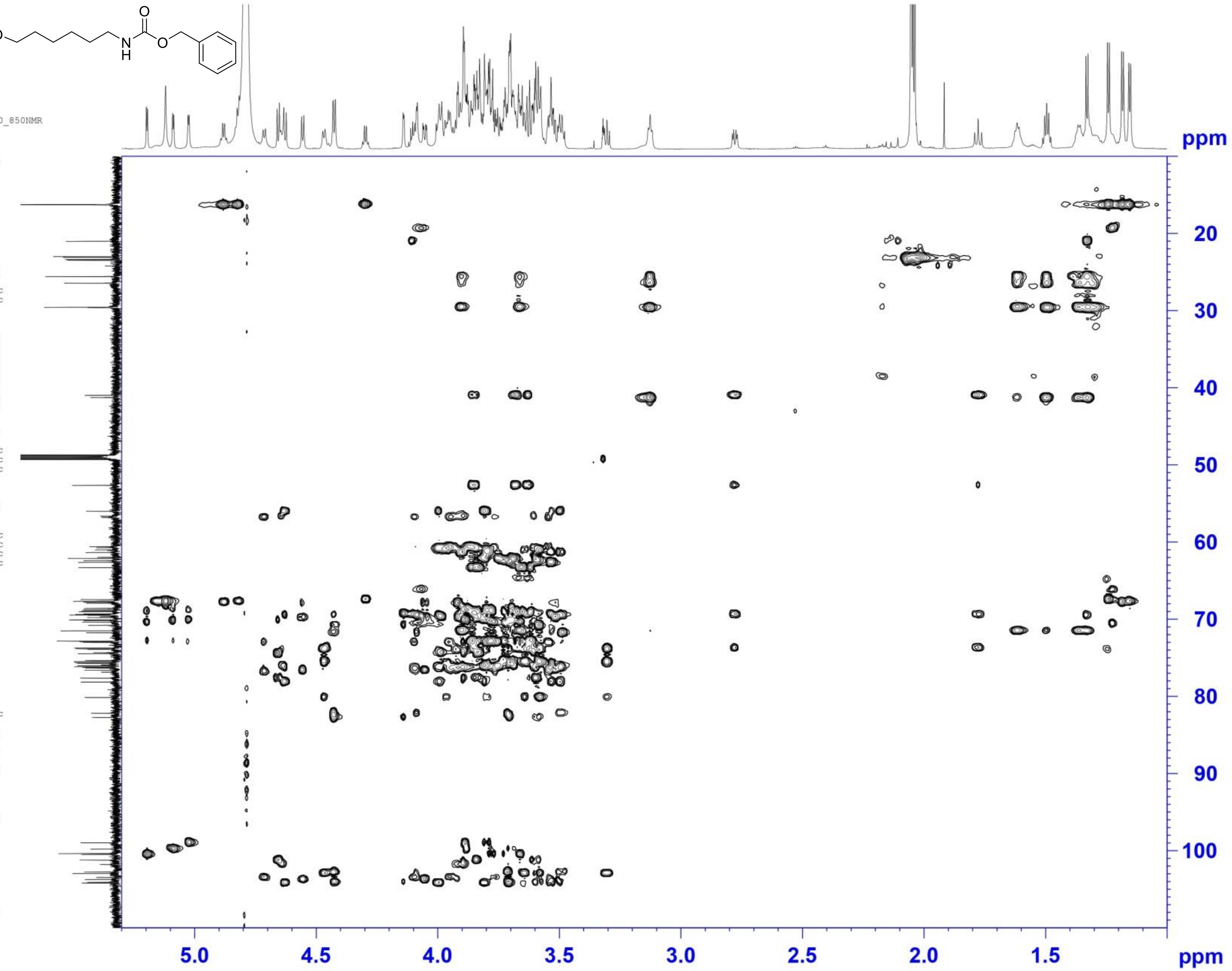

^{1}H-^{13}C HSQC-TOSCY NMR spectrum of **55** (TFS-*i*LNO) (850 MHz/214 MHz, D_2O)
